# Evolutionary Fate of the Androgen Receptor−Signaling Pathway in Ray-Finned Fishes with a Special Focus on Cichlids

**DOI:** 10.1534/g3.115.020685

**Published:** 2015-09-01

**Authors:** Thibault Lorin, Walter Salzburger, Astrid Böhne

**Affiliations:** * ENS (Ecole Normale Supérieure de Lyon), Lyon Cedex 07, France; † Zoological Institute, Department of Environmental Sciences, University of Basel, 4051 Basel, Switzerland

**Keywords:** androgen receptor, ray-finned fish, cichlids, genome duplication, sequence evolution

## Abstract

The emergence of the steroid system is coupled to the evolution of multicellular animals. In vertebrates in particular, the steroid receptor repertoire has been shaped by genome duplications characteristic to this lineage. Here, we investigate for the first time the composition of the androgen receptor–signaling pathway in ray-finned fish genomes by focusing in particular on duplicates that emerged from the teleost-specific whole-genome duplication. We trace lineage- and species-specific duplications and gene losses for the genomic and nongenomic pathway of androgen signaling and subsequently investigate the sequence evolution of these genes. In one particular fish lineage, the cichlids, we find evidence for differing selection pressures acting on teleost-specific whole-genome duplication paralogs at a derived evolutionary stage. We then look into the expression of these duplicated genes in four cichlid species from Lake Tanganyika indicating, once more, rapid changes in expression patterns in closely related fish species. We focus on a particular case, the cichlid specific duplication of the *rac1* GTPase, which shows possible signs of a neofunctionalization event.

Sex steroid hormones (androgens, estrogens, and progestogens) are involved in physiological processes as different as reproduction (Wierman 2007), the establishment of sex-specific traits [*e.g.*, pigmentation (Lindsay *et al.* 2011)], communication (Arch and Narins 2009), parental care (Dey *et al.* 2010), the immune system (García-Gómez *et al.* 2013), and even vision (Shao *et al.* 2014). Androgens, and particularly the major circulating form testosterone, were long thought of as having a predominant male function, whereas estrogens often are thought of as female hormones; however, more and more evidence is accumulating that both types of sex hormones are present in the circulating blood of both sexes (*e.g.*, Coumailleau *et al.* 2015), which is not least due to the fact that testosterone can be hydroxylated by the enzyme aromatase into estrogen.

Steroid-synthesizing enzymes and steroid hormone receptors have an ancient origin within the animal kingdom. The emergence of nuclear receptors, for example, is going along with the evolution of multicellular animals (Baker *et al.* 2015); estrogen signaling is already present in amphioxus (Callard *et al.* 2011). The true androgen receptor evolved only later in gnathostomes after their split from agnathans, which already show reactivity to androgens probably mediated by other receptors (Guerriero 2009 and references therein).

The sex steroid signaling system has been shaped throughout the evolutionary history of vertebrates by two whole-genome duplication events, characterizing this lineage (Dehal and Boore 2005). Although there is a decent understanding of the pathway’s general evolutionary origin, one particular vertebrate group (teleost fish) asks for a deeper investigation for at least two reasons. First of all, the teleost fish lineage represents, with ∼30,000 described species, the largest and most diverse group of vertebrates (Nelson 2006). Second, this lineage experienced an additional whole-genome duplication event approximately 320−350 million years ago, also known as the teleost-specific whole-genome duplication (TSGD) (Meyer and Van De Peer 2005), which generated extra genomic material not present in other vertebrate groups.

One evolutionary fate of this extra genetic material is neofunctionalization of one of the paralogs, an event that has actually been proposed for the B-paralog of the androgen receptor in teleosts (Douard *et al.* 2008).

Here we set out to study the androgen receptor (AR) and its pathway in the teleost lineage with the aim to better understand its genomic composition shaped by gene and genome duplications. We investigate selection pressures acting on this pathway and examine gene expression profiles in a particularly diverse group of fishes, the East African cichlids (Santos and Salzburger 2012; Brawand *et al.* 2014).

The AR is a cytosolic protein that, upon fixation of a ligand, undergoes a conformational change and dissociates from chaperone proteins, dimerizes, and can translocate into the nucleus. The receptor subsequently binds to specific target sequences called androgen response elements and acts as a transcription factor together with coactivators or corepressors on specific target genes. This “classic” or “genomic signaling” is described as the general pathway of nuclear receptors (Figure 1, left panel).

**Figure 1:**
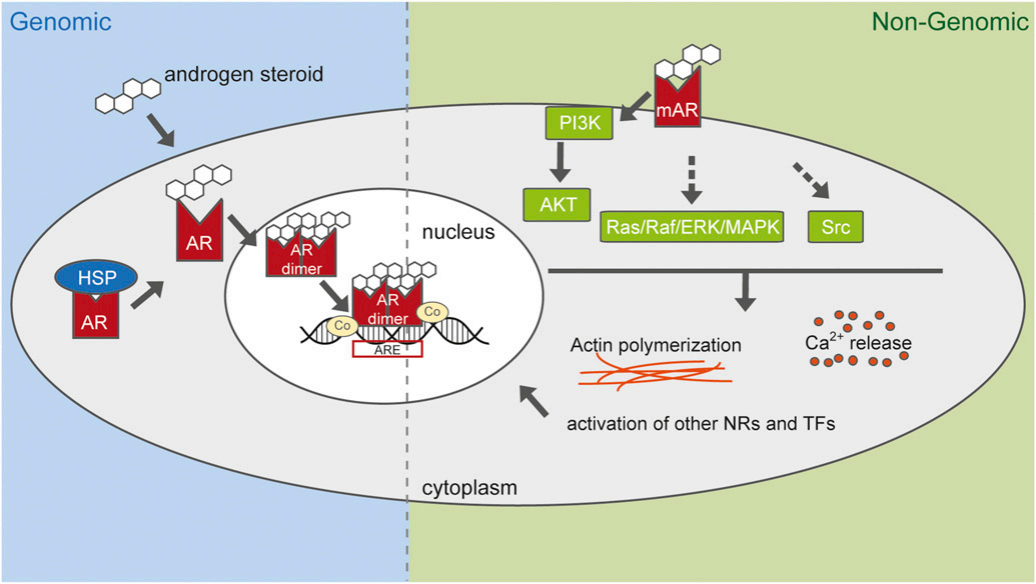
In the classic or genomic pathway, androgens can move in the cytoplasm, where they bind to the AR. This induces a conformational change in AR, releasing it from heat shock proteins. AR then translocates to the nucleus, where it dimerizes, interacts with cofactors (activators or repressors of transcription), and binds to androgen response elements, where it regulates expression of target genes. Nongenomic androgen actions are regulated over a membrane-bound AR or other receptors, which then transduce the signal into the cytoplasm, interacting directly with other membrane bound factors such as PI3K or activating other pathways, which lead to changes in second messengers, cytoplasmic calcium concentration, actin skeleton changes, and eventually also to the activation of other nuclear receptors and transcription factors. AKT, serine/threonine-protein kinase; AR, androgen receptor; ARE, androgen response element; Co, cofactors of AR, either activators or repressors of transcription; HSP, heat shock protein; mAR, membrane-bound AR; NR, nuclear receptor; PI3K, phosphatidylinositol 4,5-bisphosphate 3-kinase; Ras/Raf/ERK/MAPK, kinase signaling pathway; Src, proto-oncogene tyrosine-protein kinase; TF, transcription factor. Figure based on (Foradori *et al.* 2008; Guerriero 2009; Bennett *et al.* 2010; Kandasamy *et al.* 2010).

Alternative signaling of steroids not immediately involving gene transcription can occur via a “nongenomic” or “extranuclear pathway,” implying a membrane and cytosolic protein cascade (reviewed in Foradori *et al.* 2008; Bennett *et al.* 2010) (Figure 1, right panel). This nongenomic response involves the rapid induction of second-messenger signal transduction cascades. For AR, they include the release of intracellular calcium, the activation of protein kinases such as extracellular signal-regulated protein kinases 1/2, protein kinase A, protein kinase C, and Akt, as well as actin cytoskeleton reorganization (Bennett *et al.* 2010 and references therein). These cascades usually trigger actions within seconds or minutes (Yamada 1979; Christian *et al.* 2000), whereas the activation of transcription usually peaks several hours after steroid exposure (Cato *et al.* 1988), although the latency for transcription has been reported to be as short as 7.5 minutes (Groner *et al.* 1983); however, the translational process then still may require hours.

To complete the picture of AR-signaling in teleosts we here investigate the described nongenomic and genomic cascades including orthologs to all human genes associated with the Gene Ontology term GO:0030521 “androgen receptor signaling pathway” and adding the nongenomic pathways as described in NetPath [(Kandasamy *et al.* 2010) and (Foradori *et al.* 2008; Bennett *et al.* 2010)]. The exhaustive gene list is given in Supporting Information, Table S1. We were particularly interested in duplicates derived from the TSGD and their evolution in cichlid fishes.

## Materials and Methods

### *In silico* screening of teleost genomes

We initially searched spotted gar (*Lepisosteus oculatus*) orthologs to the human genes listed in Table S1 in the genome accessible over Ensembl (www.ensembl.org, Version 79). Subsequently, the spotted gar sequences were used as query in two searches. First, we exported all annotated Ensembl orthologs for each gene for the following teleost fish species: zebrafish (*Danio rerio*), cavefish (*Astyanax mexicanus*), platyfish (*Xiphophorus maculatus*), Amazon molly (*Poecilia formosa*), fugu (*Takifugu rubripes*), green spotted puffer (*Tetraodon nigroviridi*s), three-spined stickleback *(Gasterosteus aculeatus*), medaka (*Oryzias latipes*), and Nile tilapia (*Oreochromis niloticus*). If this search did not retrieve an ortholog for the given species, we used the spotted gar coding sequence of the corresponding gene in a second search with BLASTn (search sensitivity “Normal”) against the genome of the species of interest in Ensembl. If this again did not retrieve an ortholog for the species of interest, we extended our BLASTn search to Genbank (default settings).

Additionally, we used the sequences of the Nile tilapia as query in a BLASTn search against the available East African cichlid genomes/transcriptomes of *Neolamprologus brichardi*, *Astatotilapia/Haplochromis burtoni*, *Pundamilia nyererei*, and *Maylandia zebra* in Genbank at http://blast.ncbi.nlm.nih.gov/Blast.cgi.

If orthologous blast hits were found outside annotated genomic regions, coding sequences were annotated manually; in this case chromosome or scaffold information is given (Table S1).

**Figure 2:**
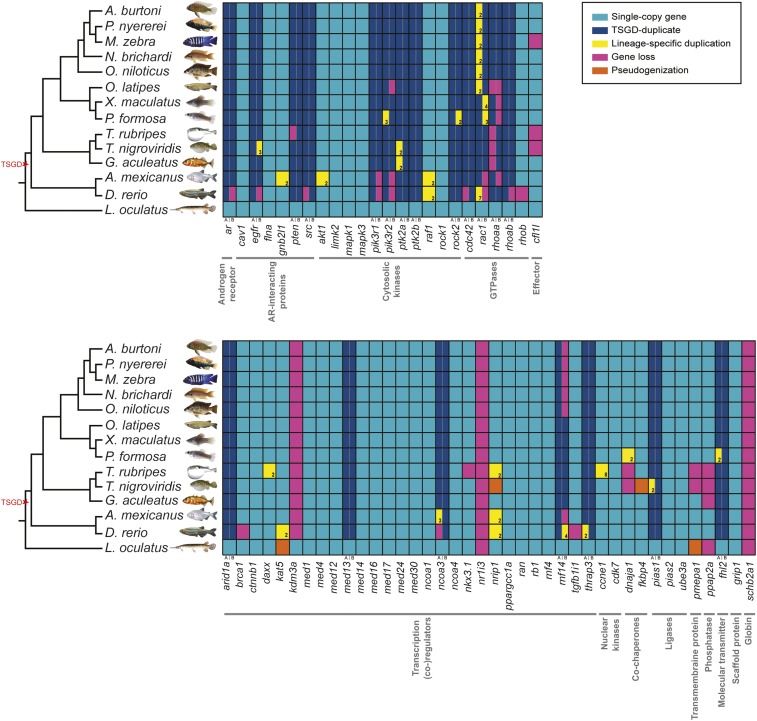
Genes belonging to the androgen receptor−signaling pathway in ray-finned fish genomes. Indicated are all genes and their paralogs in the available teleost fish genomes and the out-group spotted gar. A and B denote duplicates from the teleost-specific whole-genome duplication (TSGD), which took place at the basis of this fish lineage. Genes are grouped according to the nongenomic (upper panel) and genomic (low panel) pathway as depicted in Figure 1, and in addition, their general function is given below the gene names following www.genecards.org. Gene name abbreviations follow the human gene name.

In addition to identification of homologous genes using BLASTn, we confirmed gene localization and copy number using syntenic analysis over the Genomicus genome browser [http://www.genomicus.biologie.ens.fr/genomicus-80.01/cgi-bin/search.pl (Louis *et al.* 2013, 2015)] and Ensembl gene trees. All gene IDs are listed in Table S1, and an illustration of all found genes is given in Figure 2. Tree topology represented on Figure 2 represents the consensus tree for the whole pathway and was built using DupTree (Wehe *et al.* 2008) using all single gene trees as input (see *Phylogenetic reconstruction*).

### Codon alignments

For each gene, the longest available coding sequence was exported and loaded into SeaView 4.5.4 to be manually verified (Gouy *et al.* 2010). Subsequently, in-frame alignments from start to stop codon were performed using a codon empirical model with PRANK v.140110 (Löytynoja and Goldman 2005, 2008), which has been shown to outperform other alignment software for alignments of codon blocks and, in particular, generates less false-positive cases in selection analysis (Fletcher and Yang 2010).

### Phylogenetic reconstruction

The best-fitting nucleotide substitution model for each codon-alignment was estimated using jModelTest 2.1.7 (Guindon and Gascuel 2003; Darriba *et al.* 2012) according to the corrected Akaike information criterion model. The generalized-time-reversible + gamma + I model was found to be the best-fitting model for most trees (40 of 61; see Table S2 for details). Hence, maximum-likelihood phylogenies for all genes were reconstructed using PhyML 3.1 (Guindon *et al.* 2010) under the generalized-time-reversible + gamma + I model, with 1000 bootstrap replicates (all single gene phylogenetic reconstructions are shown in Figure S1).

### Sequence evolution

To determine selection patterns along the teleost phylogeny for genes involved in AR-signaling, we conducted the following analyses. In a first step, we performed site-wise Ka/Ks estimates by using Selecton (http://selecton.tau.ac.il/; Doron-Faigenboim *et al.* 2005; Stern *et al.* 2007) under the M8 model (M8, beta+w > 1) enabled for positive selection (Yang and Bielawski 2000). When positively selected sites were detected under this model, it was tested against the null model [no positive selection, M8a, beta+w = 1, (Swanson *et al.* 2003)]. In case of significance of the likelihood-ratio test, the p-value is indicated. These tests were run on multisequence alignments for each gene using the nonteleost spotted gar as reference (Figure S2).

Next, we ran the branch-site model aBS-REL implemented in HyPhy (HYpothesis testing using PHYlogenies, Version 2.2.4) (Pond *et al.* 2005; Smith *et al.* 2015), which allows for different Ka/Ks ratios among sites and among branches. Resulting trees with selection values were visualized using the http://veg.github.io/hyphy-vision/absrel/ web interface, following the developer’s instructions (Figure S3). We finally ran gene-wide Ka/Ks estimates using KaKs_calculator_2.0 (Version 1.2) under the MA method (Zhang *et al.* 2006) on the following alignments: all cichlids [East African Lake (EAL) cichlids + Nile tilapia] compared with medaka and EAL cichlids compared with Nile tilapia. Ka/Ks comparisons were performed over the entire sequence length.

### Gene expression in cichlid fishes

To examine gene expression profiles, we retrieved RNA-sequencing information for the gene members of the AR-signaling pathway for four species from Lake Tanganyika (*A. burtoni*, *Eretmodus cyanostictus*, *Julidochromis ornatus*, and *Ophthalmotilapia ventralis*) from Böhne *et al.* (2014). The sequenced tissues of this study are brain and gonad from adult males and females. Note that genomic information is currently only available for *A. burtoni* (Brawand *et al.* 2014).

Cleaned reads for each individual sample for each species as described in Böhne *et al.* (2014) were mapped against our *A. burtoni* dataset of gene coding sequences. Mapping was done using Novoalign (Novocraft) with the following settings: –r All, -t 60, -l 30, and -s 5. Alignments were reported in SAM format and sorted, indexed, and transformed into count tables (number of mapped reads per transcript per sample) with the use of SAMtools (Li *et al.* 2009). In total, the data set comprised read counts for four male brain, four female brain, four ovary, and four testis samples per species except for *A. burtoni*, where three biological replicates per tissue type were available. Differential expression analyses on raw read counts were performed with the Bioconductor edgeR package (Version 3.1) (Robinson *et al.* 2010) by use of the “classical” model as described in Böhne *et al.* (2014). Count numbers were subsequently transformed into fragments per kilobase of transcript per million mapped reads (FPKM) to estimate relative transcript abundance within edgeR. FPKM values of paralogs were compared with a two-sided Welch *t*-test. Analyses using PRANK, PhyML, Novoalign, and SAMtools were run on sciCORE (center for scientific computing, University of Basel, http://scicore.unibas.ch/)

### Data availability

Sequence data are available at GenBank and Ensembl, the corresponding accession numbers are list in Table S1. Gene expression data were taken from Böhne *et al.* 2014. Scripts are available upon request.

## Results

### Tracing the AR pathway in fish genomes

We screened 14 ray-finned fish genomes (13 teleosts and the spotted gar) for 64 genes (Table S1) belonging to the AR-signaling pathway (Figure 1). The spotted gar is part of the family Lepisosteiformes, which diverged from the teleost lineage before the TSGD. Compared with teleost fishes, for which many genome rearrangements have occurred since the TSGD, the gar genome is closer in organization to the human genome (Amores *et al.* 2011) and was thus considered as the appropriate out-group for the here presented phylogenetic and sequence evolution analyses.

For two genes (*nr1i3* and *scgb2a1*), we could not detect any ray-finned fish ortholog in the screened genomes, with *nr1i3* being restricted to Sarcopterygii and *scgb2a1* being a mammalian-specific gene. Out of the remaining 62 genes, 20 were retained as duplicates in most lineages after the TSGD (Figure 2). One gene, *kdm3a* is only present in the spotted gar, which indicates a loss of this gene at the basis of teleosts.

Some genes subsequently experienced lineage specific losses (such as *cfl1l* in pufferfishes, *rnf14* in cichlids, *rhoaab* in Atherinomorphae, and *rhoaaa* in Eupercaria) and probably more recent losses on the species level (*e.g.*, *pik3r2b* in the medaka, *dnaja1* in fugu). We also found lineage- (*e.g.*, *rac1a* in cichlids and medaka independently; *rac1b* in poeciliids) and species-specific duplication events (*e.g.*, *egfrb* in tetraodon).

In summary, we detected 17 losses of one or both duplicates for genes otherwise retained in TSGD duplicates and further nine gene losses and four pseudogenes of otherwise single copy genes in teleosts. In total, we found additional lineage- or species-specific duplications for 19 genes.

### Sequence evolution of the AR pathway in teleosts with a focus on cichlids

**Figure 3:**
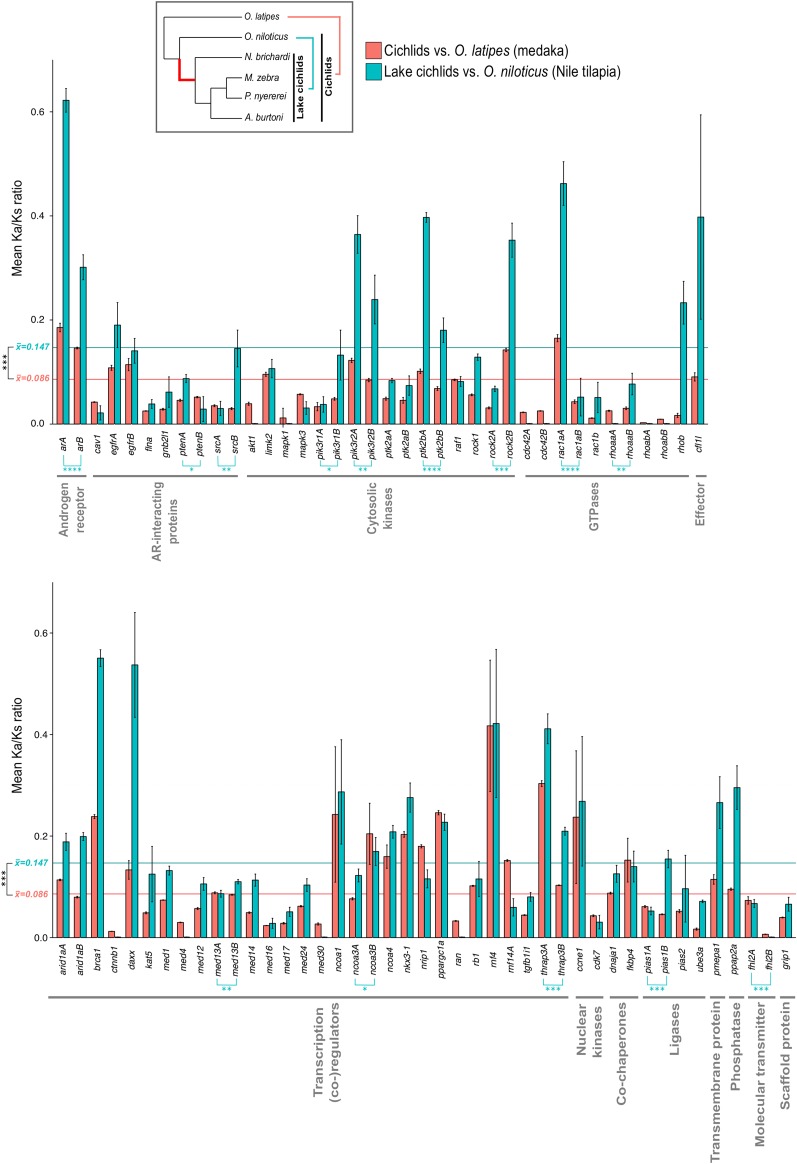
Comparisons of Ka/Ks ratios (nonsynonymous substitutions per nonsynonymous site to the number of synonymous substitutions per synonymous site) between cichlids and medaka (red) and between East African Lake cichlids and the more basal Nile tilapia (blue) for all genes of the androgen receptor−signaling pathway (upper panel: nongenomic; lower panel: genomic). Gene functions are given under gene names according to Figure 2. Error bars represent SDs. Horizontal colored bars represent mean Ka/Ks values over all genes. Significance levels of Ka/Ks comparisons tested by two-sided Welch *t*-test with: (****) p-value < 0.0001, (***) p-value < 0.001, (**) p-value < 0.01, (*) p-value < 0.05 (see Table S3 for details).

Branch-site models in HyPhy overall suggested the action of purifying selection on most of the branches over the majority of all sites (Ka/Ks <1). We detected signs of significant positive selection (Ka/Ks >1) acting on four branches in four genes, namely on the branch at the basis of the teleosts after the split from spotted gar for *ncoa3*, *pik3r2*, and *rock2* and on the branch of *pik3r1b* of *A. burtoni* (Figure S3). Site-wise models implemented in Selecton indicated strong purifying selection acting on all investigated genes, with only two genes (*fkbp4* and *rhob*) having sites under significant positive selection (Figure S2). To test for selection and changes in selection regimes on a shorter evolutionary scale, we decided to focus on the only fish lineage, for which five genomes are currently available, the cichlids (Cichlidae). These include four species from the East African Great Lakes (*A. burtoni* and *N. brichardi* from Lake Tanganyika, *M. zebra* from Lake Malawi, and *P. nyererei* from Lake Victoria) and, with the Nile tilapia (*O. niloticus*), a member of a lineage basal to the lake cichlids. We tested selection acting on all cichlids compared with the next closest relative, the medaka (*O. latipes*) and also on East African Lake (EAL) cichlids compared with Nile tilapia by using gene-wide Ka/Ks estimates. Overall, Ka/Ks ratios were significantly greater on average after the split from the Nile tilapia (0.086 *vs.* 0.147, Figure 3). Interestingly, the greatest value in the comparison EAL cichlids *vs.* Nile tilapia is reached by *ara*, the key gene of the here investigated pathway.

Furthermore, after the split from Nile tilapia, the difference in selection regimes on (TSGD) duplicates increases significantly, especially so for *ar*, *ptk2b*, and *rac1a*. This pattern is particularly striking for the comparison between *rac1aa* and *rac1ab*. Ka/Ks for *rac1aa* is 4-fold and 9.2-fold greater than for *rac1ab* in the cichlids-medaka comparisons and EAL cichlids-Nile tilapia, respectively.

### Gene expression in cichlid fishes

**Figure 4:**
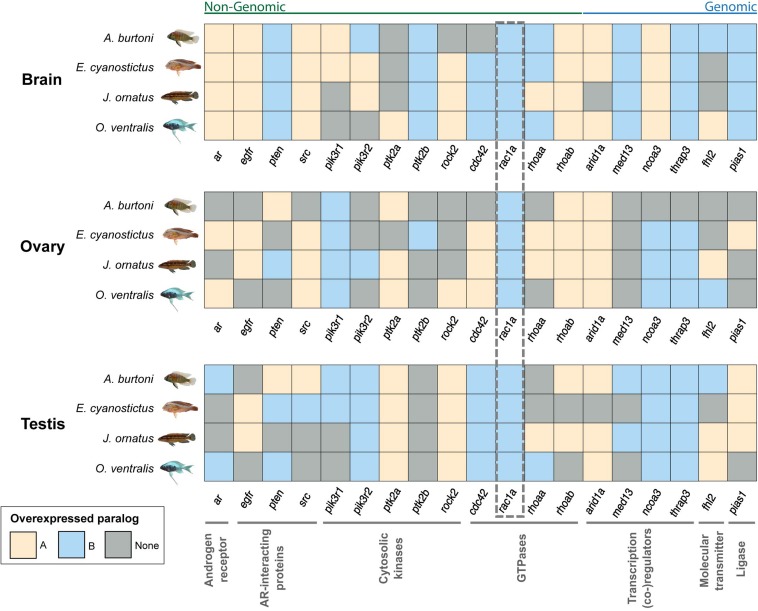
Expression patterns for teleost-specific whole-genome duplication duplicates of the androgen receptor−signaling pathway in four Lake Tanganyika cichlids, *A. burtoni*, *E. cyanostictus*, *J. ornatus*, and *O. ventralis*. Gene expression levels in fragments per kilobase of transcript per million mapped reads were compared for the two copies of each gene within three different tissues and considered significantly differentially expressed with a p-value < 0.05 (two-sided Welch *t*-test; see Table S4 for details). Grouping to functional classes indicated below the expression patterns is according to Figure 2. The dotted box highlights the GTPase *rac1a*, which is the only gene for which one paralog is always overexpressed in all tissues and all species studied.

Gene duplicates seem to experience different selection regimes even at derived evolutionary stages as in the here investigated cichlids. Previously, we showed that cichlids have a high turnover in gene expression patterns (Böhne *et al.* 2014). Hence, we next focused on expression differences of TSGD paralogs and the more recent lineage-specific duplication of *rac1a* in three tissues, brain, ovary, and testis. For this analysis, we used the RNA-sequencing data set of Böhne *et al.* (2014), which comprises four Lake Tanganyika cichlids (Figure 4), including again *A. burtoni*, for which the entire genome sequence is available (Brawand *et al.* 2014) and included in our sequence analyses.

Especially in the brain, we detected largely conserved expression patterns with always the same paralog being overexpressed in all four species (11 of 19 genes), which is indicative of ancestral subfunctionalization events of the two gene copies within this tissue. This pattern is less pronounced in testis (seven genes) and even less in ovary (three genes).

Within a tissue, we rarely observed a change in paralog-overexpression (*i.e.*, one duplicate is overexpressed in one species and the other in another species in the same tissue). This pattern was found for *fhl2* as the only gene in all three tissues, *rhoaa* in brain and testis, *pten* in ovary and testis, *src* in testis, and *pik3r2* in the brain.

Focusing on between tissue comparisons, we found that in four cases (*cdc42*, *ncoa3*, *pik3r1*, and *ar*), the overexpression of the A or B duplicate changed depending on the tissue type, again indicative of different functionalities of the gene copies. This pattern is most pronounced for *ncoa3*, for which the A-copy is overexpressed in the brain whereas in general the B paralog is overexpressed in ovary and testis. In summary, this points to recent subfunctionalization events on the species and lineage level with an influence of the tissue inspected.

The strongest indication for a general subfunctionalization on a lineage level in gene expression comes from *rac1a*. This GTPase is the only gene for which one of the lineage-specific paralogs (the B-copy, *rac1ab*) is always overexpressed in all tissues and all species studied (dotted box in Figure 4).

**Figure 5:**
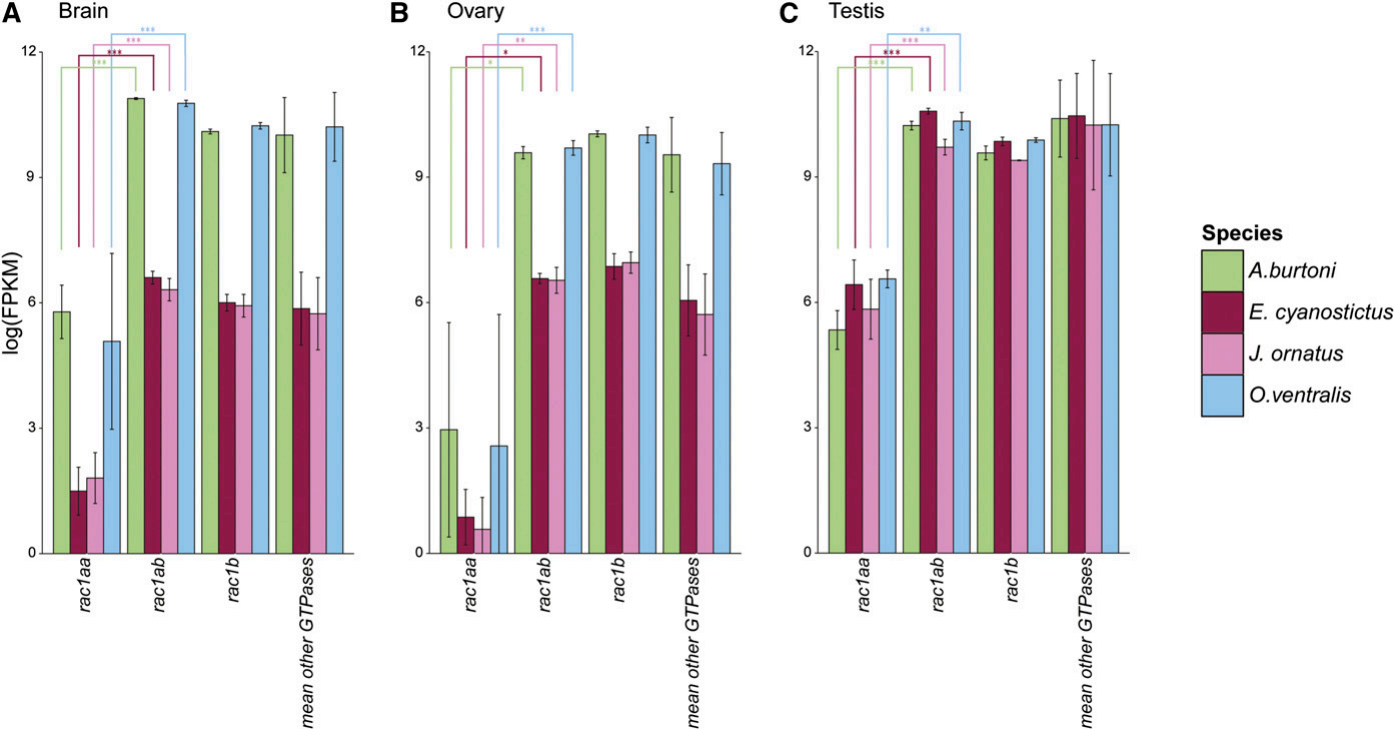
Fragments per kilobase of transcript per million mapped reads (FPKM) values for *rac1aa*, *rac1ab*, *rac1b*, and mean value for all other GTPases (see text for list) in (A) brain, (B) ovary, and (C) testis for four cichlid species. Color scheme for species is represented on the right panel. Error bars represent SD. Statistical significance: two-sided Welch *t*-test with (***) p-value < 0.001, (**) p-value < 0.01, (*) p-value < 0.05.

To assess whether this pattern was due to an increased expression of *rac1ab* or to a decreased expression of *rac1aa*, we compared expression levels (in FPKM) of both paralogs with the TSGD ohnolog *rac1b* and all other GTPase genes in the pathway: *cdc42a*, *cdc42b*, *rhoaaa*, *rhoaab*, *rhoaba*, *rhoabb*, and *rhob* (Figure 5). In all species, a 40-fold to 120-fold reduction in gene expression was observed for *rac1aa* compared with other GTPases, whereas *rac1ab* showed no difference with these ubiquitous proteins and strongly resembles *rac1b* in expression. This finding suggests that the difference in expression between the two more recent duplicates is due to a down-regulation of the general expression level of *rac1aa* compared with *rac1ab* in EAL cichlids.

## Discussion

Genome duplications, gene gains, and losses shape gene families and networks. Recently, the evolution of the androgen system in vertebrates has been described with special attention to the three rounds of whole-genome duplication that occurred in this lineage (Baker *et al.* 2015). With our study, we complete the picture focusing for the first time on the genomic composition of the AR-signaling pathway in ray-finned fishes with special attention to East African cichlids. We identified fish orthologs for 62 of the 64 genes with one gene being present only in the spotted gar, indicating a loss at the basis of the teleost lineage. Of the remaining 62 genes, 20 were retained in duplicate after the TSGD, which is in the range of the overall loss rate estimates of 76–85% of duplicates after the TSGD in teleosts (Jaillon *et al.* 2004; Woods *et al.* 2005; Brunet *et al.* 2006). Interestingly, more duplicates were retained in the nongenomic signaling pathway (13 of 24) than the genomic one (7 of 37). We confirm a gene loss of *arb* (Douard *et al.* 2008) and *rhob* in zebrafish (Salas-Vidal *et al.*, 2005) but show that the first one is, contrary to previous suggestions (Douard *et al.* 2008), not a loss in the entire Otophysi lineage, because we found *arb* to be present in the cavefish genome. Using only genomic information available in Ensembl, we identified in zebrafish only one copy for *cdc42* and *egfr* and no *rac1b* gene, contrarily to previous observations (Salas-Vidal *et al.* 2005; Laisney *et al.* 2010).

Evolutionary rates, as previously shown for at least two members of the AR pathway, *ar* (Douard *et al.* 2008) and *fhl2* (Santos *et al.* 2014), were found to be different between A and B paralogs for most ohnologs. Such differences could be suggestive of possible neofunctionalization events in one gene copy with retention of the ancestral function in the more conserved, slower evolving copy.

Neofunctionalization of gene duplicates can be driven by positive selection (Beisswanger and Stephan 2008). Overall, we found no recurrent statistical support for positive selection on specific sites or branches acting on the AR pathway in teleosts, indicating conservation or constraint acting on the pathway. However, the investigated species cover a phylogenetic spectrum of ∼350 million years. We thus decided to have a closer look into probable changes in selection regimes happening on a younger evolutionary scale. Within publicly available teleost genomes, only one particular group offers this possibility, the cichlids (Cichlidae), with five accessible genomes. In agreement with genome- and transcriptome-wide estimates (Baldo *et al.* 2011; Brawand *et al.* 2014), we could show that EAL cichlids have accelerated sequence evolution in the AR signaling pathway after their split from a common outgroup, the basal cichlid Nile tilapia around 25−50 million years ago (Brawand *et al.* 2014 and references therein), compared to values obtained in comparison all cichlids to the next closest relative with a sequenced genome, the medaka (split: ∼140 million years ago; Azuma *et al.* 2008). This pattern could be indicative of either relaxed constraint or positive selection acting on the derived evolutionary stage of the cichlid lineage. Interestingly, this pattern is most pronounced for the A-copy of the AR itself. This analysis also showed that TSGD duplicates differ in their evolutionary rate at this derived evolutionary stage, opening up the possibility for late neofunctionalization events.

Changes in gene expression levels and locations also can be indicative of a neo- or subfunctionalization. It also has been shown previously in cichlids that paralogs can have species- and tissue-specific expression patterns. These data include TSGD and other whole-genome duplicates of neuroendocrine gene families such as the pro-opiomelanocortin (*pomc*) family (Harris *et al.* 2014), as well as the ARs (Harbott *et al.* 2007) and genes implicated in sexual development (Böhne *et al.* 2014). For duplicated genes of the AR-signaling pathway, we could show here that paralogs can have tissue- and species-specific overexpression patterns. General conservation of the same paralog overexpression is seen in the brain of cichlids, whereas the gonads and especially the ovary show more species-specific patterns.

A particularly strong indication for a late neofunctionalization event comes from the expression and selection pattern of the *rac1a* A and B copies, which are specific to the cichlid lineage (note that *rac1a* also has been duplicated in the medaka, but according to our phylogenetic reconstruction, this is an independent event). Ka/Ks in *rac1ab* is similar to *rac1b*, the corresponding TSGD paralog of *rac1aa* and *rac1ab*; however, it is significantly greater in *rac1aa*, and it is *rac1ab* that is overexpressed in all tissues in all species compared with *rac1aa*, although at the same expression level than other GTPases and its ohnolog *rac1b*, probably reflecting the ancient expression level. This finding suggests reduced gene expression for the faster-evolving *rac1aa*. Reduced gene expression can promote gene neofunctionalization, as shown for a sodium channel gene duplicate in electric fishes (Thompson *et al.* 2014). Indeed, a reduced gene expression of one paralog can enable faster gene evolution of this gene without altering the individual’s fitness provided that the other copy retains the “required” ancestral expression and function.

As other neuroendocrine pathways, the AR signaling pathway has been shaped by gene and genome duplications. We found that this pathway is generally under purifying selection in teleosts but shows accelerated evolutionary rates in cichlid fishes with indications for neofunctionalization on the lineage and species level.

## 

## Supplementary Material

Supporting Information
