# Supplementary material for: Evolutionary Fate of the Androgen Receptor−Signaling Pathway in Ray-Finned Fishes with a Special Focus on Cichlids
Source: G3 (Bethesda). 2015 Sep 1;5(11):2275–83. doi: 10.1534/g3.115.020685 (PMC4632047; doi:10.1534/g3.115.020685)
Supplement: Supporting Information [file supp_5_11_2275__index.html]

Evolutionary Fate of the Androgen Receptor−Signaling Pathway in Ray-Finned Fishes with a Special Focus on Cichlids — Supporting Information 

# Evolutionary Fate of the Androgen Receptor−Signaling Pathway in Ray-Finned Fishes with a Special Focus on Cichlids

## Supporting Information for Lorin, Salzburger, and B?hne, 2015

**Files in this Data Supplement:**

- Supporting Information - Figures S1-S3 and Tables S1-S4 (PDF, 9 MB)
- Figure S1 - Maximum-likelihood single gene phylogenetic reconstructions for genes of the AR signaling pathway in ray-finned fishes using PhyML 3.1 (Guindon *et al.* 2010) under the GTR + gamma + I model, with 1,000 bootstrap replicates. (PDF, 1 MB)
- Figure S2 - Site-wise Ka/Ks estimates using Selecton under the M8 model allowing for positive selection. (PDF, 3 MB)
- Figure S3 - Resulting trees of branch-site model aBS-REL implemented in HyPhy allowing for different Ka/Ks ratios among sites and among branches for all retrieved genes of the AR signaling pathway. (PDF, 4 MB)
- Table S1 - Exhaustive gene list with accession numbers for the AR signaling pathway in ray-finned fishes. (PDF, 165 KB)
- Table S2 - Results for jmodeltest on each coding sequence alignment indicating the best fitting nucleotide substitution model per gene. (PDF, 110 KB)
- Table S3 - P-values of two-sided Welch's t-test of Ka/Ks comparisons Lake cichlids *vs.* Nile tilapia for TSGD gene copy A *vs.* gene copy B as shown in Figure 3. (PDF, 145 KB)
- Table S4 - P-values of two-sided Welch's t-test for comparisons of FPKM values of TSGD duplicate genes in four cichlid species in brain, ovary and testis as shown in Figure 4. (PDF, 157 KB)
