# Supplementary material for: Evolutionary Fate of the Androgen Receptor−Signaling Pathway in Ray-Finned Fishes with a Special Focus on Cichlids
Source: G3 (Bethesda). 2015 Sep 1;5(11):2275–83. doi: 10.1534/g3.115.020685 (PMC4632047; doi:10.1534/g3.115.020685)
Supplement: Supporting Information [file supp_g3.115.020685_020685SI.pdf]

G3 · Genes | Genomes | Genetics

Evolutionary fate of the androgen receptor-signaling pathway in ray-finned fishes with a special focus on cichlids

Thibault Lorin[*§], Walter Salzburger[§], Astrid Böhne[§1]

* ENS (Ecole Normale Supérieure de Lyon), Lyon Cedex 07, France;

[§]Zoological Institute, Department of Environmental Sciences, University of Basel, 4051 Basel, Switzerland

[1]Corresponding author: Astrid Böhne, Zoological Institute, Department of Environmental Sciences, University of Basel, Vesalgasse 1, 4051 Basel, Switzerland, +41 61 267 03 01, astrid.boehne@unibas.ch

DOI: 10.1534/g3.115.020685

Figure S1

Maximum-likelihood single gene phylogenetic reconstructions for genes of the AR signaling pathway in ray-finned fishes using PhyML 3.1 (Guindon *et al.* 2010) under the GTR + gamma + I model, with 1,000 bootstrap replicates.

Figure S1

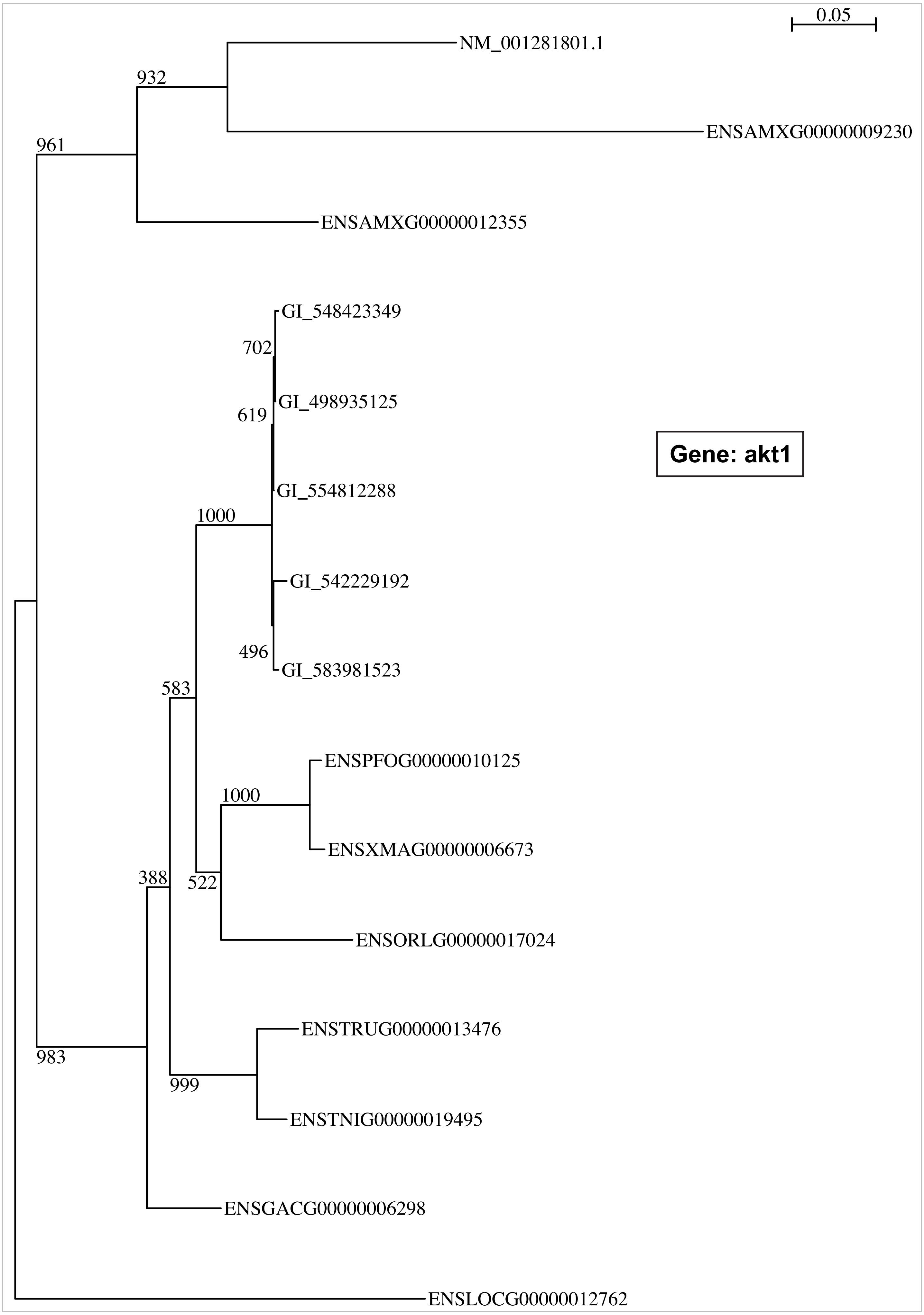
0.05
NM_001281801.1
ENSAMXG00000009230
ENSAMXG00000012355
GI_548423349
GI_498935125
GI_554812288
GI_542229192
GI_583981523
ENSPFOG00000010125
ENSXMAG00000006673
ENSORLG00000017024
ENSTRUG00000013476
ENSTNIG00000019495
ENSGACG00000006298
ENSLOCG00000012762
932
961
702
619
1000
496
583
1000
388
522
983
999
Gene: akt1

Figure S1

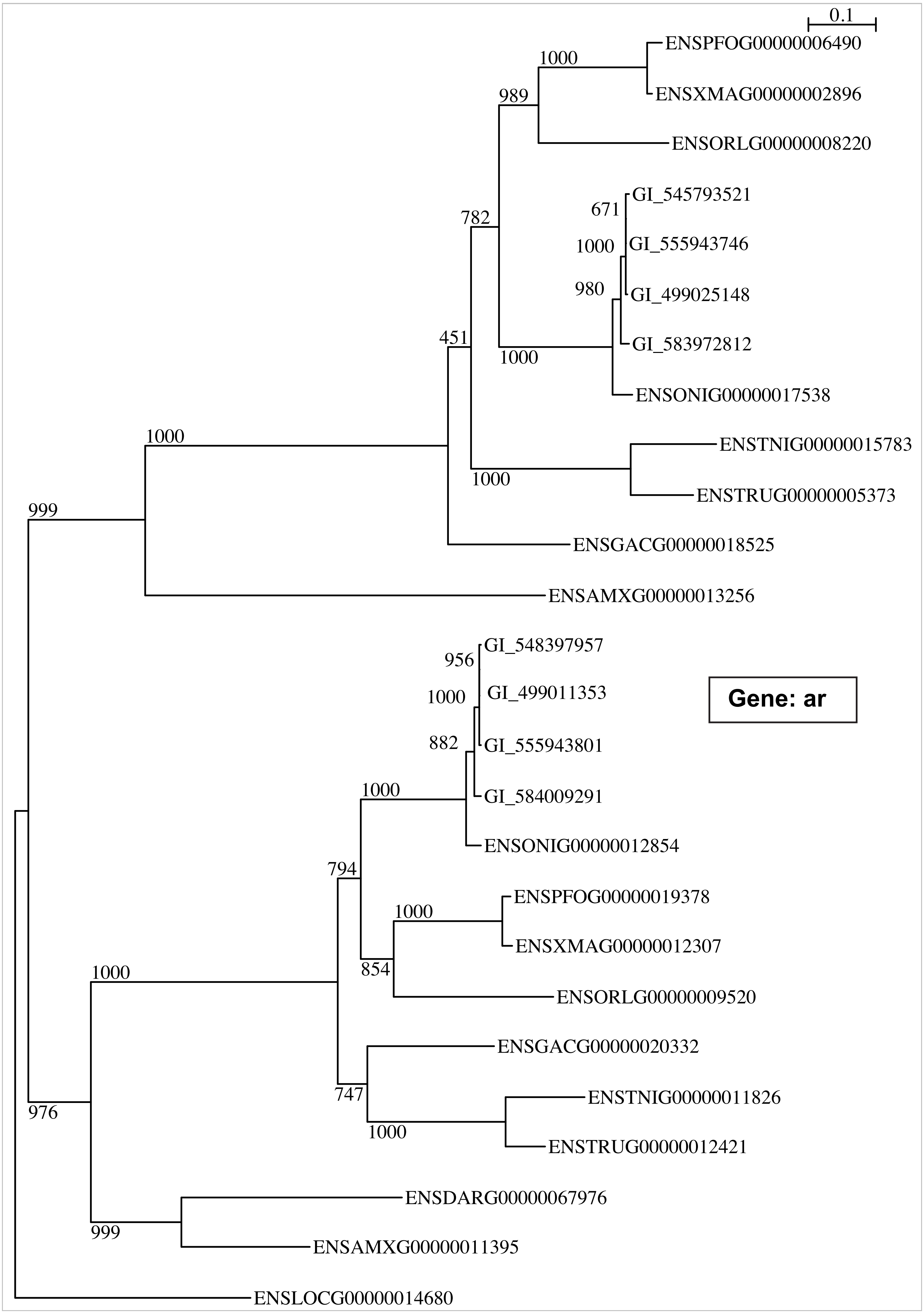
0.1
ENSPFOG00000006490
ENSXMAG00000002896
ENSORLG00000008220
GI_545793521
GI_555943746
GI_499025148
GI_583972812
ENSONIG00000017538
ENSTNIG00000015783
ENSTRUG00000005373
ENSGACG00000018525
ENSAMXG00000013256
GI_548397957
GI_499011353
GI_555943801
GI_584009291
ENSONIG00000012854
ENSPFOG00000019378
ENSXMAG00000012307
ENSORLG00000009520
ENSGACG00000020332
ENSTNIG00000011826
ENSTRUG00000012421
ENSDARG00000067976
ENSAMXG00000011395
ENSLOCG00000014680
1000
989
782
671
1000
980
451
1000
1000
1000
999
956
1000
882
1000
794
1000
854
1000
747
1000
976
999
Gene: ar

Figure S1

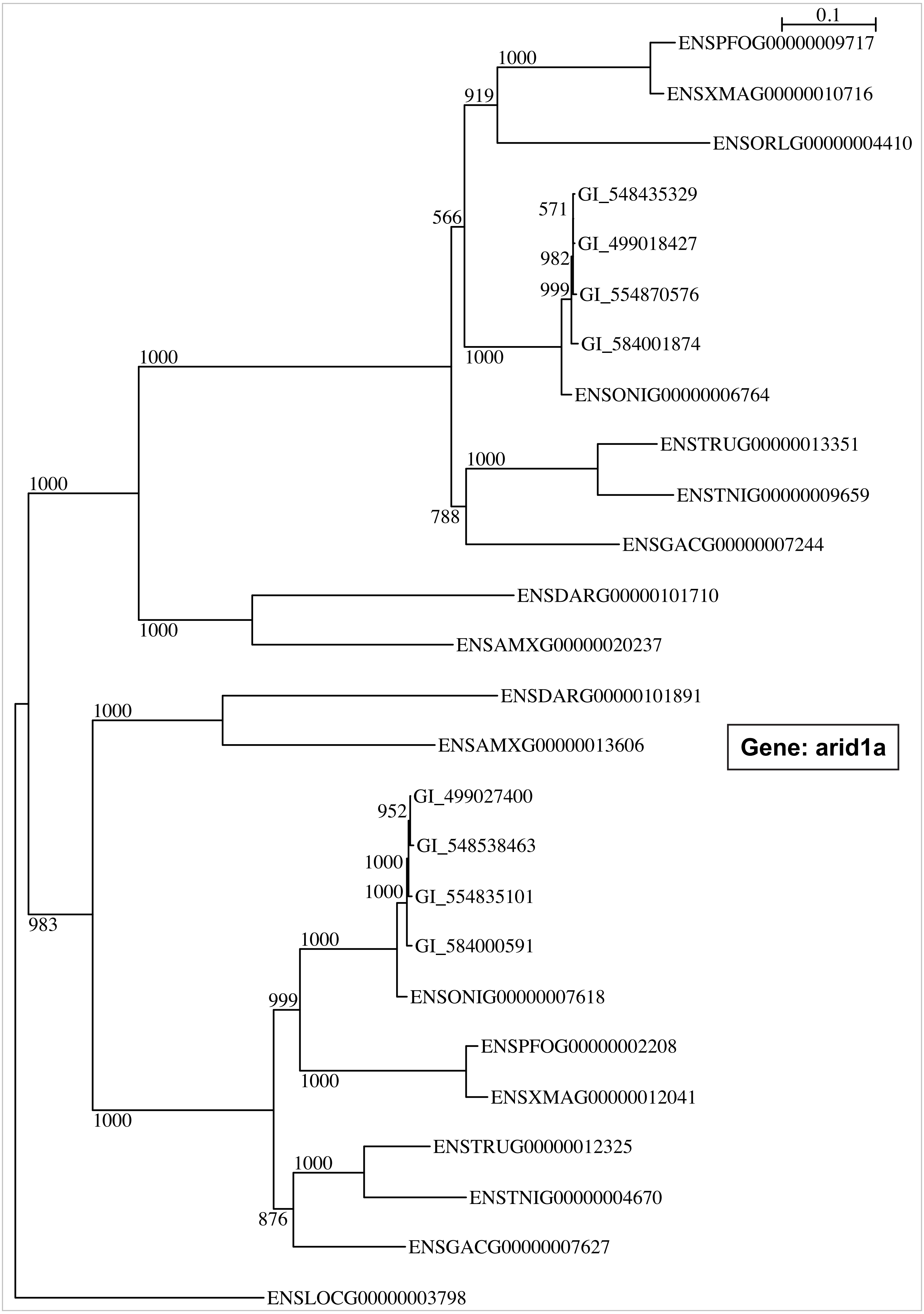

 T. Lorin, W. Salzburger & A. Böhne

Figure S1

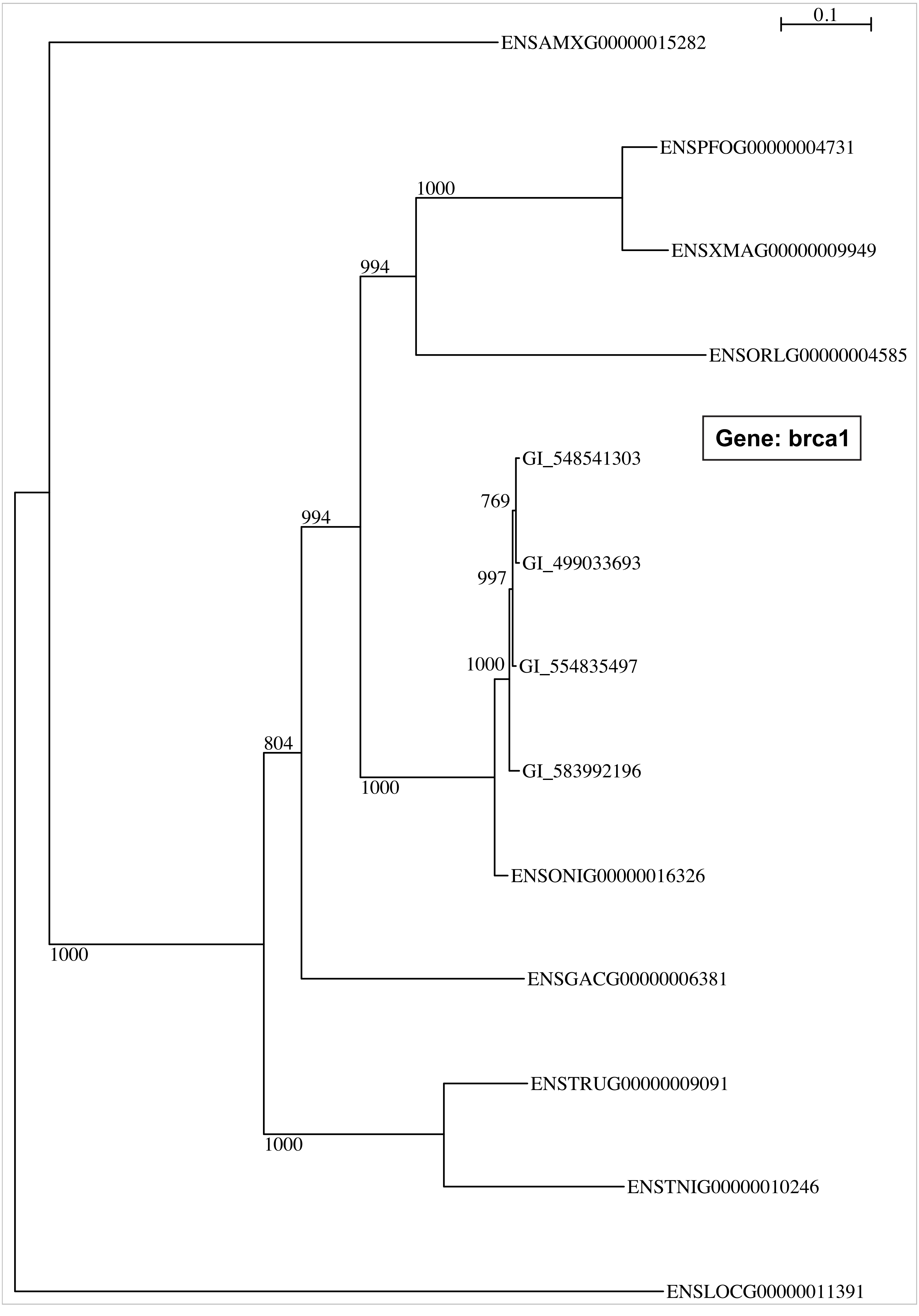

0.1
ENSAMXG00000015282
ENSPFOG00000004731
1000
ENSXMAG00000009949
994
ENSORLG00000004585
Gene: brca1
GI_548541303
769
994
GI_499033693
997
1000
GI_554835497
804
GI_583992196
1000
ENSONIG00000016326
1000
ENSGACG00000006381
ENSTRUG00000009091
1000
ENSTNIG00000010246
ENSLOCG00000011391

Figure S1

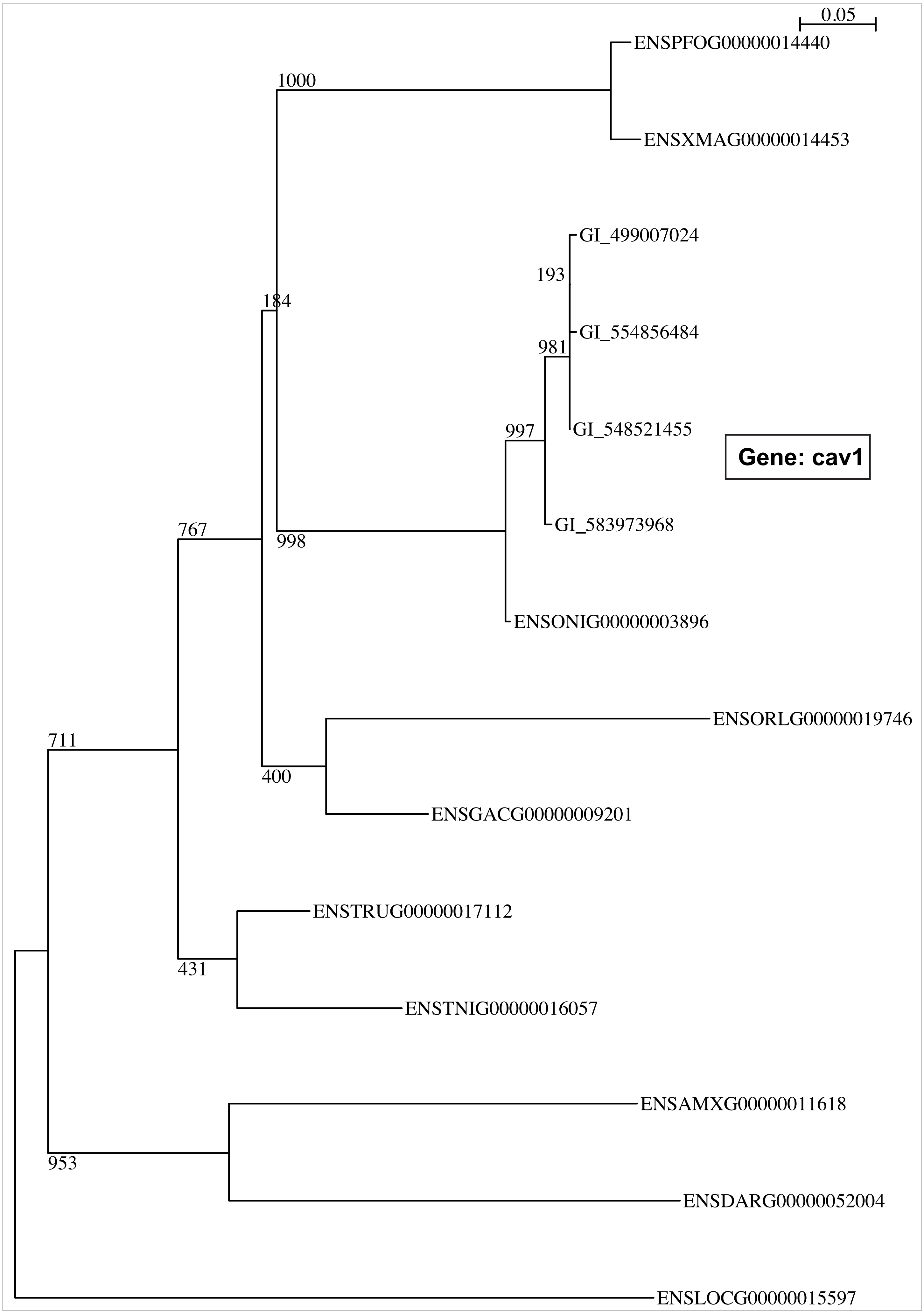

0.05
ENSPFOG00000014440
1000
ENSXMAG00000014453
GI_499007024
193
184
GI_554856484
981
GI_548521455
997
Gene: cav1
GI_583973968
767
998
ENSONIG00000003896
ENSORLG00000019746
711
400
ENSGACG00000009201
ENSTRUG00000017112
431
ENSTNIG00000016057
ENSAMXG00000011618
953
ENSDARG00000052004
ENSLOCG00000015597

Figure S1

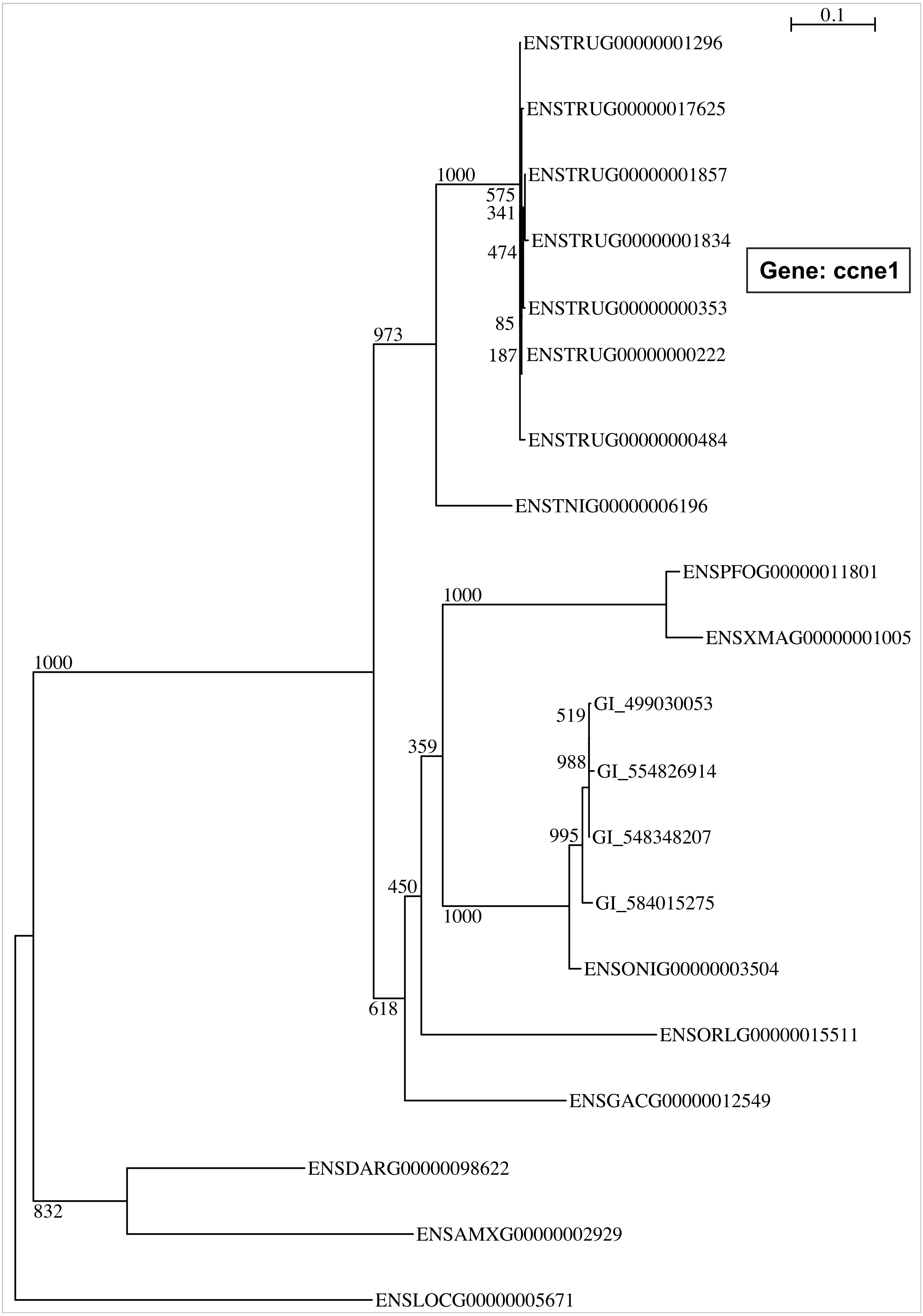
0.1
ENSTRUG00000001296
ENSTRUG00000017625
1000
ENSTRUG00000001857
575
341
ENSTRUG00000001834
474
Gene: ccne1
ENSTRUG00000000353
85
973
187
ENSTRUG00000000222
ENSTRUG00000000484
ENSTNIG00000006196
ENSPFOG00000011801
1000
ENSXMAG00000001005
1000
GI_499030053
519
359
988
GI_554826914
995
GI_548348207
450
GI_584015275
1000
ENSONIG00000003504
618
ENSORLG00000015511
ENSGACG00000012549
ENSDARG00000098622
832
ENSAMXG00000002929
ENSLOCG00000005671

Figure S1

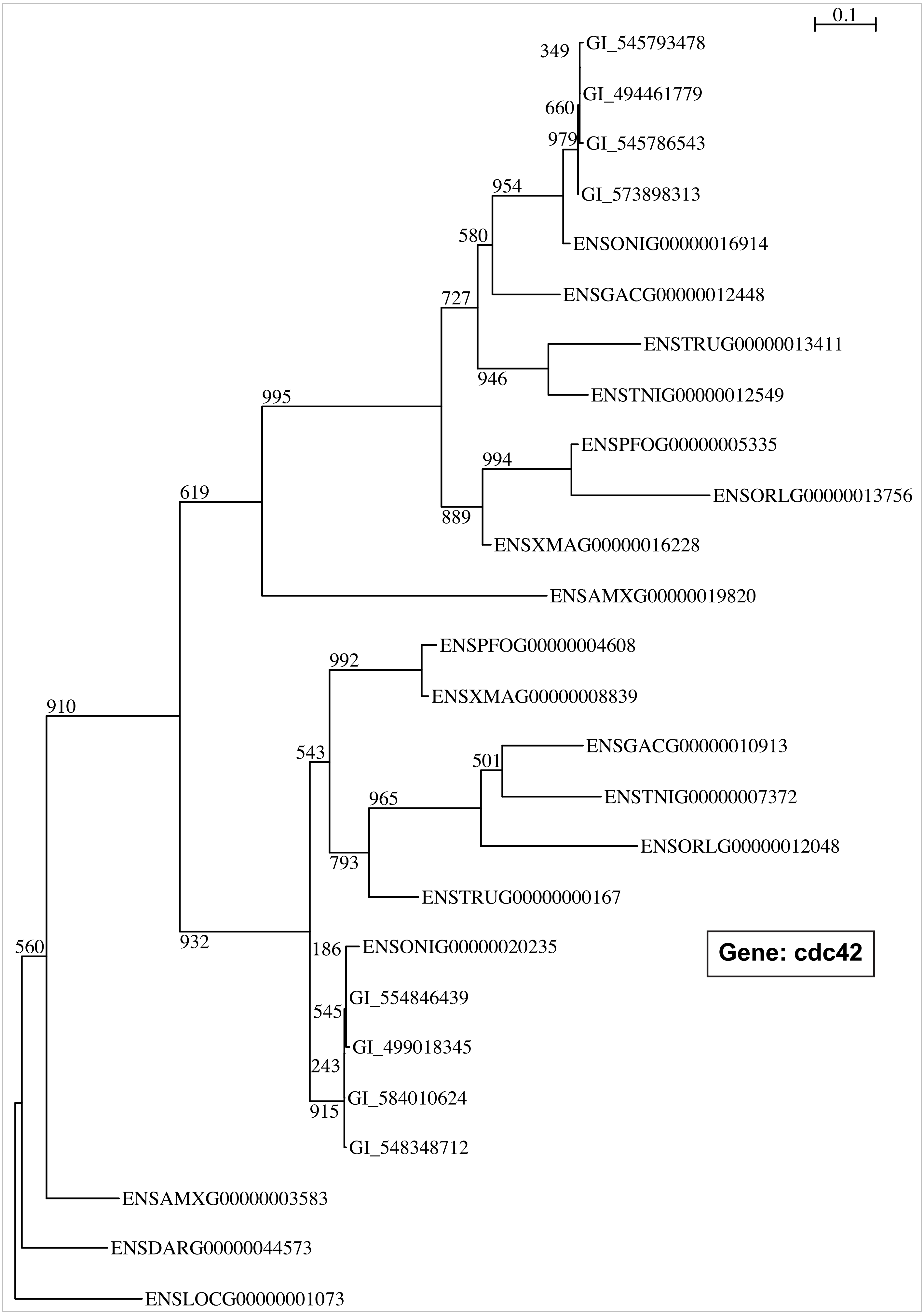
0.1
GI_545793478
GI_494461779
GI_545786543
GI_573898313
ENSONIG00000016914
ENSGACG00000012448
ENSTRUG00000013411
ENSTNIG00000012549
ENSPFOG00000005335
ENSORLG00000013756
ENSXMAG00000016228
ENSAMXG00000019820
ENSPFOG00000004608
ENSXMAG00000008839
ENSGACG00000010913
ENSTNIG00000007372
ENSORLG00000012048
ENSTRUG00000000167
ENSONIG00000020235
GI_554846439
GI_499018345
GI_584010624
GI_548348712
ENSAMXG00000003583
ENSDARG00000044573
ENSLOCG00000001073
349
660
979
954
580
727
946
995
994
889
619
992
910
543
501
965
793
932
186
560
545
243
915
Gene: cdc42

Figure S1

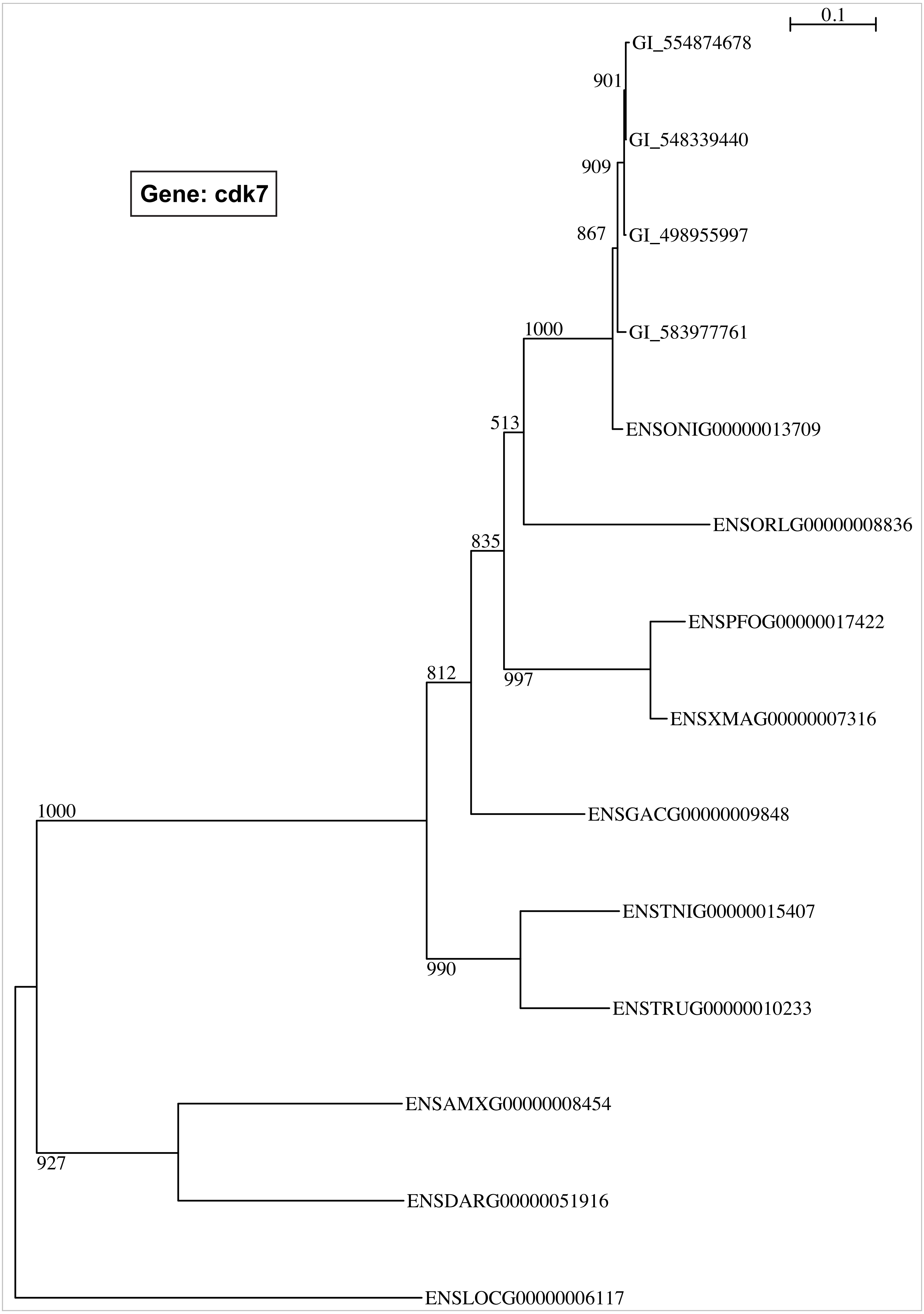
0.1
Gene: cdk7
GI_554874678
901
GI_548339440
909
GI_498955997
867
GI_583977761
1000
ENSONIG00000013709
513
ENSORLG00000008836
835
ENSPFOG00000017422
812
997
ENSXMAG00000007316
1000
ENSGACG00000009848
ENSTNIG00000015407
990
ENSTRUG00000010233
ENSAMXG00000008454
927
ENSDARG00000051916
ENSLOCG00000006117

Figure S1

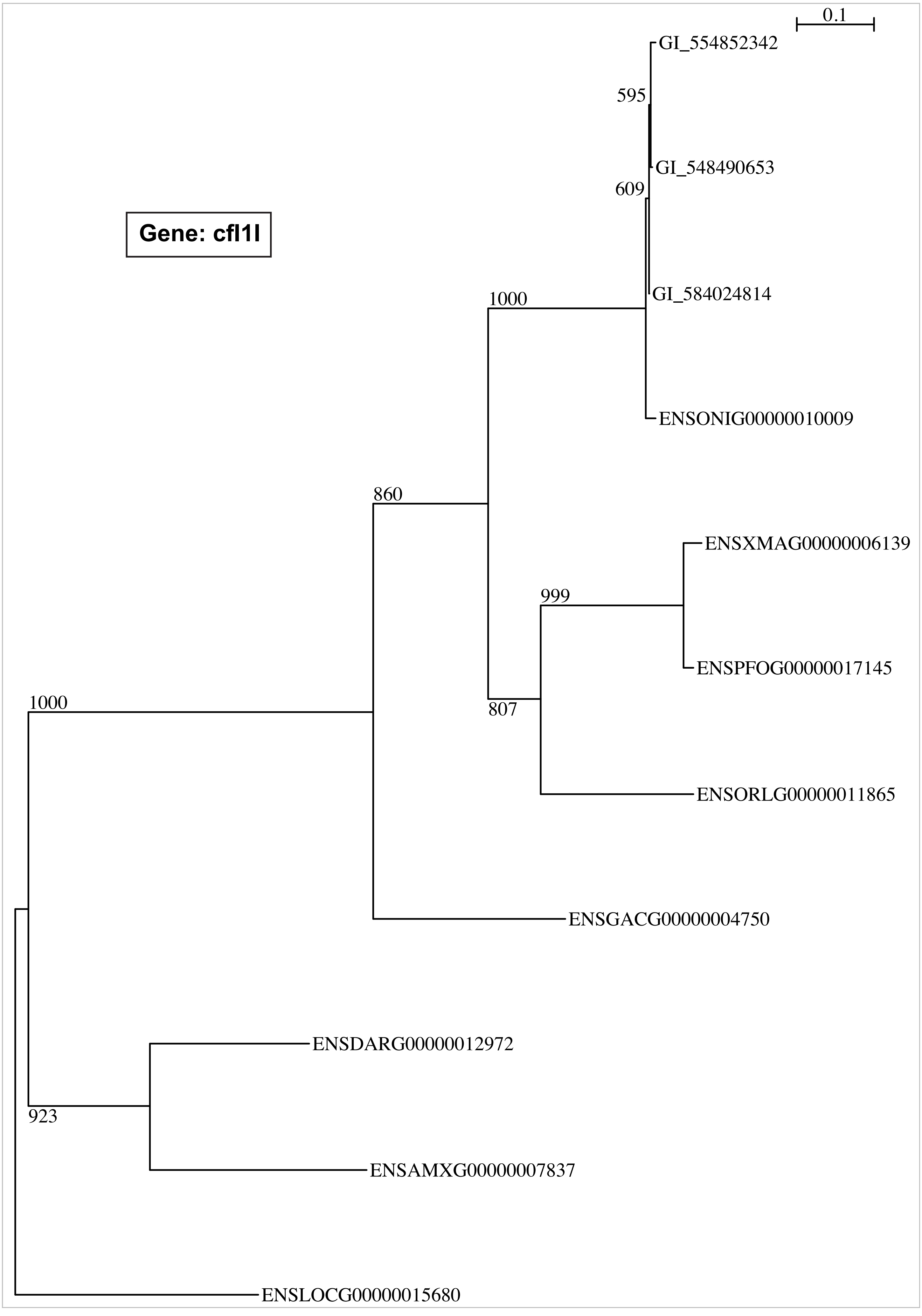

0.1
Gene: cfl1l
GI_554852342
595
GI_548490653
609
GI_584024814
1000
ENSONIG00000010009
860
ENSXMAG00000006139
999
ENSPFOG00000017145
807
ENSORLG00000011865
1000
ENSGACG00000004750
ENSDARG00000012972
923
ENSAMXG00000007837
ENSLOCG00000015680

Figure S1

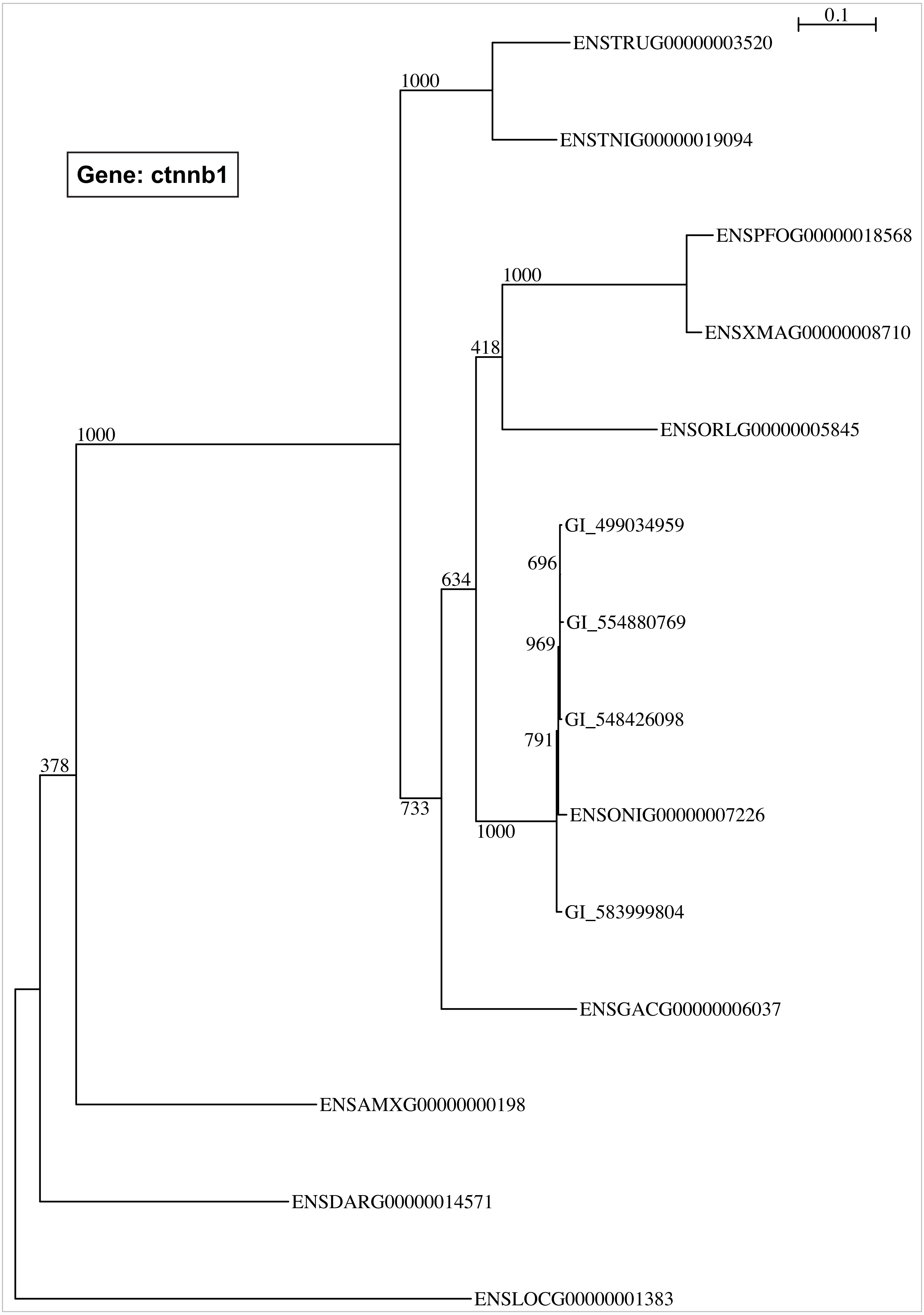

Gene: ctnnb1
0.1
ENSTRUG00000003520
ENSTNIG00000019094
ENSPFOG00000018568
ENSXMAG00000008710
ENSORLG00000005845
GI_499034959
GI_554880769
GI_548426098
ENSONIG00000007226
GI_583999804
ENSGACG00000006037
ENSAMXG00000000198
ENSDARG00000014571
ENSLOCG00000001383
1000
1000
1000
418
634
696
969
791
733
1000
378

Figure S1

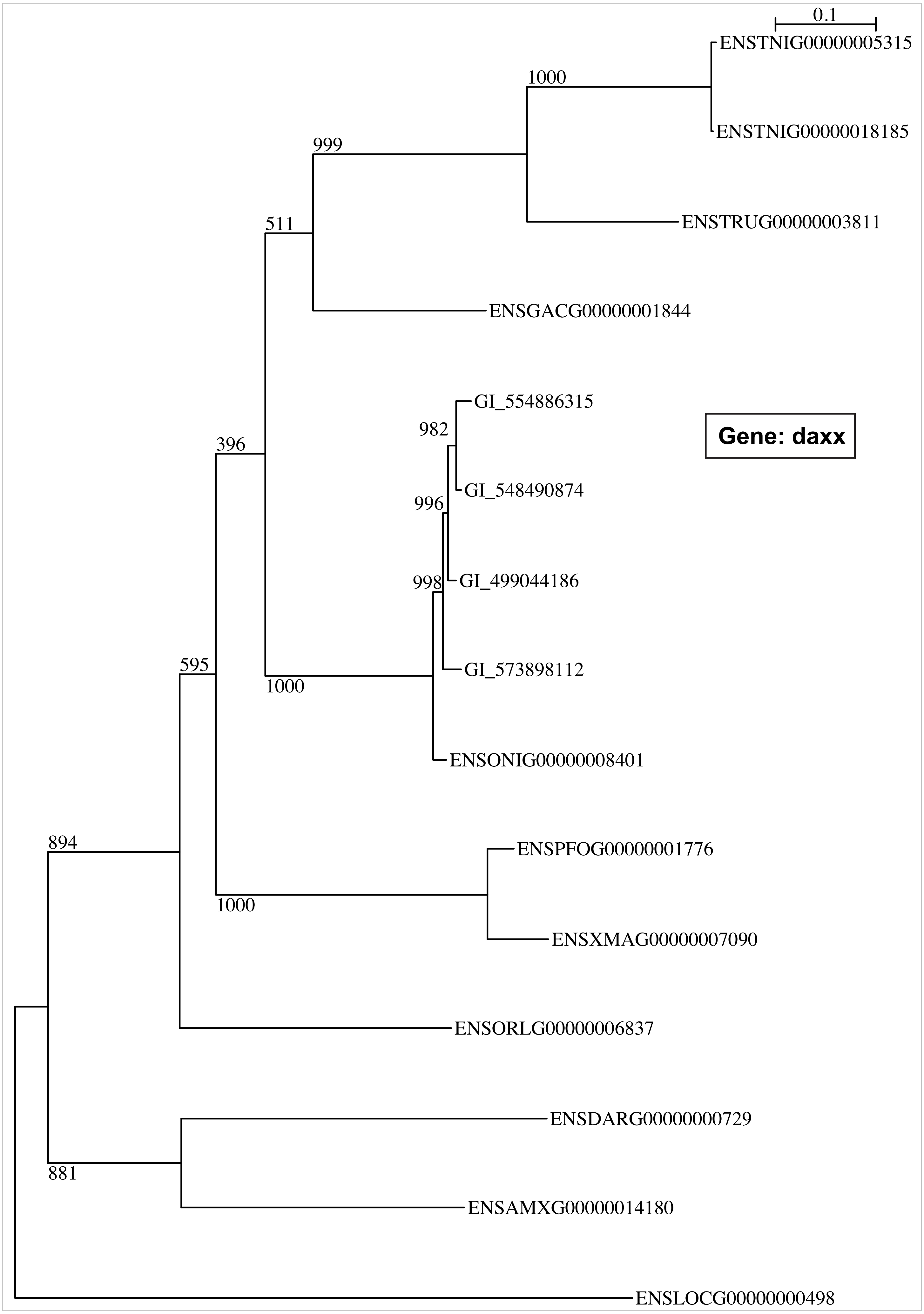
0.1
ENSTNIG00000005315
1000
ENSTNIG00000018185
999
ENSTRUG00000003811
511
ENSGACG00000001844
GI_554886315
982
Gene: daxx
396
GI_548490874
996
998
GI_499044186
595
GI_573898112
1000
ENSONIG00000008401
894
ENSPFOG00000001776
1000
ENSXMAG00000007090
ENSORLG00000006837
ENSDARG00000000729
881
ENSAMXG00000014180
ENSLOCG00000000498

Figure S1

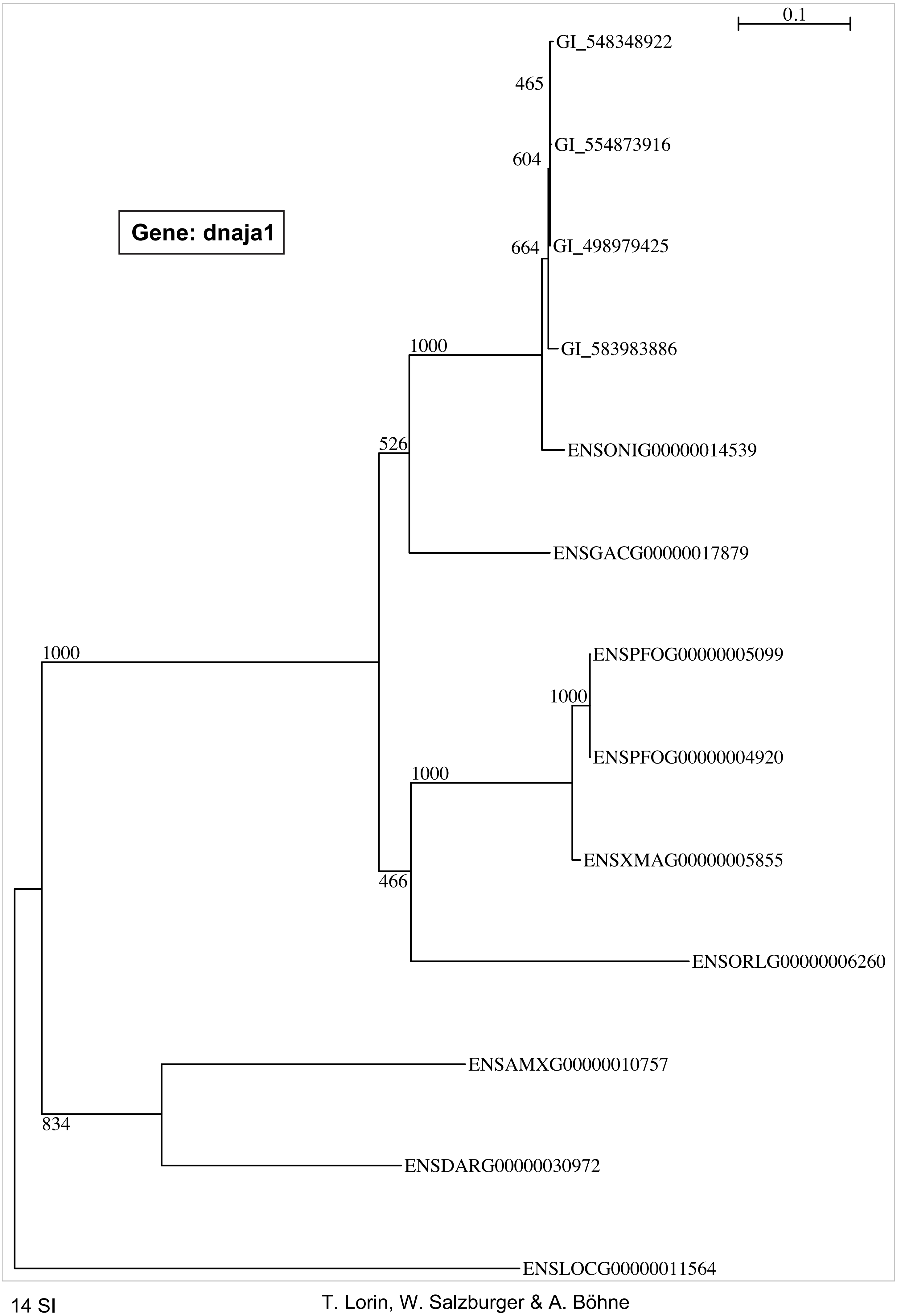
0.1
Gene: dnaja1
GI_548348922
465
GI_554873916
604
GI_498979425
664
GI_583983886
1000
ENSONIG00000014539
526
ENSGACG00000017879
1000
ENSPFOG00000005099
1000
ENSPFOG00000004920
1000
ENSXMAG00000005855
466
ENSORLG00000006260
ENSAMXG00000010757
834
ENSDARG00000030972
ENSLOCG00000011564

Figure S1

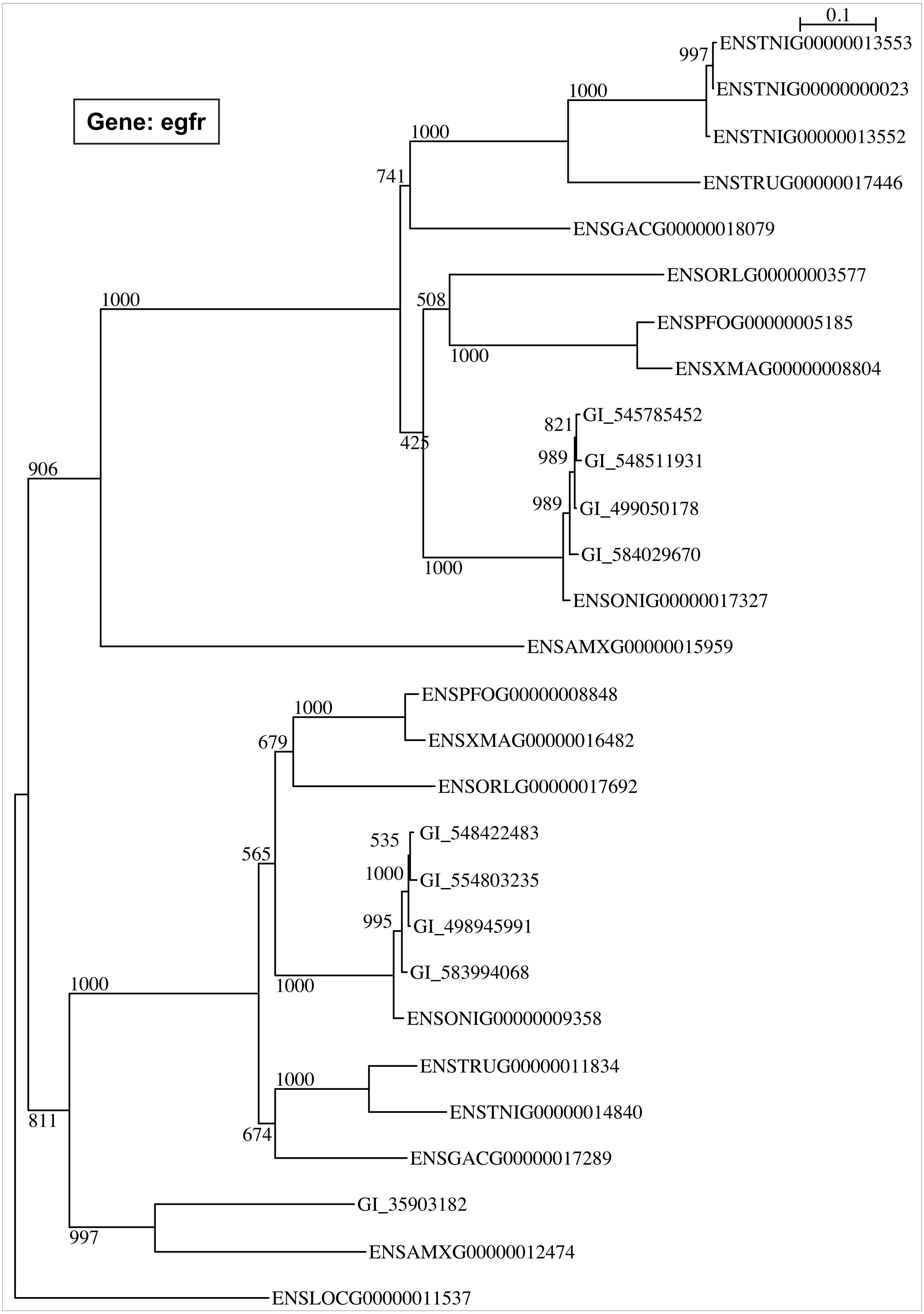
Gene: egfr
0.1
ENSTNIG00000013553
ENSTNIG00000000023
ENSTNIG00000013552
ENSTRUG00000017446
ENSGACG00000018079
ENSORLG00000003577
ENSPFOG00000005185
ENSXMAG00000008804
GI_545785452
GI_548511931
GI_499050178
GI_584029670
ENSONIG00000017327
ENSAMXG00000015959
ENSPFOG00000008848
ENSXMAG00000016482
ENSORLG00000017692
GI_548422483
GI_554803235
GI_498945991
GI_583994068
ENSONIG00000009358
ENSTRUG00000011834
ENSTNIG00000014840
ENSGACG00000017289
GI_35903182
ENSAMXG00000012474
ENSLOCG00000011537
997
1000
1000
741
1000
508
1000
821
425
989
906
989
1000
1000
679
535
565
1000
995
1000
1000
1000
811
674
997

Figure S1

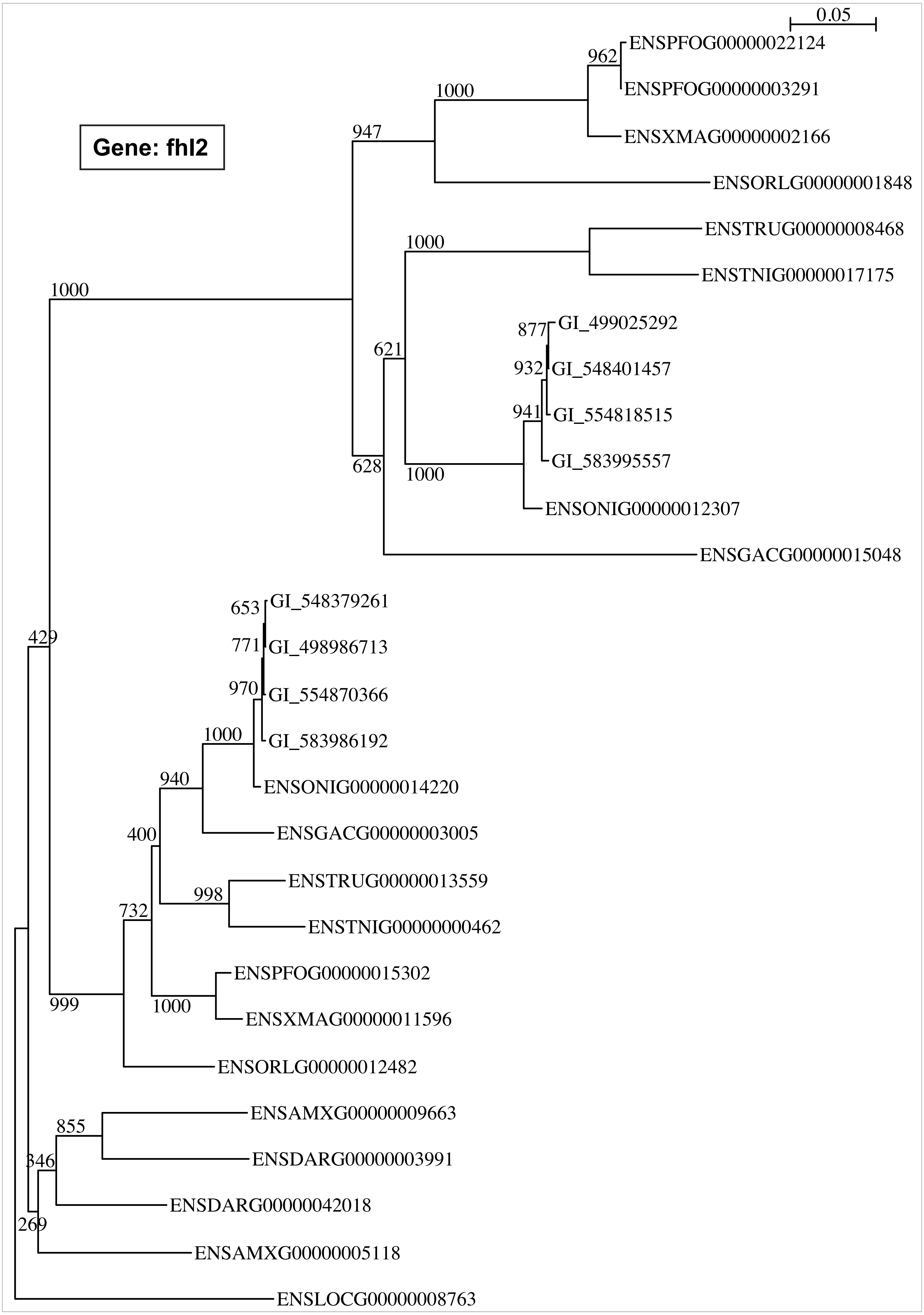
0.05
Gene: fhl2
ENSPFOG00000022124
ENSPFOG00000003291
ENSXMAG00000002166
ENSORLG00000001848
ENSTRUG00000008468
ENSTNIG00000017175
GI_499025292
GI_548401457
GI_554818515
GI_583995557
ENSONIG00000012307
ENSGACG00000015048
GI_548379261
GI_498986713
GI_554870366
GI_583986192
ENSONIG00000014220
ENSGACG00000003005
ENSTRUG00000013559
ENSTNIG00000000462
ENSPFOG00000015302
ENSXMAG00000011596
ENSORLG00000012482
ENSAMXG00000009663
ENSDARG00000003991
ENSDARG00000042018
ENSAMXG00000005118
ENSLOCG00000008763
962
1000
947
1000
1000
877
621
932
941
628
1000
653
429
771
970
1000
940
400
998
732
999
1000
855
346
269

Figure S1

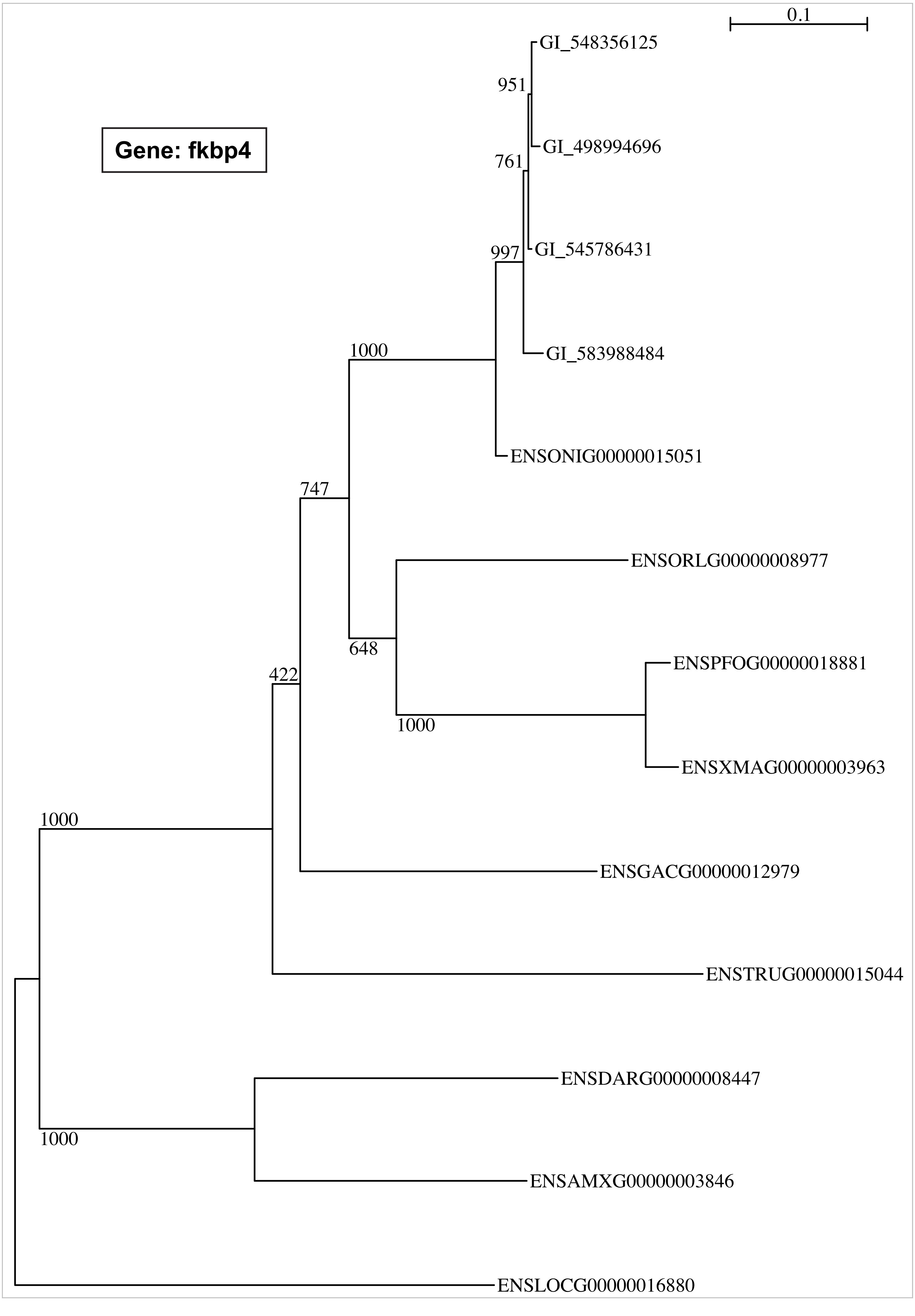

0.1
Gene: fkbp4
GI_548356125
951
GI_498994696
761
GI_545786431
997
GI_583988484
1000
ENSONIG00000015051
747
ENSORLG00000008977
648
ENSPFOG00000018881
422
1000
ENSXMAG00000003963
1000
ENSGACG00000012979
ENSTRUG00000015044
ENSDARG00000008447
1000
ENSAMXG00000003846
ENSLOCG00000016880

Figure S1

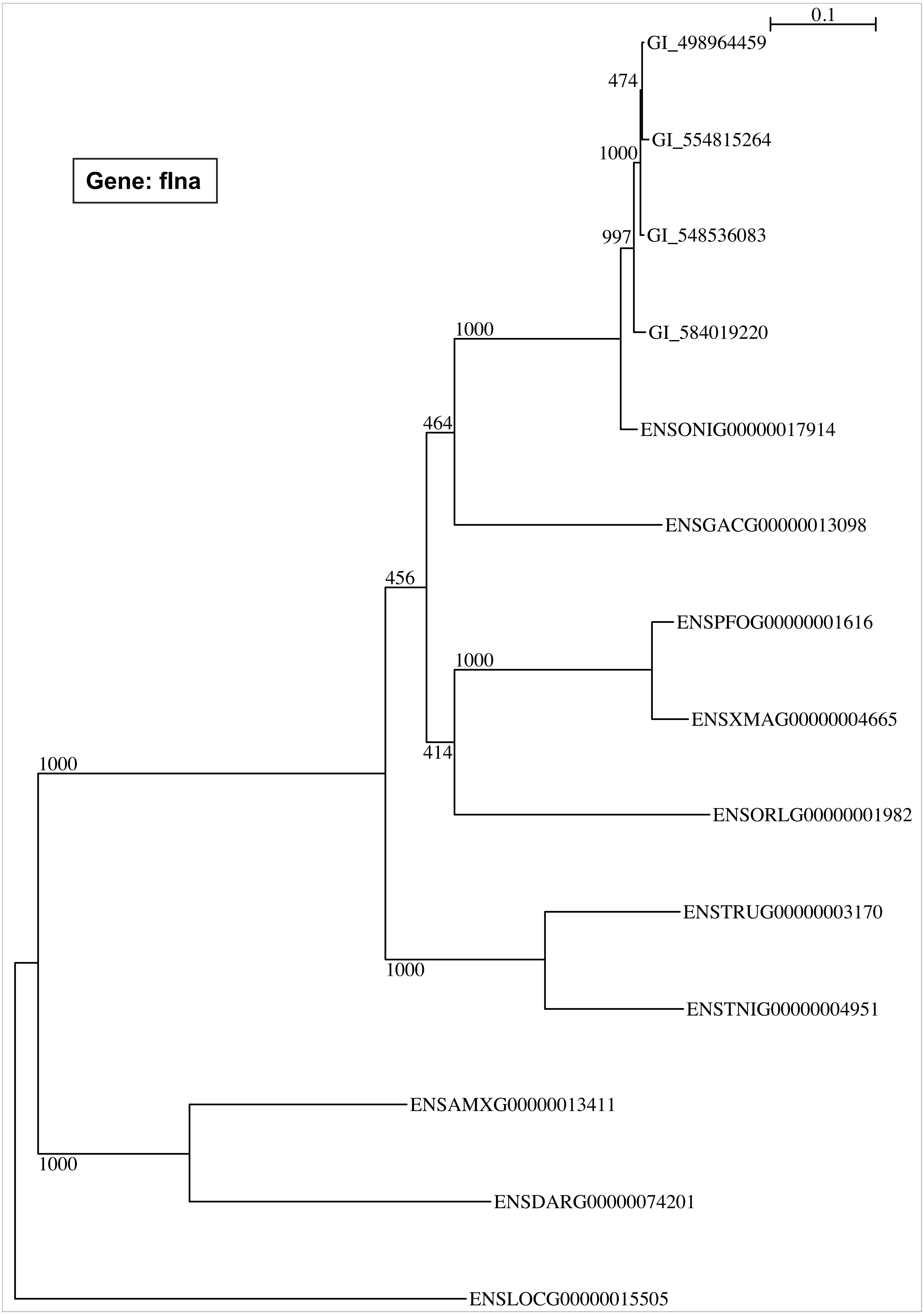

0.1
Gene: flna
GI_498964459
474
GI_554815264
1000
GI_548536083
997
GI_584019220
1000
ENSONIG00000017914
464
ENSGACG00000013098
456
ENSPFOG00000001616
1000
ENSXMAG00000004665
414
1000
ENSORLG00000001982
ENSTRUG00000003170
1000
ENSTNIG00000004951
ENSAMXG00000013411
1000
ENSDARG00000074201
ENSLOCG00000015505

Figure S1

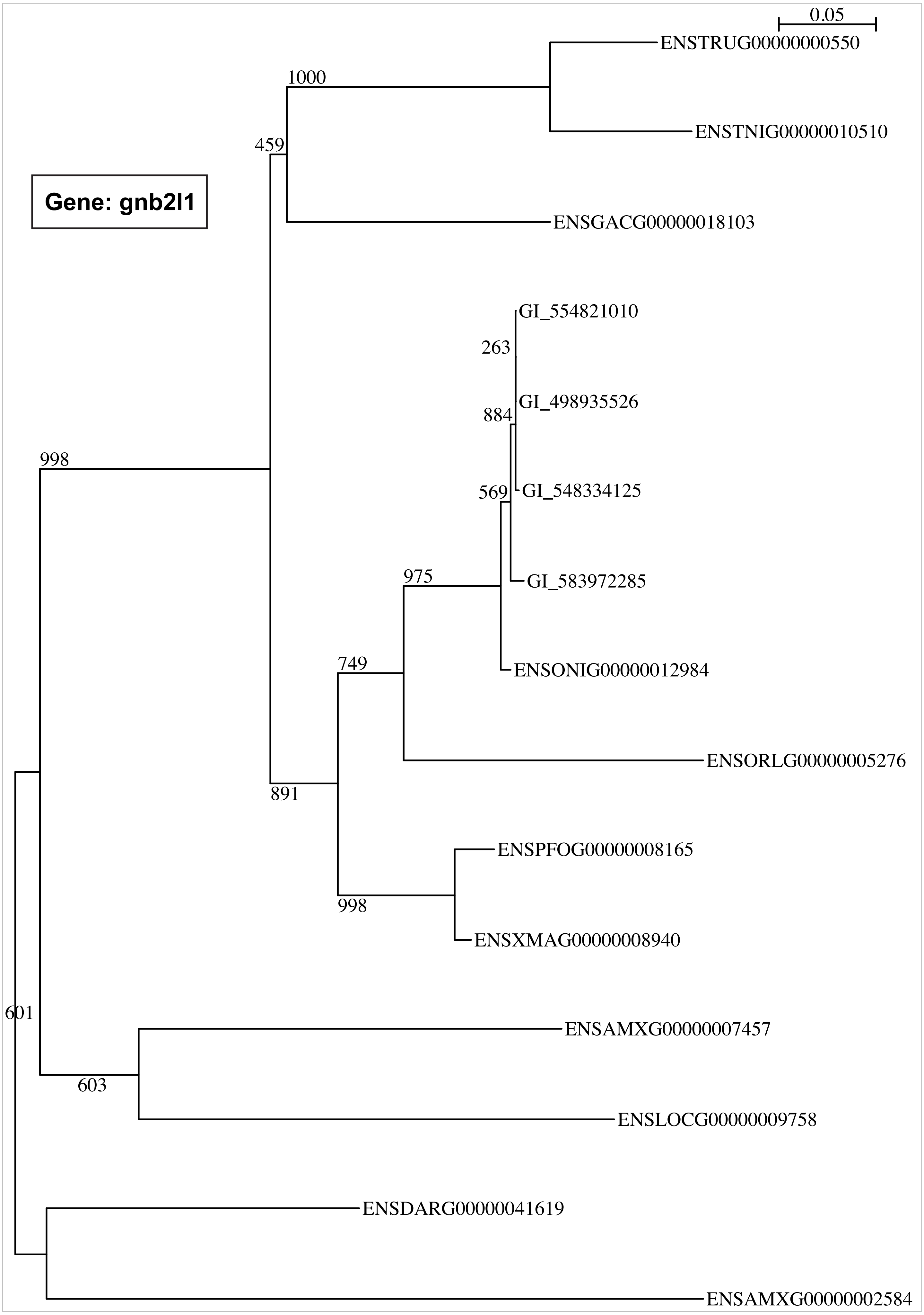
0.05
Gene: gnb2l1
1000
459
998
263
884
569
975
749
891
998
601
603
ENSTRUG00000000550
ENSTNIG00000010510
ENSGACG00000018103
GI_554821010
GI_498935526
GI_548334125
GI_583972285
ENSONIG00000012984
ENSORLG00000005276
ENSPFOG00000008165
ENSXMAG00000008940
ENSAMXG00000007457
ENSLOCG00000009758
ENSDARG00000041619
ENSAMXG00000002584

Figure S1

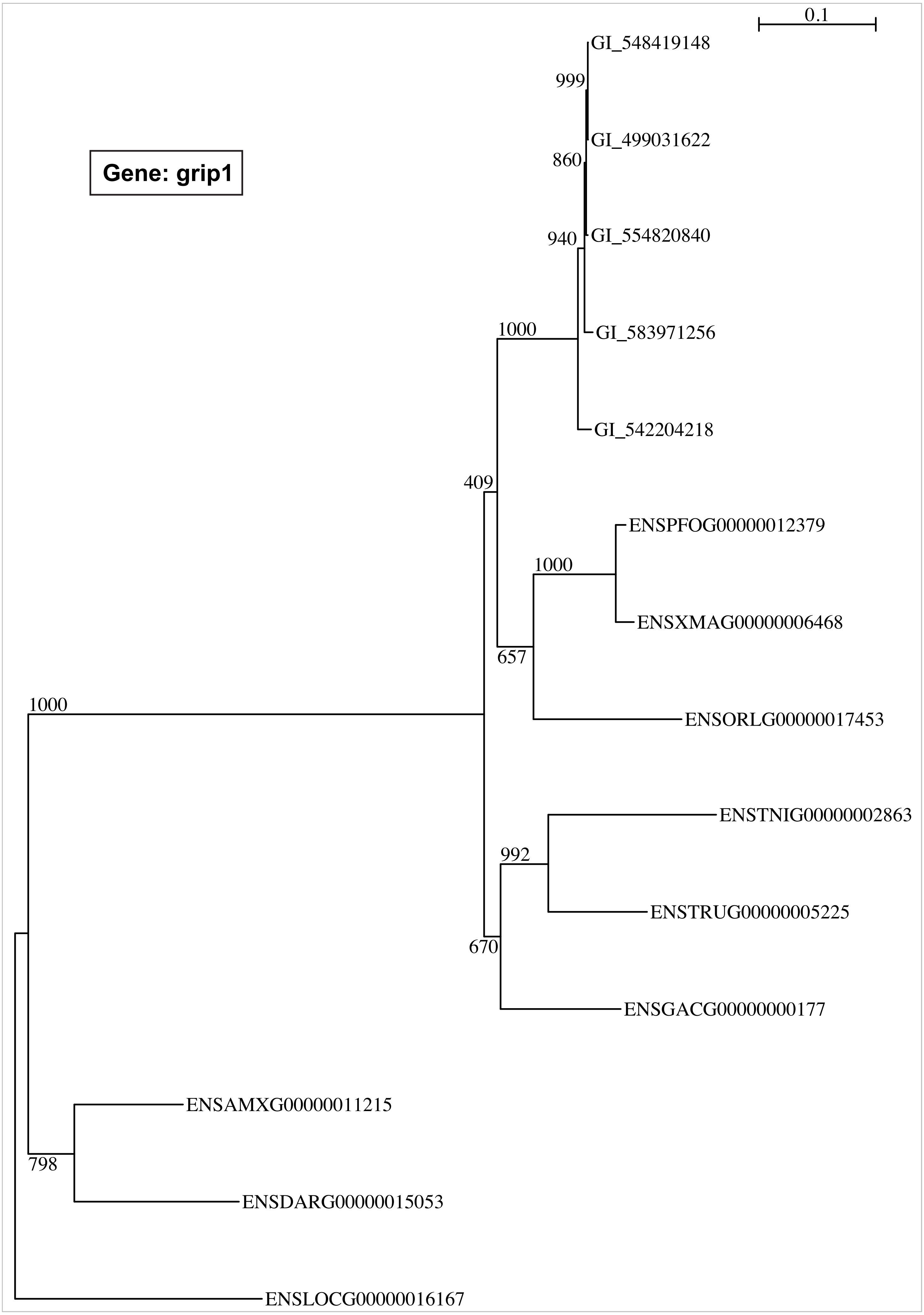

0.1
GI_548419148
999
GI_499031622
860
Gene: grip1
940
GI_554820840
1000
GI_583971256
GI_542204218
409
ENSPFOG00000012379
1000
ENSXMAG00000006468
657
1000
ENSORLG00000017453
ENSTNIG00000002863
992
ENSTRUG00000005225
670
ENSGACG00000000177
ENSAMXG00000011215
798
ENSDARG00000015053
ENSLOCG00000016167

Figure S1

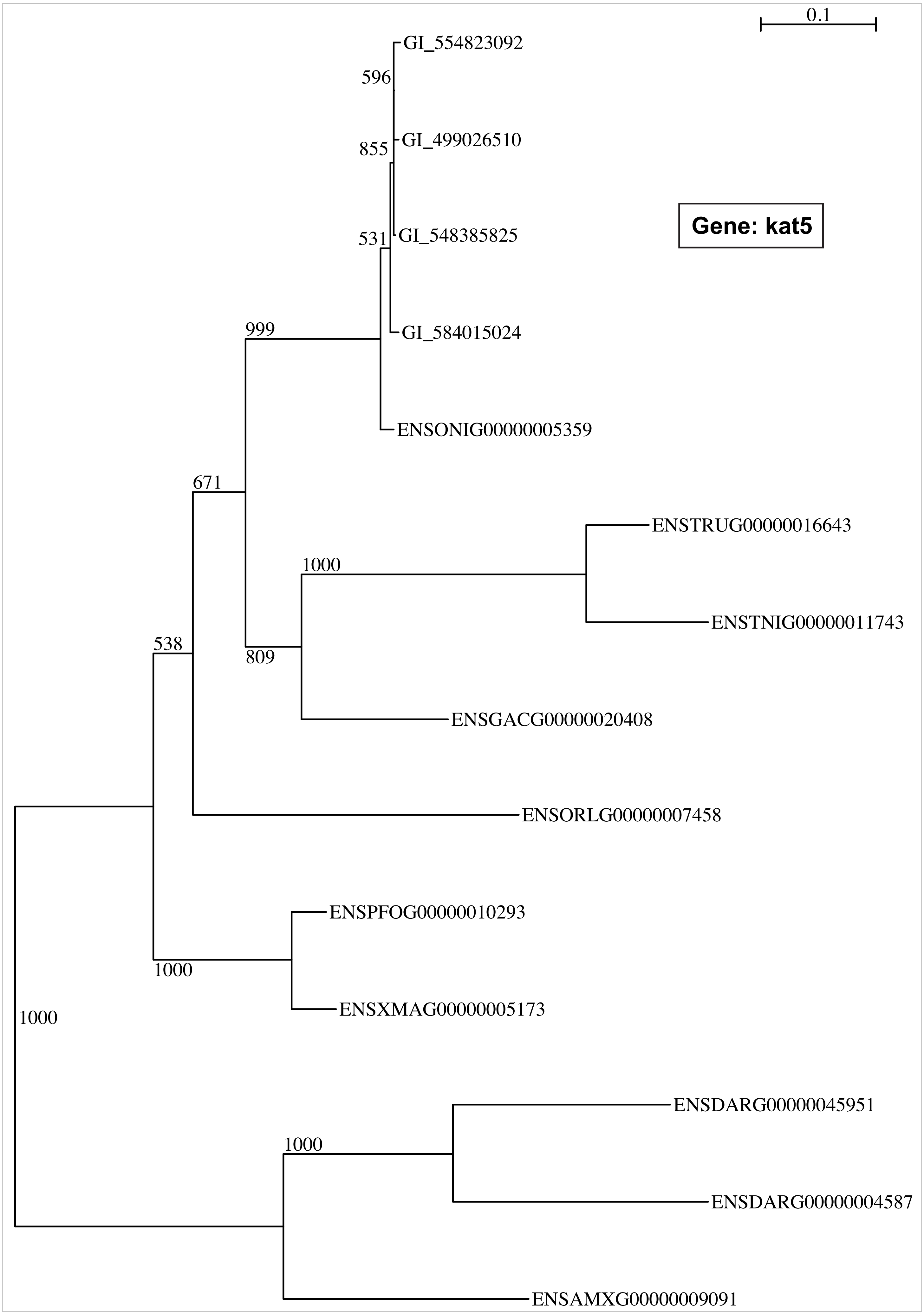
0.1
Gene: kat5
GI_554823092
596
GI_499026510
855
GI_548385825
531
GI_584015024
999
ENSONIG00000005359
671
ENSTRUG00000016643
1000
ENSTNIG00000011743
538
809
ENSGACG00000020408
ENSORLG00000007458
ENSPFOG00000010293
1000
ENSXMAG00000005173
1000
ENSDARG00000045951
1000
ENSDARG00000004587
ENSAMXG00000009091

Figure S1

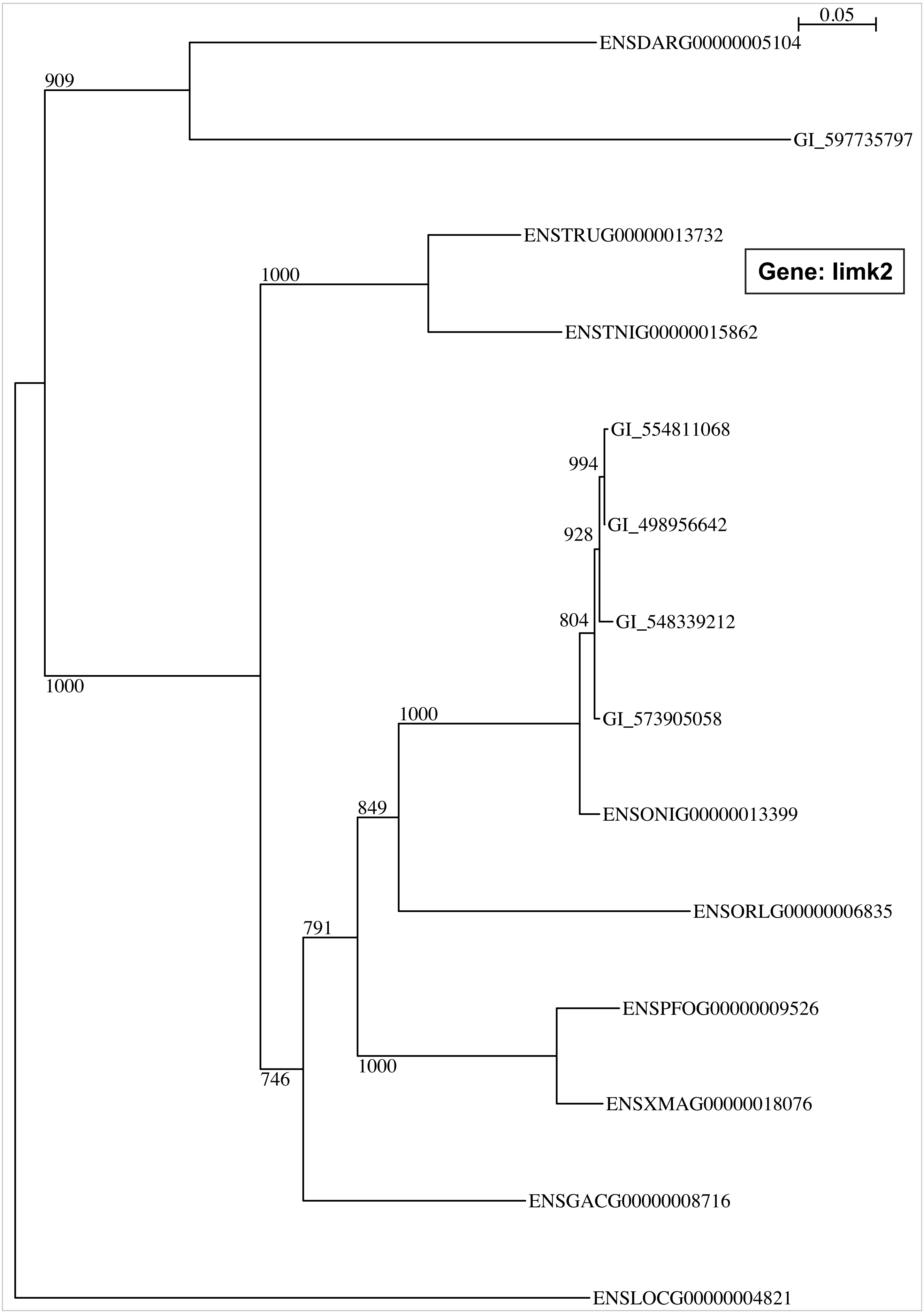
0.05
ENSDARG00000005104
909
GI_597735797
ENSTRUG00000013732
1000
Gene: limk2
ENSTNIG00000015862
GI_554811068
994
GI_498956642
928
804
GI_548339212
1000
GI_573905058
1000
849
ENSONIG00000013399
ENSORLG00000006835
791
ENSPFOG00000009526
1000
746
ENSXMAG00000018076
ENSGACG00000008716
ENSLOCG00000004821

Figure S1

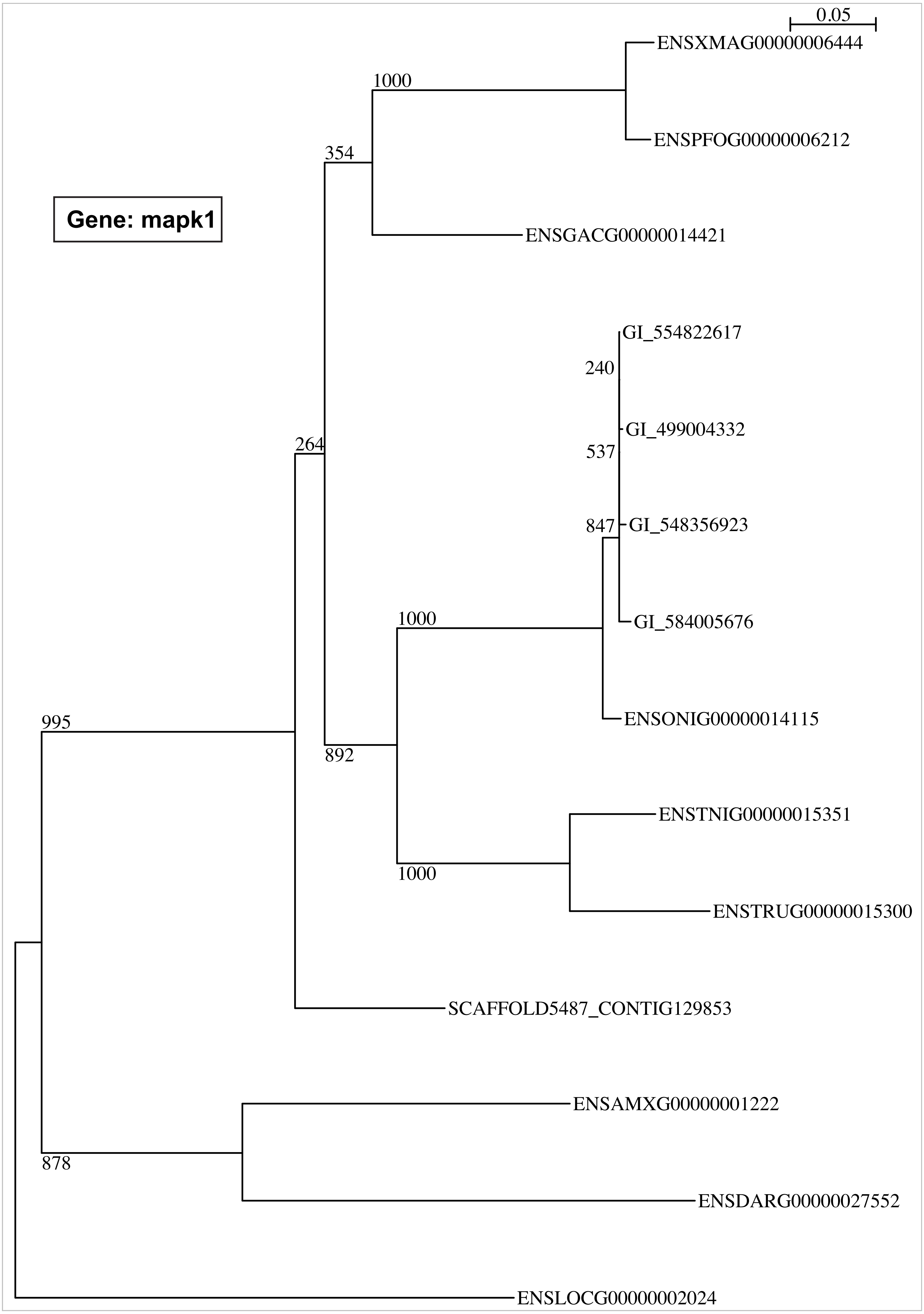

0.05
ENSXMAG00000006444
1000
ENSPFOG00000006212
354
Gene: mapk1
ENSGACG00000014421
GI_554822617
240
GI_499004332
264
537
847
GI_548356923
1000
GI_584005676
995
ENSONIG00000014115
892
ENSTNIG00000015351
1000
ENSTRUG00000015300
SCAFFOLD5487_CONTIG129853
ENSAMXG00000001222
878
ENSDARG00000027552
ENSLOCG00000002024

Figure S1

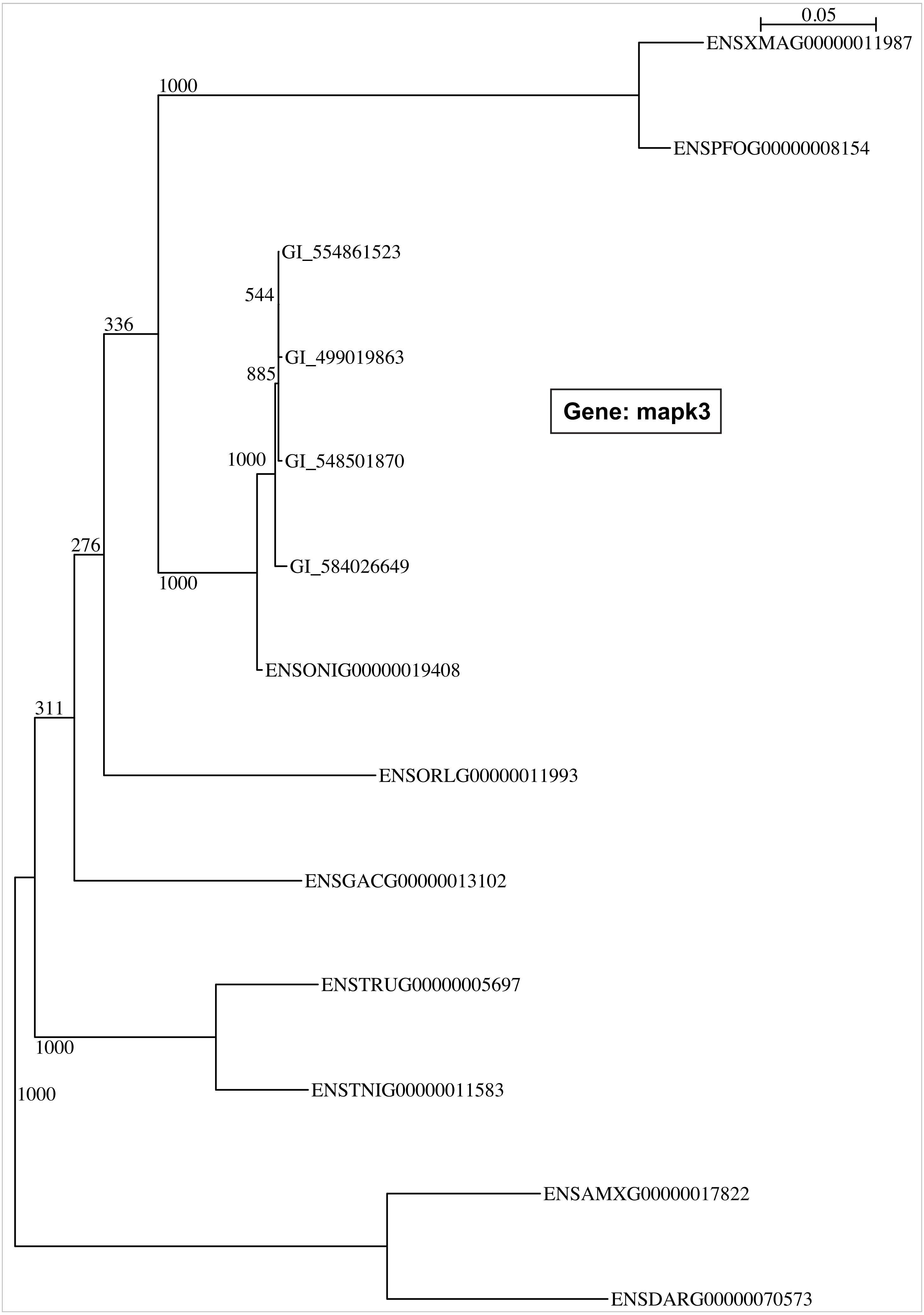
0.05
ENSXMAG00000011987
1000
ENSPFOG00000008154
GI_554861523
544
336
GI_499019863
885
Gene: mapk3
1000
GI_548501870
276
GI_584026649
1000
ENSONIG00000019408
311
ENSORLG00000011993
ENSGACG00000013102
ENSTRUG00000005697
1000
ENSTNIG00000011583
1000
ENSAMXG00000017822
ENSDARG00000070573

Figure S1

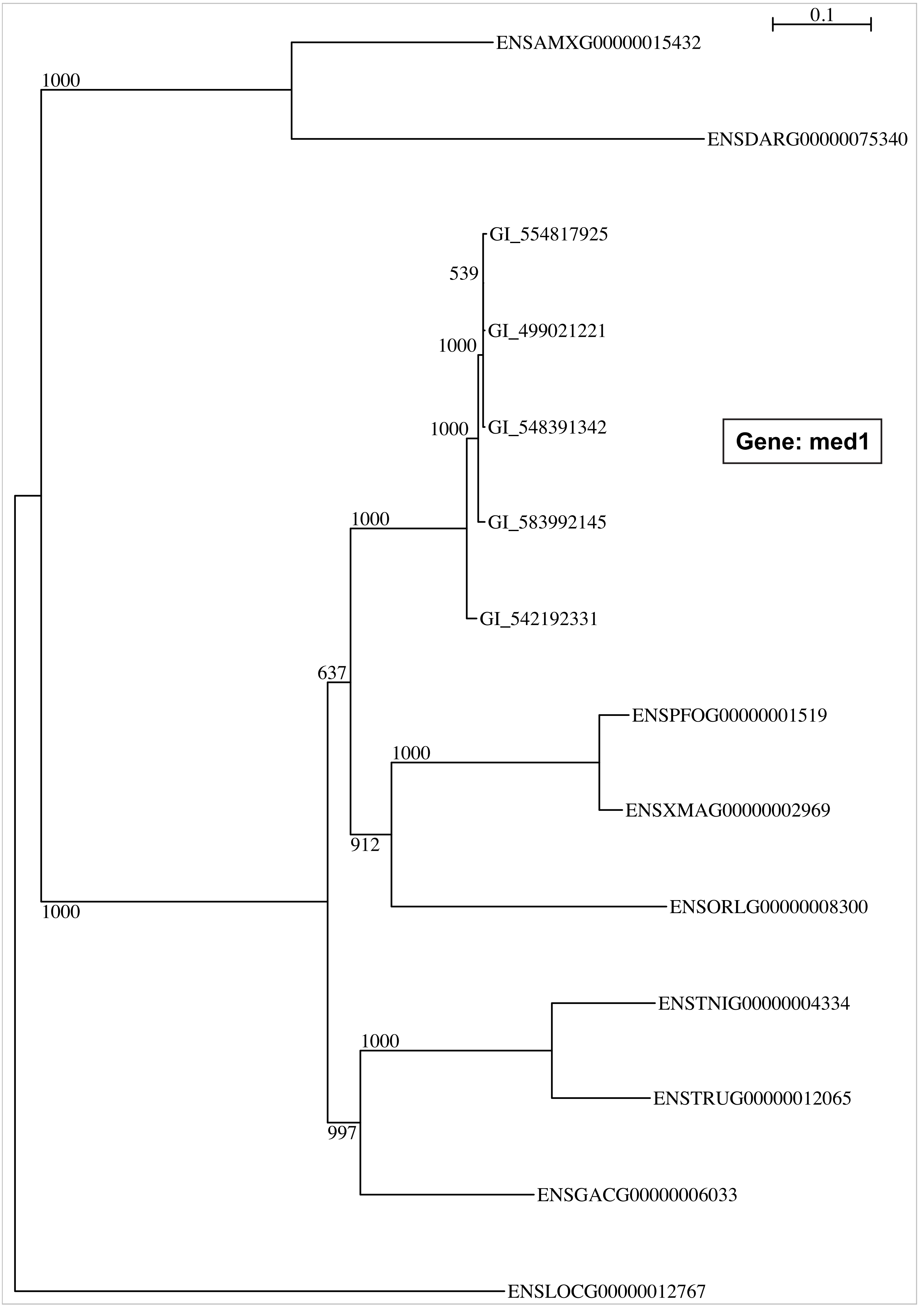

T. Lorin, W. Salzburger & A. Böhne

Figure S1

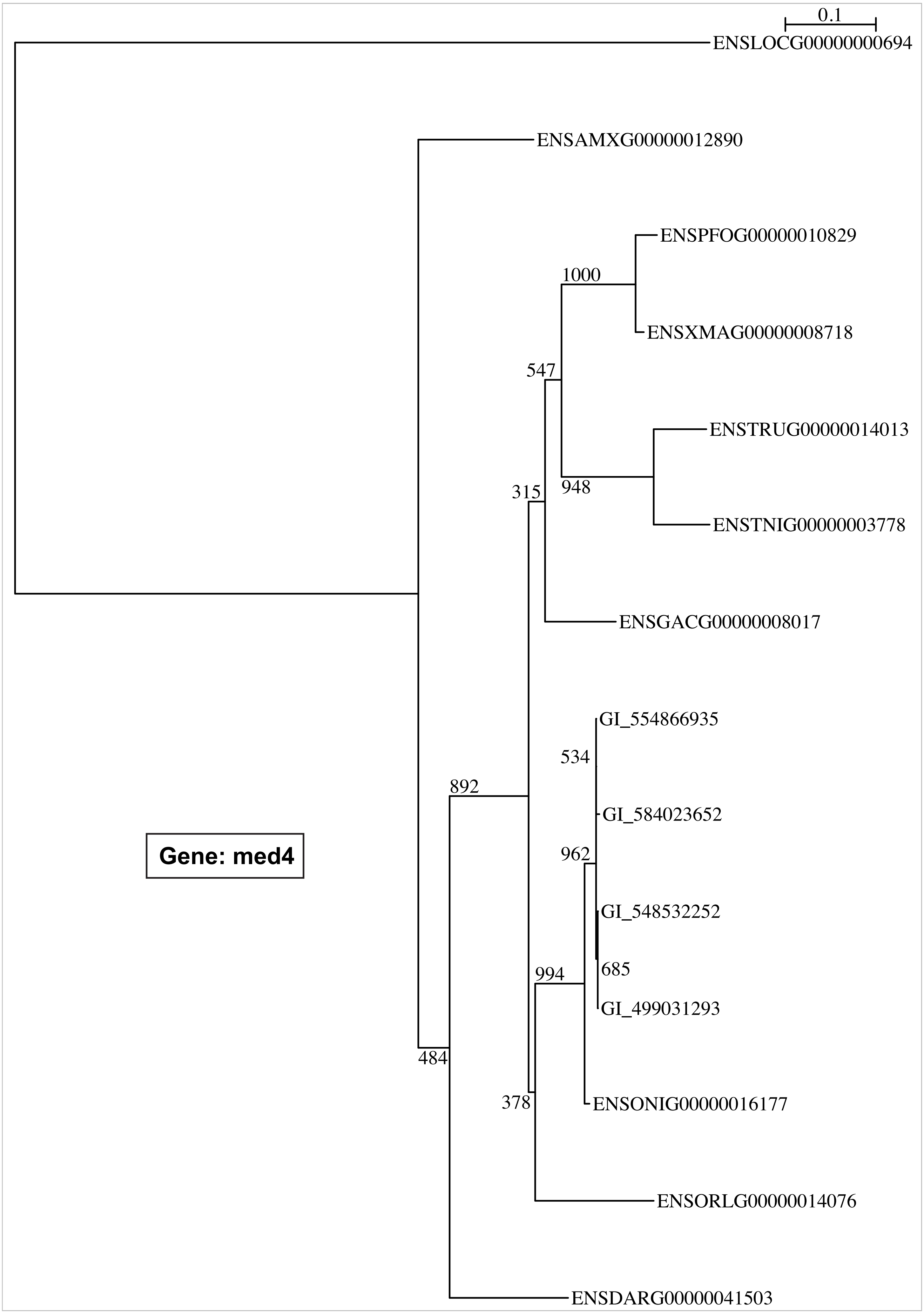
0.1
ENSLOCG00000000694
ENSAMXG00000012890
ENSPFOG00000010829
1000
ENSXMAG00000008718
547
ENSTRUG00000014013
315
948
ENSTNIG00000003778
ENSGACG00000008017
GI_554866935
534
892
GI_584023652
Gene: med4
962
GI_548532252
994
685
GI_499031293
484
378
ENSONIG00000016177
ENSORLG00000014076
ENSDARG00000041503

Figure S1

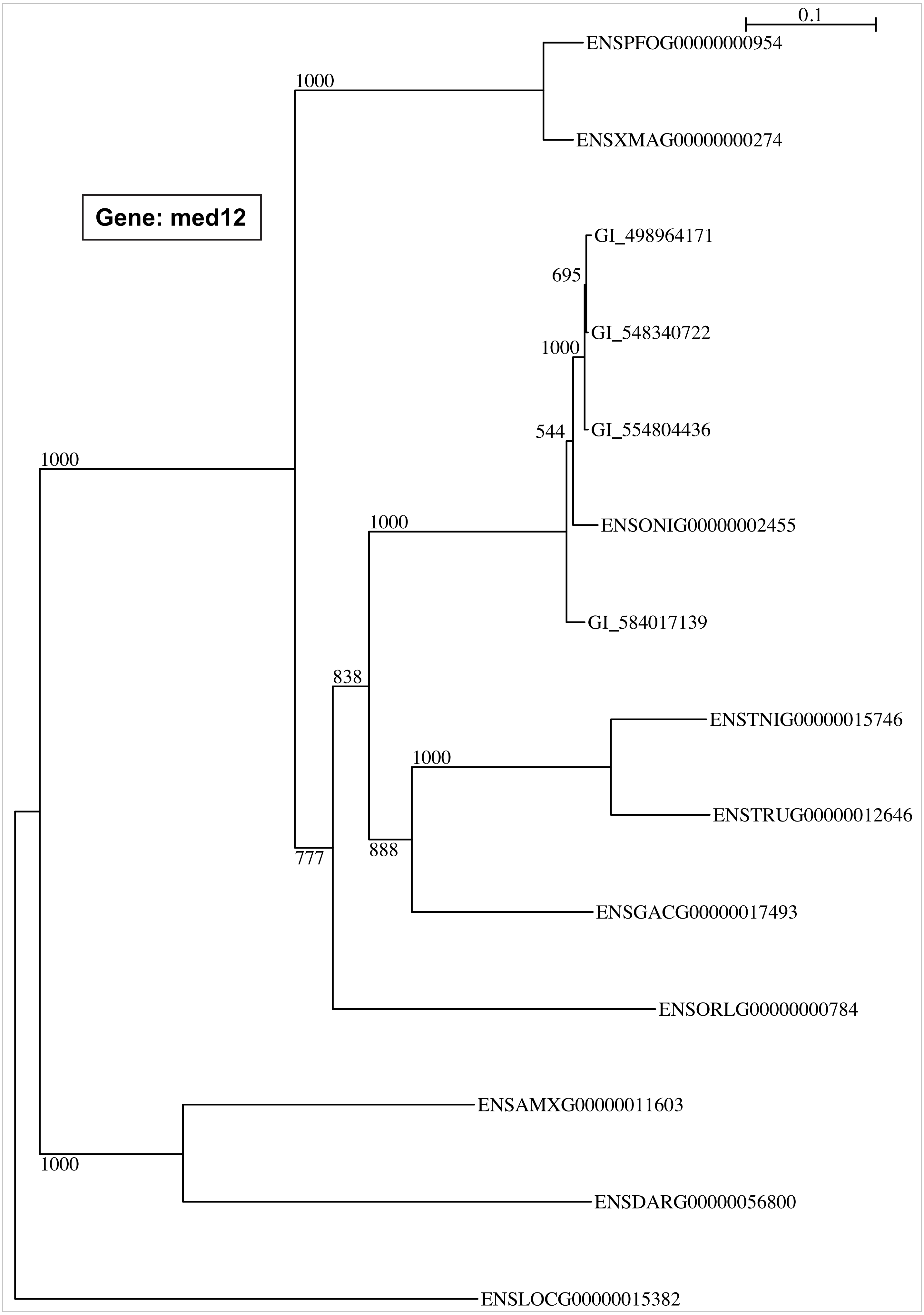
0.1
ENSPFOG00000000954
1000
ENSXMAG00000000274
Gene: med12
GI_498964171
695
GI_548340722
1000
GI_554804436
544
1000
ENSONIG00000002455
1000
GI_584017139
838
ENSTNIG00000015746
1000
ENSTRUG00000012646
777
888
ENSGACG00000017493
ENSORLG00000000784
ENSAMXG00000011603
1000
ENSDARG00000056800
ENSLOCG00000015382

Figure S1

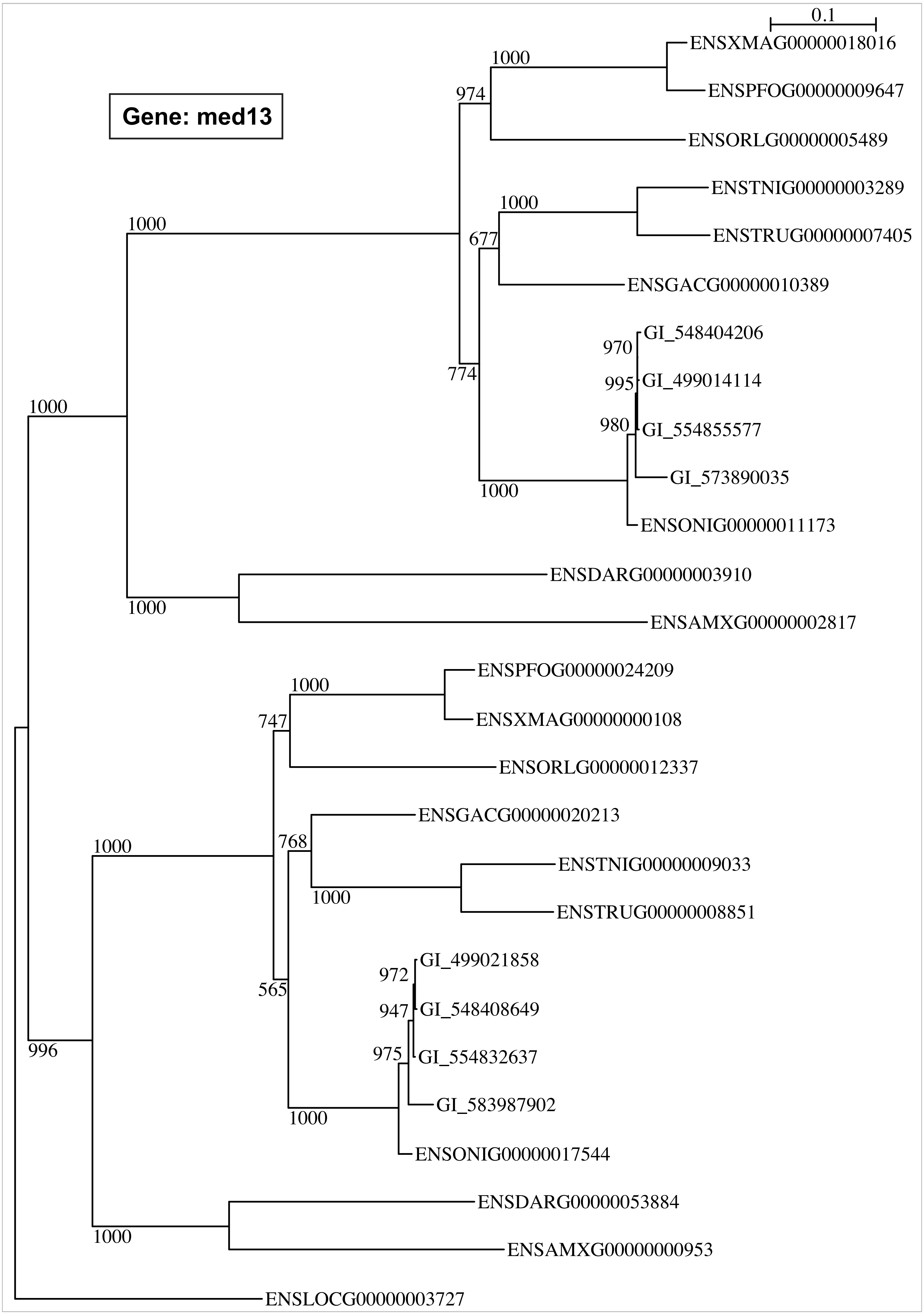

0.1
Gene: med13
ENSXMAG00000018016
ENSPFOG00000009647
ENSORLG00000005489
ENSTNIG00000003289
ENSTRUG00000007405
ENSGACG00000010389
GI_548404206
GI_499014114
GI_554855577
GI_573890035
ENSONIG00000011173
ENSDARG00000003910
ENSAMXG00000002817
ENSPFOG00000024209
ENSXMAG00000000108
ENSORLG00000012337
ENSGACG00000020213
ENSTNIG00000009033
ENSTRUG00000008851
GI_499021858
GI_548408649
GI_554832637
GI_583987902
ENSONIG00000017544
ENSDARG00000053884
ENSAMXG00000000953
ENSLOCG00000003727
1000
974
1000
677
1000
970
995
980
774
1000
1000
1000
1000
747
1000
768
1000
565
972
947
975
1000
1000
996
1000

Figure S1

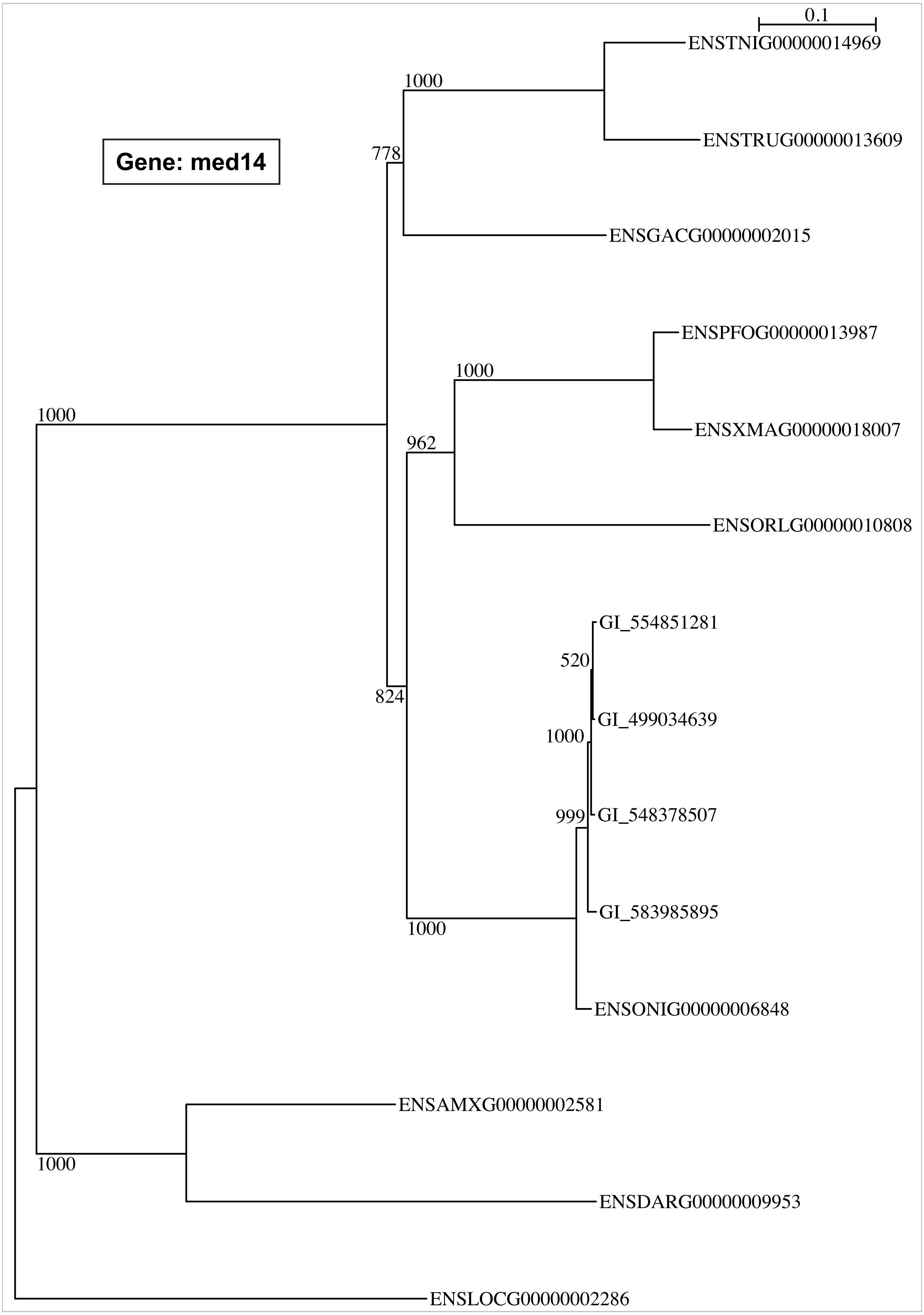
0.1
Gene: med14
ENSTNIG00000014969
ENSTRUG00000013609
ENSGACG00000002015
ENSPFOG00000013987
ENSXMAG00000018007
ENSORLG00000010808
GI_554851281
GI_499034639
GI_548378507
GI_583985895
ENSONIG00000006848
ENSAMXG00000002581
ENSDARG00000009953
ENSLOCG00000002286
1000
778
1000
962
1000
520
1000
824
999
1000
1000

Figure S1

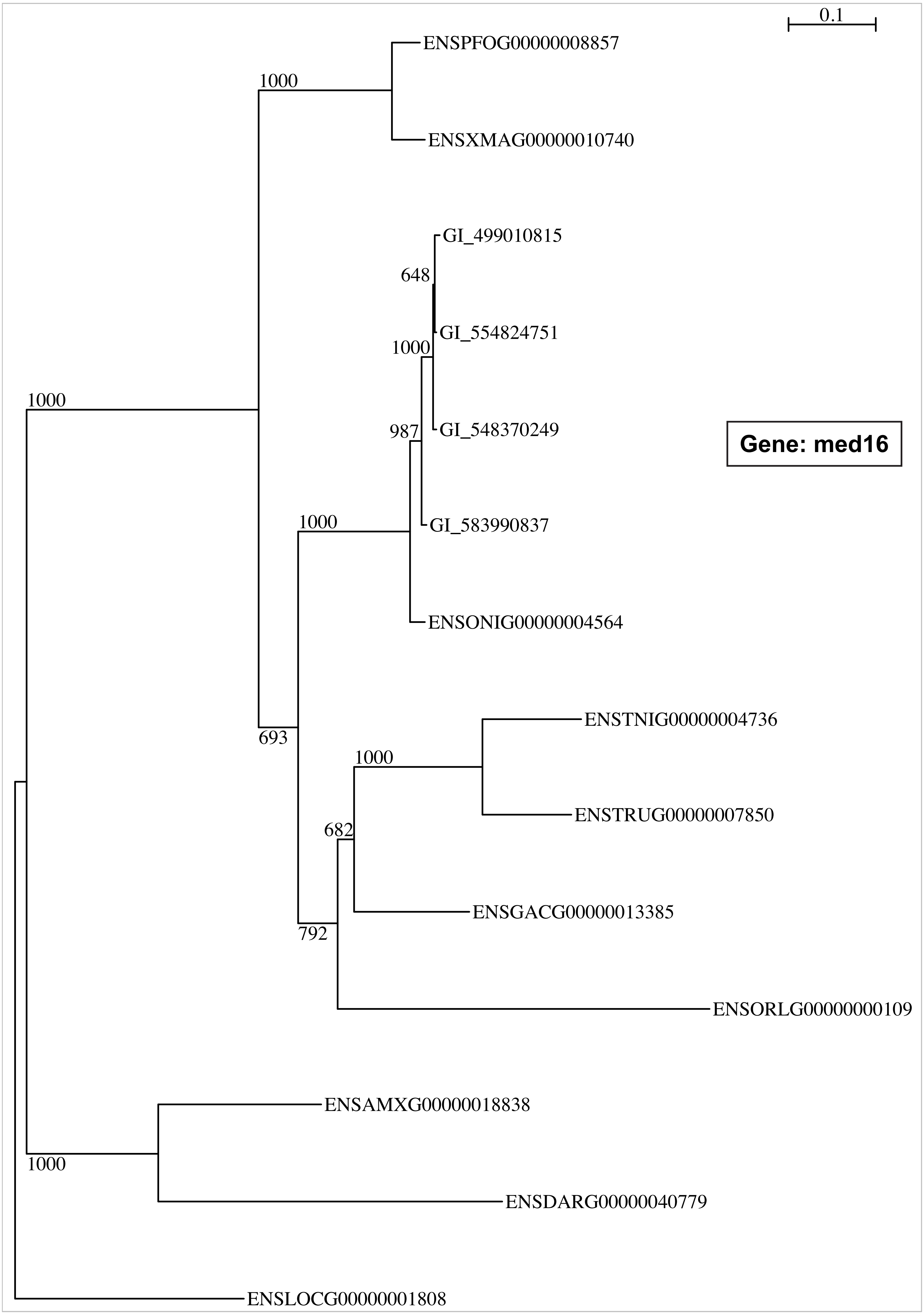
0.1
ENSPFOG00000008857
1000
ENSXMAG00000010740
GI_499010815
648
GI_554824751
1000
1000
GI_548370249
987
Gene: med16
1000
GI_583990837
ENSONIG00000004564
ENSTNIG00000004736
693
1000
ENSTRUG00000007850
682
ENSGACG00000013385
792
ENSORLG00000000109
ENSAMXG00000018838
1000
ENSDARG00000040779
ENSLOCG00000001808

Figure S1

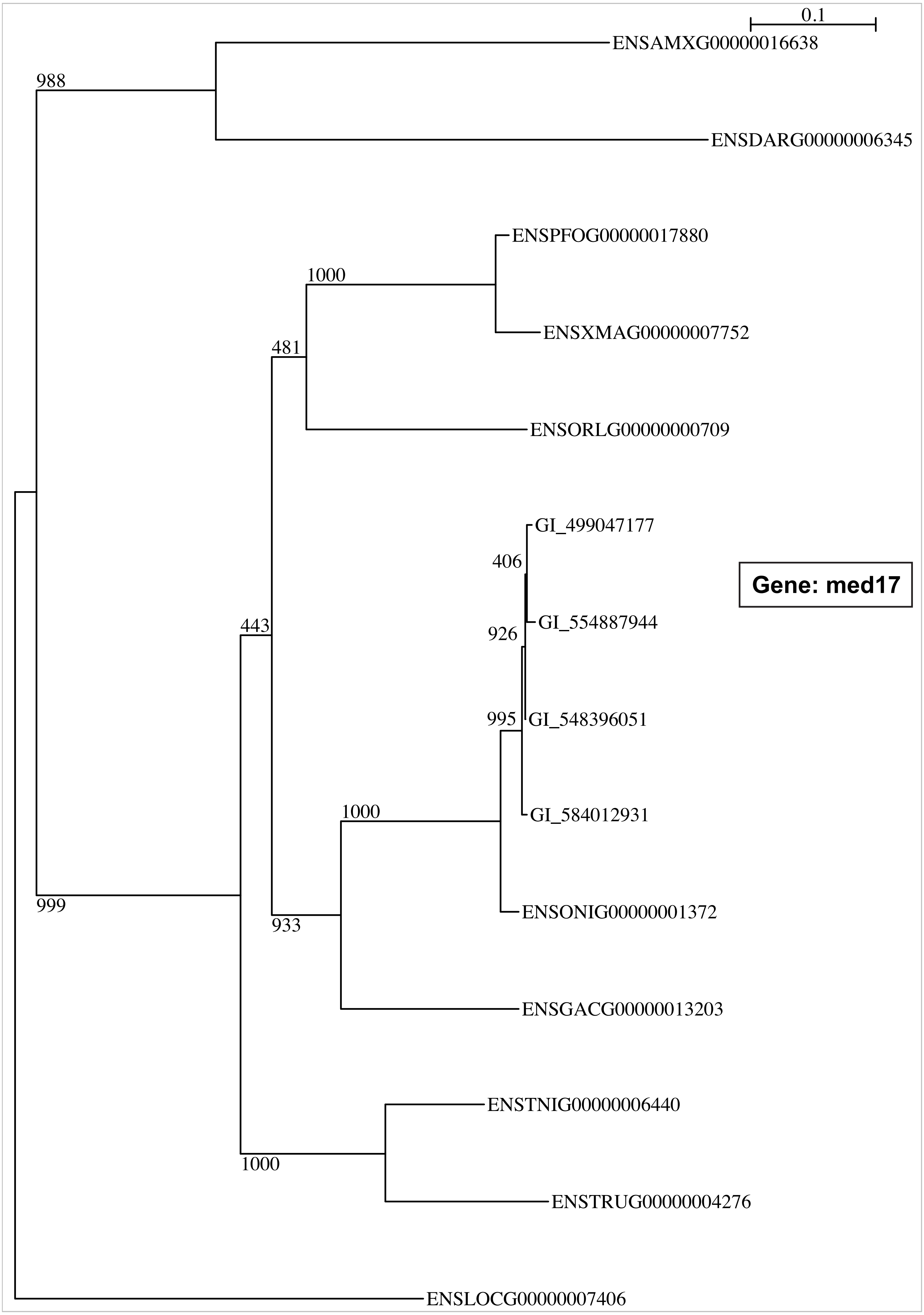
0.1
ENSAMXG00000016638
988
ENSDARG00000006345
ENSPFOG00000017880
1000
ENSXMAG00000007752
481
ENSORLG00000000709
GI_499047177
406
Gene: med17
443
GI_554887944
926
995
GI_548396051
1000
GI_584012931
999
ENSONIG00000001372
933
ENSGACG00000013203
ENSTNIG00000006440
1000
ENSTRUG00000004276
ENSLOCG00000007406

Figure S1

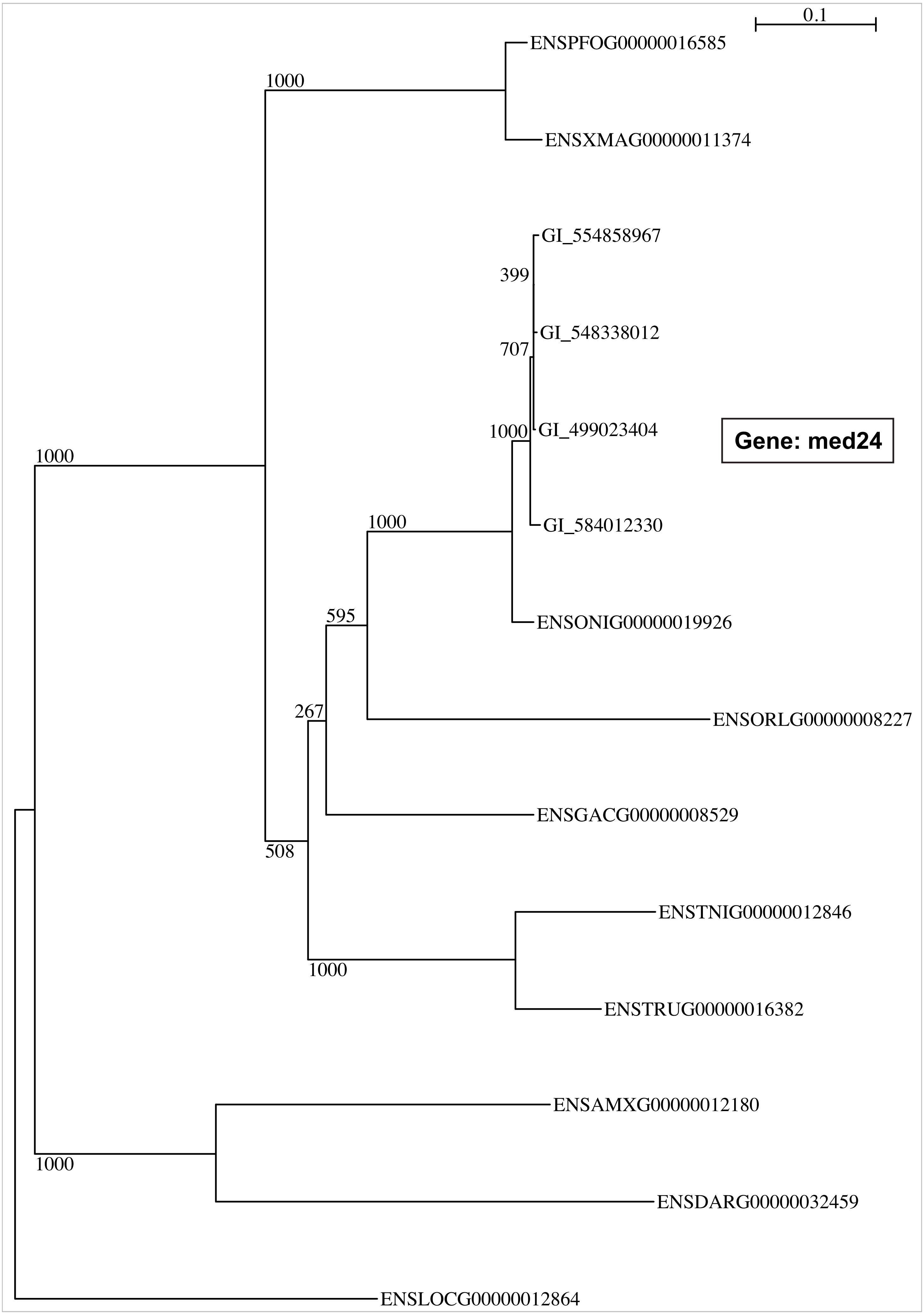
0.1
ENSPFOG00000016585
1000
ENSXMAG00000011374
GI_554858967
399
GI_548338012
707
1000
GI_499023404
Gene: med24
1000
GI_584012330
1000
ENSONIG00000019926
595
267
ENSORLG00000008227
ENSGACG00000008529
508
ENSTNIG00000012846
1000
ENSTRUG00000016382
ENSAMXG00000012180
1000
ENSDARG00000032459
ENSLOCG00000012864

Figure S1

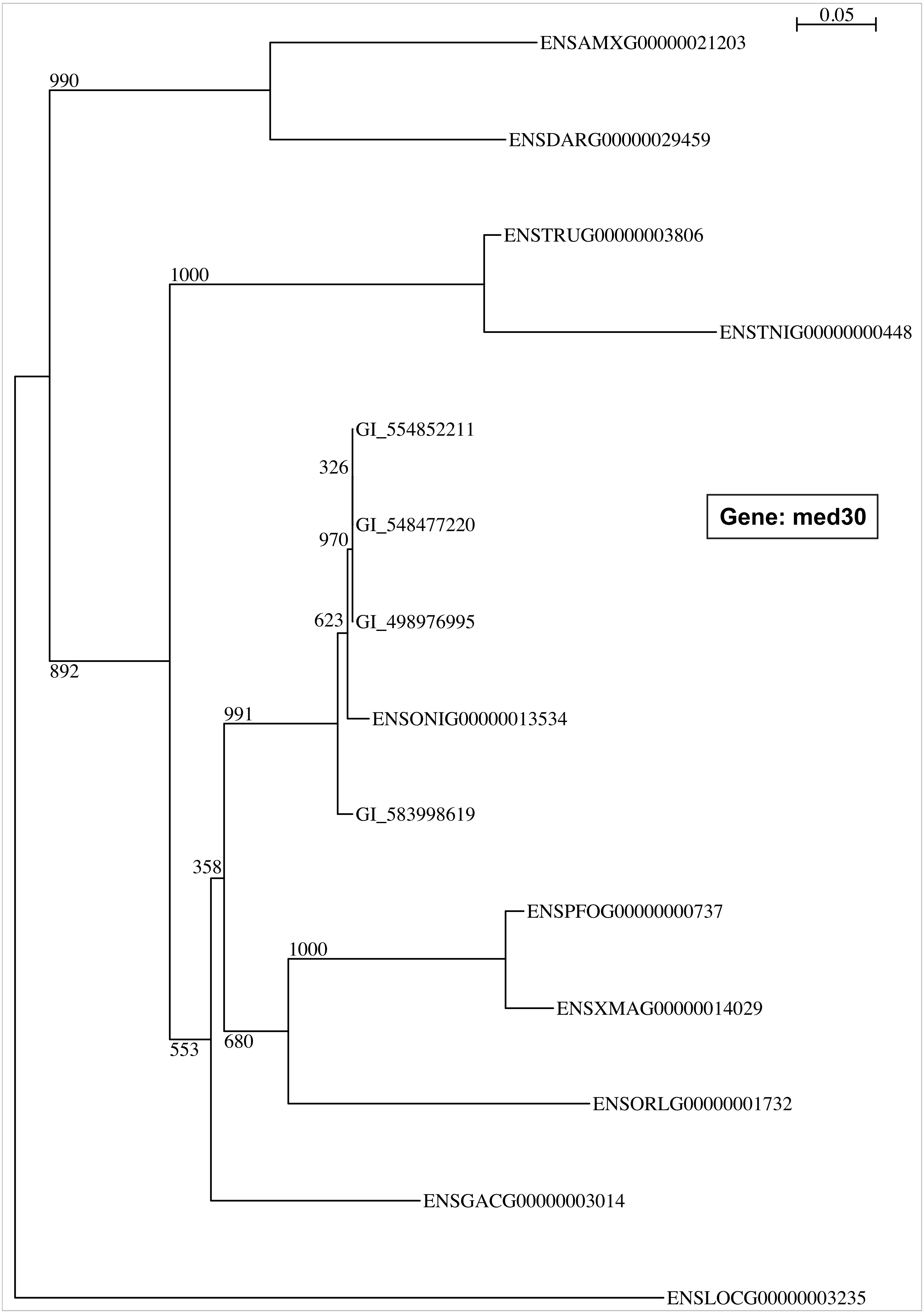
0.05
ENSAMXG00000021203
ENSDARG00000029459
ENSTRUG00000003806
ENSTNIG00000000448
GI_554852211
GI_548477220
GI_498976995
ENSONIG00000013534
GI_583998619
ENSPFOG00000000737
ENSXMAG00000014029
ENSORLG00000001732
ENSGACG00000003014
ENSLOCG00000003235
990
1000
892
326
970
623
991
358
1000
680
553
Gene: med30

Figure S1

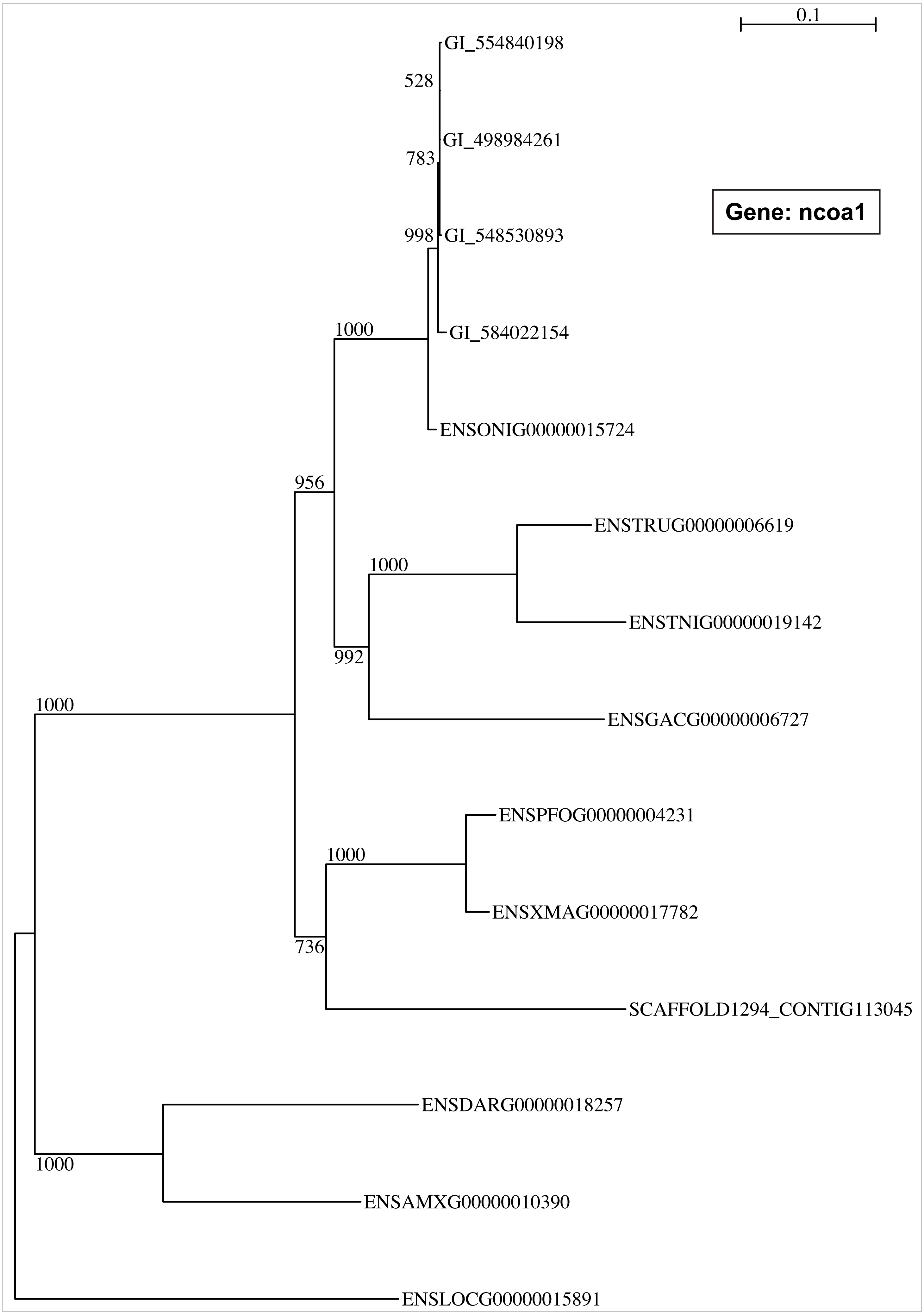
0.1
Gene: ncoa1
GI_554840198
528
GI_498984261
783
998
GI_548530893
GI_584022154
1000
ENSONIG00000015724
956
ENSTRUG00000006619
1000
ENSTNIG00000019142
992
ENSGACG00000006727
1000
ENSPFOG00000004231
1000
ENSXMAG00000017782
736
SCAFFOLD1294_CONTIG113045
ENSDARG00000018257
1000
ENSAMXG00000010390
ENSLOCG00000015891

Figure S1

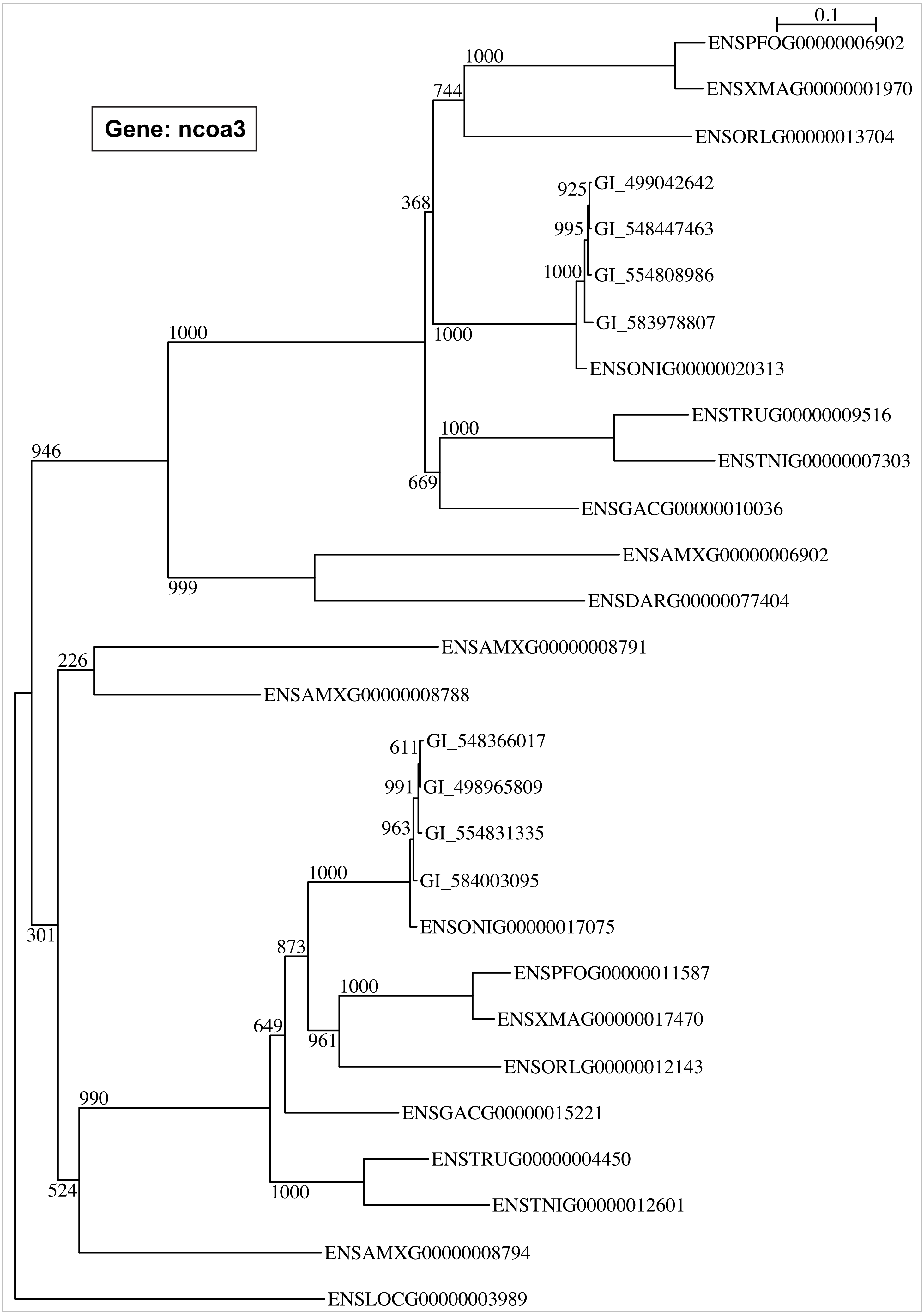
Gene: ncoa3
0.1
1000
744
368
1000
946
925
995
1000
1000
669
999
226
301
611
991
963
1000
873
1000
649
961
990
524
1000
ENSPFOG00000006902
ENSXMAG00000001970
ENSORLG00000013704
GI_499042642
GI_548447463
GI_554808986
GI_583978807
ENSONIG00000020313
ENSTRUG00000009516
ENSTNIG00000007303
ENSGACG00000010036
ENSAMXG00000006902
ENSDARG00000077404
ENSAMXG00000008791
ENSAMXG00000008788
GI_548366017
GI_498965809
GI_554831335
GI_584003095
ENSONIG00000017075
ENSPFOG00000011587
ENSXMAG00000017470
ENSORLG00000012143
ENSGACG00000015221
ENSTRUG00000004450
ENSTNIG00000012601
ENSAMXG00000008794
ENSLOCG00000003989

Figure S1

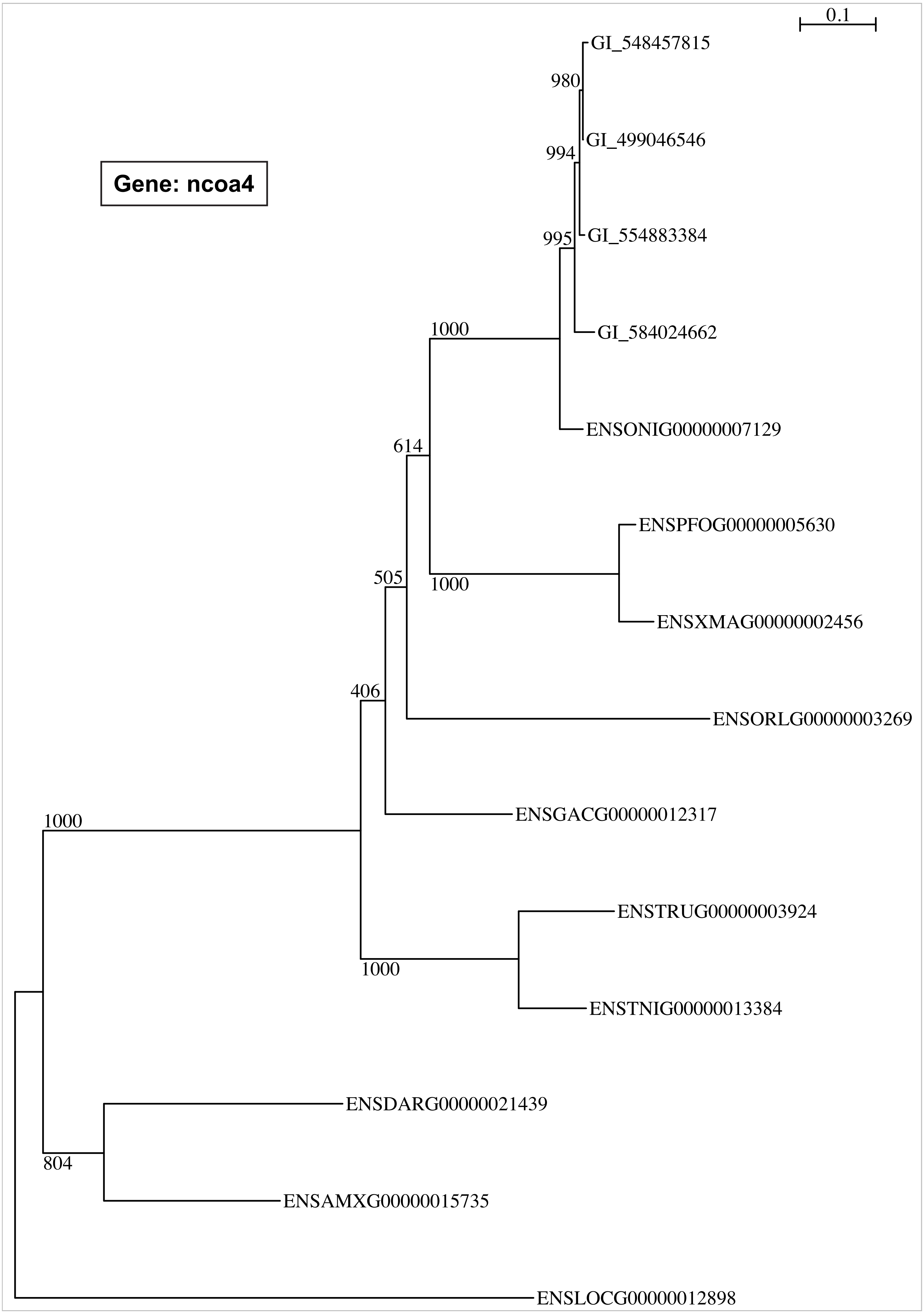
0.1
Gene: ncoa4
GI_548457815
980
GI_499046546
994
GI_554883384
995
GI_584024662
1000
ENSONIG00000007129
614
ENSPFOG00000005630
505
1000
ENSXMAG00000002456
406
ENSORLG00000003269
ENSGACG00000012317
1000
ENSTRUG00000003924
1000
ENSTNIG00000013384
ENSDARG00000021439
804
ENSAMXG00000015735
ENSLOCG00000012898

Figure S1

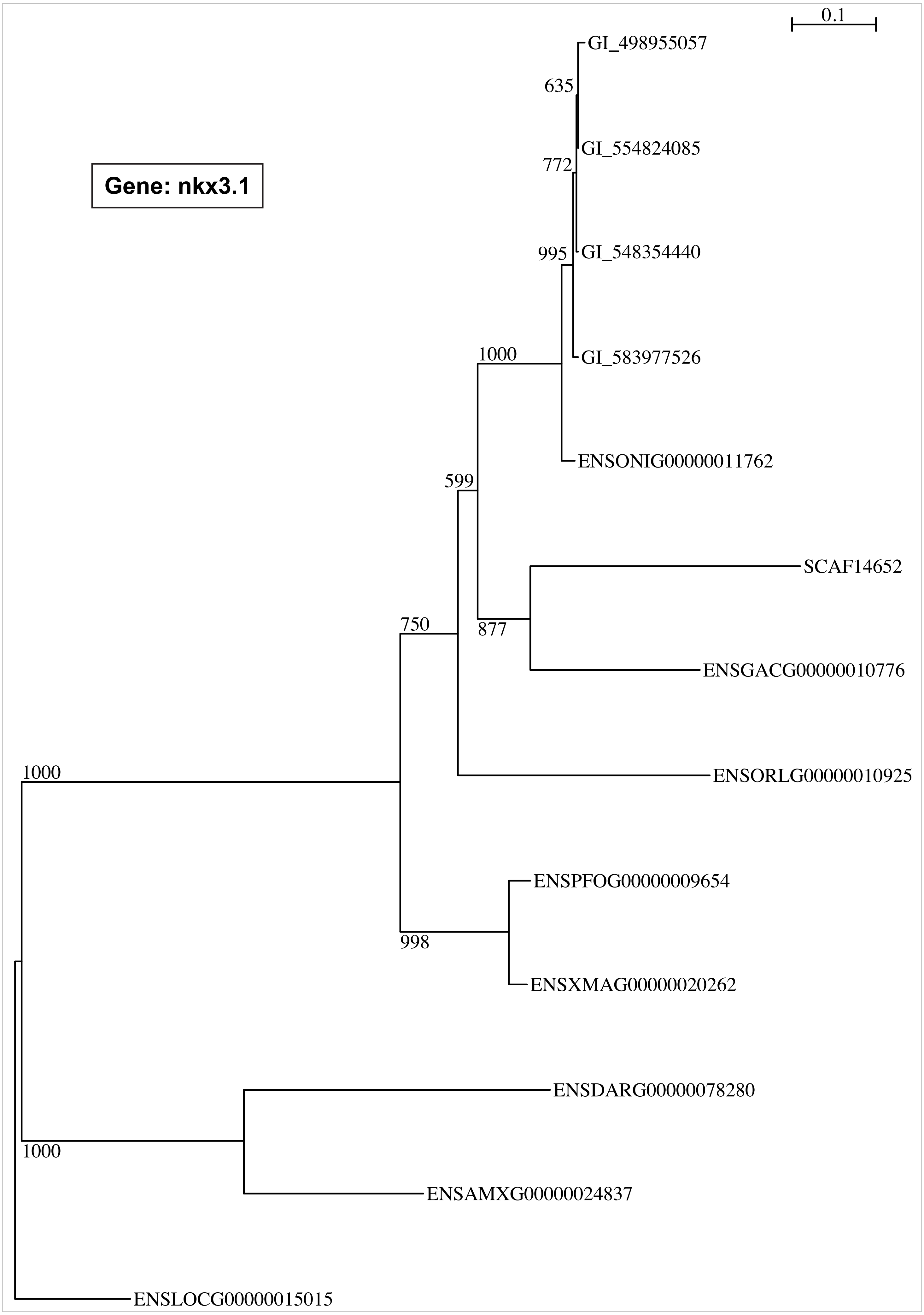
0.1
Gene: nkx3.1
GI_498955057
635
GI_554824085
772
GI_548354440
995
GI_583977526
1000
ENSONIG00000011762
599
SCAF14652
750
877
ENSGACG00000010776
ENSORLG00000010925
1000
ENSPFOG00000009654
998
ENSXMAG00000020262
ENSDARG00000078280
1000
ENSAMXG00000024837
ENSLOCG00000015015

Figure S1

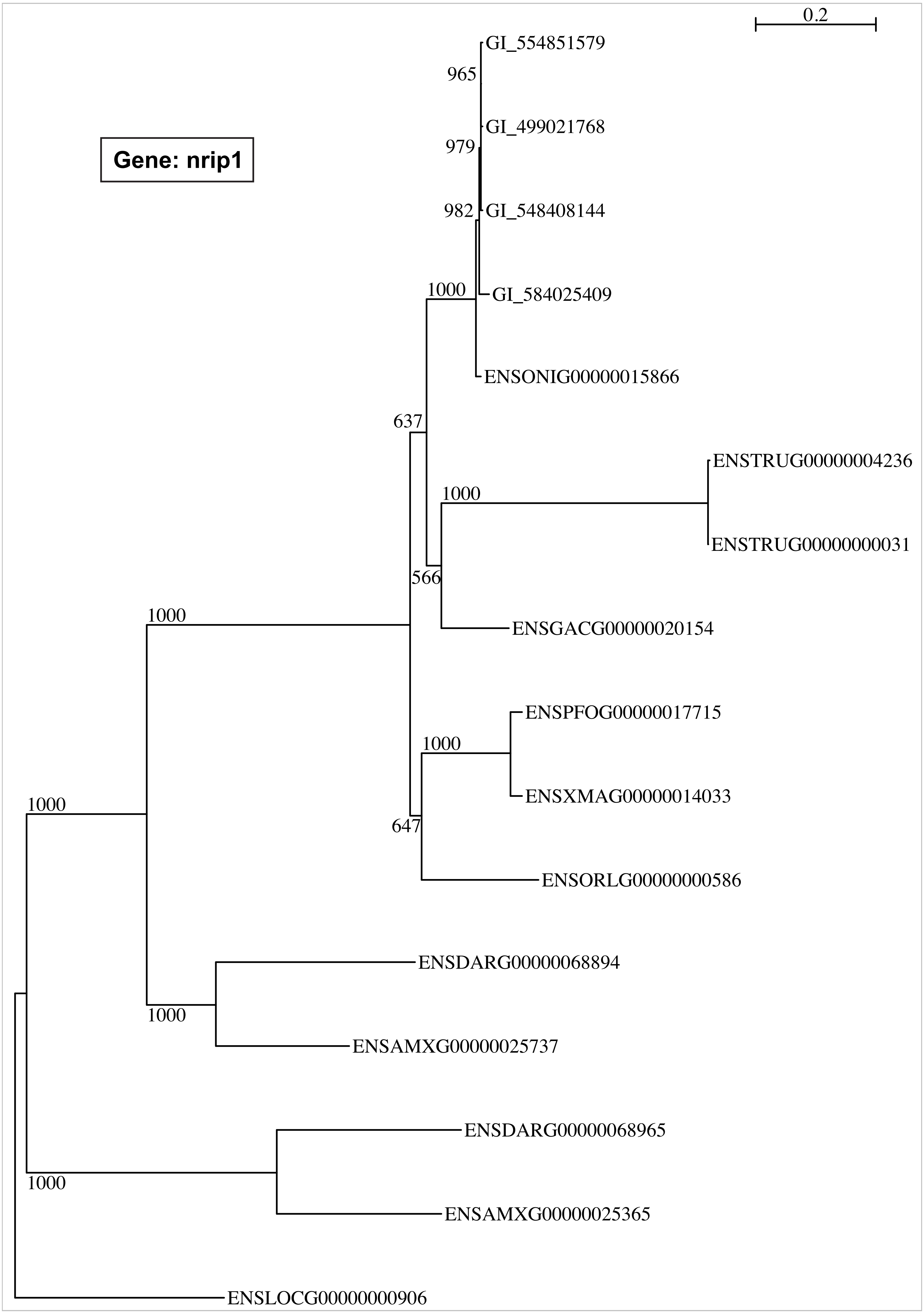

0.2
Gene: nrip1
GI_554851579
965
GI_499021768
979
982
GI_548408144
1000
GI_584025409
ENSONIG00000015866
637
ENSTRUG00000004236
1000
ENSTRUG00000000031
566
1000
ENSGACG00000020154
ENSPFOG00000017715
1000
ENSXMAG00000014033
1000
647
ENSORLG00000000586
ENSDARG00000068894
1000
ENSAMXG00000025737
ENSDARG00000068965
1000
ENSAMXG00000025365
ENSLOCG00000000906

Figure S1

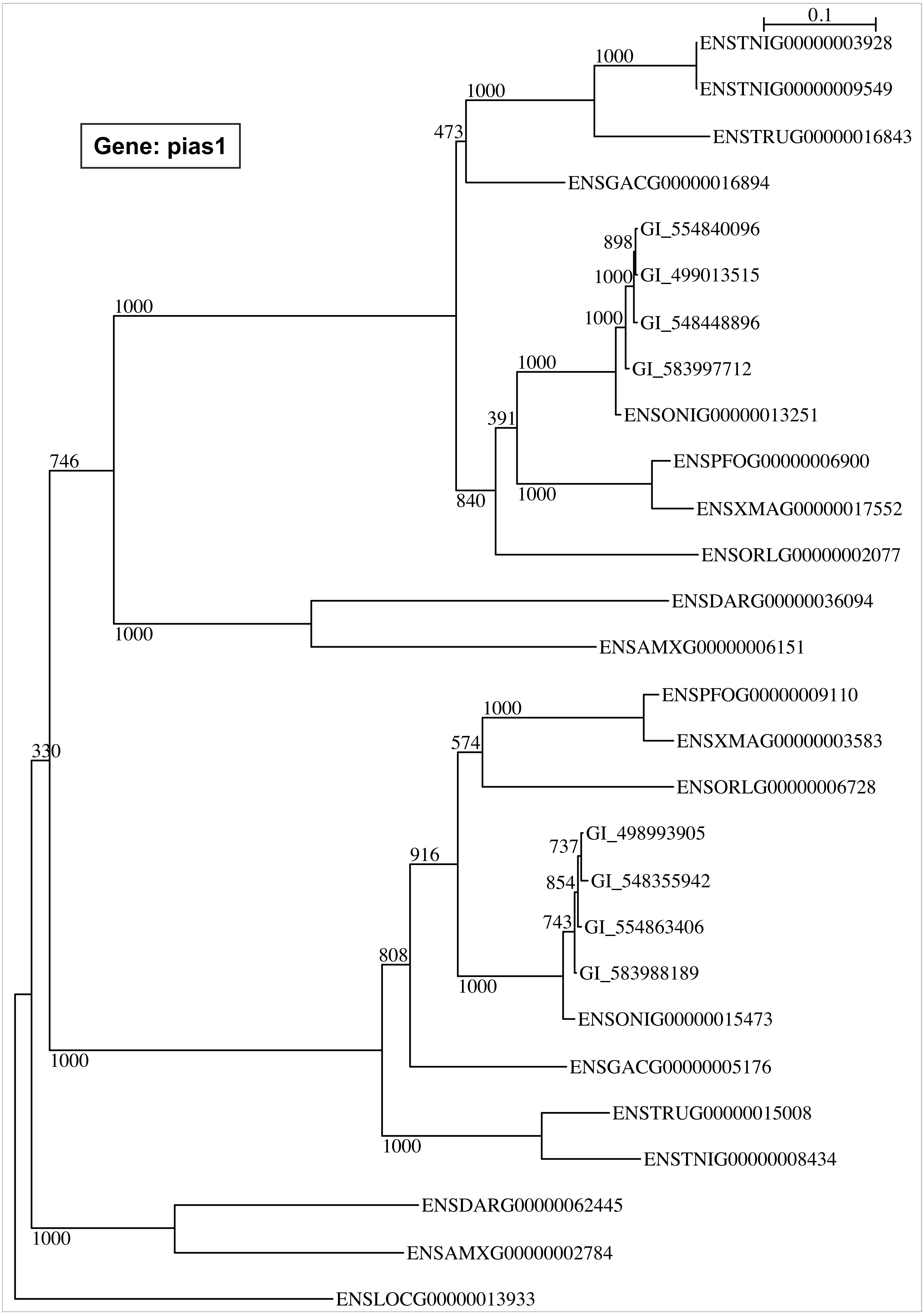

Gene: pias1
0.1
1000
1000
473
ENSTNIG00000003928
ENSTNIG00000009549
ENSTRUG00000016843
ENSGACG00000016894
898
1000
1000
GI_554840096
GI_499013515
GI_548448896
GI_583997712
1000
1000
391
ENSONIG00000013251
746
ENSPFOG00000006900
1000
840
ENSXMAG00000017552
ENSORLG00000002077
ENSDARG00000036094
1000
ENSAMXG00000006151
1000
ENSPFOG00000009110
574
ENSXMAG00000003583
330
ENSORLG00000006728
737
GI_498993905
916
854
GI_548355942
743
GI_554863406
808
GI_583988189
1000
ENSONIG00000015473
1000
ENSGACG00000005176
ENSTRUG00000015008
1000
ENSTNIG00000008434
ENSDARG00000062445
1000
ENSAMXG00000002784
ENSLOCG00000013933

Figure S1

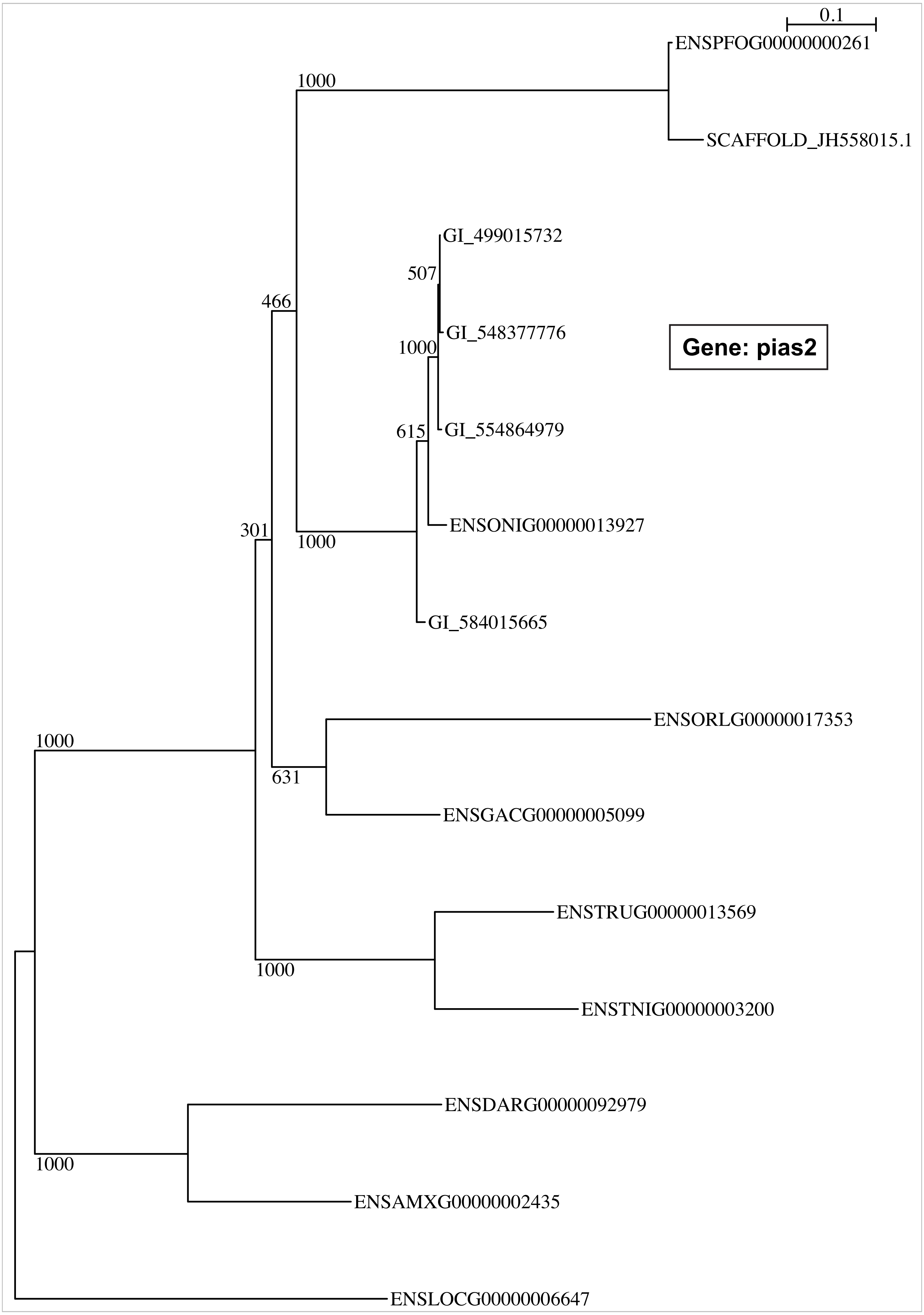
0.1
ENSPFOG00000000261
SCAFFOLD_JH558015.1
1000
GI_499015732
507
466
GI_548377776
1000
Gene: pias2
GI_554864979
615
ENSONIG00000013927
301
1000
GI_584015665
ENSORLG00000017353
1000
631
ENSGACG00000005099
ENSTRUG00000013569
1000
ENSTNIG00000003200
ENSDARG00000092979
1000
ENSAMXG00000002435
ENSLOCG00000006647

Figure S1

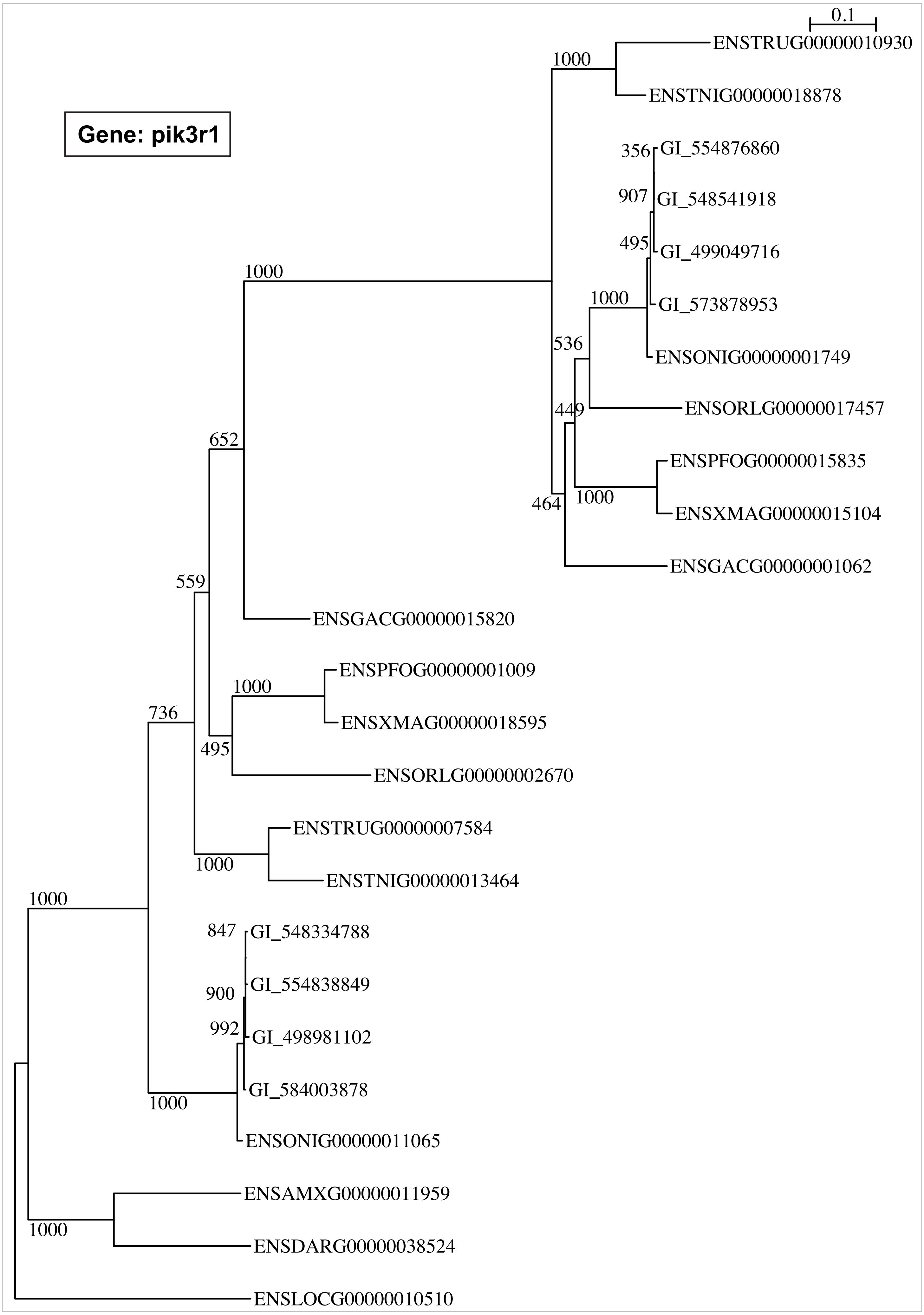

0.1
Gene: pik3r1
ENSTRUG00000010930
ENSTNIG00000018878
GI_554876860
GI_548541918
GI_499049716
GI_573878953
ENSONIG00000001749
ENSORLG00000017457
ENSPFOG00000015835
ENSXMAG00000015104
ENSGACG00000001062
ENSGACG00000015820
ENSPFOG00000001009
ENSXMAG00000018595
ENSORLG00000002670
ENSTRUG00000007584
ENSTNIG00000013464
GI_548334788
GI_554838849
GI_498981102
GI_584003878
ENSONIG00000011065
ENSAMXG00000011959
ENSDARG00000038524
ENSLOCG00000010510
1000
356
907
495
1000
1000
536
449
652
1000
464
559
1000
736
495
1000
1000
847
900
992
1000
1000

Figure S1

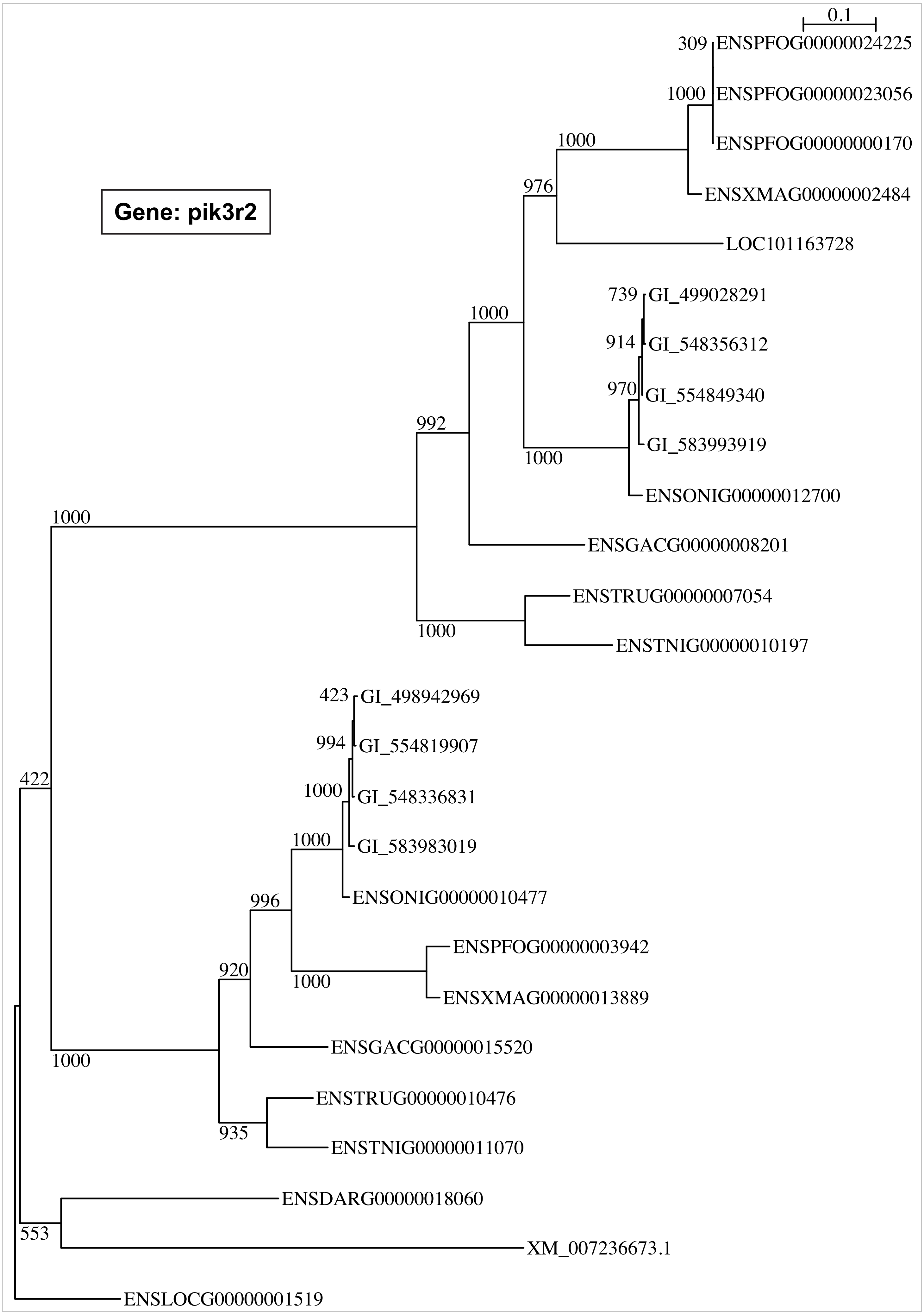
0.1
Gene: pik3r2
309
ENSPFOG00000024225
1000
ENSPFOG00000023056
1000
ENSPFOG00000000170
976
ENSXMAG00000002484
LOC101163728
739
GI_499028291
1000
914
GI_548356312
970
GI_554849340
992
GI_583993919
1000
ENSONIG00000012700
1000
ENSGACG00000008201
ENSTRUG00000007054
1000
ENSTNIG00000010197
423
GI_498942969
994
GI_554819907
422
1000
GI_548336831
1000
GI_583983019
ENSONIG00000010477
996
ENSPFOG00000003942
920
1000
ENSXMAG00000013889
ENSGACG00000015520
1000
ENSTRUG00000010476
935
ENSTNIG00000011070
ENSDARG00000018060
553
XM_007236673.1
ENSLOCG00000001519

Figure S1

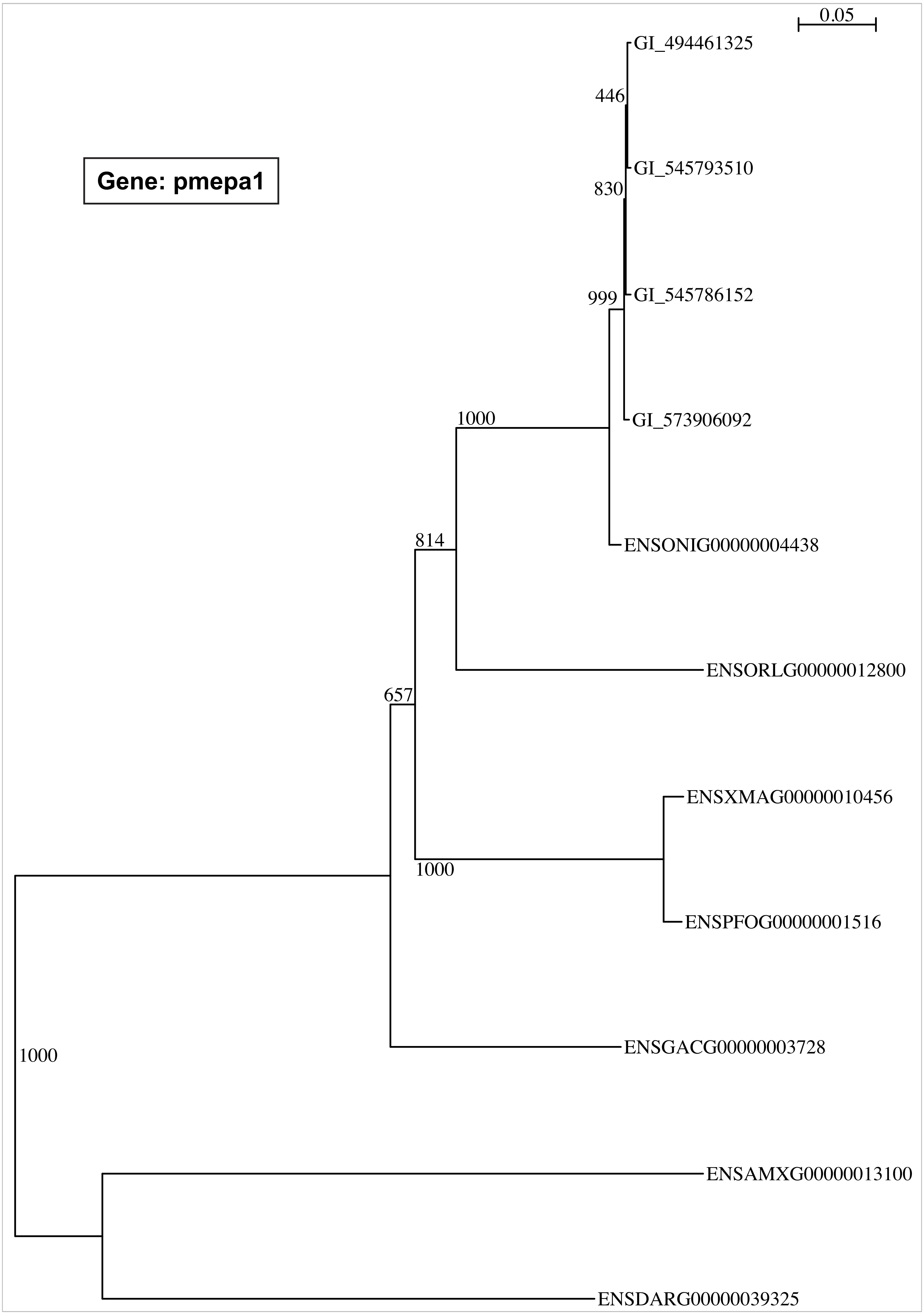

Gene: pmepa1
0.05
GI_494461325
446
GI_545793510
830
GI_545786152
999
GI_573906092
1000
ENSONIG00000004438
814
ENSORLG00000012800
657
ENSXMAG00000010456
1000
ENSPFOG00000001516
ENSGACG00000003728
1000
ENSAMXG00000013100
ENSDARG00000039325

Figure S1

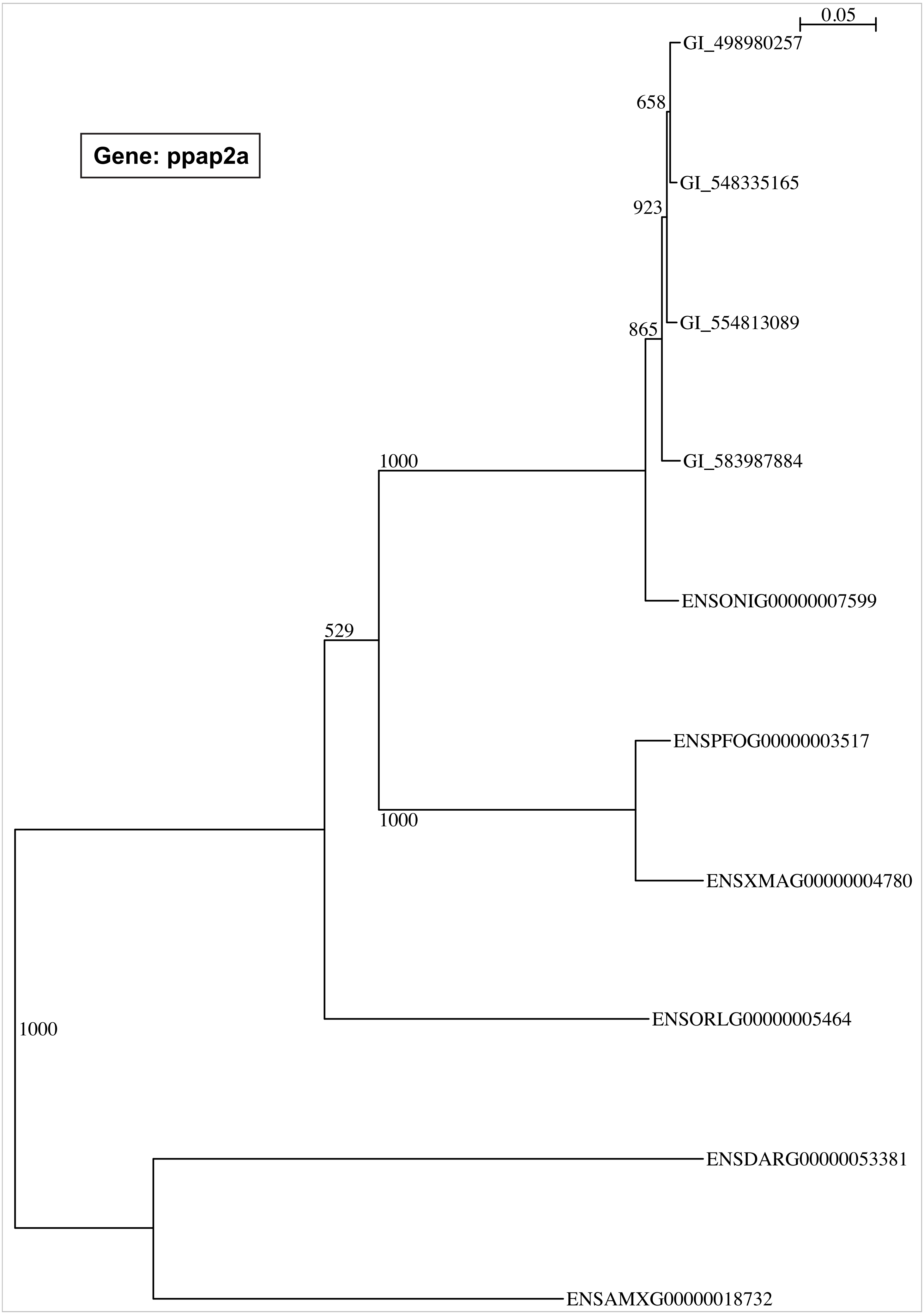
0.05
Gene: ppap2a
GI_498980257
658
GI_548335165
923
GI_554813089
865
1000
GI_583987884
ENSONIG00000007599
529
ENSPFOG00000003517
1000
ENSXMAG00000004780
ENSORLG00000005464
1000
ENSDARG00000053381
ENSAMXG00000018732

Figure S1

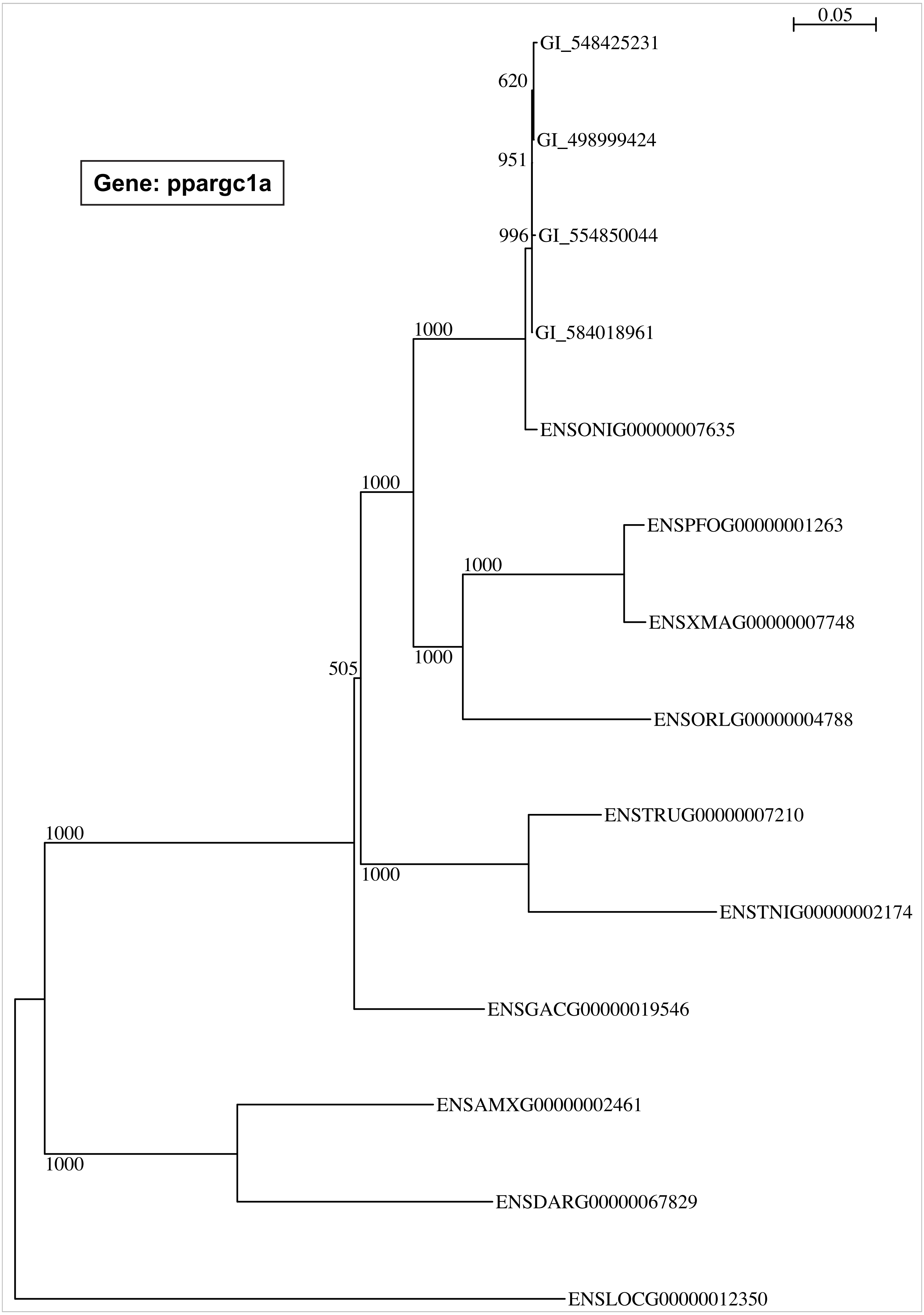
0.05
Gene: ppargc1a
GI_548425231
620
GI_498999424
951
996
GI_554850044
GI_584018961
1000
ENSONIG00000007635
1000
ENSPFOG00000001263
1000
ENSXMAG00000007748
1000
505
ENSORLG00000004788
ENSTRUG00000007210
1000
1000
ENSTNIG00000002174
ENSGACG00000019546
ENSAMXG00000002461
1000
ENSDARG00000067829
ENSLOCG00000012350

Figure S1

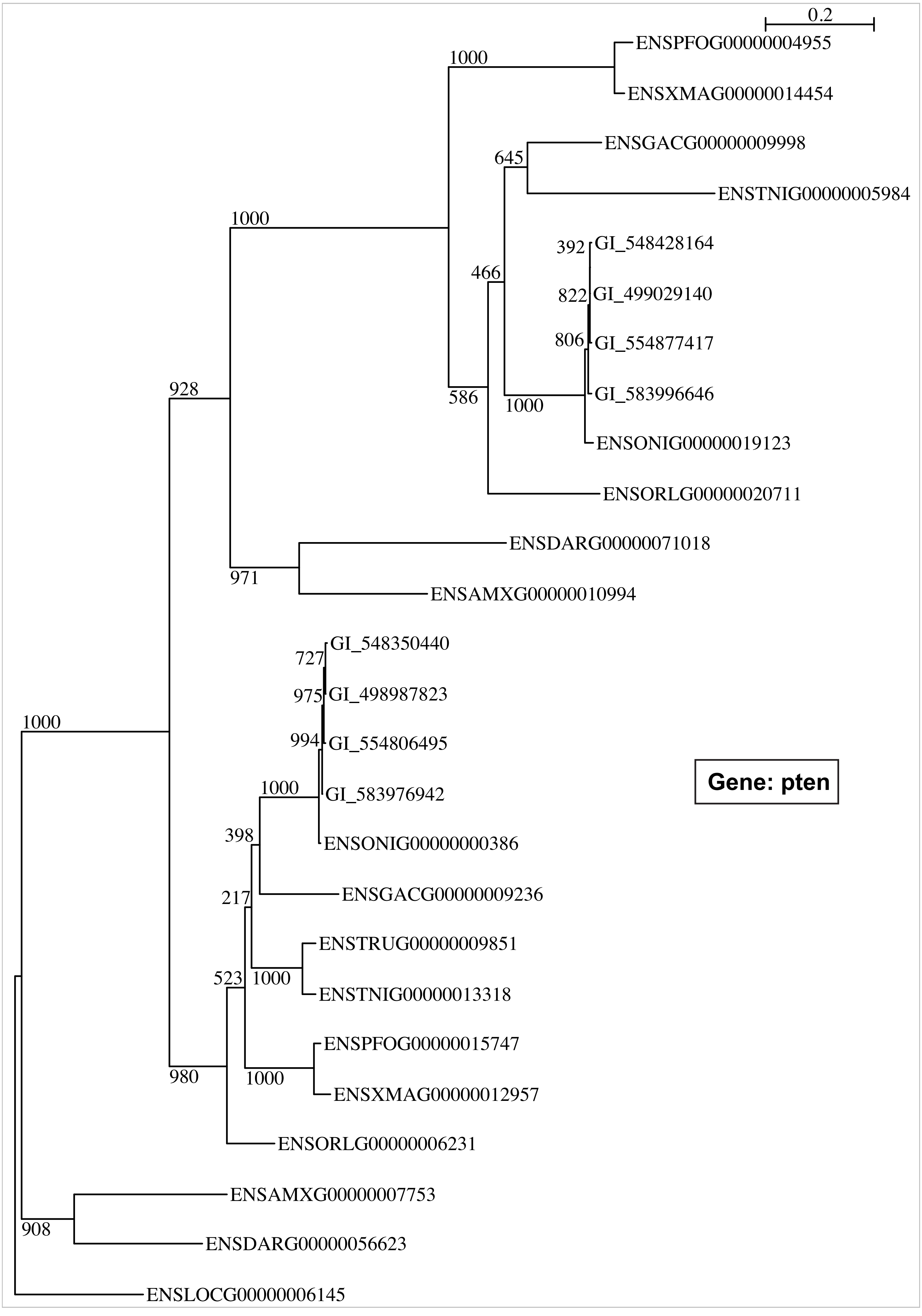
0.2
1000
ENSPFOG00000004955
ENSXMAG00000014454
ENSGACG00000009998
645
ENSTNIG00000005984
1000
392
GI_548428164
466
822
GI_499029140
806
GI_554877417
928
GI_583996646
586
1000
ENSONIG00000019123
ENSORLG00000020711
ENSDARG00000071018
971
ENSAMXG00000010994
GI_548350440
727
975
GI_498987823
1000
994
GI_554806495
Gene: pten
1000
GI_583976942
398
ENSONIG00000000386
217
ENSGACG00000009236
ENSTRUG00000009851
523
1000
ENSTNIG00000013318
ENSPFOG00000015747
980
1000
ENSXMAG00000012957
ENSORLG00000006231
ENSAMXG00000007753
908
ENSDARG00000056623
ENSLOCG00000006145

Figure S1

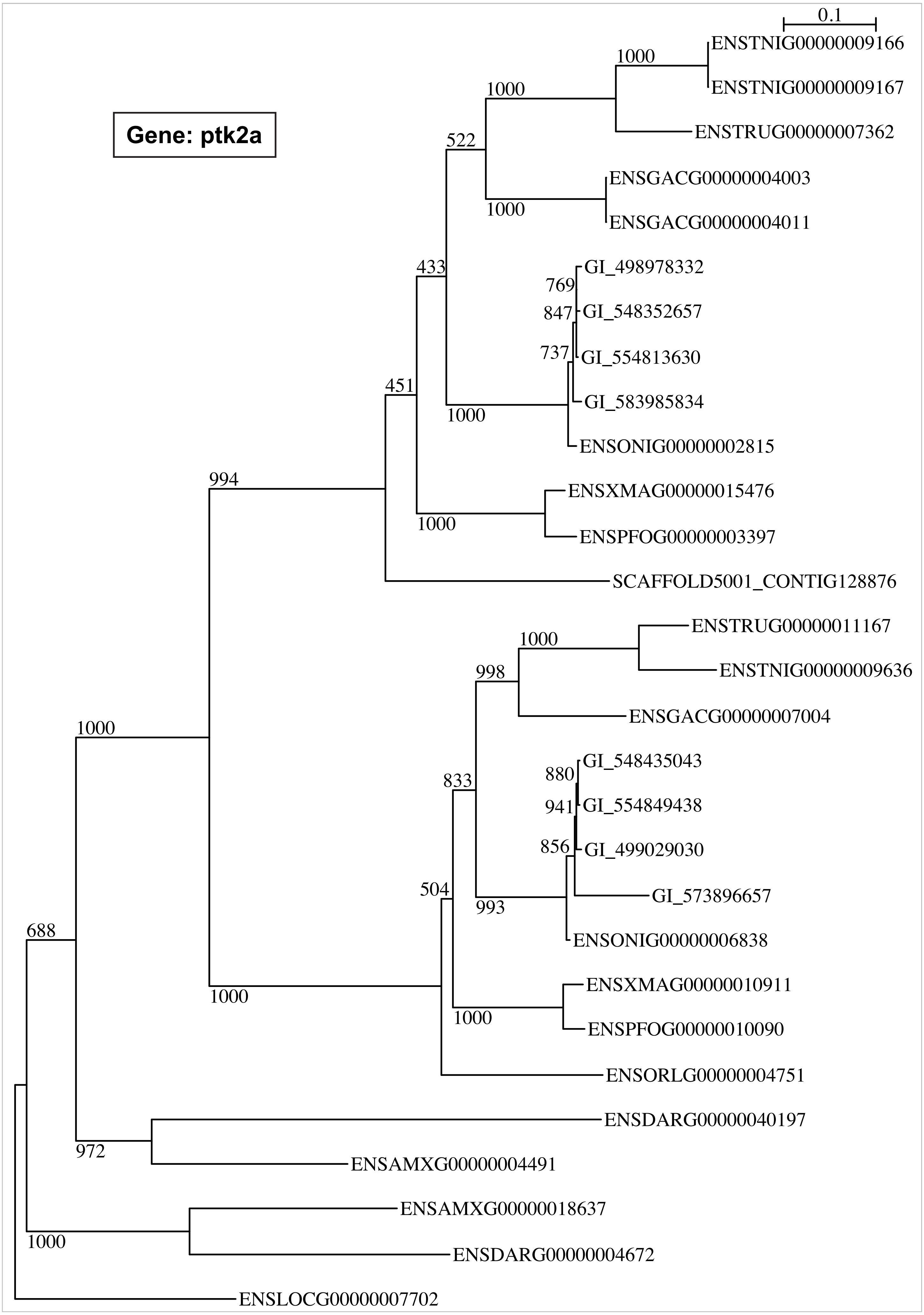

0.1
Gene: ptk2a
1000
ENSTNIG00000009166
ENSTNIG00000009167
1000
ENSTRUG00000007362
522
ENSGACG00000004003
1000
ENSGACG00000004011
433
GI_498978332
769
847
GI_548352657
737
GI_554813630
451
GI_583985834
1000
ENSONIG00000002815
994
ENSXMAG00000015476
1000
ENSPFOG00000003397
SCAFFOLD5001_CONTIG128876
ENSTRUG00000011167
1000
ENSTNIG00000009636
998
ENSGACG00000007004
1000
GI_548435043
880
833
941
GI_554849438
856
GI_499029030
504
GI_573896657
993
688
ENSONIG00000006838
1000
ENSXMAG00000010911
1000
ENSPFOG00000010090
ENSORLG00000004751
ENSDARG00000040197
972
ENSAMXG00000004491
ENSAMXG00000018637
1000
ENSDARG00000004672
ENSLOCG00000007702

Figure S1

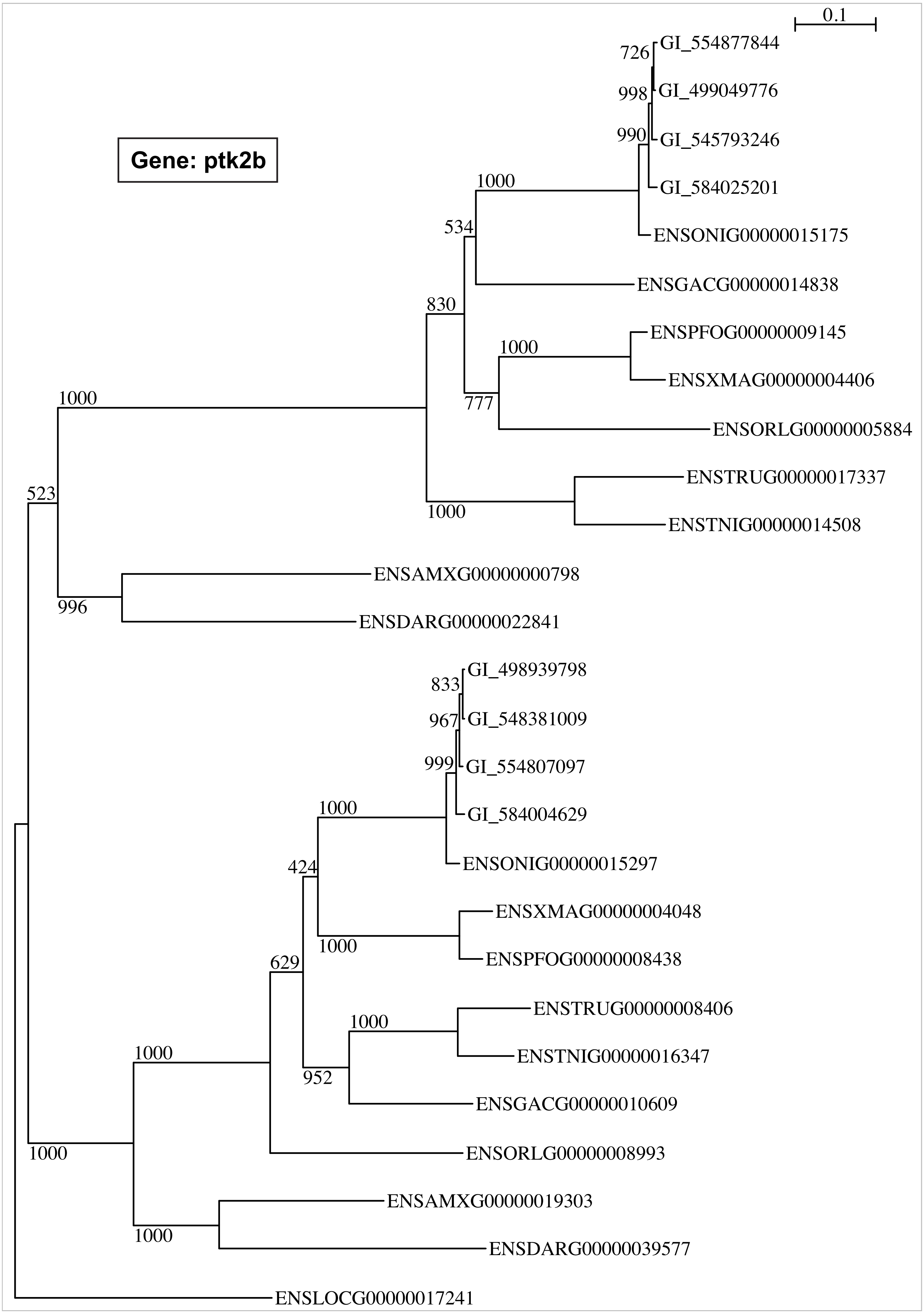

0.1
Gene: ptk2b
726
GI_554877844
998
GI_499049776
990
GI_545793246
GI_584025201
1000
ENSONIG00000015175
534
ENSGACG00000014838
830
ENSPFOG00000009145
1000
ENSXMAG00000004406
1000
777
ENSORLG00000005884
ENSTRUG00000017337
523
1000
ENSTNIG00000014508
ENSAMXG00000000798
996
ENSDARG00000022841
GI_498939798
833
967
GI_548381009
999
GI_554807097
1000
GI_584004629
ENSONIG00000015297
424
ENSXMAG00000004048
1000
629
ENSPFOG00000008438
ENSTRUG00000008406
1000
ENSTNIG00000016347
1000
952
ENSGACG00000010609
1000
ENSORLG00000008993
ENSAMXG00000019303
1000
ENSDARG00000039577
ENSLOCG00000017241

Figure S1

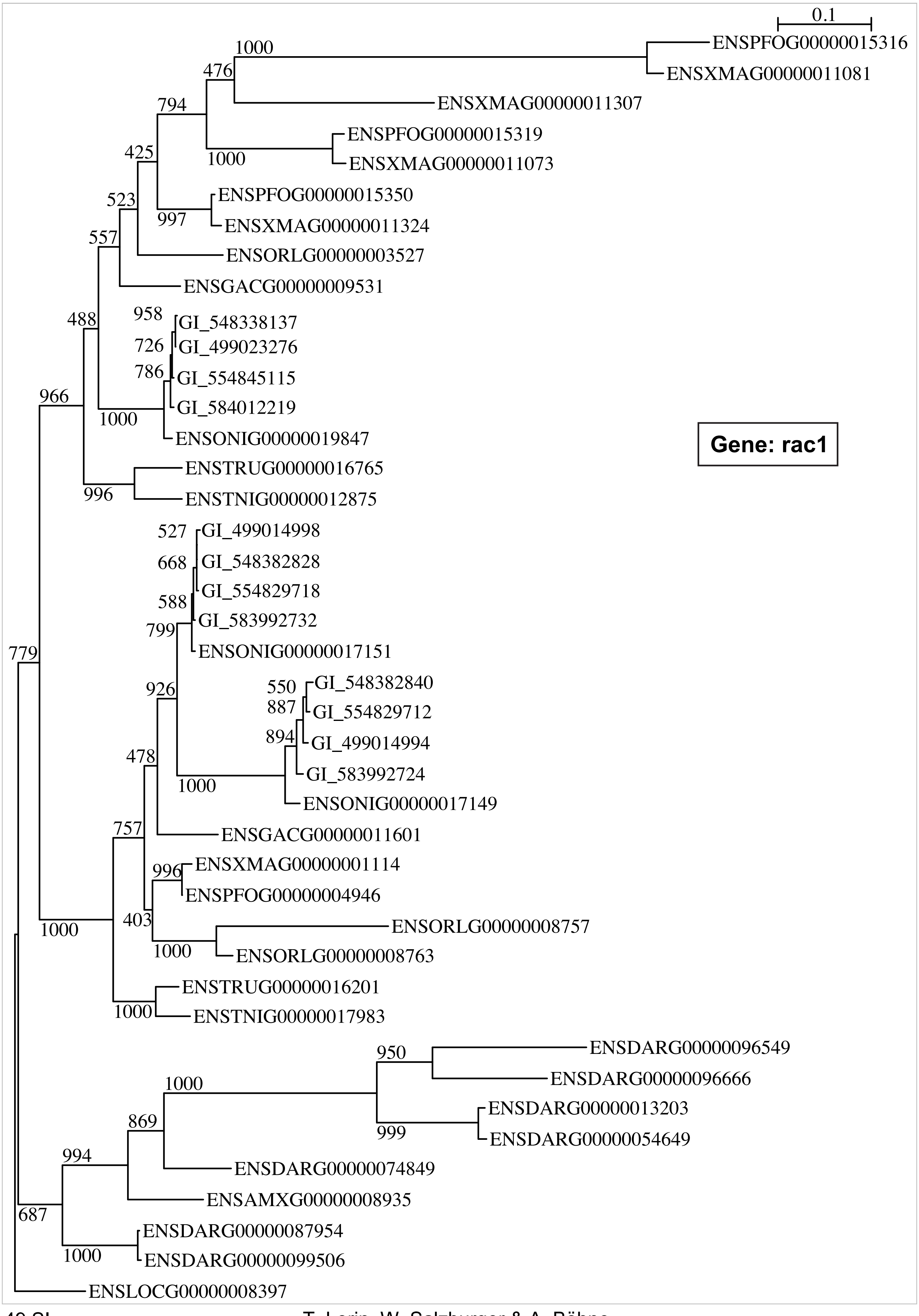

 T. Lorin, W. Salzburger & A. Böhne

Figure S1

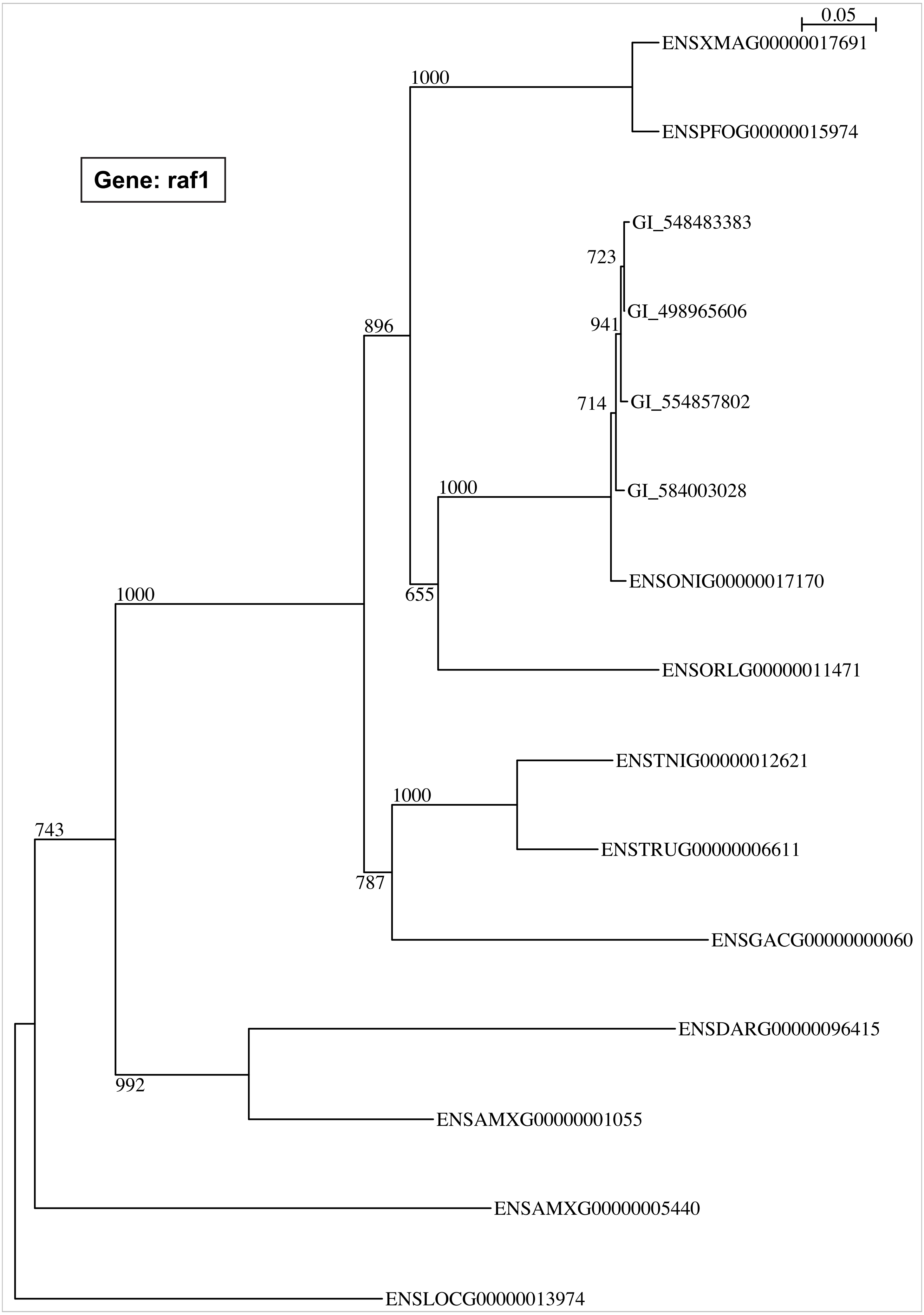
0.05
Gene: raf1
ENSXMAG00000017691
ENSPFOG00000015974
GI_548483383
GI_498965606
GI_554857802
GI_584003028
ENSONIG00000017170
ENSORLG00000011471
ENSTNIG00000012621
ENSTRUG00000006611
ENSGACG00000000060
ENSDARG00000096415
ENSAMXG00000001055
ENSAMXG00000005440
ENSLOCG00000013974
1000
723
941
896
714
1000
1000
655
1000
743
787
992

Figure S1

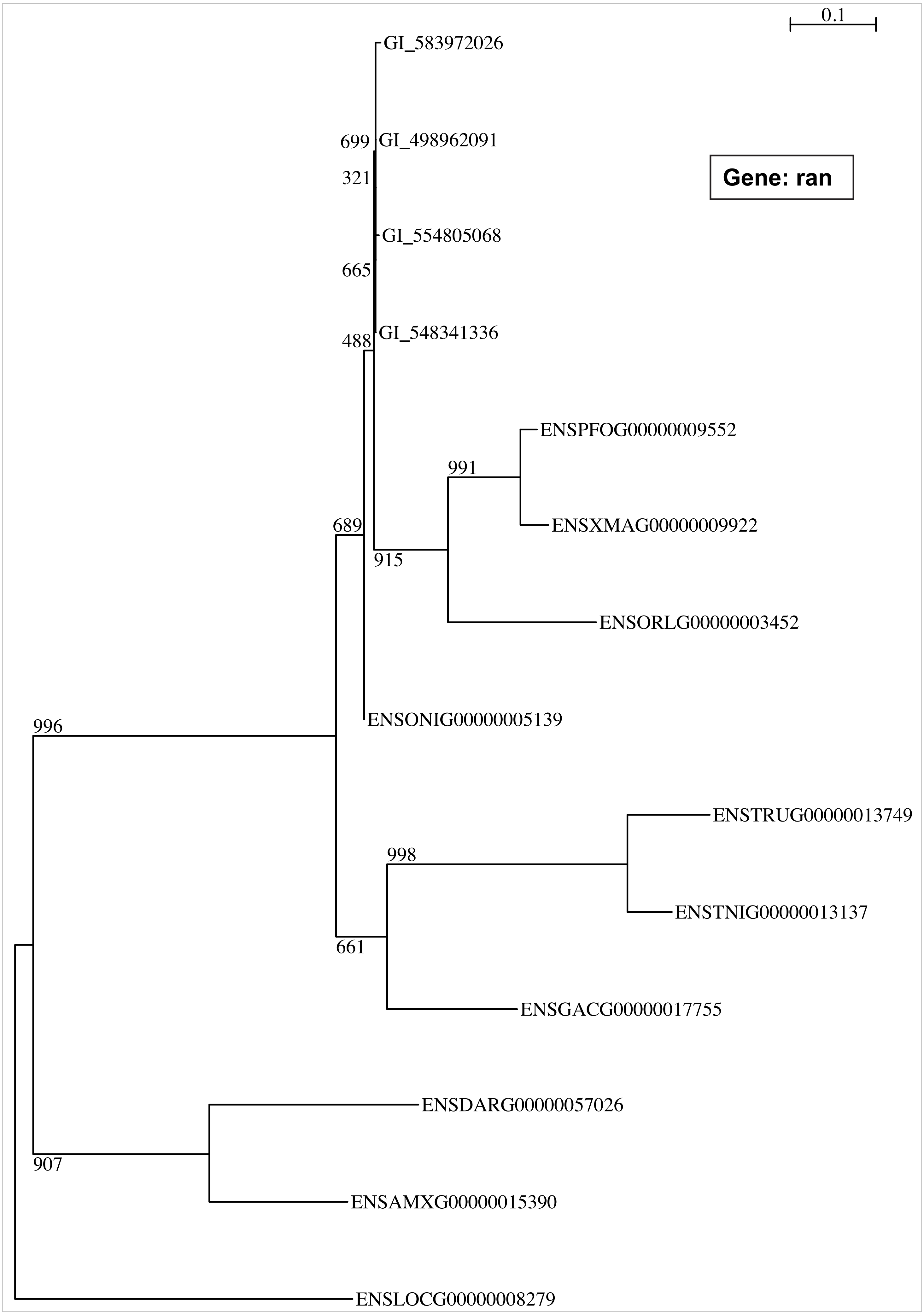
0.1
Gene: ran
GI_583972026
699
GI_498962091
321
GI_554805068
665
GI_548341336
488
ENSPFOG00000009552
991
ENSXMAG00000009922
689
915
ENSORLG00000003452
ENSONIG00000005139
996
ENSTRUG00000013749
998
ENSTNIG00000013137
661
ENSGACG00000017755
ENSDARG00000057026
907
ENSAMXG00000015390
ENSLOCG00000008279

Figure S1

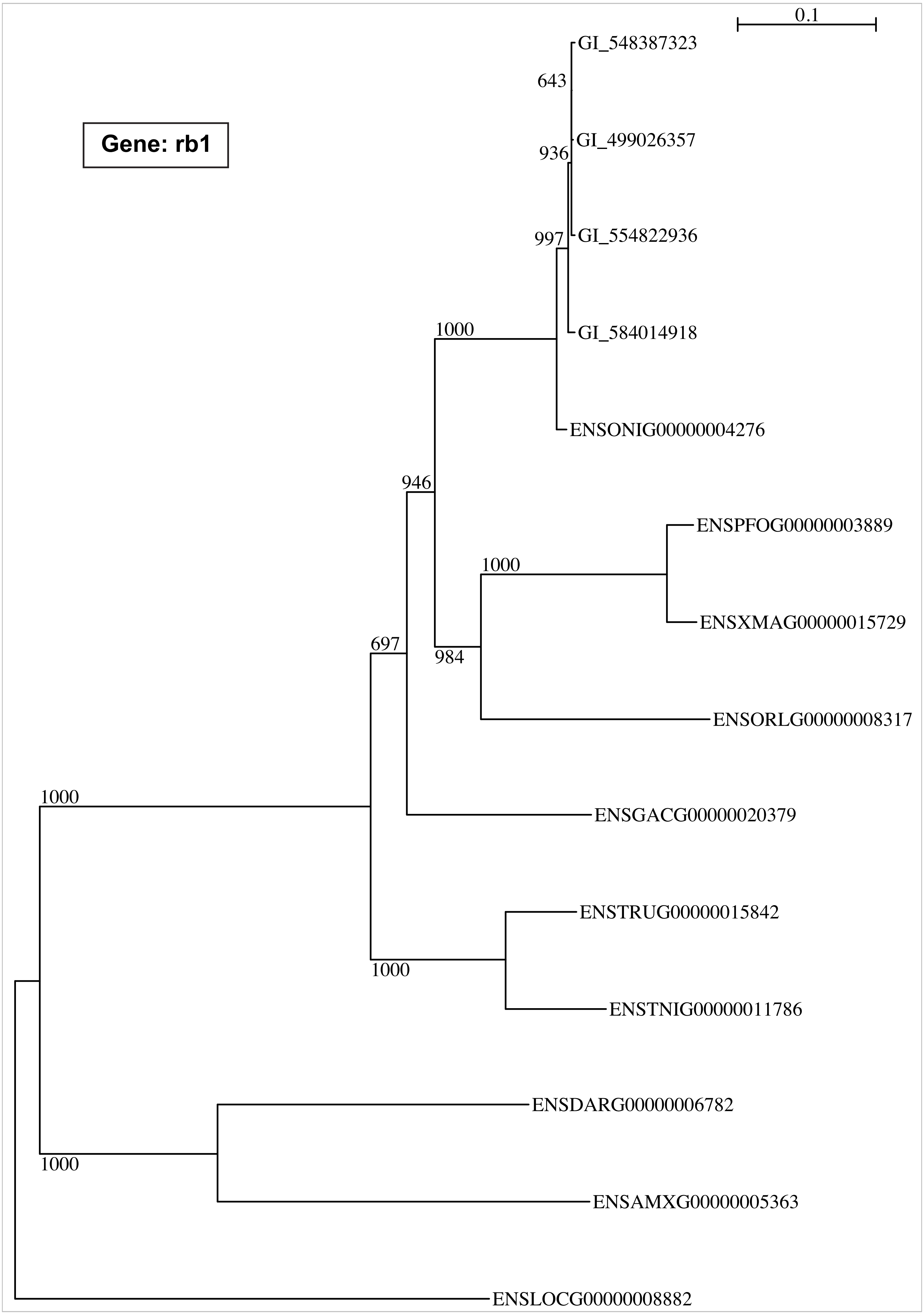

0.1
Gene: rb1
GI_548387323
643
GI_499026357
936
GI_554822936
997
GI_584014918
1000
ENSONIG00000004276
946
ENSPFOG00000003889
1000
ENSXMAG00000015729
697
984
ENSORLG00000008317
1000
ENSGACG00000020379
ENSTRUG00000015842
1000
ENSTNIG00000011786
ENSDARG00000006782
1000
ENSAMXG00000005363
ENSLOCG00000008882

Figure S1

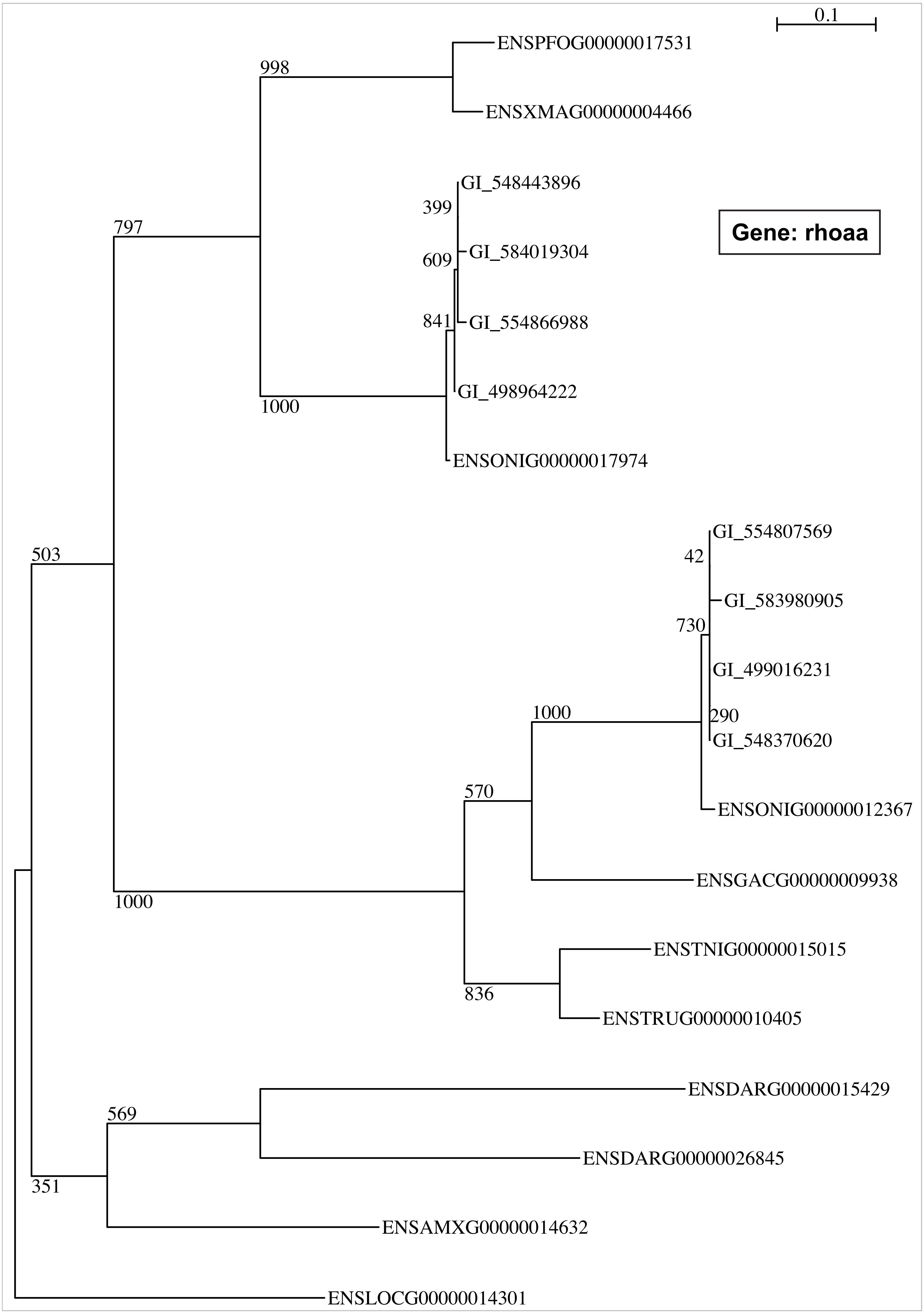
0.1
Gene: rhoaa
ENSPFOG00000017531
ENSXMAG00000004466
998
GI_548443896
399
GI_584019304
609
GI_554866988
841
GI_498964222
1000
ENSONIG00000017974
797
503
GI_554807569
42
GI_583980905
730
GI_499016231
290
GI_548370620
1000
ENSONIG00000012367
570
ENSGACG00000009938
1000
ENSTNIG00000015015
836
ENSTRUG00000010405
ENSDARG00000015429
569
ENSDARG00000026845
351
ENSAMXG00000014632
ENSLOCG00000014301

Figure S1

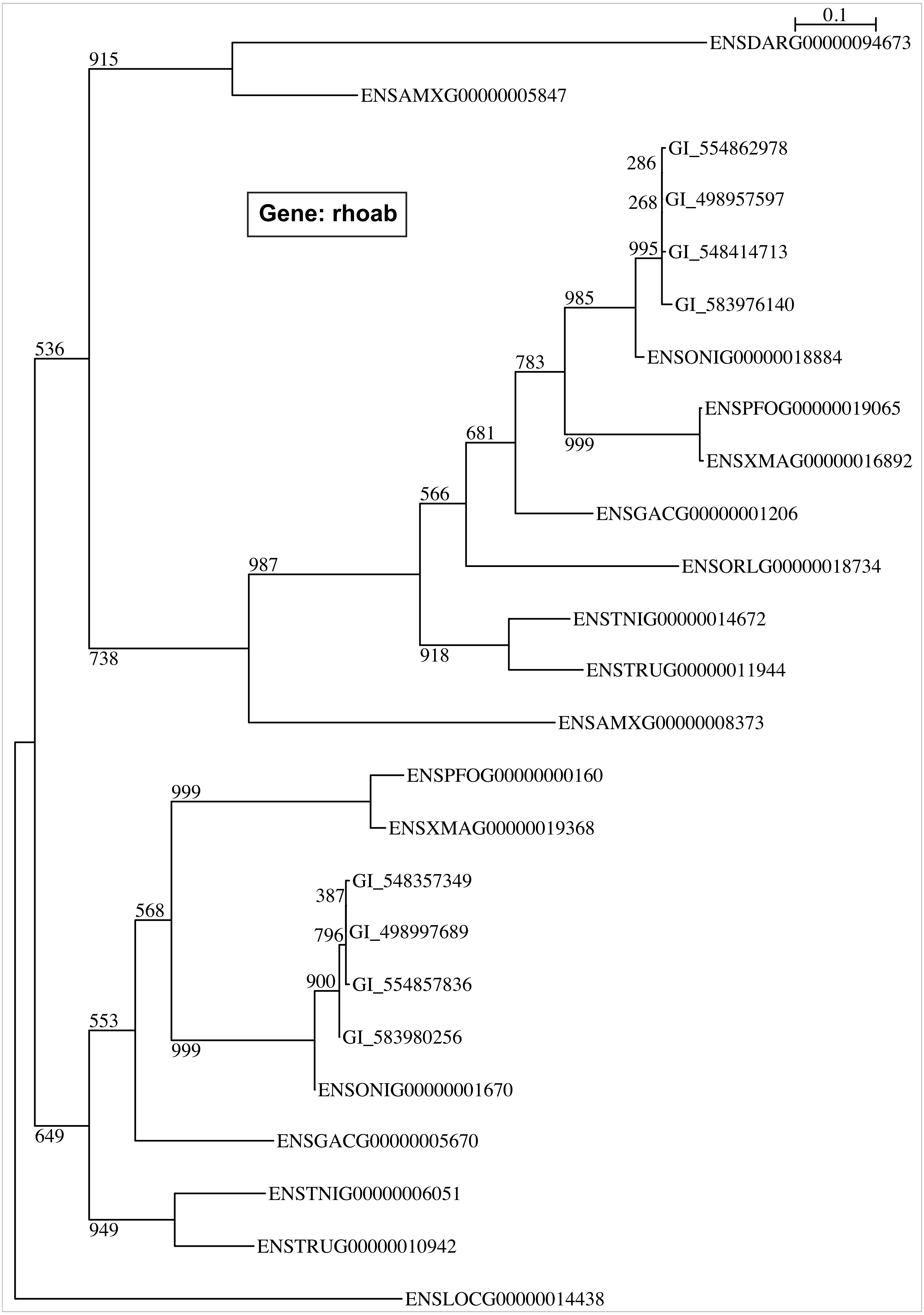
0.1
Gene: rhoab
ENSDARG00000094673
ENSAMXG00000005847
GI_554862978
GI_498957597
GI_548414713
GI_583976140
ENSONIG00000018884
ENSPFOG00000019065
ENSXMAG00000016892
ENSGACG00000001206
ENSORLG00000018734
ENSTNIG00000014672
ENSTRUG00000011944
ENSAMXG00000008373
ENSPFOG00000000160
ENSXMAG00000019368
GI_548357349
GI_498997689
GI_554857836
GI_583980256
ENSONIG00000001670
ENSGACG00000005670
ENSTNIG00000006051
ENSTRUG00000010942
ENSLOCG00000014438
915
536
286
268
995
985
783
999
681
566
987
918
738
999
387
796
900
568
553
999
649
949

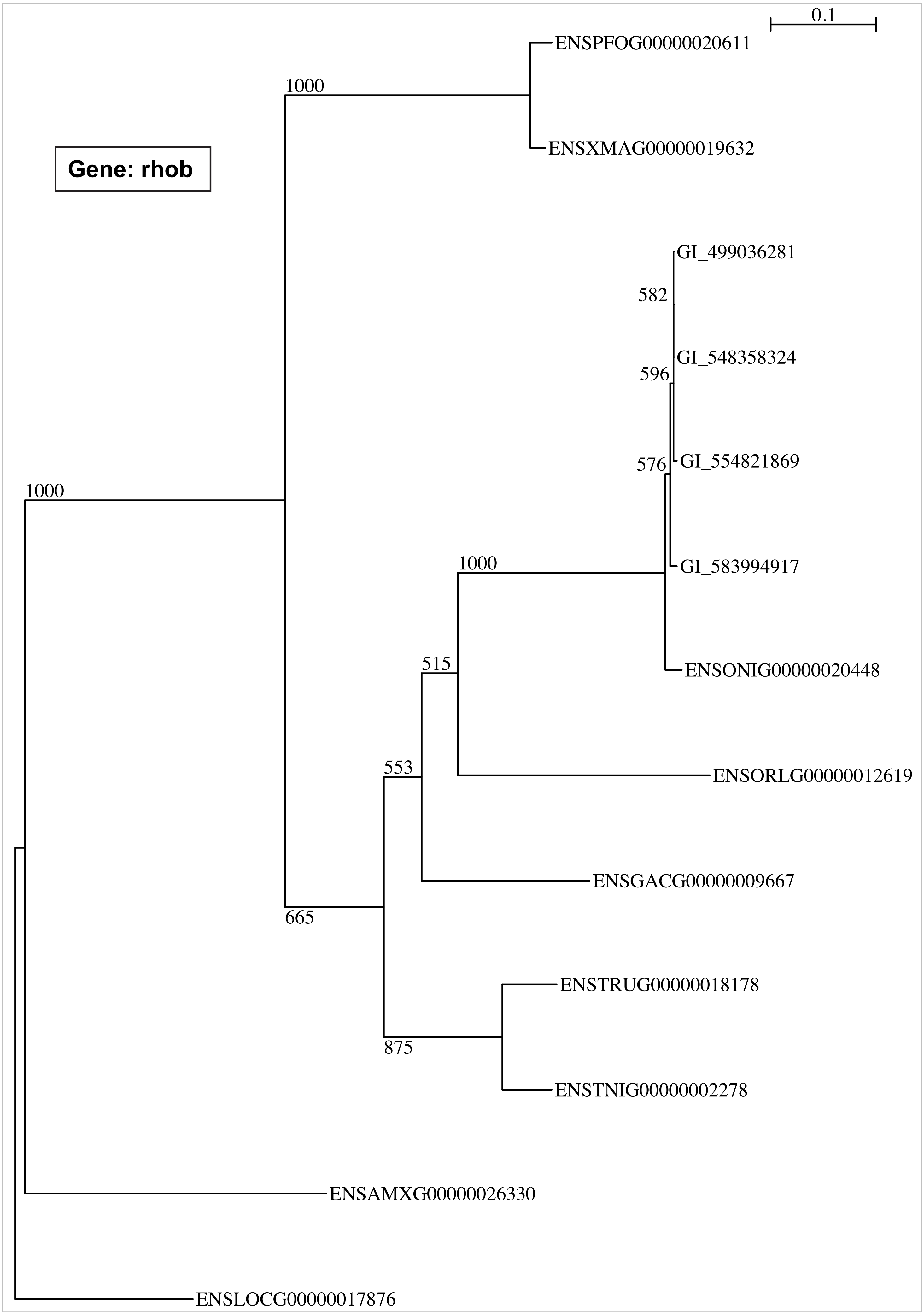
0.1
ENSPFOG00000020611
1000
ENSXMAG00000019632
Gene: rhob
GI_499036281
582
GI_548358324
596
GI_554821869
576
1000
GI_583994917
1000
ENSONIG00000020448
515
553
ENSORLG00000012619
ENSGACG00000009667
665
ENSTRUG00000018178
875
ENSTNIG00000002278
ENSAMXG00000026330
ENSLOCG00000017876

Figure S1

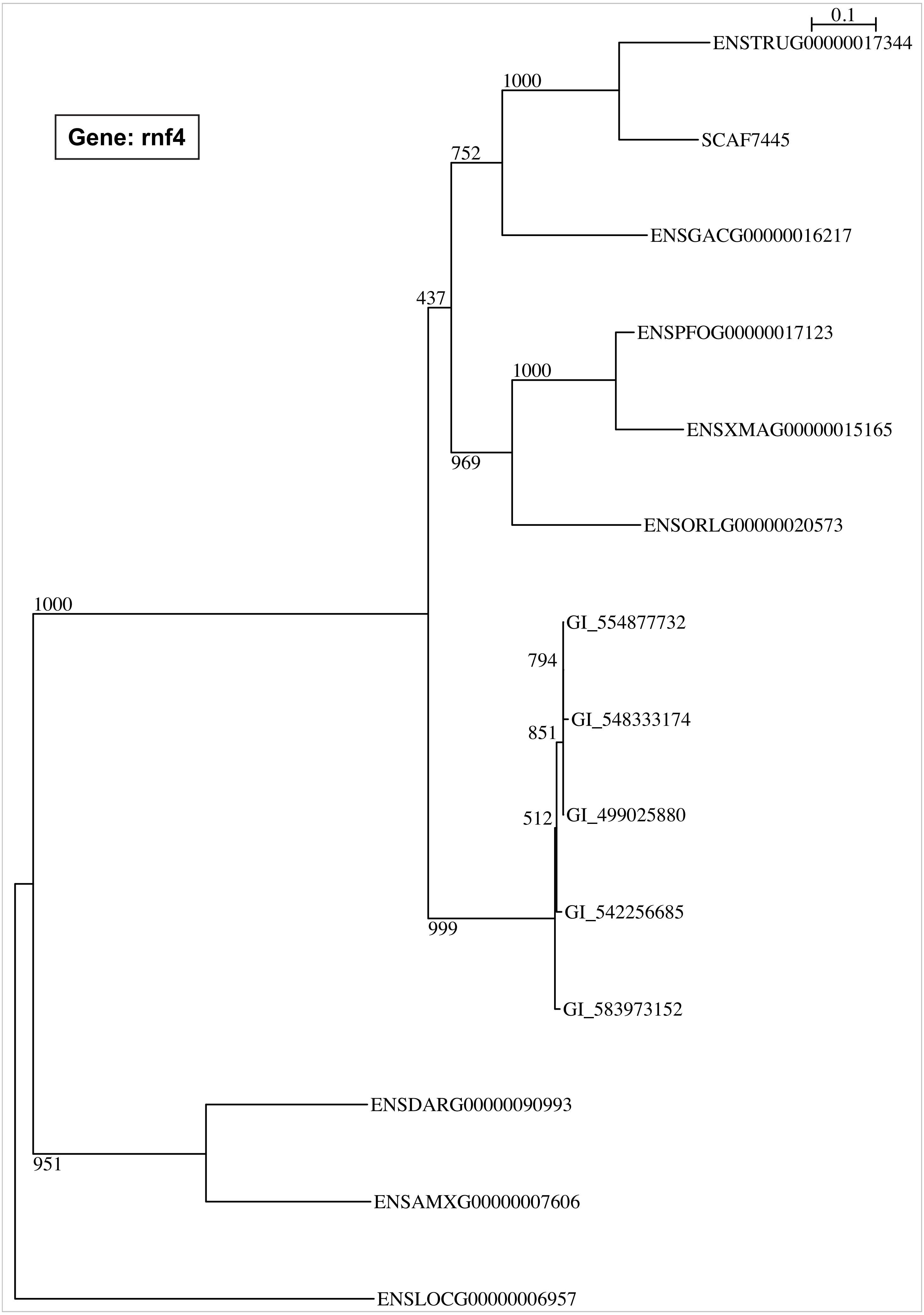
0.1
Gene: rnf4
ENSTRUG00000017344
SCAF7445
ENSGACG00000016217
ENSPFOG00000017123
ENSXMAG00000015165
ENSORLG00000020573
GI_554877732
GI_548333174
GI_499025880
GI_542256685
GI_583973152
ENSDARG00000090993
ENSAMXG00000007606
ENSLOCG00000006957
1000
752
437
1000
969
1000
794
851
512
999
951

Figure S1

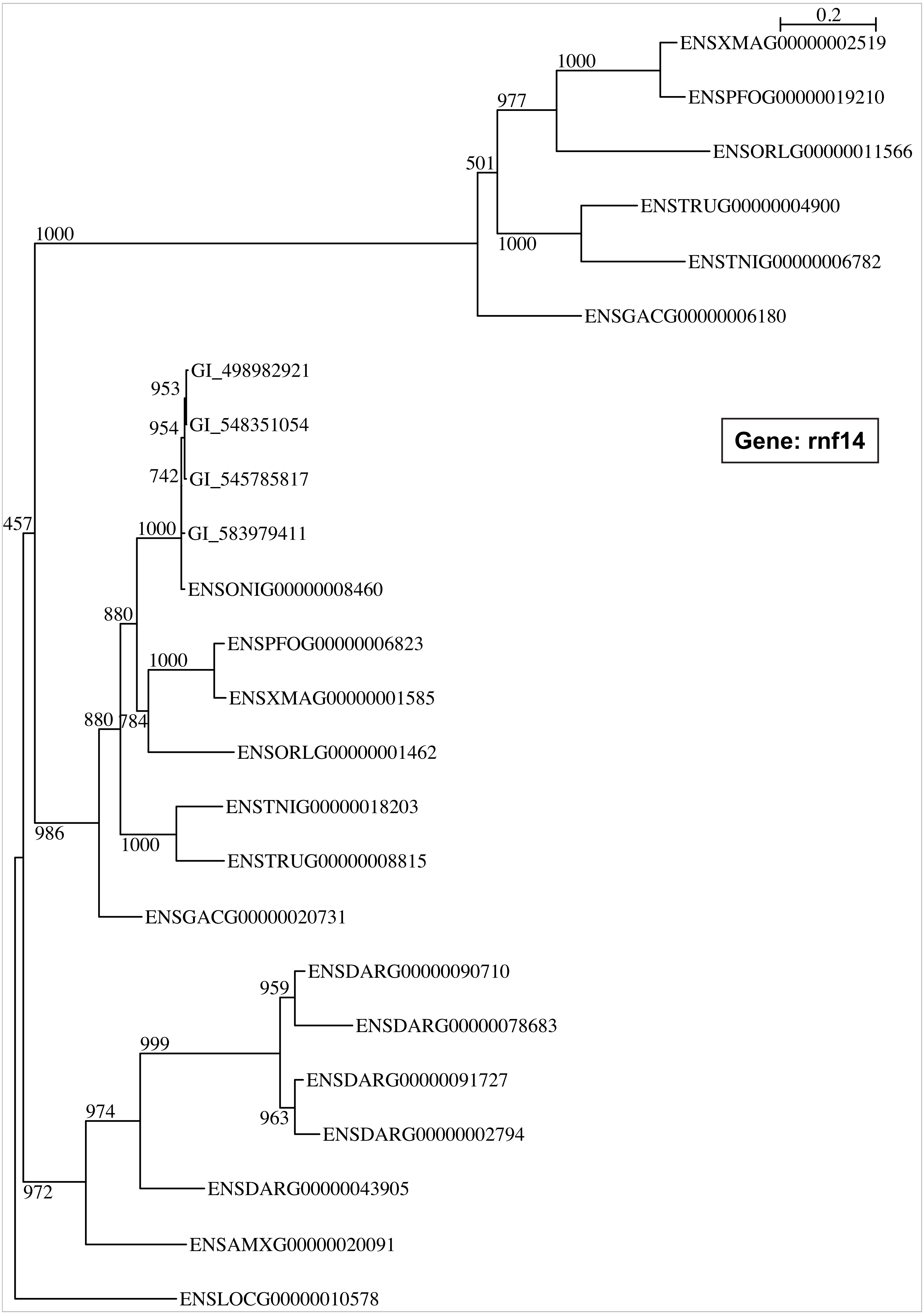
0.2
Gene: rnf14
1000
ENSXMAG00000002519
977
ENSPFOG00000019210
ENSORLG00000011566
501
ENSTRUG00000004900
1000
1000
ENSTNIG00000006782
ENSGACG00000006180
GI_498982921
953
GI_548351054
954
742
GI_545785817
457
1000
GI_583979411
ENSONIG00000008460
880
ENSPFOG00000006823
1000
ENSXMAG00000001585
880
784
ENSORLG00000001462
ENSTNIG00000018203
986
1000
ENSTRUG00000008815
ENSGACG00000020731
ENSDARG00000090710
959
ENSDARG00000078683
999
ENSDARG00000091727
974
963
ENSDARG00000002794
ENSDARG00000043905
972
ENSAMXG00000020091
ENSLOCG00000010578

Figure S1

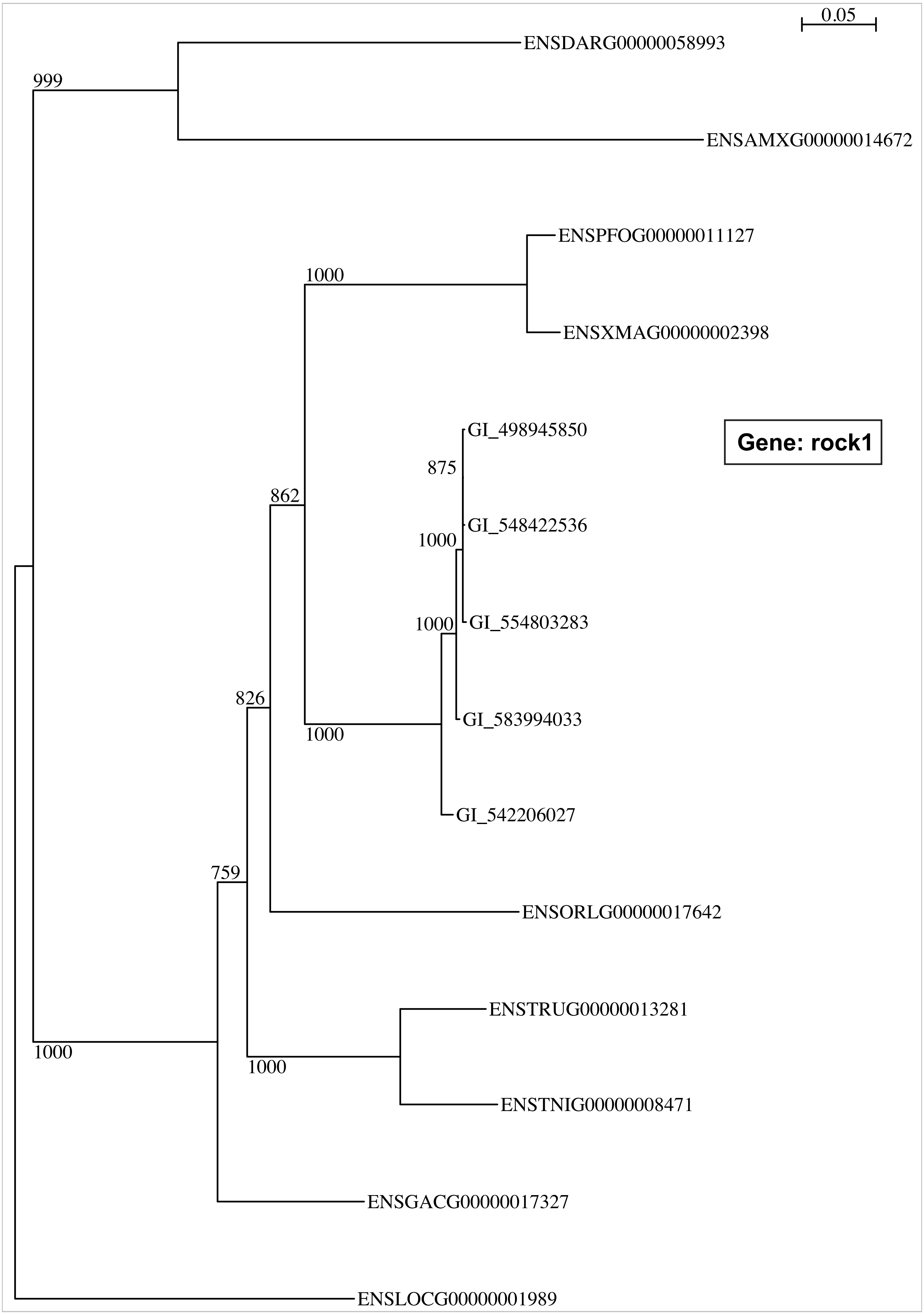
0.05
ENSDARG00000058993
999
ENSAMXG00000014672
ENSPFOG00000011127
1000
ENSXMAG00000002398
GI_498945850
Gene: rock1
875
862
GI_548422536
1000
GI_554803283
1000
826
GI_583994033
1000
GI_542206027
759
ENSORLG00000017642
ENSTRUG00000013281
1000
1000
ENSTNIG00000008471
ENSGACG00000017327
ENSLOCG00000001989

Figure S1

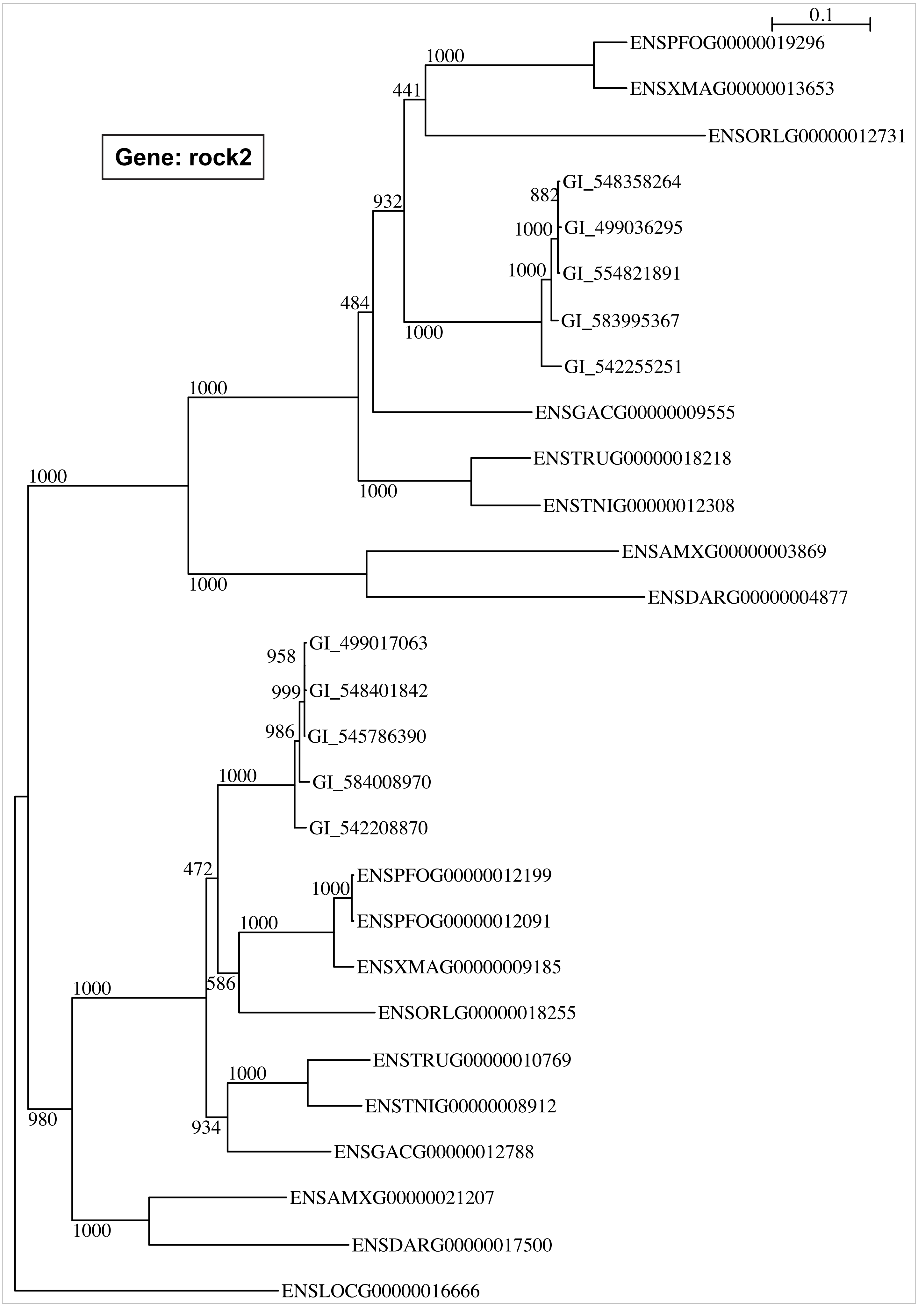
0.1
Gene: rock2
1000
441
932
484
1000
1000
882
1000
1000
1000
1000
1000
ENSPFOG00000019296
ENSXMAG00000013653
ENSORLG00000012731
GI_548358264
GI_499036295
GI_554821891
GI_583995367
GI_542255251
ENSGACG00000009555
ENSTRUG00000018218
ENSTNIG00000012308
ENSAMXG00000003869
ENSDARG00000004877
958
999
986
1000
472
1000
1000
586
1000
980
934
1000
1000
GI_499017063
GI_548401842
GI_545786390
GI_584008970
GI_542208870
ENSPFOG00000012199
ENSPFOG00000012091
ENSXMAG00000009185
ENSORLG00000018255
ENSTRUG00000010769
ENSTNIG00000008912
ENSGACG00000012788
ENSAMXG00000021207
ENSDARG00000017500
ENSLOCG00000016666

Figure S1

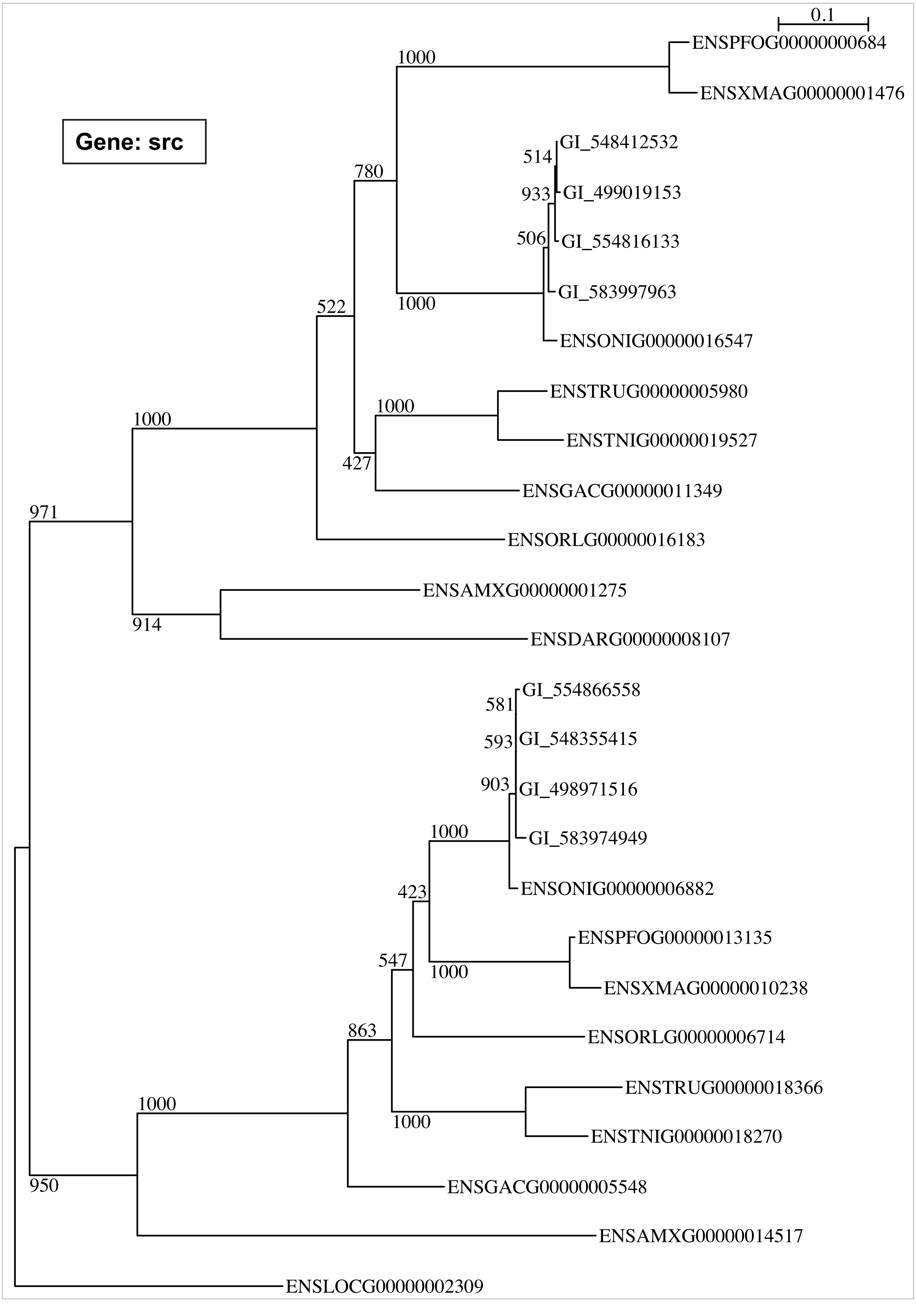

Gene: src
0.1
1000
ENSPFOG00000000684
ENSXMAG00000001476
780
514
GI_548412532
933
GI_499019153
506
GI_554816133
1000
GI_583997963
522
ENSONIG00000016547
1000
ENSTRUG00000005980
1000
ENSTNIG00000019527
427
ENSGACG00000011349
971
ENSORLG00000016183
ENSAMXG00000001275
914
ENSDARG00000008107
581
GI_554866558
593
GI_548355415
903
GI_498971516
1000
GI_583974949
423
ENSONIG00000006882
ENSPFOG00000013135
547
1000
ENSXMAG00000010238
863
ENSORLG00000006714
ENSTRUG00000018366
1000
1000
ENSTNIG00000018270
950
ENSGACG00000005548
ENSAMXG00000014517
ENSLOCG00000002309

Figure S1

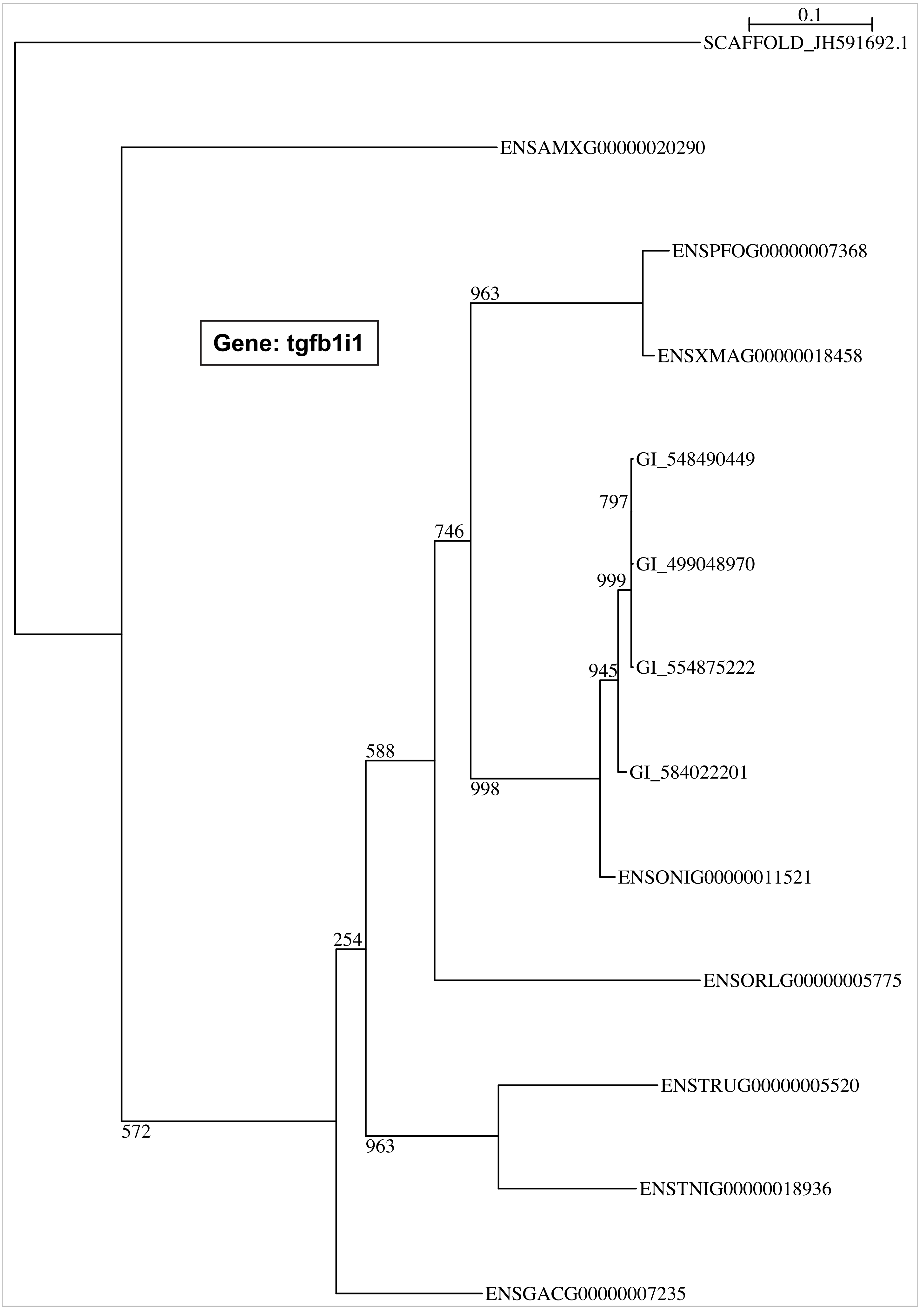
0.1
SCAFFOLD_JH591692.1
ENSAMXG00000020290
ENSPFOG00000007368
963
Gene: tgfb1i1
ENSXMAG00000018458
GI_548490449
797
746
GI_499048970
999
GI_554875222
945
GI_584022201
588
998
ENSONIG00000011521
254
ENSORLG00000005775
ENSTRUG00000005520
572
963
ENSTNIG00000018936
ENSGACG00000007235

Figure S1

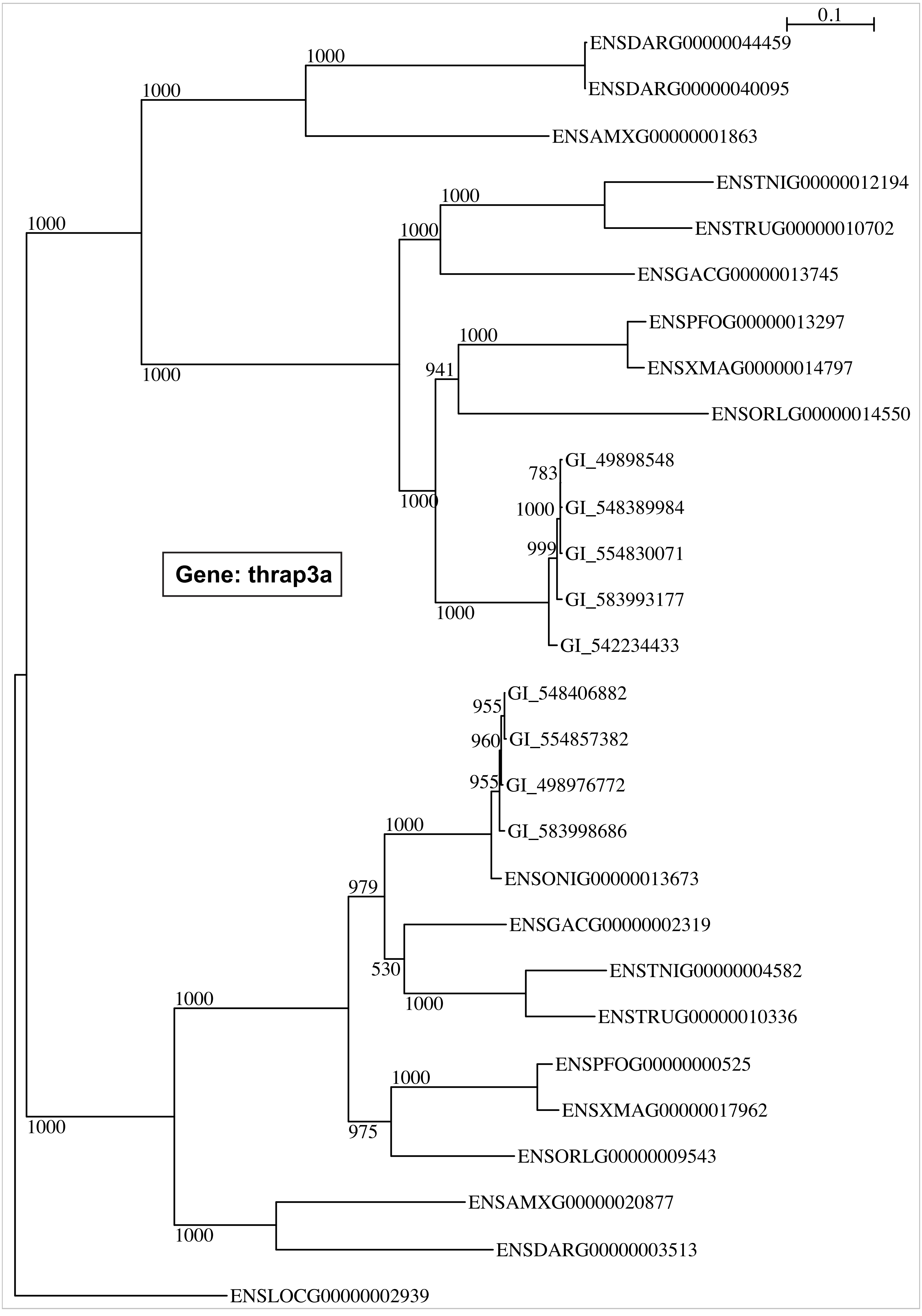
0.1
ENSDARG00000044459
ENSDARG00000040095
ENSAMXG00000001863
ENSTNIG00000012194
ENSTRUG00000010702
ENSGACG00000013745
ENSPFOG00000013297
ENSXMAG00000014797
ENSORLG00000014550
GI_49898548
GI_548389984
GI_554830071
GI_583993177
GI_542234433
Gene: thrap3a
GI_548406882
GI_554857382
GI_498976772
GI_583998686
ENSONIG00000013673
ENSGACG00000002319
ENSTNIG00000004582
ENSTRUG00000010336
ENSPFOG00000000525
ENSXMAG00000017962
ENSORLG00000009543
ENSAMXG00000020877
ENSDARG00000003513
ENSLOCG00000002939
1000
1000
1000
1000
1000
1000
1000
941
783
1000
999
1000
1000
955
960
955
1000
979
530
1000
1000
1000
975
1000
1000

Figure S1

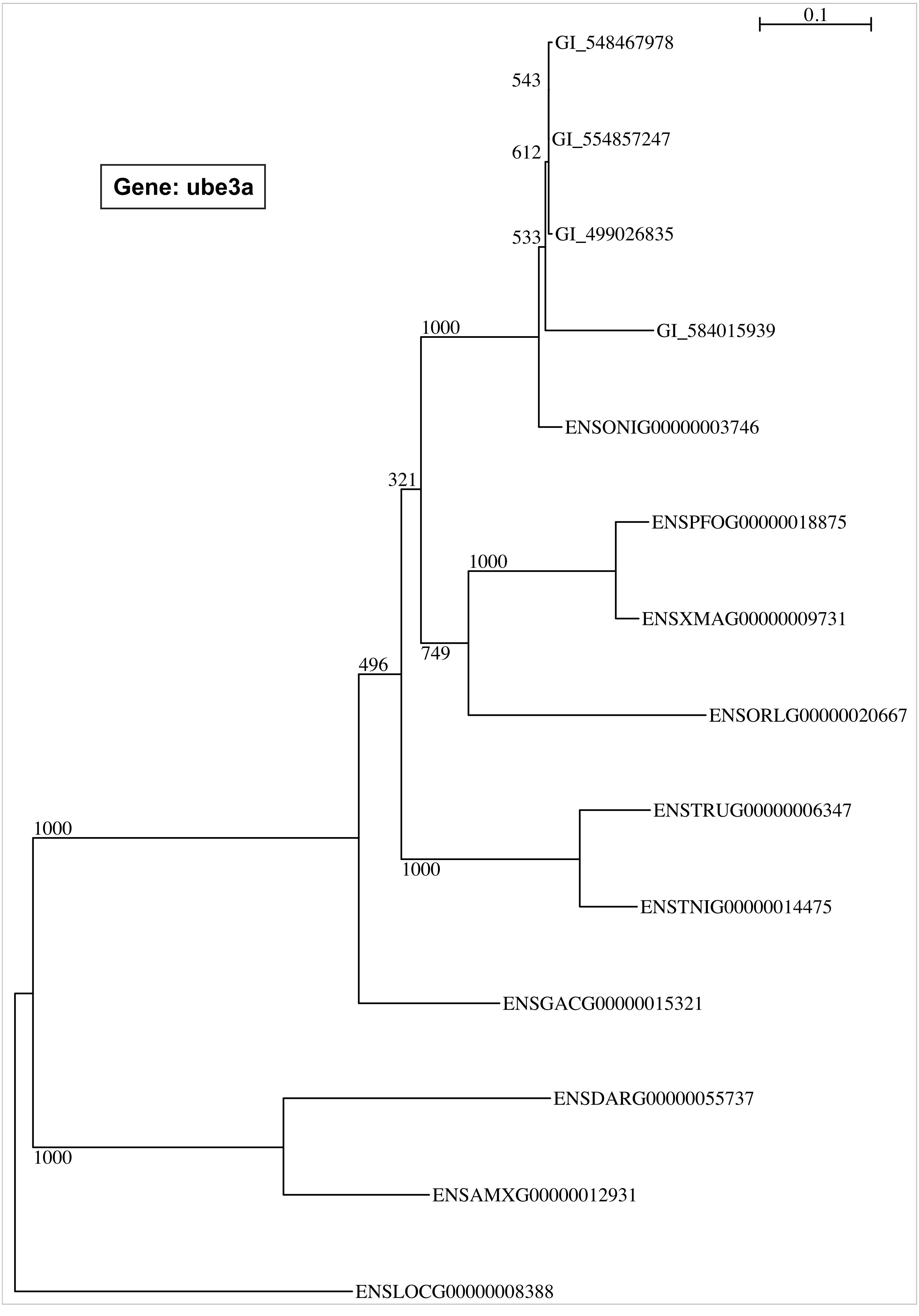
0.1
Gene: ube3a
GI_548467978
543
GI_554857247
612
GI_499026835
533
GI_584015939
1000
ENSONIG00000003746
321
ENSPFOG00000018875
1000
ENSXMAG00000009731
749
ENSORLG00000020667
496
ENSTRUG00000006347
1000
1000
ENSTNIG00000014475
ENSGACG00000015321
ENSDARG00000055737
1000
ENSAMXG00000012931
ENSLOCG00000008388

Figure S2

Site-wise Ka/Ks estimates using Selecton under the M8 model allowing for positive selection. In case positive selected sites were detected, they were compared to the null model M8a. If the likelihood ratio test returned significant results for positive selection, these are indicated below the sequence.

Figure S2

Gene: *akt1*

1	11	21	31	41
MNEVVIVKEG	WLHKRGEYIK	TWRPRYFLLK	SDGTFIGYKE	RPQDVDQLET
51	61	71	81	91
PLNNFSVAQC	QLMKTERPKP	NTFIIRCLQW	TTVIERTFHV	ETPEEREEWT
101	111	121	131	141
KTIQAVADSL	QKQEEEMMDC	RSGSPSDNIG	IEDMEVYLTK	PRLKVTMHDF
151	161	171	181	191
EYLKLLGKGT	FGKVILVKEK	ATGRYYAMKI	LKKEVIVAKD	EVAHTLTENR
201	211	221	231	241
VLQSSKHPFL	TGLKYSFQTH	DRLCFVMEYA	NGGELFFHLS	RERVFSEERA
251	261	271	281	291
RFYGAEIVSA	LDYLHSEKNV	VYRDLKLENL	MLDKDGHIKI	TDFGLCKEGI
301	311	321	331	341
KDGATMKTFC	GTPEYLAPEV	LEDNDYGRAV	DWWGLGVVMY	EMMCGRLPFY
351	361	371	381	391
NQDHEKLFEL	ILMEDIRFPR	TLAPEGQSLL	SGLLKKDPKQ	RLGGGPDDAK
401	411	421	431	441
EVMQHKFFAG	IVWQDVYEKK	LIPPFKPQVT	SETDTRYFDE	EFTAQTITIT
451	461	471		
PPGQDDNMES	MDSERRPHFP	QFSYSASGTA		

Legend:

The selection scale:

1	2	3	4	5	6	7

Positive selection Purifying selection

Figure S2

Gene: *ar*

```
1          11         21         31         41
MRQTNRQLPH RGLCIGTSHI SDTGVKEVQS ISFPRQTDVL ARSSHFAKRL
51         61         71         81         91
LGSRSEFSSV TKADDVEEGA YGFLPGLQGN TEQKSSIPIP PHHRKETKTH
101        111        121        131        141
GDALVESSNS DIASTTISET ARELCKAVSV SLGLNMDPNE MNDLGTSHDP
151        161        171        181        191
SLVSDRSQEN YLFQMHLADR SANGTGVTVT DYKCPIANDG PSVQSHENIR
201        211        221        231        241
MLKRVHANTT PEVVPSQHIS AAHSKQDGHE SRLNAELSNA TCEDSENHMN
251        261        271        281        291
MDGGHTVFCQ FDQSVRPNPA AYGQNDPLSV EQGNVASHGL YKCEAFPNAS
301        311        321        331        341
EENSEEVAAY PPEYRVRIKK EVSNEHADVW PVQYRYSDNG NTPYGLIRQF
351        361        371        381        391
THPQNTGQGT GSSFACNAYG EDGVSPEQWY SGGMVGRVPY TSSSCLKNEM
401        411        421        431        441
GDWMEVPYTD IRFESGRENL FPMEYFFPPQ RTCLICGDEA SGCHYGALTC
451        461        471        481        491
GSCKVFFKRA AEGKQKYLCA SRNDCTIDKL RRKNCPSCRL RKCFEAGMTL
501        511        521        531        541
GARKLKKIGQ LKGLEEAGNQ NPIETVQCMS PKPSITTHTQ PVFLNILESI
551        561        571        581        591
EPEVVNAGHD NAQPDSAACL LTSLNELGER QLVKVVKWAK GLPGFRSLHV
601        611        621        631        641
DDQMTVIQHA WMGVMVFALG WRSYKNVNAR MLYFAPDLVF NDHRMHISSM
651        661        671        681        691
YDHCIRMKRL AQEFAWLQIT QEEYLCMKAL LLFSIIPVEG LKSQTYFDEL
701        711        721        731        741
RTNYIHELDR LVSSGRKTSS NYSQRFFQLT QLMDSLQPIV RMLHQFTFEL
751        761        771        781
FVQAQSYPTK VNFPEMMSEI ISVQVPKMLA GMVKPILFHK
```

Legend:

The selection scale:

1	2	3	4	5	6	7

Positive selection   Purifying selection

Figure S2

Gene: *arid1a*

1 11 21 31 41
MAAQVASVAT LNTSPPSELK KADRDPQEEP VPGEKQQENK EPGSESGSPG
51 61 71 81 91
QKELQDGADG GNAGGGGDPE MKNGNGNPSR VNNNNNQNDS GAPEGNNHPG
101 111 121 131 141
MAHHHPAAFP PPPYGYSPHY GRGPFHQHGG QQSPGMAAAA GPAVQPGSMM
151 161 171 181 191
DSYQPNSHDH GFPNHQFNNY SPFPNRTAYP GQGYTMNSPR NNQPPAAGGQ
201 211 221 231 241
PAKQQQQQPP PPAGGTTAMA VTYNNQRYTM GNPQPTSTPT LNQLLTSPSS
251 261 271 281 291
TRGYQNYPPS DYSNQEGANK GPVDVGSSSQ YGGHQGWQQR THHPPPMSPG
301 311 321 331 341
STGQPLSRNQ PSSPMDQMGK MRGQPYAAAN PYSQQPQQGP PPGSQQGASY
351 361 371 381 391
PGQGYGPPTP QRYPMGMQSR TPGGMGSMQY GQQMPPYGQQ GPAGYGQQGQ
401 411 421 431 441
ASYYSQQAQP PHAAQQQSPY PQQPQSQPGA QTPYSQPSAP LAQAQPPYSQ
451 461 471 481 491
PHQTPQSQAQ QQQQQQQQQP PPVPPQSQAP YSQSQAPQSA QPPYPQQQVP
501 511 521 531 541
PPPPQQQQQP PSQPPPPQPT PATQPQSQQP PGHAPPQQSQ AAPYSQAPPQ
551 561 571 581 591
QQQQQPSPYQ RFPPPPQELS QDSFSSQSSV PPSNPAMASS KSGSEDVNMQ
601 611 621 631 641
GRPSSLPDLS GSIDDLPTGT EGALSPGVST SGVSSSQGEQ SNPAQSPFSP
651 661 671 681 691
HTSPHLPGIR GPSPSPVGSP ASVTQSRSGP LSPAGVPGTQ MPPRPPSGQS
701 711 721 731 741
ESILHPSMNQ SAMGQDRVYM QRNPQMPAYG SPQPGSALSP RQSSGGQMHA
751 761 771 781 791
GMGPYQQNNS MGNYGPQGAQ YGPQGYPRQP NYTGMPNASY PGPGMGGSMN
801 811 821 831 841
PMPGQGGGPP YTGMPPGRMG PGQMGTRPYG PNMGPNMGSM PPQVGSGMCP
851 861 871 881 891
PPGGLNRKAQ EAAAAAMHAA VNSSHNRPPG YPNMPQSGMI PAGTPYGQSM
901 911 921 931 941
NSMPGMMNPQ GPPYPMGGNM PNNTAGMAPS PELMGLDVKL NQAQKMNNKA
951 961 971 981 991
DGTPKPETKS KKSSSSTTTN EKITKLYELG PEPERKMWVD RYLAFTEEKA
1001 1011 1021 1031 1041

Figure S2

MGMNNLPAVG RKPLDLFRLY VSVKEIGGLT QVNKNKKWRE LATNLNVGTS
1051 1061 1071 1081 1091

SSAASSLKKQ YIQCLYAFEC KIERGEDPPP DIFSAAEAKK NQPKIQPPSP
1101 1111 1121 1131 1141

AGSGSLQGPQ TPQSTSSSMA EGGDLKPPTP ASTPHSQMPP MPGVRSSVVS
1151 1161 1171 1181 1191

LQDPFADSSD PAFPRRNSMT PNSAYQQGMN TPDMMGRMPY EPNKDPFSAM
1201 1211 1221 1231 1241

RKGGEFMSPG QGPNSGMGEQ YNRAPPGSMG NMGMGQRQQY PYGPGYDRRQ
1251 1261 1271 1281 1291

EPGMGPEGSM GPGAPQPNLM PSNADTGMYS PSRYPPQQQR HDSYGNQYPG
1301 1311 1321 1331 1341

QGAPSGGPYP NQPPGMYAQQ QPNYKRPVDG GYGPPAKRHE GEMYNVPFSG
1351 1361 1371 1381 1391

QQQQQAQQQT APPAQQEMYS QYGNAYAGSE RRPPGPQNQF PFQFGRERVQ
1401 1411 1421 1431 1441

ATAGPNSQQS MPPQMMGSPM QSTPDGPQGS MWPNRNDMGY NFPNRQGPGA
1451 1461 1471 1481 1491

AAQGPGYHSM NRSEEMMPSD QRMNHEGQWP AHVNQRQPPY GPSGPVPPMT
1501 1511 1521 1531 1541

RPLQPNYQTP PAIQNHIPQV SSPAPMPRPI ESRTSPSKPF MHSGIKMQKA
1551 1561 1571 1581 1591

GPPVPASHIT PAPVQPPLIR RDITFPPGSI EATQPILKPR RRLTMKDIGT
1601 1611 1621 1631 1641

PEAWRVMMSL KSGLLAESTW ALDTINILLY DDNSIATFSL CQLPGFLELL
1651 1661 1671 1681 1691

VEYFRRCLIE IFGILKEYEV GDPGQRTLLD PEGLNSERDT GSEDEEQEPE
1701 1711 1721 1731 1741

EAEEMEEEDE DEDEEQPEAS EQQQQQQQTP QPPALEKQEG EQQNGERAGE
1751 1761 1771 1781 1791

QQEEQEGEAA VKDPSVLTLS QDTGTAQEKP KQASKFDKLP IKLVRKKDPF
1801 1811 1821 1831 1841

VVDCSDKLGR LQEFDSGLLH WRIGGGDTTE HIQTHFESKL DLLQARKRVP
1851 1861 1871 1881 1891

PASGGSAGRK KSPAGENVTE GVEKVKTSEE QPPAKSITAT IDDVLSARPG
1901 1911 1921 1931 1941

SMTVEAVRGT PESHKENSKF LFSINPAQSH RNIKILEDEP RSKDETPLST
1951 1961 1971 1981 1991

IADWQDSLAK RCICVSNIVR SLSFVPGNDL EMSKHPGLLL LLGRLILLHH
2001 2011 2021 2031 2041

EHPERKQAPL TYEKEEEEDE GVSCEKDEWW WDCLELLREN TLVTLANISG
2051 2061 2071 2081 2091

QLDLSIYPES ICLPLLDGLL HWAVCPSAEA QDPFPTLGVN GVLSPQRLVL
2101 2111 2121 2131 2141

ETLSKLSIQD NNVDLILATP PFSRLEKLYG TLVRLVGERK IPVCREMAVV
2151 2161 2171 2181 2191

Figure S2

LLANLAQGDS LAARAIAVQK GSIGNLLGFL EDSLAATQFQ QSQSSLLHMQ
2201 2211 2221 2231 2241
SAPFEPTSVD MMRRAARALH AMAKVEENHS EFTLYESRLL DISVSPLMNS
2251 2261
LVSHVICDVL FLIGQS

Legend:

The selection scale:

1	2	3	4	5	6	7

Positive selection Purifying selection

Figure S2

Gene: *brca1*

```
1          11         21         31         41
MAAPTCKVED VKQVVSMIRR NLECPICLDL LRDPVSTRCD HQFCRMCMLK
51         61         71         81         91
HLSGSKRAGA QCPLCKAEVT KRSLQDSSRF KQLVEALQRT IHAFEQDTGT
101        111        121        131        141
KYFSEVLLEQ KNGLGGLTEA QAIGTKTRAE SSACERLGGR ITDSESQQEL
151        161        171        181        191
EAAISSAAAA KEGFAKLMDL DDSSYFANSG TAVAGSSEPT TQDRERSAAD
201        211        221        231        241
VTDPVIPVRL HMGNNRDLTS SREEEEHSFV SASKGENKTF DVSEKSKTNN
251        261        271        281        291
KVSSNKILEK RRKTSVQKVM DWLSRIDEKA CFPEEENRPA ETPSDADSLL
301        311        321        331        341
ATEKCDELVQ ECVESLKKTD RRSTLEDQVF GVVYKRARRT SAQLNKSFSS
351        361        371        381        391
DRKGSSKEHQ ANKKVCKRKS NELTPADFIK RPSPVAETSD ATSQPARSDA
401        411        421        431        441
GGRVRSESLH VLVSDGNQPE IEQRPSPSQR TARAETTQAG GSWKDEEEDL
451        461        471        481        491
QPDRQERKGK KSKPNTKDNR KRNALTRKKA IKAAKPLDLV SCQQNNTGQD
501        511        521        531        541
ADSAGPWKPT INETEAQIES YPSSEEPAGR AGEPHATRRS RRLQLLTEKV
551        561        571        581        591
RCTGRRLRTE DSELERDPQA PDQSENDQAG MLTCKGVEQL CESSEKENET
601        611        621        631        641
TEVLKVSEGY EDIQQQSGHI VVESSLVSII RNAESPCNSN KAFKGDQLVP
651        661        671        681        691
SALDISEGPD TGSDVAVMDE LLPGELTEGK NDSELETEQI LKTFKISKRR
701        711        721        731        741
SFNLLPSPEY PSQESEFCDN KASEKNENPE EEKGQVNVAV ERTSLQSEHV
751        761        771        781        791
ETENSGCTDI IPPTKPASPS CRNVDIKSAD KISDGSTSTF SSICTDSQRP
801        811        821        831        841
RDRNGRRKKT KMDESTLGSV RGRKKNLNLS SIHFSQVPPN TEQLVCASLL
851        861        871        881        891
NRQIPIDGLP QENESLVENC KVKSTMKESF VGNGEQSDFV QSFPLARKEL
901        911        921        931        941
STGPNISQES SMTPDGLLPL RAVASDRADN EEQCLPGEGK EQLSQWSLNS
951        961        971        981        991
NPRKRMRARM LESSDSDSNT EEDLPSLAEL LGPKQPATNP QEDSNQWNPL
1001       1011       1021       1031       1041
```

Figure S2

QNRDLEASPS STVESSVVLP VQQSVPASQG SVDLFSTQSD SPSHRIEQID
1051 1061 1071 1081 1091
LVETESSQSE ILTTQQKDTI QKDLRRIEEE MAVLEAALKG QPDYPKSSLQ
1101 1111 1121 1131 1141
TVVAQQSTET QPVSHEFRAL PCHVQLNKSV KRNLIKPLEK HANENTNIKP
1151 1161 1171 1181 1191
GKHIFYPGFS VFILSSCFEG LLSVAKLGIG TTKQSGSSGA CDASGPIPNP
1201 1211 1221 1231 1241
RVSADRGAAR GRMVLVGSGL DRSELIMVQK FARKMDGTLA AQVTPGTTHV
1251 1261 1271 1281 1291
IMKTDPELVC ERTLKYFLGI AGRMWVVSFL WIRECFRQGQ LLDEAAFEVK
1301 1311 1321 1331 1341
GDVINGRNHQ GPRRARTATR EELLLSNYEI CCHGSFTDMT TGQLEWMVEL
1351 1361 1371 1381 1391
CGATVAKEPF LFTYNPELIQ VVVVQPEAEE SQTDYKALQR KYSARVVTRE
1401 1411 1421
WVLDSVALYS CQALDSYLVK PLS

Legend:

The selection scale:

1 2 3 4 5 6 7

Positive selection Purifying selection

Figure S2

Gene: *cav1*

1	11	21	31	41
MTGGIKDEES	EGFLHSPFIR	KQGNIYKPNN	KSMDNDSMNE	KAMHDVHTKE
**51**	**61**	**71**	**81**	**91**
IDLVNRDPKH	LNDDVVKVDF	EDVIAEPEGT	HSFDGIWKAS	FTTFTVTKYW
**101**	**111**	**121**	**131**	**141**
CYRLLTALVG	IPLALIWGLF	FAILSFLHIW	AVVPCVKSYL	IEIQCVSRVY
**151**	**161**	**171**		
SICIHTFCDP	LFEAMGKVFS	GVRVSMTKEV		

Legend:

The selection scale:

1	2	3	4	5	6	7

Positive selection   Purifying selection

Figure S2

Gene: *ccne1*

```
1          11         21         31         41
MPRKNLKTGL QTEANDEVTK ERSTTRSRKR KADVAIHLQD PDEEVIEMTN
51         61         71         81         91
KKQYDTQTSW NPATGCTSPC RLICTPDKEV DEPVALNDVG CAQYMFKNIF
101        111        121        131        141
ITPTRSSPLP VLCWANKDDV WNNLLQKDKV YVRDNRFMER HPHLQPKMRA
151        161        171        181        191
ILLDWLMEVC EVYKLHRETF YLAQDFFDRF MATQMNVVKT RLQLIGISSL
201        211        221        231        241
FIAAKLEEIY PPKLHQFAYV TDGACTEDEI LDMELIIMKE LKWSLSPLTA
251        261        271        281        291
MSWLNIYMQV AYLKESSEML IPQYPQTTFV QIAELLDLCA LDVRCLEFSY
301        311        321        331        341
GVLAASALFH FSSVELVQKV SGYQLQDLEP CVKWMVPFAM SIREVGSSEL
351        361        371        381        391
KKFKGIAADD LHNIQTHAAL LEWLEKAYAY EAMGLDQNQR SPVPLSVLTP
401        411
PPSSEKTGVS ES
```

Legend:

The selection scale:

1	2	3	4	5	6	7

Positive selection   Purifying selection

 T. Lorin, W. Salzburger & A. Böhne

Figure S2

Gene: *cdc42*

1	11	21	31	41
MQTIKCVVVG	DGAVGKTCLL	ISYTTNKFPS	EYVPTVFDNY	AVTVMIGGEP

51	61	71	81	91
YTLGLFDTAG	QEDYDRLRPL	SYPQTDVFLV	CFSVVSPSSF	ENVKEKWVPE

101	111	121	131	141
ITHHCPKTPF	LLVGTQIDLR	DDPSTIEKLA	KNKQKPITPE	TAEKLARDLK

151	161	171	181
AVKYVECSAL	TQKGLKNVFD	EAILAALEPP	EPKKKRKCKL L

Legend:

The selection scale:

1	2	3	4	5	6	7

Positive selection Purifying selection

Figure S2

Gene: *cdk7*

```
1          11         21         31         41
MAVDVKSRAK RYEKLDFLGE GQFATVYKAR DKNTNTIVAI KKIKVGHRAE
51         61         71         81         91
AKDGINRTAL REIKLLQELS HPNIIGLLDA FGHKSNISLV FDYMETDLEV
101        111        121        131        141
IIKDTSLVLT PANIKAYILM TLQGLEYMHQ HWILHRDLKP NNLLLDENGV
151        161        171        181        191
LKLADFGLAK AFGSPNRVYT HQVVTRWYRS PELLFGARMY GVGVDMWAVG
201        211        221        231        241
CILAELLLRV PFLPGDSDLD QLTKIFEALG TPTEETWPGL TSLPDYISFK
251        261        271        281        291
MFPGTPLEHI FSAAGDDLLD LLRGLFTYNP CTRITATQAL KKRYFSNRPG
301        311        321        331        341
PTPGPQLPRP NCSVEALKEQ ENFKIGVKRK RDGTEQGSLK KKLIF
```

Legend:

The selection scale:

1	2	3	4	5	6	7

Positive selection Purifying selection

Figure S2

Gene: *cfl1l*

1	11	21	31	41
MASGVAVHDD	VVNIFNAMKV	RKAGEAEQDR	LKAILFRLSN	DEKQIIVDEG
51	61	71	81	91
SELHVRDLQP	GEDIFKRVVG	LLPEKDCRYA	LYDSSYETKD	SKKEDLVFIM
101	111	121	131	141
WAPENAPLKR	KMIYASSKNA	LRSKLTGIKF	EWQINEMGEI	KDPSTLVEKF
151	161			
GGKGLVVSVE	GKPI			

Legend:

The selection scale:

1	2	3	4	5	6	7

Positive selection   Purifying selection

Figure S2

Gene: *ctnnb1*

1	11	21	31	41
MATQADLMEL	DMAMEPDRKA	AVSHWQQQSY	LDSGIHSGAT	TTAPSLSGKG
51	61	71	81	91
NPEDDDVDNQ	VLYEWETGFS	QSFTQDQVAD	IDGQYAMTRA	QRVRAAMFPE
101	111	121	131	141
TLDEGMQIPS	TQFDGAHPTN	VQRLAEPSQM	LKHAVVNLIN	YQDDAELATR
151	161	171	181	191
AIPELTKLLN	DEDQVVVNKA	AVMVHQLSKK	EASRHAIMRS	PQMVSAIVRT
201	211	221	231	241
MQNTNDVETA	RCTAGTLHNL	SHHREGLLAI	FKSGGIPALV	KMLGSPVDSV
251	261	271	281	291
LFYAITTLHN	LLLHQEGAKM	AVRLAGGLQK	MVALLNKTNV	KFLAITTDCL
301	311	321	331	341
QILAYGNQES	KLIILASGGP	QALVNIMRTY	TYEKLLWTTS	RVLKVLSVCS
351	361	371	381	391
SNKPAIVEAG	GMQALGLHLT	DPSQRLVQNC	LWTLRNLSDA	ATKQEGMEGL
401	411	421	431	441
LGTLVQLLGS	DDINVVTCAA	GILSNLTCNN	YKNKMMVCQV	GGIEALVRTV
451	461	471	481	491
LRAGDREDIT	EPAICALRHL	TSRHQDAEMA	QNAVRLHYGL	PVVVKLLHPP
501	511	521	531	541
SHWPLIKATV	GLIRNLALCP	ANHAPLREQG	AIPRLVQLLV	RAHQDTQRRT
551	561	571	581	591
SMGGTQQQFV	EGVRMEEIVE	GCTGALHILA	RDVHNRIVIR	GLNTIPLFVQ
601	611	621	631	641
LLYSPIENIQ	RVAAGVLCEL	AQDKEAAEAI	EAEGATAPLT	ELLHSRNEGV
651	661	671	681	691
ATYAAAVLFR	MSEDKPQDYK	KRLSVELTSS	LFRTEPMTWN	ETGDLGLDIG
701	711	721	731	741
AQGEPLGYRQ	DDPSYRSFHS	GGYGQDAMGM	DPMMDHDMGG	HHPGAEYPVD
751	761	771		
GLPDLGHAQD	LIEGLPPGDS	NQLAWFDTDL		

Legend:

The selection scale:

1 2 3 4 5 6 7

Positive selection Purifying selection

Figure S2

Gene: *daxx*

```
1          11         21         31         41
XXXXXXXXXX XXXXXXXXXX XXXXXXXXXX XXXXXXXXXX XXXXXXXXXX
51         61         71         81         91
XXXXXXXXXX XXXXXXXXXX XXXXXXXXXX XXXXXXXXXX XXXXXXXXXX
101        111        121        131        141
XXXXXXXXXX XXXXXXXXXX XXXXXXXXXX XXXXXXXXXX XXXXXXXXXX
151        161        171        181        191
XXXXXXXXXX XXXXXXXXXX XXXXXXXXXX XXXXXXXXXX XXXXXXXXXX
201        211        221        231        241
XXXXXXXXXX XXXXXXXXXX XXXXXXXXMV TDSSLSPLPP AQIDKFINSQ
251        261        271        281        291
ESRLCPPDYS DILRVVRRAD ERHGLGLSRR QLTQIAQDAF RDTGNSLQER
301        311        321        331        341
RHLDMVYNFG SHLTDGYQPA TDPALADPTL DRRLRTNRTV ALISLDDVIX
351        361        371        381        391
XXXXXXXXXX XXXXXXXXXX XXXXXXXXXX XXXXXXXXXX XXXXXXXXXX
401        411        421        431        441
XXXXXXXXXX XXXXXXXXXX XXXXXXXXXX XXXXXXXXXX XXXXXXXXXX
451        461        471        481        491
XXXXXXXXXX XXXXXXXXXX XXXXXXXXXX XXXXXXXXXX XXXXXXXXXX
501        511        521        531        541
XXXXXXXXXX XXXXXXXXXX XXXXXXXXXX XXXXXXXXXX XXXXXXXXXX
551        561        571        581        591
XXXXXXXXXX XXXXXXXXXX XXXXXXXXXX XXXXXXXXXX XXXXXXXXXX
601        611        621        631        641
XXXXXXXXXX XXXXXXXXXX XXXXXXXXXX XXXXXXXXXX XXXXXXXXXX
651        661        671        681
XXXXXXXXXX XXXXXXXXXX XXXXXXXXXX XXXXXXXX
```

Legend:

The selection scale:

1	2	3	4	5	6	7

Positive selection — Purifying selection

Figure S2

Gene: *dnaja1*

1	11	21	31	41
MVKETGYYDV	LGVKPNASAD	ELKKAYRKLA	LKYHPDKNPN	EGEKFKQISQ
51	61	71	81	91
AYEVLSDSKK	RELYDRGGEQ	AIKEGGMGGG	GFGSPMDIFD	MFFGGGGRMH
101	111	121	131	141
RERRGKNVVH	QLTVSLEDLY	NGATRKLAVQ	KNVICDRCEG	RGGRRGVVEC
151	161	171	181	191
CPMCRGSGMQ	VRLHQIGPGM	VQQISTICVG	CQGQGQRISQ	KDRCKACNAR
201	211	221	231	241
KIVRQKKILE	VHIDKGMKDG	QKITFHGEGD	QEPGLEPGDI	IIVLDQKVHP
251	261	271	281	291
VYTRQGEDLI	MNLEIKLVEA	LCGFQKPIKT	LDDRILLLTS	HPGEVIRPGD
301	311	321	331	341
RKCIMNEGMP	FHRRPFEKGR	LIVLFTVNFP	EENFLPLDKL	TQLERYLPER
351	361	371	381	391
EETDDTENMD	DLYIYADLED	CDLSQERPHF	DDDEYHSSGG	VQCQTS

Legend:

The selection scale:

1 2 3 4 5 6 7

Positive selection Purifying selection

Figure S2

Gene: *egfr*

```
1          11         21         31         41
MQFEIAIVLS SLLTLGCCSI QDKRVCQGLS NKLTLLGTSQ DHYNNIVRMY
51         61         71         81         91
SNCTVVMENL EITYVEKDYD LSFLKSIREV GGYVLIALNK VSRIPIDNLR
101        111        121        131        141
IIRGHILYQN SYALAVLSNF DKQSSVNKNQ SSNVGLQELS LKSLTEILKG
151        161        171        181        191
GVKFTGNPFL CNAETIQWYD IVDKSKNPEM FLIFANNTLN CKKCDPNCFN
201        211        221        231        241
GSCWAPGPSN CQTLTKLNCA EQCSHRCKGP KPSDCCNEHC AAGCTGPRST
251        261        271        281        291
DCLACRDFQD EGTCKQVCPQ LMLYNPNTHQ LEMNPDGKYS FGATCVKSCP
301        311        321        331        341
HNYVVTDHGS CVRTCTANTH EVDENGVRKC KKCEGPCPKA CNGLGMGNLV
351        361        371        381        391
NVLSINASNI DSFRNCTKIN GDVSILPVAF RGDSYTKTPV LDPSKLDVFK
401        411        421        431        441
TVKEITGFLL IQAWPENMTS LSPLENLEII RGRTKQHGTV SVAAVNIDIT
451        461        471        481        491
SLGLRSLKEI SDGDVVIRGN PHLCYTNADQ WKRLFKLEKQ NARVSENADP
501        511        521        531        541
TECSALTQTC DELCTSEGCW GPGPSMCFSC QHFMRQQQCV NACNVLEGLP
551        561        571        581        591
REFIMDKKCI ECDPECMPQN GTQTCTGSGP DKCAECAHFK DGPHCVHKCP
601        611        621        631        641
SGIPGENDTF IWKYADEKKV CQLCHPNCTQ GCTGPGLAGC DHQTSQLSSI
651        661        671        681        691
AAGVVGGLLV TVIIALAIFI LMRRRYIKRK RTLRRLLQER ELVEPLTPSG
701        711        721        731        741
EAPNQALLRI LKETEFKKIQ VLGSGAFGTV YKGLWIPEGE DVKIPVAIKV
751        761        771        781        791
LREATSPKAN KEILDEAYVM ASVNNPHVCR LLGICLTSTV QLITQLMPYG
801        811        821        831        841
CLLDYVKEHK DNIGSQYLLN WCVQIAKGMN YLEERHLVHR DLAARNVLVK
851        861        871        881        891
TPQHVKITDF GLAKLLSADE KEYHADGGKV PIKWMALESI LHRIYTHQSD
901        911        921        931        941
VWSYGVTVWE LMTFGSKPYD GIPASEIAGI LEKGERLPQP PICTIDVYMI
951        961        971        981        991
MVKCWMIDAD SRPRFRELIA EFSKMARDPP RYLVIQGDER MHLPSPTDSK
1001       1011       1021       1031       1041
```

Figure S2

FYRTLIDGDE MDDVVDADEY LVPYNGFFNS PSTSRMQLLH SMSLNSSMGS

1051 1061 1071 1081 1091

CTIRNGQGYP MREDSFIQRY ITDPTDGAFE KDFQPVPEYV NQNGNPVSDV

1101 1111 1121 1131 1141

MNPIYQDSVV PISAVTRAQN ADSQYLNSFS NTADFHEYLN TVQPALPRPL

1151 1161 1171 1181 1191

FDNPHYCDQK VTHQTSLDNP DYQQDFFPPK SKVNGHLPAA ENTEYLALSA

1201

QAEVH

Legend:

The selection scale:

1	2	3	4	5	6	7

Positive selection   Purifying selection

Figure S2

Gene: *fhl2*

1	11	21	31	41
MTERFDCHYC	KESLFGKKYI	LREENPYCVK	CYESLYSNAC	EECKKPIGCN
51	61	71	81	91
SKDLSYKDRH	WHEDCFHCFQ	CKRSLVDKPF	SAKDELLLCT	ECYSNEYSSK
101	111	121	131	141
CHECKKTIMP	GTRKMEHKGN	SWHETCFTCH	RCQQPIGTKS	FIPKDAHNYC
151	161	171	181	191
VPCYEKQFAM	QCVQCKKPIT	SGGVTYHEQP	WHKDCFLCTG	CKKQLSGQRF
201	211	221	231	241
TSRDDFAYCL	NCFCNLYAKK	CAGCTNPISG	LGGSKYISFE	ERQWHNDCFN
251	261	271		
CKKCSVSLVG	RGFLTERDDI	LCPECGKDI		

Legend:

The selection scale:

1 2 3 4 5 6 7

Positive selection — Purifying selection

Figure S2

Gene: *fkbp4*

1	11	21	31	41
MTAEEVNAEG	QQTTQMEGED	ITPKKDGGVL	KSIKKEGTGD	ENPMIGDKVF
51	61	71	81	91
VHYVGTLLDG	TKFDSSRDRS	EKFSFDLGKG	QVIKAWDIGV	ATMKIGEVCQ
101	111	121	131	141
LICKPEYAYG	SAGSPPKIPP	NSTLVFEIEL	FGFQGEDLTD	DEDGGIIRRI
151	161	171	181	191
ATKGEGYSKA	NEGATVEVSL	EGTFEGRVFD	QRELKFEIGD	GESLGIPPGV
201	211	221	231	241
EKALQSMEKG	EESLITLSPK	YGFGTAGNSQ	FNIPPDAMLN	YKIKLISFEK
251	261	271	281	291
AKESWEMNTD	EKLEQSVIVK	EKGTQYFKEG	KYKQASVQYK	KIVSWLEHES
301	311	321	331	341
GLSEEYENKA	KALRLAAHLN	LAMCYLKVQE	HSQAVENCNK	ALELDSSNEK
351	361	371	381	391
ALFRRGEALF	AMKEFEQARD	DFQKVVQLYP	SNKAAKTQVV	VCQRQIKEQH
401	411	421	431	441
EKDKRLYANM	FQKFAERDAK	KEAEKQKDTK	KQENTEGQNG	DEMDIDQNET
451				
QEQTAA				

Legend:

The selection scale:

1	2	3	4	5	6	7

Positive selection — Purifying selection

Likelihood ratio test between the null model (no positive selection) and the alternative model (enabling positive selection) shows a significance level of: 0.05

Figure S2

Gene: *flna*

1	11	21	31	41
MSSPHPRLNQ	SSAPSSAAVS	NAALADKDAD	MPATEKDLAE	DAPWKKIQQN
51	61	71	81	91
TFTRWCNEHL	KCVNKRIANL	QTDLSDGLRL	IGLLEVLSQK	KMFRKYNQRP
101	111	121	131	141
TFRQMQLENV	SVALEFLDKE	NIKLVSIDSK	AIVDGNLKLI	LGLIWTLILH
151	161	171	181	191
YSISMPMWDE	EEDDDVAKQK	TPKQRLLGWI	QNKLPQLPIT	NFSRDWQSGK
201	211	221	231	241
ALGALVDSCA	PGLCPDWDSW	DQTKPVDNAR	EAMQQADDWL	GIPQVITPEE
251	261	271	281	291
IVDPNVDEHS	VMTYLSQFPK	AKLKPGAPLR	PKLNPKKARA	YGPGIEPTGN
301	311	321	331	341
VVMKKAEFTV	ETISAGMGEV	LVYVEDPAGH	REEAKVTANN	DKNRTYSVVY
351	361	371	381	391
IPKVTGTHKV	TVLFAGLHIS	KSPFDVDVGM	SQGDASKVTA	QGPGLEPSGN
401	411	421	431	441
IANKTTYFDV	YTAGAGIGEV	GVSIVDPTGK	KDTVECNIED	KGNSSYRCTY
451	461	471	481	491
KPTKEGVHTI	YITFAGSQIS	KSPFTVTVGE	ACNPSLCRAK	GRGLQPKGLR
501	511	521	531	541
VKETADFKVY	TKGAGTGDLK	VTIKGPKGLE	EPCKKKDLGD	GVYSFEYYPT
551	561	571	581	591
TTGNYTITIT	WGGQQIPRSP	FEVKIGTEAG	PQKVRAWGPG	LEGGVVGSSA
601	611	621	631	641
DFVVEAVGDD	VGTLGFSVEG	PSQAKIECDD	KGDGSCDVRY	WPTEAGEYAV
651	661	671	681	691
HVLCNNEDIQ	YSPFMAEIKP	SPGKDFHPEK	VKAYGPGLQS	TGLAMSKPAE
701	711	721	731	741
FTVDAKQGGK	APLKIQAQDG	DGNPVDVQVK	DNGNGTYSCF	YTPRKPLKHT
751	761	771	781	791
VMVSWGGVNI	PDSPFRMSIG	AGSHPNKVKV	SGPGVAKTGL	KAFEPTYFTV
801	811	821	831	841
DCSEAGQGDI	SIGIKCAPGV	VGPAEADIDF	DIIRNDNDTF	TVKYTPPGAG
851	861	871	881	891
SYTIMVLFAD	QAIPMTPFRI	KVDPSHDASK	VKAEGPGLSR	SGIEWNKPTH
901	911	921	931	941
FTVNTKGAGK	AKLDVQFTGP	TKADAVKDFD	IVNNHDNTYT	VKYTPVQQGN
951	961	971	981	991
MGVNVTYGGD	SIPKSPFSVG	VAPSLDLSKI	KVSGPGEKMT	VGKDQEFTVK
1001	1011	1021	1031	1041

Figure S2

SKGAGGQGKV AAKVTGPSGK PVPCKVEPSL SPETSQVRFI PREQGPYEVE
1051 1061 1071 1081 1091

LTYDGAPIPG SPFPVEAVAP ADPSKVRCSG PGLERAKVGE TGKFNVDCTN
1101 1111 1121 1131 1141

AGPAELTIEI ISDNGTEAEV HIQDNGDGTY TITYIPLYPG VYTITIRYGG
1151 1161 1171 1181 1191

QDVPNFPARL TVEPAVDSTG VKVFGPGVGN KGVFREATTD FTVDARALTK
1201 1211 1221 1231 1241

SGGNHIKTRI NNPSGNRTDA LIRDLGDGTY QVEYTPYEEG VHNVEVCYDD
1251 1261 1271 1281 1291

APVPNSPFRV PVTEGCDPAR VRVHGPGLQS GITNKPNKFT VETRGAGTGG
1301 1311 1321 1331 1341

LGLAVEGPSE AKMSCTDNKD GSCSVEYVPY EPGTYNLNIT YGGQPVKGSP
1351 1361 1371 1381 1391

FSVPVHDVVD ASKVKCLGQG LGNNVRANIP QSFTVDTSKA GVAPLQVRVQ
1401 1411 1421 1431 1441

GPKGIVEPVE VVDNGDKTHT VSYVPTREGP YSVSVLYADE EIPRSPFKVK
1451 1461 1471 1481 1491

VLPTHDASKV KASGPGLNTT GVPASLPVEF TIDAKDAGEG LLAVQITDPE
1501 1511 1521 1531 1541

GKPKKANIRD NQDGTYLVSY VPDMTGRYTI LIKYGGDDIP YSPYRIRALP
1551 1561 1571 1581 1591

TGDASKCTVT VSIGGHGLGA GVGPTIQIGE ETVITVDAKA AGKGKVTCTV
1601 1611 1621 1631 1641

CTPDGTEVDV DVVENEDGTF DIFYTAPQPG KYVICVRFGG EHIPNSPFQV
1651 1661 1671 1681 1691

TALDGAPTEQ MLQQTQVPQY AYAPGVGQPW ATDRPVGMNG LDVAGLRPFD
1701 1711 1721 1731 1741

LVIPFTIKKG EITGDVRMPS GKIAKPDITD NKDGTVTVKY APTEAGLHEM
1751 1761 1771 1781 1791

DIKYDGIHIP GSPLQFYVDY VNSGHVTAYG PGLIHGMVNK PAIFTVNTKD
1801 1811 1821 1831 1841

AGEGGLSLAI EGPSKADISC TDNQDGTCTV SYLPVLPGDY NILVRYNDKH
1851 1861 1871 1881 1891

IPGSPFVAKI TGDDSMRMSH LKVGSAADIP LDIGELDLSQ LTASLTTPSG
1901 1911 1921 1931 1941

REEPCLLKML RNGHVGISFV PKEIGEHLVN IKKNGRHIPS SPITVMINQS
1951 1961 1971 1981 1991

EIGDASRVRV SGPGLSEART FEPAEFIIDT REAGYGGLSL SIEGPSKVDI
2001 2011 2021 2031 2041

NTEDQEDGTC KVTYCPTEPG NYIINIKFAD QHVPGSAFTV KVTGEGRMKE
2051 2061 2071 2081 2091

SITRRRRAAS VANVGSQCDL SLKIPEINIG DMTAQVTSPS GKVHKAEIME
2101 2111 2121 2131 2141

GENNTYCIRF VPTEMGVHTV SVKYQGQHVP GSPFQFTVGP LGEGGAHKVR
2151 2161 2171 2181 2191

Figure S2

AGGPGLERAE AGIPAEFSIW TREAGAGGLS IAVEGPSKAE IAFEDRKDGS
2201 2211 2221 2231 2241
SGVSYIVQEP GDYEVSIKFN DEHIPDSPFI VPVASPSDDA RRLTVASLQE
2251 2261 2271 2281 2291
SGLKVNQPAS FAVSLNGAKG VIDAKVHSPS GALEECCVTE IDEDKYAVRF
2301 2311 2321 2331 2341
IPRENGLYLI DVKFNGSHIP GSPFKIRVGE TGQAGDPGMV SAYGAGLEGG
2351 2361 2371 2381 2391
NTGSPCEFIV NTSSAGPGAL AVTIDGPSKV KMDCQECPEG YKVIYTPMAP
2401 2411 2421 2431 2441
GNYLISIKYG GPYHIVGSPY KAKITGPRLV SSHSLHETSS VLVDPISRSS
2451 2461 2471 2481 2491
ATMQQGAPAW ASSDASHVVA KGLGLTKGFV GQKNSFSVDC SKAGRNMLLV
2501 2511 2521 2531 2541
GVDGPKVPCE EILVKHLGNR LYNVSYQLKE KGDYILVVKW GDEHIPGSPF
2551
HISVP

Legend:

The selection scale:

1 2 3 4 5 6 7

Positive selection Purifying selection

Figure S2

Gene: *gnb2l1*

1	11	21	31	41
MTEQMTVRGT	LKGHNGWVTQ	IATTPQFPDM	ILSASRDKSI	IMWKLTRDET
**51**	**61**	**71**	**81**	**91**
NYGIPQRALR	GHSHFVSDVV	ISSDGQFALS	GSWDGTLRLW	DLTTGTTTRR
**101**	**111**	**121**	**131**	**141**
FVGHTKDVLS	VAFSADNRQI	VSGSRDKTIK	LWNTLGVCKY	TIQDESHTEW
**151**	**161**	**171**	**181**	**191**
VSCVRFSPNS	SNPIIVSCGW	DKMVKVWNLA	NCKLKTNHIG	HTGYLNTVTV
**201**	**211**	**221**	**231**	**241**
SPDGSLCASG	GKDGQAMLWD	LNEGKHLYTL	DGGDTINALC	FSPNRYWLCA
**251**	**261**	**271**	**281**	**291**
ATGPSIKIWD	LEGKIIVDEL	RQEVISTSSK	AEPPQCTSLA	WSADGQTLFA
**301**	**311**			
GYTDNLIRVW	QVTIGTR			

Legend:

The selection scale:

1	2	3	4	5	6	7

Positive selection   Purifying selection

Figure S2

Gene: *grip1*

1 11 21 31 41
MERFLGFVKQ IRRSRRRKGK KYRPEEDYHE GYEDVYYYAS EHLTFVRDEG
51 61 71 81 91
PYTKPSNPSK PPDGALAVRR QSIPDEFKGS TIVELMKKEG TTLGLTVSGG
101 111 121 131 141
IDKDGKPRVS NLRQGGIAAR SDQLNVGDYI KSVNGINLTK FRHDEIISLL
151 161 171 181 191
KNVGERVVLE VEYELPPISV QGSGVIFKNV EVTLHKEGNT FGFVIRGGAH
201 211 221 231 241
EDRNKSRPVV ITTVRPGGPA DREGTVKSGD RLLSIDGIRL HGTSHAEAMS
251 261 271 281 291
ILKQCGQEAT LLIEYDVSVM DSIATASGPL LVEVAKSPGS SLGIALTTSM
301 311 321 331 341
YCNKQVIVID KVKPASIADR CGALHAGDHI LSVDGTSMEY CTLAEATQLL
351 361 371 381 391
ASACEHVKLE ILPHHQTRLP LKGSDHVKVQ RSNRQLPWDS CANNNRNFLP
401 411 421 431 441
YQHYNTYHPD HSRTQASKYQ KPSPNNLPLV SSSFSPTSMS AYSLSSLNMG
451 461 471 481 491
TLPRNMYPTS PRGTLMRRKL KKKDHKSSLS LASSTLGLAG QVVHTETTEV
501 511 521 531 541
TLVGDPILGF GIQLQGGVFA TETLSSPPLI AYMDPDSPAE RSGILQIGDR
551 561 571 581 591
ILAINGIPTE DSTLEETNQL LRDSSITSKV TLEIEFDVAE SVIPSSGTFH
601 611 621 631 641
VKLPKKPGVE LGITISSPSS RKPGDPLIIS DIKKGSVAHR TGTLELGDKL
651 661 671 681 691
LAIDNIRLDN CSMEDAVQIL QQCEDLVKLK IRKDEDNSDE QESSGAIIYT
701 711 721 731 741
VELKRYGGPL GITISGTEEP FDPIIISSLT KGGLAERTGA IHIGDRILAI
751 761 771 781 791
NSNSLKGKPL SEAIHLLQMA GESVTLKIKK QGEATSPKKP SASGRLSELS
801 811 821 831 841
DVEDESQAAQ KTGKLSDMYS TTIPSVDSAV ESWDGSGIDT VFGTQVPGYQ
851 861 871 881 891
ASGYSFHSHE WRNAKSRGSL SPVSRQRNNI FQDIGLSDDE WDRPTTSGFT
901 911 921 931 941
VGNDGTEPDQ EENFWSQALE DLETCGQSGI LRELEATIMS GSTMSLNHEP
951 961 971 981 991
QPQRSLLGRQ ASFQERSLSR PQYTPTNRSN TLPTEAGRKA FAMRKIKQEM
1001 1011 1021 1031 1041

Figure S2

```
KDIMSPTPVE LHKVTLYKDT DAEDFGFSVS DGLLEKGVYV NNIRPSGPAD
1051       1061       1071       1081       1091
LGGLKPYDRL LQINHVRTRD FDCCLVVPLI AESGNKLDLV ISRNPLASQP
1101       1111       1121       1131       1141
VTELDQQPSE APSEWSEQND ALQQPQQQQS SRLAGVDARE PTKTL
```

Legend:

The selection scale:

1	2	3	4	5	6	7

Positive selection Purifying selection

T. Lorin, W. Salzburger & A. Böhne

Figure S2

Gene: *kat5*

1	11	21	31	41
MADTSMEIVE	GCRLPVLRKN	QENEDEWPLA	EILSVKDTLG	RKLFYVHYID
51	61	71	81	91
FNKRLDEWVT	PDRLDMKKLQ	FPKKEAKTPT	KNGLPGSRPS	SPEREVRKSL
101	111	121	131	141
DLNVQAASAP	SRGKTLPTPK	RKAETVSLAS	QVTPATSVPS	LPSSAEATQA
151	161	171	181	191
SVYPAARETF	TTKTREDEPL	TSITTVTNGT	TRRVLPPSQP	GRKRKTCGTD
201	211	221	231	241
EMIKVFQYNS	PRTTSGCLPP	GEDSQDSSDG	IPTAPRMTGS	LVSDRSHDDI
251	261	271	281	291
ITRMKNIDCI	ELGRHRLKPW	YFSPYPQELT	TLPILYLCEF	CLKYLKSLKC
301	311	321	331	341
LQRHLTKCNL	RHPPGNEIYR	KGTISFFEID	GRKNKTYSQN	LCLLAKCFLD
351	361	371	381	391
HKTLYYDTDP	FLFYVMTEYD	CKGFHIVGYF	SKEKESTEDY	NVACILTLPP
401	411	421	431	441
YQRRGYGKLL	IEFSYELSKV	EGKTGTPEKP	LSDLGLLSYR	SYWSQTILEI
451	461	471	481	491
LMDLKSDNGE	RPQITINEIS	EITSVKKEDV	ISTLQYLNLI	NYYKGQYILT
501	511	521	531	
LSEDIVDGHE	RAMQKRHLRI	DPKCLHFTPK	DWSKRGKW	

Legend:

The selection scale:

1	2	3	4	5	6	7

Positive selection — Purifying selection

Figure S2

Gene: *limk2*

1	11	21	31	41
MEDRQGDESY	RCVGCGSRIL	DTFYIKVLQD	VWHNSCFQCS	ECCDHLTNWY
51	61	71	81	91
YEKDGKLYCR	KHYWENFGES	CHGCSLLMTG	PVMAAGEYKY	HPECFVCLSC
101	111	121	131	141
KVVIEERDTY	ALVERSKLYC	GTCHQQIVLT	PVLEKRSSVL	ACDSLPHTVT
151	161	171	181	191
LISVPAATNG	KRGLSVSVIG	DCSGGTTSVQ	VKEVLGMHIS	PEVRNAIHAG
201	211	221	231	241
DRILEINGVP	VGTLMEEEVE	DLIQRTNQTL	QLVIEYDPVR	QRLDRLRLGS
251	261	271	281	291
PRSRLRVPGA	SRLRLPSPAD	PVLESRETLE	DSTLKRRSLR	RSNSICKSPG
301	311	321	331	341
PASPKEYPGL	FRDISRSESL	RSSTSCSHRI	FRPCDLIHGE	VLGKGFFGQA
351	361	371	381	391
IKVTHKATGE	VMVMKELIRC	DEETQKTFLK	EVKVMRSLEH	PNVLKFIGVL
401	411	421	431	441
YKDKRLNLIT	EFIEGGTLKD	FIRDMDPFPW	EQRVSFAKGI	ASGMAYLHSM
451	461	471	481	491
SIIHRDLNSH	NCLVKLDNTV	VVADFGLSRL	IVEEKSRAPP	EKQSAKKRTF
501	511	521	531	541
RRIDRKKRYT	VVGNPYWMAP	EMLNGKRYDE	RVDIFSFGIV	LCEIIGQVYA
551	561	571	581	591
DPECLPRTLD	FGLNVRKFLE	KFLPEDCPSA	FFPLAVACCD	LVPENRPTFE
601	611	621	631	641
KLVDCFDALA	LNLELGIPLP	AELEVLHHHL	CGAHSPVGAD	LPGSGPPVRH
651				
PASASLGENG S				

Legend:

The selection scale:

1	2	3	4	5	6	7

Positive selection Purifying selection

Figure S2

Gene: *mapk1*

1	11	21	31	41
MATAAVSGAA	GGAPNTGSGA	ELVRGQAFDV	GPRYSNLSYI	GEGAYGMVCS
51	61	71	81	91
AYDKENKVRV	AIKKISPFEH	QTYCQRTLRE	IKILLRFKHE	NIIGINDIIR
101	111	121	131	141
APSIDQMKDV	YIVQDLMETD	LYKLLKTQHL	SNDHICYFLY	QILRGLKYIH
151	161	171	181	191
SANVLHRDLK	PSNLLLNTTC	DLKICDFGLA	RVADPDHDHT	GFLTEYVATR
201	211	221	231	241
WYRAPEIMLN	SKGYTKSIDI	WSVGCILAEM	LSNRPIFPGK	HYLDQLNHIL
251	261	271	281	291
GILGSPSQED	LNCIINIKAR	NYLLSLPLRN	KVPWNRLFPN	ADPKALDLLD
301	311	321	331	341
KMLTFNPHKR	IEVEEALAHP	YLEQYYDPTD	EPVAEAPFKF	DMELDDLPKE
351	361			
TLKELIFEET	ARFQPGYRS			

Legend:

The selection scale:

1	2	3	4	5	6	7

Positive selection   Purifying selection

Figure S2

Gene: *mapk3*

1	11	21	31	41
MAESGSSAAA	AGAAGSNSSA	AGPGGAVAPG	GPSGAAGSKP	GLESVKGQNF
51	61	71	81	91
DVGPRYTDLQ	YIGEGAYGMV	CSAFDNVNKI	RVAIKKISPF	EHQTYCQRTL
101	111	121	131	141
REIKILLRFH	HENIIGINDI	LRARHIDYMR	DVYIVQDLME	TDLYKLLKTQ
151	161	171	181	191
QLSNDHICYF	LYQILRGLKY	IHSANVLHRD	LKPSNLLINT	TCDLKICDFG
201	211	221	231	241
LARIADPEHD	HTGFLTEYVA	TRWYRAPEIM	LNSKGYTKSI	DIWSVGCILA
251	261	271	281	291
EMLSNRPIFP	GKHYLDQLNH	ILGVLGSPSQ	DDLNCIINMK	ARNYLQSLPQ
301	311	321	331	341
KPKIPWNKLF	PKADNKALDL	LDRMLTFNPL	KRINVEQALA	HPYLEQYYDP
351	361	371	381	391
SDEPVAEEPF	TFNMELDDLP	KEKLKELIFE	ETARFQANYQ	GS

Legend:

The selection scale:

1	2	3	4	5	6	7

Positive selection   Purifying selection

Figure S2

Gene: *med1*

1 11 21 31 41
MAAVSGVVIS GCSPARELGP GAPTVGPPTI GDRPRPEEGT EAEKQSRVGA
51 61 71 81 91
LLERLHAKHC ASRPWQETSK VVRQAMEKRN VLNAGGHQLL LTCLETLQRA
101 111 121 131 141
LKVSSLPAMT DRLESIARQN GLGSHLSPSG TECYITSDMF YVEVQLDSGG
151 161 171 181 191
QLVDVKVAHH GENPASCPEL VQHLRERNFE EFSKHLKGLV NLYKLPGDNK
201 211 221 231 241
LKTKMYLALQ SLEMDLTKMM HMFRLATNAN TVETILHGSV GLVTARSGGH
251 261 271 281 291
LVTLQCYVSP YDVFEEETGA LLNLTDSNVP RNLGVGVSVT IEGTSSVYKL
301 311 321 331 341
PIAPLITGTH PVDNKGTPSF SSVTNSNCVD LPACFFLKLR RPLPFSLSFI
351 361 371 381 391
HRMGNATGIP LFETAPPLAP LYELITQSQL QEEGGGALPP LAHNMRFYAS
401 411 421 431 441
LPGQQHCYFL NRDAPVQDGR CLQGALVTKV PFRHPAQVPA LLDIIRHQAA
451 461 471 481 491
YNTLIGSCVK RTVLKEDTPG LLQFEVCPLT DSSFSVSFQH PVNESLVCVV
501 511 521 531 541
MEVLDSRQVS CKLYKGLSDA LICTDDFITK VVQRCMSIPV TMRAIRRKAE
551 561 571 581 591
TIQADTPALS LIAETVEDMV KKNLPPAGSP GYGMGTGGGG NLMGIPGVGG
601 611 621 631 641
GNTPTGGGGS SAGAAGGGGG AGASFPGPIS TLFGMGLALK ERHEGRGPGG
651 661 671 681 691
EPMSQAGGAQ QQQAPLQQPA QQGHGDDFSK VTQNPILTSL LQITGNVGSS
701 711 721 731 741
PTQAPPAAGH QPHHTPPPAS SPASNTKNHP MLMNLLKDNP SQDFSSLYSS
751 761 771 781 791
SPLERQNSSG SPRTDIMGGG SCGGGGGVSG GKTKKKRQRN PDKAGGMGGA
801 811 821 831 841
MGLKPQGSLP LALQHHQHHQ LEDDFHRELF SMDVDASQNP IFDVNLPGDG
851 861 871 881 891
LDTPHSITPA PSQCGTPPTG PGVPYLSQGP PQSQSQQQQQ QQQVAPPQPP
901 911 921 931 941
PSGPSRMVRL SSSDSIGADI NEILSDIPEQ AGKMAAGGGG GHGPHHHGLG
951 961 971 981 991
GGDDGGALGT PIRDSSSSGQ GSAVFEADLF SAGSNENPFT DPVDLIADAA
1001 1011 1021 1031 1041

Figure S2

TAATPNSDSS SNNFFPDTVD FNPELLAGPG FPQAYFEDSS PSADGDLDLV

1051 1061 1071 1081 1091

KGFGGGSQQH TPAGTPQNPT PHGRSTPDPS MKDPFEMSMV FGGSGGGGKP

1101 1111 1121 1131 1141

LLGGPLPAPD LADSHSGGGQ SPLMMMGMGG GGGSDFKSDA KAKQQQLMRG

1151 1161 1171 1181 1191

GGKEGNGGGG GGGSGLGGAS GGSSEGKQGK RSRTPSSDGK SKDKPPKRKK

1201 1211 1221 1231 1241

LDPEGKSPSH SSGGRPYTPP SGIGGSGGGS MGAGGGSKSP GSSGRSQTPP

1251 1261 1271 1281 1291

GGATPPIPKI TIQIPKGTLT GGKTSSHGGY TSSSSAGGGG GGAAGGGGSK

1301 1311 1321 1331 1341

SHHSHSSSSS SSSGKMKGSK SDGSLGQGGG SKPGGGGGGC SGGGGGGGGG

1351 1361 1371 1381 1391

SQSKGSSQGM GGGKPGSSPI TKHGLAGAGV VAGGGVGGGA KMKPQGGKPP

1401 1411 1421 1431 1441

ASLINPNIKP NISPSHSRGV GAPDKLSSPM KLQQQQVPGT PPSSKAKSPM

1451 1461 1471 1481 1491

GSGGGGLGSG SKTSSGSGMG PQKQQMGGTS SSGSSSSSSS SSSSSSSSSS

1501 1511 1521 1531 1541

SSSSSSFSAG SQSQYGGGGG GGGGGGGNNN PNAKGKSPSR NKKPSLTAVI

1551 1561 1571 1581 1591

DKLKHGVGTG GHGGEYMSKR DKGDKESKSK VSMSGVGSGD KKALDSKSGV

1601 1611 1621 1631 1641

VSSTGVAKII ISKPDGGSPS IKAKVTLQKP GEGSGEAMRS QLSGPKAYGS

1651 1661 1671 1681 1691

PLFSASTPKH DRCSPSHGRS PGYTPLNPDS ESESGSSSVA EKSHQNSPSS

1701 1711 1721 1731 1741

DDDQALRPLQ PPHEYMSSMP ISSGEKHKKH KKEKKKQKER ERERERDRDK

1751 1761 1771 1781 1791

EKKKSSSSCS TSSSHPLKAD SWSRSPIAAP DPSLSLLGSD RPARPSPAYL

1801 1811

RTEDDDLMDS ALTGNLDSFK

Legend:

The selection scale:

1 2 3 4 5 6 7

Positive selection   Purifying selection

Figure S2

Gene: *med4*

1 XXXXXXXXXX 11 XXXXXXXXXX 21 XXXXXXXXXX 31 XXXXXXXXXX 41 XXXXXXXXXX

51 XXXXXXXXXX 61 XXXXXXXXXX 71 XXXXXXXXXX 81 XXXXXXXXXX 91 XXXXXXXXXX

101 XXXXXXMVHT 111 DVECALMVNV 121 FVSGSISSEE 131 IIKYAHRISA 141 SNAVCAPLNW

151 VPGDPRRPYP 161 TDLEMRSGML 171 GHMSNLPTNG 181 VNGHLPGDAL 191 AAGRLPGSLV

201 SPAAPTCLXX 211 XXXXXXXXXX 221 XXXXXXXXXX 231 XXXXXXXXXX 241 XXXXXXXXXX

251 XX

Legend:

The selection scale:

1	2	3	4	5	6	7

Positive selection Purifying selection

Figure S2

Gene: *med12*

1	11	21	31	41
MAAFGILSYE	HRPLKRPRLG	PPDVYPQDPK	QKEDELTALN	VKQGFNNQPA
51	61	71	81	91
VSGDEHGSAK	NVNFNPSKIS	SNFSSIIAEK	LRCNTFPDTG	KRKPQVNQKD
101	111	121	131	141
NFWLVTARSQ	SSINNWFTDL	AGTKPLTQLA	KKVPIFSKKE	EVFGYLAKYT
151	161	171	181	191
VPVMRAAWMI	KMTCAYYAAI	TETKVKKRHV	IDPCIEWTQI	ITKYLWEQLQ
201	211	221	231	241
KVAEFYRQSS	SQGCVSPLPS	TPVEVETAMK	QWEYNEKLAM	FMFQDGMLDR
251	261	271	281	291
HEFLTWVLEC	FEKIRPGEDE	LLKLLLPLML	QYSGEFVQSA	YLSRRLAYFC
301	311	321	331	341
TRRLNLLLSD	GTVGPGAGGH	QTHSITAQPG	NALPPTPTPQ	PAGGNQPQTP
351	361	371	381	391
FTDFYICPQH	RPLVFGLSCM	LQSIVLCCPS	ALVWHYSLTD	SRNKTGSPLD
401	411	421	431	441
LLPIAPSNLP	MPGGNTTFTQ	QVRAKVREIE	EQVKERGQAV	EFRWSFDKCQ
451	461	471	481	491
ETTAGFTIGR	VLHTLEVLDS	HSFEKSDFSN	SLDSLYNRIF	GSGQSKDGHE
501	511	521	531	541
MNPDDDAVVT	LLCEWAVSCK	RSGRHRAMVV	AKLLEKRQAE	IEAERCGESE
551	561	571	581	591
VVDEKGSVSS	GSLSAATLPV	FQDVLLQFLD	TQAPMLTEPG	NESERVEFSN
601	611	621	631	641
LVLLFCELIR	HDVFSHNIYM	CTLISRGDLA	SDSHLPRPRS	PSDEPSDESE
651	661	671	681	691
RKEQEAASSV	KMEDAGLSES	MEIDHNSSAI	FDDVMFSPPM	HCESKGSPSP
701	711	721	731	741
EKPAPEQDGK	STTKDKTLDP	AFPLVYELPR	HIQYATHFPI	PQEESASHEC
751	761	771	781	791
NQRLVVLYGV	GKQRDEARHT	IKKITKDILK	VLNRKSTAET	GGEEGQKRKR
801	811	821	831	841
TKPEAFPTAE	DIFSKFQHLS	HFDQHQVTSQ	VSRNVLEQIT	SFALGMSYHL
851	861	871	881	891
PLVQHIQFIF	DLMEYSLNIS	GLIDFAIQLL	NELSLVEAEL	LLKSSSLVGS
901	911	921	931	941
YTTGLCLCIV	AVLRRYHSCL	ILNPDQTAQV	FDGLRIVVKH	GVNPADCSSA
951	961	971	981	991
ERCILAYLYD	LYTSCSHLKS	KFGEIFSDFC	SKVKNSIYCN	IDPSDSNMLW
1001	1011	1021	1031	1041

Figure S2

DPVFMIETIA NPSAHNFNHS MVGKILNDSP ANRYSFVCNV LMDVCVDHRD
1051 1061 1071 1081 1091

PERVNDIGIL CAELTAYCRS LSAEWLGVLK ALCCSSNNGN CGFNDLLCNV
1101 1111 1121 1131 1141

DVSDLSFHDS LATFVAILIA RQCLLLEDLV RCVAIPSLLN AACSEQDSEP
1151 1161 1171 1181 1191

GARLTCRILL HLFKTPQRNP SPQDSSKSDK PAVGIRSSCD RHLLAASQNS
1201 1211 1221 1231 1241

IVVGAVFAVL KAVFMLGDAE LKGSGFPHPA GLDDTPEDDL GSKKSGGRAV
1251 1261 1271 1281 1291

SIETASLDVY AKYVLKSICQ QEWVGERCLK SLSEDSSALQ DPVLVNIQAQ
1301 1311 1321 1331 1341

RLLQLICYPH RQLDSEEGEN PQRQRIKRIL QNMDQWTMRQ SSLELQLMIK
1351 1361 1371 1381 1391

QSTNNELNSL LENIAKATIE VFQKSAEMIS SNPLGNGSAV SGPIQGPVTN
1401 1411 1421 1431 1441

SNNASKMKPV LSSSERSGVW LVAPLIAKLP TTVQGHVLKA AGEELEKGQH
1451 1461 1471 1481 1491

LGSSSRKERD RQKQKSMSLL SQQPFLSLVL TCLKGQDEQR EGLLTSLYSQ
1501 1511 1521 1531 1541

VQQIVTNWRE DQYQDDCKAK QMMHEALKLR LNLVGGMFDT VQRSTQQTTE
1551 1561 1571 1581 1591

WAVLLLDIIS SGTVDMQSNN ELFTTVLDML SVLINGTLAA DMSSISQGSM
1601 1611 1621 1631 1641

EENKRAYMNL VKKLRKELGD RQSESLEKVR QLLPLPKQTR DVITCEPQGS
1651 1661 1671 1681 1691

LIDTKGNKIA GFEKEGLQVS TKQKISPWDV FEGLKHSAPL SWGWFGTVRV
1701 1711 1721 1731 1741

DRKVTKFEEQ QRLLLYHTHL KPKPRSYYLE PLPLPPEEEE PPTPVAPEPE
1751 1761 1771 1781 1791

KKLAEAVKPE KSSAAVATDS SKKKSSKKKR NHSSSKTEDF ASTQRGVPYT
1801 1811 1821 1831 1841

AGMPPEMLHG QQGHPFNRMV YGPQSMGMYP QNQPLPPGGP RLDTTYRPTR
1851 1861 1871 1881 1891

TLPMRPNRPA AYPNMMTGMP GGVGNLITAL DQQAYRAYKP QPPIQGQILR
1901 1911 1921 1931 1941

QQLQAKLSQG MLGQQVRQMP PNPSYGTLQP TQVPPPQGYT SYGPHMGMQQ
1951 1961 1971 1981 1991

HPSQTGGMVP PAYANQPFQG SHPAPNPAMV DSLRQMQQRP SGYIHQQAPG
2001 2011 2021 2031 2041

YVHAMQNTPR FAHQQASIIQ GLSHMPGQGV HPGMRSNQML DFLRQQQALR
2051 2061 2071 2081 2091

VGIHSLFGLS GHQACLLLHW RAGAHVTVML LCLYQTPFDQ VSAAQPPAQA
2101 2111 2121 2131 2141

QPQALGMQPL PPQQPMFPRQ GMQQTQQQQQ TAALVRQLQQ QLSNTQPQQN
2151

Figure S2

T N S F Y

Legend:

The selection scale:

1	2	3	4	5	6	7

Positive selection Purifying selection

Figure S2

Gene: *med13*

1 11 21 31 41
MSSCFVPNGA SLEDCHSNLF CLADLTGIKW KKYVWQGPTS APILFPVTEE
51 61 71 81 91
DPILCSFSRC LKADVLSVWR RHQTAGRREL WLFWWGEDPN FAELIHHELT
101 111 121 131 141
GEEEGAWESG LSYECRTLLF KAIHNLLERC LMNRSFVRIG KWFVKPYEKD
151 161 171 181 191
EKPINKSEHL SCAFTFFLHG DSNVCTSVEI NQHQPIYHLS EGHLTLAQQS
201 211 221 231 241
NSPFQVILSP FGLNGTLTGQ SFKLSDPPTQ KLIEEWKQFY PISPSSKESS
251 261 271 281 291
EDKPDDMDWE DDSLAAVEVL VAGVRMVYPA CLVLVPQSDI PAVTPVGSSH
301 311 321 331 341
CTAVYSGVHQ VPASTRDPAI SSVTLTPPTS PEEAQTMDSQ SAQKWVKFSS
351 361 371 381 391
VSDGFSTDST SHHGGKIPRK LASQVVDRVW QECNINRAQN KRKFSATTNG
401 411 421 431 441
TCEEESTDKA GSWDFVDPTQ RSNCNCSRHK NLKQRAGSTP GQPQAAGQPP
451 461 471 481 491
QPPPKHKAVE KPEKGEKQQK RPLTPFHHRT SICEDVSMEP DATGQRLGMR
501 511 521 531 541
AQDGGEPLKN PATPHSQHFY QPAPEPCLVP QKPAEDPRLD PLAQPFPPAY
551 561 571 581 591
SEAMEPTVYV GAAVSPDEDN AHAPWKFFIL PRKKDSDFAP PQLPGDKLRD
601 611 621 631 641
DLPGTGSQES VVSVTELMAT SKNPLKVSEG LVQMYIQRRT QYLSAAISDS
651 661 671 681 691
DHEPELDPYA FVDGDVEFSF RDKKDKQGGE REAGKKHKAD DGSNGGPVPA
701 711 721 731 741
EGEDAMSLFS PSAKTEDAQR SAAHNRTAST SLMHENDLAV SINDLDNLFN
751 761 771 781 791
SDEDELAPGA RRAVNGTDEK FGNKEAKPAS LDQLSCISSA DLHQMFPTPP
801 811 821 831 841
SLEQHIMGYS PMNMNSKEYG SLDNASGMTV LDGSSVLGGQ FKIEVEEGFC
851 861 871 881 891
SPKPSEIKDF SFVYKPEGCQ AFVGCSMFAP LKTLPSQCLP PIKLPEECLY
901 911 921 931 941
RPSWTVGKLE LLNPVPALTF LNKDGNIPSV GSAMDQDYIQ TYTPQTHTPF
951 961 971 981 991
MSNSAPPSNG GTGILPSPAT PRFSAPTPRT PRTPRTPRGP ASVQGSLKYE
1001 1011 1021 1031 1041

Figure S2

NSDLYSPAST PSTCRPLNSV EPATVPSIPE AHSLYVNLIL SESVMNLFKD

1051 1061 1071 1081 1091

CNFDSCCVCV CNMNIKGADV GVYIPDPNCE VQYSCTCGFS AVMNRRYGNG

1101 1111 1121 1131 1141

SGLFLEDELD IIGRGSDASR EVEKRFEAVH GSGASAERAG SLRDQVPDDL

1151 1161 1171 1181 1191

ILLLQDQCTN PFSPITAPEP DSAPARGPGA PPPCVRVEER DCYSDCYLAL

1201 1211 1221 1231 1241

EHGRQFMDNM SGGKVDEALV KSTCLHHWAK RNAVDVSMLC SQDVLRVLLS

1251 1261 1271 1281 1291

LQPVLQDAIQ KKRTVRSWGV QGPLTWQQFH KMAGRGSYGT DESPEPLPIP

1301 1311 1321 1331 1341

TFLVGYEYDF VVLSPFGLPY WEKLLLDPFG SQRDVGYLVL CPENEALLSG

1351 1361 1371 1381 1391

AKGFFRDLTA VYESCRLGQH RPISKTHPDG IVRVGTAAAK KLADQPVSDW

1401 1411 1421 1431 1441

FLKAASSNSD SFAKLKLYAQ VCRHDLAPYL AAQSLDSSLL VQPSPPPASS

1451 1461 1471 1481 1491

QSSSTPAPVV SAPGSQGTLT SSGTMTATAG TAIPPSASGT PSSTLPSSGP

1501 1511 1521 1531 1541

VGMGSSLQSS KPSSFPPFGN MGAQSGSSQS GTLGQQAGTQ APGITGENVP

1551 1561 1571 1581 1591

GAAQTQGPAE PPESTMEREK VGVPTDGDSH AITYPPAIVV YIVDPFTYEE

1601 1611 1621 1631 1641

MEGGAQSSSV WTLGLLRCYL EMLQSLPPHI RNAVSVQIIP CQYLLQPVKN

1651 1661 1671 1681 1691

DERHIYAQHL KSLAFSVFSQ CRRPLPISTN VKTLTGFGPG LALDTALKSP

1701 1711 1721 1731 1741

ERPECLRLYT PPFILAPVKD KQTELGETFG EASQKYNVLF VGYCLSHDQK

1751 1761 1771 1781 1791

WLLATCTDLY GELLETCIIN IDVPNRARRK KGSARRLGLQ KLWEWCLGLV

1801 1811 1821 1831 1841

QMTSLPWRVV IGRLGRIGHG ELRDWSILLS RRNLQSLSRR LKEMCRMCGI

1851 1861 1871 1881 1891

SAADTPSILS ACLVAMEPQG SFVIMPDSVS TGSVFGRSTT LNMQTSQLNT

1901 1911 1921 1931 1941

PQDTSCTHIL VFPTSAFVQV ANSNYTTENI DIAFNPINDG SDGMGIFDLL

1951 1961 1971 1981 1991

DTGEDLVDPD IINILPASPT TSPVHSPGSH YPHGGDGSKG QSTDRLESHE

2001 2011 2021 2031 2041

EAPNILQQPL ALGYFVSTAK AGPLPDWFWA ACPQAQNQCP LFLKASLHLH

2051 2061 2071 2081 2091

VSSVQSDELL HSKHSHPLDS NQTSDVLRFV LEQYNALSWL TCDPATQDRR

2101 2111

SCLPIHFVVL NQMYNFIMNM L

Figure S2

<u>Legend:</u>

The selection scale:

1	2	3	4	5	6	7

Positive selection Purifying selection

Figure S2

Gene: *med14*

```
1          11         21         31         41
MAPVQIGSDG QLVPAGGPTS ALQPPPPGAP SGTPATHGVR LSVLIEFLLQ
51         61         71         81         91
RTYHEITLLA ELLPRKTDME RKIEIVQFAS RTRQLFVRLL ALVKWASNAG
101        111        121        131        141
KVEKCAMISS FLDQQAFLFV DTADRLASLA RDALVHARLP SFAIPFAIDV
151        161        171        181        191
LTTGSYPRLP TCIRDKIIPP DPITKAEKQA TLSQLNQILR HRLVTTDLPP
201        211        221        231        241
QLANLTVANG RVKFRVEGEF EATLTVMGDD PDIPWRLLKL EILVEDKETG
251        261        271        281        291
DGRALVHSMQ VSFIHELVQS RLFADEKPLQ DMYNCLHSFC LSLQLEVLHS
301        311        321        331        341
QTLMLIRERW GDLVQVERYI PAKCLTLAVW NQQVLGRKTG TASVHKVHIK
351        361        371        381        391
IDETDGSKPL QISHEPLLPA CDSKLMERAM KIDHLSVEKL LIDSVHARSH
401        411        421        431        441
QKLQELKAIL KNYNPSDNSF IETALPTLVI PILEPCGRSE CLHIFVDLHS
451        461        471        481        491
GTFQPMLYGT DQSTLDEMEK SINDDMKRII PWLQQLKFWL GEQRCRQSVK
501        511        521        531        541
HLPTVCSKTL HLSNAASHPA GSLSKHRLFI RLTRLPQYYI VAEMFDVPGC
551        561        571        581        591
PTELEYKYYF LSVTMLEGDE GPPSAQLLQQ FKPNLEELVL DATPGRGAKS
601        611        621        631        641
GTKRKLSGDQ GAIEPKKPKR AGEMCAFNKV LAHLVAMCDT NMPFIGLRCE
651        661        671        681        691
LSTMEIPHQG VQVEGDGCSH AIRILKIPPS KGVSEETRRV LERSLLDCTF
701        711        721        731        741
RLQGRNNRTW VAELVFANCP LTSTSSKEQA STRHVYLTYE NPLSEPVGGR
751        761        771        781        791
KVVEMFLNDW CSIAQLYECV LDFARSLGDM PSYLSLFSEI RLYNYRKLVL
801        811        821        831        841
GYGSTKGSSI TIQWNSVTQK FHISLGTVGP NSGCSNCHNI ILHQLQEMFN
851        861        871        881        891
KTPSVVQLLQ VLFDTQGPLN AINKLPTVPM LGLTQRTNTA YQCFSILPQS
901        911        921        931        941
PTHIRLAFRN MYCIDIYCRS RGVVAIRDGA YSLFDNTKIV EGFYPAPGLK
951        961        971        981        991
TFLNMFVDSN QDARRRSVNE DDNPPSPVGV DVMDALMSQL QPPPGQPQPF
1001       1011       1021       1031       1041
```

Figure S2

```
GKQAGGTGVY PITSPPTSYH TGVTPSPSMM PTQSPGTIHA ASSPSGALRA
1051       1061       1071       1081       1091
PSPASFGPTP SPSSLGISMG QTSNFASPHG TLDPSSPYAL MSPGQRSSNW
1101       1111       1121       1131       1141
PGSPQVSGPS PAARMPGMSP ANPSLHSPIP DASHSPRAGT SSQAMPTNMP
1151       1161       1171       1181       1191
PPRKLPQRSW AASIPTILTH SALHVLLLPS PTPCLVPGLA GSYLCSPLER
1201       1211       1221       1231       1241
FLGSVIMRRH LQRIIQQEPN LQIVNSNEPG VIMFKTDVLK CRVALNPKTY
1251       1261       1271       1281       1291
QTLQLKVTPD NTGPWSQEEL QVLEKFFETR VAGPPFKYNT LNAFTKLLGA
1301       1311       1321       1331       1341
PTHILRDCVR IMKLELFPDQ ATQLKWNVQF CLTIPPSAPP IAPPGTIAVV
1351       1361       1371       1381       1391
LKSKMLFFLQ LTQRLPVPQE PISIIVPIVY DMATGLTQQA DIPRQPSSSG
1401       1411       1421       1431       1441
AAALMVSSIL KRFNEMHPPR QSECTIFASV HELMANLTLP PGGRP
```

Legend:

The selection scale:

1 2 3 4 5 6 7

Positive selection   Purifying selection

Figure S2

Gene: *med16*

1	11	21	31	41
MDIAYVCEWE	KRPKSNHCPS	IPLVCAWSCR	NLLAFTTDLK	NEEEEKDLTH
51	61	71	81	91
MIHIIDSEHP	WDVYSINSGH	TEVISCLEWD	QSGSRLLSAD	GDGQIKCWAM
101	111	121	131	141
TEHLVNSWES	TLGSAVEGDP	IVALSWLHNG	VKLALHVEKS	GSTNFGEKFS
151	161	171	181	191
RVKFSPSLTL	FGGKPMEGWM	AVTVSGLVTV	SLLKPNGALL	TASESLCRLR
201	211	221	231	241
GRVALADIAF	TGGGNIVVAA	TDGSSSAPVQ	FYKVCVSVVN	EKCRIDTELL
251	261	271	281	291
PSLFMRCTTD	PARKDKYPAV	THLKFLTREN	SEQVLLCASS	QLGSIVECWS
301	311	321	331	341
LRKEGLPVNN	IFQHRSPVVG	EKQPMILKWR	ILSATNDLDR	VSAVALPKLP
351	361	371	381	391
ISISNTDLKV	ASDTKFCPGL	GLALAFHDGS	IHILHRLSLH	TMGVFYGSSS
401	411	421	431	441
SNPQRVGEEP	ALKRQRGGGP	TTHFKALQFS	CTSLALVGVD	NHGKLHMLRV
451	461	471	481	491
SPSMGQMLDI	NTLLRHLLFL	LEYCMVTGYD	WWDVLLHVQP	NMVHNLVEKL
501	511	521	531	541
HEEYMRQNQA	LQQVLSTRVV	AMKASLCKLS	SATAPRACDF	HAKLLLIAIS
551	561	571	581	591
STLKSLLRPH	LLNTPDKSPG	DRLAEICAKN	TDTDIDKVMI	NLKTEEFVLD
601	611	621	631	641
ASTLQSLQQL	IQWVGDFVLY	LLANLPNQGS	IVRPGFGFLR	DGASLGLLRE
651	661	671	681	691
MMVVIRIWGL	LKPGCLPIYT	ATSDNQDSMF	LLFRLLTKLW	LCSRDESHPQ
701	711	721	731	741
EPDEPLIDEC	CLLPSQLLVP	AMDWLPVSDG	IICKLQGKHP	LRLQFGKPYS
751	761	771	781	791
LPGLNSGTQV	EIFTRTPGSQ	RMDNLRCLHM	GVCPTEDSKA	CTRCGCVTML
801	811	821	831	
RSPNKTTAVK	QWEQRWIKNC	LCGGLWRRIP	TTIS	

Legend:

The selection scale:

1 2 3 4 5 6 7

Positive selection Purifying selection

Figure S2

Gene: *med17*

```
1          11         21         31         41
MSGGPAVRVS IESACEKQVQ EVSLDGIETY VQPLSMSQNL AKLAQRIDFG
51         61         71         81         91
QGSDSEDEGG EGESRDQEWG KQEAEEDEAT VKFQPSLWPW DSVRNNLRSA
101        111        121        131        141
LTEMCVLYDV LSVVKEKKYM TLDPVSHDVT AGKTPQVLQL IGKKKSLATA
151        161        171        181        191
AQLLLKGAEK LSKSVAENQE NRRQRDFNSE LLRLRSQWKL RKVGDKILGD
201        211        221        231        241
LSYKSAGSLF PHHGTFEVIK NTDIDLDKKI PDDYCPLDVQ IPSDLEGSAY
251        261        271        281        291
IKVSIQKQAP DIGDLGTVSL FRRQPKAKSG SQPWHAKLEA AQNVLLCKEI
301        311        321        331        341
FAQLSREAVQ IKSQIPHIVV KNQIISQPFP GLQLSISLCH STGEKKNQKA
351        361        371        381        391
SPEKSKPDDH LYVLEHNLHQ LMREFHKQTL SSAVMPHPAS APFGHKRLRL
401        411        421        431        441
AGPMAFDKAE IASLQQNEGL LEKIIKQAKH IFLRRRTART IDSLASRIED
451        461        471        481        491
PQIQAHWSNI NDVYESSVKV LITSQGYEQI CKSIQLQLNI GVEQIRVVHR
501        511        521        531        541
DGRVITLSHQ EQELQDFLLS QMSQHQVHAV QQLAKVMGWH VLSFSNHVGL
551        561        571        581        591
GSVESIGNAS AITVASPNGE FAISVRNGPE SGCKVIVQFP RSQCKDLPKS
601        611        621        631        641
DVIQDNKWNH LRGPSKEVHW NKMEGRNFVY KMELLMAALT PCP
```

Legend:

The selection scale:

1	2	3	4	5	6	7

Positive selection — Purifying selection

Figure S2

Gene: *med24*

1	11	21	31	41
MKVVNLKQAI	LQAWKERWSD	FQWAINIKKN	FPKGATWDYL	NLAEALLEQA
51	61	71	81	91
MIGLSPNPLI	LSYLKYAISS	QMVSYSSILT	AVSKFDDFSR	ELCVKSLLEI
101	111	121	131	141
MDMFSHQLSC	HGKAEECIGL	CRSLLAALVW	LLQGCAWYSA	RLREQGEQAG
151	161	171	181	191
EASLRACVDR	LEGLLRGTKN	RALIHIARLE	EQASWTNVEQ	AVIRVTENLN
201	211	221	231	241
SLTNQVLRSK	LEECLSLVKS	IPLMLVVQAE	PPQRTSFPSV	HTLVMLEGTM
251	261	271	281	291
NLTGETQPLV	EQLMMIKRMQ	RIPSPLFVLE	IWKACFTGLI	ESPEGTEELK
301	311	321	331	341
WTAFTFLKIP	QVLLRLKKYP	QGEKDFMEDV	NIAFEYLLKL	TPLLDKADQR
351	361	371	381	391
CNCDCIELLL	QECRKLGLLS	EANTSNLSTK	RAVDREHAPR	LKTAENANIQ
401	411	421	431	441
PNPGLILRAE	PTVTNILKTV	DADHSKSPEG	LLGVLGHMLS	GKSLDLLLAA
451	461	471	481	491
AAATGKLKSF	ARKFIKLNEF	PKHISGEGSK	PASVRALLFD	ISFLMLCHVV
501	511	521	531	541
QTYGSEVILS	DPSPSGETPF	FETWLQTCMP	EDGKILNPDH	PCFRPEPGKV
551	561	571	581	591
ENLVALLNNS	SEMKLVQMKW	HEICLSTPAA	ILEVLNAWEN	GVLTVESVQQ
601	611	621	631	641
KITDNIKGKV	CSMAICAVAW	LVAHVRMLGL	DEREKPQTMI	RQLMTPLYGH
651	661	671	681	691
SAENTLQFYN	ERVVIMSSIL	EHMCADVFQQ	TGVVLRPPME	GLEPVPYRNL
701	711	721	731	741
LAPREPIRAA	LSHQFSEVLQ	RGWVDSQALH	LFESLLHMGG	VFWFTNNLVK
751	761	771	781	791
ELLRGTRQEW	VMCAVELLYS	IFCLDMQQIT	LTLLGQILPS	LLTDSARWHS
801	811	821	831	841
LADPPGKALA	KLAVWCALSS	YSTHHKGQAS	ARQRKRQRED	IEDYNSLFPL
851	861	871	881	891
DDTQPSKLMR	LLSSNEDDPV	ILSSPGDRSM	SSSLSASQLH	TVNMRDPLNR
901	911	921	931	941
VLANLFLLVS	SILSSKTAGP	HTQFVQSFME	ECVECLEQGS	RGSILQFMPF
951	961	971	981	
TMVSELVKLT	ALAKPKVVLA	ITDLSLPLGR	RVAAKAIAAL	

Figure S2

Legend:

The selection scale:

1	2	3	4	5	6	7

Positive selection Purifying selection

Figure S2

Gene: *med30*

```
1          11         21         31         41
MATPPVAGAG MPAGAFGGQQ AQAAREVNTA SLCRIGQETV QDIVFRTMEI
51         61         71         81         91
FQLLRNMQLP NGVTYHASTH QDRLGKLQEH LRQLSVLFRK LRLVYDKCNE
101        111        121        131        141
NCAGLDPVPA EQLIPYVEDD GSKHDDRSTN LARYATEERR EVLEVNQKLK
151        161        171
QKNQQLKQIM DQLRNLIWEI NSMLAVRN
```

Legend:

The selection scale:

1	2	3	4	5	6	7

Positive selection   Purifying selection

Figure S2

Gene: *ncoa1*

1 11 21 31 41
MSGLGESALD PTNLDSLKRK GSPCDTSGQS VEKRRRELEC RYIEELAELL
51 61 71 81 91
SANMGDIDSL SVKPDKCHIL KSTVDQIQQI KRREQEKAAA ALMSPDDEVQ
101 111 121 131 141
KSDISSSSQG MIEKEALCPL LLEALDGFFF VVNREGRIVF VSENVTNYLG
151 161 171 181 191
YPQEELMSSS VYSILHVGDH NEFVRNLLPK SLVNGVPWPQ ESTGRNSHTF
201 211 221 231 241
NCRMLKRPPD EVDSENQEAR QQYEIMQCFT VSQPRAMHEE GEDLQSCLIC
251 261 271 281 291
IACRLPRPQL PVSTESFITR QDPTGKIISI ETSALRATGR PGWEDLVRKC
301 311 321 331 341
IYAFFQPQGK EPSYAKQLLQ EVISHGTAIS PVYRFTLNDG TTLSAQTRCK
351 361 371 381 391
LCYPPNQDMQ PFIMGIHTID REHNTASSQE NTNTGPLSTH GSQSPAQPTR
401 411 421 431 441
SPAVQPSMEL GQTAGMGVHL NNGNSSGTGP ATPTSHPPGY LTPSRMGTQQ
451 461 471 481 491
LNSPSPLGSP LGTTPTSFMS PRPPRGSPGL GGSPRVSGNP FSPSTPSLHS
501 511 521 531 541
PAGAPSGSGN LSRQQSGCHG ECAGTPLSFS TPSPVPQRQT STPASSPVRP
551 561 571 581 591
PLAKPAEGLP ESREDHTKTP QQLSNAKLGQ LLDGCGGRPS ETDTPPSLPA
601 611 621 631 641
PCPASHSSLT ERHKILHRLL QDNSPTEPTE GGRKDMEIKK EPSASSPSSR
651 661 671 681 691
EPQDHQLLRF LLDTDDKDLG GLPPSSALSL QTVRVKAGKT PEAESSASPK
701 711 721 731 741
PNDRPRNQVA LFPSMPELKH HSGFLRLEKK IQSVISFKYP LFVILQVSDS
751 761 771 781 791
TYHLFQPSLQ LNPATLLYYF FTYLRIREVL AAADLDTVSQ LLHTLAGGPG
801 811 821 831 841
VKLPEEQGDS PQPGESSLPR GVSVKQEPSS TPGRGFSDGP RLQSQSPFEF
851 861 871 881 891
CSPSTPSQGQ GDHFLSPKGS SPFRESGHVN TFRTDTGLPK MELTGSQQFH
901 911 921 931 941
PPPMAEPMPF EGNMGSVNDS ALTVPPEQCI PCPLDEMLCP PTTVEGRNDE
951 961 971 981 991
KALLDQLVSF LSGTDESELA ALDRALGIDK LVQGCCLDSL SQQFPAQAPM
1001 1011 1021 1031 1041

Figure S2

LMDPKPPGYT SQFPPGSPQF SSAMGRAQGL GFGGPRGAFP GGIGMGPRPP
1051 1061 1071 1081 1091
LARPPGLPNQ LRLQLQQRLQ GQQQLMQQNR QAQMGQFPGA VPVAMGMRPS
1101 1111 1121 1131 1141
MQQPQLPSQQ PPLNAQMLAQ RQRELYSIQH RQRQLLQQKA MLMRQQGFAG
1151 1161 1171 1181 1191
GPMGVARIPK GPQQQQQFTF PPGYSPAAGN PPNSPSPFTP MGGPPDPTLP
1201 1211 1221 1231 1241
ARGPMANQGL MGNVQGQFGG AMNSPVQPSI FQQFGGPGMV QQGEPPFPPA
1251 1261 1271 1281 1291
LSPTSPLLSP QISTSQSPML QQTQPPPGYQ SPDLKNWQQG SMGNNSMYNQ
1301 1311 1321 1331 1341
SGQTPPQAFG QQGVYNNMSI TVSMAGGTGG VGSIPSMGPP VGMGNSNMGS
1351 1361 1371 1381 1391
VSSMCNDQVQ QVQVFADVQC TVNLVGSDPY LNQSGPLGSQ KGGAGAQTTP
1401 1411
SQQKSLLQQL LTE

Legend:

The selection scale:

1 2 3 4 5 6 7

Positive selection   Purifying selection

Figure S2

Gene: *ncoa3*

```
1          11         21         31         41
MSGLGDNSLD PLCSDRKRKL STCDTPGLGC DKRRREQESK YIEELAELIS
51         61         71         81         91
ANLSDIDNFN VKPDKCAILK ETVRQIRQIK EQGKASSNDD DVQKADVSST
101        111        121        131        141
GQGVIDKDHL GPLLLQALDG FLFVVNRDGS IVFVSDNVTQ YLQFKQEELI
151        161        171        181        191
NTSVYNILHE EDREEFHKNL PKSNMNGVSW ATEAPRQKSH TFNCRMLMKY
201        211        221        231        241
GHSPPEEGAG SPRYETMQCF ALTQPKAMME EGDDLQSCMI CVARRITAVE
251        261        271        281        291
RTESFITKHE LSGKLIQIDH SSLRSTMRPG WEDLLRRCIQ MFMHHSEGQP
301        311        321        331        341
WSHKRHYQEA FMQGHAETPL YRFSLSDGTP VTAQTKSKLY RNPMTNEPQG
351        361        371        381        391
FISTHLLQRE QNGYRANQGP MIQGMRPMGN STPNASMNMP PGPGMGMGGN
401        411        421        431        441
RGFSMNESGH MGQMGGSMYG ATNRMMQMNP MSQMSQMGQM SQMNHPGPGM
451        461        471        481        491
QQQPPYQGSG YGLGMNSPSQ GSPGMNVPQQ NLMVSPRTRG SPKMVPSQFS
501        511        521        531        541
PGGMHSPMGP GSAGGAGGGG NSNFSSSSLN ALQAISEGVG NPMPSSLSSP
551        561        571        581        591
AHKPDSSPSI NSSQQQQQQN QQCKAGRVDS PSPAGMYVPG GDHHHHLHHH
601        611        621        631        641
HHTPTESATD RPDSQASLRV TKEGSEVGTG GAEPQRRLSD SKGNKKLLQL
651        661        671        681        691
LTSPTDDLGM VAGGVPPTGA STPSTLEPKE PAGCVTSPSS TGVSSSSSSS
701        711        721        731        741
SSAAQPPGGV SSSSSSAHHA ASLQEKHKIL HKLLQNGNSP DEVAKITAEA
751        761        771        781        791
TGKETSSHEA GVADLGTIGT AGTGGGGVGG SGVPDIKQEQ PSPKKTHALL
801        811        821        831        841
HYLLNNDPKE PADIKPKLEE LEGKTQQNAC SSSGLMPSTP ENGENKIKTE
851        861        871        881        891
QPDELHDTLE TILGGFRNSS SSFYQESGVG AGSDVANKQP ACPDDAMHGL
901        911        921        931        941
RSPAGLRSPE LGPRGPFQRA VSVDGKPPVG ANSLGRRSAP CPMLVKQESM
951        961        971        981        991
DNQRMMGGPE NFPGNMGMVN RGLGVPQRSP MGGSGEWGIQ RSNASPVGTS
1001       1011       1021       1031       1041
```


T. Lorin, W. Salzburger & A. Böhne

Figure S2

SHPMVRPGMD YSSKPMMGGP MVGRSNSVPG NRSMLQQQLM DMGPNDINMG

1051 1061 1071 1081 1091

MTPFNQQGPP NQSPSWPENV MPIEHGPQNS QSRRQFANPL DDLLVPPATS

1101 1111 1121 1131 1141

EGQSDERALL DQLDSLLNNT DVMALEEIDR ALGIPDLVSQ GQGVDPQSEP

1151 1161 1171 1181 1191

FPGQDPALVM DQKPPMYSQG YPGPPGMAMQ PAYSSPMQGQ PPGFNPMMNQ

1201 1211 1221 1231 1241

MSQQGNFPMA GMHPRPNMIR PRMMNALKPL RQQLVQRLQG QQFMNQSRQG

1251 1261 1271 1281 1291

LGMKMDNTTA GNPGLRPALQ APMGGQPGFL NAQMMAQRNR EMLNHQLRRQ

1301 1311 1321 1331 1341

RMMMLMQQQQ QAAAGGFSPP PNVTAPAGMD TPMGGPPMTQ PGPQQFSYAA

1351 1361 1371 1381 1391

NYGINQQGDP SFVRAAGSPP NSIMSARLGP PQNPMMQQHP QGGPMYPTAD

1401 1411 1421 1431 1441

MKSWPQGGMP RSSSYPQQQF PQQGNPGSYG NMMMNGSLPV NGNGGHMGQM

1451 1461 1471

PGQMNMNPMA MGRMPMGPEQ KYC

Legend:

The selection scale:

1	2	3	4	5	6	7

Positive selection Purifying selection

Figure S2

Gene: *ncoa4*

1	11	21	31	41
MSSAVEDRDR	DSLRRCLQAR	CQLEASISGV	TRAELQLRDN	SREVKAQIHS
51	61	71	81	91
CISRHLEVLR	SREVWLLEQI	EIVQHLKEEA	LQQQHHQLHC	LLGQLNFLIY
101	111	121	131	141
QLENNNSNNL	ANQITSCLEK	LSSLNLKPEE	TPEMSFQADI	RSLRQAITSF
151	161	171	181	191
GTIETKQLSY	YLISPPSKSS	LDDVWLFQNC	PLAYTQPKVE	SKLWTELDNW
201	211	221	231	241
ILKSSSGSSP	QSGCQLSFNL	DDWLYKPKEK	SQVSCPFAQF	EFDKMCTKTD
251	261	271	281	291
VEAWVLKDRV	PPRERSVSET	SSSTMMSIEK	IEDADLNLSE	GEEGQETEDL
301	311	321	331	341
KDWLVTSPTE	EKEELAENKS	HADDKPKTMF	WPFSEPFRAS	DWLVKYDDSC
351	361	371	381	391
GACRGQSKAV	EIENLGALKC	LKHSPSPSPA	ATSSTETVRV	EQVCKANEPC
401	411	421	431	441
GSFLDCVCEE	NCGREALCGL	LFKKEGRDKN	GMPLDKNKVL	EESYVNDIDR
451	461	471	481	491
WLHPRKKEVP	ASSFPSYGLT	LQPQEQEKTT	QEEPELKCKD	PFKVSFNPED
501	511	521	531	541
WLMPRRKPSL	PAPCTNTEED	KWLLRKRAPV	AEQNGPTSFS	DLFACMTLKG
551	561			
DMEKWLYKAP	IQHILPDH			

Legend:

The selection scale:

1 2 3 4 5 6 7

Positive selection Purifying selection

 T. Lorin, W. Salzburger & A. Böhne

Figure S2

Gene: *nkx3.1*

```
1          11         21         31         41
MSASVKPLTS FLIQDILAFK EESRLSTPRY ACAKEPRERD KSPDSISAGR
51         61         71         81         91
DTRHARETGS HDKNTSTDLK AVGNGEETPQ SSSSGGKQKR SRAAFTHLQV
101        111        121        131        141
LELEKKFSHQ KYLSAPERAH LAHSLRLTET QVKIWFQNRR YKTKRKQLAT
151        161        171        181        191
EYGKDPFKRA TGLPCVGTEE DLIRASLFAT MYKTYHDQQP CLYGLNVWNS
201
AV
```

Legend:

The selection scale:

1	2	3	4	5	6	7

Positive selection — Purifying selection

Figure S2

Gene: *nrip1*

```
1          11         21         31         41
MTHGEEPGSE MHQDSAVLTY LEGLLMHQVS GRQSAAATRR SDAGHSERNQ
51         61         71         81         91
NNKTVGSHQL PNHSSNQEDR SVPLGRATQH LKKARLLRSS EAWNGPETQQ
101        111        121        131        141
LPASVVNLNG QNRDLLTGAL DSSPKCKAES TLLASLLQSF SSRLQSVALS
151        161        171        181        191
QQIMQNLKQQ DNLQSDKSTQ VEDEALRCYG SASSRLKGLL KKNKMQNHNS
201        211        221        231        241
VPYQRRNSQE RFSDSPEASQ SSVQPAARDS ISCAARLKAV ASIVKNRSSP
251        261        271        281        291
TSSPKPSVAC SQLALLLSSE AHLQQYSREQ ALKAQLSGRS ASERLAAMAT
301        311        321        331        341
QQTQDIKQSS MGQHQVSADI VNPLNGQNGT LPQTVDSSKP SPSPIQGQSR
351        361        371        381        391
VNSSQRSLHS FRDKHSFDRH SSRPSPNCSS LLLHLLNNHN TQKYTNGNSL
401        411        421        431        441
MEEDYSVFPN HSSPLRSESE YSNLENSLTK DNSDAESSHS SCSPIDLSVK
451        461        471        481        491
GRASGSGLGS SSSLDKLTET LISNWNPETS SHKVTEARES ENSSVIKPHH
501        511        521        531        541
KVTLLQLLLG HKNNEKVNKN SDNPDLQSCT TSKPNCLPTG RVTPSSRFEE
551        561        571        581        591
TRTRNSPDAL CFRKLQSLPV FSQEQDTNGS ASPYSLSLSP QVQAIPLDLC
601        611        621        631        641
KAKSHSNENV EESSFSASKL LQNLAQCGLQ KSIPSPPVET SVSPGIRQTY
651        661        671        681        691
EPRTDKPVAL LERLNAPLTK NKTTVLEEPL VNSMKLPYVM DPSPSVSEIE
701        711        721        731        741
NLLERRTVLQ LLLGATTSKE KASGKRKRPV DKGDSLDKHS DPSPGSGNSY
751        761        771        781        791
EPTLDIKIKT EPRDEVHLSN TNGEEKRSQV EERLNGGNNP HSSNQKDIKS
801        811        821        831        841
EVLSAEAIPK DGLLSQLLKH PPSTYQVKNQ DVCTISGKEG LSLNQGPAIP
851        861        871        881        891
KKRKLCMGMD ETLNTEPCIR AVSAVQRDGS SEPSGSRTSD GRNVHNEVNR
901        911        921        931        941
LEADSLLQVG CPVSESLPKD GKGFNVLKQL LLSDNCLKDI SLPRSATSPS
951        961        971        981        991
IIQANCKING NIPSKSGYNH DFAVLQHNSS PQGPVSLDFK PLSAASERSK
1001       1011       1021       1031       1041
```

Figure S2

PPGSPWGYHA ARQDSPKLNL IPVKRELEGP IQWVISGEEK QDPSIDSPRL

1051 1061 1071 1081 1091

TKSNPILYYM LQKGNSHIGK LRKAEAPECE MQVKVKEEPM SELDAYSHRL

1101 1111 1121

RLKHHHQLSN EKQGNETESF NRTVEKC

Legend:

The selection scale:

1	2	3	4	5	6	7

Positive selection Purifying selection

Figure S2

Gene: *pias1*

1	11	21	31	41
MVMSLRVSEL	QVLLGYAGRN	KHGRKHELLT	KALHLLKAGC	SPAVQMKIKE
51	61	71	81	91
LYRRRFPTKM	VTPAELSLPS	VHSTTAPLPA	GLPQLSFDGH	AASSPLLPVS
101	111	121	131	141
LLGPKHELDL	PHLPSALHPV	HPDVKLQKLP	FYDMLDELIK	PTSLASDNSQ
151	161	171	181	191
RFQETCFAFA	LTPQQVQQIS	SSIFLSQQIR	HITFQDTLKF	CLSETSCPQE
201	211	221	231	241
DHFPPNLCVK	VNGKPCNLPG	YLPPTKNGVE	PKRPSRPINI	TSLVRLSTTV
251	261	271	281	291
PNTIVVSWTA	EIGRSYSMAV	YLVKQQSSSV	LLQRLRSKGI	RNPDHSRALI
301	311	321	331	341
KEKLTADPDS	EIATTSLRVS	LLCPLGKMRL	MIPCRALTCS	HLQCFDATLY
351	361	371	381	391
IQMNEKKPTW	VCPVCDKKAP	YEHLIIDGLF	MEILNSCVDC	DEIQFKEDGS
401	411	421	431	441
WAPMRSKKEV	QQVNSYNGVE	GACRTAIPEH	RHSSSSNSKK	VEVIDLTLDS
451	461	471	481	491
SSDEEEDEEP	PPKRTCPSLS	PASPPVNKGV	LNLHHQASPV	ARAPSMPPVD
501	511	521	531	541
TNYIPPPPPL	IQDYRHSYHT	LPYELQGLDF	FPFLQGDNQH	YSTNLLVAAA
551	561	571	581	591
AAASASDEHD	MLHPSLNRFL	PYNSSQMYLE	QPGTPVSSAL	VPANGGGSSS
601	611	621	631	641
SSSSSLVSSS	SLRESHGHPG	PTRATAEAAA	AIYGSMPDVI	SLD

Legend:

The selection scale:

1	2	3	4	5	6	7

Positive selection   Purifying selection

Figure S2

Gene: *pias2*

1	11	21	31	41
MTLTQPAPPI	PPVHPDVQLK	SLPFYDVLDV	LIKPSSLGAS	SIQRFQEKYF
51	61	71	81	91
IFALTPQQVR	EVCISRDFLP	GGRRDYMVQI	QLRFCLAETS	CPQEDNYPNS
101	111	121	131	141
LCIKVNGKPF	PLPGYPPPPK	NGVEQKRQGR	PLNITSSVRL	SSAVPNQISV
151	161	171	181	191
TWAPEIGKTY	SMSVYLVRQL	TSSLLLQRLK	MKGIRNPDHS	RALIKEKLTA
201	211	221	231	241
DPDSEIATTS	LRVSLMCPLG	KMRLTVPCRA	VTCSHLQCFD	AALYLQMNEK
251	261	271	281	291
KPTWICPVCD	KKAAYESLII	DGLFMEILND	CTDVDEIKFQ	EDGSWCPMRP
301	311	321	331	341
KKETLKVSAQ	CSTKIETSDP	SSLKKADIID	LTIESSSEGE	EEEEEIPPLK
351	361	371	381	391
KRCLYMSKPE	EVHGKGVLTY	QPSTVRVPNV	QTVETSYITS	PLADYAVPFH
401	411	421	431	441
HSTLSTISAD	MQISETDAQW	DSGRYRTPMF	LDNLPSTLQQ	QATSGGLIST
451	461	471	481	
SSHYESSSHS	SGSARNETGV	ITGGGGTIIP	DIISLD	

Legend:

The selection scale:

1	2	3	4	5	6	7

Positive selection — Purifying selection

Figure S2

Gene: *pik3r1*

1	11	21	31	41
MSAEGYQYRA	LYDYKKEREE	DIDLHLGDIL	IVSKGALLAL	GYGDGFEEKP
51	61	71	81	91
DEIGWLHGFN	ETTQEKGDFP	GTYVEFIGKK	RISPPTPKPR	PQRPLPVAPG
101	111	121	131	141
PTKADSDSEQ	ALLDLTEQFS	PPELAPPILV	KLVEAIERKG	LDCPTLYRNF
151	161	171	181	191
GSCSGLEIRQ	CFESDPCAVD	LDQFDLQTLC	DGLRRYLQDL	PHPVVPVSVY
201	211	221	231	241
SEMVHVAQEV	HNPEECALLL	RRIVQSPNVP	HQYWLTLQYL	IKHFCRVCQN
251	261	271	281	291
TGKNLLSARV	LGDVFSPLVF	RHQAASCDAS	PEYHIRIIEV	FITSEWNESQ
301	311	321	331	341
AAPALPPKPP	KPAPVTNNGM	NNNMSLQDAE	WYWGDISREE	VNEKLRDTAD
351	361	371	381	391
GTFLVRDAST	KMHGDYTLTL	RKGGNNKLIK	IFHRDGKYGF	SDPLTFNSVV
401	411	421	431	441
ELINHYRHES	LAQYNPKLDV	KLLYPVSKHQ	QDQVVKEDNV	EAVGKKLHEY
451	461	471	481	491
HQQYQEKNKE	YDRLYEEYTR	TSQEIQMKRT	AIEAFNETIK	IFEEQCQTQE
501	511	521	531	541
RYSKEYIEKF	RREGNEKEIQ	RIMVNYEKLK	SRISEIVDSK	RRLEEDLKKQ
551	561	571	581	591
AADYREIDKR	MNSIKPDLIQ	LRKTRDQYLM	WLTQKGVRQK	KLNEWLGIKN
601	611	621	631	641
ETTEDQYSMV	EDDEDLPHHD	ERSWKLGNIN	RVQAEALLRG	KRDGTFLVRD
651	661	671	681	691
SSKQGCFACS	VVVDGEVKHC	VINKTPTGYG	FAEPYNLYNS	LKELVLHYQH
701	711	721		
TSLVQHNDSL	NVTLAYPVYA	HQRR		

Legend:

The selection scale:

1	2	3	4	5	6	7

Positive selection Purifying selection

Figure S2

Gene: *pik3r2*

1	11	21	31	41
MSAEGFQYRA	LYVYNKDWDE	DVDLQPGDLL	TVNKASLLAL	GFKEGDEERP
51	61	71	81	91
GELGWMVGVN	ERTKQRGDFP	GTYVQYVGPV	KMSLPLNQPR	FQRPLPAAPR
101	111	121	131	141
AEPPPVRQAL	PPKPPKTKAL	TNLVANGNSS	LLADAEWYWG	DISREEVNEK
151	161	171	181	191
MRDTPDGTFL	VRDASSKVQG	EYTLTLRKGG	NNKLIKIFHR	GGMYGFSEPL
201	211	221	231	241
TFLSVVELIS	HYRHESLAQY	NAKLDTRLLY	PVSKYQQEQV	VKEDSIEAVG
251	261	271	281	291
EQLRVYHEQY	QEKSREYDVL	YEEYTRTSQE	LQMKRTAIEA	FNETIKIFEE
301	311	321	331	341
QCETQERYSK	EYIEKFRREG	NDKEIQRIQS	NSEKLKSRVT	EIHDSKRKLE
351	361	371	381	391
QDLKKQATDN	REIDKRMNSL	KPDLMQLRKI	RDQYLVWLTQ	KGTRQRRINE
401	411	421	431	441
WLGITNEAED	LYSLMDEEDD	LPHHDERMWY	VGDVKRVQAE	DLLRGKRDGT
451	461	471	481	491
FLIRDSQSQK	GSYACSVVVD	GDIKHCVIYK	TVTGYGFAEP	YNLYGSLKEL
501	511	521		
VLHYKHTSLV	QHNDSLNVTL	AWPVLSPQPR		

Legend:

The selection scale:

1	2	3	4	5	6	7

Positive selection — Purifying selection

Figure S2

Gene: *ppap2a*

1	11	21	31	41
MFDTKGIPFV	LLDLACLVLA	GLPLAAFNLA	KIKPYRRGFF	CNDESIGYPF
51	61	71	81	91
HSSTVTSTTL	YTVGFTLPIC	SMVVGECLSV	YLNRLKSKSS	FSNSYVACVY
101	111	121	131	141
KAVGTFVFGA	AMSQSLTDIA	KYSIGRLRPH	FLDVCKPDWR	LINCSAGAYI
151	161	171	181	191
ENFTCTGDKT	MVNEGSSSFK	QGHNIIKLIR	LLKEILALYL	QARMQAEWAR
201	211	221	231	241
LLRPTIQFFL	IAASVYTGLS	RVSDYKHHWS	DVLTGLIQGA	VMAVLVVFFV
251	261	271	281	
SDFFKKPVQP	QKPVEIPHTT	LQETPTNGNH	YDSPN	

Legend:

The selection scale:

1	2	3	4	5	6	7

Positive selection   Purifying selection

Figure S2

Gene: *pmepa1*

```
1          11         21         31         41
MGLTNGTTDT LANVSCTCNC QRSTSFQSMA ISQLEFVQIL VIVVVMMMMV
51         61         71         81         91
LVITCLLNHY RLSARSLMSR HTHERRRHLP LPSEGSLWSS DGPGSSSAMS
101        111        121        131        141
EVYTPRAPDR VPSFLQRERV SRFQPTFPFL PPVIELPPTI ALSDGEEPPP
151        161        171        181        191
YQGPCTLQLR DREQQLELNR ESVRPPPNRT VYDSALTHTG ISGGRLQGPP
201        211        221        231        241
PAYSEVIGHY YHPTTLPSPC RFRAPSAGTS PTAVVLASAP PLSLPNIHSS
251        261        271        281        291
TRMFSPKPGH RKRPEELLPN QLTWKILGSL APGRCSMLSQ WERY
```

Legend:

The selection scale:

1	2	3	4	5	6	7

Positive selection — Purifying selection

Figure S2

Gene: *ppargc1a*

1	11	21	31	41
MAWDRCNQDS	VWRELECAAL	VGEDQPLCSD	LPELDLSELD	VSDLDADSFL
51	61	71	81	91
GGLKWYSDQS	EIISSQYGSE	PSNLFEKIDE	ENEANLLAVL	TETLDSLPVD
101	111	121	131	141
EDGLPSFDAL	AEGDVTNTSD	QSCPSSPDGS	PPTPEAEEPS	LLKKLLLAPA
151	161	171	181	191
NSQLSYNQYT	GGKAQNEAPS	NQRIRPTPAV	VKTENPSWNN	KQRVPCQQQQ
201	211	221	231	241
KPTRRPCAEL	LKYLTASDET	IQTKACEQKS	GGGSSSKVKS	GGASSSSSSV
251	261	271	281	291
ASSSSSSSKK	KPVHPSQQQL	HHQRAKPTTL	PLPLTPESPN	DQKGSPFENK
301	311	321	331	341
SIERTLSVEL	SGTAGLTPPT	TPPHKATQEN	PFKPSVKPKL	SSCSSSAQAS
351	361	371	381	391
KRPHYSDSAV	CPSIKKGPEQ	TELYAQLSKV	SALPAGQEDR	KSKRSTLRLF
401	411	421	431	441
GDHDYCQFVS	TKRDISLAYS	VESQDGRQLE	CWDLVLSPSS	LAVQQQNYSS
451	461	471	481	491
PDQDLWCNRE	GKQLQTYQNR	QQANSRKPLR	DQEIRAELNK	HFGNPKQAIY
501	511	521	531	541
NEDEKAGPQQ	RDESDSEYYY	KLTDYVHPGL	PMEGVFDDSE	DENEKFLYPW
551	561	571	581	591
EGTHLDLLFG	GSPSCSPSSC	SPSRSSVSPP	KSLFSSQRCH	WSRSASHSRS
601	611	621	631	641
RSRSSSCHRR	RSYSRSPYSR	SRSRSPYSRS	SSRSHHCVDS	SNTRPRSRRS
651	661	671	681	691
PPIHSRSQSR	SPFSRRPRYD	SYEEYQHERL	KREEYRRDYE	KREFERAEQR
701	711	721	731	741
ERQRQKAIEE	RRVVYVGRLR	PDITRTELKR	RFEVFGEIEE	CAVNLRDDGD
751	761	771	781	791
NFGFITYRYT	CDAFAALENG	HTLRRSNEPQ	FELCFGGRKQ	FCKSNYTDLD
801	811	821	831	
SHSDDFDPSS	TKSKYDSMDF	DSLLKEAQRS	LRR	

Legend:

The selection scale:

1 2 3 4 5 6 7

Positive selection — Purifying selection

Figure S2

Gene: *pten*

1	11	21	31	41
MFQWRRSCCL	AHLVISYIYP	NIIAMGFPAE	RLEGVYRNNI	DDVVRFLDSK
51	61	71	81	91
HKNHYKIYNL	CAERHYDAAK	FNCRVAQYPF	EDHNPPQLEL	IKPFCEDLDQ
101	111	121	131	141
WLSEDDNHVA	AIHCKAGKGR	TGVMICAYLL	HRGKFLKAQE	ALDFYGEVRT
151	161	171	181	191
RDKKGVTIPS	QRRYVYYYSY	LLKNQLEYKP	VALLFHKMVF	ETVPMFSGGT
201	211	221	231	241
CRDTAVKNKT	EQIPHGFRKG	NWHWDPQFVV	YQLKVKIHTS	NPAHTRREDK
251	261	271	281	291
YMFFEFPQPL	PVCGDIKVEF	FHKQNKMMKK	DKMFHFWINT	FFIPGPEENL
301	311	321	331	341
EKVENGSLVG	VKELDGIQST	ERGDNDKDYL	ILTLTKNDLD	KANKDKANKN
351	361	371	381	391
FSPNFKVKLY	FTKTVEEPSN	SEASTSTSVT	PDVSDNEPDH	YRYSDTTDSD
401	411			
PENEPFDEEQ	HTQITKV			

Legend:

The selection scale:

1	2	3	4	5	6	7

Positive selection — Purifying selection

 T. Lorin, W. Salzburger & A. Böhne

Figure S2

Gene: *ptk2a*

```
1          11         21         31         41
MAAAYLDPNL NHALGSGAKS RLSMGMERSP GALDRVLKVF HYFESNSEPS
51         61         71         81         91
TWASNIRHGD ATDVRGIIQK IVDIHKVKCV SCYGLRLSHL QSEEIHWLHP
101        111        121        131        141
DMGVSHVREK YELNHPQEEW RYELRIRYLP KGFLNQFTDD KPTLNYFYQQ
151        161        171        181        191
VRNDYMLEIA DQVDQEIALK LGCLEIRRFF REMRGNALDK KSNYELLEKD
201        211        221        231        241
VGLRRFFPKS LLDSVKAKTL RKLIQQTFKQ FANLNDEQSI LKFFEILSPV
251        261        271        281        291
YRFDKECFKC ALGSSWVISV ELAIGPEEGI SYLTDKGSTP THLANFNQVQ
301        311        321        331        341
TIQYSSSDDK DRKGMLQLNV AGAPEPLTVT TPSLTIAENM ADLIDGYCRL
351        361        371        381        391
VNGASQSFII RPQKAEGERA LPSIPKLANN EKQGVRTRTV SVSETDDYAE
401        411        421        431        441
IIDEEDTYTM PSTRDYEIQR ERIELGRCIG EGQFGDVHQG VYMSPENPSL
451        461        471        481        491
SVAIKTCKNC TSDSVREKFL QEALTMRQFD HPHIVKLIGV ITENPVWIIM
501        511        521        531        541
ELCTLGEVRT QFMGIVGKES MSLSVTLMYV VHNLLKPLNT DSSYCQSKLI
551        561        571        581        591
VVVAVAENNS RTSTSLSLLL QHLWENKYIH NNTHLNTEEL IEQLVLPVLW
601        611        621        631        641
PADRSSERDT VFAGVCMWEI LMYGVKPFQG VKNNDVIGRI ENGERLAMPP
651        661        671        681        691
NCPPTLYSLM TKCWAYDPSK RPRFTELKVQ LSTILEEEKA QQEERIRMEM
701        711        721        731        741
RRQVTVSWDS GGSDEAPPKP SRPGYPSPRS SEGFYPSPQH AVQHNHYQVS
751        761        771        781        791
GYPGSHGMSS MPSAVYPPQA SVLDPHDSWN HHRPQDIPMW SPNMEEGGAL
801        811        821        831        841
DLRGMGQGLP THLMEERLMM QQQQMEEDQR WLEQEERFLK PDPRNSRGSI
851        861        871        881        891
DREDCSLQGP MGNQHIYQPV GKPEHVAPPK KPPRPGAPSH LGSLASLNPV
901        911        921        931        941
DSYNEGVKIQ PQEISPPPTA NLDRSNDKVY ENVTGLVKAV IEMSSKIQPA
951        961        971        981        991
PPEEYVPMVK EVGLALRTLL ATVDETIPVL PASTHREIEM AQKLLNSDLA
1001       1011       1021       1031       1041
```

Figure S2

ELINKMKLAQ QYVMTSLQQE YKKQMLTAAH ALAVDAKNLL DVIDQARLKM

1051

IGQPRPH

Legend:

The selection scale:

1	2	3	4	5	6	7

Positive selection Purifying selection

Figure S2

Gene: *ptk2b*

1 11 21 31 41
MSGDSRTLSW TLGSTGKGEP SGLSERTNDT LSVGDKIIKV CFTSNSFNLG
51 61 71 81 91
KNFKLVKCDS SWEVKEIINS ILSSGRLGPN ITFSGCYGLL LKHLKSDEIY
101 111 121 131 141
WLHPDLTVGD VVQKYEQRHC EAEWRYDLRI RYIPNNFIEK FKEDRTTLLY
151 161 171 181 191
FYQQVRCDYM QRCANKVSDG MALQLGCLEI SNRRFYKDMN AKGLEKKSNF
201 211 221 231 241
ELLEKDVGLD LFFPKELMDS MKPKHLRKMI QQTFQQYATL KEDECIAKFF
251 261 271 281 291
ETLSSVSSFE EEVYPCELVQ GWSIAVDLVI GPKGIRQRSN KEAVAICLAE
301 311 321 331 341
FKQIRSIKCS LQDNDRGQLH LEITGAKQLL SINTSNLAMA ENMADLIDGY
351 361 371 381 391
CRLENGANTS VIVRPRKDKD FRNSLPAVPT ADHKNMGSNT TVRESVGSDI
401 411 421 431 441
YAEIPDERPK SVIKFGISRD DIVLGRILGE GFFGEVHEGV YKDKKGERIS
451 461 471 481 491
VAVKTCKDCS PDVKEKFMSE AVIMKKLDHP HIVRLIGIIE EDPVWIVMEL
501 511 521 531 541
YQYGELGNYL TDNKHNLTTA TLILYSLQIC KALAYLEGVN MVHRDIAVRN
551 561 571 581 591
VLVAAPECVK LGDFGLSRYI EDEEYYKASV TRLPIKWMAP ESINFRRFTC
601 611 621 631 641
SSDVWMFAVC MWEIMSMGQQ PFFWLENKDV INQLEQGVRL PKPDICPPTL
651 661 671 681 691
YTLMTRCWTY DPQERPKFTE LVCKLSDMFK MEKEQETLQQ SKNRSRATKF
701 711 721 731 741
FDPIITVSEP PPKPSRMKSS RFGSTLNVGL QIQLPESLCA SSPAIASPPD
751 761 771 781 791
YQTPVDSNNR LLLPRQVPRR RSMGEGDFIV EPVSKEDAQR LWEIEKARMQ
801 811 821 831 841
ETLKRQKQEM IEDNKWLEKE EKLLDPMIQE GSKTSEVPEK ESGYAQFTGP
851 861 871 881 891
PEKPPRLTAQ QPAPTAEMDR SEDKVYHFVM ELVKVVVQLK NDVNVLPASE
901 911 921 931 941
YVNVVKSVGL TLRDLIRSVD EVLPTLHASK RTEIEGTQKL LNKDMAELIS
951 961 971 981 991
KMKLAQQNAI TSLSEECKRQ MLAAAHTLAM DSKNLLDAVD QARVRANTAK
1001

Figure S2

PSAS

Legend:

The selection scale:

1	2	3	4	5	6	7

Positive selection Purifying selection

Figure S2

Gene: *rac1*

1	11	21	31	41
MQAIKCVVVG	DGAVGKTCLL	ISYTTNAFPG	EYIPTVFDNY	SANVMVDGKP
51	61	71	81	91
VNLGLWDTAG	QEDYDRLRPL	SYPQTDVFLI	CFSLVSPASF	ENVRAKWYPE
101	111	121	131	141
VRHHCPNTPI	ILVGTKLDLR	DDKDTIEKLK	EKKLTPITYP	QGLAMAKEIG
151	161	171	181	191
AVKYLECSAL	TQRGLKTVFD	EAIRAVLCPP	PVKKRRRKCR	LF

Legend:

The selection scale:

1	2	3	4	5	6	7

Positive selection   Purifying selection

Figure S2

Gene: *raf1*

1	11	21	31	41
MEHIQGAWKT	ISNGFGLKES	VFDGQCMSPT	IVQGFPYQRR	SSDDGKIPDP
51	61	71	81	91
SKASSTIRVY	LPNKQRTVVN	VRPGVTLHNC	LIKALKVRGL	QPECCAVFRL
101	111	121	131	141
HPGQRSRKSR	MDWNTDSTSL	IGEELLVEVL	DHVPLTTHNF	VRKTFLKLAF
151	161	171	181	191
CDICQKFLLN	GFRCQTCGYK	FHEHCSTKVP	TMCVDWSNIR	QLLLFPTPGE
201	211	221	231	241
SGAPSLPPLT	SRRMRDSLSR	FPPSSQHRHS	TPHAFNYITP	YPPTGGALSQ
251	261	271	281	291
RQRSTSTPNV	HMVSTTMPVD	SSIIEDAIRS	HESGSPTSQS	PTGWSQSKTP
301	311	321	331	341
VPAHRERAPA	SNTQEKNKIR	PRDKRDSSYY	WEIEANEVML	LSRIGSGSFG
351	361	371	381	391
TVYKGKWHGD	VAVKILKVTD	PTPEQFQAFR	NEVAVLRKTR	HVNILLFMGY
401	411	421	431	441
MTKDNLAIVT	QWCEGSSLYK	HLHVQETNFQ	MFRLIDIARQ	TAQGMDYLHA
451	461	471	481	491
KHIIHRDMKS	NNIFLHEGLT	VKIGDFGLAT	VKARWSGSHQ	VEQPSGSILW
501	511	521	531	541
MAPEVIRMQD	NNPYSFQSDV	YSYGIVLFEL	MTGELPYSHI	ASRDQIIYMV
551	561	571	581	591
GRGYLSPDLS	KLYKSCPKAM	KRLVADCIKK	SKDERPLFPQ	ILSSIELLQH
601	611	621	631	
SLPKINRSAS	EPSLHRASHT	EDINSCTLTS	MKLPVF	

Legend:

The selection scale:

1	2	3	4	5	6	7

Positive selection   Purifying selection

Figure S2

Gene: *ran*

1	11	21	31	41
XXXXXXXXXX	XXXXXXXXXX	XXXXXXXXXX	XXXXXXXXXA	TLGVEVHPLV
51	61	71	81	91
FHTNRGAIKF	NVWDTAGQEK	FGGLRDGYYI	QAQCAIIMFD	VTSRVTYKNV
101	111	121	131	141
PNWHRDLVRV	CENIPIVLCG	NKVDIKDRKV	KAKSIVFHRK	KNLQYYDISA
151	161	171	181	191
KSNYNFEKPF	LWLARKLIGD	PNLEFVAMPA	LAPPEVSMDP	SLAAQYEHDL
201	211			
KVASETALPD	EEDDL			

Legend:

The selection scale:

1	2	3	4	5	6	7

Positive selection Purifying selection

Figure S2

Gene: *rb1*

1	11	21	31	41
MPPKRRSAAP	SAQLSKAAED	KHTNRVGVPG	TPGRIPQLSL	ERHKDKEPPF
51	61	71	81	91
VSLCQELRVT	DEVCNLAWKI	WETMVASVDI	VLLLGIKDTS	RKHWGACIFV
101	111	121	131	141
AATDLDAMTF	TITEVQKTVG	LNVVEFFNLL	KKMDINMDTI	SVKVNTAVSR
151	161	171	181	191
LEKKYEVLLA	LFQRFERTCG	KIYIAVSDNA	GNAGPDAKQI	MRSCWTLFLL
201	211	221	231	241
AKGNVLQMED	DLVISFQLLL	CVLEYFIKRS	PPTVLQEPFK	IAANGVHSTT
251	261	271	281	291
PTRTSRRGLG	RGARAAPKPA	DTDLRVMEAL	CKENECTVDE	VKNVYFTSFV
301	311	321	331	341
AFLDSVGLAG	SQEIPESEDL	SKQYEELYFK	SKDFDARLFL	DHDETLQPVK
351	361	371	381	391
TDVSQLERTP	RKNPLEEEVL	PIPPQTPVRA	AMNCIQQLKV	ILNSASDWPS
401	411	421	431	441
ENLVKYFSNC	TVNPSEEITE	RVVNMRELFI	DRFARAVGQQ	CIEIGSQRFV
451	461	471	481	491
LGVRLYYRVM	ESMLRSEEER	LSIQNFSKLL	SNADFHTSLL	ACALEVVMAT
501	511	521	531	541
YDWSTFKSSS	SSSETDLSFP	WILDVFKLKA	FDFYKVIESF	IKAEPSLTRE
551	561	571	581	591
MIKHLERCEH	LIMESIAWRS	DSPLFELLRQ	SREQEGAVEQ	AEPPGMLNQL
601	611	621	631	641
LQHDHTAADL	YLSPVRSPWK	KSQSVASSAS	QGADAQTGPS	SQSQTSSHKH
651	661	671	681	691
LKSTSLSLFY	KKVYRLAYLR	LNMLFSHLLK	DHPELEHIIW	TLFQHTLQHE
701	711	721	731	741
HELLKDRHLD	QIMMSAMYAI	CKVKNIDLRF	KTIVTAYKEL	PNTHQETFKH
751	761	771	781	791
VLIREGQYDS	IIAFYNQVFM	QKLKTNILQY	ASPRPPTLSP	IPHIPCSPYK
801	811	821	831	841
FSNSPLRVPG	SNNVYVSPLK	SARMSPSVMT	PRSRILVSIG	ESFGTSEKFQ
851	861	871	881	891
RINQMVSSSD	WSLKRGLESN	SAPKPLKRLR	FDTDGQDEAD	GSKSAGESKF
901	911	921	931	
QQKLAEMTST	RTRMQEQKLK	DAAVDSLKEE	KH	

Legend:

The selection scale:

1	2	3	4	5	6	7

Positive selection   Purifying selection

Figure S2

Gene: *rhoaa*

1	11	21	31	41
MAAIRKKLVI	VGDGACGKTC	LLIVFSKDQF	PEVYVPTVFE	NYVADIEVDG
51	61	71	81	91
KQVELALWDT	AGQEDYDRLR	PLSYPDTDVI	LMCFSIDSPD	SLENIPEKWT
101	111	121	131	141
PEVKHFCPNV	PIILVGNKKD	LRNDEHTRRE	LAKMKQEPVK	PEEGRDMANR
151	161	171	181	191
INAFGYLECS	AKTKDGVREV	FEMATRAALQ	AKKRGKKNAC	LLL

Legend:

The selection scale:

1 2 3 4 5 6 7

Positive selection Purifying selection

Figure S2

Gene: *rhoab*

1	11	21	31	41
MAAIRKKLVI	VGDGACGKTC	LLIVFSKDQF	PEVYVPTVFE	NYVADIEVDG
51	61	71	81	91
KQVPLNALKM	EIYDRLRPLS	YPDTDVILMC	FSIDSPDSLE	NIPEKWTPEV
101	111	121	131	141
KHFCPNVPII	LVGNKKDLRN	DEHTRRELAK	MKQEPVKPEE	GRDMANRIGA
151	161	171	181	
FGYMECSAKT	KDGVREVFEM	ATRAALQARR	GKKTSKCLLL	

Legend:

The selection scale:

1 2 3 4 5 6 7

Positive selection Purifying selection

Figure S2

Gene: *rhob*

```
1          11         21         31         41
MAAIRKKLVV VGDGACGKTC LLIVFSKDEF PEVYVPTVFE NYVADIEVDS
51         61         71         81         91
KQVELALWDT AGQEDYDRLR PLSYPDTDVI LMCFSVDSPD SLENIPEKWV
101        111        121        131        141
PEVKHFCPNV PIILVANKKD LRNDENVRNE LSRMKQEPVK TEDGRAMAVR
151        161        171        181        191
IGAYDYLECS AKTKEGVREV FETATRAALQ KRSRPSGGCL NCCKLL
```

Legend:

The selection scale:

1	2	3	4	5	6	7

Positive selection — Purifying selection

Likelihood ratio test between the null model (no positive selection) and the alternative model (enabling positive selection) shows a significance level of: 0.05

Figure S2

Gene: *rnf4*

1	11	21	31	41
XQAQIKRRSG	GSSRRPSKRR	RVTNAPPAME	PETIEVLENN	LHSSEDVVDL
51	61	71	81	91
TCEGSEPAVV	DLTSNDSVVI	VDEGLQGHRE	ASGESYVLSS	DEEEVEDSGM
101	111	121	131	141
MPTPAFLSSL	ESSNKARATP	GTISCPICLD	TYGEIVGSGR	LVVSTKCGHL
151	161	171	181	
FCSQCIRDCL	RKASKCPTCR	KKLNYKQYHP	IYI	

Legend:

The selection scale:

1 2 3 4 5 6 7

Positive selection   Purifying selection

Figure S2

Gene: *rnf14*

1	MSSEDLEAQE	11	DELLALASIY	21	NEEEFHRAES	31	AQGGEIRINL	41	ELPQDFRIFA
51	KGEEHIKYPV	61	SFLPTLILNF	71	ELPSDYPSNS	81	PPVFTLSSRW	91	LSRVQLGTLC
101	KRLDELWQEN	111	VGSVVLFSWI	121	QFLKEDLFSF	131	LSIESPLEVT	141	NSNKGKQERK
151	KEGAGKDNAE	161	DTEKDSKEQP	171	DPRAIQEVDP	181	RSNLLLQLLD	191	FDEMQQQKAF
201	DSKMFCCNIC	211	FSEKMGSECT	221	CFKDCKHVYC	231	KECMRDYFQI	241	QIRDGNVQCL
251	NCPEPECSSV	261	ATPAQVKHLV	271	GEELFARYDR	281	LLLQSSLDLM	291	ADVMYCPRKM
301	CETAVLVEPD	311	TTMGICPSCQ	321	YAFCTLCKMG	331	YHGLSPCRAT	341	AAELRGLRDE
351	YLKASEEEKQ	361	FLEQRFGKRV	371	IQKAVEESFS	381	SEWLEKNSKA	391	CPRCSSNIQK
401	VDGCNKMTCT	411	SCKQYFCWLC	421	LGPLSRVNPY	431	GHFNDTSSPC	441	FNQLFQGVDI
451	DDDEEFWSDE	461	ED						

Legend:

The selection scale:

1 2 3 4 5 6 7

Positive selection — Purifying selection

Figure S2

Gene: *rock1*

1 11 21 31 41
MSAGESLEAR FEKIDAMLKD PKSEVNTDCL LDGLDALVYD LDFPALRKNK
51 61 71 81 91
SIDNFLNRYK DTISKIRDLR MKAEDYEVVK VIGRGAFGEV QLVRHKATRK
101 111 121 131 141
VYAMKLLSKF EMIKRSDSAF FWEERDIMAF ANSPWVVQLF YAFQDDRYLY
151 161 171 181 191
MVMEYMPGGD LVNLMSNYDV PEKWARFYTA EVVLALDGIH SMGFIHRDVK
201 211 221 231 241
PDNMLLDKAG HLKLADFGTC MKMNQDGMVR CDTAVGTPDY ISPEVLKSQG
251 261 271 281 291
GDGYYGRECD WWSVGVFLYE MLVGDTPFYA DSLVGTYSKI MNHKNALTFP
301 311 321 331 341
DDSEISKDAK NLICAFLTDR EVRLGRNGVD EIKRHPFFKN DQWTWENIRE
351 361 371 381 391
TAAPVVPELS SDIDTSNFDD IEEDKGDEET FPIPKAFVGN QLPFVGFTYY
401 411 421 431 441
SSNQLARGLS TKTSEKRSSS VKEDKSQLEN LQKRIYLLEE QLHSEMQLKD
451 461 471 481 491
EMEQKCRASN IKLDKIMKEL DEETNLRKNM DSTVSHLEKE KMMAQHRATE
501 511 521 531 541
YQRKAEQEAE KRRNVENEVS TLKEQLEDMR KISQNSQISN DKITQLQKQL
551 561 571 581 591
EEANDLLRVE SDTAARLRKS HTELTKSMSH LETVNRELQE KSRAADGARH
601 611 621 631 641
QLEKEVLQLQ AALESERRNW SQGSEEIQEL QGRITGLQED LKNLKLTLSK
651 661 671 681 691
VETERKQAQE RSNNLEKEKN NLEIDLNYKL KTLQQRLEHE LAEHAATKAR
701 711 721 731 741
LTDKYESIEE AKSATMQAVE QKVSEESMAR LKAENRVVEV EKQCSMLEFD
751 761 771 781 791
LKQSVQKIEQ LMKQKERLEE EVRSVRVQLE QESGRRVQAQ AELKTRAVEA
801 811 821 831 841
DRMKGSEKQL KQEMNTALEA KRSLEFQLAQ LTNYSPDMSG IFFPKTTHSS
851 861 871 881 891
LVTAVSAQYF EKACLDDIKL GFNGFDSRAC FSVAICFCTD EGGSCAGNIF
901 911 921 931 941
SSCGSRRILC EWSRRMFRCR QLEETNKTLT KDVENLSKEK TELSEKMRVQ
951 961 971 981 991
EEVSATEKEE LTNSVKAYYE KILNTERTLK TQAVNKLAEI MNRKDMKLDQ
1001 1011 1021 1031 1041

T. Lorin, W. Salzburger & A. Böhne

Figure S2

```
KKKGSTADLR KKEKENRKLQ LELNQEKEKF NHMAIKYQKE LSEMQAQLAE
1051       1061       1071       1081       1091
ESTYRNELQM QLDSKESDIE QLREKLNDLQ LRMDNSSVAS LQTDEADGNI
1101       1111       1121       1131       1141
AESRLEGWLS IPNRANIKRY GWKKQYVVVS SKKILFYNDE QDKEQSNPSM
1151       1161       1171       1181       1191
VLDIDKLFHV RPVTQGDVYR AETDEIPRIF QILYANEGEC RKDADTESVH
1201       1211       1221       1231       1241
QGEKTNCLPH KGHEFIPTLY HFPTNCEACA KPLWHVFKPP PALECRRCHV
1251       1261       1271       1281       1291
KCHKDHLDRK EDVIAPCKVN YDVTTARDML LLALSQEEQK KWIGHLGKKI
1301       1311       1321       1331
PKTPPSSFTR ASPRTMSTRS VANQSFRRNP KSVTGKPR
```

Legend:

The selection scale:

1	2	3	4	5	6	7

Positive selection Purifying selection

Figure S2

Gene: *rock2*

1 11 21 31 41
MSAGAERRME NRLKKLEAMI KDPRSLINLE SLLDSMNALV LDLDFPALRK
51 61 71 81 91
NKNIETFLNR YEKVTGKIQD LQMKSEDYEV VKVIGRGAFG EVQLVRHKAS
101 111 121 131 141
QKVYAMKLLS KFEMIKRSDS AFFWEERDIM AFANSPWVVQ LCCAFQDDRY
151 161 171 181 191
LYMVMEYMPG GDLVNLTSTY DVPEKWAKFY TAEVVLALDA IHSMGFIHRD
201 211 221 231 241
VKPDNMLLDR HGHLKLADFG TCMKMDKTGM VRCDTAVGTP DYISPEVLKS
251 261 271 281 291
QGGDGYYGRE CDWWSVGVFI FEMLVGDTPF YADSLVGTYS KIMDHKNSLN
301 311 321 331 341
FPDDVEISED AKNLICAFLT DREVRLGRSG VEEIKRHPFF KNDQWTFDTI
351 361 371 381 391
RETVAPVVPE LSSDIDTSNF DEIEDDKGDV ETFPVPKAFV GNQLPFIGFT
401 411 421 431 441
YFREDQLLSD SNPSAVENEH RTSIKGEDSV ASVQLQKKLH QLEEQLNNEM
451 461 471 481 491
QSKDELDHKC RAACSRLEKV SKELDEEITS RKHLESSLRQ LEREKALLQH
501 511 521 531 541
KNVEYQRKAE NEADKKRCLE NDVNSLRDQL EDLKKRNQNS QISNEKNIQL
551 561 571 581 591
QRQLDEVNSL LRTESDTAAR LRKTQTEMTK QTQQLESNNR ELQDKCCMLE
601 611 621 631 641
NAKLKLEKDF ISLQSALESE KRDRNHGTEI ISDLQGRISG LEDELKHVKC
651 661 671 681 691
SLSKAEMEKR QLHERLTDFE KEKSNIEIDM TYKLKVLQQS LEQEEAEHKA
701 711 721 731 741
TKARLADKNK IYESIEEAKS EAMKEMEKKL QEERSSKMKV ENILLEVEKQ
751 761 771 781 791
CSMLDCDLKQ SHQKLEELRR QKEKLTEEVK NLTLKIEQET QKRSLTQNDL
801 811 821 831 841
KMQSQQVNAL KMSEKQLKQE INHLLEIKLS LEKQNSELRK ERQDADGQMK
851 861 871 881 891
ELQDQLEAEQ YFSTLYKTQV RELKEECEEK NKLCKEMQQK LQELQDERDS
901 911 921 931 941
LAAQLEITLT KADSEQLARS IAEEQYSDLE KEKIMKELEI KEMMARHKQE
951 961 971 981 991
LAEKDATIGS LEEANRTLTS DVANLANEKE ELNNRLKETQ EQLQKAKEDE
1001 1011 1021 1031 1041

Figure S2

```
HSMALTKVQF EKQLQSERTL KTQAVNKLAE IMNRKEVRGG ASRRGNDTDV
1051       1061       1071       1081       1091
RRKEKENRKL QLELRSEREK LNGTIIKYQK EINEMQAQIA DESQVRIELQ
1101       1111       1121       1131       1141
MALDSKDSDI EQLRSLLNSM NVHGLDSASI GSGPDFDSDE SYPDTTRLEG
1151       1161       1171       1181       1191
WLSLPVRNNT KRFGWEKKYV VVSSKKILFY NNEQDKEQSN PYMVLDIDKL
1201       1211       1221       1231       1241
FHVRPVTQTD VYRADAKEIP RIFQILYANE GESKKDQEFP VEPLPIGEKS
1251       1261       1271       1281       1291
SYICHKGHEF IPTLYHFPTN CEACTKPLWN MFKPPPALEC RRCHIKCHKD
1301       1311       1321       1331       1341
HMDKKEEVIA PCRVNYDVST AKNLLLLANS QEEQQKWVSR LVKKIPKKPP
1351       1361       1371       1381       1391
APDHFSRSSP RASMKVQPNQ SIRRPSRQLP PNKPSTGPML RIILH
```

Legend:

The selection scale:

1	2	3	4	5	6	7

Positive selection Purifying selection

Figure S2

Gene: *src*

1	11	21	31	41
MGATKSKPKD	PGQRSRSLDG	TIGIVGSYHP	SHSPVQQTLT	PNRSPAAEGS
51	61	71	81	91
RRGNQSLTSN	AELVLFGGVD	SSIGITSPQR	VLIFLFSVTG	GVTTFVALYD
101	111	121	131	141
YESRTASDLS	FKKGERLQIV	NNTEGDWWLA	RSLTTGESGY	IPSNYVAPSD
151	161	171	181	191
SIQAEEWYFG	KITRRDSERL	LLNLENRRGT	FLVRESETTK	GAYCLSVLDY
201	211	221	231	241
DNTKGLNVKH	YKIRKLDSGG	FYITSRTQFS	NLQQLVSHYR	KHADGLCHPL
251	261	271	281	291
TEVCPVIKPQ	TQGLARDAWE	IPRESLRLDV	KLGQGCFGEV	WMGTWNGTTR
301	311	321	331	341
VAIKTLKPGT	MSTEAFLQEA	QVMKKLRHEK	LVQLYAVVSE	EPIYIVTEFM
351	361	371	381	391
GQGSLLDFLK	GDMGKILRLP	QLVDMASQIA	AGMAYVERMN	YVHRDLRAAN
401	411	421	431	441
ILVGDNLVCK	VADFGLARLI	EDNEYTARQG	AKFPIKWTAP	EAALYGRFTI
451	461	471	481	491
KSDVWSFGIL	LTELATKGRV	PYPGMVNREV	LDQVERGYRM	PCPAECPESL
501	511	521	531	541
HELMLTCWRK	EPEERPTFEY	LQSFLEDYFT	STEPQYQPGE	NL

Legend:

The selection scale:

1	2	3	4	5	6	7

Positive selection   Purifying selection

Figure S2

Gene: *tgb1i1*

```
1          11         21         31         41
XXXXXXXXXX XXXXXXXXXX XXXXXXXXXX XXXXXXXXXX XXXXXXXXXX
51         61         71         81         91
XXXXXXXXXX XXXXXXXXXX XXXXXXXXXX XXXXXXXXXX XXXXXXXXXX
101        111        121        131        141
XXXXXXXXXX XXXXXXXXXX XXXXXXXXXX XXXXXXXXXX XXXXXXXXXX
151        161        171        181        191
XXXXXXXXXX XXXXXXXXXX XXXXXXXXXX PVLTSSRQAA PPPQNPSGSL
201        211        221        231        241
DSMLGLLQSD LSRQGITTTA KGTCSACQKP IVGQVXXXXX XXXXXXXXXX
251        261        271        281        291
XXXXXXXXXX XXXXXXXXXX XXXXXXXXXX XXXXXXXXXX XXXXXXXXXX
301        311        321        331        341
XXXXXXXXXX XXXXXXXXXX XXXXXXXXXX XXXXXXXXXX XXXXXXXXXX
351        361        371        381        391
XXXXXXXXXX XXXXXXXXSE CYTPFVNGSF FEHEGQPLCE AHYHRSRGSV
401        411        421        431        441
CQACQEPIRG RCVTAMGAKF HPQHLVCAFC LKALSKGSFK EQDNKPYCQG
451
CFIKLFG
```

Legend:

The selection scale:

1	2	3	4	5	6	7

Positive selection — Purifying selection

 T. Lorin, W. Salzburger & A. Böhne

Figure S2

Gene: *thrap3*

1 11 21 31 41
MSKVPVSKSP SRSRSGSGSR SPSRSFSRSR TRTRSRSRSR KHRYSSRSRS
51 61 71 81 91
RSRSHSPPHN RERNYPREYQ NNREFRGYNR GFRRPYYYRG RGRGYYPRGR
101 111 121 131 141
YQRGGGYGNY RPNWQNYRQQ PQQHHQHSPR RGRSRSRSPK RRSGTPRSRS
151 161 171 181 191
RSRRSGSSKR NAAKELKESG RPDSAAKEAQ RAGSRDEEAA GAAGGEGAPD
201 211 221 231 241
RASGSWQGLI DYDTSPKRTS PAVRSAIIVS QGTAHPSPSL QSVAVKRPSP
251 261 271 281 291
AVKGGSPSPS RSSAAAQSKS LGNAPWQSSG PAPTSKSPPQ QSPTAVFSGF
301 311 321 331 341
GFFSKDDVRA GEKPSSVSTA FKKFLEEHKN KIQVAEWENG REKEQKMAEL
351 361 371 381 391
ERDKGNGKAG SFDKGAAYSG LKPDYYSKNE EEKYGYDDEF ELGSAAEFLK
401 411 421 431 441
GPQFGSAEAG EDQEKRHKVR NQKEREMEDE PKHKSKITIT ANRDMFDERF
451 461 471 481 491
NKWDELAYFP SAKEKLRKEE DAGEDDIDDV EEELYRSRKQ ERAAAAAAAA
501 511 521 531 541
KKAEASGYRG FSPDKAPKAS RKKEKQGQSP SPPARKSSEN REREMFNVRR
551 561 571 581 591
DDSPPRSTPA YSGKRSAEVS VRMDPFHEDY ASSSGVLANE RRFSRDLVHP
601 611 621 631 641
SKKDQEFRSI FQHIQSTQLR RSPSELFAQH IVTIVHHIKA QHFESSGMTL
651 661 671 681 691
NERFAMYQRR AAEMMKPRKS PEIHRRIDVS PSAFRKHSHL FEEMKSSRES
701 711 721 731 741
SSKDEGKKMK TDSMDLRLDI ERRKKYSSKE REHKRDGMRD SGESRGSSLS
751 761 771 781 791
RERSTEKSSK HHKKTKKRKK TRERSRSSSE SSSSSHSFKG GDYPEGPEQK
801 811 821 831 841
EEGFNKARLG VRDYGGPMER GRGRGGFQFR IRGRGWNRGN YPGNNSNGNP
851 861 871 881 891
SNPGNPVRSK EEEWDPEYTP KSKKYYLHDD REGEGEKKWV DNRGRGRGTF
901 911 921 931 941
QRGRGRFMFR KTSSSPKWTH DKFQGSGEEG ELPDDSDAEN KEEDKSGGTA
951
ATEQ

Figure S2

Legend:

The selection scale:

1	2	3	4	5	6	7

Positive selection Purifying selection

Figure S2

Gene: *ube3a*

```
1          11         21         31         41
MKRAAAKHLI ERYYHQLTEG CGNEACTNEF CASCPSFHRM DNNAAAVKAL
51         61         71         81         91
ELYKINAKLC DPHPSKKGTS SAYLENSAKG AHNNSCSDRK MNRKEAHAAR
101        111        121        131        141
DDFKDLNYLT EEKVYEILEI CGEKEDYSPL IRVIGRVFSS AEGLVQSFRK
151        161        171        181        191
AKQHTKEELK SLQGKDEDKD EDEKEKAACS AAAAAMEEDS GASSSRIGDG
201        211        221        231        241
ATQGDNNVHK LGPDEVSVDV EAVRRVYNKL LANEKIETAF LNALVYLSPN
251        261        271        281        291
VECDLTYHNV YSRDPNYLNL FIIVMENSNL HSPEYLEIAL PLFCKAMSKL
301        311        321        331        341
PLPAQAKLVR LWSQYSAEQI RRMMETFQQL ITYKVISNEF NSRNLVNDDD
351        361        371        381        391
SVVAATKCLK IVYYANVLGG DVDTDHNEEE DDEPIPESSE LTLQELLGEE
401        411        421        431        441
RRNKKGPRVD PLETELGVKT IDCRKPLIPF EEFVNEPLND VLEMDKDYTF
451        461        471        481        491
FKVETENKFS FMTCPFILNA VTKNLGLYYD NRIRMYSERR ITVLYSLVQG
501        511        521        531        541
QQLNPYLRLK VRRDHIIDDA LVRLEMIAME NPADLKKQLY VEFEGEQGVD
551        561        571        581        591
EGGVSKEFFQ LVVEEIFNPD IGMFTYDETT KLFWFNPSSF ETEGQFTLIG
601        611        621        631        641
IVLGLAIYNN CILDVHFPMV VYRKLMGKKG TFRDLADSHP VLFQSLKELL
651        661        671        681        691
EYEGSVEEDM MITFQISQTD LFGNPLMYDL RENGDKIPVT NENRKDFVTQ
701        711        721        731        741
YAEYILNKAV EKQFKAFRRG FHMVTNESPL KYLFRPEEIE LLICGSRNLD
751        761        771        781        791
FQALEETTEY DGGYNRDSRI IKDFWDIVHA FGEEQKRLFL QFTTGTDRAP
801        811        821        831        841
VGGLGKLKMI IAKNGPDTDR LPTSHTCFNV LLLPEYTTKE KLKERLLKAI
851
TYAKGFGML
```

Legend:

The selection scale:

1	2	3	4	5	6	7

Figure S3

Resulting trees of branch-site model aBS-REL implemented in HyPhy allowing for different Ka/Ks ratios among sites and among branches for all retrieved genes of the AR signaling pathway. When available, the sequence of the spotted gar was used to root the tree, otherwise trees were rooted at the split of Otophysa from the other teleosts.

Figure S3

Resulting trees of branch-site model aBS-REL implemented in HyPhy (Version 2.2.4) allowing for different Ka/Ks ratios among sites and among branches.
Visulaization over http://veg.github.io/hyphy-vision/absrel/ web interface

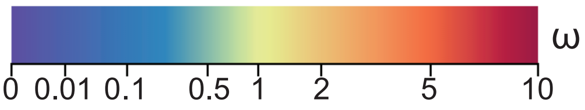

Ka/Ks selection scale for all pages of Figure S1

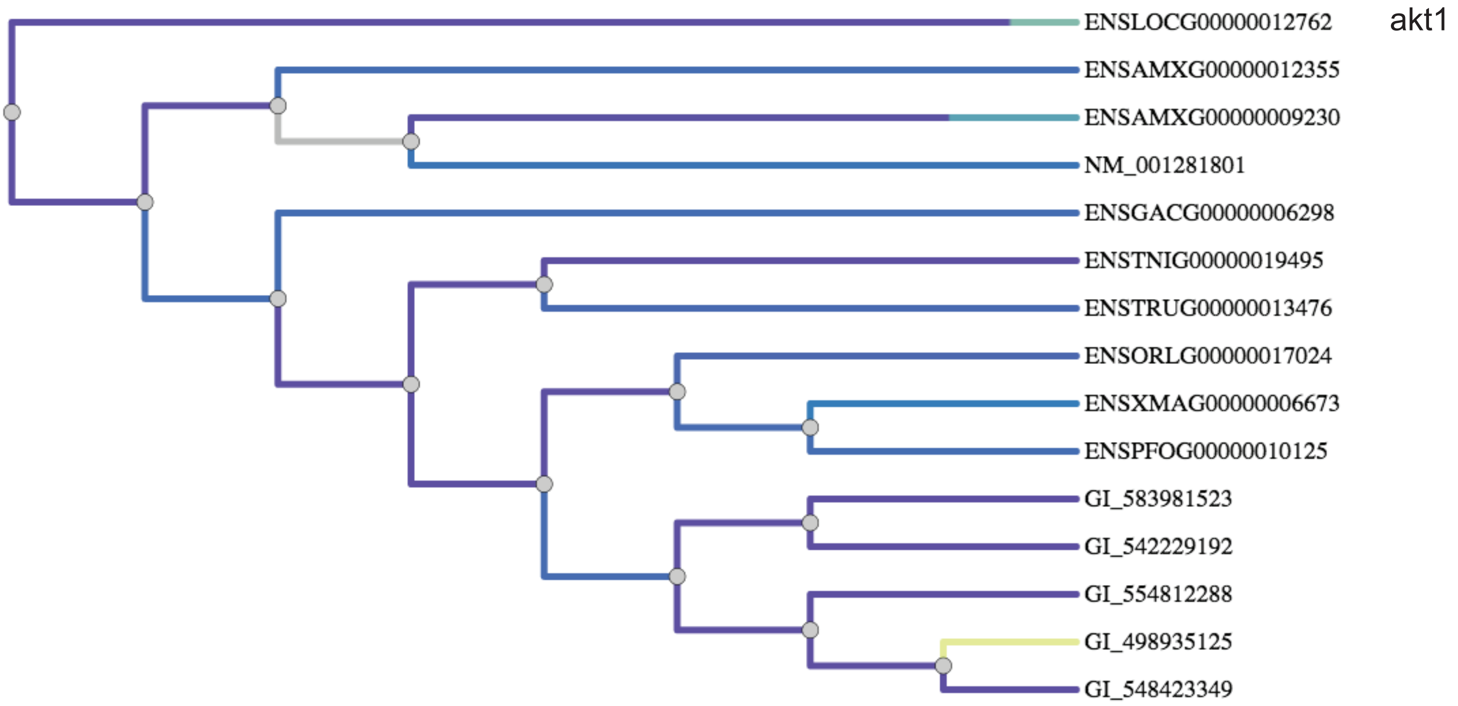

Figure S3

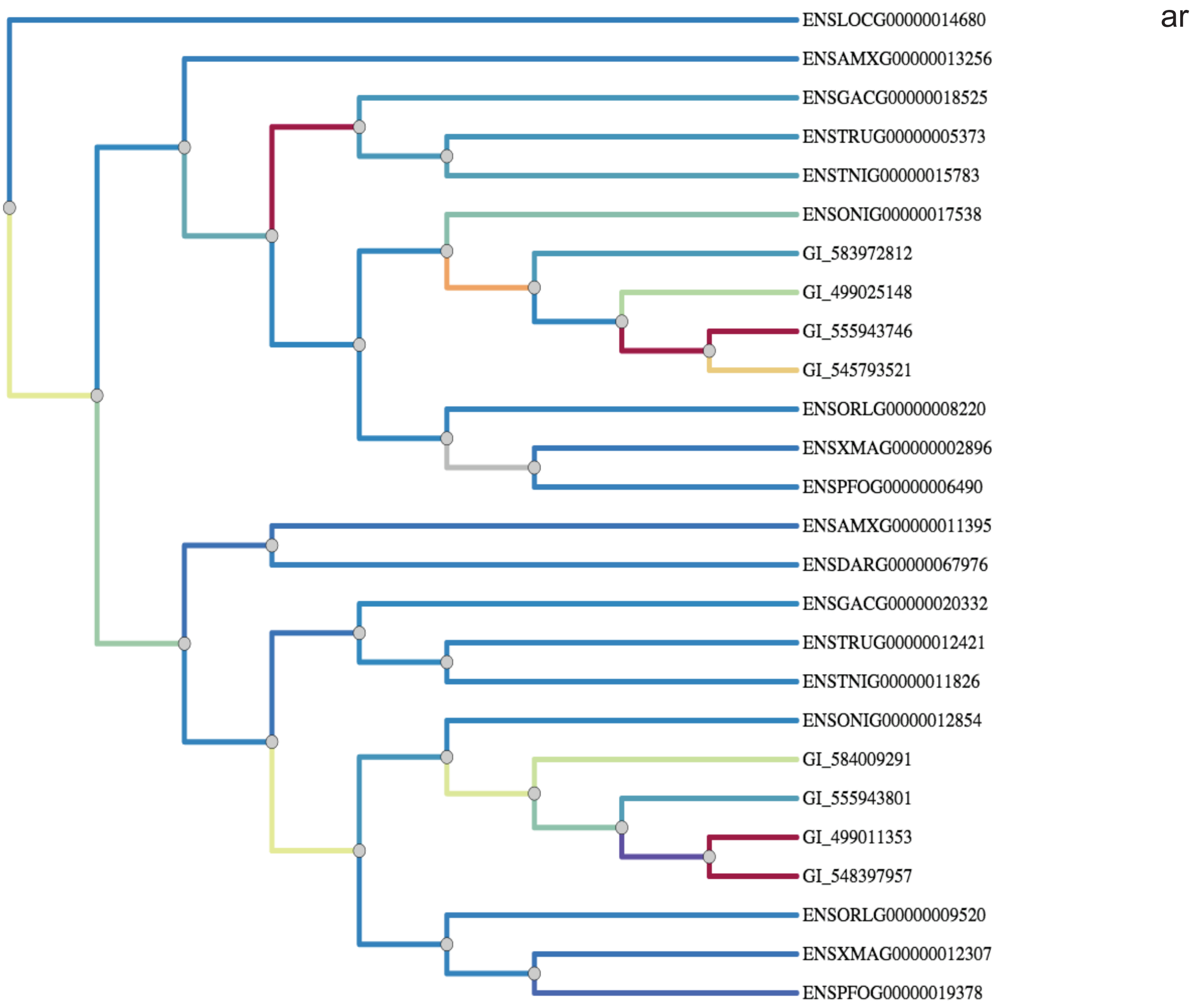

ar
ENSLOCG00000014680
ENSAMXG00000013256
ENSGACG00000018525
ENSTRUG00000005373
ENSTNIG00000015783
ENSONIG00000017538
GI_583972812
GI_499025148
GI_555943746
GI_545793521
ENSORLG00000008220
ENSXMAG00000002896
ENSPFOG00000006490
ENSAMXG00000011395
ENSDARG00000067976
ENSGACG00000020332
ENSTRUG00000012421
ENSTNIG00000011826
ENSONIG00000012854
GI_584009291
GI_555943801
GI_499011353
GI_548397957
ENSORLG00000009520
ENSXMAG00000012307
ENSPFOG00000019378

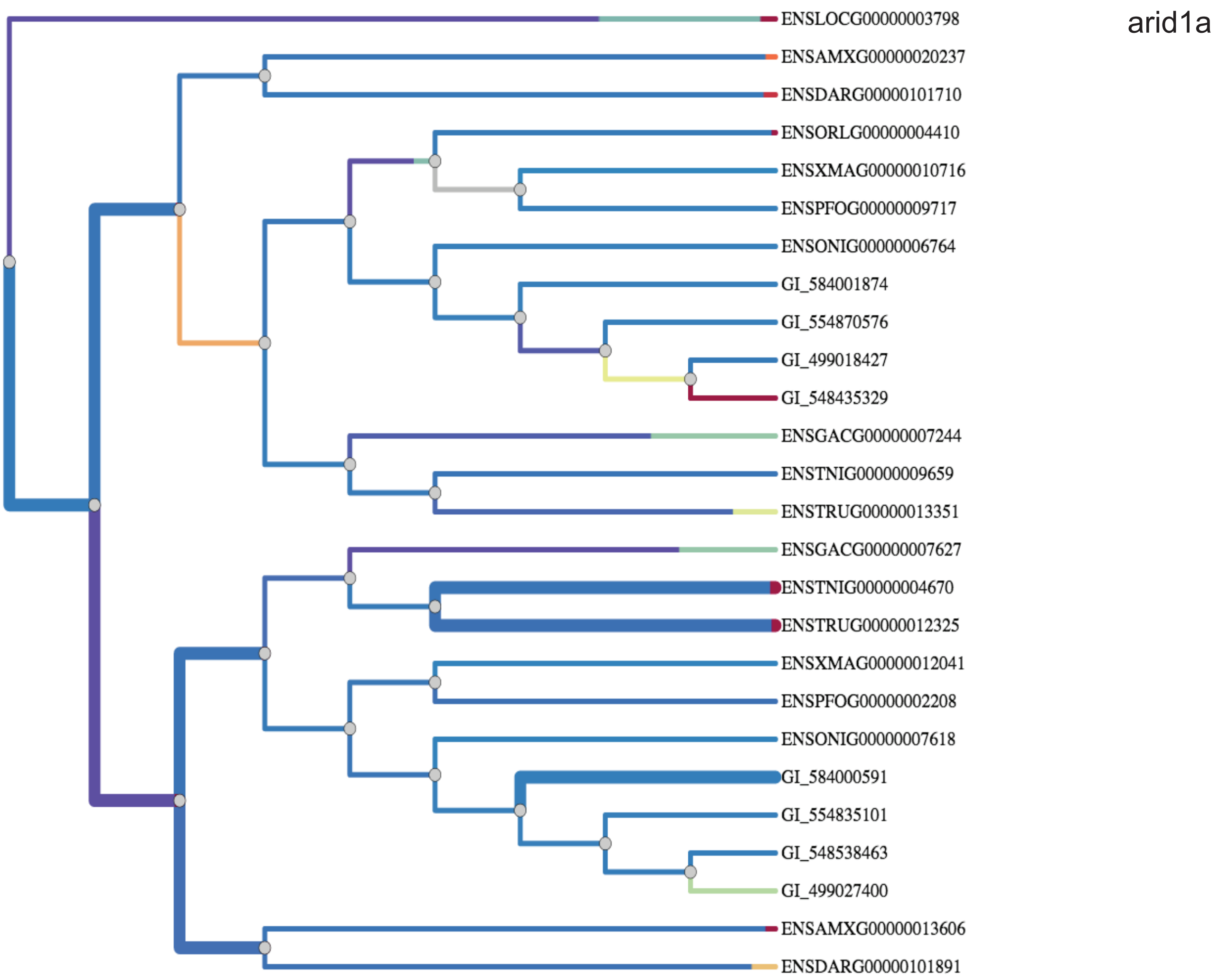

arid1a
ENSLOCG00000003798
ENSAMXG00000020237
ENSDARG00000101710
ENSORLG00000004410
ENSXMAG00000010716
ENSPFOG00000009717
ENSONIG00000006764
GI_584001874
GI_554870576
GI_499018427
GI_548435329
ENSGACG00000007244
ENSTNIG00000009659
ENSTRUG00000013351
ENSGACG00000007627
ENSTNIG00000004670
ENSTRUG00000012325
ENSXMAG00000012041
ENSPFOG00000002208
ENSONIG00000007618
GI_584000591
GI_554835101
GI_548538463
GI_499027400
ENSAMXG00000013606
ENSDARG00000101891

Figure S3

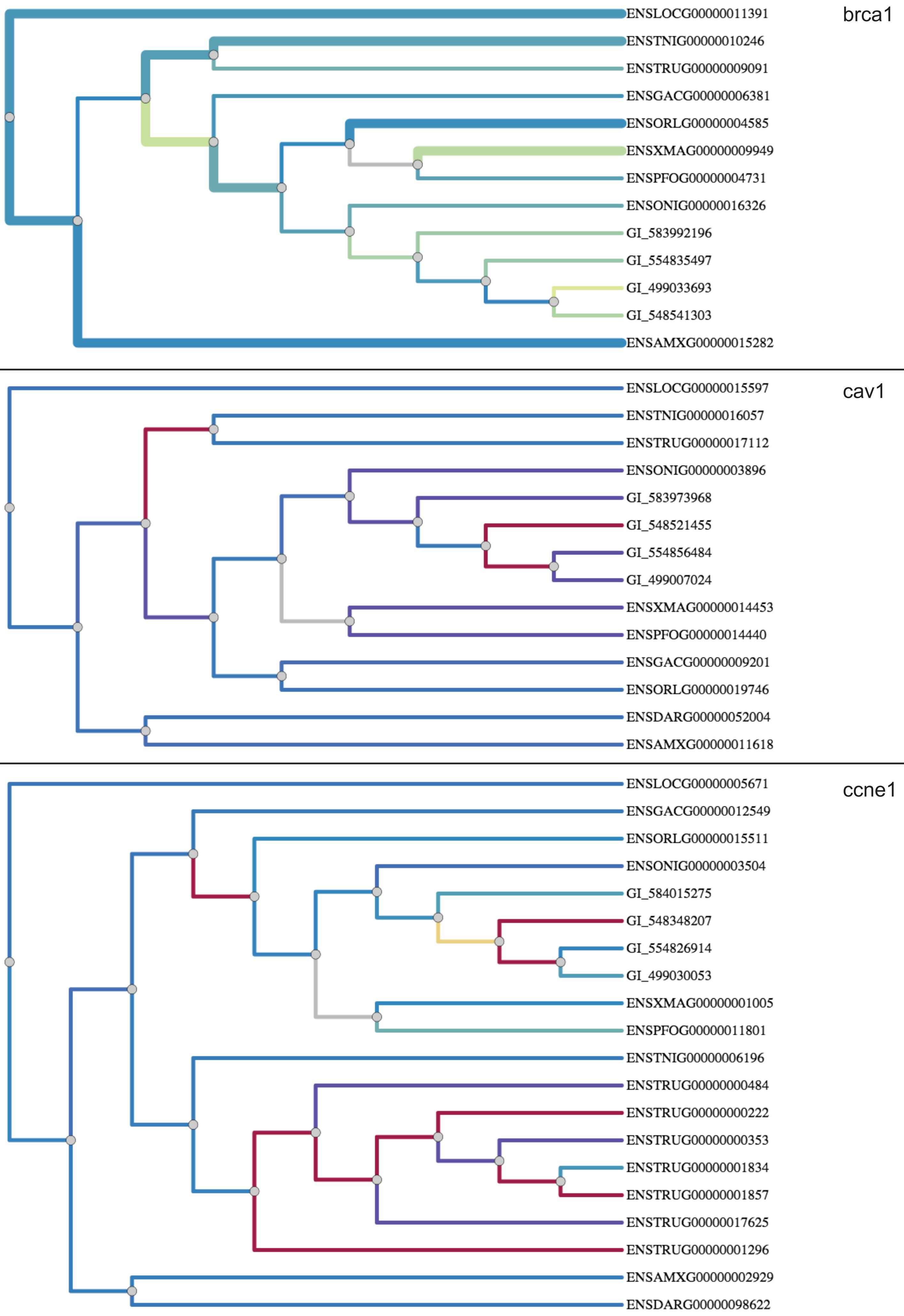
brca1
ENSLOCG00000011391
ENSTNIG00000010246
ENSTRUG00000009091
ENSGACG00000006381
ENSORLG00000004585
ENSXMAG00000009949
ENSPFOG00000004731
ENSONIG00000016326
GI_583992196
GI_554835497
GI_499033693
GI_548541303
ENSAMXG00000015282
cav1
ENSLOCG00000015597
ENSTNIG00000016057
ENSTRUG00000017112
ENSONIG00000003896
GI_583973968
GI_548521455
GI_554856484
GI_499007024
ENSXMAG00000014453
ENSPFOG00000014440
ENSGACG00000009201
ENSORLG00000019746
ENSDARG00000052004
ENSAMXG00000011618
ccne1
ENSLOCG00000005671
ENSGACG00000012549
ENSORLG00000015511
ENSONIG00000003504
GI_584015275
GI_548348207
GI_554826914
GI_499030053
ENSXMAG00000001005
ENSPFOG00000011801
ENSTNIG00000006196
ENSTRUG00000000484
ENSTRUG00000000222
ENSTRUG00000000353
ENSTRUG00000001834
ENSTRUG00000001857
ENSTRUG00000017625
ENSTRUG00000001296
ENSAMXG00000002929
ENSDARG00000098622

Figure S3

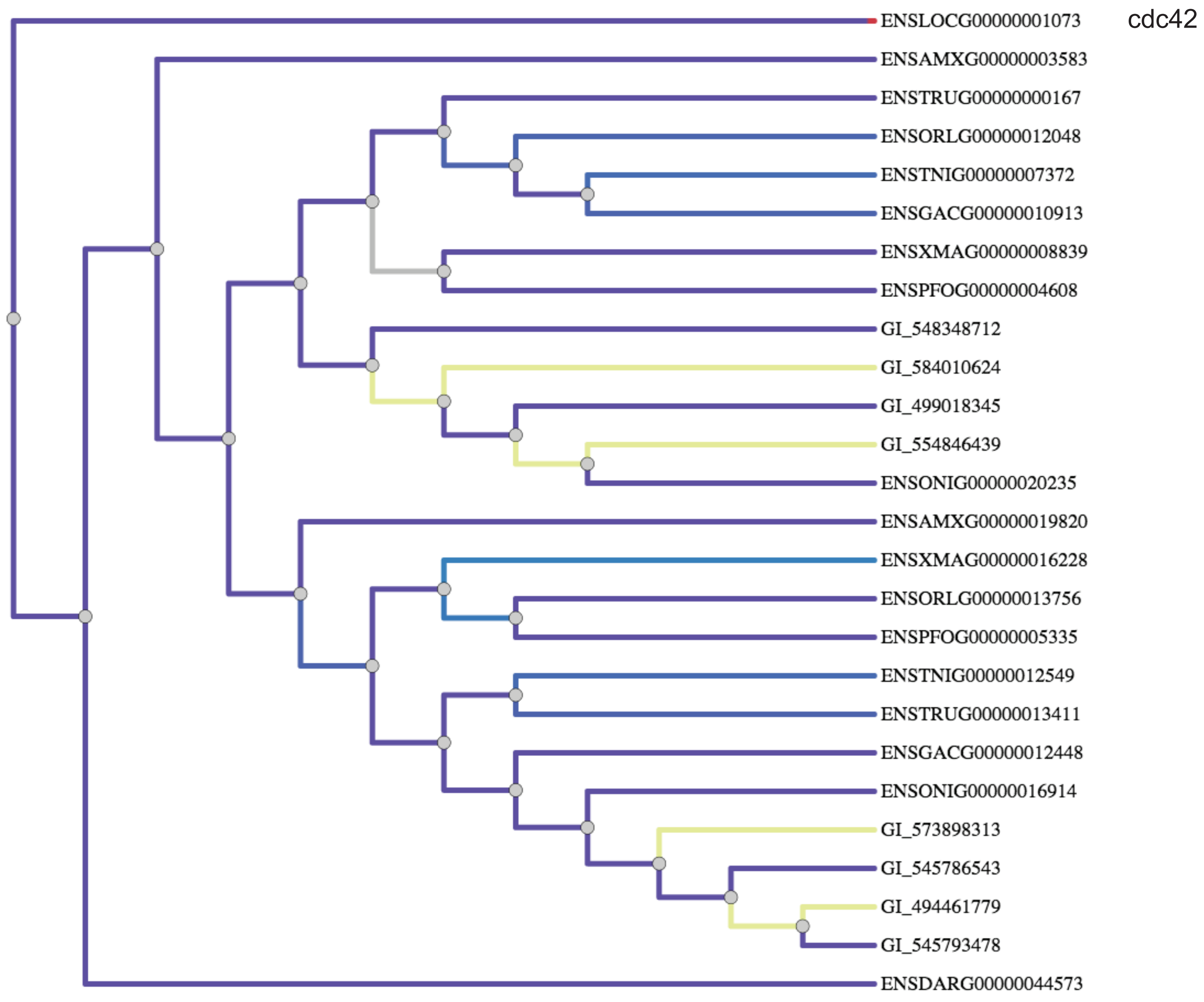
ENSLOCG00000001073
cdc42
ENSAMXG00000003583
ENSTRUG00000000167
ENSORLG00000012048
ENSTNIG00000007372
ENSGACG00000010913
ENSXMAG00000008839
ENSPFOG00000004608
GI_548348712
GI_584010624
GI_499018345
GI_554846439
ENSONIG00000020235
ENSAMXG00000019820
ENSXMAG00000016228
ENSORLG00000013756
ENSPFOG00000005335
ENSTNIG00000012549
ENSTRUG00000013411
ENSGACG00000012448
ENSONIG00000016914
GI_573898313
GI_545786543
GI_494461779
GI_545793478
ENSDARG00000044573

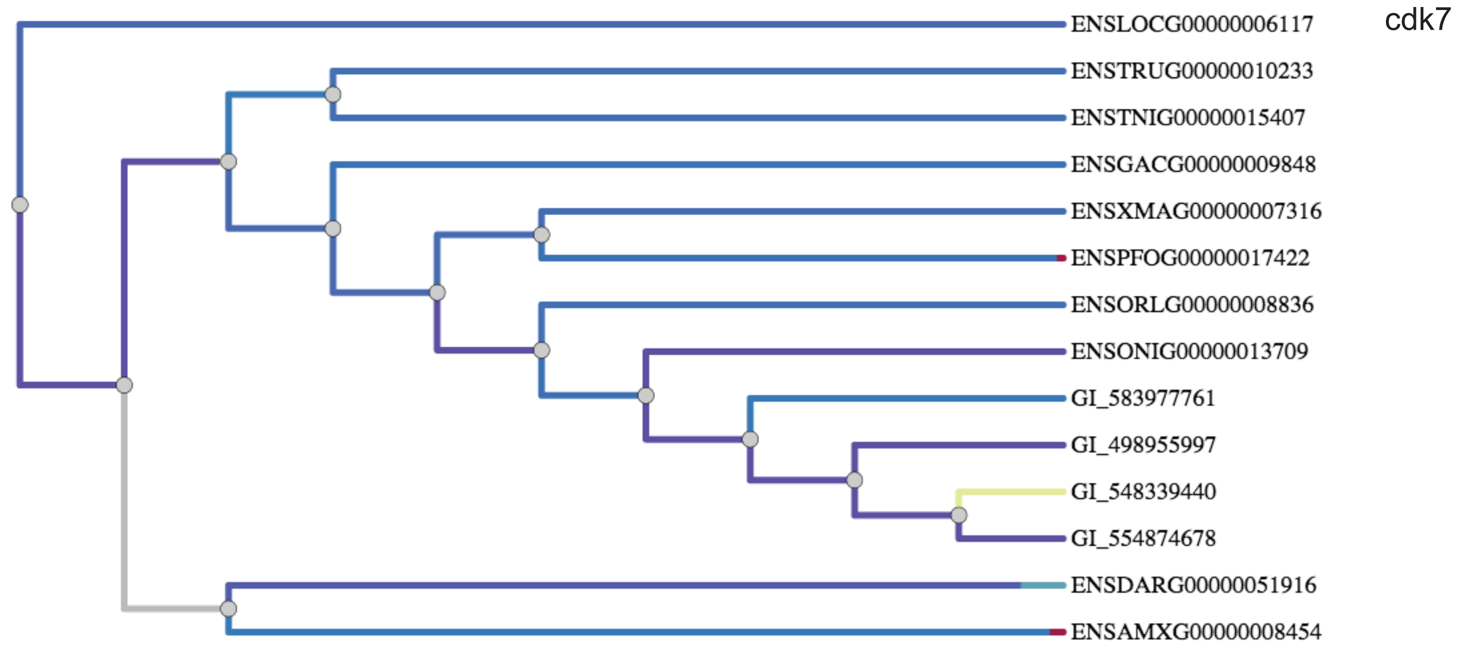
ENSLOCG00000006117
cdk7
ENSTRUG00000010233
ENSTNIG00000015407
ENSGACG00000009848
ENSXMAG00000007316
ENSPFOG00000017422
ENSORLG00000008836
ENSONIG00000013709
GI_583977761
GI_498955997
GI_548339440
GI_554874678
ENSDARG00000051916
ENSAMXG00000008454

Figure S3

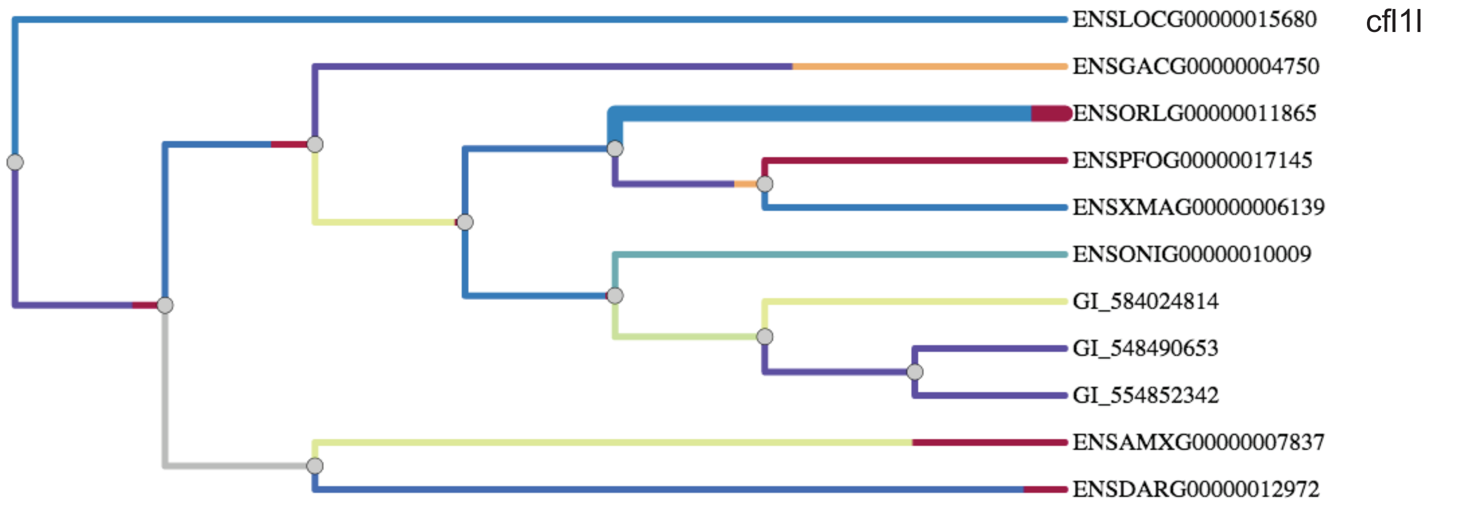
ENSLOCG00000015680
ENSGACG00000004750
ENSORLG00000011865
ENSPFOG00000017145
ENSXMAG00000006139
ENSONIG00000010009
GI_584024814
GI_548490653
GI_554852342
ENSAMXG00000007837
ENSDARG00000012972
cfl1l

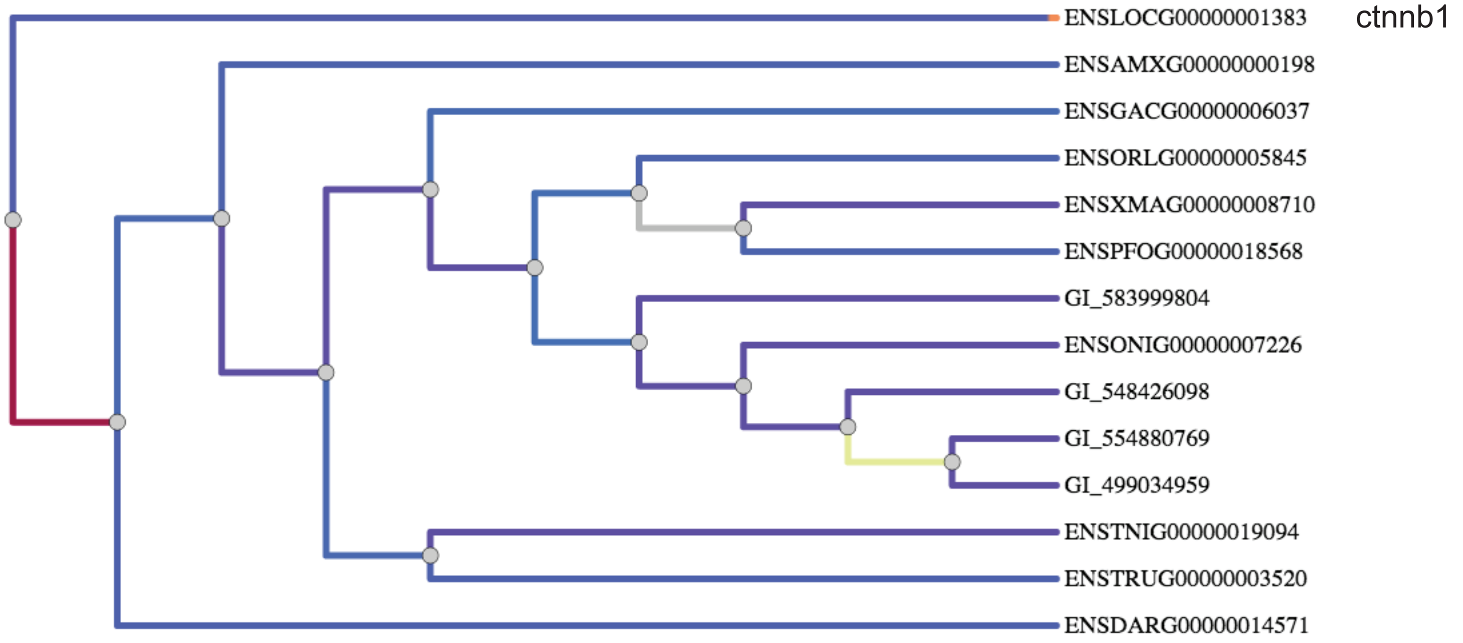
ENSLOCG00000001383
ENSAMXG00000000198
ENSGACG00000006037
ENSORLG00000005845
ENSXMAG00000008710
ENSPFOG00000018568
GI_583999804
ENSONIG00000007226
GI_548426098
GI_554880769
GI_499034959
ENSTNIG00000019094
ENSTRUG00000003520
ENSDARG00000014571
ctnnb1

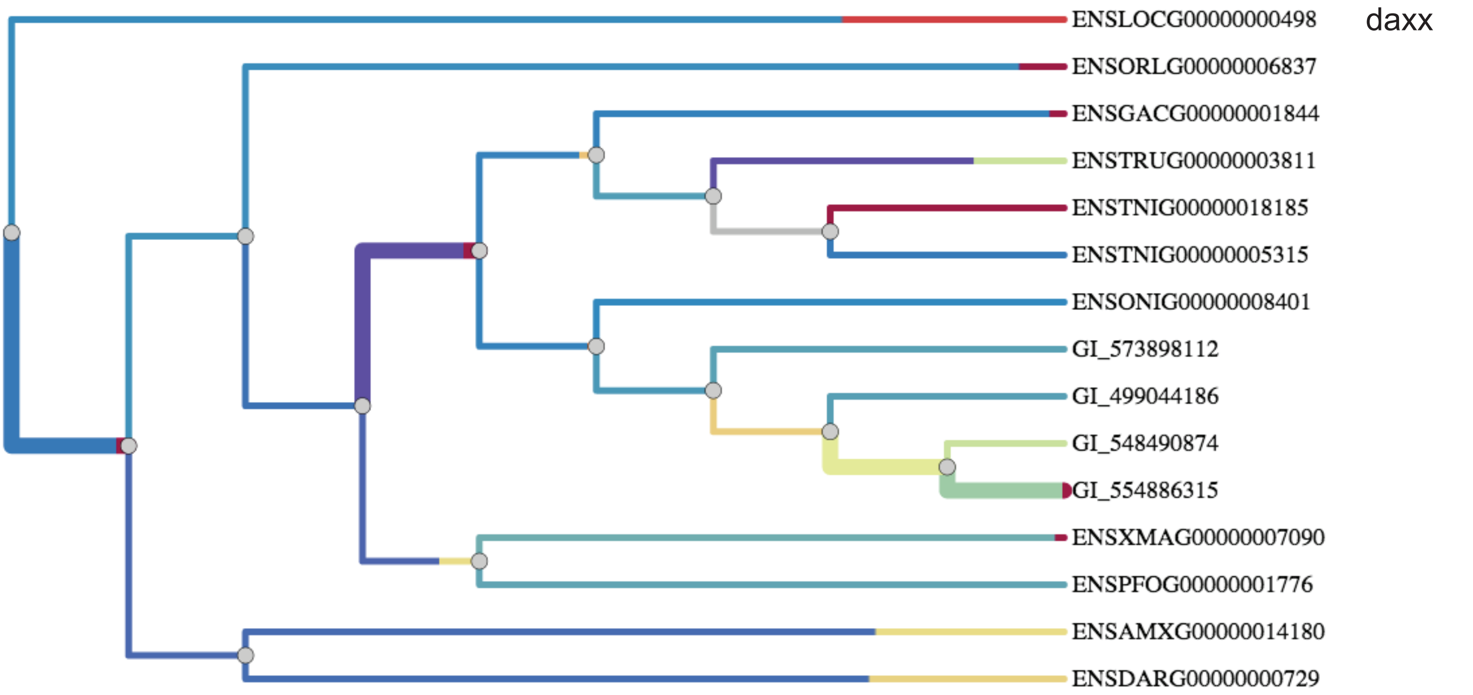
ENSLOCG00000000498
ENSORLG00000006837
ENSGACG00000001844
ENSTRUG00000003811
ENSTNIG00000018185
ENSTNIG00000005315
ENSONIG00000008401
GI_573898112
GI_499044186
GI_548490874
GI_554886315
ENSXMAG00000007090
ENSPFOG00000001776
ENSAMXG00000014180
ENSDARG00000000729
daxx

Figure S3

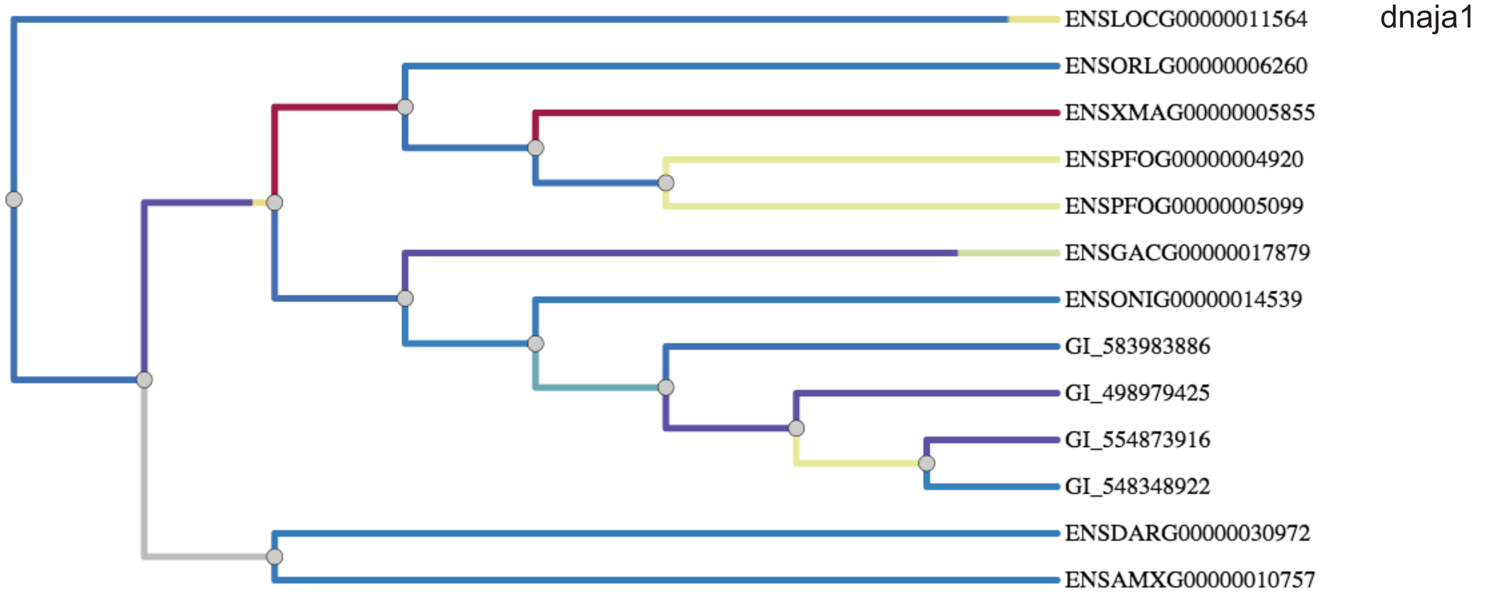

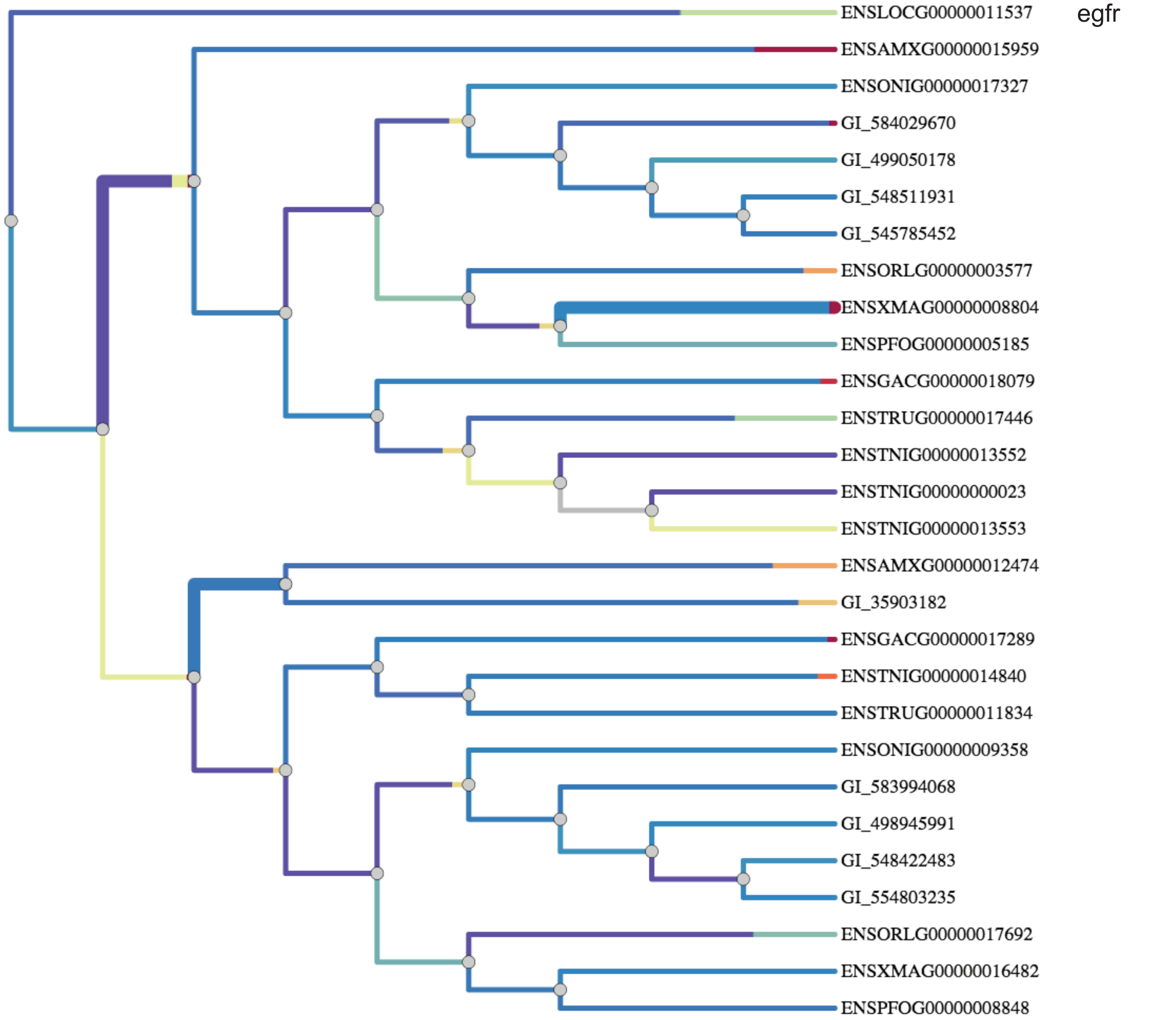

 T. Lorin, W. Salzburger & A. Böhne

Figure S3

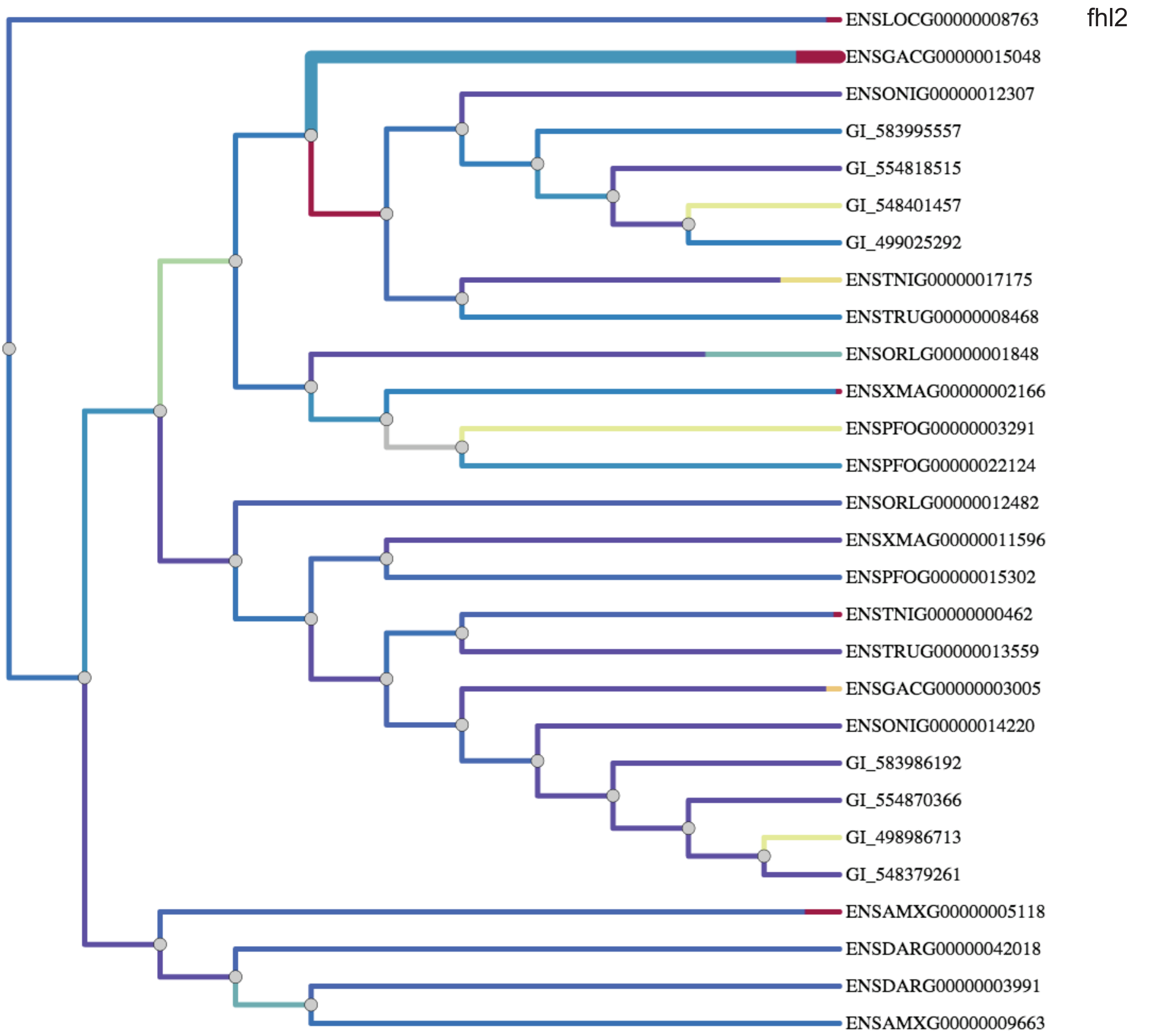

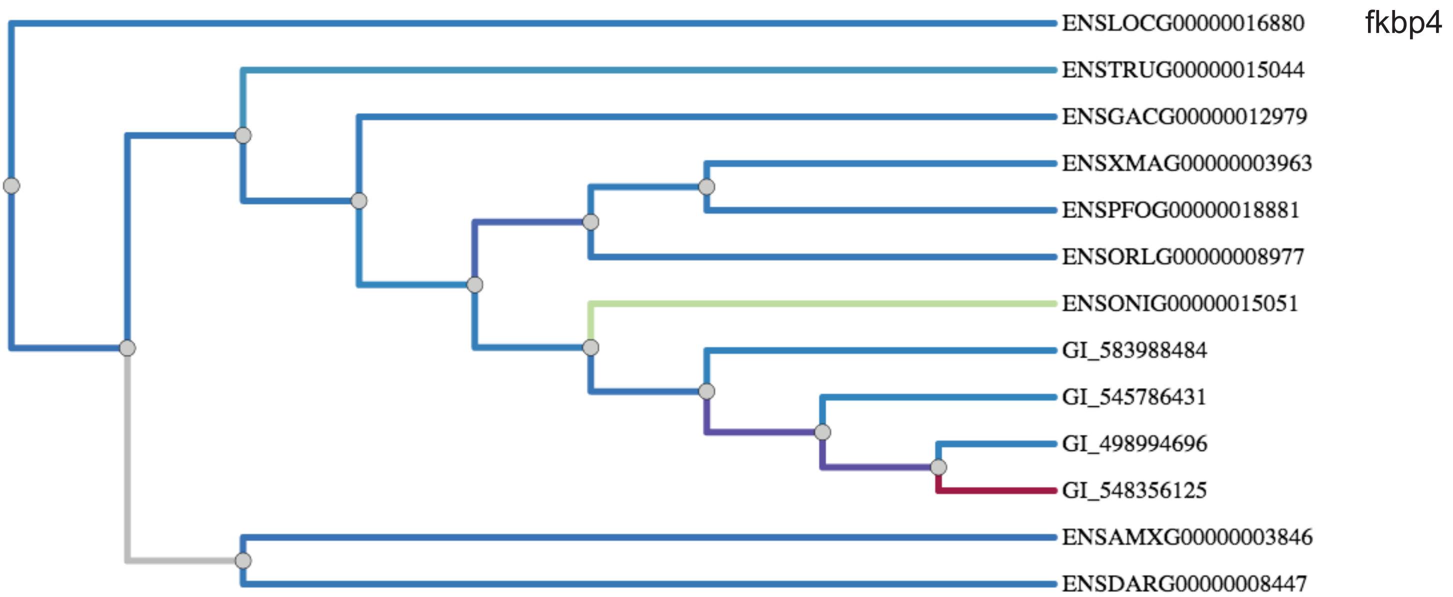

 T. Lorin, W. Salzburger & A. Böhne

Figure S3

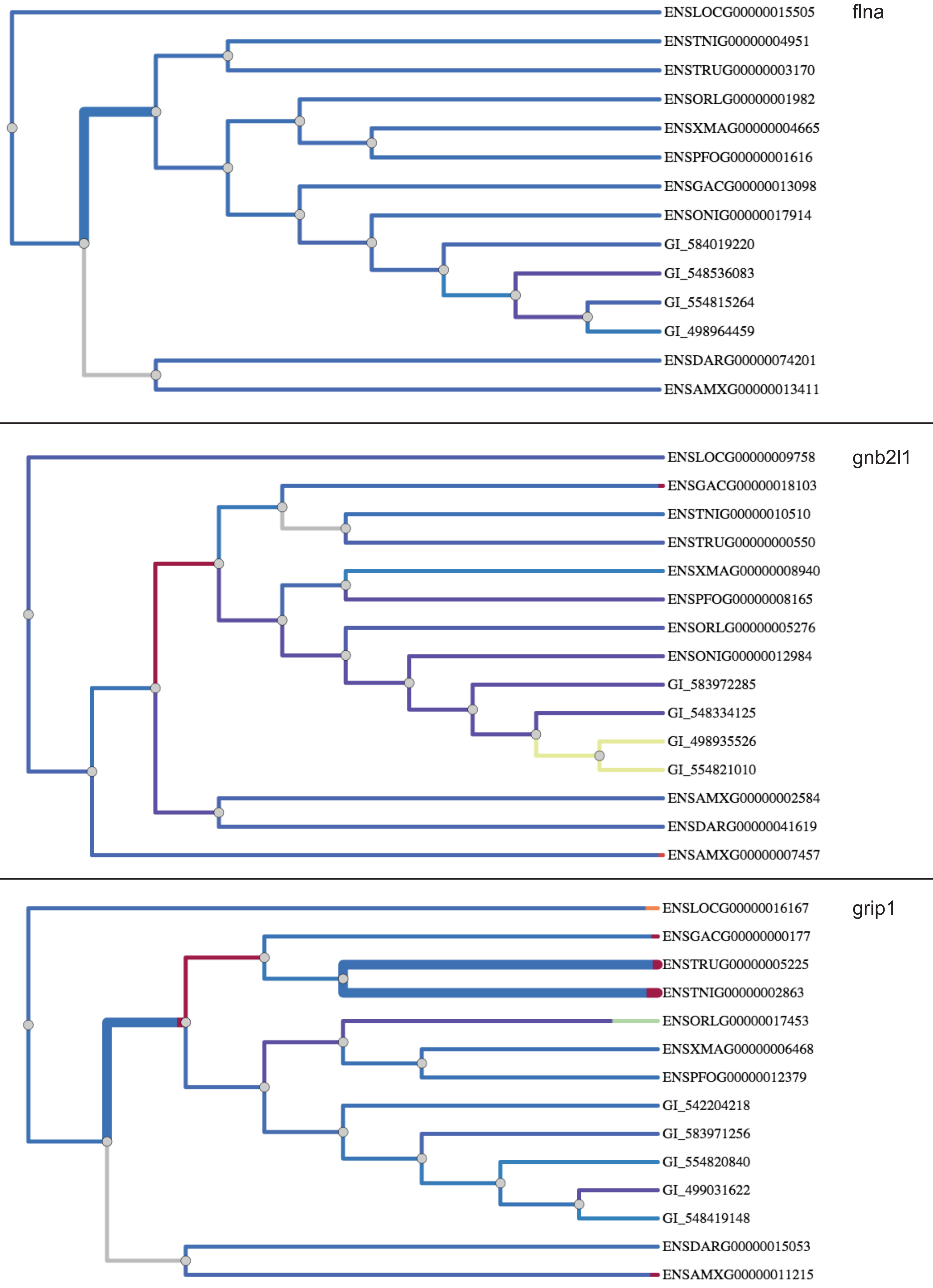

flna
ENSLOCG00000015505
ENSTNIG00000004951
ENSTRUG00000003170
ENSORLG00000001982
ENSXMAG00000004665
ENSPFOG00000001616
ENSGACG00000013098
ENSONIG00000017914
GI_584019220
GI_548536083
GI_554815264
GI_498964459
ENSDARG00000074201
ENSAMXG00000013411
gnb2l1
ENSLOCG00000009758
ENSGACG00000018103
ENSTNIG00000010510
ENSTRUG00000000550
ENSXMAG00000008940
ENSPFOG00000008165
ENSORLG00000005276
ENSONIG00000012984
GI_583972285
GI_548334125
GI_498935526
GI_554821010
ENSAMXG00000002584
ENSDARG00000041619
ENSAMXG00000007457
grip1
ENSLOCG00000016167
ENSGACG00000000177
ENSTRUG00000005225
ENSTNIG00000002863
ENSORLG00000017453
ENSXMAG00000006468
ENSPFOG00000012379
GI_542204218
GI_583971256
GI_554820840
GI_499031622
GI_548419148
ENSDARG00000015053
ENSAMXG00000011215

Figure S3

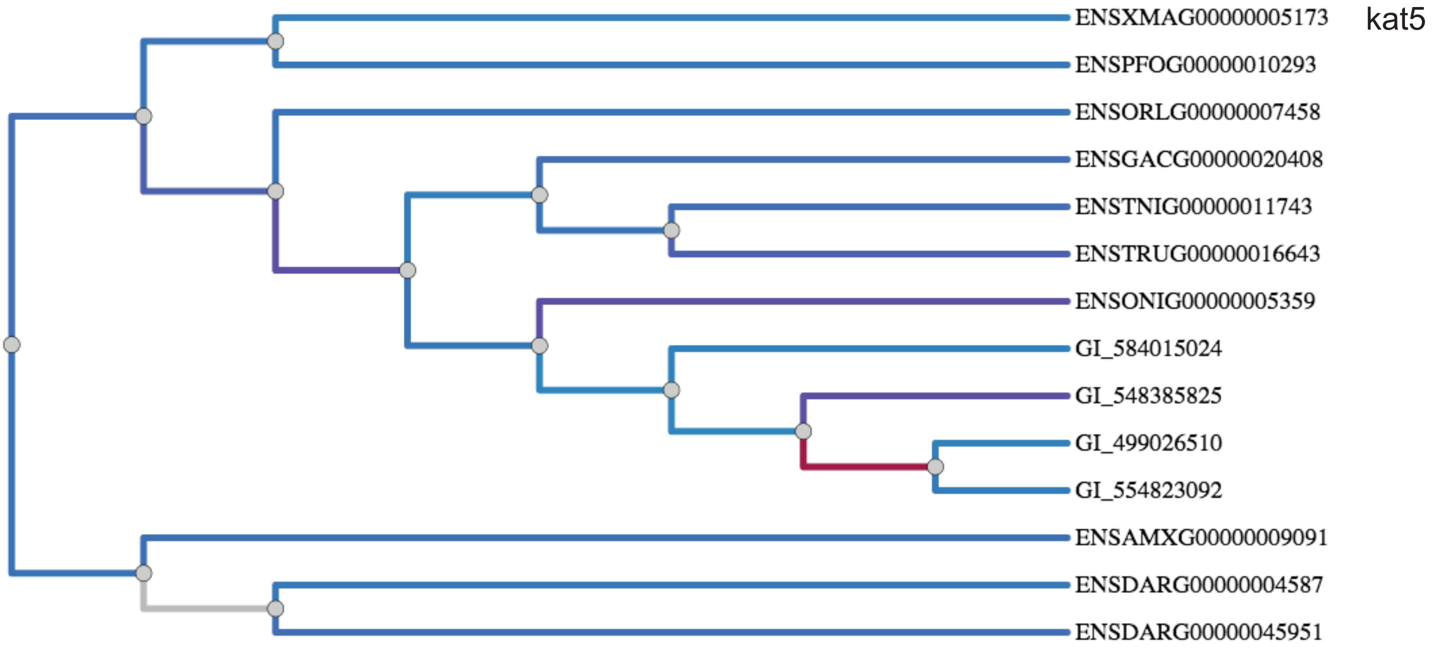
ENSXMAG00000005173
ENSPFOG00000010293
ENSORLG00000007458
ENSGACG00000020408
ENSTNIG00000011743
ENSTRUG00000016643
ENSONIG00000005359
GI_584015024
GI_548385825
GI_499026510
GI_554823092
ENSAMXG00000009091
ENSDARG00000004587
ENSDARG00000045951
kat5

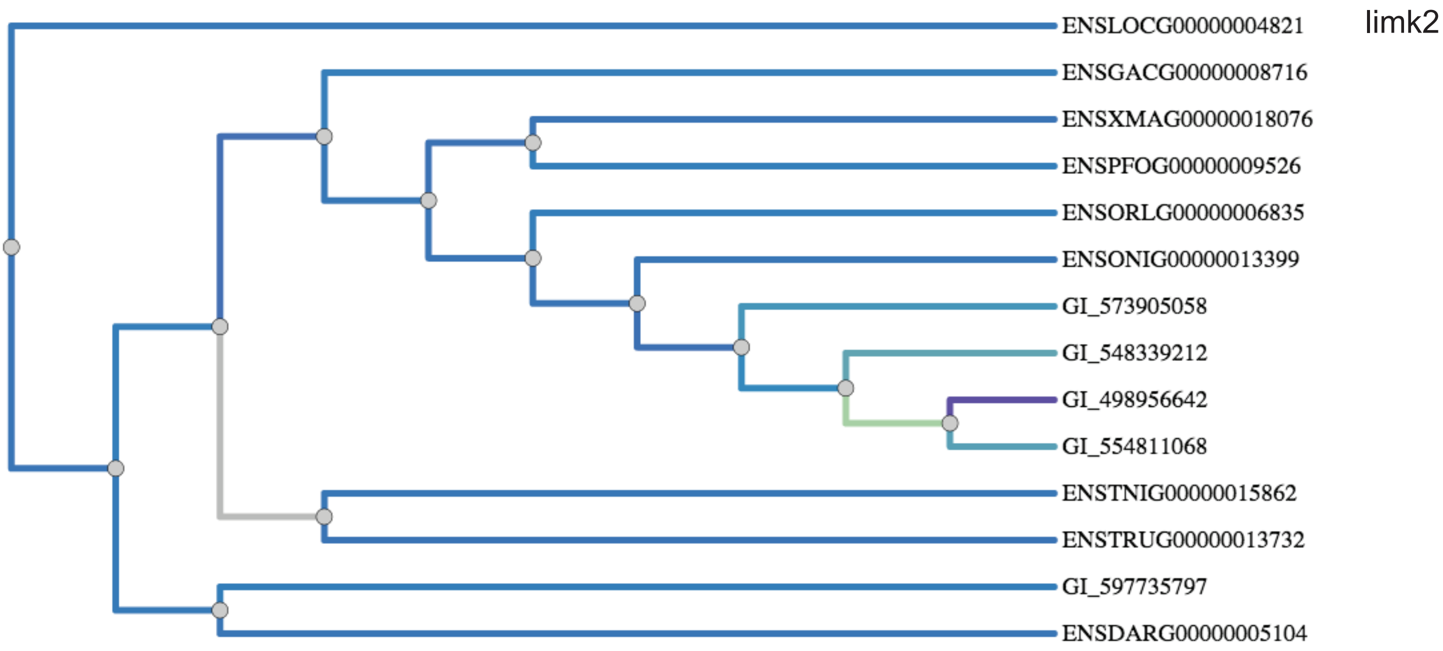
ENSLOCG00000004821
ENSGACG00000008716
ENSXMAG00000018076
ENSPFOG00000009526
ENSORLG00000006835
ENSONIG00000013399
GI_573905058
GI_548339212
GI_498956642
GI_554811068
ENSTNIG00000015862
ENSTRUG00000013732
GI_597735797
ENSDARG00000005104
limk2

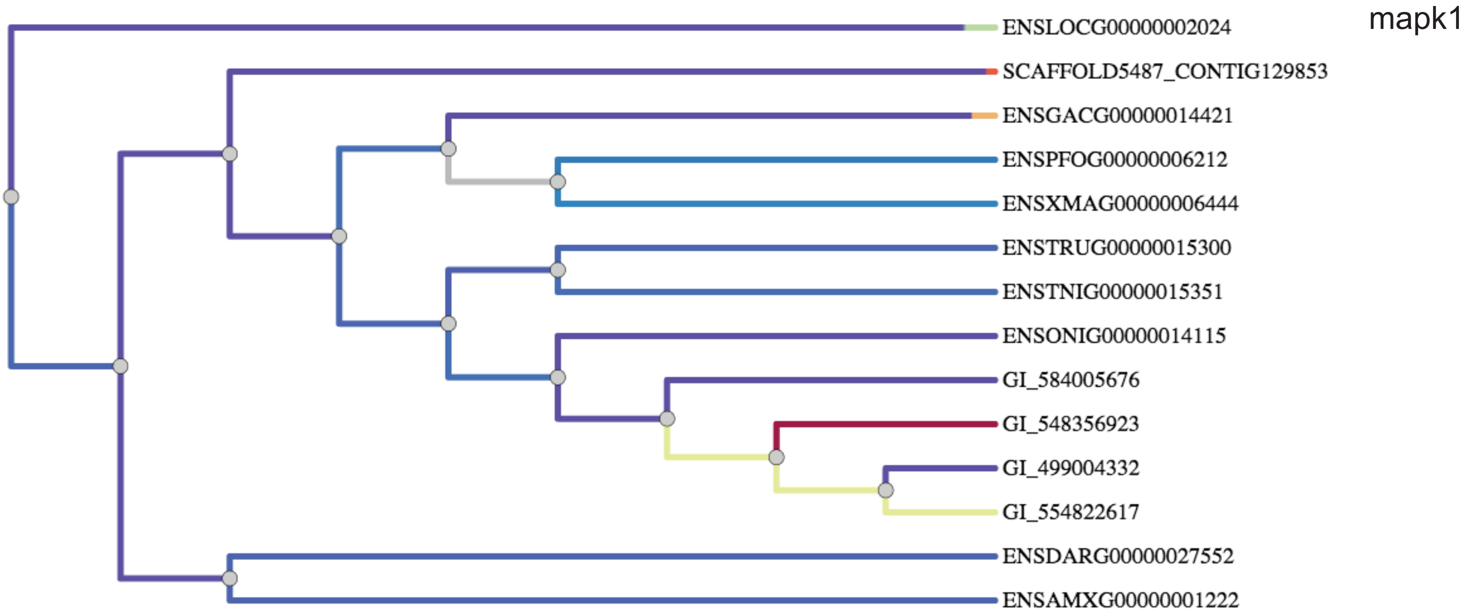
ENSLOCG00000002024
SCAFFOLD5487_CONTIG129853
ENSGACG00000014421
ENSPFOG00000006212
ENSXMAG00000006444
ENSTRUG00000015300
ENSTNIG00000015351
ENSONIG00000014115
GI_584005676
GI_548356923
GI_499004332
GI_554822617
ENSDARG00000027552
ENSAMXG00000001222
mapk1

Figure S3

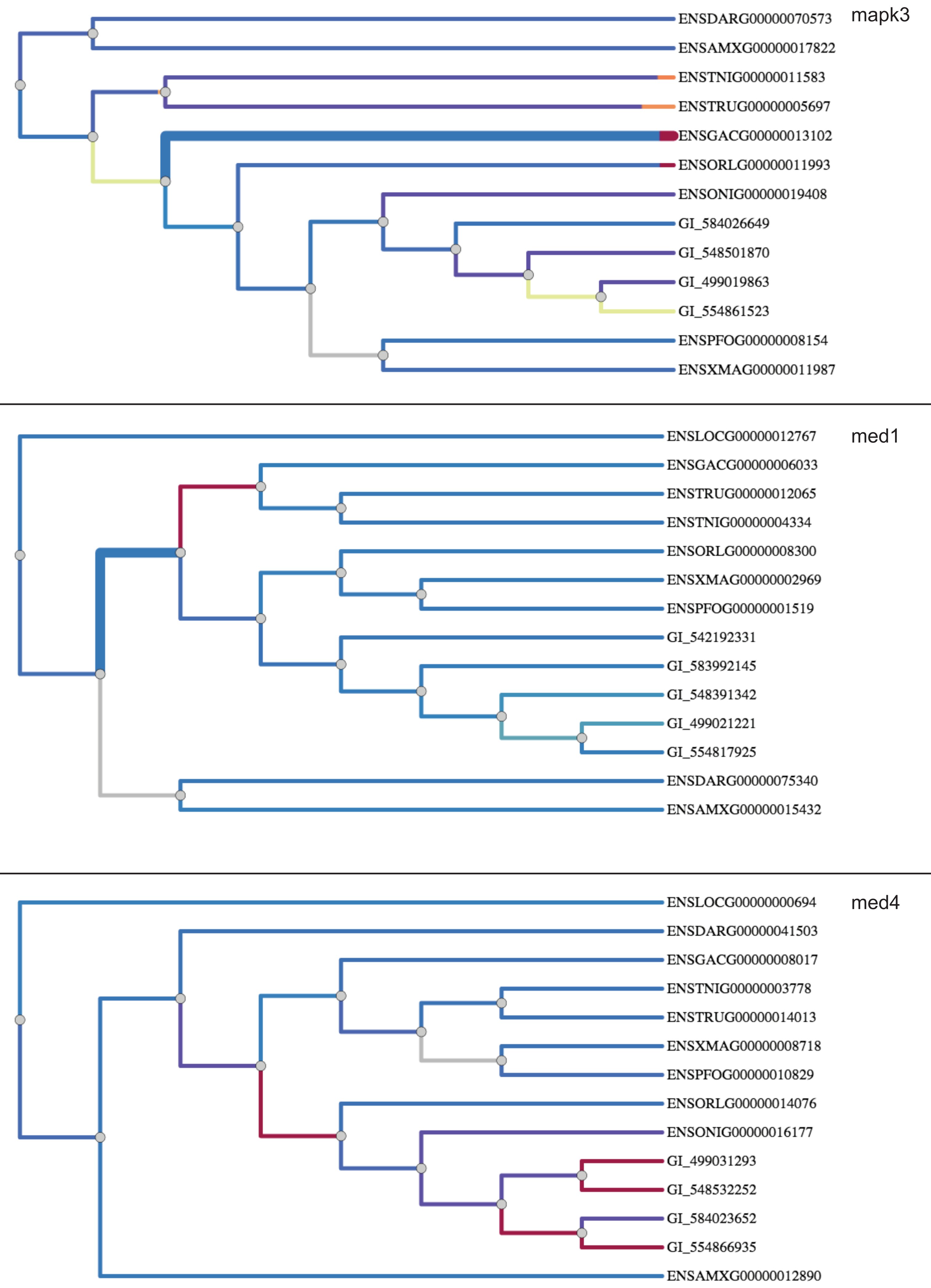
mapk3
ENSDARG00000070573
ENSAMXG00000017822
ENSTNIG00000011583
ENSTRUG00000005697
ENSGACG00000013102
ENSORLG00000011993
ENSONIG00000019408
GI_584026649
GI_548501870
GI_499019863
GI_554861523
ENSPFOG00000008154
ENSXMAG00000011987
med1
ENSLOCG00000012767
ENSGACG00000006033
ENSTRUG00000012065
ENSTNIG00000004334
ENSORLG00000008300
ENSXMAG00000002969
ENSPFOG00000001519
GI_542192331
GI_583992145
GI_548391342
GI_499021221
GI_554817925
ENSDARG00000075340
ENSAMXG00000015432
med4
ENSLOCG00000000694
ENSDARG00000041503
ENSGACG00000008017
ENSTNIG00000003778
ENSTRUG00000014013
ENSXMAG00000008718
ENSPFOG00000010829
ENSORLG00000014076
ENSONIG00000016177
GI_499031293
GI_548532252
GI_584023652
GI_554866935
ENSAMXG00000012890

Figure S3

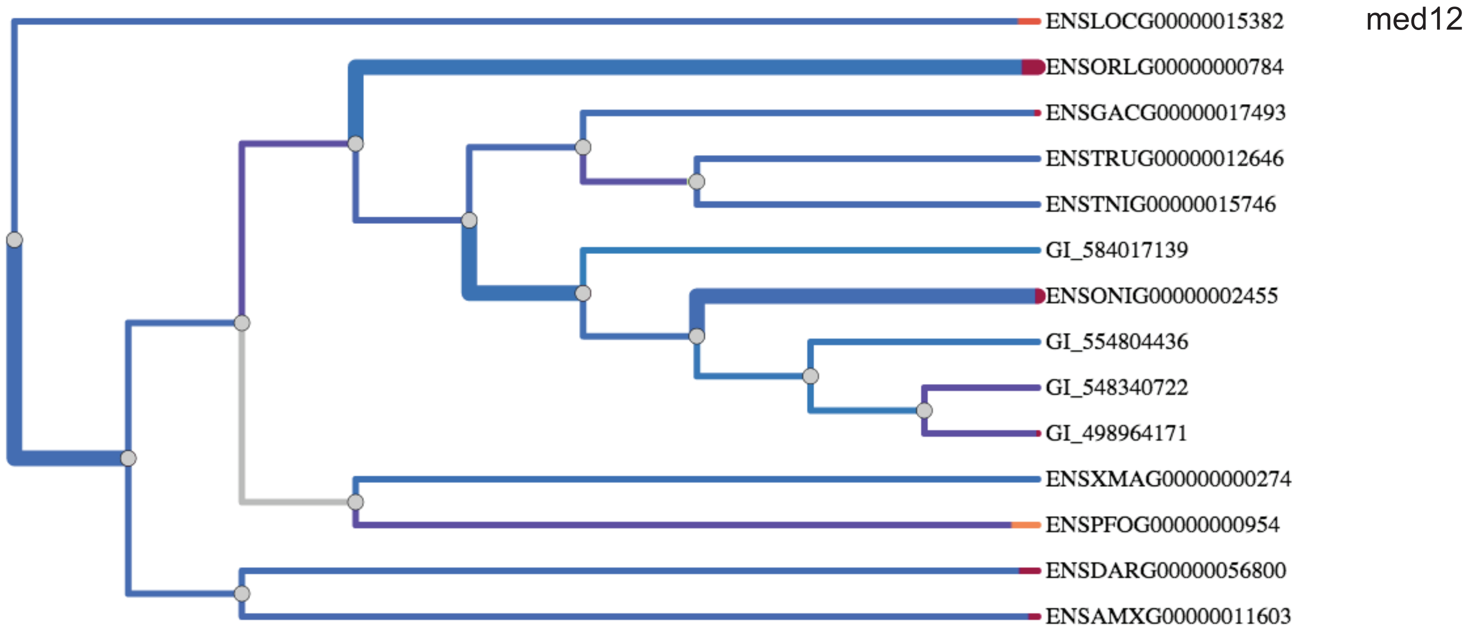

ENSLOCG00000015382
ENSORLG00000000784
ENSGACG00000017493
ENSTRUG00000012646
ENSTNIG00000015746
GI_584017139
ENSONIG00000002455
GI_554804436
GI_548340722
GI_498964171
ENSXMAG00000000274
ENSPFOG00000000954
ENSDARG00000056800
ENSAMXG00000011603
med12

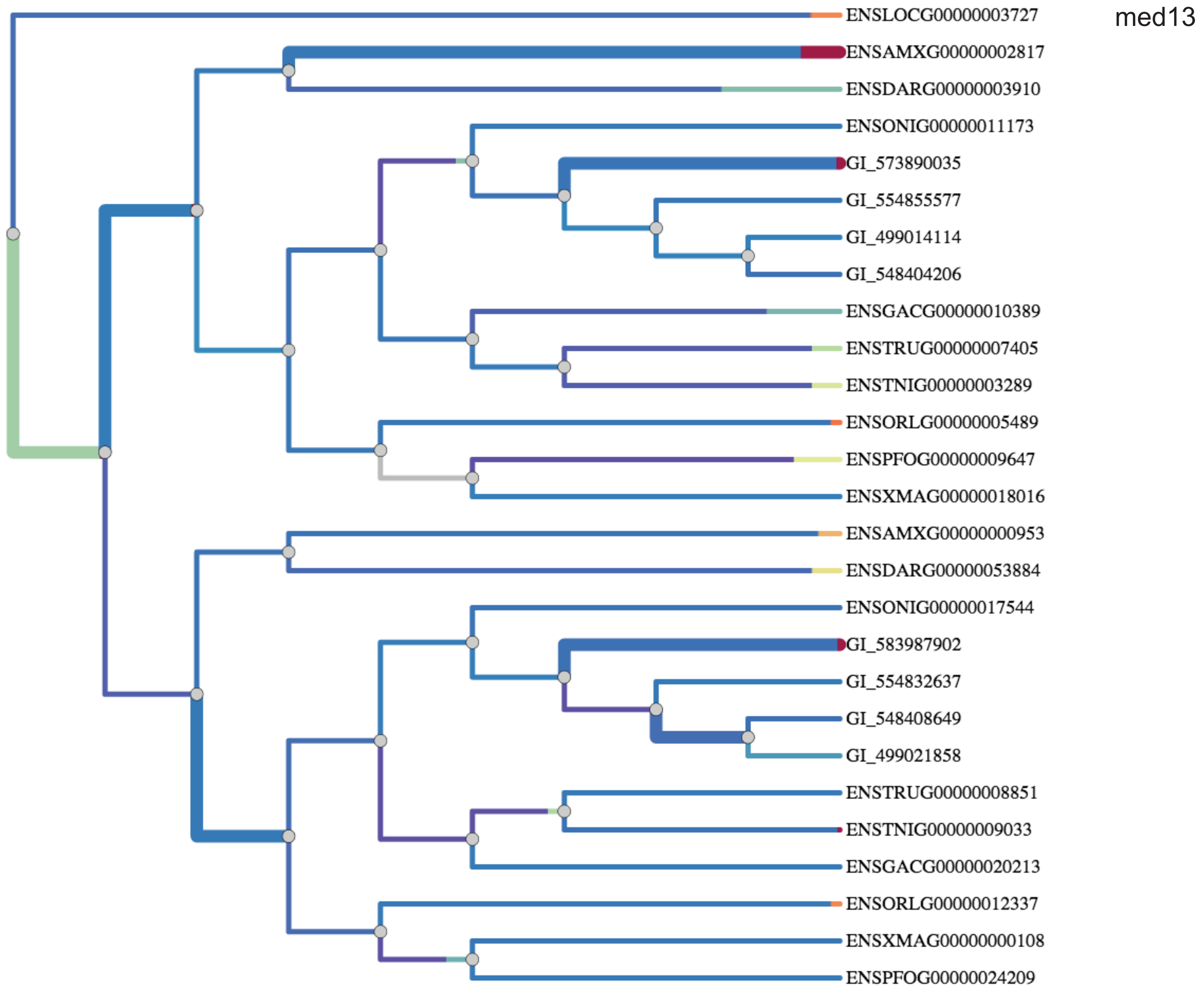

ENSLOCG00000003727
ENSAMXG00000002817
ENSDARG00000003910
ENSONIG00000011173
GI_573890035
GI_554855577
GI_499014114
GI_548404206
ENSGACG00000010389
ENSTRUG00000007405
ENSTNIG00000003289
ENSORLG00000005489
ENSPFOG00000009647
ENSXMAG00000018016
ENSAMXG00000000953
ENSDARG00000053884
ENSONIG00000017544
GI_583987902
GI_554832637
GI_548408649
GI_499021858
ENSTRUG00000008851
ENSTNIG00000009033
ENSGACG00000020213
ENSORLG00000012337
ENSXMAG00000000108
ENSPFOG00000024209
med13

Figure S3

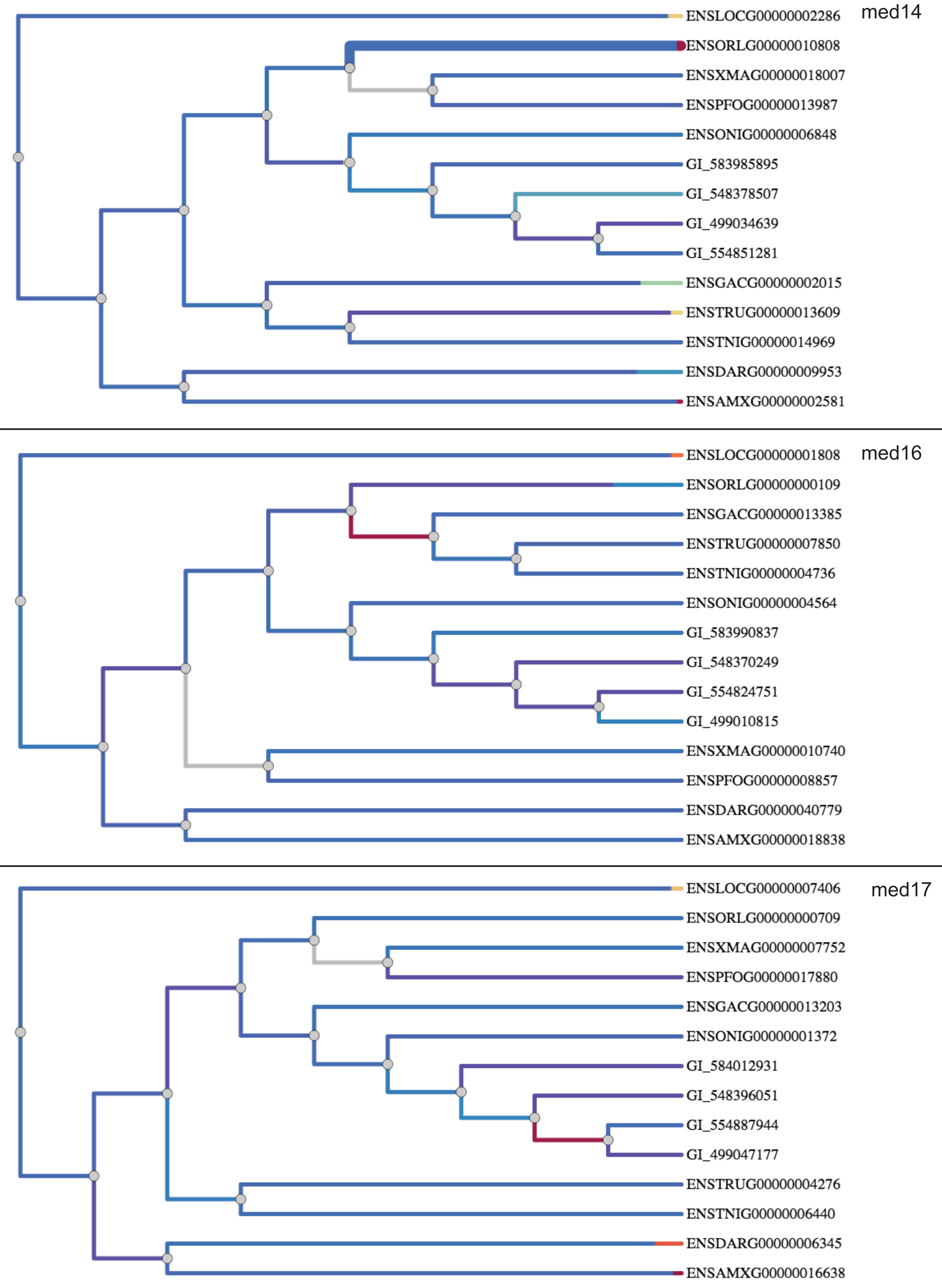
med14
ENSLOCG00000002286
ENSORLG00000010808
ENSXMAG00000018007
ENSPFOG00000013987
ENSONIG00000006848
GI_583985895
GI_548378507
GI_499034639
GI_554851281
ENSGACG00000002015
ENSTRUG00000013609
ENSTNIG00000014969
ENSDARG00000009953
ENSAMXG00000002581
med16
ENSLOCG00000001808
ENSORLG00000000109
ENSGACG00000013385
ENSTRUG00000007850
ENSTNIG00000004736
ENSONIG00000004564
GI_583990837
GI_548370249
GI_554824751
GI_499010815
ENSXMAG00000010740
ENSPFOG00000008857
ENSDARG00000040779
ENSAMXG00000018838
med17
ENSLOCG00000007406
ENSORLG00000000709
ENSXMAG00000007752
ENSPFOG00000017880
ENSGACG00000013203
ENSONIG00000001372
GI_584012931
GI_548396051
GI_554887944
GI_499047177
ENSTRUG00000004276
ENSTNIG00000006440
ENSDARG00000006345
ENSAMXG00000016638

Figure S3

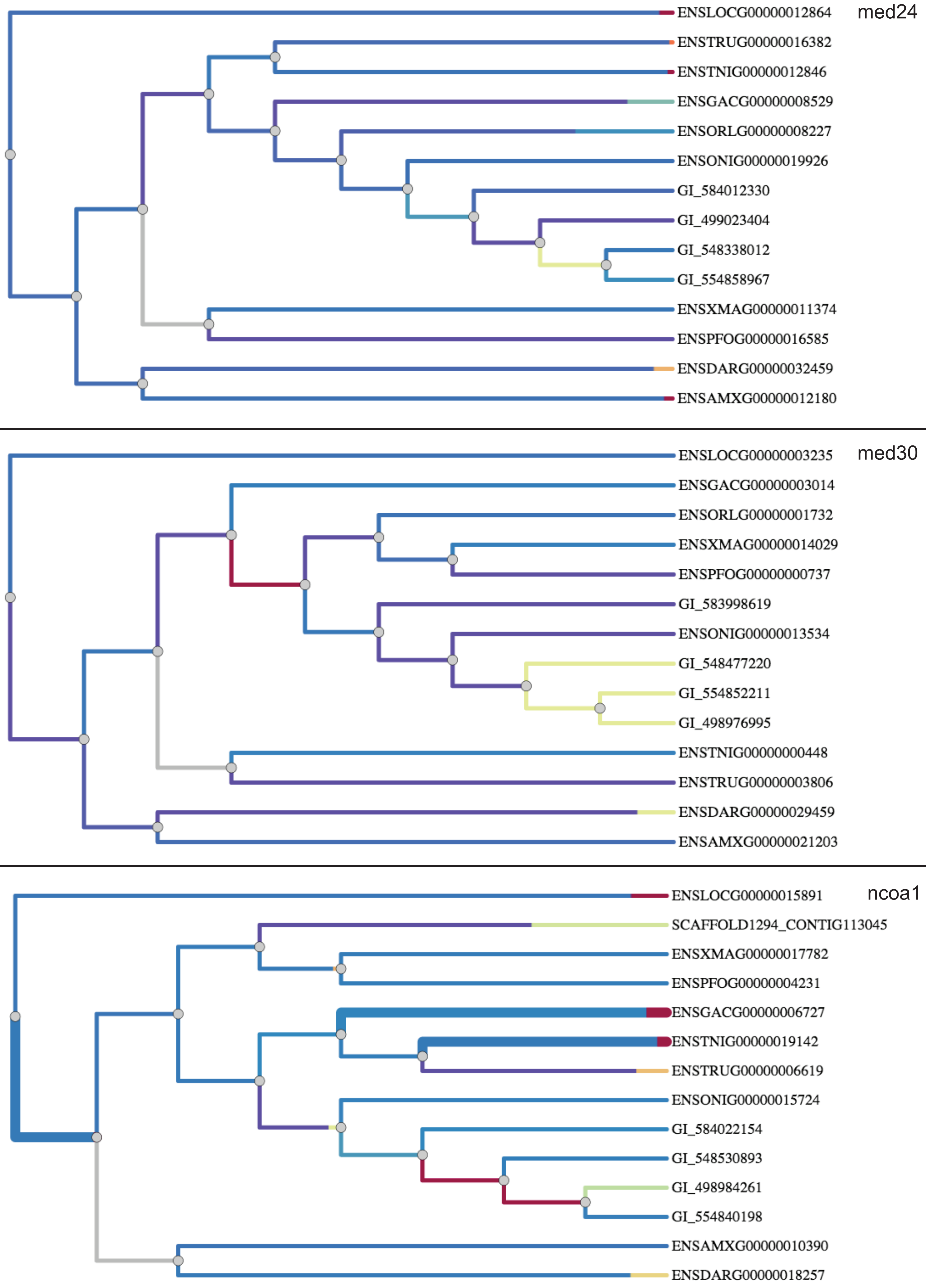
med24
ENSLOCG00000012864
ENSTRUG00000016382
ENSTNIG00000012846
ENSGACG00000008529
ENSORLG00000008227
ENSONIG00000019926
GI_584012330
GI_499023404
GI_548338012
GI_554858967
ENSXMAG00000011374
ENSPFOG00000016585
ENSDARG00000032459
ENSAMXG00000012180
med30
ENSLOCG00000003235
ENSGACG00000003014
ENSORLG00000001732
ENSXMAG00000014029
ENSPFOG00000000737
GI_583998619
ENSONIG00000013534
GI_548477220
GI_554852211
GI_498976995
ENSTNIG00000000448
ENSTRUG00000003806
ENSDARG00000029459
ENSAMXG00000021203
ncoa1
ENSLOCG00000015891
SCAFFOLD1294_CONTIG113045
ENSXMAG00000017782
ENSPFOG00000004231
ENSGACG00000006727
ENSTNIG00000019142
ENSTRUG00000006619
ENSONIG00000015724
GI_584022154
GI_548530893
GI_498984261
GI_554840198
ENSAMXG00000010390
ENSDARG00000018257

Figure S3

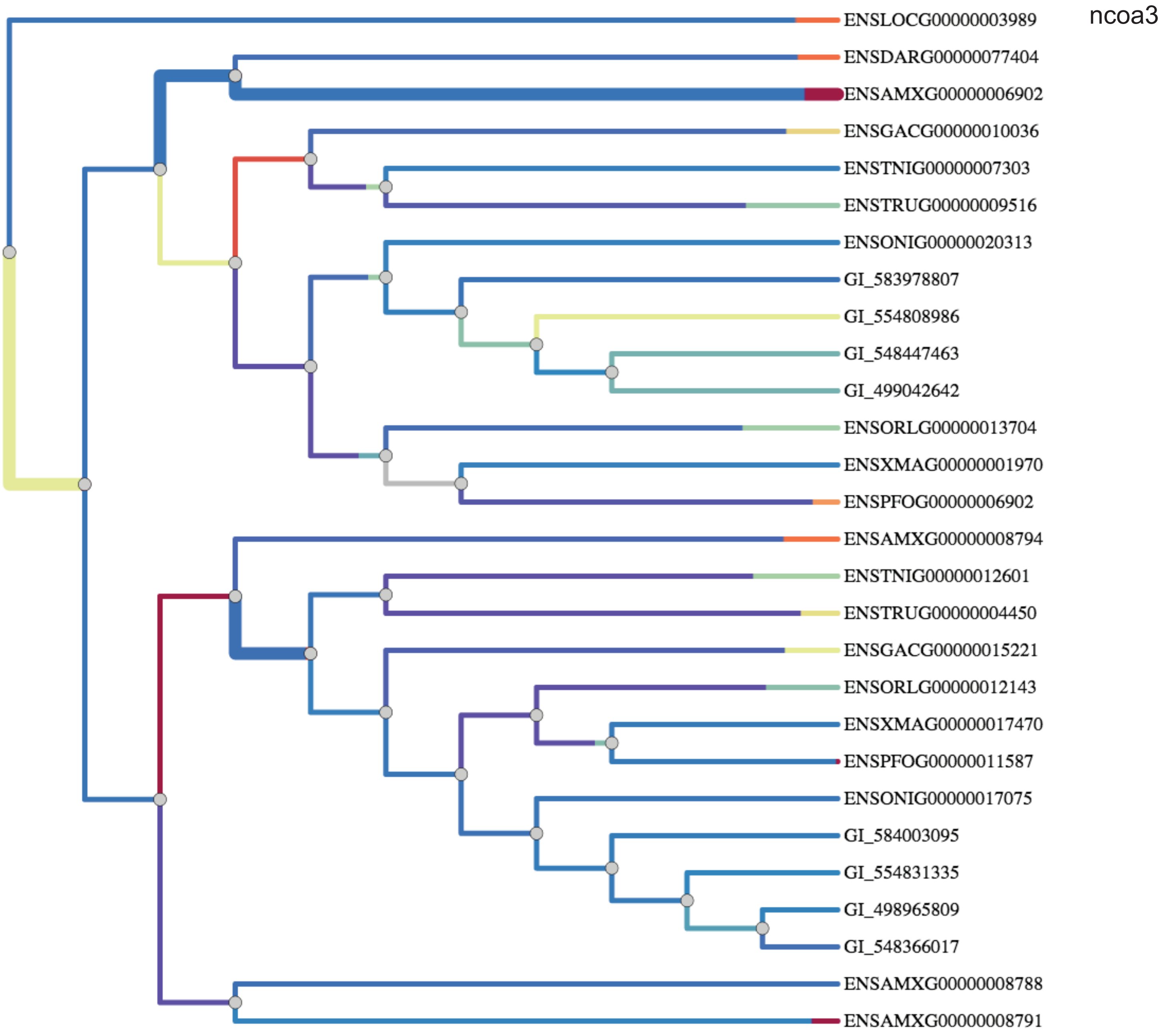
ncoa3
ENSLOCG00000003989
ENSDARG00000077404
ENSAMXG00000006902
ENSGACG00000010036
ENSTNIG00000007303
ENSTRUG00000009516
ENSONIG00000020313
GI_583978807
GI_554808986
GI_548447463
GI_499042642
ENSORLG00000013704
ENSXMAG00000001970
ENSPFOG00000006902
ENSAMXG00000008794
ENSTNIG00000012601
ENSTRUG00000004450
ENSGACG00000015221
ENSORLG00000012143
ENSXMAG00000017470
ENSPFOG00000011587
ENSONIG00000017075
GI_584003095
GI_554831335
GI_498965809
GI_548366017
ENSAMXG00000008788
ENSAMXG00000008791

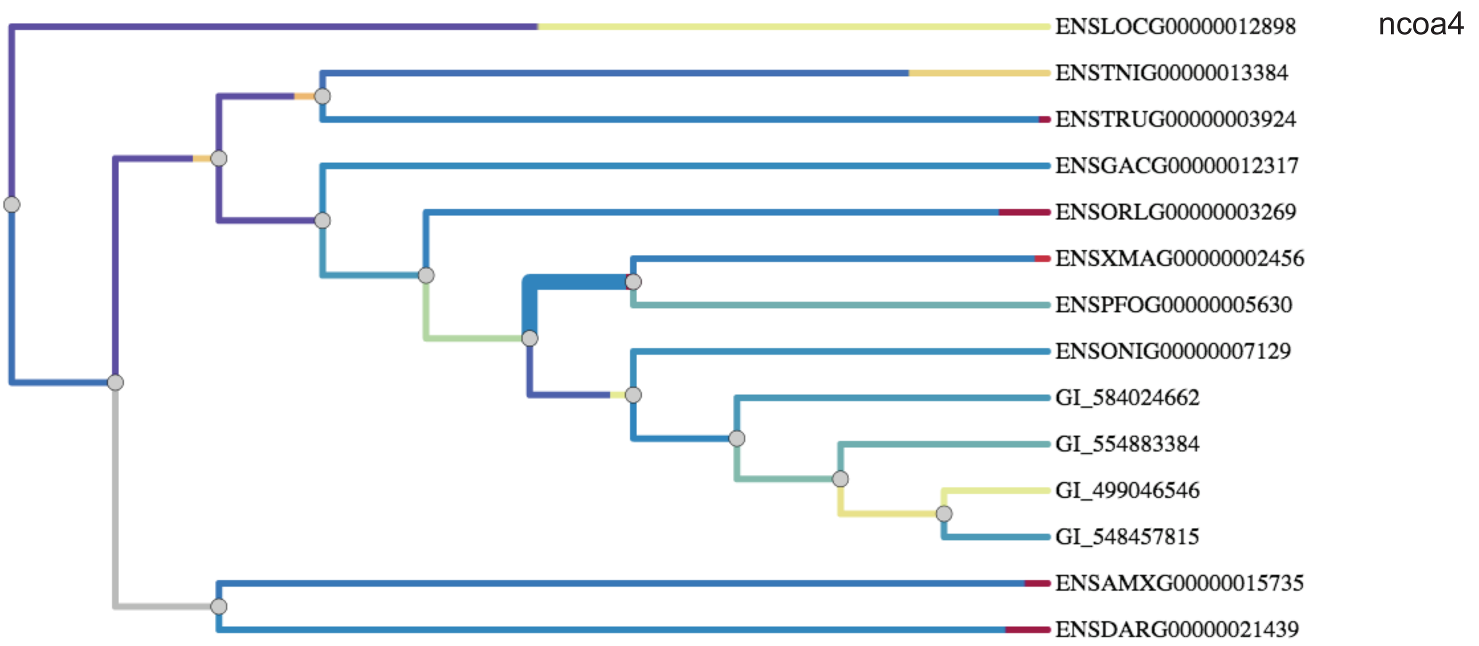
ncoa4
ENSLOCG00000012898
ENSTNIG00000013384
ENSTRUG00000003924
ENSGACG00000012317
ENSORLG00000003269
ENSXMAG00000002456
ENSPFOG00000005630
ENSONIG00000007129
GI_584024662
GI_554883384
GI_499046546
GI_548457815
ENSAMXG00000015735
ENSDARG00000021439

Figure S3

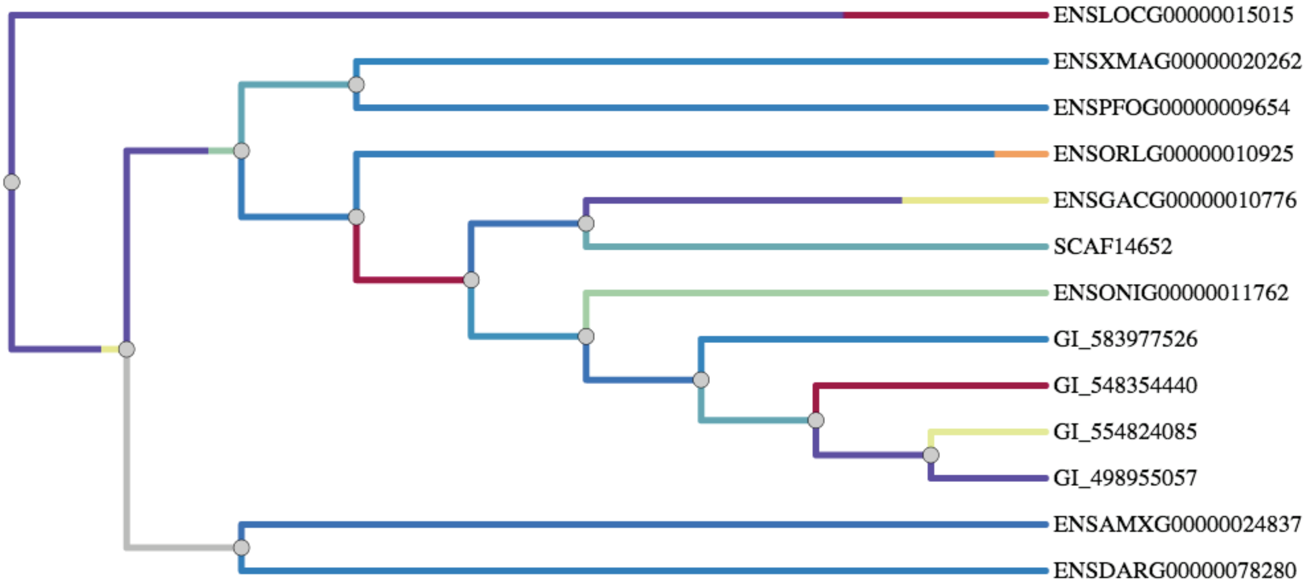
ENSLOCG00000015015
ENSXMAG00000020262
ENSPFOG00000009654
ENSORLG00000010925
ENSGACG00000010776
SCAF14652
ENSONIG00000011762
GI_583977526
GI_548354440
GI_554824085
GI_498955057
ENSAMXG00000024837
ENSDARG00000078280
nkx3.1

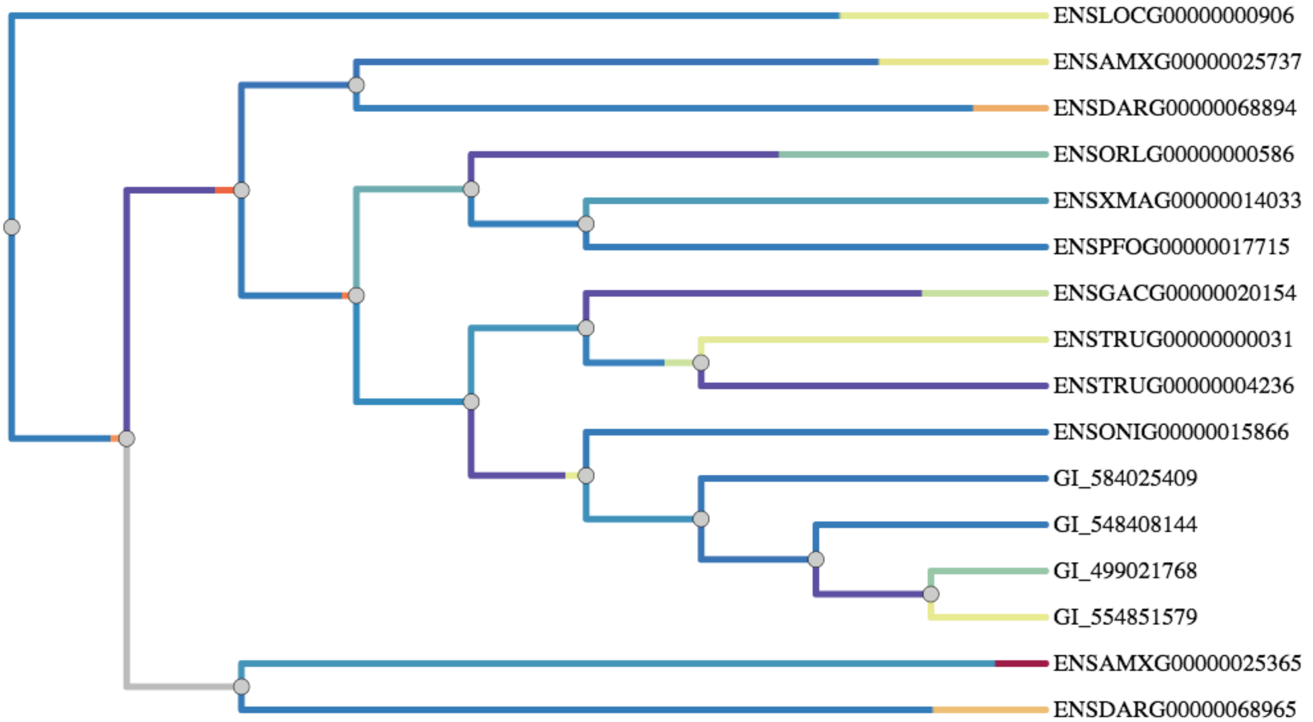
ENSLOCG00000000906
ENSAMXG00000025737
ENSDARG00000068894
ENSORLG00000000586
ENSXMAG00000014033
ENSPFOG00000017715
ENSGACG00000020154
ENSTRUG00000000031
ENSTRUG00000004236
ENSONIG00000015866
GI_584025409
GI_548408144
GI_499021768
GI_554851579
ENSAMXG00000025365
ENSDARG00000068965
nrip1

Figure S3

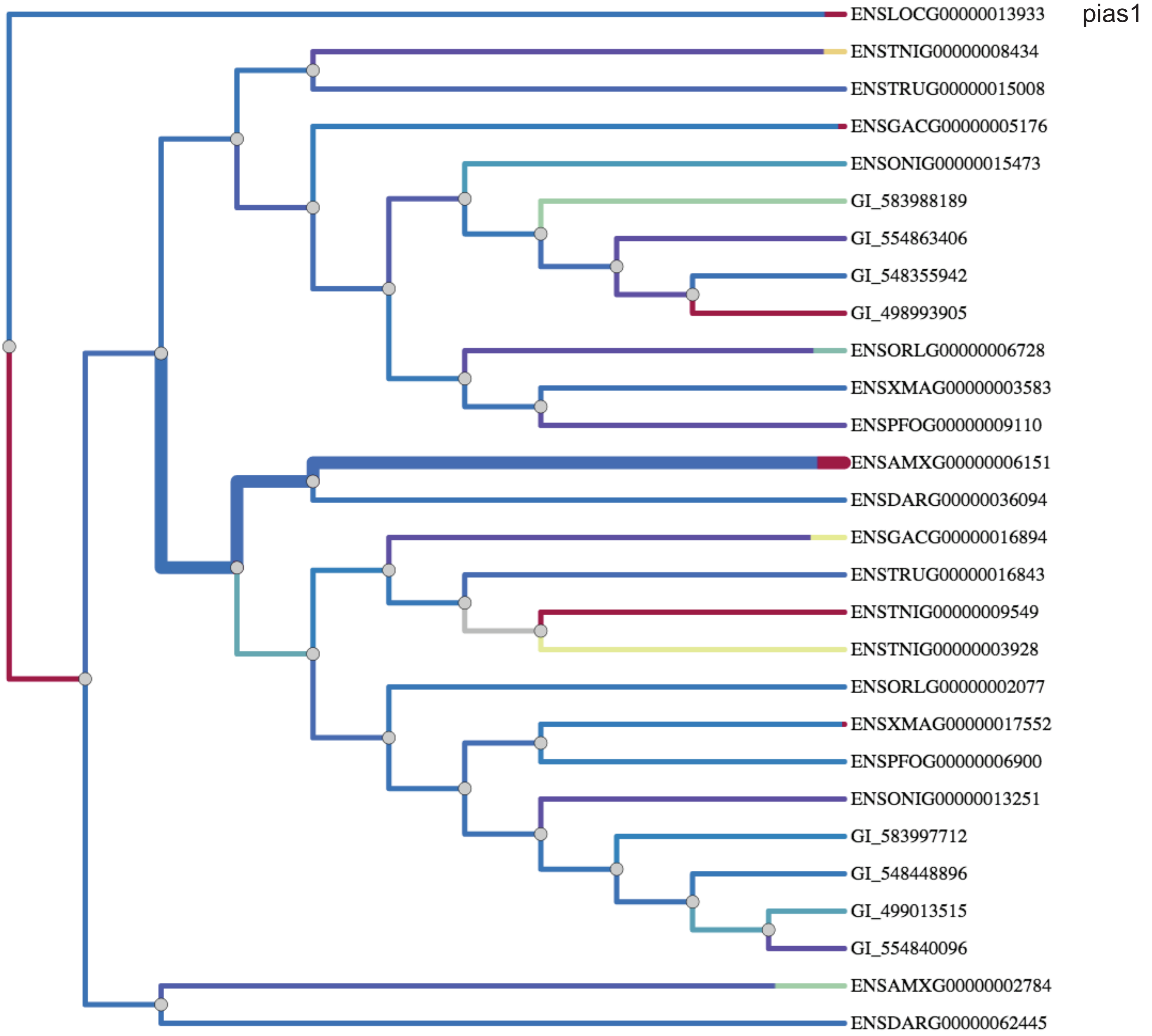
ENSLOCG00000013933
pias1
ENSTNIG00000008434
ENSTRUG00000015008
ENSGACG00000005176
ENSONIG00000015473
GI_583988189
GI_554863406
GI_548355942
GI_498993905
ENSORLG00000006728
ENSXMAG00000003583
ENSPFOG00000009110
ENSAMXG00000006151
ENSDARG00000036094
ENSGACG00000016894
ENSTRUG00000016843
ENSTNIG00000009549
ENSTNIG00000003928
ENSORLG00000002077
ENSXMAG00000017552
ENSPFOG00000006900
ENSONIG00000013251
GI_583997712
GI_548448896
GI_499013515
GI_554840096
ENSAMXG00000002784
ENSDARG00000062445

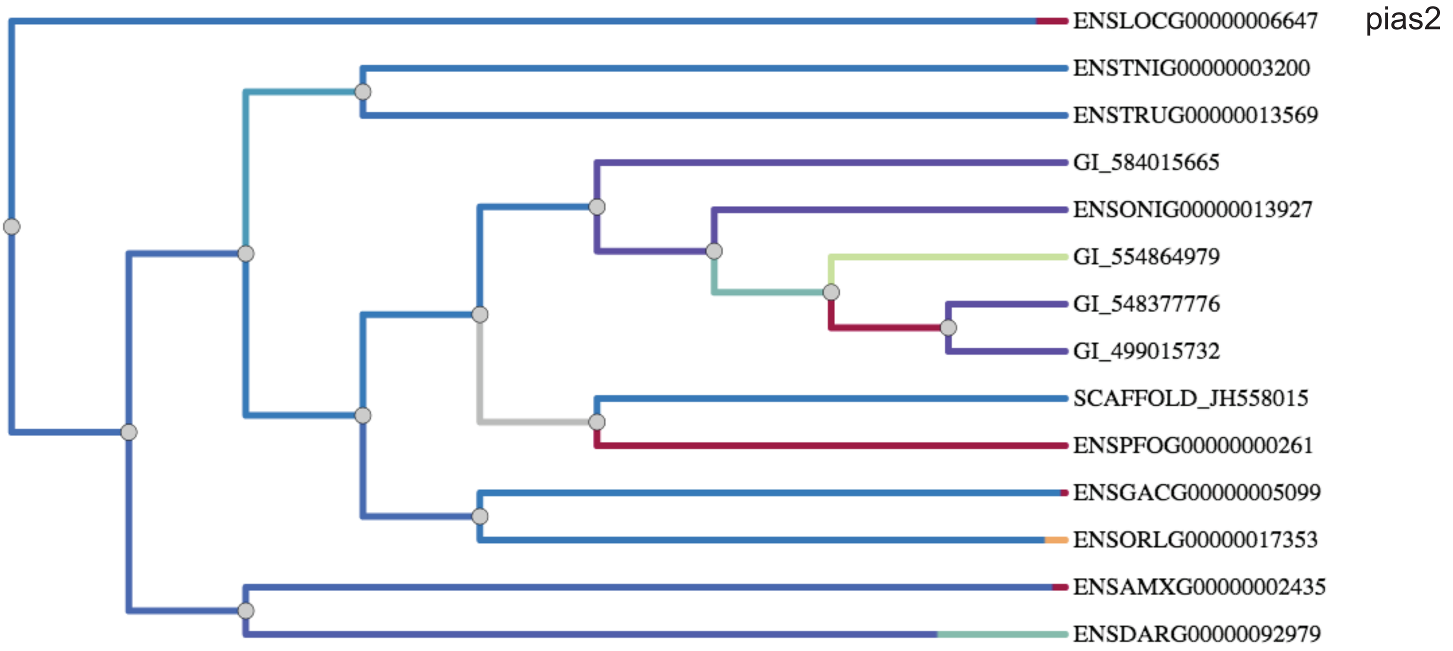
ENSLOCG00000006647
pias2
ENSTNIG00000003200
ENSTRUG00000013569
GI_584015665
ENSONIG00000013927
GI_554864979
GI_548377776
GI_499015732
SCAFFOLD_JH558015
ENSPFOG00000000261
ENSGACG00000005099
ENSORLG00000017353
ENSAMXG00000002435
ENSDARG00000092979

Figure S3

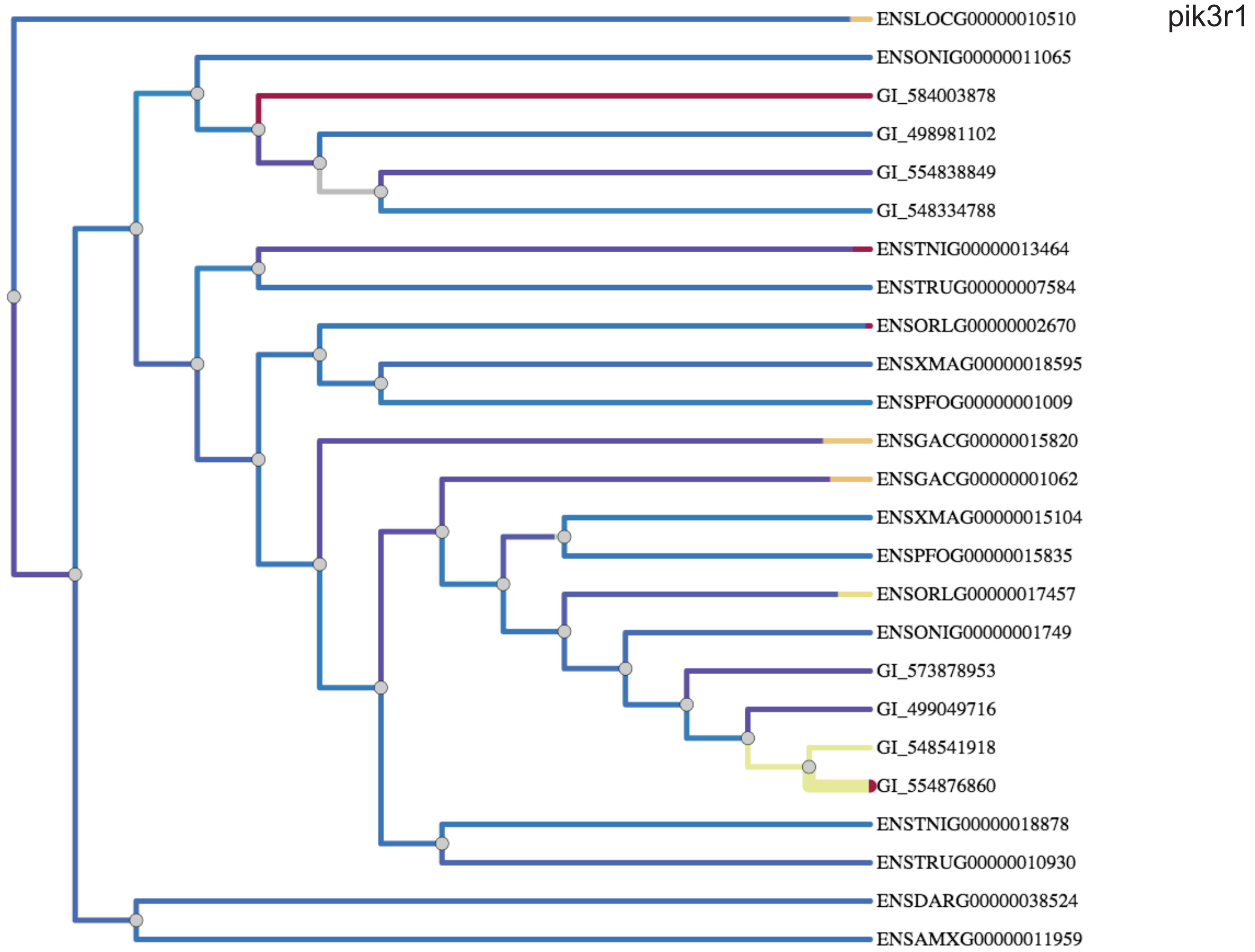
pik3r1
ENSLOCG00000010510
ENSONIG00000011065
GI_584003878
GI_498981102
GI_554838849
GI_548334788
ENSTNIG00000013464
ENSTRUG00000007584
ENSORLG00000002670
ENSXMAG00000018595
ENSPFOG00000001009
ENSGACG00000015820
ENSGACG00000001062
ENSXMAG00000015104
ENSPFOG00000015835
ENSORLG00000017457
ENSONIG00000001749
GI_573878953
GI_499049716
GI_548541918
GI_554876860
ENSTNIG00000018878
ENSTRUG00000010930
ENSDARG00000038524
ENSAMXG00000011959

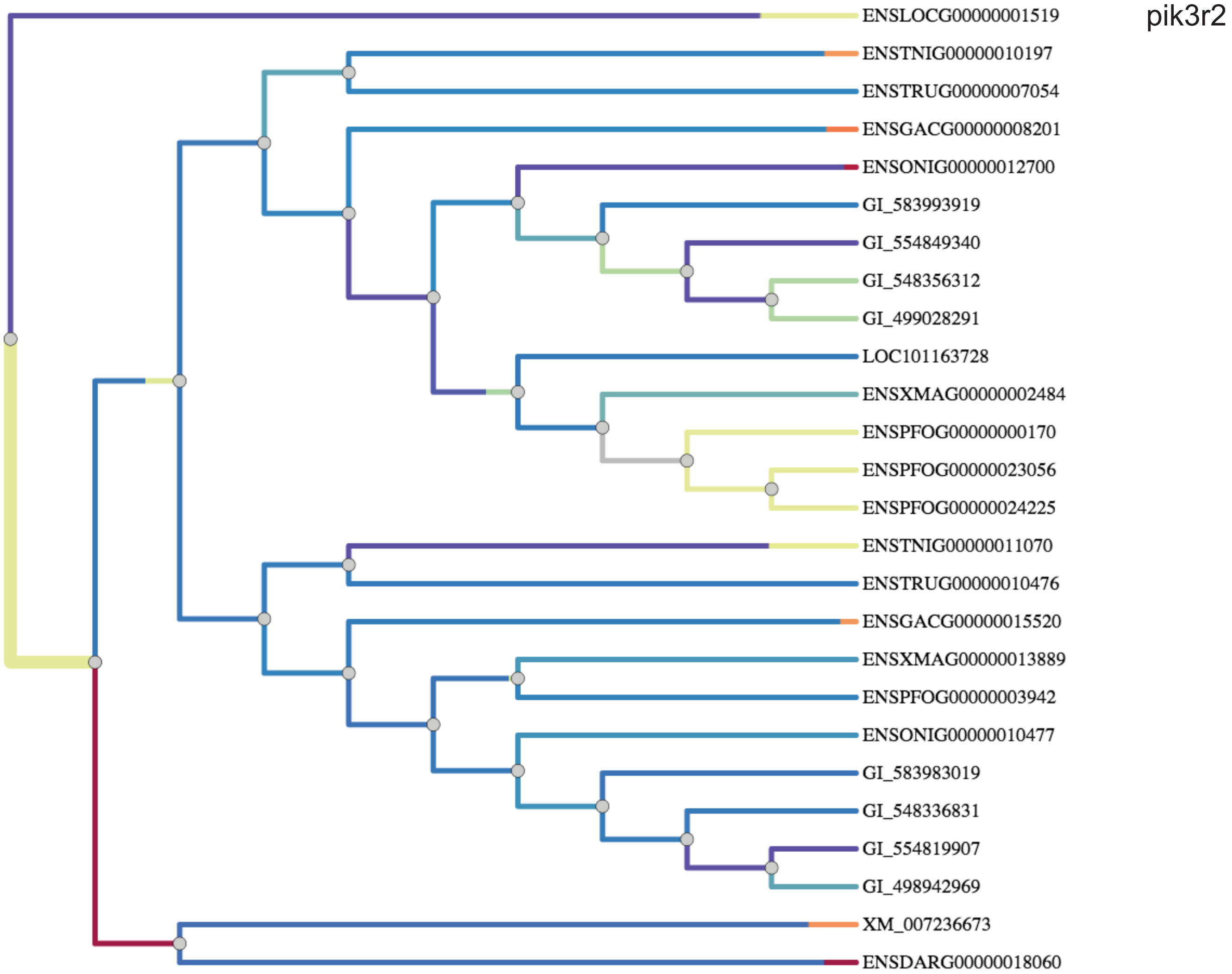
pik3r2
ENSLOCG00000001519
ENSTNIG00000010197
ENSTRUG00000007054
ENSGACG00000008201
ENSONIG00000012700
GI_583993919
GI_554849340
GI_548356312
GI_499028291
LOC101163728
ENSXMAG00000002484
ENSPFOG00000000170
ENSPFOG00000023056
ENSPFOG00000024225
ENSTNIG00000011070
ENSTRUG00000010476
ENSGACG00000015520
ENSXMAG00000013889
ENSPFOG00000003942
ENSONIG00000010477
GI_583983019
GI_548336831
GI_554819907
GI_498942969
XM_007236673
ENSDARG00000018060

Figure S3

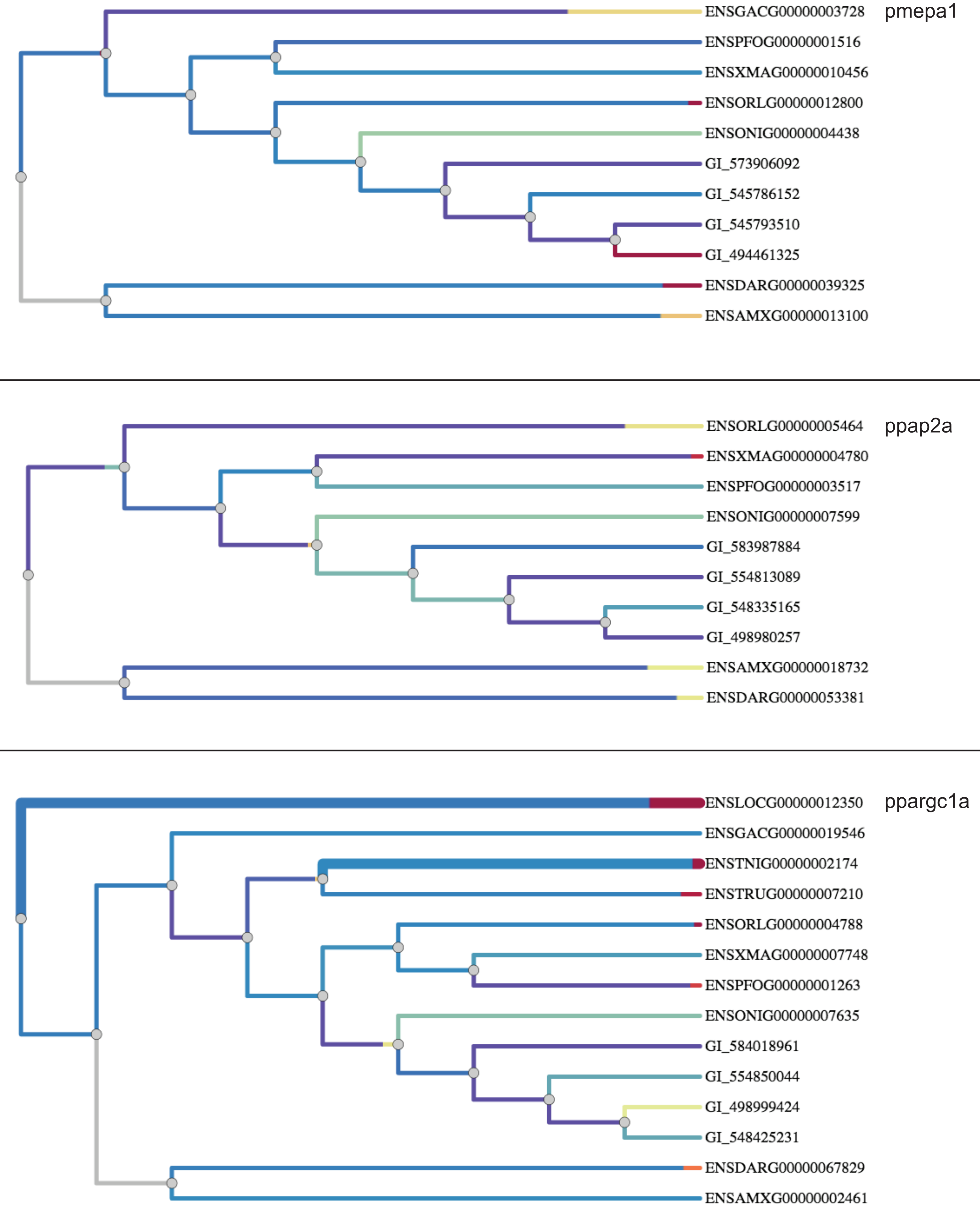

ENSGACG00000003728
pmepa1
ENSPFOG00000001516
ENSXMAG00000010456
ENSORLG00000012800
ENSONIG00000004438
GI_573906092
GI_545786152
GI_545793510
GI_494461325
ENSDARG00000039325
ENSAMXG00000013100
ENSORLG00000005464
ppap2a
ENSXMAG00000004780
ENSPFOG00000003517
ENSONIG00000007599
GI_583987884
GI_554813089
GI_548335165
GI_498980257
ENSAMXG00000018732
ENSDARG00000053381
ENSLOCG00000012350
ppargc1a
ENSGACG00000019546
ENSTNIG00000002174
ENSTRUG00000007210
ENSORLG00000004788
ENSXMAG00000007748
ENSPFOG00000001263
ENSONIG00000007635
GI_584018961
GI_554850044
GI_498999424
GI_548425231
ENSDARG00000067829
ENSAMXG00000002461

Figure S3

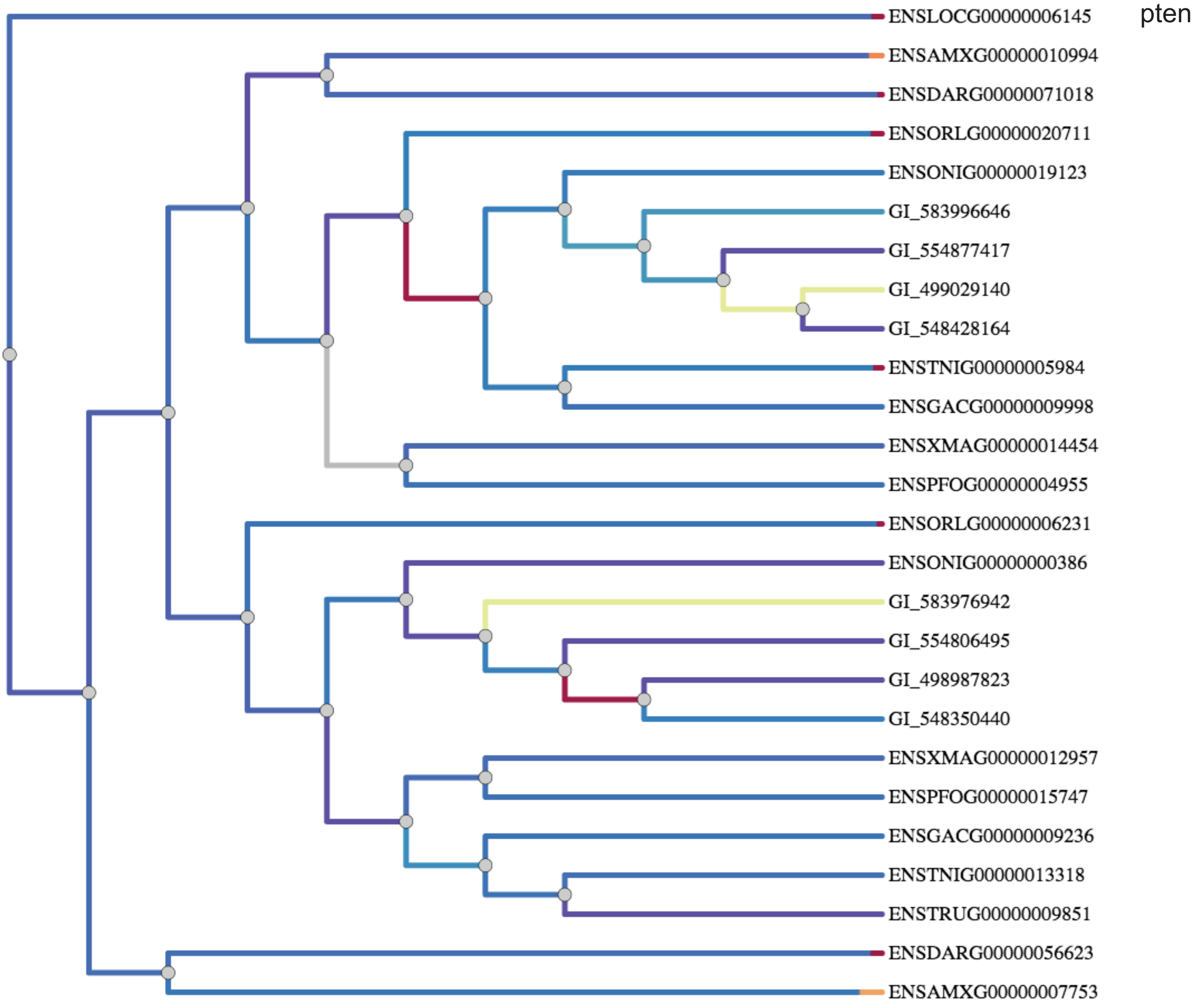
pten
ENSLOCG00000006145
ENSAMXG00000010994
ENSDARG00000071018
ENSORLG00000020711
ENSONIG00000019123
GI_583996646
GI_554877417
GI_499029140
GI_548428164
ENSTNIG00000005984
ENSGACG00000009998
ENSXMAG00000014454
ENSPFOG00000004955
ENSORLG00000006231
ENSONIG00000000386
GI_583976942
GI_554806495
GI_498987823
GI_548350440
ENSXMAG00000012957
ENSPFOG00000015747
ENSGACG00000009236
ENSTNIG00000013318
ENSTRUG00000009851
ENSDARG00000056623
ENSAMXG00000007753

Figure S3

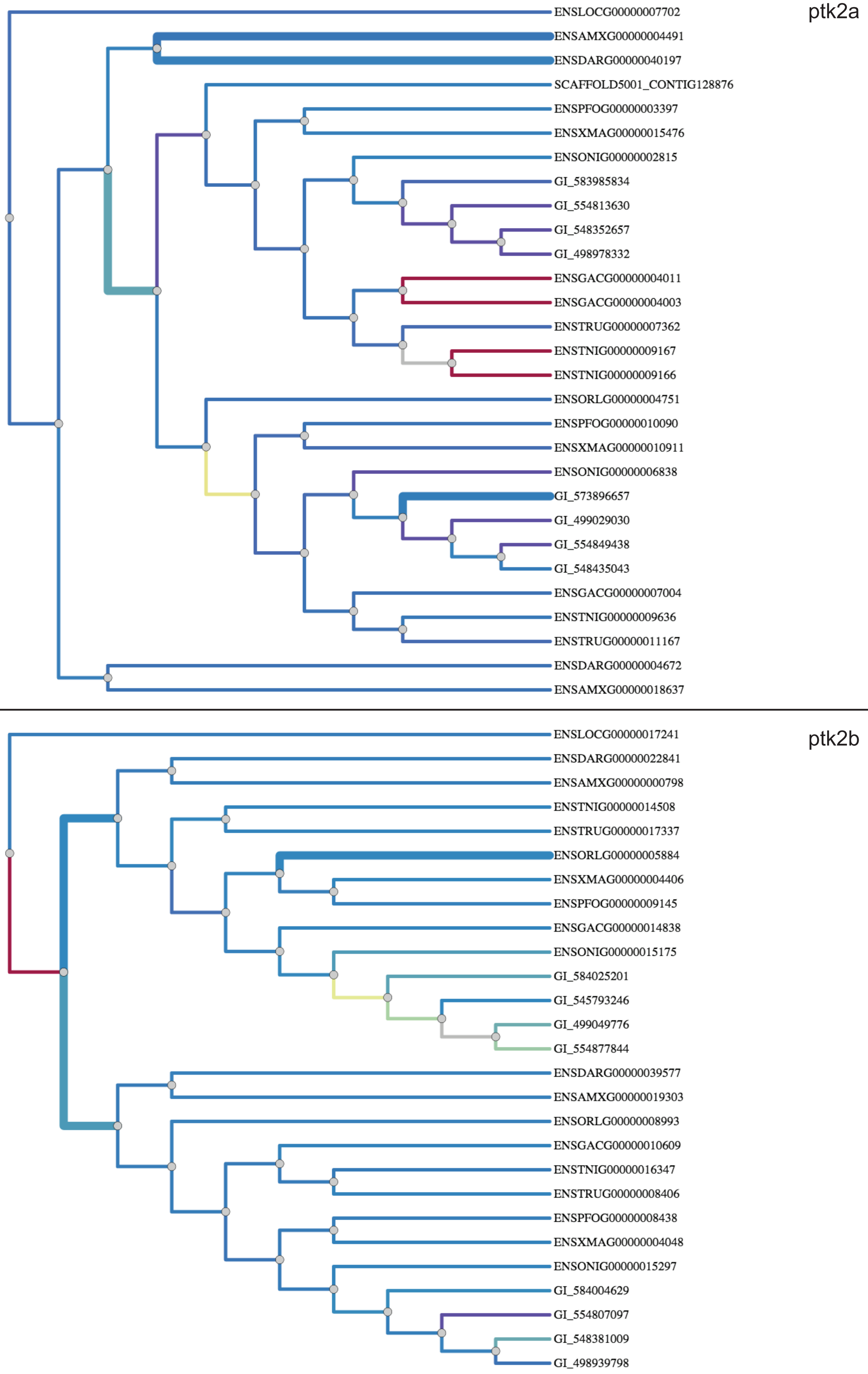

ptk2a
ENSLOCG00000007702
ENSAMXG00000004491
ENSDARG00000040197
SCAFFOLD5001_CONTIG128876
ENSPFOG00000003397
ENSXMAG00000015476
ENSONIG00000002815
GI_583985834
GI_554813630
GI_548352657
GI_498978332
ENSGACG00000004011
ENSGACG00000004003
ENSTRUG00000007362
ENSTNIG00000009167
ENSTNIG00000009166
ENSORLG00000004751
ENSPFOG00000010090
ENSXMAG00000010911
ENSONIG00000006838
GI_573896657
GI_499029030
GI_554849438
GI_548435043
ENSGACG00000007004
ENSTNIG00000009636
ENSTRUG00000011167
ENSDARG00000004672
ENSAMXG00000018637
ptk2b
ENSLOCG00000017241
ENSDARG00000022841
ENSAMXG00000000798
ENSTNIG00000014508
ENSTRUG00000017337
ENSORLG00000005884
ENSXMAG00000004406
ENSPFOG00000009145
ENSGACG00000014838
ENSONIG00000015175
GI_584025201
GI_545793246
GI_499049776
GI_554877844
ENSDARG00000039577
ENSAMXG00000019303
ENSORLG00000008993
ENSGACG00000010609
ENSTNIG00000016347
ENSTRUG00000008406
ENSPFOG00000008438
ENSXMAG00000004048
ENSONIG00000015297
GI_584004629
GI_554807097
GI_548381009
GI_498939798

Figure S3

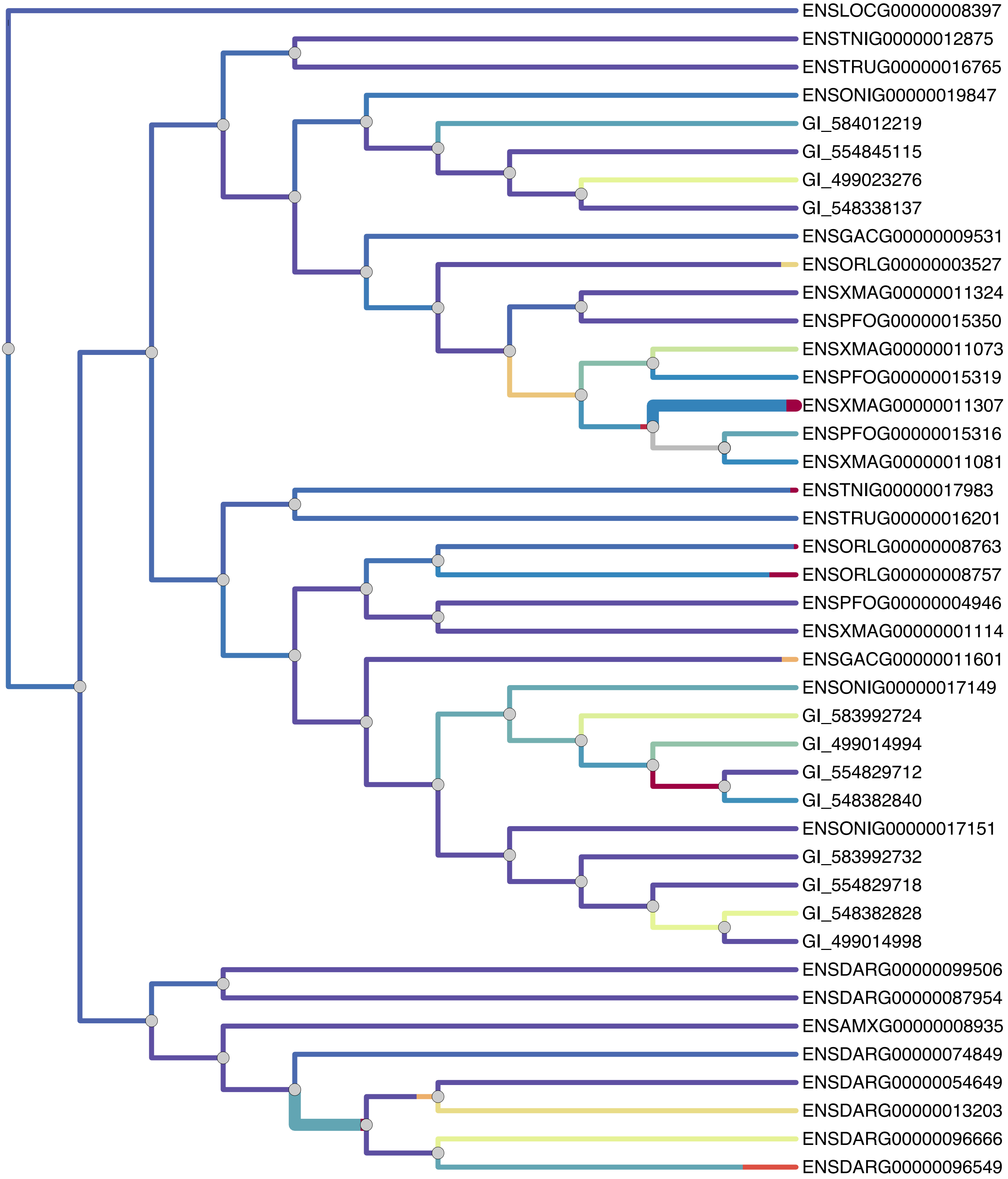

rac1

Figure S3

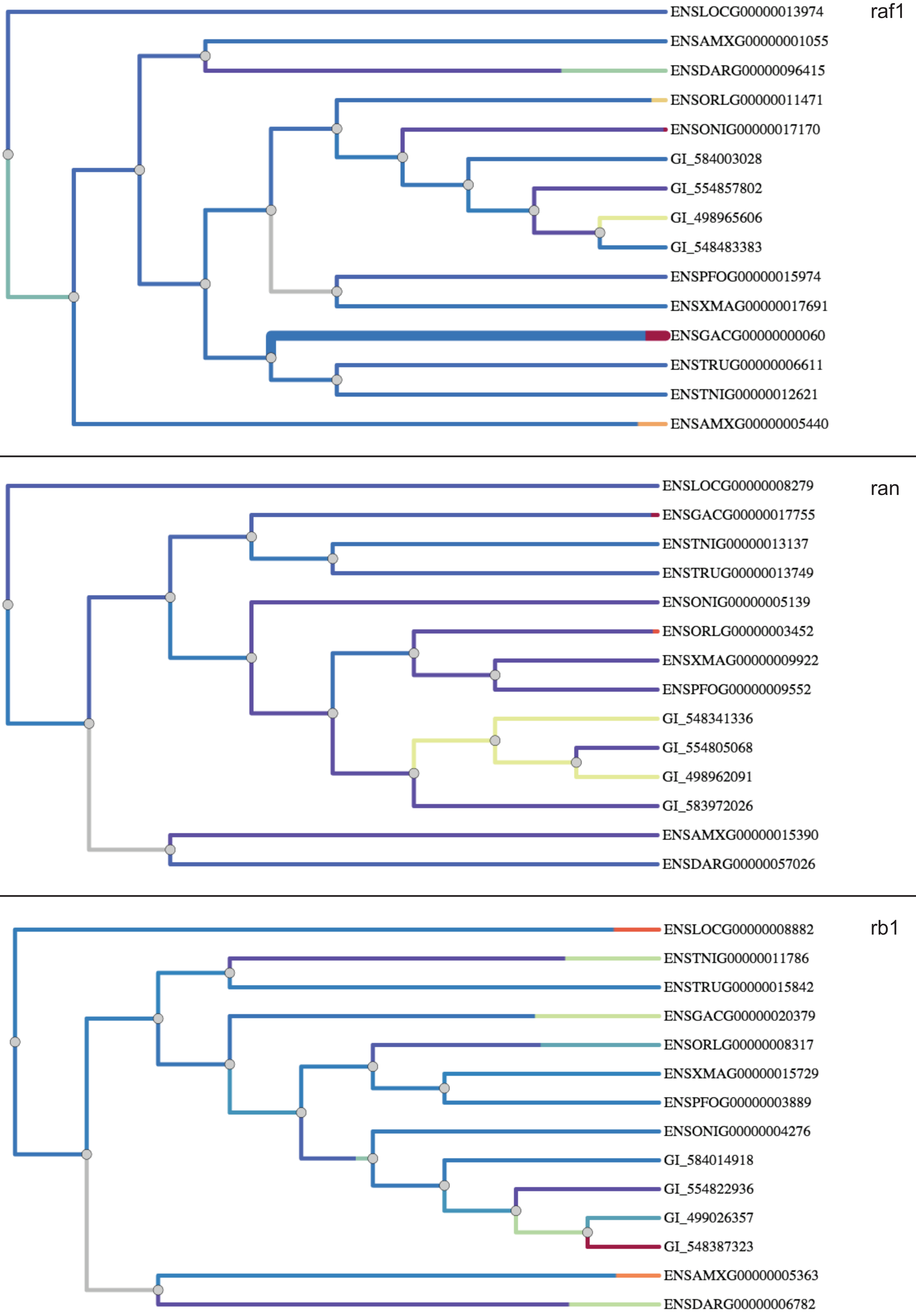
raf1
ENSLOCG00000013974
ENSAMXG00000001055
ENSDARG00000096415
ENSORLG00000011471
ENSONIG00000017170
GI_584003028
GI_554857802
GI_498965606
GI_548483383
ENSPFOG00000015974
ENSXMAG00000017691
ENSGACG00000000060
ENSTRUG00000006611
ENSTNIG00000012621
ENSAMXG00000005440
ran
ENSLOCG00000008279
ENSGACG00000017755
ENSTNIG00000013137
ENSTRUG00000013749
ENSONIG00000005139
ENSORLG00000003452
ENSXMAG00000009922
ENSPFOG00000009552
GI_548341336
GI_554805068
GI_498962091
GI_583972026
ENSAMXG00000015390
ENSDARG00000057026
rb1
ENSLOCG00000008882
ENSTNIG00000011786
ENSTRUG00000015842
ENSGACG00000020379
ENSORLG00000008317
ENSXMAG00000015729
ENSPFOG00000003889
ENSONIG00000004276
GI_584014918
GI_554822936
GI_499026357
GI_548387323
ENSAMXG00000005363
ENSDARG00000006782

Figure S3

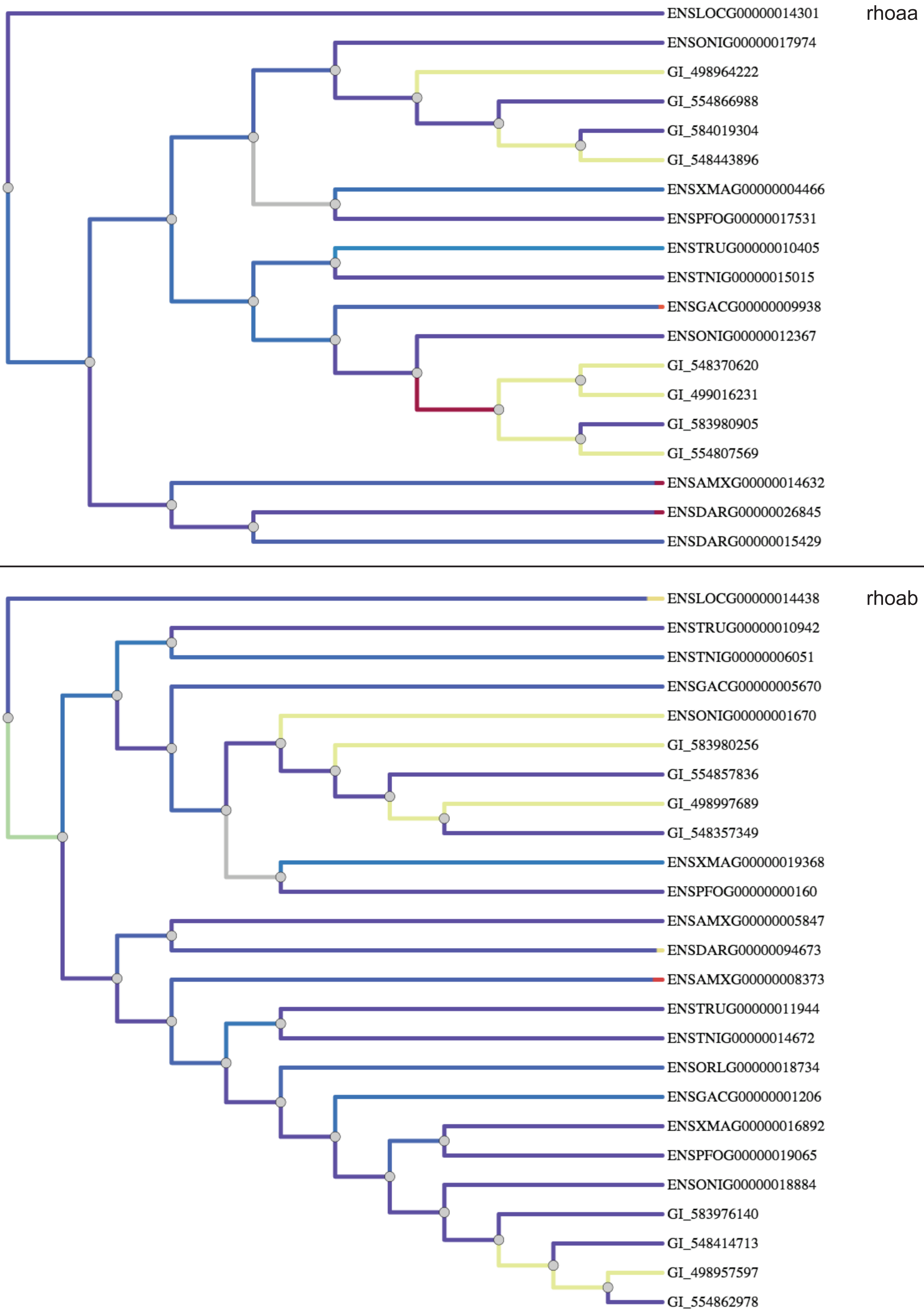
ENSLOCG00000014301
rhoaa
ENSONIG00000017974
GI_498964222
GI_554866988
GI_584019304
GI_548443896
ENSXMAG00000004466
ENSPFOG00000017531
ENSTRUG00000010405
ENSTNIG00000015015
ENSGACG00000009938
ENSONIG00000012367
GI_548370620
GI_499016231
GI_583980905
GI_554807569
ENSAMXG00000014632
ENSDARG00000026845
ENSDARG00000015429
ENSLOCG00000014438
rhoab
ENSTRUG00000010942
ENSTNIG00000006051
ENSGACG00000005670
ENSONIG00000001670
GI_583980256
GI_554857836
GI_498997689
GI_548357349
ENSXMAG00000019368
ENSPFOG00000000160
ENSAMXG00000005847
ENSDARG00000094673
ENSAMXG00000008373
ENSTRUG00000011944
ENSTNIG00000014672
ENSORLG00000018734
ENSGACG00000001206
ENSXMAG00000016892
ENSPFOG00000019065
ENSONIG00000018884
GI_583976140
GI_548414713
GI_498957597
GI_554862978

Figure S3

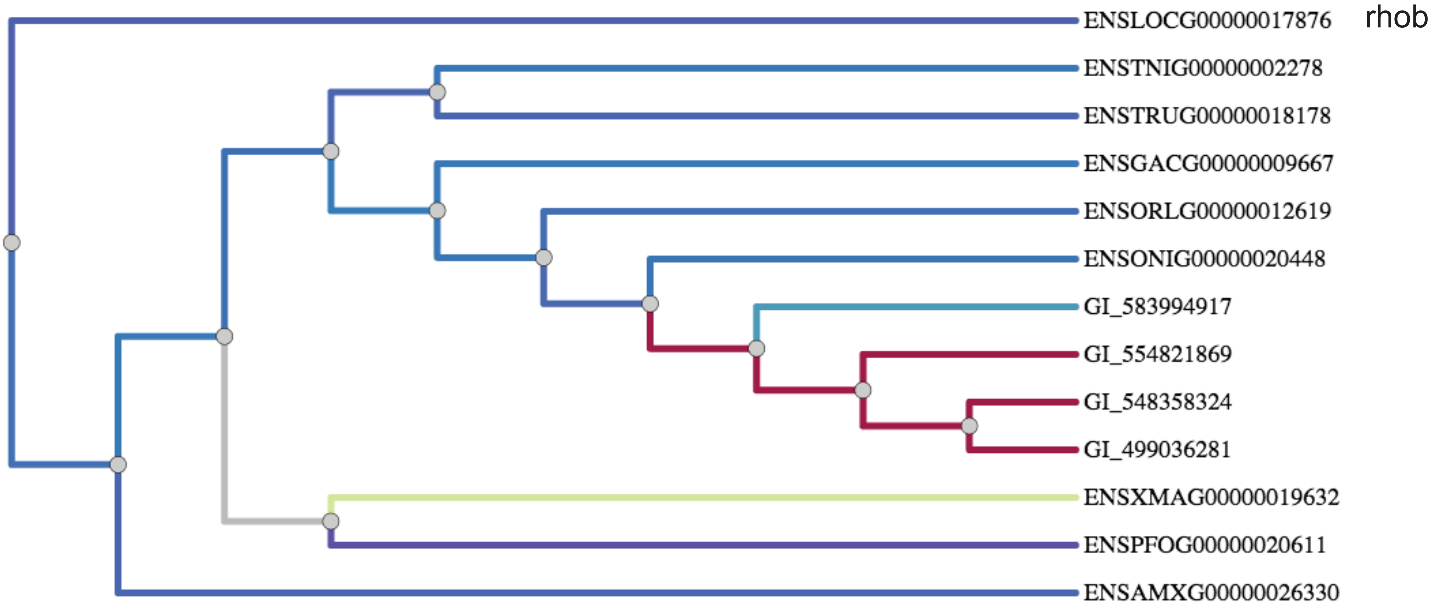
ENSLOCG00000017876
rhob
ENSTNIG00000002278
ENSTRUG00000018178
ENSGACG00000009667
ENSORLG00000012619
ENSONIG00000020448
GI_583994917
GI_554821869
GI_548358324
GI_499036281
ENSXMAG00000019632
ENSPFOG00000020611
ENSAMXG00000026330

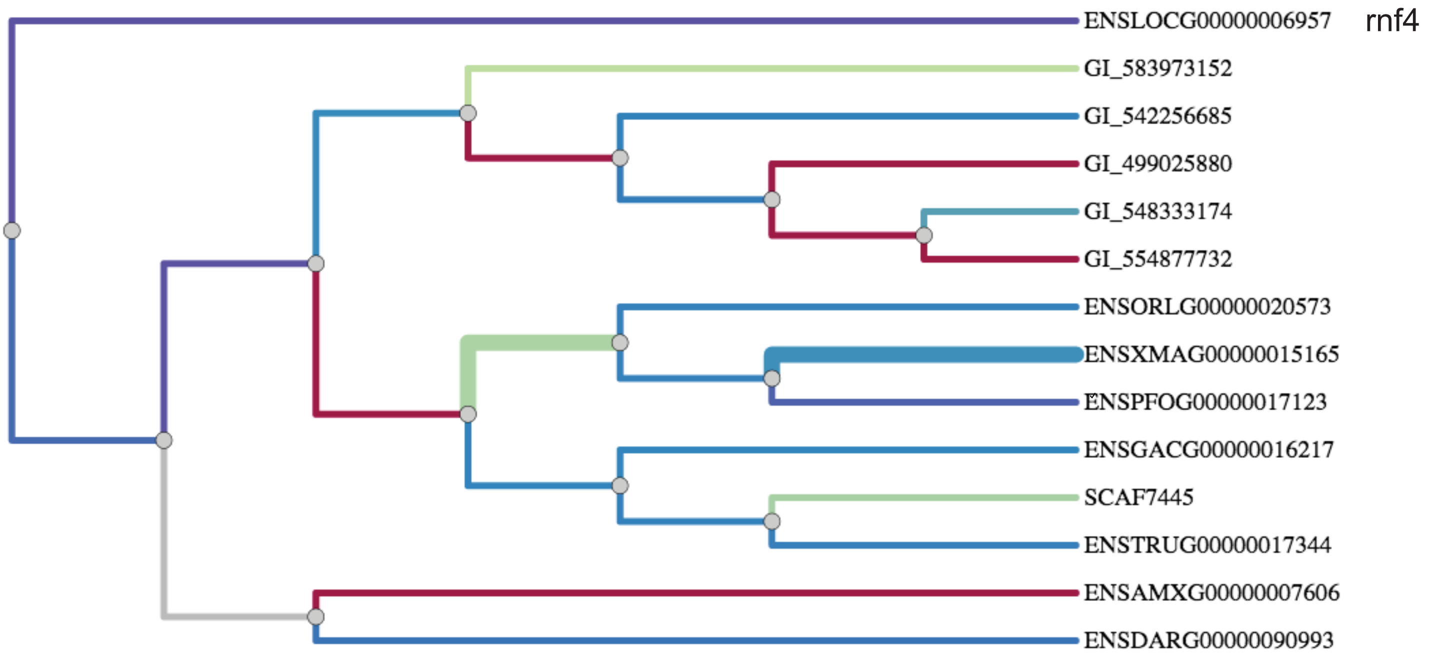
ENSLOCG00000006957
rnf4
GI_583973152
GI_542256685
GI_499025880
GI_548333174
GI_554877732
ENSORLG00000020573
ENSXMAG00000015165
ENSPFOG00000017123
ENSGACG00000016217
SCAF7445
ENSTRUG00000017344
ENSAMXG00000007606
ENSDARG00000090993

Figure S3

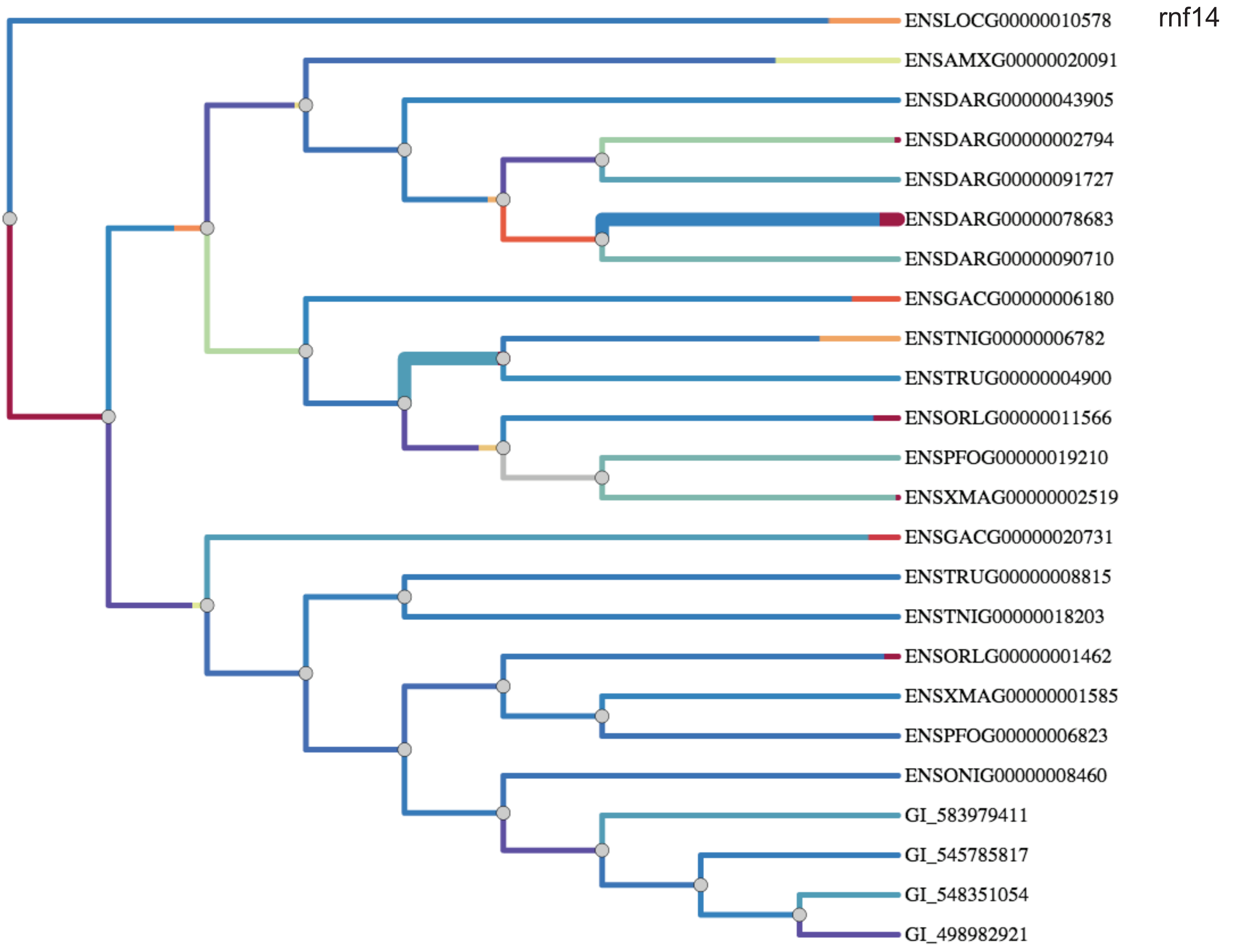
rnf14
ENSLOCG00000010578
ENSAMXG00000020091
ENSDARG00000043905
ENSDARG00000002794
ENSDARG00000091727
ENSDARG00000078683
ENSDARG00000090710
ENSGACG00000006180
ENSTNIG00000006782
ENSTRUG00000004900
ENSORLG00000011566
ENSPFOG00000019210
ENSXMAG00000002519
ENSGACG00000020731
ENSTRUG00000008815
ENSTNIG00000018203
ENSORLG00000001462
ENSXMAG00000001585
ENSPFOG00000006823
ENSONIG00000008460
GI_583979411
GI_545785817
GI_548351054
GI_498982921

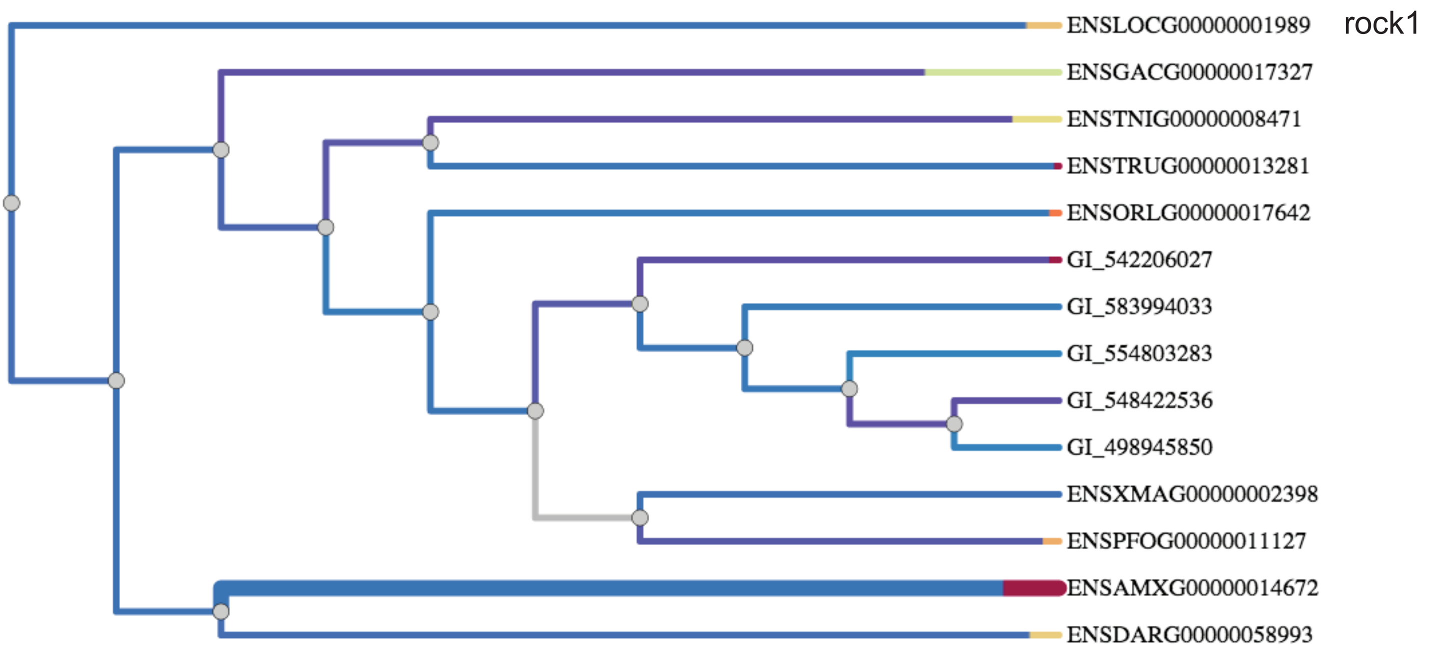
rock1
ENSLOCG00000001989
ENSGACG00000017327
ENSTNIG00000008471
ENSTRUG00000013281
ENSORLG00000017642
GI_542206027
GI_583994033
GI_554803283
GI_548422536
GI_498945850
ENSXMAG00000002398
ENSPFOG00000011127
ENSAMXG00000014672
ENSDARG00000058993

Figure S3

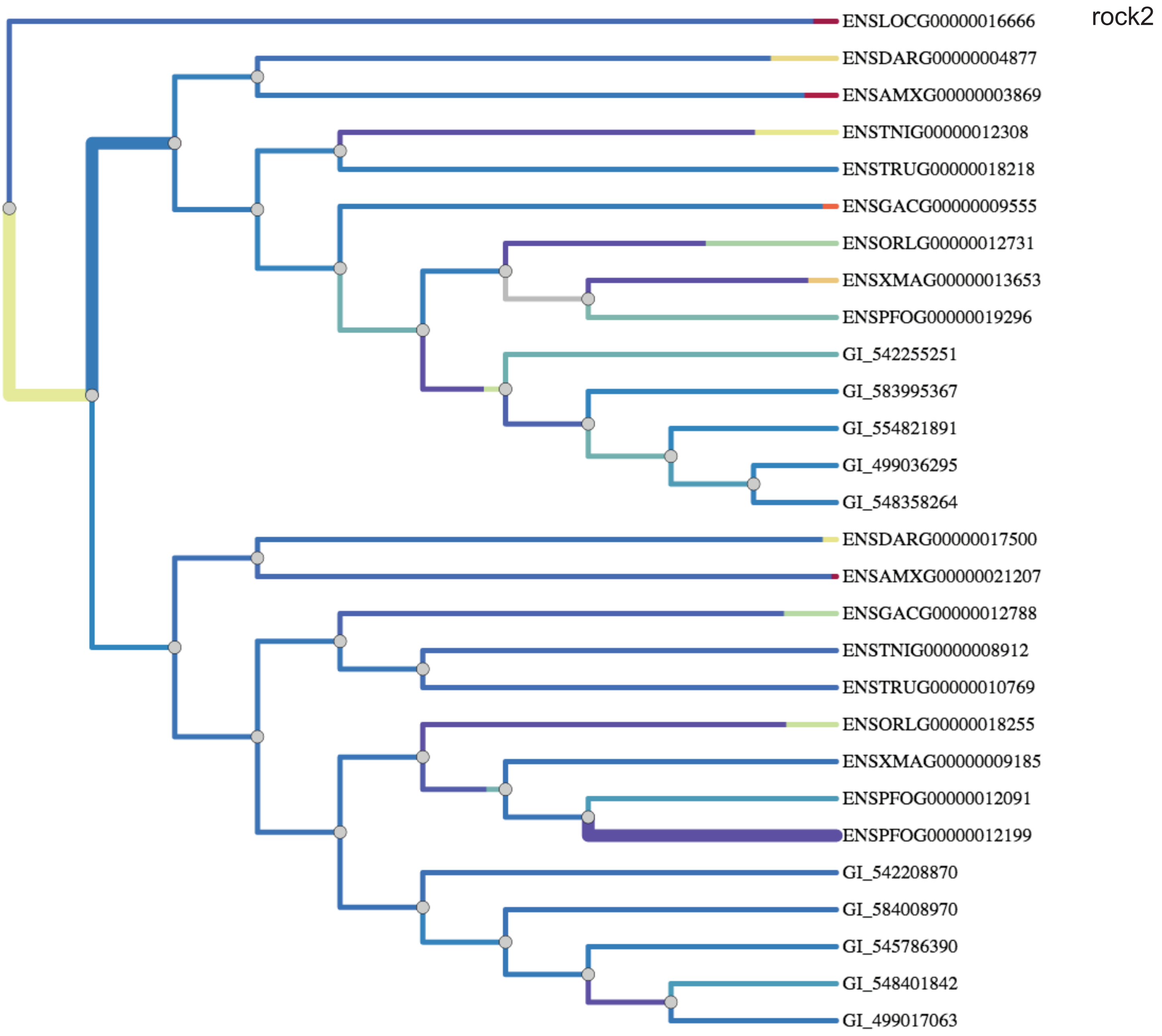
rock2
ENSLOCG00000016666
ENSDARG00000004877
ENSAMXG00000003869
ENSTNIG00000012308
ENSTRUG00000018218
ENSGACG00000009555
ENSORLG00000012731
ENSXMAG00000013653
ENSPFOG00000019296
GI_542255251
GI_583995367
GI_554821891
GI_499036295
GI_548358264
ENSDARG00000017500
ENSAMXG00000021207
ENSGACG00000012788
ENSTNIG00000008912
ENSTRUG00000010769
ENSORLG00000018255
ENSXMAG00000009185
ENSPFOG00000012091
ENSPFOG00000012199
GI_542208870
GI_584008970
GI_545786390
GI_548401842
GI_499017063

Figure S3

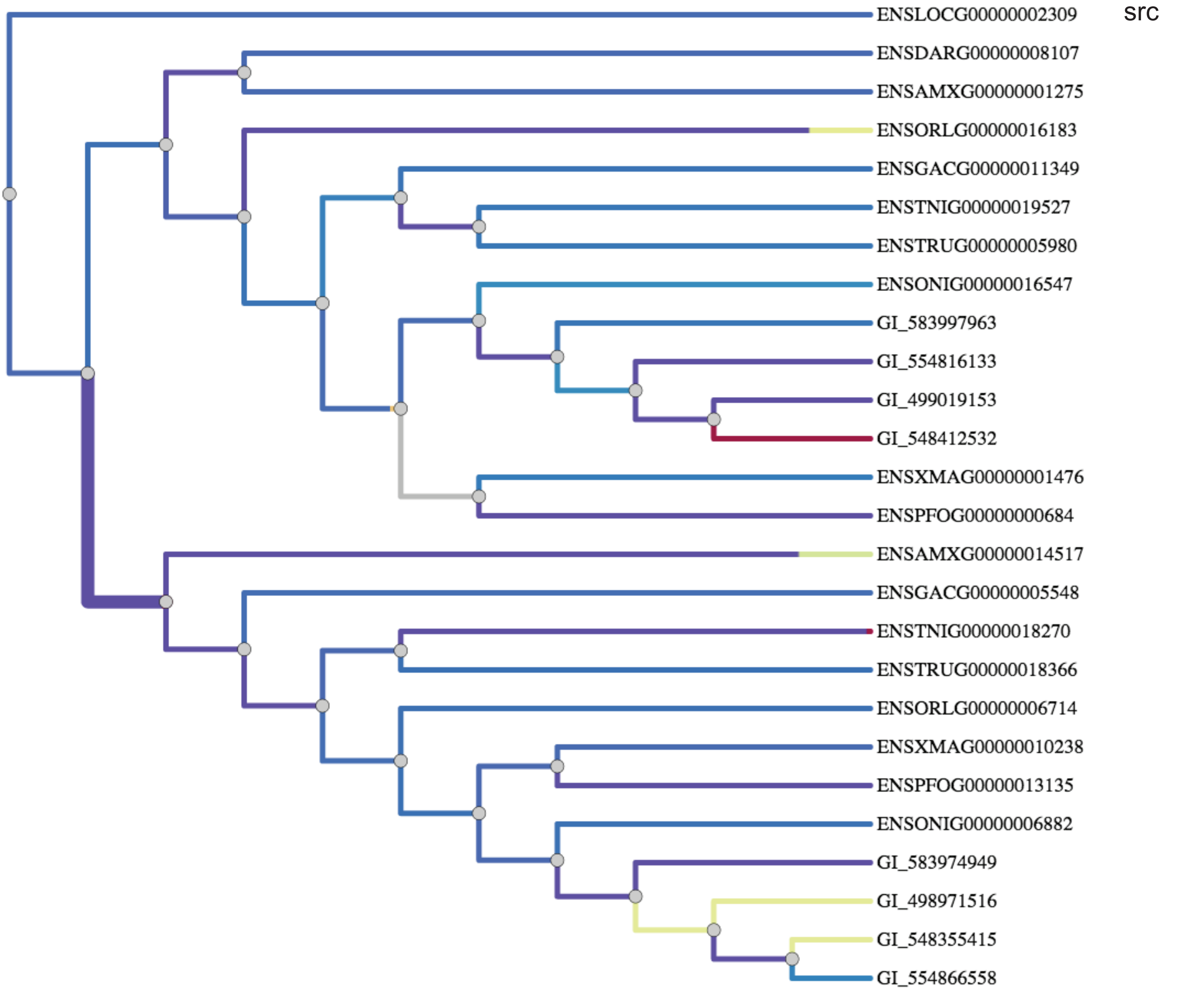

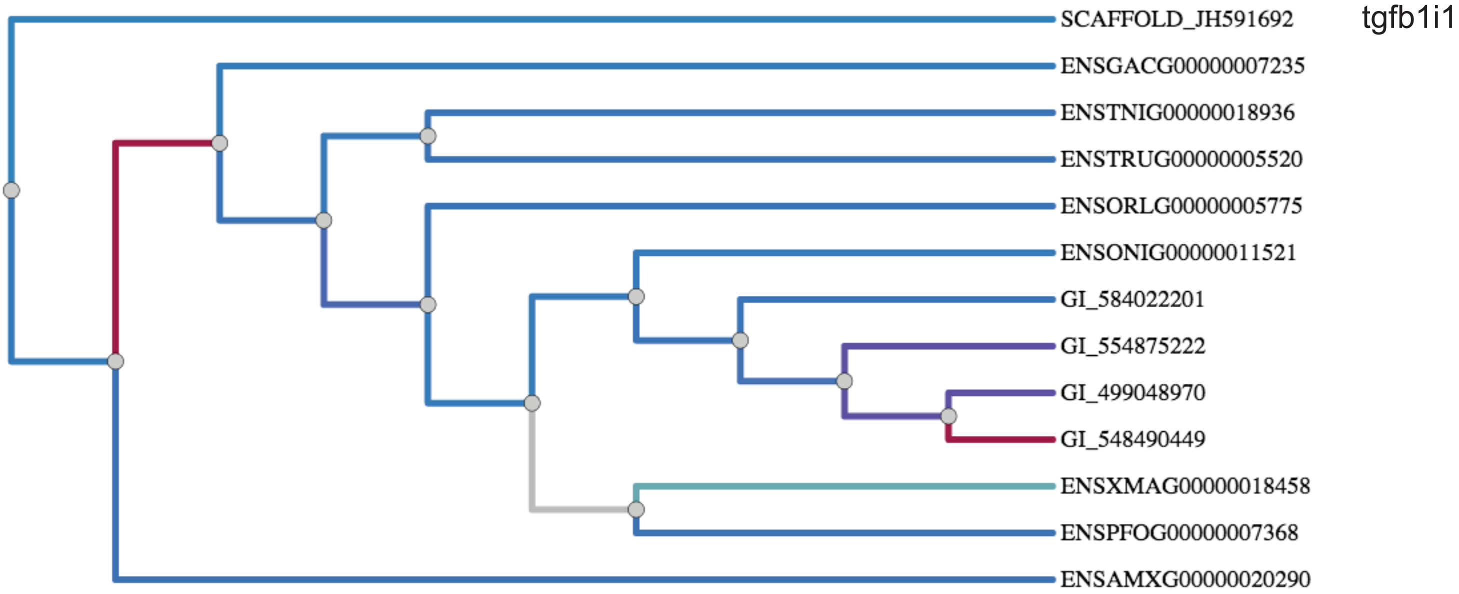

 T. Lorin, W. Salzburger & A. Böhne

Figure S3

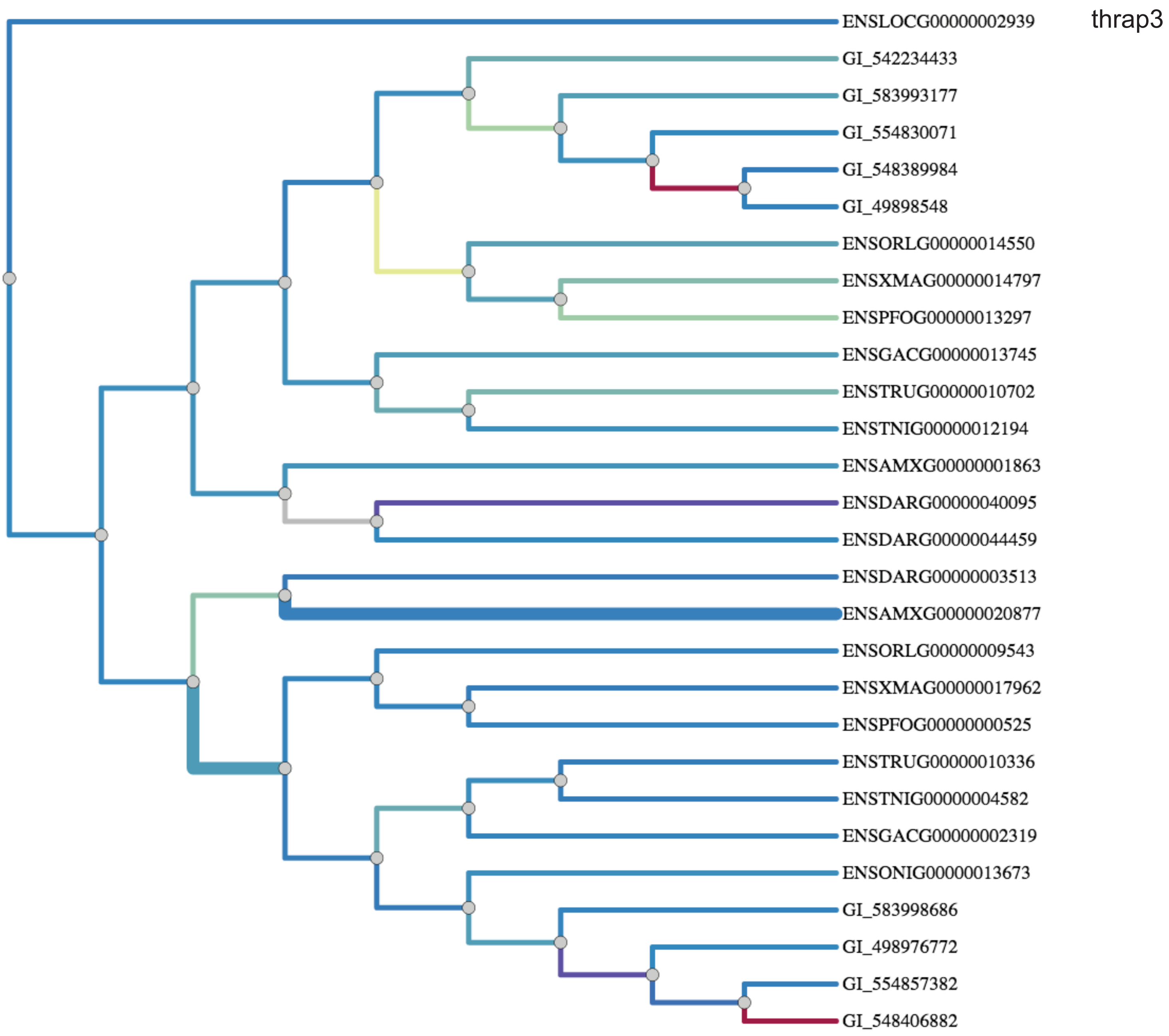
thrap3
ENSLOCG00000002939
GI_542234433
GI_583993177
GI_554830071
GI_548389984
GI_49898548
ENSORLG00000014550
ENSXMAG00000014797
ENSPFOG00000013297
ENSGACG00000013745
ENSTRUG00000010702
ENSTNIG00000012194
ENSAMXG00000001863
ENSDARG00000040095
ENSDARG00000044459
ENSDARG00000003513
ENSAMXG00000020877
ENSORLG00000009543
ENSXMAG00000017962
ENSPFOG00000000525
ENSTRUG00000010336
ENSTNIG00000004582
ENSGACG00000002319
ENSONIG00000013673
GI_583998686
GI_498976772
GI_554857382
GI_548406882

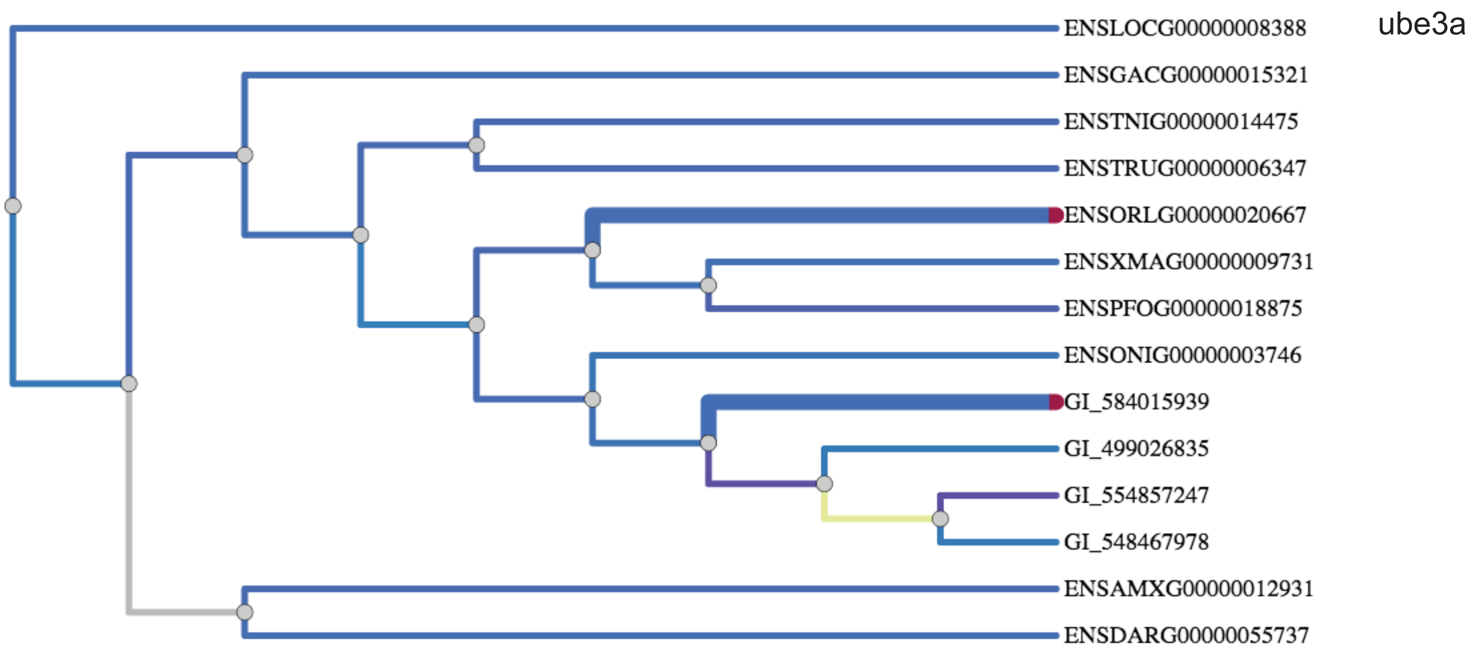
ube3a
ENSLOCG00000008388
ENSGACG00000015321
ENSTNIG00000014475
ENSTRUG00000006347
ENSORLG00000020667
ENSXMAG00000009731
ENSPFOG00000018875
ENSONIG00000003746
GI_584015939
GI_499026835
GI_554857247
GI_548467978
ENSAMXG00000012931
ENSDARG00000055737

Table S1

Ray-finned fish genes of the non-genomic and genomic Androgen Receptor signaling pathway according to NetPath, GO0030521, Bennett *et al.* 2010, Foradori *et al.* 2008

Gene	Species *L.oculatus*	Gene	Species *D.rerio*	*A.mexicanus*	*G.aculeatus*	*T.nigroviridis*	*T.rubripes*	*P.formosa*	*X.maculatus*	*O.latipes*	*O.niloticus*	*N.brichardi*	*M.zebra*	*P.nyererei*	*A.burtoni*	Source of gene	Pathway association
AKT1	ENSLOCG00000012762		NM_001281801.1	ENSAMXG00000009230<br>ENSAMXG00000012355	ENSGACG00000006298	ENSTNIG00000019495	ENSTRUG00000013476	ENSPFOG00000010125	ENSXMAG00000006673	ENSORLG00000017024	gi_542229192	gi_583981523	gi_498935125	gi_548423349	gi_554812288	NetPath, Bennett et al 2010, Foradori et al 2008	non-genomic, genomic
AR	ENSLOCG00000014680	ARA		ENSAMXG00000013256	ENSGACG00000018525	ENSTNIG00000015783	ENSTRUG00000005373	ENSPFOG00000006490	ENSXMAG00000002896	ENSORLG00000008220	ENSONIG00000017538	gi_583972812	gi_499025148	gi_545793521	gi_555943746	NetPath	receptor, genomic, non-genomic
		ARB	ENSDARG00000067976	ENSAMXG00000011395	ENSGACG00000020332	ENSTNIG00000011826	ENSTRUG00000012421	ENSPFOG00000019378	ENSXMAG00000012307	ENSORLG00000009520	ENSONIG00000012854	gi_584009291	gi_499011353	gi_548397957	gi_555943801	NetPath	receptor, genomic, non-genomic
ARID1	ENSLOCG00000003798	ARID1AA	ENSDARG00000101710	ENSAMXG00000020237	ENSGACG00000007244	ENSTNIG00000009659	ENSTRUG00000013351	ENSPFOG00000009717	ENSXMAG00000010716	ENSORLG00000004410	ENSONIG00000006764	gi_584001874	gi_499018427	gi_548435329	gi_554870576	GO:0030521	genomic
		ARID1AB	ENSDARG00000101891	ENSAMXG00000013606	ENSGACG00000007627	ENSTNIG00000004670	ENSTRUG00000012325	ENSPFOG00000002208	ENSXMAG00000012041		ENSONIG00000007618	gi_584000591	gi_499027400	gi_548538463	gi_554835101	GO:0030521	genomic
BRCA1	ENSLOCG00000011391	BRCA1		ENSAMXG00000015282	ENSGACG00000006381	ENSTNIG00000010246	ENSTRUG00000009091	ENSPFOG00000004731	ENSXMAG00000009949	ENSORLG00000004585	ENSONIG00000016326	gi_583992196	gi_499033693	gi_548541303	gi_554835497	NetPath, GO:0030521	genomic, positive regulator
CAV1	ENSLOCG00000015597	CAV1	ENSDARG00000052004	ENSAMXG00000011618	ENSGACG00000009201	ENSTNIG00000016057	ENSTRUG00000017112	ENSPFOG00000014440	ENSXMAG00000014453	ENSORLG00000019746	ENSONIG00000003896	gi_583973968	gi_499007024	gi_548521455	gi_554856484	NetPath	non-genomic
CCNE1	ENSLOCG00000005671	CCNE1	ENSDARG00000098622	ENSAMXG00000002929	ENSGACG00000012549	ENSTNIG00000006196	ENSTRUG00000000222<br>ENSTRUG00000000353<br>ENSTRUG00000001296<br>ENSTRUG00000001834<br>ENSTRUG00000001857<br>ENSTRUG00000017625<br>ENSTRUG00000000484	ENSPFOG00000011801	ENSXMAG00000001005	ENSORLG00000015511	ENSONIG00000003504	gi_584015275	gi_499030053	gi_548348207	gi_554826914	NetPath, GO:0030521	genomic, positive regulator
CDC4	ENSLOCG00000001073	CDC42A		ENSAMXG00000019820	ENSGACG00000012448	ENSTNIG00000012549	ENSTRUG00000013411	ENSPFOG00000005335	ENSXMAG00000016228	ENSORLG00000013756	ENSONIG00000016914	gi_573898313	gi_494461779	gi_545793478	gi_545786543	NetPath	non-genomic
		CDC42B	ENSDARG00000044573	ENSAMXG00000003583	ENSGACG00000010913	ENSTNIG00000007372	ENSTRUG00000000167	ENSPFOG00000004608	ENSXMAG00000008839	ENSORLG00000012048	ENSONIG00000020235	gi_584010624	gi_499018345	gi_548348712	gi_554846439	NetPath	non-genomic
CDK7	ENSLOCG00000006117	CDK7	ENSDARG00000051916	ENSAMXG00000008454	ENSGACG00000009848	ENSTNIG00000015407	ENSTRUG00000010233	ENSPFOG00000017422	ENSXMAG00000007316	ENSORLG00000008836	ENSONIG00000013709	gi_583977761	gi_498955997	gi_548339440	gi_554874678	GO:0030521	genomic
CFL1L	ENSLOCG00000015680	CFL1L	ENSDARG00000012972	ENSAMXG00000007837	ENSGACG00000004750			ENSPFOG00000017145	ENSXMAG00000006139	ENSORLG00000011865	ENSONIG00000010009	gi_584024814	no blast hit on n	gi_548490653	gi_554852342	NetPath	non-genomic
CTNNB1	ENSLOCG00000001383	CTNNB1	ENSDARG00000014571	ENSAMXG00000000198	ENSGACG00000006037	ENSTNIG00000019094	ENSTRUG00000003520	ENSPFOG00000018568	ENSXMAG00000008710	ENSORLG00000005845	ENSONIG00000007226	gi_583999804	gi_499034959	gi_548426098	gi_554880769	NetPath, GO:0030521	genomic, positive regulator
DAXX	ENSLOCG00000000498	DAXX	ENSDARG00000000729	ENSAMXG00000014180	ENSGACG00000001844	ENSTNIG00000005315<br>ENSTNIG00000018185	ENSTRUG00000003811	ENSPFOG00000001776	ENSXMAG00000007090	ENSORLG00000006837	ENSONIG00000008401	gi_573898112	gi_499044186	gi_548490874	gi_554886315	NetPath, GO:0030521	genomic, negative regulator
DNAJA1	ENSLOCG00000011564	DNAJA1	ENSDARG00000030972	ENSAMXG00000010757	ENSGACG00000017879			ENSPFOG00000004920<br>ENSPFOG00000005099	ENSXMAG00000005855	ENSORLG00000006260	ENSONIG00000014539	gi_583983886	gi_498979425	gi_548348922	gi_554873916	GO:0030521	genomic
EGFR	ENSLOCG00000011537	EGFRA	gi_35903182	ENSAMXG00000012474	ENSGACG00000017289	ENSTNIG00000014840	ENSTRUG00000011834	ENSPFOG00000008848	ENSXMAG00000016482	ENSORLG00000017692	ENSONIG00000009358	gi_583994068	gi_498945991	gi_548422483	gi_554803235	NetPath	non-genomic
		EGFRB		ENSAMXG00000015959	ENSGACG00000018079	ENSTNIG00000000023<br>ENSTNIG00000013552<br>ENSTNIG00000013553	ENSTRUG00000017446	ENSPFOG00000005185	ENSXMAG00000008804	ENSORLG00000003577	ENSONIG00000017327	gi_584029670	gi_499050178	gi_548511931	gi_545785452	NetPath	non-genomic
FHL2	ENSLOCG00000008763	FHL2A	ENSDARG00000003991	ENSAMXG00000005118	ENSGACG00000015048	ENSTNIG00000017175	ENSTRUG00000008468	ENSPFOG00000003291<br>ENSPFOG00000022124	ENSXMAG00000002166	ENSORLG00000001848	ENSONIG00000012307	gi_583995557	gi_499025292	gi_548401457	gi_554818515	NetPath, GO:0030521	genomic, positive regulator
		FHL2B	ENSDARG00000042018	ENSAMXG00000009663	ENSGACG00000003005	ENSTNIG00000000462	ENSTRUG00000013559	ENSPFOG00000015302	ENSXMAG00000011596	ENSORLG00000012482	ENSONIG00000014220	gi_583986192	gi_498986713	gi_548379261	gi_554870366	NetPath, GO:0030521	genomic, positive regulator
FKBP4	ENSLOCG00000016880	FKBP4	ENSDARG00000008447	ENSAMXG00000003846	ENSGACG00000012979	NA	ENSTRUG00000015044	ENSPFOG00000018881	ENSXMAG00000003963	ENSORLG00000008977	ENSONIG00000015051	gi_583988484	gi_498994696	gi_548356125	gi_545786431	GO:0030521	genomic, negative regulator
FLNA	ENSLOCG00000015505	FLNA	ENSDARG00000074201	ENSAMXG00000013411	ENSGACG00000013098	ENSTNIG00000004951	ENSTRUG00000003170	ENSPFOG00000001616	ENSXMAG00000004665	ENSORLG00000001982	ENSONIG00000017914	gi_584019220	gi_498964459	gi_548536083	gi_554815264	NetPath	non-genomic
GNB2I1	ENSLOCG00000009758	GNB2I1	ENSDARG00000041619	ENSAMXG00000007457<br>ENSAMXG00000002584	ENSGACG00000018103	ENSTNIG00000010510	ENSTRUG00000000550	ENSPFOG00000008165	ENSXMAG00000008940	ENSORLG00000005276	ENSONIG00000012984	gi_583972285	gi_498935526	gi_548334125	gi_554821010	NetPath	genomic
GRIP1	ENSLOCG00000016167	GRIP1	ENSDARG00000015053	ENSAMXG00000011215	ENSGACG00000000177	ENSTNIG00000002863	ENSTRUG00000005225	ENSPFOG00000012379	ENSXMAG00000006468	ENSORLG00000017453	gi_542204218	gi_583971256	gi_499031622	gi_548419148	gi_554820840	GO:0030521	genomic
KAT5	NA	KAT5	ENSDARG00000004587<br>ENSDARG00000045951	ENSAMXG00000009091	ENSGACG00000020408	ENSTNIG00000011743	ENSTRUG00000016643	ENSPFOG00000010293	ENSXMAG00000005173	ENSORLG00000007458	ENSONIG00000005359	gi_584015024	gi_499026510	gi_548385825	gi_554823092	NetPath, GO:0030521	genomic, positive regulator
KDM3A	ENSLOCG00000010817	KDM3A	NA	NA	NA	NA	NA	NA	NA	NA	NA	NA	NA	NA	NA	GO:0030521	genomic
LIMK2	ENSLOCG00000004821	LIMK2	ENSDARG00000005104	gi_597735797	ENSGACG00000008716	ENSTNIG00000015862	ENSTRUG00000013732	ENSPFOG00000009526	ENSXMAG00000018076	ENSORLG00000006835	ENSONIG00000013399	gi_573905058	gi_498956642	gi_548339212	gi_554811068	NetPath	non-genomic
MAPK1	ENSLOCG00000002024	MAPK1	ENSDARG00000027552	ENSAMXG00000001222	ENSGACG00000014421	ENSTNIG00000015351	ENSTRUG00000015300	ENSPFOG00000006212	ENSXMAG00000006444	scaffold5487_contig1298	ENSONIG00000014115	gi_584005676	gi_499004332	gi_548356923	gi_554822617	Bennett et al 2010	non-genomic, genomic
MAPK3	NA	MAPK3	ENSDARG00000070573	ENSAMXG00000017822	ENSGACG00000013102	ENSTNIG00000011583	ENSTRUG00000005697	ENSPFOG00000008154	ENSXMAG00000011987	ENSORLG00000011993	ENSONIG00000019408	gi_584026649	gi_499019863	gi_548501870	gi_554861523	Bennett et al 2010	non-genomic, genomic
MED1	ENSLOCG00000012767	MED1	ENSDARG00000075340	ENSAMXG00000015432	ENSGACG00000006033	ENSTNIG00000004334	ENSTRUG00000012065	ENSPFOG00000001519	ENSXMAG00000002969	ENSORLG00000008300	gi_542192331	gi_583992145	gi_499021221	gi_548391342	gi_554817925	GO:0030521	genomic
MED4	ENSLOCG00000000694	MED4	ENSDARG00000041503	ENSAMXG00000012890	ENSGACG00000008017	ENSTNIG00000003778	ENSTRUG00000014013	ENSPFOG00000010829	ENSXMAG00000008718	ENSORLG00000014076	ENSONIG00000016177	gi_584023652	gi_499031293	gi_548532252	gi_554866935	GO:0030521	genomic
MED12	ENSLOCG00000015382	MED12	ENSDARG00000056800	ENSAMXG00000011603	ENSGACG00000017493	ENSTNIG00000015746	ENSTRUG00000012646	ENSPFOG00000000954	ENSXMAG00000000274	ENSORLG00000000784	ENSONIG00000002455	gi_584017139	gi_498964171	gi_548340722	gi_554804436	GO:0030521	genomic
MED13	ENSLOCG00000003727	MED13A	ENSDARG00000053884	ENSAMXG00000000953	ENSGACG00000020213	ENSTNIG00000009033	ENSTRUG00000008851	ENSPFOG00000024209	ENSXMAG00000000108	ENSORLG00000012337	ENSONIG00000017544	gi_573902086	gi_499021858	gi_548408649	gi_554832637	GO:0030521	genomic
		MED13B	ENSDARG00000003910	ENSAMXG00000002817	ENSGACG00000010389	ENSTNIG00000003289	ENSTRUG00000007405	ENSPFOG00000009647	ENSXMAG00000018016	ENSORLG00000005489	ENSONIG00000011173	gi_573890035	gi_499014114	gi_548404206	gi_554855577	GO:0030521	genomic
MED14	ENSLOCG00000002286	MED14	ENSDARG00000009953	ENSAMXG00000002581	ENSGACG00000002015	ENSTNIG00000014969	ENSTRUG00000013609	ENSPFOG00000013987	ENSXMAG00000018007	ENSORLG00000010808	ENSONIG00000006848	gi_583985895	gi_499034639	gi_548378507	gi_554851281	GO:0030521	genomic
MED16	ENSLOCG00000001808	MED16	ENSDARG00000040779	ENSAMXG00000018838	ENSGACG00000013385	ENSTNIG00000004736	ENSTRUG00000007850	ENSPFOG00000008857	ENSXMAG00000010740	ENSORLG00000000109	ENSONIG00000004564	gi_583990837	gi_499010815	gi_548370249	gi_554824751	GO:0030521	genomic
MED17	ENSLOCG00000007406	MED17	ENSDARG00000006345	ENSAMXG00000016638	ENSGACG00000013203	ENSTNIG00000006440	ENSTRUG00000004276	ENSPFOG00000017880	ENSXMAG00000007752	ENSORLG00000000709	ENSONIG00000001372	gi_584012931	gi_499047177	gi_548396051	gi_554887944	GO:0030521	genomic
MED24	ENSLOCG00000012864	MED24	ENSDARG00000032459	ENSAMXG00000012180	ENSGACG00000008529	ENSTNIG00000012846	ENSTRUG00000016382	ENSPFOG00000016585	ENSXMAG00000011374	ENSORLG00000008227	ENSONIG00000019926	gi_584012330	gi_499023404	gi_548338012	gi_554858967	GO:0030521	genomic
MED30	ENSLOCG00000003235	MED30	ENSDARG00000029459	ENSAMXG00000021203	ENSGACG00000003014	ENSTNIG00000000448	ENSTRUG00000003806	ENSPFOG00000000737	ENSXMAG00000014029	ENSORLG00000001732	ENSONIG00000013534	gi_583998619	gi_498976995	gi_548477220	gi_554852211	GO:0030521	genomic
NCOA1	ENSLOCG00000015891	NCOA1	ENSDARG00000018257	ENSAMXG00000010390	ENSGACG00000006727	ENSTNIG00000019142	ENSTRUG00000006619	ENSPFOG00000004231	ENSXMAG00000017782	scaffold1294_contig11304	ENSONIG00000015724	gi_584022154	gi_498984261	gi_548530893	gi_554840198	NetPath, GO:0030521	genomic, positive regulator
NCOA3	ENSLOCG00000003989	NCOA3A		ENSAMXG00000008788<br>ENSAMXG00000008791<br>ENSAMXG00000008794	ENSGACG00000015221	ENSTNIG00000012601	ENSTRUG00000004450	ENSPFOG00000011587	ENSXMAG00000017470	ENSORLG00000012143	ENSONIG00000017075	gi_584003095	gi_498965809	gi_548366017	gi_554831335	NetPath, GO:0030521	genomic, positive regulator
		NCOA3B	ENSDARG00000077404	ENSAMXG00000006902	ENSGACG00000010036	ENSTNIG00000007303	ENSTRUG00000009516	ENSPFOG00000006902	ENSXMAG00000001970	ENSORLG00000013704	ENSONIG00000020313	gi_583978807	gi_499042642	gi_548447463	gi_554808986	NetPath, GO:0030521	genomic, positive regulator
NCOA4	ENSLOCG00000012898	NCOA4	ENSDARG00000021439	ENSAMXG00000015735	ENSGACG00000012317	ENSTNIG00000013384	ENSTRUG00000003924	ENSPFOG00000005630	ENSXMAG00000002456	ENSORLG00000003269	ENSONIG00000007129	gi_584024662	gi_499046546	gi_548457815	gi_554883384	NetPath, GO:0030521	genomic, positive regulator
NKX3.1	ENSLOCG00000015015	NKX3.1	ENSDARG00000078280	ENSAMXG00000024837	ENSGACG00000010776	SCAF14652	NA	ENSPFOG00000009654	ENSXMAG00000020262	ENSORLG00000010925	ENSONIG00000011762	gi_583977526	gi_498955057	gi_548354440	gi_554824085	GO:0030521	genomic
NR1I3	NA	NR1I3	NA	NA	NA	NA	NA	NA	NA	NA	NA					GO:0030521	genomic
NRIP1	ENSLOCG00000000906	NRIP1A	ENSDARG00000068965	ENSAMXG00000025365												GO:0030521	genomic
		NRIP1B	ENSDARG00000068894	ENSAMXG00000025737	ENSGACG00000020154		ENSTRUG00000004236<br>ENSTRUG00000000031	ENSPFOG00000017715	ENSXMAG00000014033	ENSORLG00000000586	ENSONIG00000015866	gi_584025409	gi_499021768	gi_548408144	gi_554851579	GO:0030521	genomic
PIAS1	ENSLOCG00000013933	PIAS1A	ENSDARG00000036094	ENSAMXG00000006151	ENSGACG00000016894	ENSTNIG00000003928<br>ENSTNIG00000009549	ENSTRUG00000016843	ENSPFOG00000006900	ENSXMAG00000017552	ENSORLG00000002077	ENSONIG00000013251	gi_583997712	gi_499013515	gi_548448896	gi_554840096	NetPath, GO:0030521	genomic, negative regulator
		PIAS1B	ENSDARG00000062445	ENSAMXG00000002784	ENSGACG00000005176	ENSTNIG00000008434	ENSTRUG00000015008	ENSPFOG00000009110	ENSXMAG00000003583	ENSORLG00000006728	ENSONIG00000015473	gi_583988189	gi_498993905	gi_548355942	gi_554863406	NetPath, GO:0030521	genomic, negative regulator
PIAS2	ENSLOCG00000006647	PIAS2	ENSDARG00000092979	ENSAMXG00000002435	ENSGACG00000005099	ENSTNIG00000003200	ENSTRUG00000013569	ENSPFOG00000000261	scaffold_JH558015.1	ENSORLG00000017353	ENSONIG00000013927	gi_584015665	gi_499015732	gi_548377776	gi_554864979	NetPath, GO:0030521	genomic, negative regulator
PIK3R1	ENSLOCG00000010510	PIK3R1A	ENSDARG00000038524	ENSAMXG00000011959	ENSGACG00000015820	ENSTNIG00000013464	ENSTRUG00000007584	ENSPFOG00000001009	ENSXMAG00000018595	ENSORLG00000002670	ENSONIG00000011065	gi_584003878	gi_498981102	gi_548334788	gi_554838849	NetPath, Bennett et al 2010, Foradori et al 2008	non-genomic
		PIK3R1B			ENSGACG00000001062	ENSTNIG00000018878	ENSTRUG00000010930	ENSPFOG00000015835	ENSXMAG00000015104	ENSORLG00000017457	ENSONIG00000001749	gi_573878953	gi_499049716	gi_548541918	gi_554876860	NetPath, Bennett et al 2010, Foradori et al 2008	non-genomic
PIK3R2	ENSLOCG00000001519	PIK3R2A	ENSDARG00000018060	XM_007236673.1	ENSGACG00000008201	ENSTNIG00000010197	ENSTRUG00000007054	ENSPFOG00000000170<br>ENSPFOG00000023056<br>ENSPFOG00000024225	ENSXMAG00000002484	LOC101163728	ENSONIG00000012700	gi_583993919	gi_499028291	gi_548356312	gi_554849340	NetPath, Bennett et al 2010, Foradori et al 2008	non-genomic
		PIK3R2B	NA	NA	ENSGACG00000015520	ENSTNIG00000011070	ENSTRUG00000010476	ENSPFOG00000003942	ENSXMAG00000013889	NA	ENSONIG00000010477	gi_583983019	gi_498942969	gi_548336831	gi_554819907	NetPath, Bennett et al 2010, Foradori et al 2008	non-genomic
PMEPA1	NA	PMEPA1	ENSDARG00000039325	ENSAMXG00000013100	ENSGACG00000003728	NA	NA	ENSPFOG00000001516	ENSXMAG00000010456	ENSORLG00000012800	ENSONIG00000004438	gi_573906092	gi_494461325	gi_545793510	gi_545786152	GO:0030521	genomic
PPAP2A	NA	PPAP2A	ENSDARG00000053381	ENSAMXG00000018732	NA	NA	NA	ENSPFOG00000003517	ENSXMAG00000004780	ENSORLG00000005464	ENSONIG00000007599	gi_583987884	gi_498980257	gi_548335165	gi_554813089	GO:0030521	genomic
PPARGC1A	ENSLOCG00000012350	PPARGC1A	ENSDARG00000067829	ENSAMXG00000002461	ENSGACG00000019546	ENSTNIG00000002174	ENSTRUG00000007210	ENSPFOG00000001263	ENSXMAG00000007748	ENSORLG00000004788	ENSONIG00000007635	gi_584018961	gi_498999424	gi_548425231	gi_554850044	GO:0030521	genomic
PTEN	ENSLOCG00000006145	PTENA	ENSDARG00000071018	ENSAMXG00000010994	ENSGACG00000009998	ENSTNIG00000005984	NA	ENSPFOG00000004955	ENSXMAG00000014454	ENSORLG00000020711	ENSONIG00000019123	gi_583996646	gi_499029140	gi_548428164	gi_554877417	NetPath	non-genomic
		PTENB	ENSDARG00000056623	ENSAMXG00000007753	ENSGACG00000009236	ENSTNIG00000013318	ENSTRUG00000009851	ENSPFOG00000015747	ENSXMAG00000012957	ENSORLG00000006231	ENSONIG00000000386	gi_583976942	gi_498987823	gi_548350440	gi_554806495	NetPath	non-genomic
PTK2A	ENSLOCG00000007702	PTK2AA	ENSDARG00000040197	ENSAMXG00000018637	ENSGACG00000004011<br>ENSGACG00000004003	ENSTNIG00000009167<br>ENSTNIG00000009166	ENSTRUG00000007362	ENSPFOG00000003397	ENSXMAG00000015476	scaffold5001_contig12887	ENSONIG00000002815	gi_583985834	gi_498978332	gi_548352657	gi_554813630	NetPath	non-genomic
		PTK2AB	ENSDARG00000004672	ENSAMXG00000004491	ENSGACG00000007004	ENSTNIG00000009636	ENSTRUG00000011167	ENSPFOG00000010090	ENSXMAG00000010911	ENSORLG00000004751	ENSONIG00000006838	gi_573896657	gi_499029030	gi_548435043	gi_554849438	NetPath	non-genomic
PTK2B	ENSLOCG00000017241	PTK2BB	ENSDARG00000039577	ENSAMXG00000019303	ENSGACG00000010609	ENSTNIG00000016347	ENSTRUG00000008406	ENSPFOG00000008438	ENSXMAG00000004048	ENSORLG00000008993	ENSONIG00000015297	gi_584004629	gi_498939798	gi_548381009	gi_554807097	NetPath	non-genomic
		PTK2BA	ENSDARG00000022841	ENSAMXG00000000798	ENSGACG00000014838	ENSTNIG00000014508	ENSTRUG00000017337	ENSPFOG00000009145	ENSXMAG00000004406	ENSORLG00000005884	ENSONIG00000015175	gi_584025201	gi_499049776	gi_545793246	gi_554877844	NetPath	non-genomic
RAC1	ENSLOCG00000008397	RAC1AA	ENSDARG00000074849<br>ENSDARG00000099506<br>ENSDARG00000087954<br>ENSDARG00000013203<br>ENSDARG00000096666<br>ENSDARG00000096549<br>ENSDARG00000054649	ENSAMXG00000008935	ENSGACG00000011601	ENSTNIG00000017983	ENSTRUG00000016201	ENSPFOG00000004946	ENSXMAG00000001114	ENSORLG00000008757<br>ENSORLG00000008763	ENSONIG00000017149	gi_583992724	gi_499014994	gi_548382840	gi_554829712	NetPath	non-genomic
		RAC1AB								ENSORLG00000008757<br>ENSORLG00000008763	ENSONIG00000017151	gi_583992732	gi_499014998	gi_548382828	gi_554829718	NetPath	non-genomic
		RAC1B			ENSGACG00000009531	ENSTNIG00000012875	ENSTRUG00000016765	ENSPFOG00000015316<br>ENSPFOG00000015319<br>ENSPFOG00000015350	ENSXMAG00000011073<br>ENSXMAG00000011081<br>ENSXMAG00000011307<br>ENSXMAG00000011324	ENSORLG00000003527	ENSONIG00000019847	gi_584012219	gi_499023276	gi_548338137	gi_554845115	NetPath	non-genomic

RAF1	ENSLOCG00000013974	RAF1	ENSDARG00000059406<br>ENSDARG00000096415	ENSAMXG00000005440<br>ENSAMXG00000001055	ENSGACG00000000060	ENSTNIG00000012621	ENSTRUG00000006611	ENSPFOG00000015974	ENSXMAG00000017691	ENSORLG00000011471	ENSONIG00000017170	gi_584003028	gi_498965606	gi_548483383	gi_554857802	Foradori et al 2008	non-genomic
RAN	ENSLOCG00000008279	RAN	ENSDARG00000057026	ENSAMXG00000015390	ENSGACG00000017755	ENSTNIG00000013137	ENSTRUG00000013749	ENSPFOG00000009552	ENSXMAG00000009922	ENSORLG00000003452	ENSONIG00000005139	gi_583972026	gi_498962091	gi_548341336	gi_554805068	NetPath, GO:0030521	genomic, positive regulator
RB1	ENSLOCG00000008882	RB1	ENSDARG00000006782	ENSAMXG00000005363	ENSGACG00000020379	ENSTNIG00000011786	ENSTRUG00000015842	ENSPFOG00000003889	ENSXMAG00000015729	ENSORLG00000008317	ENSONIG00000004276	gi_584014918	gi_499026357	gi_548387323	gi_554822936	NetPath, GO:0030521	genomic, positive regulator
RHOAA	ENSLOCG00000014301	RHOAAA	ENSDARG00000026845	ENSAMXG00000014632				ENSPFOG00000017531	ENSXMAG00000004466		ENSONIG00000017974	gi_584019304	gi_498964222	gi_548443896	gi_554866988	NetPath	non-genomic
		RHOAAB	ENSDARG00000015429		ENSGACG00000009938	ENSTNIG00000015015	ENSTRUG00000010405				ENSONIG00000012367	gi_583980905	gi_499016231	gi_548370620	gi_554807569	NetPath	non-genomic
RHOAB	ENSLOCG00000014438	RHOABA	ENSDARG00000094673	ENSAMXG00000005847	ENSGACG00000005670	ENSTNIG00000006051	ENSTRUG00000010942	ENSPFOG00000000160	ENSXMAG00000019368	ENSORLG00000002742	ENSONIG00000001670	gi_583980256	gi_498997689	gi_548357349	gi_554857836	NetPath	non-genomic
		RHOABB		ENSAMXG00000008373	ENSGACG00000001206	ENSTNIG00000014672	ENSTRUG00000011944	ENSPFOG00000019065	ENSXMAG00000016892	ENSORLG00000018734	ENSONIG00000018884	gi_583976140	gi_498957597	gi_548414713	gi_554862978	NetPath	non-genomic
RHOB	ENSLOCG00000017876	RHOB		ENSAMXG00000026330	ENSGACG00000009667	ENSTNIG00000002278	ENSTRUG00000018178	ENSPFOG00000020611	ENSXMAG00000019632	ENSORLG00000012619	ENSONIG00000020448	gi_583994917	gi_499036281	gi_548358324	gi_554821869	NetPath	non-genomic
RNF4	ENSLOCG00000006957	RNF4	ENSDARG00000090993	ENSAMXG00000007606	ENSGACG00000016217	SCAF7445	ENSTRUG00000017344	ENSPFOG00000017123	ENSXMAG00000015165	ENSORLG00000020573	gi_542256685	gi_583973152	gi_499025880	gi_548333174	gi_554877732	NetPath, GO:0030521	genomic, positive regulator
RNF14	ENSLOCG00000010578	RNF14A	ENSDARG00000002794	ENSAMXG00000020091	ENSGACG00000020731	ENSTNIG00000018203	ENSTRUG00000008815	ENSPFOG00000006823	ENSXMAG00000001585	ENSORLG00000001462	ENSONIG00000008460	gi_583979411	gi_498982921	gi_548351054	gi_545785817	GO:0030521	genomic
		RNF14B	ENSDARG00000043905<br>ENSDARG00000078683<br>ENSDARG00000090710<br>ENSDARG00000091727		ENSGACG00000006180	ENSTNIG00000006782	ENSTRUG00000004900	ENSPFOG00000019210	ENSXMAG00000002519	ENSORLG00000011566						GO:0030521	genomic
ROCK1	ENSLOCG00000001989	ROCK1	ENSDARG00000058993	ENSAMXG00000014672	ENSGACG00000017327	ENSTNIG00000008471	ENSTRUG00000013281	ENSPFOG00000011127	ENSXMAG00000002398	ENSORLG00000017642	gi_542206027	gi_583994033	gi_498945850	gi_548422536	gi_554803283	NetPath	non-genomic
ROCK2	ENSLOCG00000016666	ROCK2A	ENSDARG00000017500	ENSAMXG00000021207	ENSGACG00000012788	ENSTNIG00000008912	ENSTRUG00000010769	ENSPFOG00000012091<br>ENSPFOG00000012199	ENSXMAG00000009185	ENSORLG00000018255	gi_542208870	gi_584008970	gi_499017063	gi_548401842	gi_545786390	NetPath	non-genomic
		ROCK2B	ENSDARG00000004877	ENSAMXG00000003869	ENSGACG00000009555	ENSTNIG00000012308	ENSTRUG00000018218	ENSPFOG00000019296	ENSXMAG00000013653	ENSORLG00000012731	gi_542255251	gi_583995367	gi_499036295	gi_548358264	gi_554821891	NetPath	non-genomic
SCGB2A1	NA	SCGB2A1	NA	NA	NA	NA	NA	NA	NA	NA	NA	NA	NA	NA	NA	GO:0030521	genomic
SRC	ENSLOCG00000002309	SRCA		ENSAMXG00000014517	ENSGACG00000005548	ENSTNIG00000018270	ENSTRUG00000018366	ENSPFOG00000013135	ENSXMAG00000010238	ENSORLG00000006714	ENSONIG00000006882	gi_583974949	gi_498971516	gi_548355415	gi_554866558	NetPath, Bennett et al 2010, Foradori et al 2008	non-genomic
		SRCB	ENSDARG00000008107	ENSAMXG00000001275	ENSGACG00000011349	ENSTNIG00000019527	ENSTRUG00000005980	ENSPFOG00000000684	ENSXMAG00000001476	ENSORLG00000016183	ENSONIG00000016547	gi_583997963	gi_499019153	gi_548412532	gi_554816133	NetPath, Bennett et al 2010, Foradori et al 2008	non-genomic
TGFB1I1	Scaffold_JH591692.1	TGFB1I1		ENSAMXG00000020290	ENSGACG00000007235	ENSTNIG00000018936	ENSTRUG00000005520	ENSPFOG00000007368	ENSXMAG00000018458	ENSORLG00000005775	ENSONIG00000011521	gi_584022201	gi_499048970	gi_548490449	gi_554875222	NetPath, GO:0030521	genomic, positive regulator
THRAP3	ENSLOCG00000002939	THRAP3A	ENSDARG00000040095<br>ENSDARG00000044459	ENSAMXG00000001863	ENSGACG00000013745	ENSTNIG00000012194	ENSTRUG00000010702	ENSPFOG00000013297	ENSXMAG00000014797	ENSORLG00000014550	gi_542234433	gi_583993177	gi_498985480	gi_548389984	gi_554830071	GO:0030521	genomic
		THRAP3B	ENSDARG00000003513	ENSAMXG00000020877	ENSGACG00000002319	ENSTNIG00000004582	ENSTRUG00000010336	ENSPFOG00000000525	ENSXMAG00000017962	ENSORLG00000009543	ENSONIG00000013673	gi_583998686	gi_498976772	gi_548406882	gi_554857382	GO:0030521	genomic
UBE3A	ENSLOCG00000008388	UBE3A	ENSDARG00000055737	ENSAMXG00000012931	ENSGACG00000015321	ENSTNIG00000014475	ENSTRUG00000006347	ENSPFOG00000018875	ENSXMAG00000009731	ENSORLG00000020667	ENSONIG00000003746	gi_584015939	gi_499026835	gi_548467978	gi_554857247	NetPath, GO:0030521	genomic, positive regulator

Table S2
Results of jmodeltest on each coding sequence alignment indicating the best fitting nucelotide substitution model per gene

Tree	Best model according to AICc
akt1	GTR+G
ar	GTR+G+I
arid1a	GTR+G+I
brca1	GTR+G+I
cav1	HKY+I
ccne1	GTR+G+I
cdc42	SYM+G+I
cdk7	GTR+G+I
cfl1l	GTR+G
ctnnb1	GTR+G+I
daxx	GTR+G+I
dnaja1	GTR+G+I
egfr	GTR+G+I
fhl2	GTR+G
fkbp4	GTR+G+I
flna	GTR+G+I
gnb2l1	GTR+G+I
grip1	GTR+G
kat5	GTR+G+I
limk2	GTR+G+I
mapk1	GTR+G+I
mapk3	GTR+G
med1	GTR+G+I
med4	GTR+G
med12	GTR+G+I
med13	GTR+G+I
med14	GTR+G+I
med16	GTR+G+I
med17	GTR+G+I
med24	GTR+G+I
med30	HKY+G
ncoa1	HKY+G
ncoa3	GTR+G+I
ncoa4	GTR+G+I
nkx3-1	HKY+G+I
nrip1	GTR+G+I
pias1	HKY+G+I
pias2	GTR+G+I
pik3r1	GTR+G+I
pik3r2	GTR+G+I
pmepa1	HKY+G+I
ppap2a	GTR+G
ppargc1a	GTR+G
pten	GTR+G+I
ptk2a	GTR+G
ptk2b	GTR+G+I
rac1	SYM+G
raf1	GTR+G+I
ran	SYM+G+I
rb1	HKY+G
rhoaa	GTR+G+I
rhoab	GTR+G+I
rhob	HKY+G
rnf14	GTR+G+I
rnf4	GTR+G+I
rock1	GTR+G+I
rock2	GTR+G+I
src	GTR+G+I
tgfb1i1	GTR+G
thrap3	GTR+G+I
ube3a	GTR+G+I

Table S3

p-values of two-sided Welch's t-test of Ka/Ks comparisons Lake cichlids *vs.* Nile tilapia for TSGD gene copy A *vs.* gene copy B as shown in Figure 3

**Gene**	**p-value**
ar	1.26E-06
arid1a	0.3198596
cdc42	NA
egfr	0.1058882
fhl2	0.0004413
med13	0.0017968
ncoa3	0.0335987
pias1	0.0003377
pik3r1	0.0240627
pik3r2	0.0063452
pten	0.0122406
ptk2a	0.3775205
ptk2b	7.81E-05
rac1a	7.16E-06
rhoaa	0.0052176
rhoab	NA
rock2	0.0003164
src	0.0040781
thrap3	0.0004625

Table S4
p-values of two-sided Welch's t-test for comparisons of FPKM values of TSGD duplicate genes in four cichlid species in brain, ovary and testis as shown in Figure 4

		**Gene**																		
**Tissue**	**Species**	*ar*	*cdc42*	*egfr*	*med13*	*pik3r1*	*pik3r2*	*pten*	*ptk2a*	*ptk2b*	*rac1a*	*rhoaa*	*rhoab*	*rock2*	*src*	*thrap3*	*arid1a*	*fhl2a*	*ncoa3*	*pias1*
Brain	*A. burtoni*	**0.00346**	0.05275	**0.00049**	**0.00000**	**0.00002**	**0.00256**	**0.01560**	0.13330	**0.01368**	**0.00001**	**0.00023**	**0.00023**	0.34635	**0.00033**	**0.00005**	**0.00484**	**0.00829**	**0.00065**	**0.01194**
Brain	*E. cyanostictus*	**0.00045**	**0.00008**	**0.00006**	**0.00001**	**0.00000**	**0.00035**	**0.00000**	0.10212	**0.01937**	**0.00000**	**0.00000**	**0.00300**	**0.00566**	**0.00000**	**0.00000**	**0.00019**	0.08535	**0.00006**	**0.00000**
Brain	*J. ornatus*	**0.01436**	**0.00000**	**0.00075**	**0.00000**	0.26959	**0.00000**	**0.00000**	0.66626	**0.00638**	**0.00000**	**0.00000**	**0.00073**	**0.00013**	**0.00002**	**0.00000**	0.21432	0.90236	**0.00000**	**0.00002**
Brain	*O. ventralis*	**0.00024**	**0.03156**	**0.00004**	**0.00003**	0.05098	0.16351	**0.00000**	**0.01268**	**0.02682**	**0.00000**	**0.00001**	**0.00246**	**0.04742**	**0.00113**	**0.00000**	**0.00024**	**0.00001**	**0.00000**	**0.00003**
Ovary	*A. burtoni*	0.40845	0.30001	0.29335	0.08093	**0.01684**	0.11070	**0.02884**	**0.02092**	0.32720	**0.01615**	0.99574	**0.03633**	0.06140	0.13777	0.06973	**0.03892**	0.16489	0.08868	0.17044
Ovary	*E. cyanostictus*	**0.03572**	**0.00000**	**0.01759**	0.14124	**0.00007**	0.08404	0.06825	0.24108	**0.01788**	**0.00001**	**0.00002**	**0.00000**	0.23874	**0.00018**	**0.00101**	**0.00005**	0.39100	**0.01284**	**0.00483**
Ovary	*J. ornatus*	0.20389	**0.00011**	**0.03002**	0.11506	**0.01300**	**0.03726**	**0.00926**	**0.02540**	0.66233	**0.00079**	**0.00016**	**0.00006**	0.47422	**0.00033**	**0.01942**	**0.00116**	**0.00561**	**0.00020**	0.10924
Ovary	*O. ventralis*	**0.01364**	**0.01686**	0.92110	0.44459	**0.00426**	0.28511	0.19111	**0.00001**	0.39317	**0.00114**	0.62893	**0.00186**	**0.00199**	**0.00903**	**0.00379**	**0.01364**	**0.01016**	**0.00651**	0.44459
Testis	*A. burtoni*	**0.01080**	**0.00154**	0.08909	**0.00005**	**0.00001**	**0.00759**	**0.00328**	**0.00055**	0.19324	**0.00073**	0.44422	**0.00701**	**0.00049**	**0.00013**	**0.00000**	**0.00023**	**0.00072**	**0.00029**	**0.01682**
Testis	*E. cyanostictus*	0.75190	**0.00007**	**0.00501**	0.29676	**0.02859**	**0.02680**	**0.02637**	**0.00001**	0.14388	**0.00009**	0.08819	0.05501	**0.00200**	**0.02596**	**0.00155**	0.52935	0.58034	**0.01598**	**0.02277**
Testis	*J. ornatus*	0.78731	**0.00002**	**0.00000**	**0.00006**	0.06754	**0.00616**	0.76850	**0.00885**	0.88815	**0.00181**	**0.00550**	**0.00681**	**0.00034**	0.50971	**0.00002**	**0.00341**	**0.01825**	**0.00189**	**0.00898**
Testis	*O. ventralis*	**0.02149**	**0.00885**	0.06161	0.06250	0.25395	**0.03823**	**0.03967**	**0.00582**	0.11568	**0.00258**	**0.00217**	0.07943	**0.00377**	0.68541	**0.00106**	**0.02149**	**0.00202**	**0.00002**	0.06250
