# Supplementary material for: Evolutionary Fate of the Androgen Receptor−Signaling Pathway in Ray-Finned Fishes with a Special Focus on Cichlids
Source: G3 (Bethesda). 2015 Sep 1;5(11):2275–83. doi: 10.1534/g3.115.020685 (PMC4632047; doi:10.1534/g3.115.020685)
Supplement: Supporting Information [file supp_g3.115.020685_FigureS1.pdf]

Figure S1

Maximum-likelihood single gene phylogenetic reconstructions for genes of the AR signaling pathway in ray-finned fishes using PhyML 3.1 (Guindon *et al.* 2010) under the GTR + gamma + I model, with 1,000 bootstrap replicates.

Figure S1

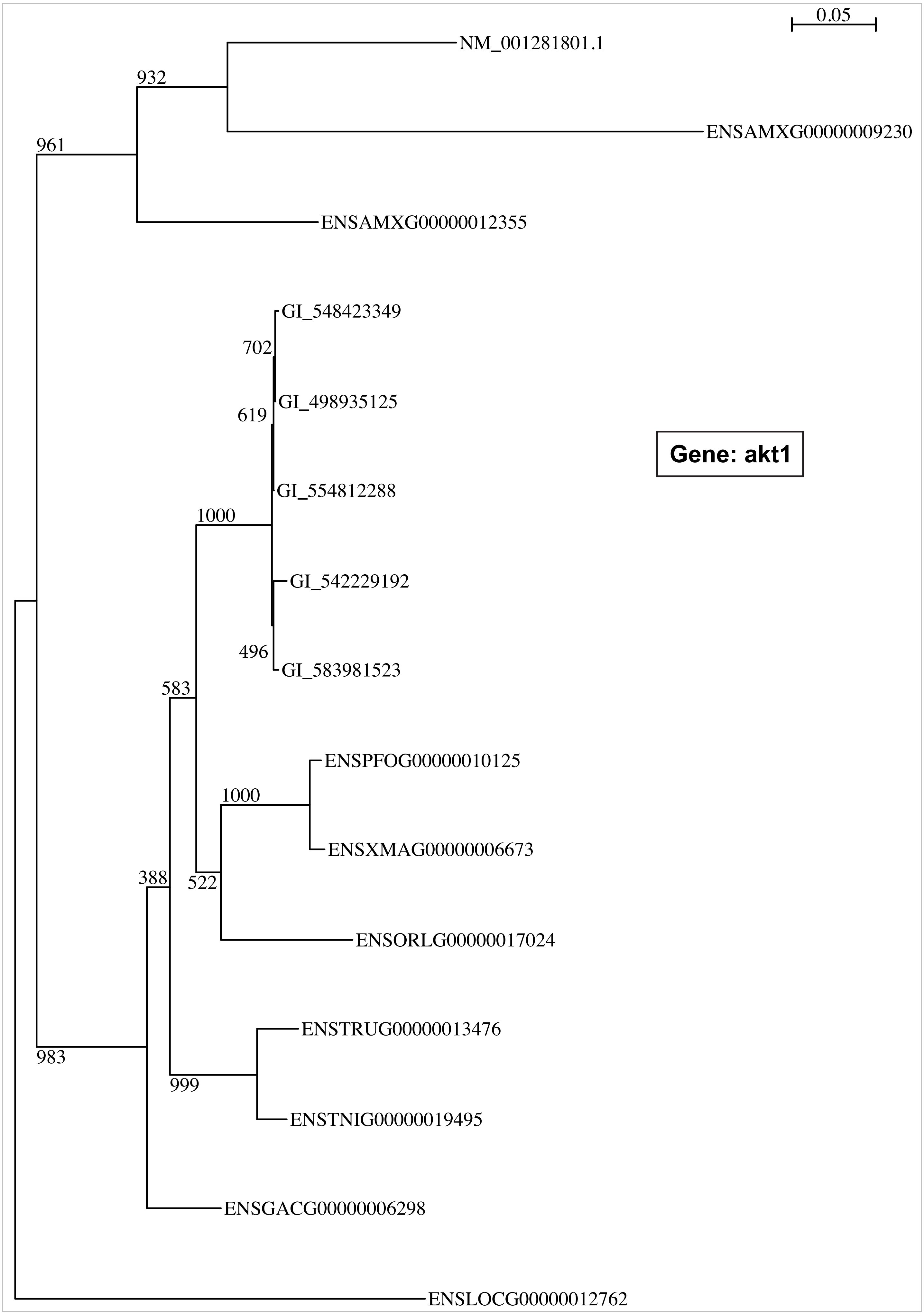
0.05
NM_001281801.1
932
ENSAMXG00000009230
961
ENSAMXG00000012355
GI_548423349
702
GI_498935125
619
Gene: akt1
GI_554812288
1000
GI_542229192
496
GI_583981523
583
ENSPFOG00000010125
1000
ENSXMAG00000006673
388
522
ENSORLG00000017024
ENSTRUG00000013476
983
999
ENSTNIG00000019495
ENSGACG00000006298
ENSLOCG00000012762

Figure S1

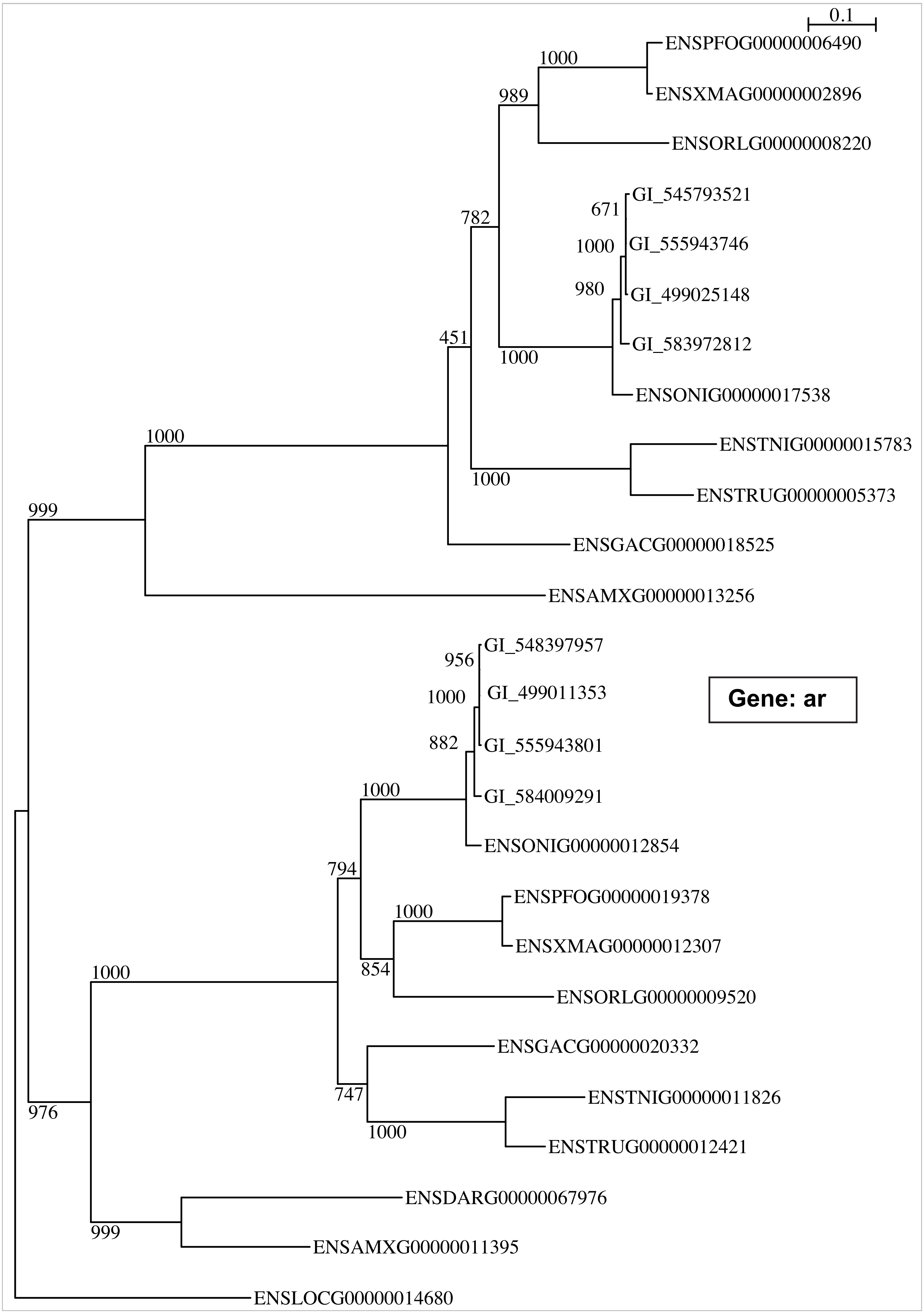

0.1
ENSPFOG00000006490
ENSXMAG00000002896
ENSORLG00000008220
GI_545793521
GI_555943746
GI_499025148
GI_583972812
ENSONIG00000017538
ENSTNIG00000015783
ENSTRUG00000005373
ENSGACG00000018525
ENSAMXG00000013256
GI_548397957
GI_499011353
GI_555943801
GI_584009291
ENSONIG00000012854
ENSPFOG00000019378
ENSXMAG00000012307
ENSORLG00000009520
ENSGACG00000020332
ENSTNIG00000011826
ENSTRUG00000012421
ENSDARG00000067976
ENSAMXG00000011395
ENSLOCG00000014680
1000
989
671
782
1000
980
451
1000
1000
1000
999
956
1000
882
1000
794
1000
854
1000
747
1000
976
999
Gene: ar

Figure S1

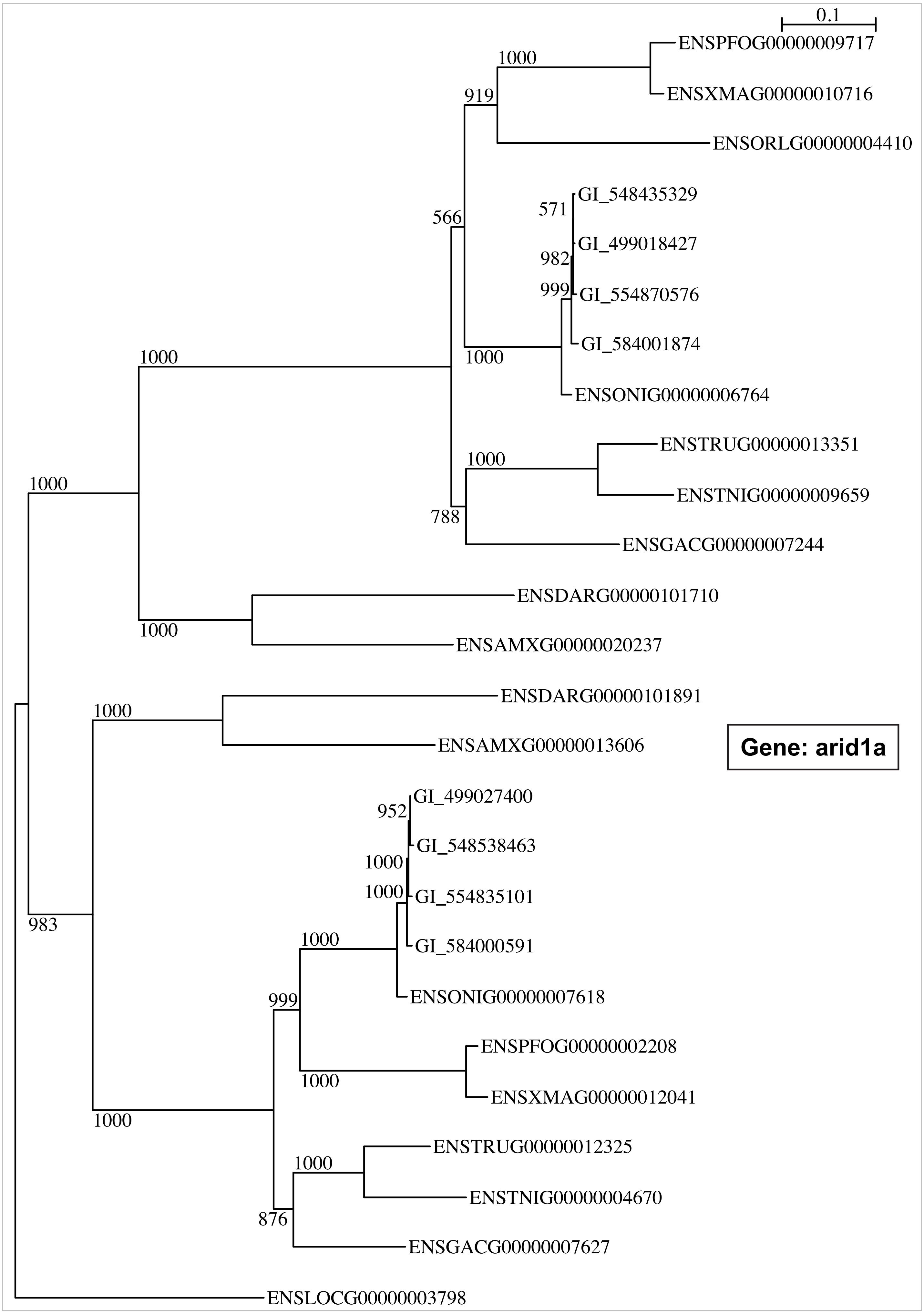
0.1
ENSPFOG00000009717
ENSXMAG00000010716
ENSORLG00000004410
GI_548435329
GI_499018427
GI_554870576
GI_584001874
ENSONIG00000006764
ENSTRUG00000013351
ENSTNIG00000009659
ENSGACG00000007244
ENSDARG00000101710
ENSAMXG00000020237
ENSDARG00000101891
ENSAMXG00000013606
Gene: arid1a
GI_499027400
GI_548538463
GI_554835101
GI_584000591
ENSONIG00000007618
ENSPFOG00000002208
ENSXMAG00000012041
ENSTRUG00000012325
ENSTNIG00000004670
ENSGACG00000007627
ENSLOCG00000003798
1000
919
571
566
982
999
1000
1000
1000
1000
788
1000
1000
983
952
1000
1000
1000
999
1000
1000
1000
876

Figure S1

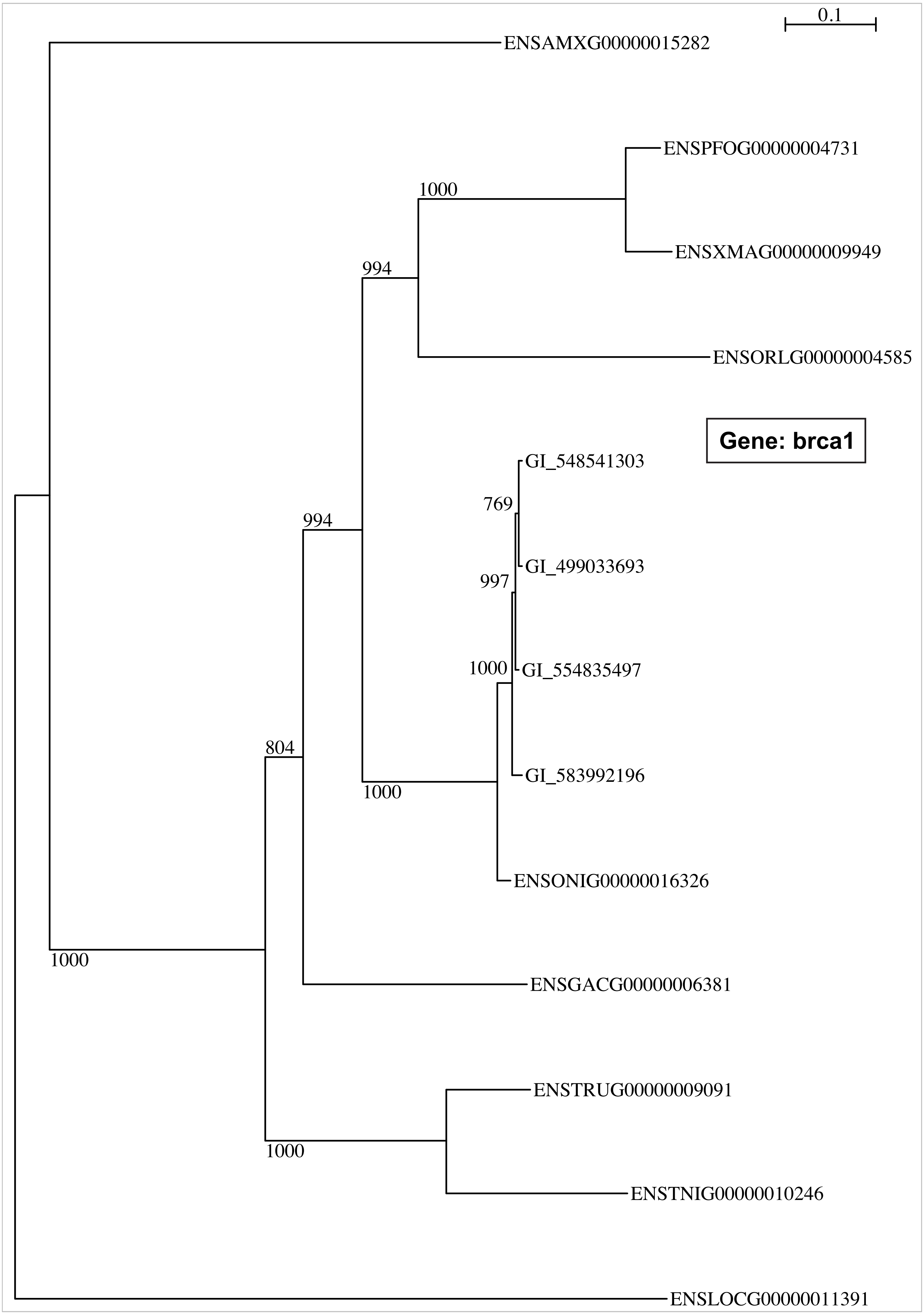

 T. Lorin, W. Salzburger & A. Böhne

Figure S1

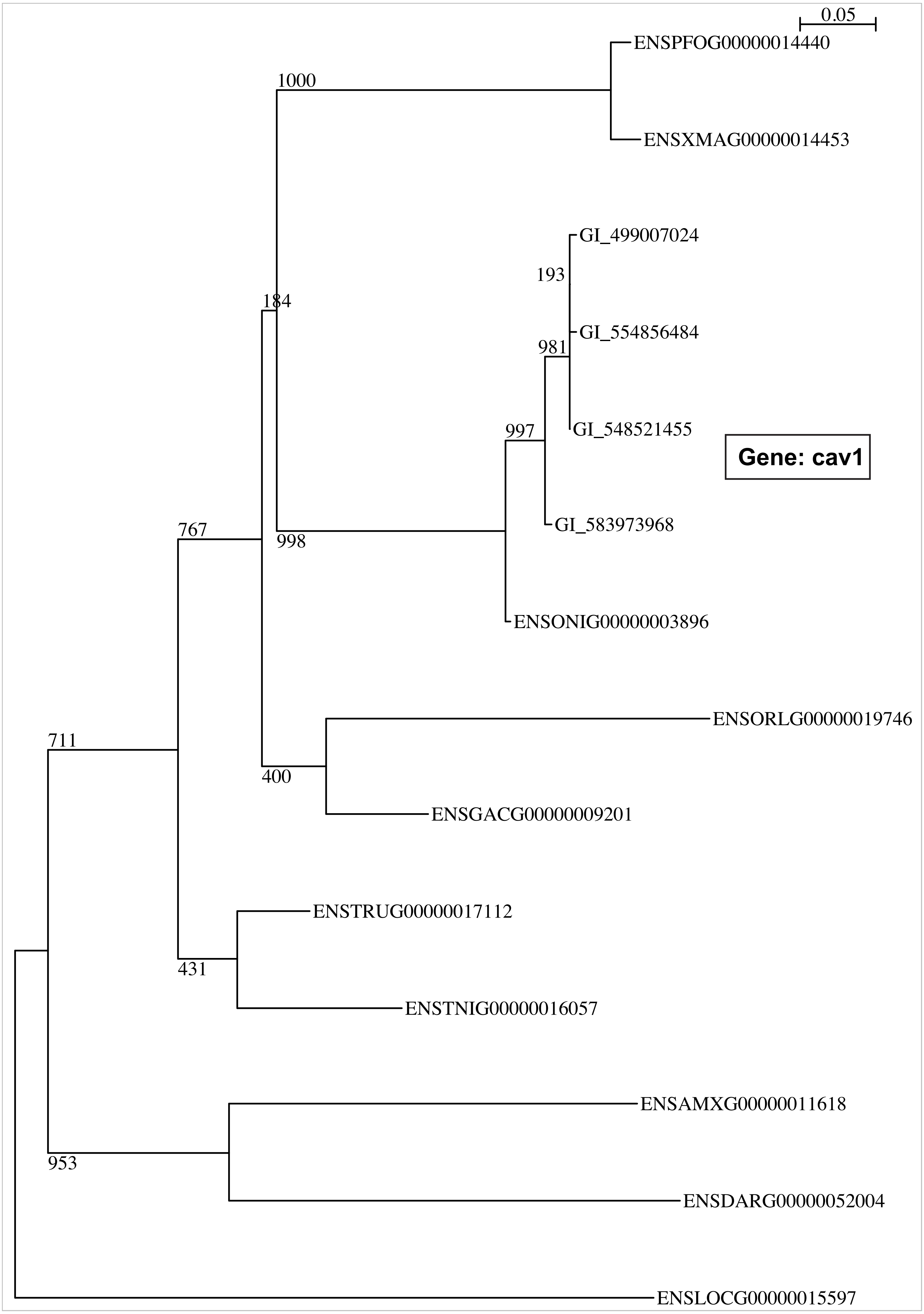
0.05
1000
ENSPFOG00000014440
ENSXMAG00000014453
GI_499007024
193
184
GI_554856484
981
997
GI_548521455
Gene: cav1
GI_583973968
767
998
ENSONIG00000003896
ENSORLG00000019746
711
400
ENSGACG00000009201
ENSTRUG00000017112
431
ENSTNIG00000016057
ENSAMXG00000011618
953
ENSDARG00000052004
ENSLOCG00000015597

Figure S1

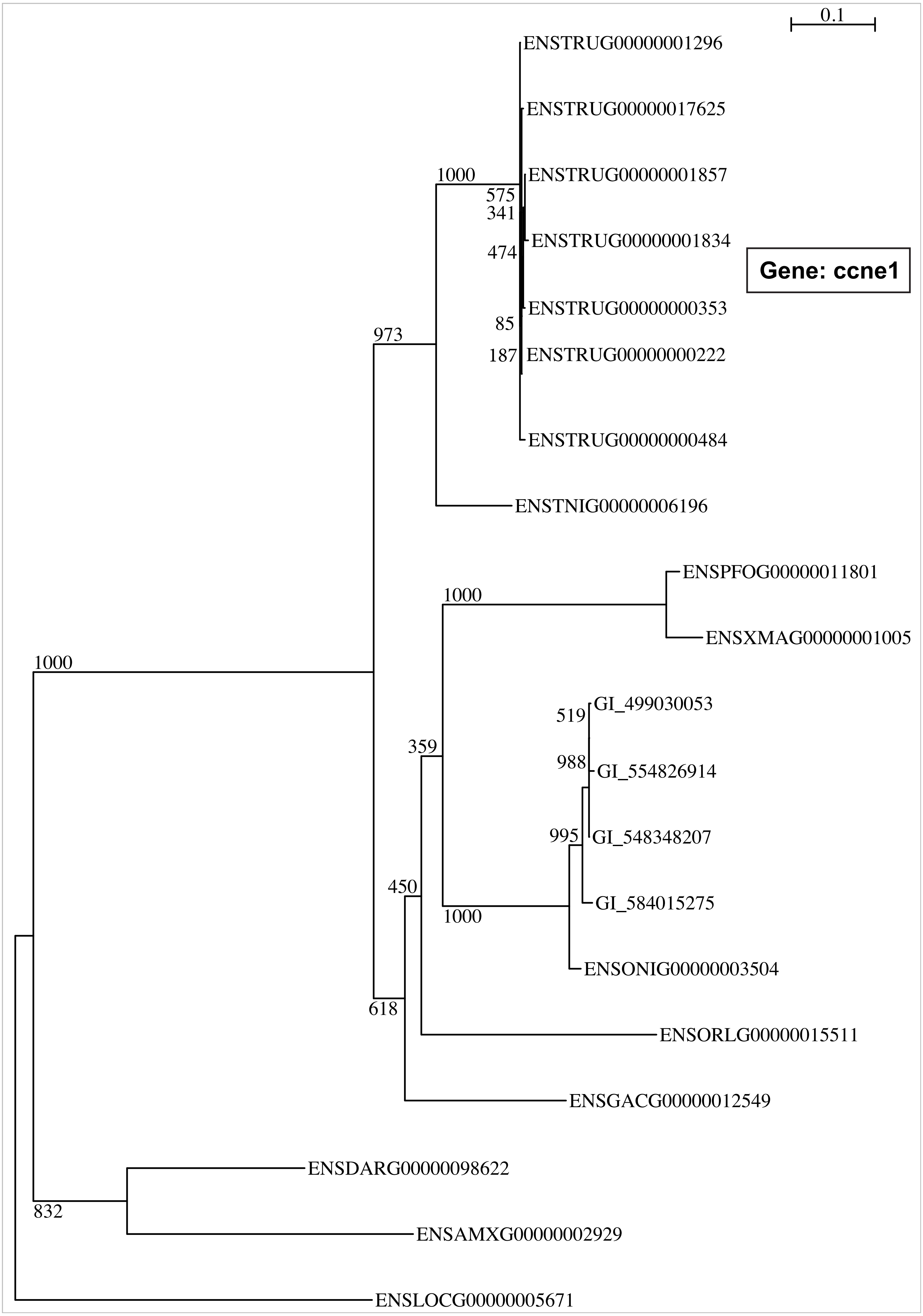
0.1
ENSTRUG00000001296
ENSTRUG00000017625
1000
ENSTRUG00000001857
575
341
ENSTRUG00000001834
474
Gene: ccne1
ENSTRUG00000000353
973
85
187
ENSTRUG00000000222
ENSTRUG00000000484
ENSTNIG00000006196
ENSPFOG00000011801
1000
ENSXMAG00000001005
1000
GI_499030053
519
359
988
GI_554826914
995
GI_548348207
450
GI_584015275
1000
ENSONIG00000003504
618
ENSORLG00000015511
ENSGACG00000012549
ENSDARG00000098622
832
ENSAMXG00000002929
ENSLOCG00000005671

Figure S1

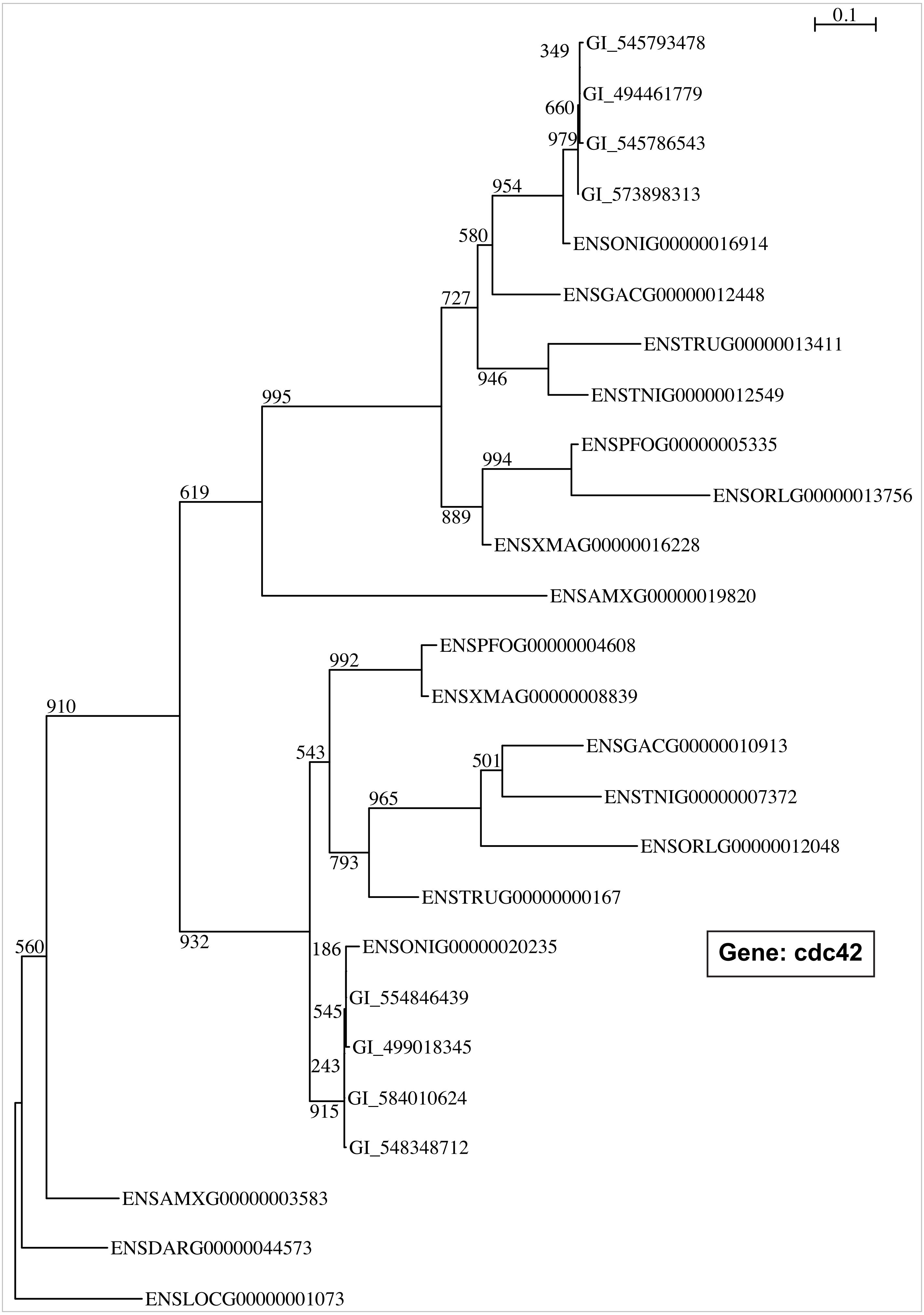

 T. Lorin, W. Salzburger & A. Böhne

Figure S1

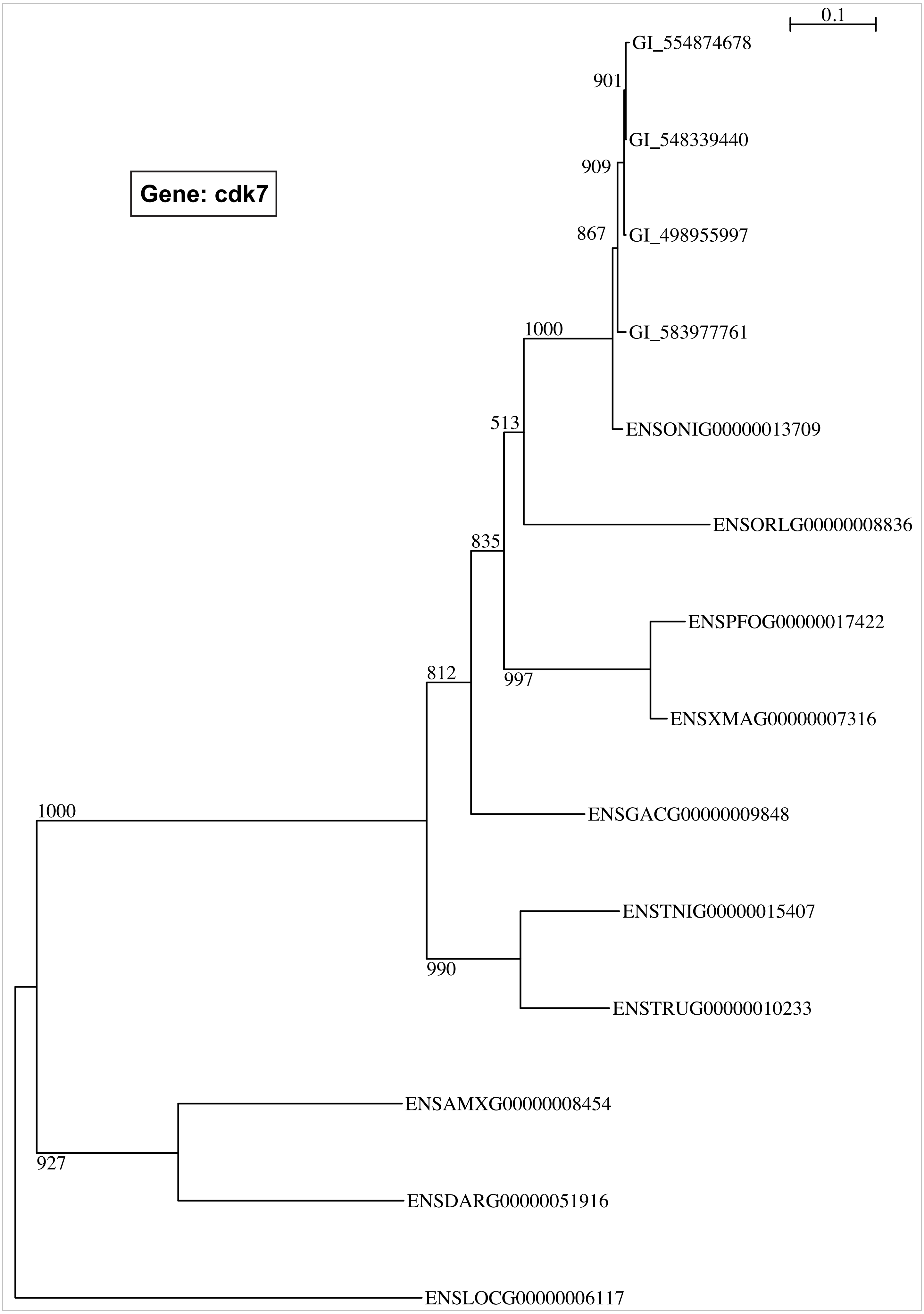
0.1
Gene: cdk7
GI_554874678
901
GI_548339440
909
GI_498955997
867
GI_583977761
1000
513
ENSONIG00000013709
ENSORLG00000008836
835
ENSPFOG00000017422
812
997
ENSXMAG00000007316
1000
ENSGACG00000009848
ENSTNIG00000015407
990
ENSTRUG00000010233
ENSAMXG00000008454
927
ENSDARG00000051916
ENSLOCG00000006117

Figure S1

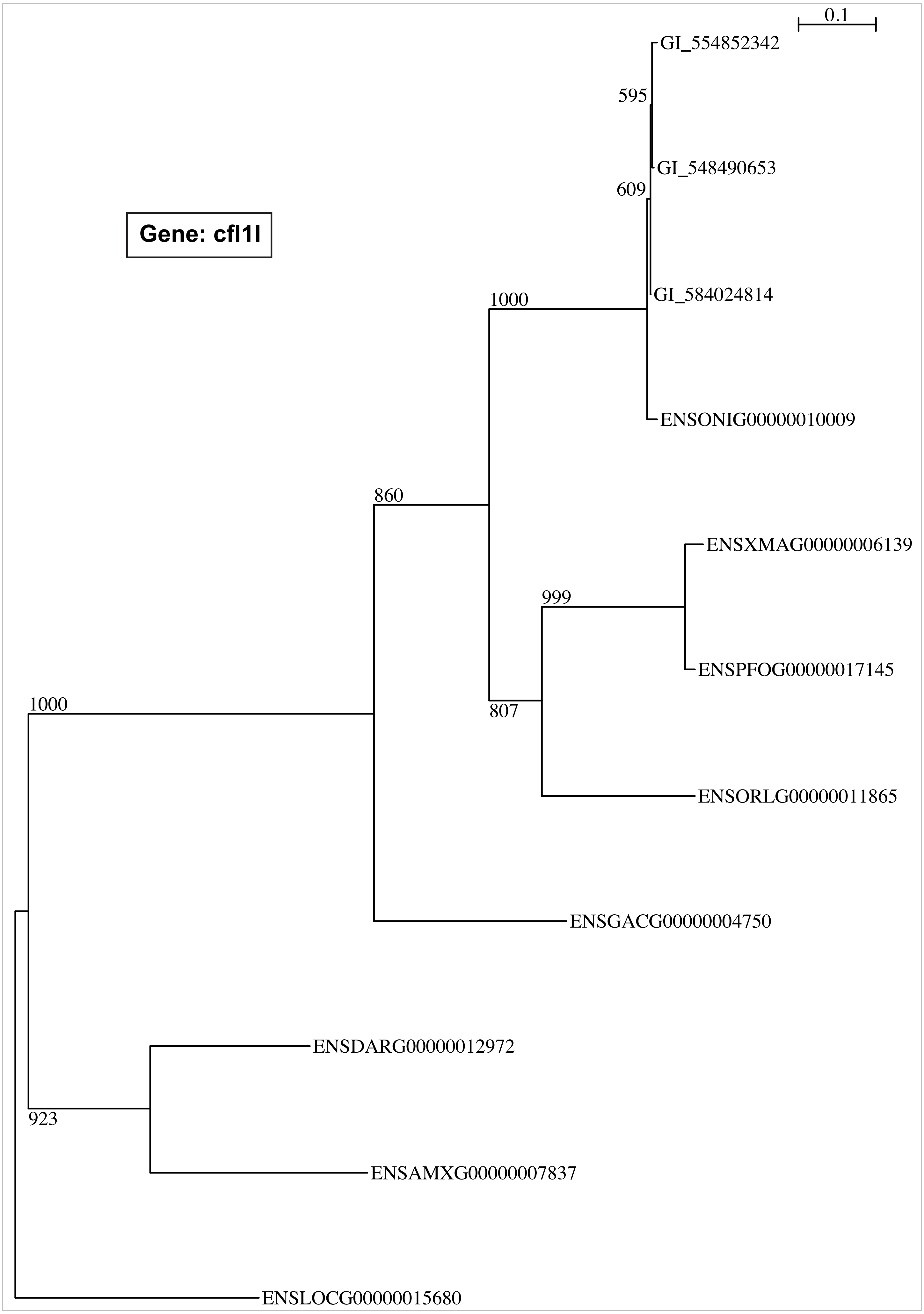

 T. Lorin, W. Salzburger & A. Böhne

Figure S1

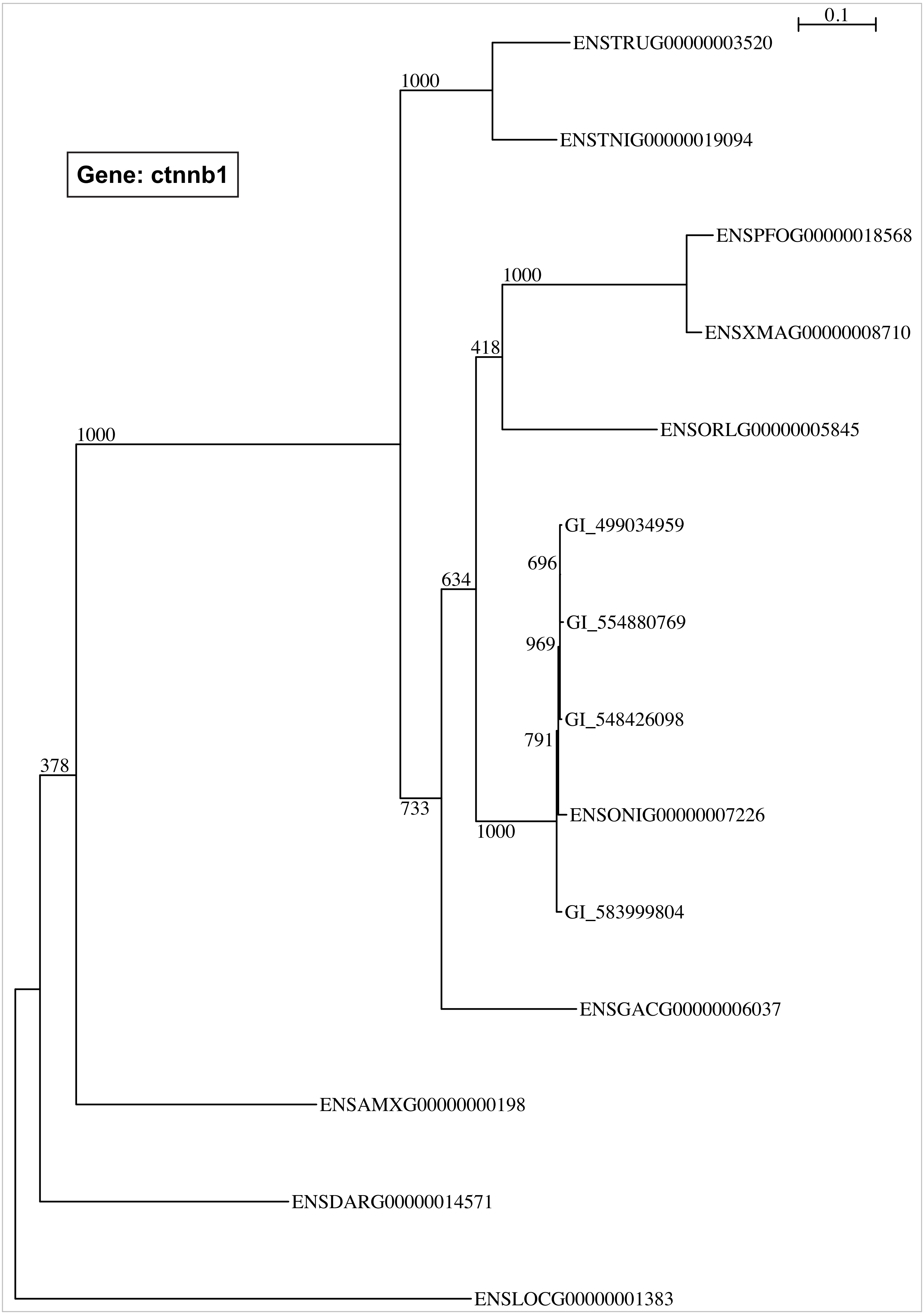
0.1
Gene: ctnnb1
1000
ENSTRUG00000003520
ENSTNIG00000019094
1000
ENSPFOG00000018568
ENSXMAG00000008710
418
ENSORLG00000005845
1000
GI_499034959
696
634
GI_554880769
969
GI_548426098
791
378
733
ENSONIG00000007226
1000
GI_583999804
ENSGACG00000006037
ENSAMXG00000000198
ENSDARG00000014571
ENSLOCG00000001383

Figure S1

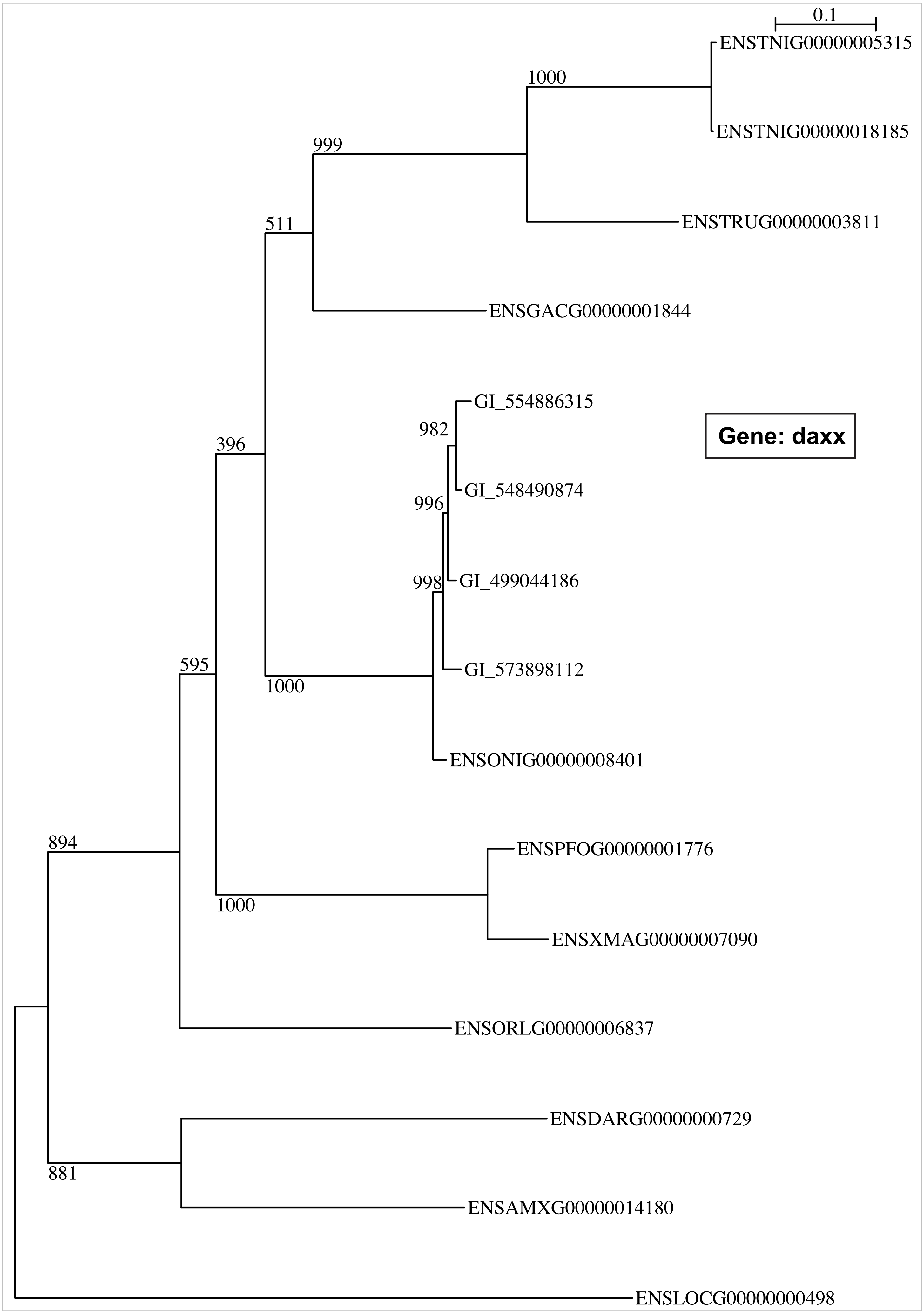
0.1
ENSTNIG00000005315
1000
ENSTNIG00000018185
999
ENSTRUG00000003811
511
ENSGACG00000001844
GI_554886315
982
Gene: daxx
396
GI_548490874
996
998
GI_499044186
595
GI_573898112
1000
ENSONIG00000008401
ENSPFOG00000001776
894
1000
ENSXMAG00000007090
ENSORLG00000006837
ENSDARG00000000729
881
ENSAMXG00000014180
ENSLOCG00000000498

Figure S1

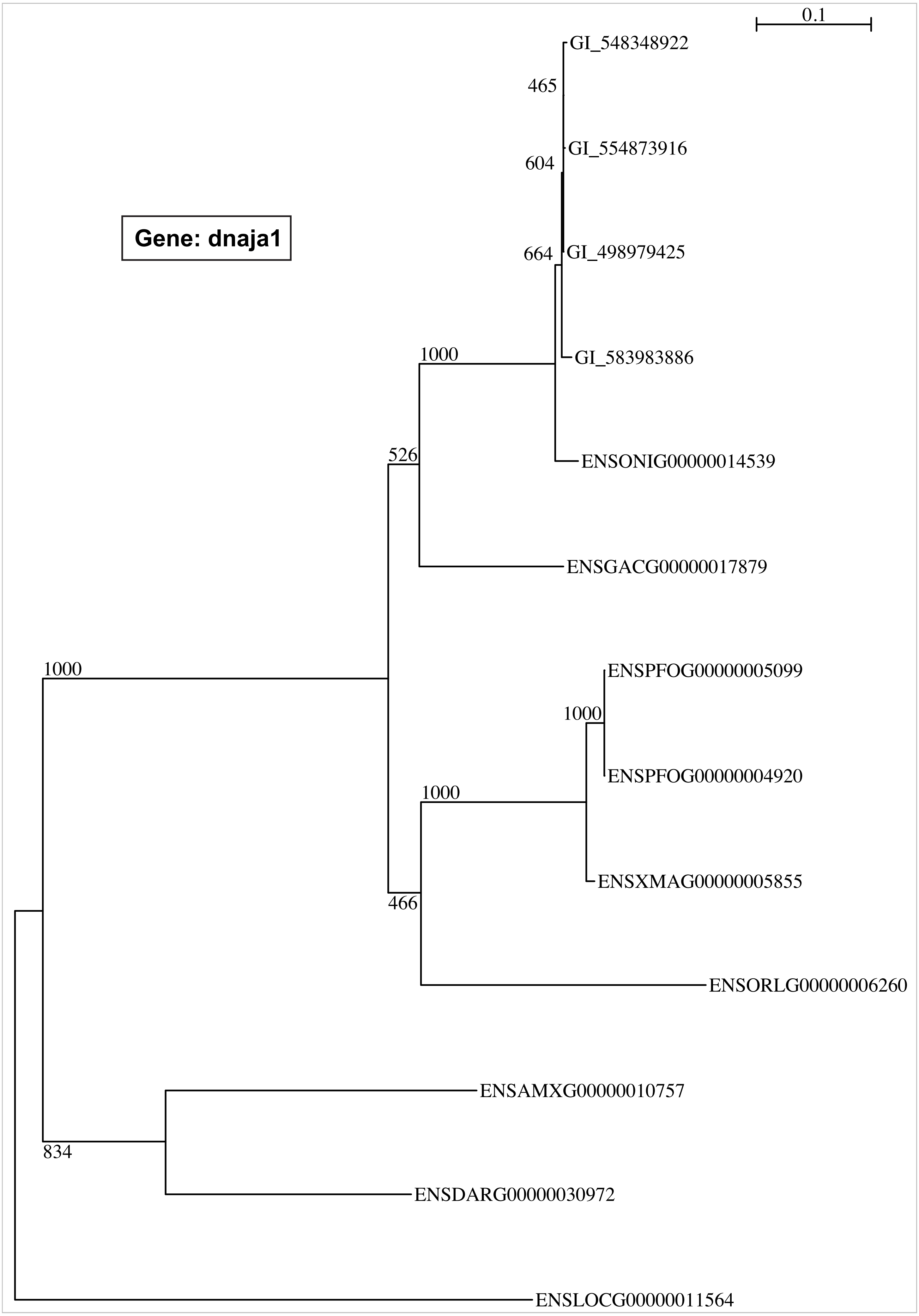
0.1
GI_548348922
465
GI_554873916
604
Gene: dnaja1
664
GI_498979425
1000
GI_583983886
526
ENSONIG00000014539
ENSGACG00000017879
1000
ENSPFOG00000005099
1000
ENSPFOG00000004920
1000
ENSXMAG00000005855
466
ENSORLG00000006260
ENSAMXG00000010757
834
ENSDARG00000030972
ENSLOCG00000011564

Figure S1

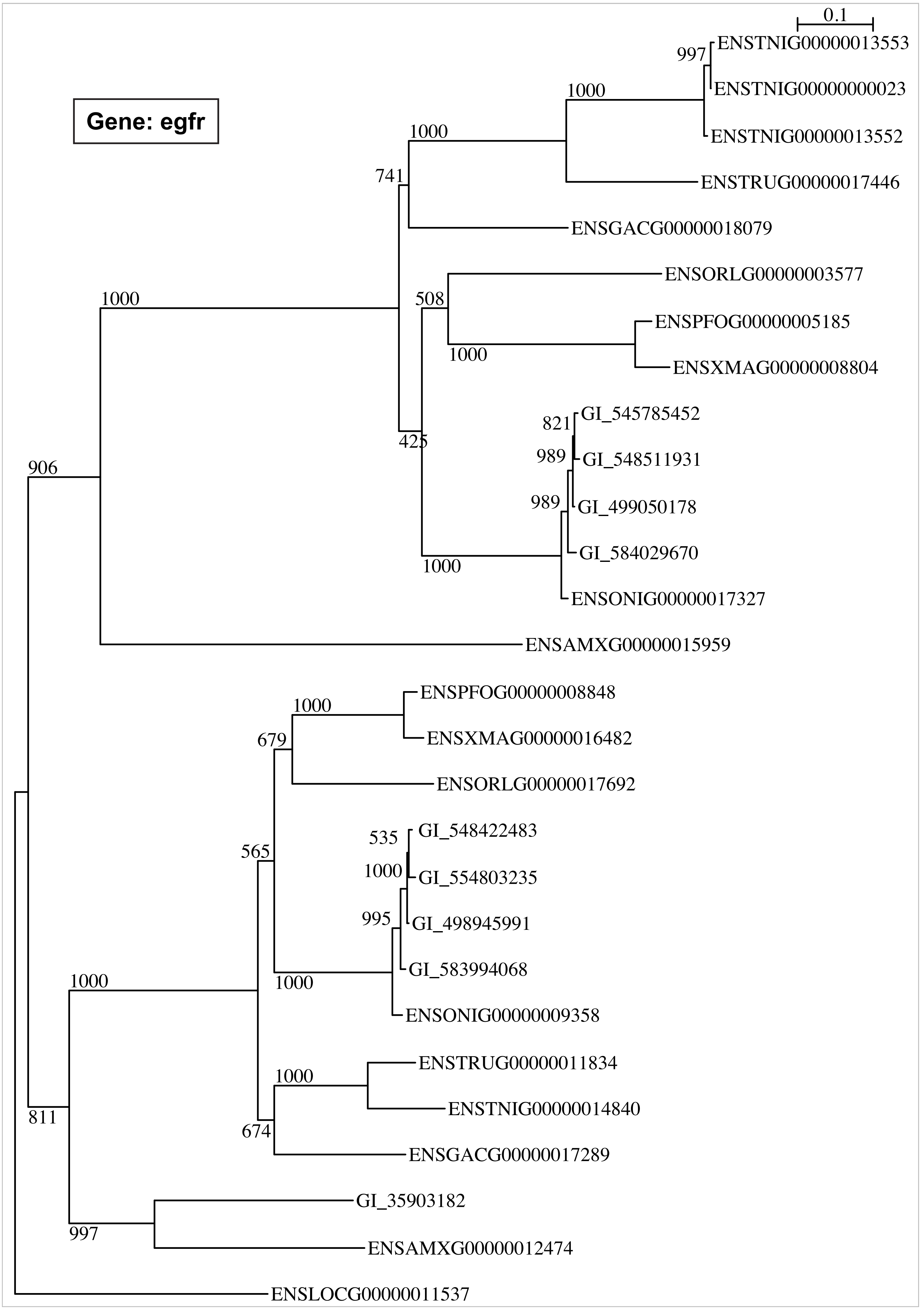
Gene: egfr
0.1
ENSTNIG00000013553
ENSTNIG00000000023
ENSTNIG00000013552
ENSTRUG00000017446
ENSGACG00000018079
ENSORLG00000003577
ENSPFOG00000005185
ENSXMAG00000008804
GI_545785452
GI_548511931
GI_499050178
GI_584029670
ENSONIG00000017327
ENSAMXG00000015959
ENSPFOG00000008848
ENSXMAG00000016482
ENSORLG00000017692
GI_548422483
GI_554803235
GI_498945991
GI_583994068
ENSONIG00000009358
ENSTRUG00000011834
ENSTNIG00000014840
ENSGACG00000017289
GI_35903182
ENSAMXG00000012474
ENSLOCG00000011537
997
1000
1000
741
1000
508
1000
425
821
989
989
1000
906
1000
679
535
1000
565
995
1000
1000
1000
811
674
997

Figure S1

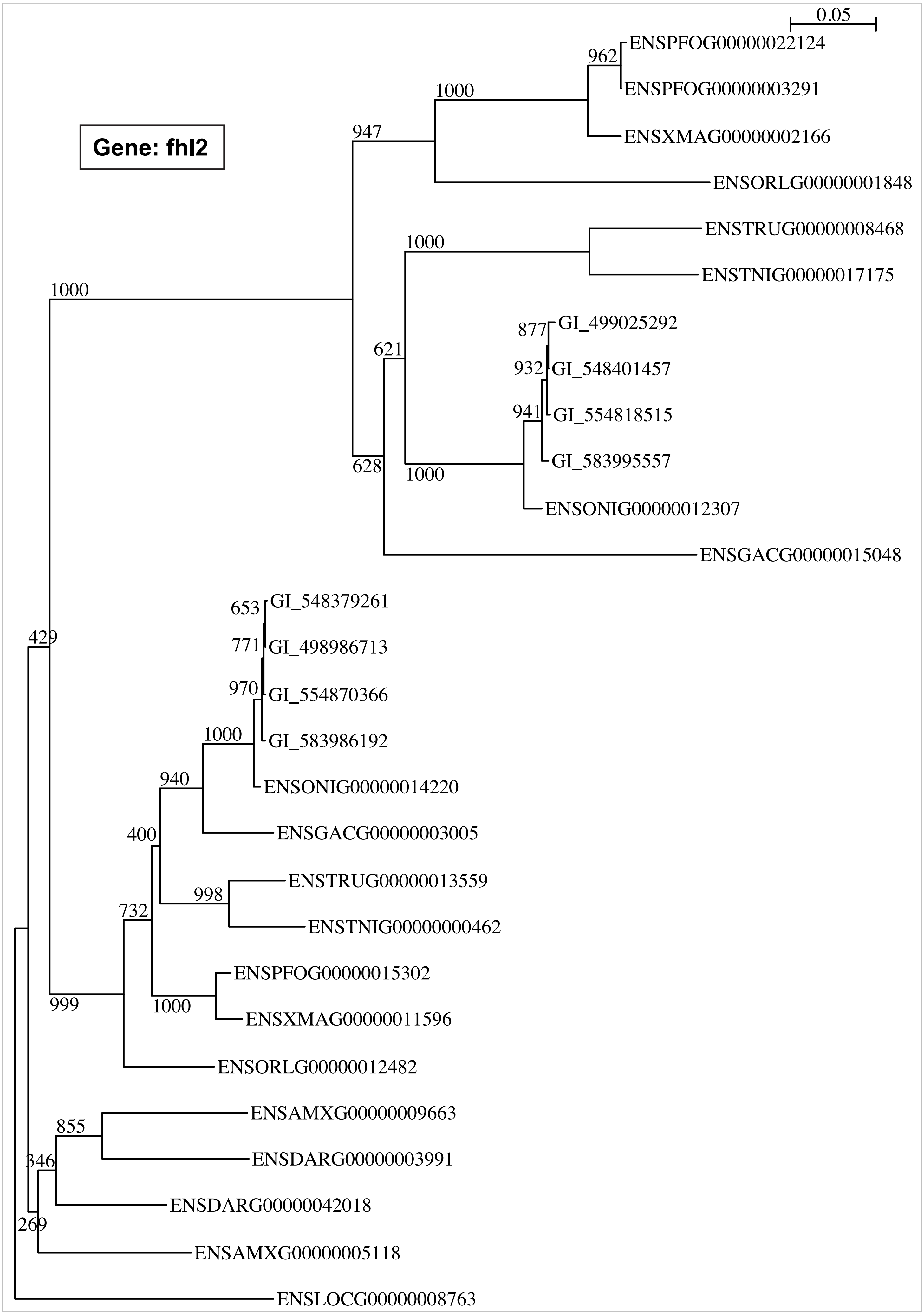
0.05
Gene: fhl2
962
1000
947
1000
1000
621
877
932
941
628
1000
ENSPFOG00000022124
ENSPFOG00000003291
ENSXMAG00000002166
ENSORLG00000001848
ENSTRUG00000008468
ENSTNIG00000017175
GI_499025292
GI_548401457
GI_554818515
GI_583995557
ENSONIG00000012307
ENSGACG00000015048
429
653
771
970
1000
940
400
998
732
999
1000
855
346
269
GI_548379261
GI_498986713
GI_554870366
GI_583986192
ENSONIG00000014220
ENSGACG00000003005
ENSTRUG00000013559
ENSTNIG00000000462
ENSPFOG00000015302
ENSXMAG00000011596
ENSORLG00000012482
ENSAMXG00000009663
ENSDARG00000003991
ENSDARG00000042018
ENSAMXG00000005118
ENSLOCG00000008763

Figure S1

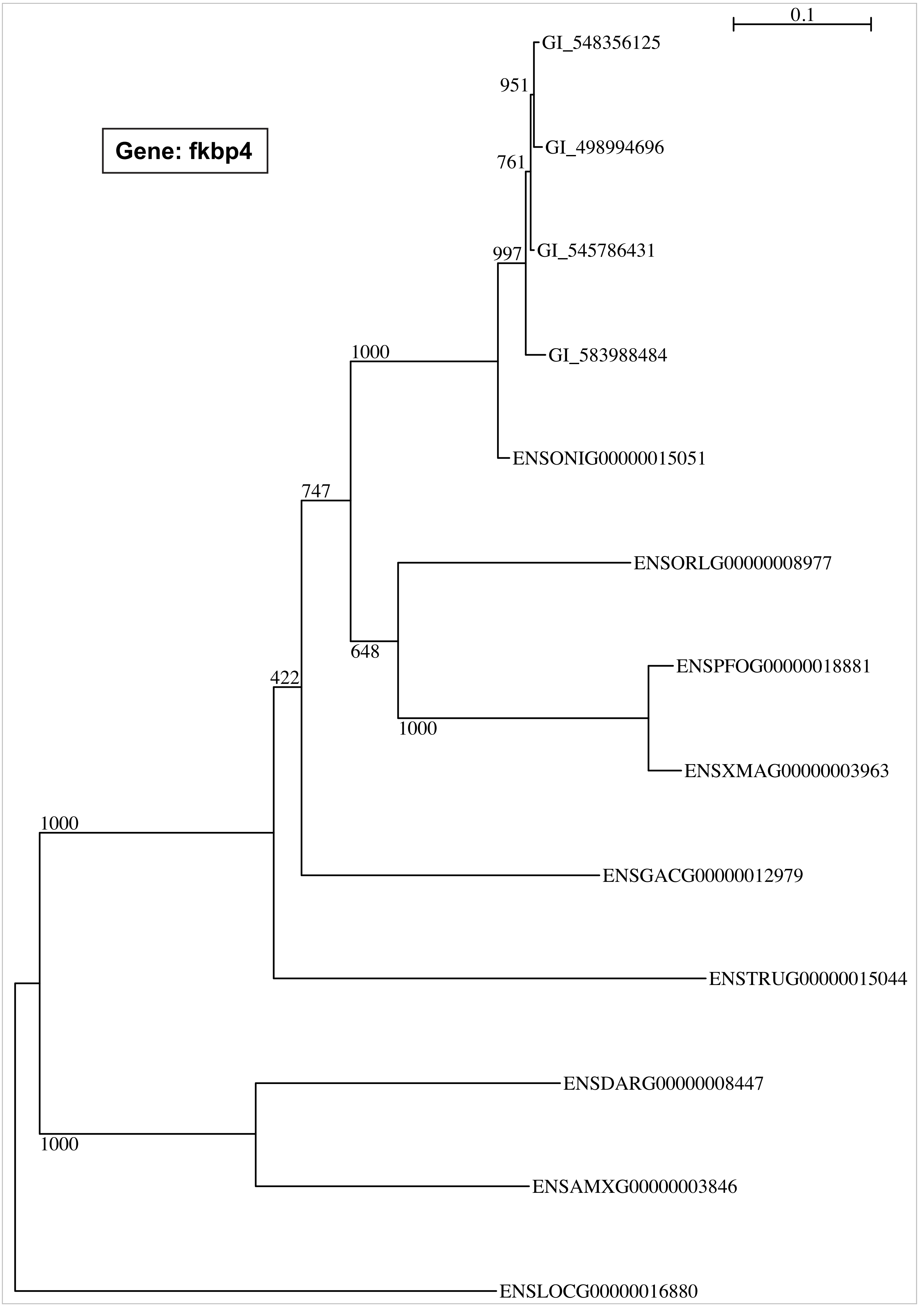
0.1
Gene: fkbp4
GI_548356125
951
GI_498994696
761
GI_545786431
997
GI_583988484
1000
ENSONIG00000015051
747
ENSORLG00000008977
648
422
ENSPFOG00000018881
1000
ENSXMAG00000003963
1000
ENSGACG00000012979
ENSTRUG00000015044
ENSDARG00000008447
1000
ENSAMXG00000003846
ENSLOCG00000016880

Figure S1

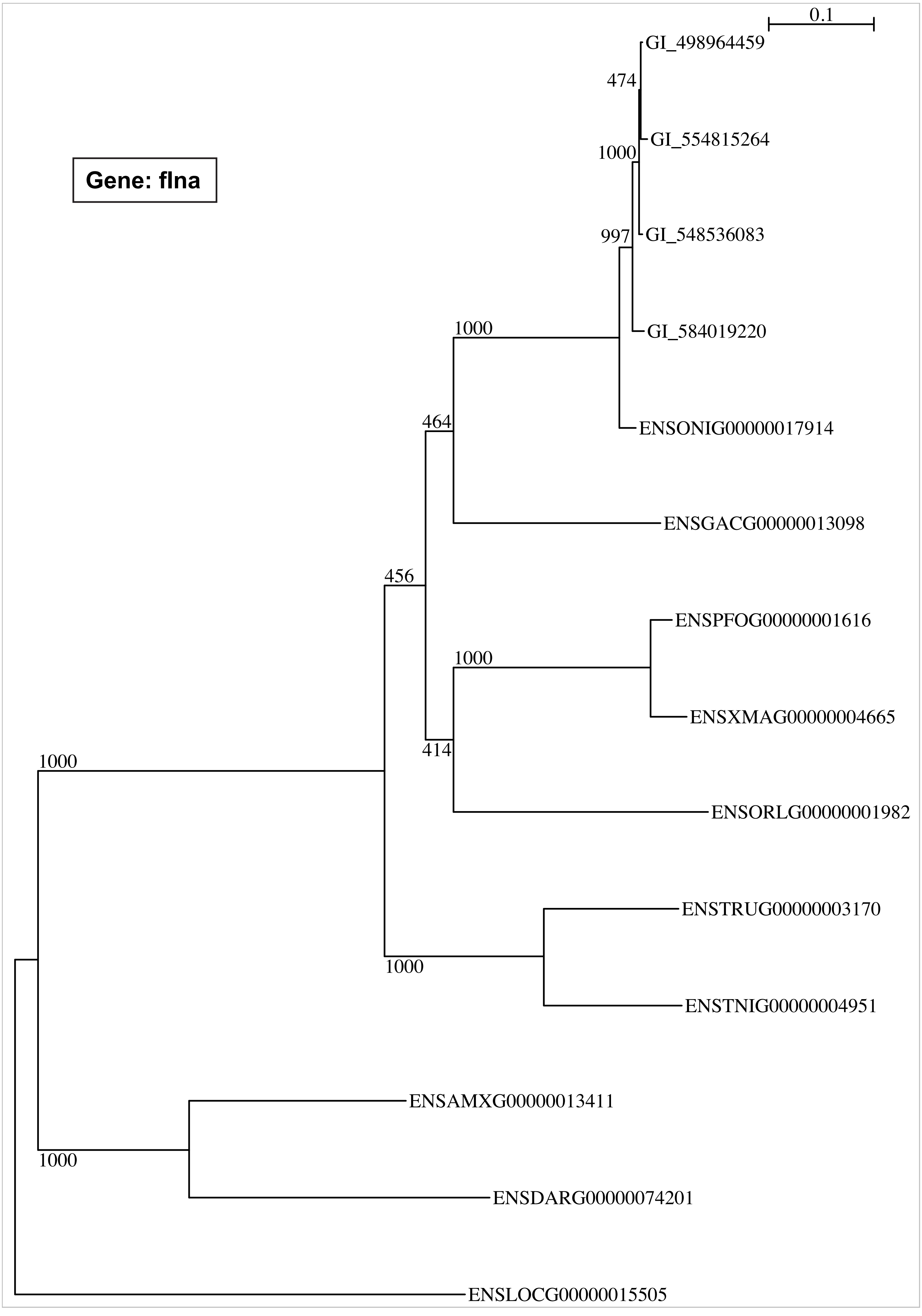
0.1
Gene: flna
GI_498964459
474
GI_554815264
1000
GI_548536083
997
GI_584019220
1000
ENSONIG00000017914
464
ENSGACG00000013098
456
ENSPFOG00000001616
1000
ENSXMAG00000004665
414
ENSORLG00000001982
1000
ENSTRUG00000003170
1000
ENSTNIG00000004951
ENSAMXG00000013411
1000
ENSDARG00000074201
ENSLOCG00000015505

Figure S1

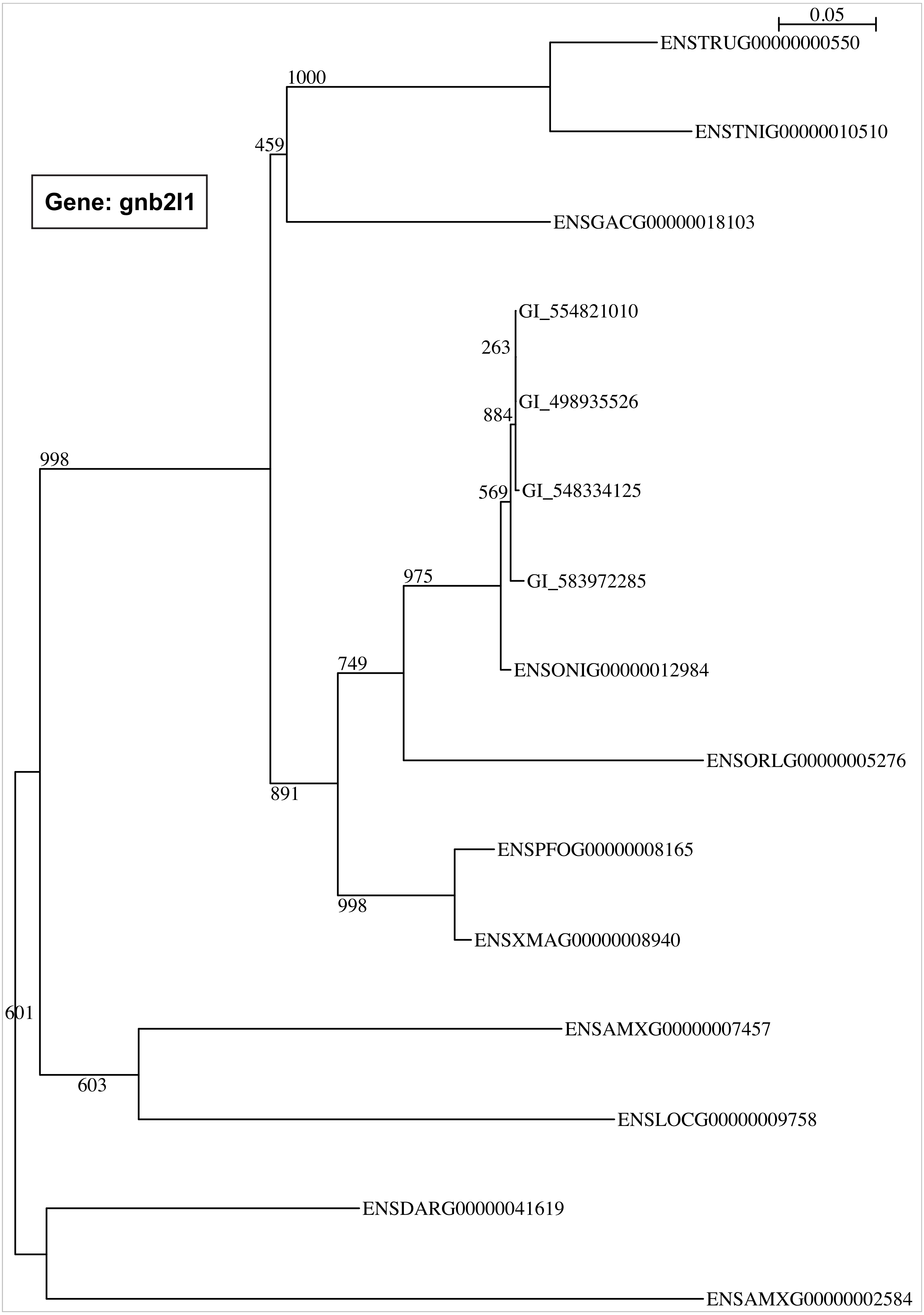

0.05
Gene: gnb2l1
1000
459
998
263
884
569
975
749
891
998
601
603
ENSTRUG00000000550
ENSTNIG00000010510
ENSGACG00000018103
GI_554821010
GI_498935526
GI_548334125
GI_583972285
ENSONIG00000012984
ENSORLG00000005276
ENSPFOG00000008165
ENSXMAG00000008940
ENSAMXG00000007457
ENSLOCG00000009758
ENSDARG00000041619
ENSAMXG00000002584

Figure S1

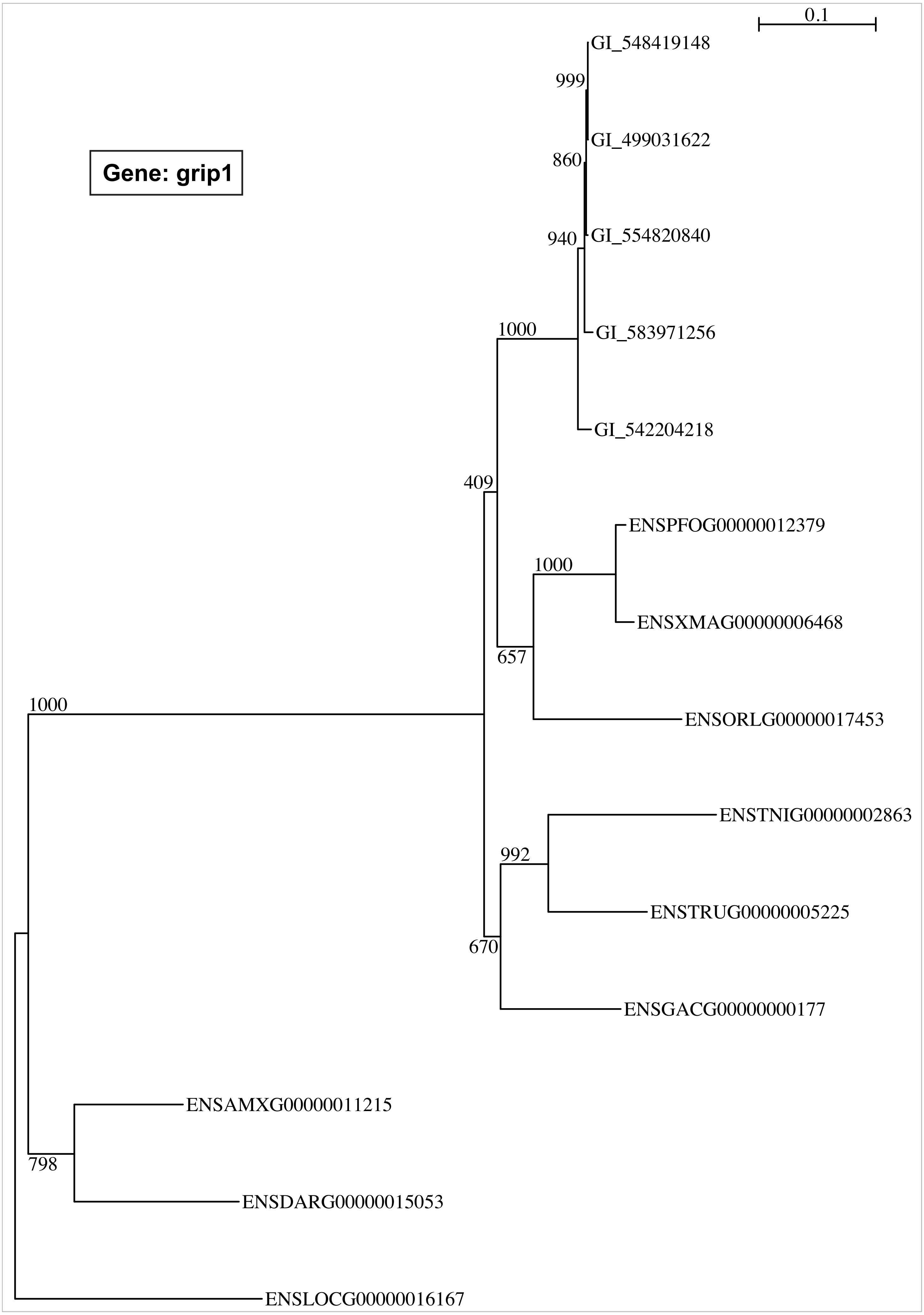
0.1
Gene: grip1
GI_548419148
999
GI_499031622
860
GI_554820840
940
GI_583971256
1000
GI_542204218
409
ENSPFOG00000012379
1000
ENSXMAG00000006468
657
ENSORLG00000017453
1000
ENSTNIG00000002863
992
ENSTRUG00000005225
670
ENSGACG00000000177
ENSAMXG00000011215
798
ENSDARG00000015053
ENSLOCG00000016167

Figure S1

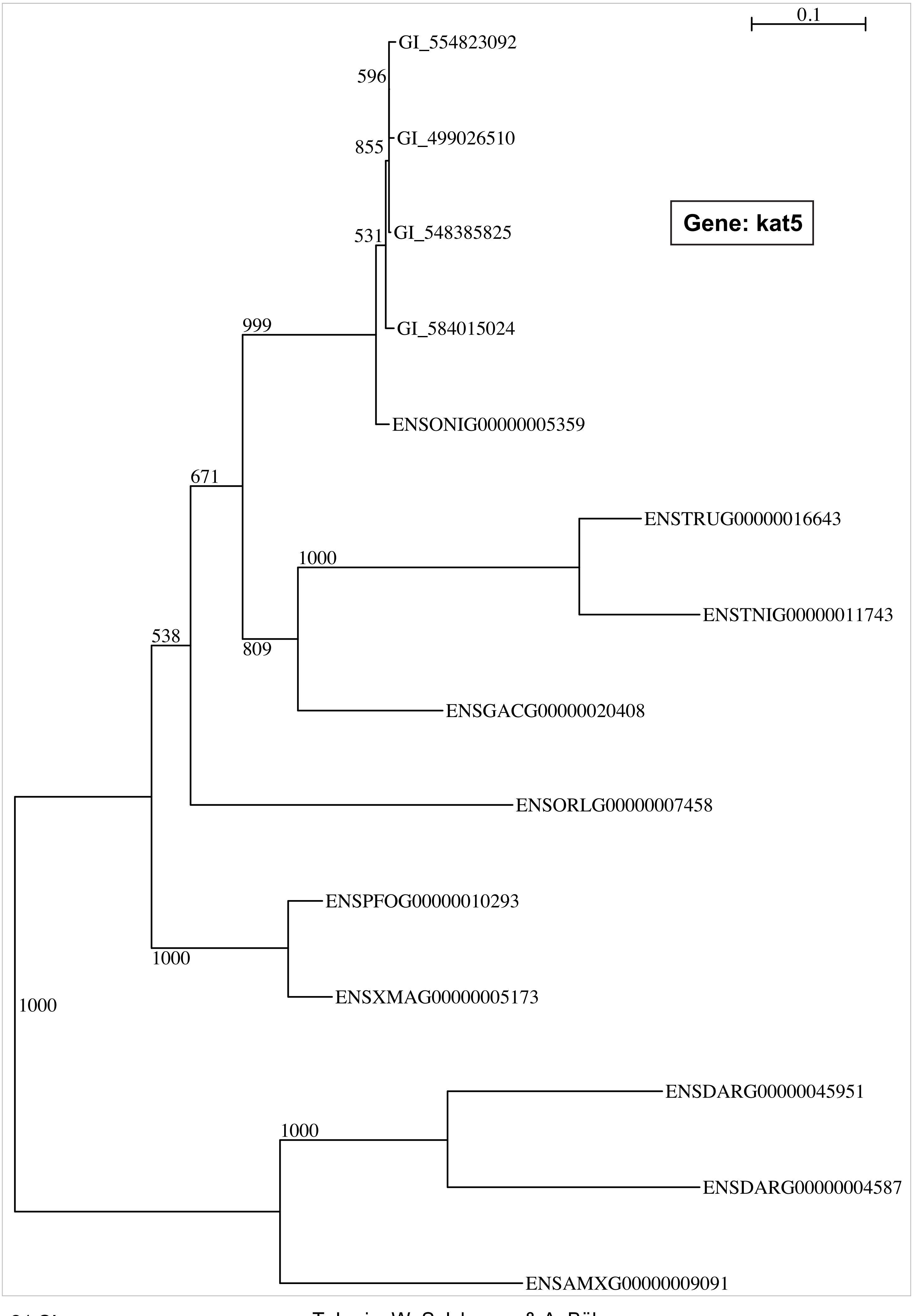

 T. Lorin, W. Salzburger & A. Böhne

Figure S1

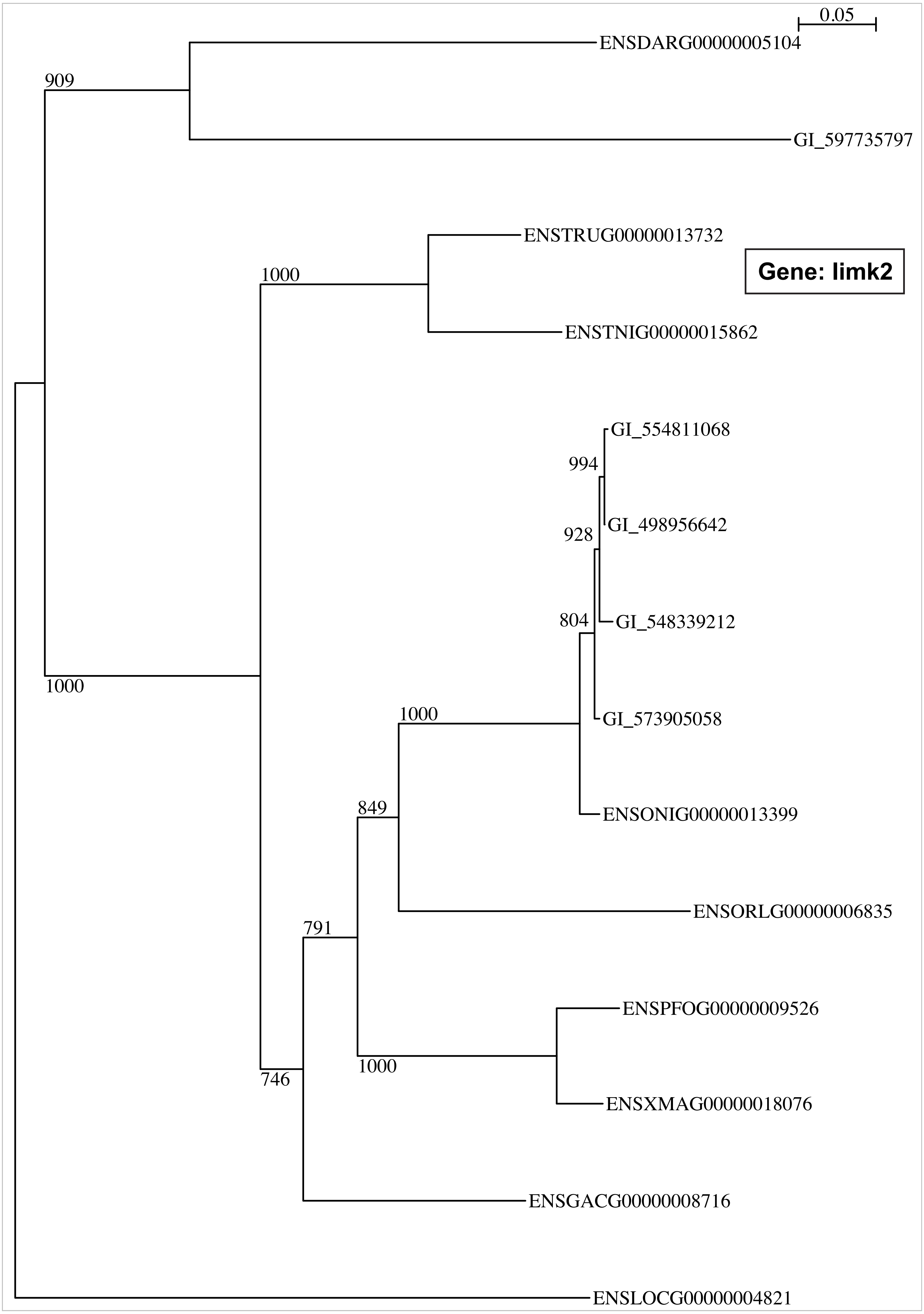
0.05
ENSDARG00000005104
909
GI_597735797
ENSTRUG00000013732
1000
Gene: limk2
ENSTNIG00000015862
GI_554811068
994
GI_498956642
928
GI_548339212
804
1000
GI_573905058
1000
ENSONIG00000013399
849
ENSORLG00000006835
791
ENSPFOG00000009526
1000
746
ENSXMAG00000018076
ENSGACG00000008716
ENSLOCG00000004821

Figure S1

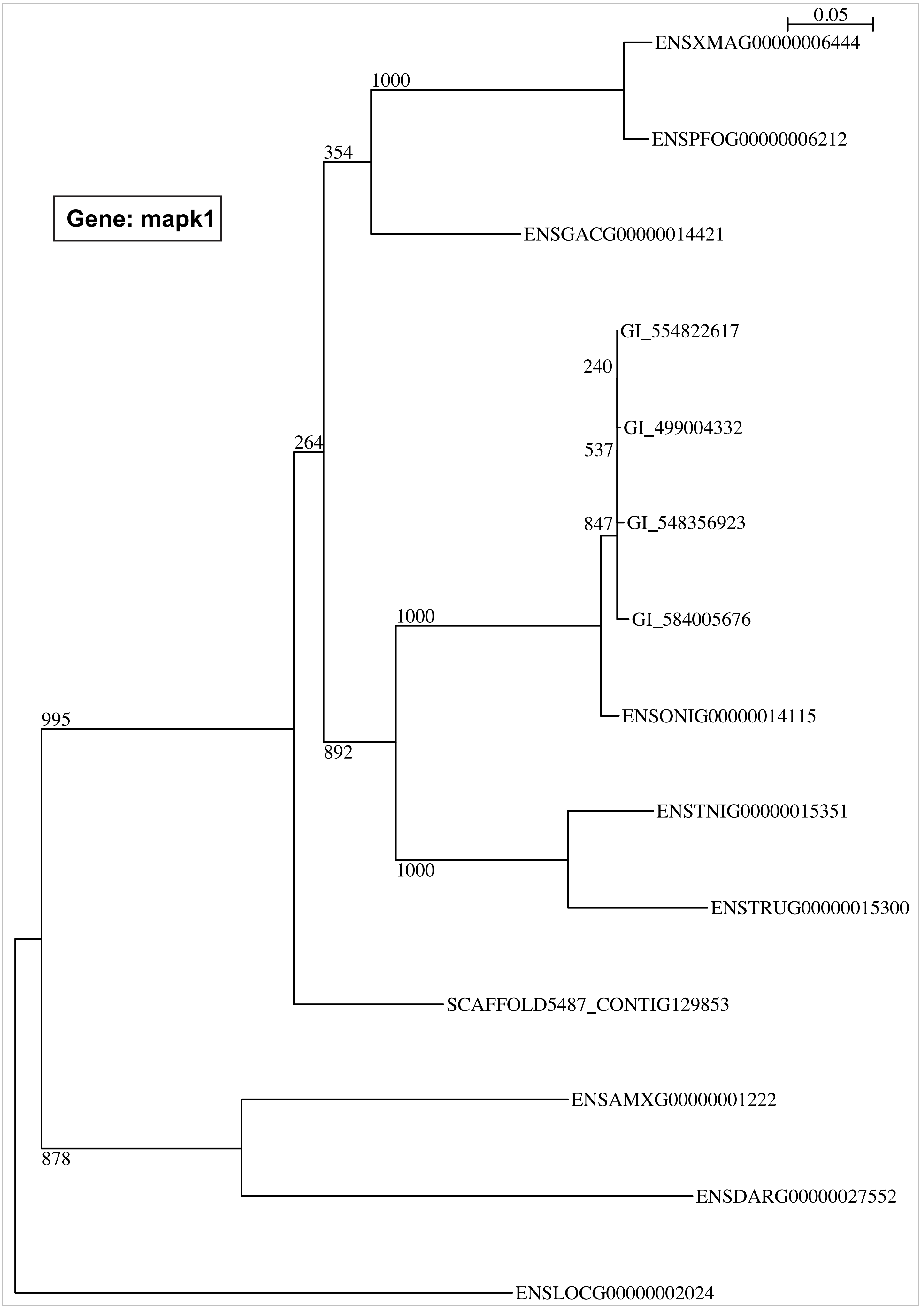
0.05
ENSXMAG00000006444
1000
ENSPFOG00000006212
354
Gene: mapk1
ENSGACG00000014421
GI_554822617
240
GI_499004332
264
537
847
GI_548356923
1000
GI_584005676
995
ENSONIG00000014115
892
ENSTNIG00000015351
1000
ENSTRUG00000015300
SCAFFOLD5487_CONTIG129853
ENSAMXG00000001222
878
ENSDARG00000027552
ENSLOCG00000002024

Figure S1

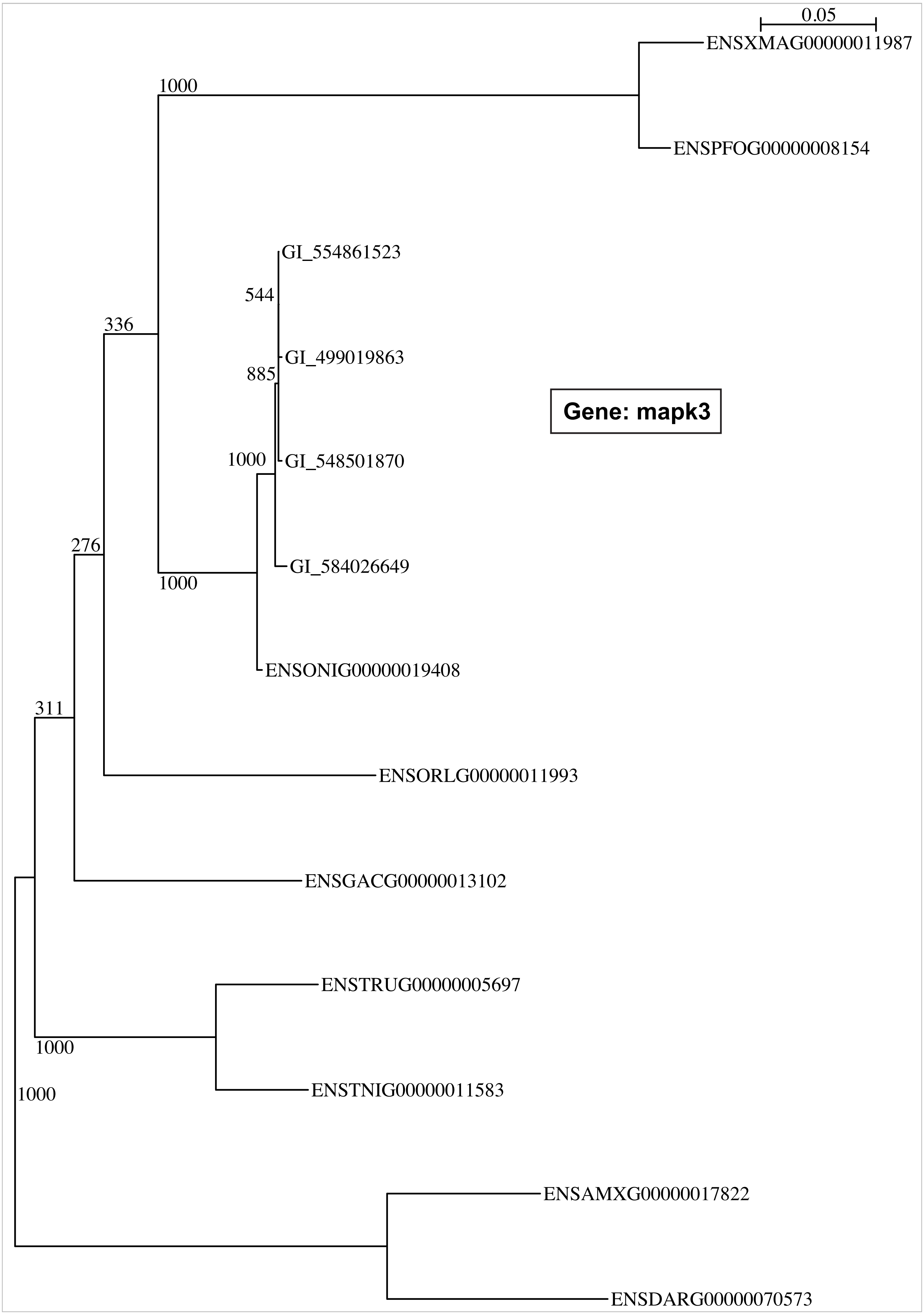
0.05
ENSXMAG00000011987
1000
ENSPFOG00000008154
GI_554861523
544
336
GI_499019863
885
Gene: mapk3
1000
GI_548501870
276
GI_584026649
1000
ENSONIG00000019408
311
ENSORLG00000011993
ENSGACG00000013102
ENSTRUG00000005697
1000
1000
ENSTNIG00000011583
ENSAMXG00000017822
ENSDARG00000070573

Figure S1

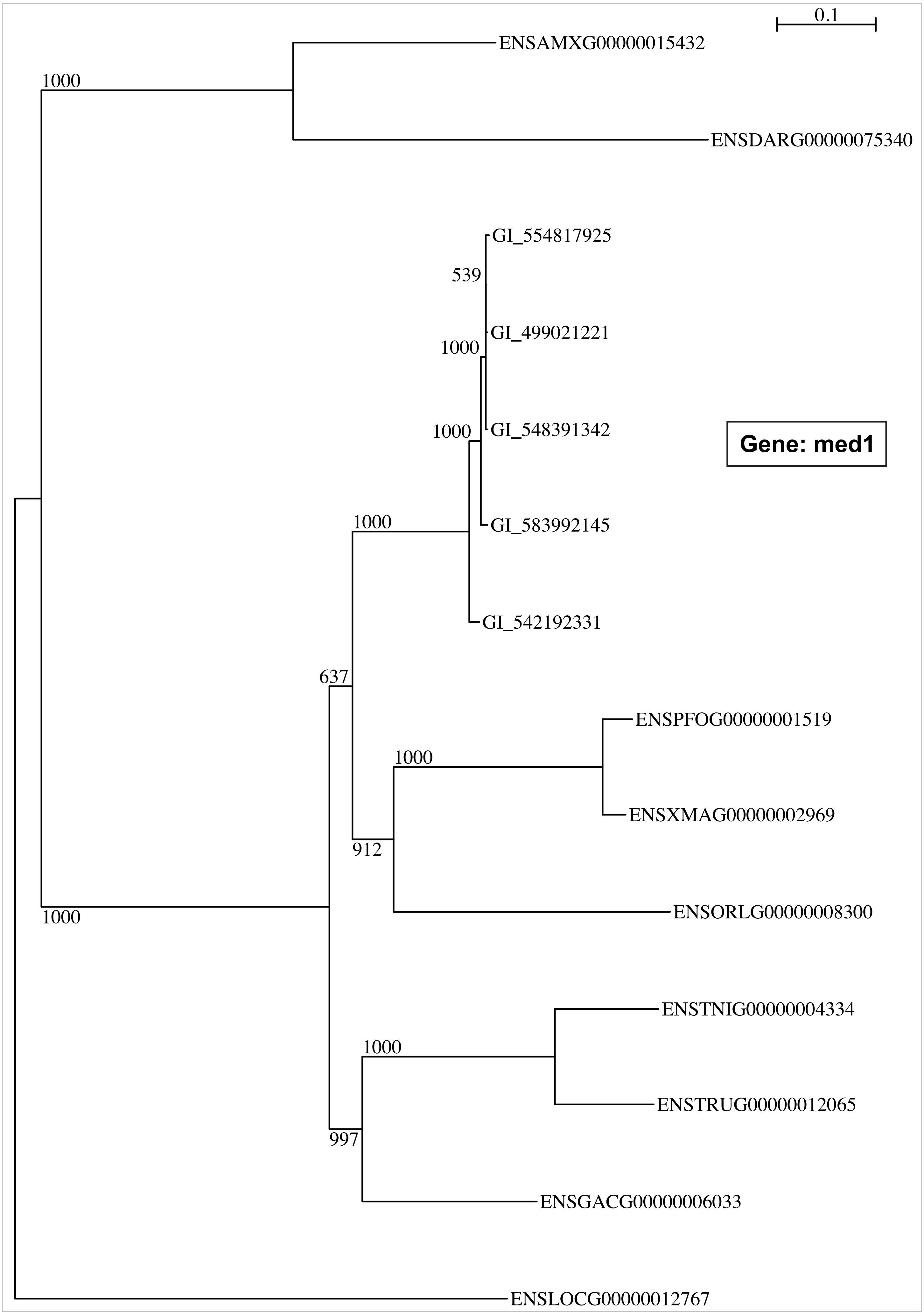
0.1
ENSAMXG00000015432
1000
ENSDARG00000075340
GI_554817925
539
GI_499021221
1000
GI_548391342
1000
Gene: med1
1000
GI_583992145
GI_542192331
637
ENSPFOG00000001519
1000
ENSXMAG00000002969
912
ENSORLG00000008300
1000
ENSTNIG00000004334
1000
ENSTRUG00000012065
997
ENSGACG00000006033
ENSLOCG00000012767

Figure S1

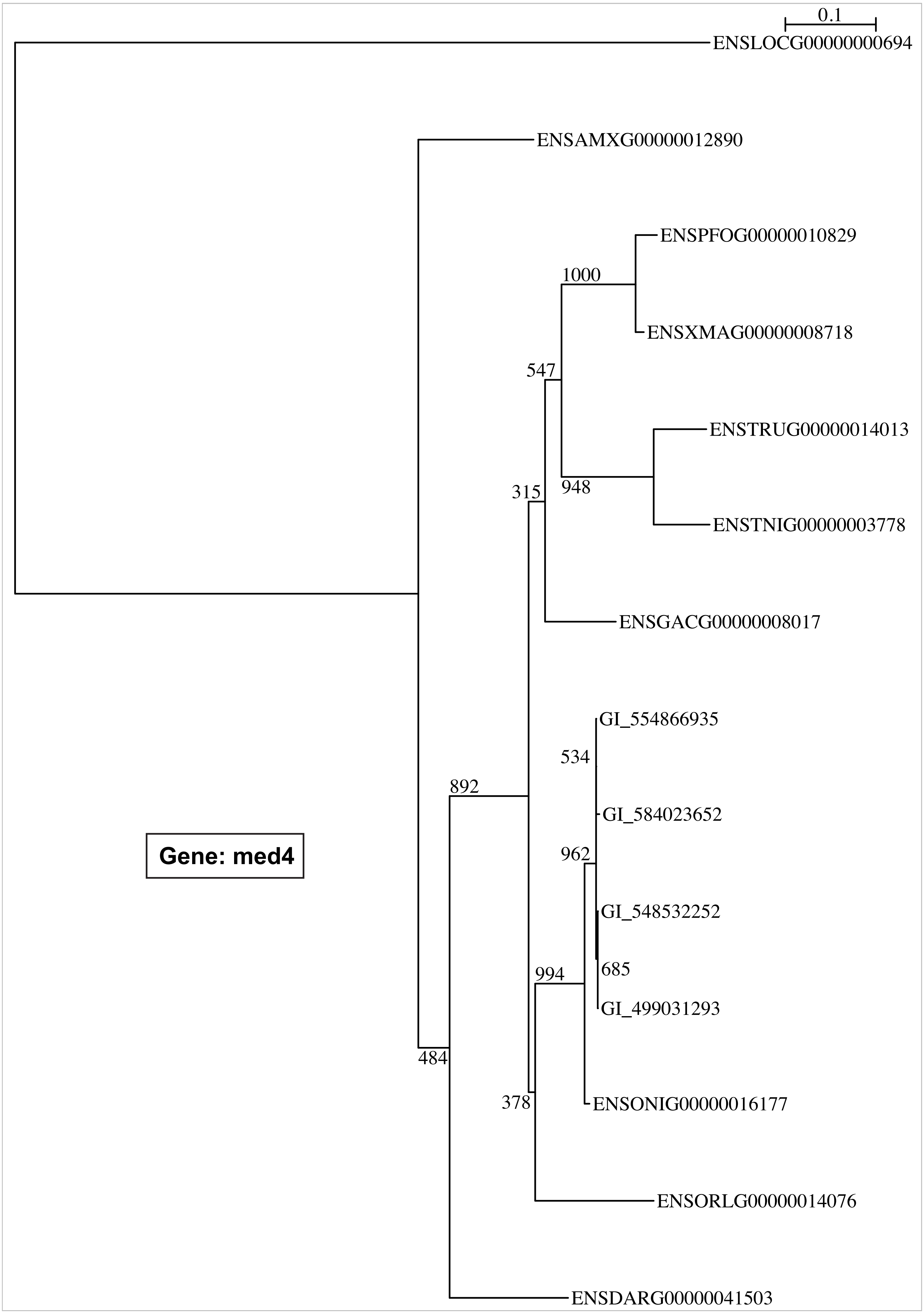
0.1
ENSLOCG00000000694
ENSAMXG00000012890
ENSPFOG00000010829
1000
ENSXMAG00000008718
547
ENSTRUG00000014013
315
948
ENSTNIG00000003778
ENSGACG00000008017
GI_554866935
534
892
GI_584023652
Gene: med4
962
GI_548532252
994
685
GI_499031293
484
378
ENSONIG00000016177
ENSORLG00000014076
ENSDARG00000041503

Figure S1

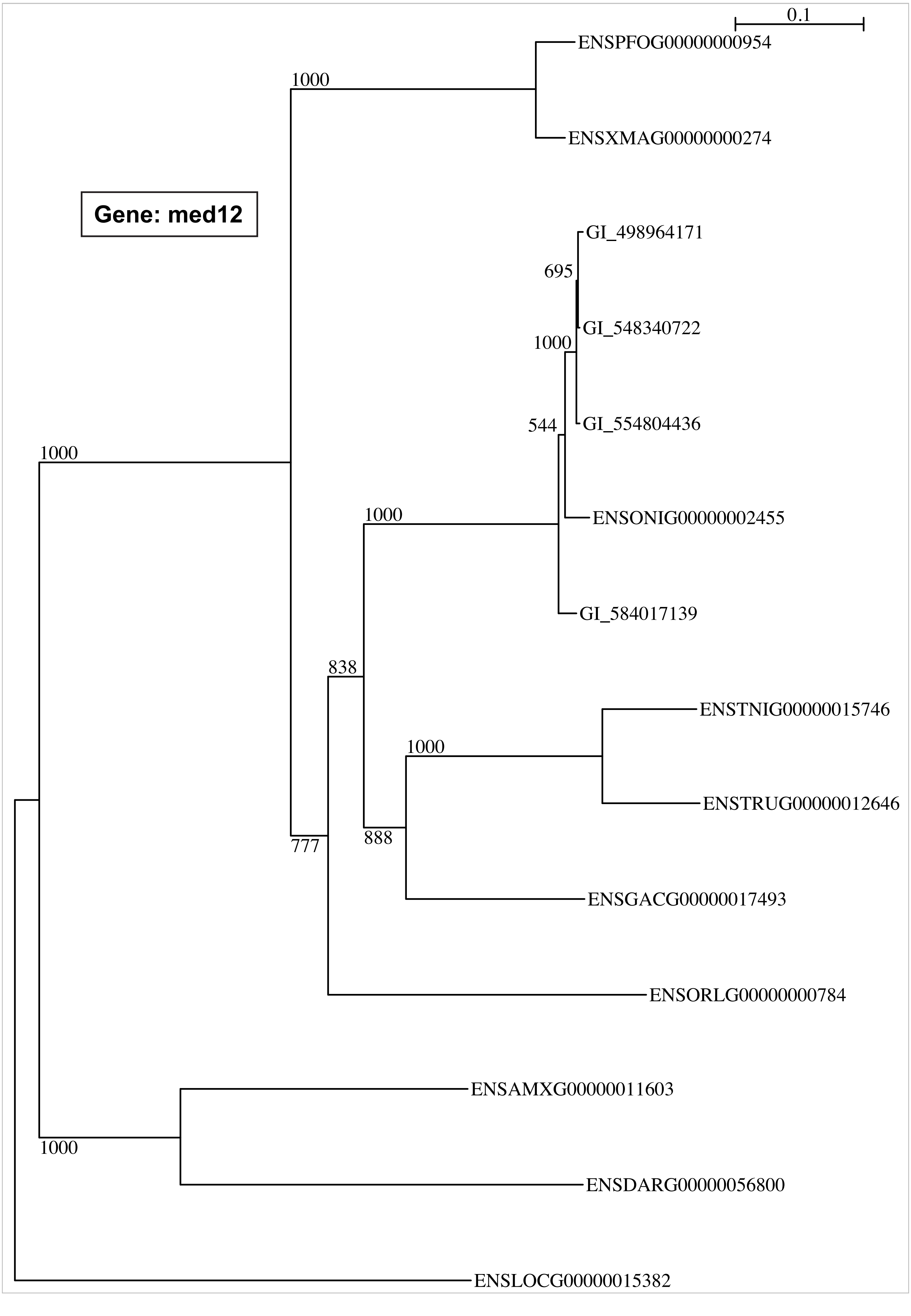

 T. Lorin, W. Salzburger & A. Böhne

Figure S1

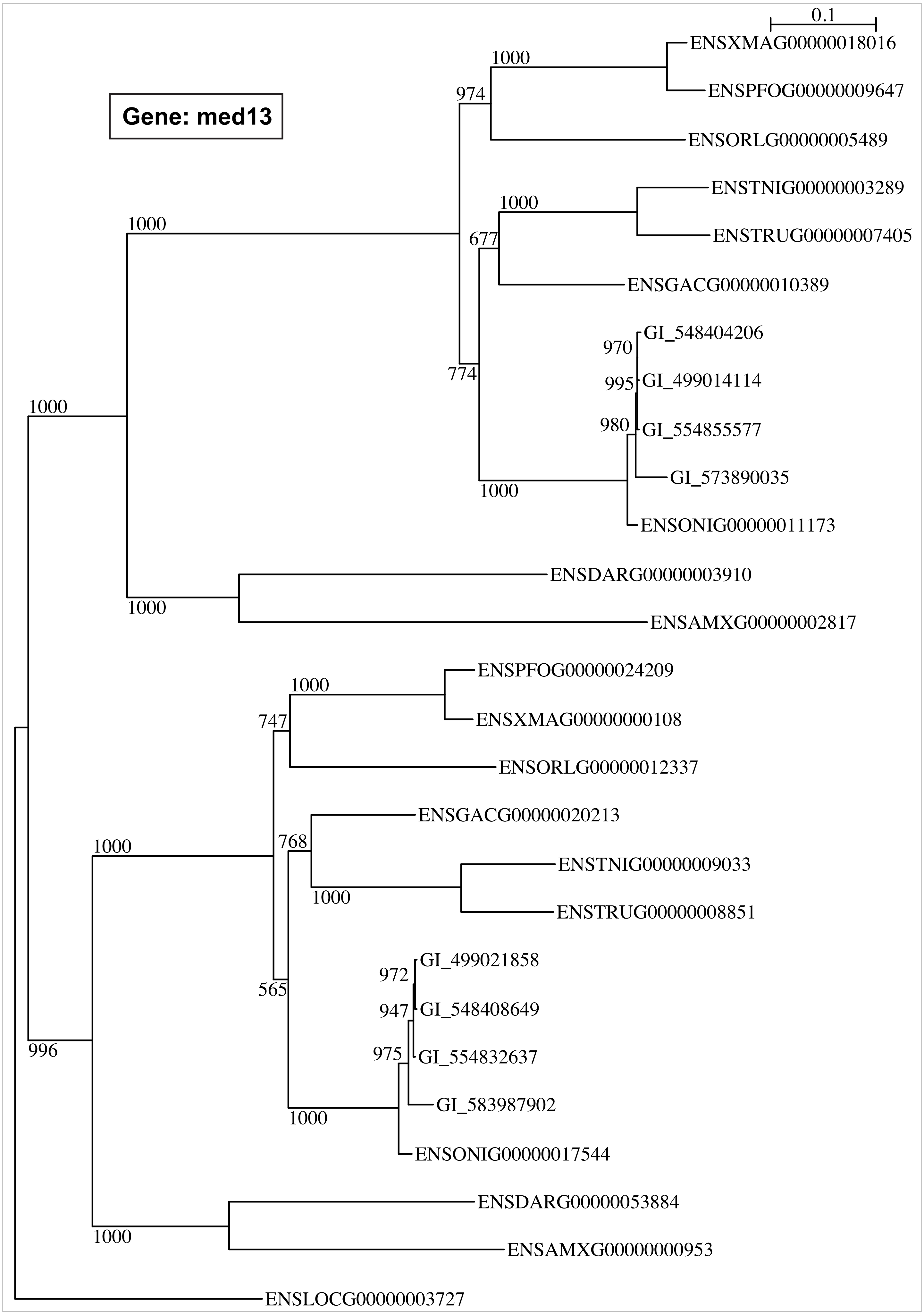
Gene: med13
0.1
1000
974
ENSXMAG00000018016
ENSPFOG00000009647
ENSORLG00000005489
1000
677
ENSTNIG00000003289
ENSTRUG00000007405
ENSGACG00000010389
1000
774
970
995
980
GI_548404206
GI_499014114
GI_554855577
GI_573890035
1000
ENSONIG00000011173
1000
1000
ENSDARG00000003910
ENSAMXG00000002817
1000
747
ENSPFOG00000024209
ENSXMAG00000000108
ENSORLG00000012337
768
ENSGACG00000020213
1000
ENSTNIG00000009033
ENSTRUG00000008851
565
972
947
975
GI_499021858
GI_548408649
GI_554832637
GI_583987902
1000
ENSONIG00000017544
996
1000
ENSDARG00000053884
ENSAMXG00000000953
ENSLOCG00000003727

Figure S1

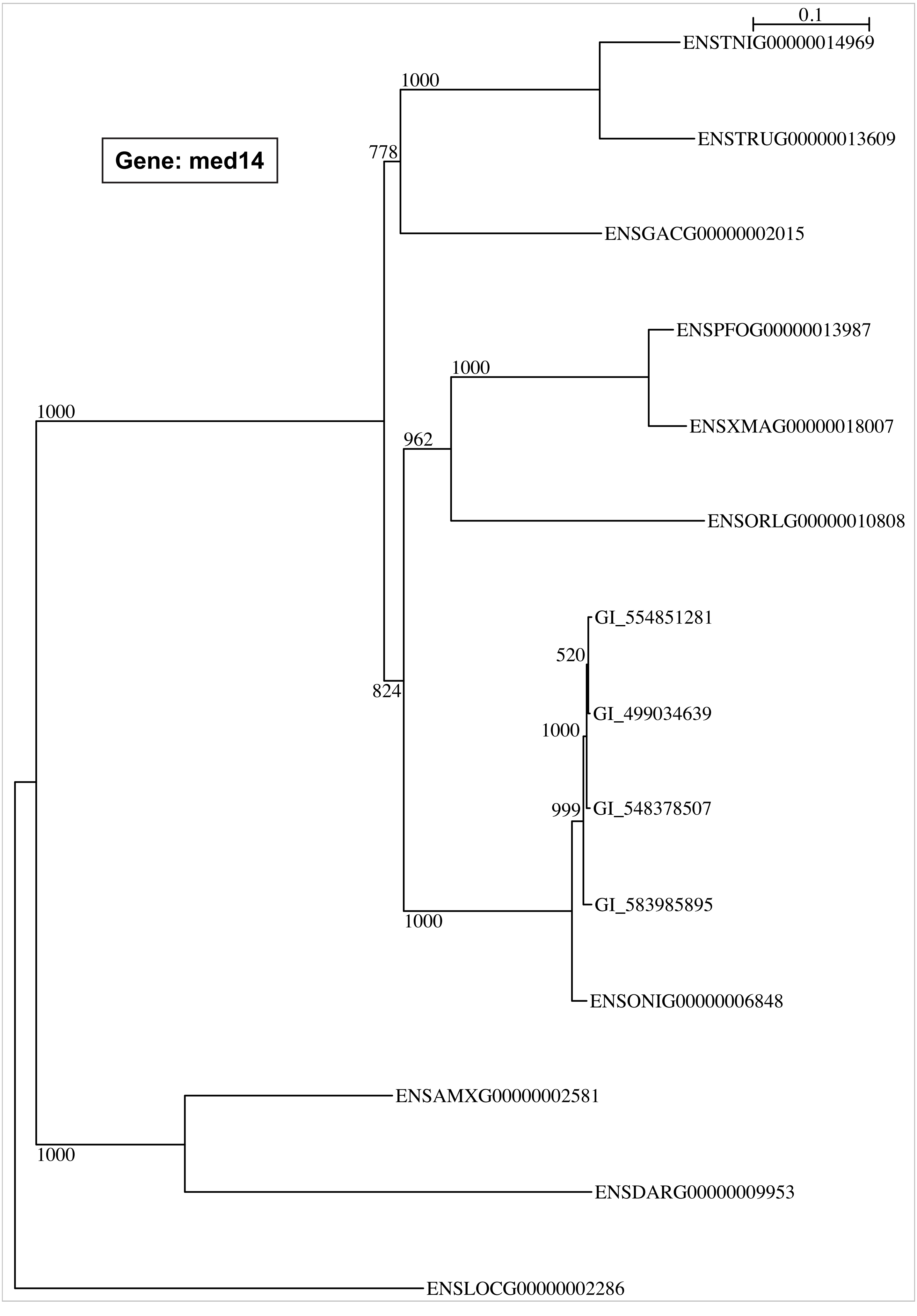
Gene: med14
0.1
ENSTNIG00000014969
ENSTRUG00000013609
ENSGACG00000002015
ENSPFOG00000013987
ENSXMAG00000018007
ENSORLG00000010808
GI_554851281
GI_499034639
GI_548378507
GI_583985895
ENSONIG00000006848
ENSAMXG00000002581
ENSDARG00000009953
ENSLOCG00000002286
1000
778
1000
962
1000
520
1000
824
999
1000
1000

Figure S1

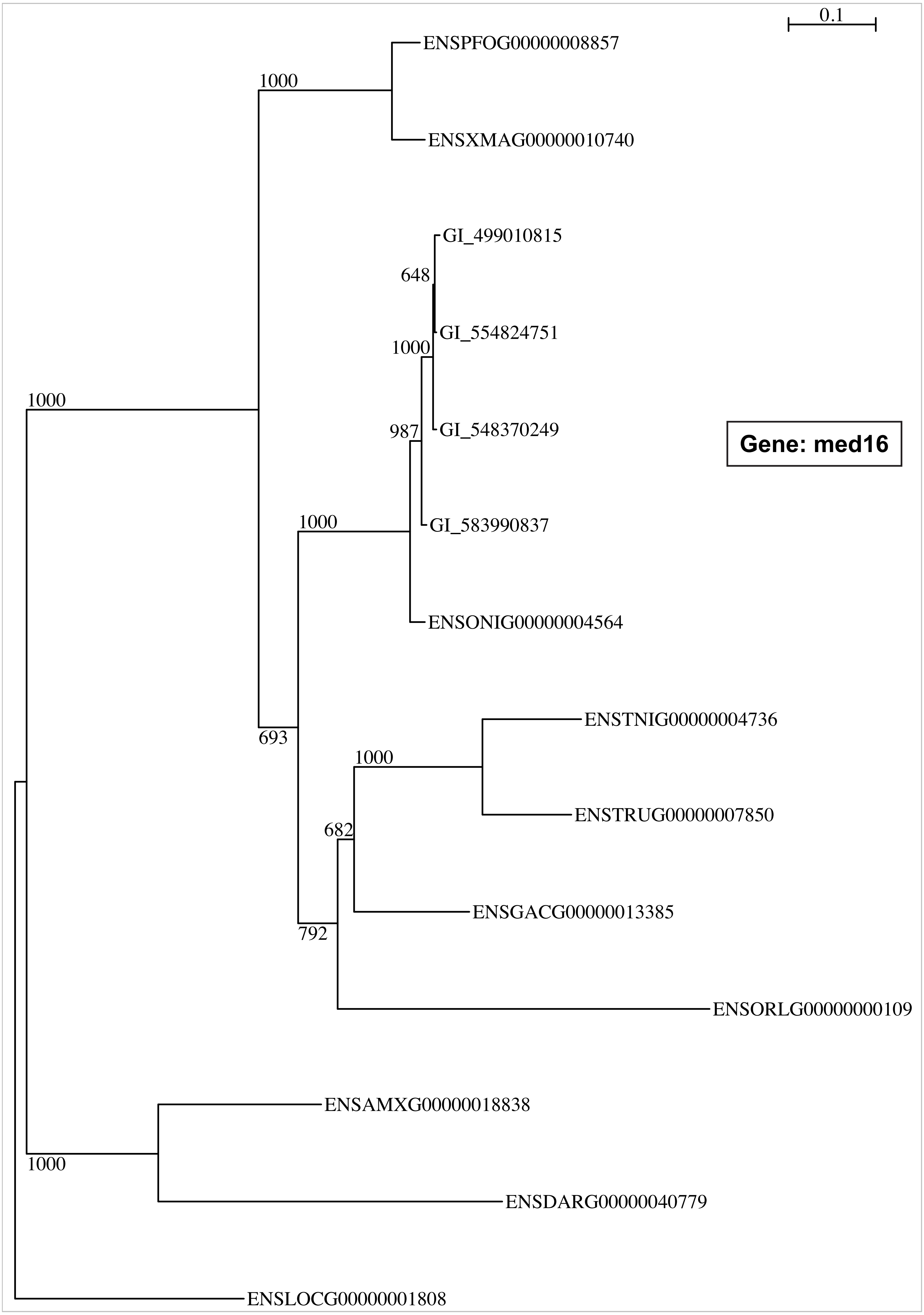
0.1
1000
ENSPFOG00000008857
ENSXMAG00000010740
GI_499010815
648
GI_554824751
1000
1000
GI_548370249
987
Gene: med16
1000
GI_583990837
ENSONIG00000004564
ENSTNIG00000004736
693
1000
ENSTRUG00000007850
682
ENSGACG00000013385
792
ENSORLG00000000109
ENSAMXG00000018838
1000
ENSDARG00000040779
ENSLOCG00000001808

Figure S1

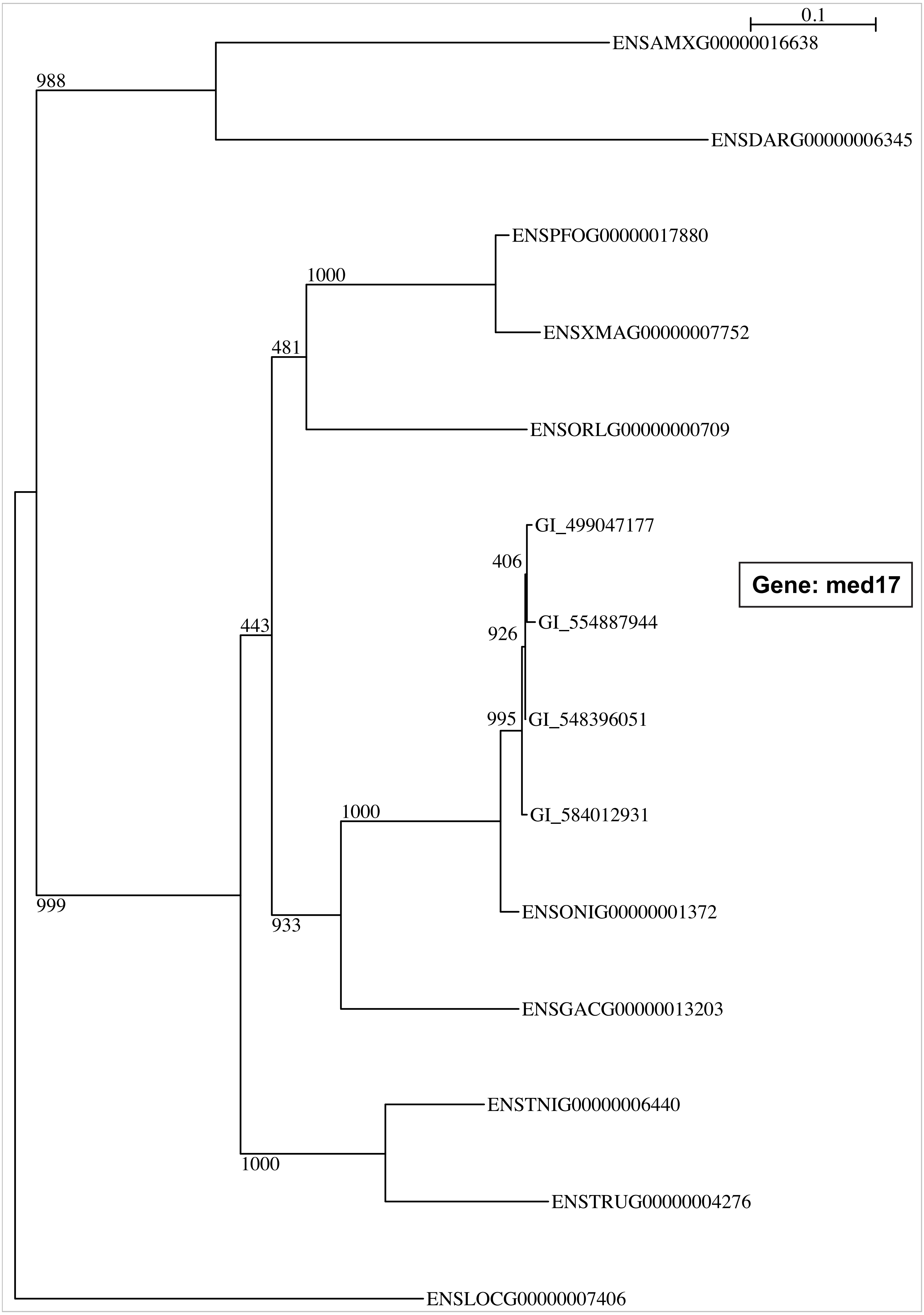
0.1
ENSAMXG00000016638
988
ENSDARG00000006345
ENSPFOG00000017880
1000
ENSXMAG00000007752
481
ENSORLG00000000709
GI_499047177
406
Gene: med17
443
GI_554887944
926
995
GI_548396051
1000
GI_584012931
999
933
ENSONIG00000001372
ENSGACG00000013203
ENSTNIG00000006440
1000
ENSTRUG00000004276
ENSLOCG00000007406

Figure S1

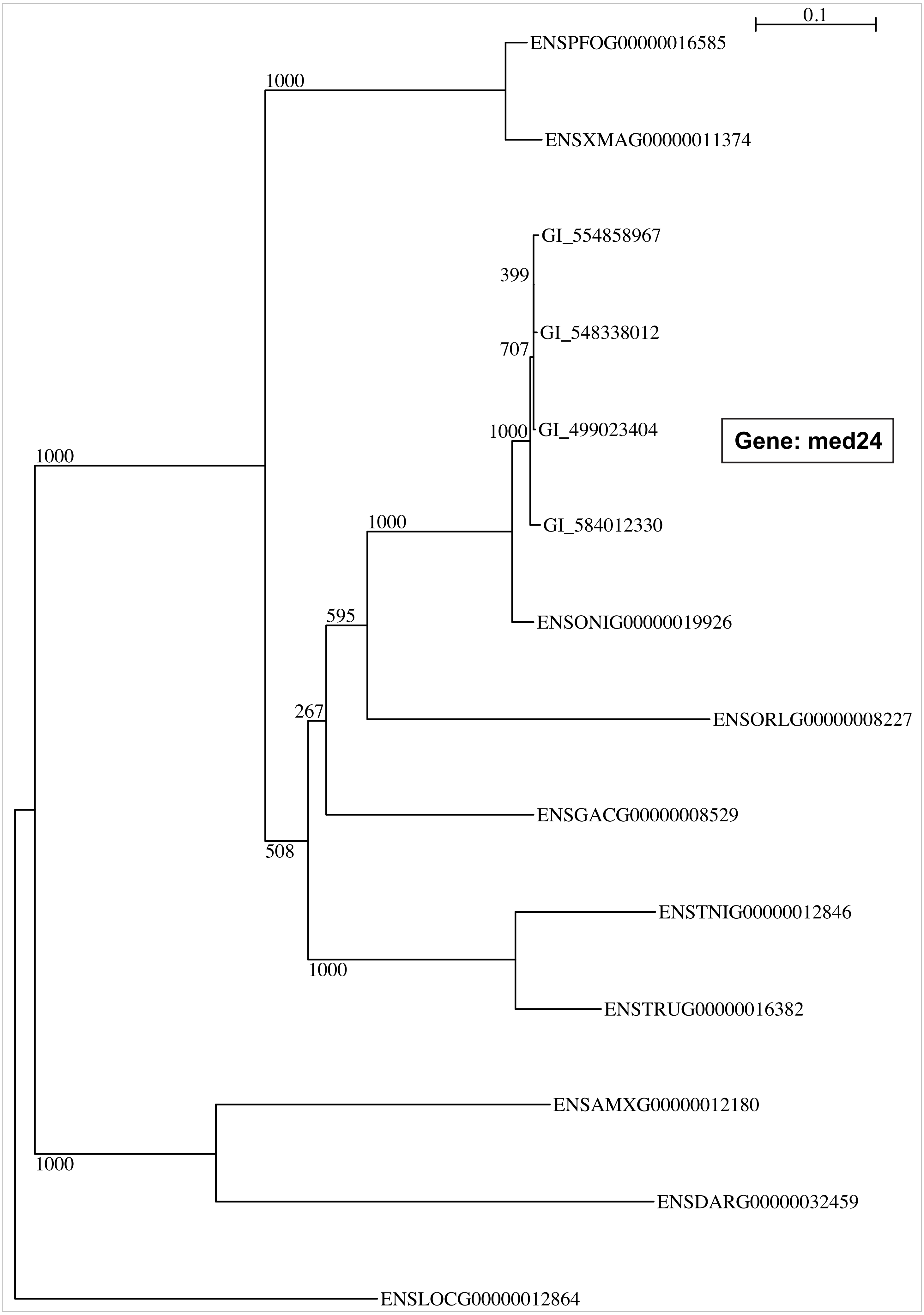
0.1
ENSPFOG00000016585
1000
ENSXMAG00000011374
GI_554858967
399
GI_548338012
707
1000
GI_499023404
Gene: med24
1000
GI_584012330
1000
ENSONIG00000019926
595
267
ENSORLG00000008227
ENSGACG00000008529
508
ENSTNIG00000012846
1000
ENSTRUG00000016382
ENSAMXG00000012180
1000
ENSDARG00000032459
ENSLOCG00000012864

Figure S1

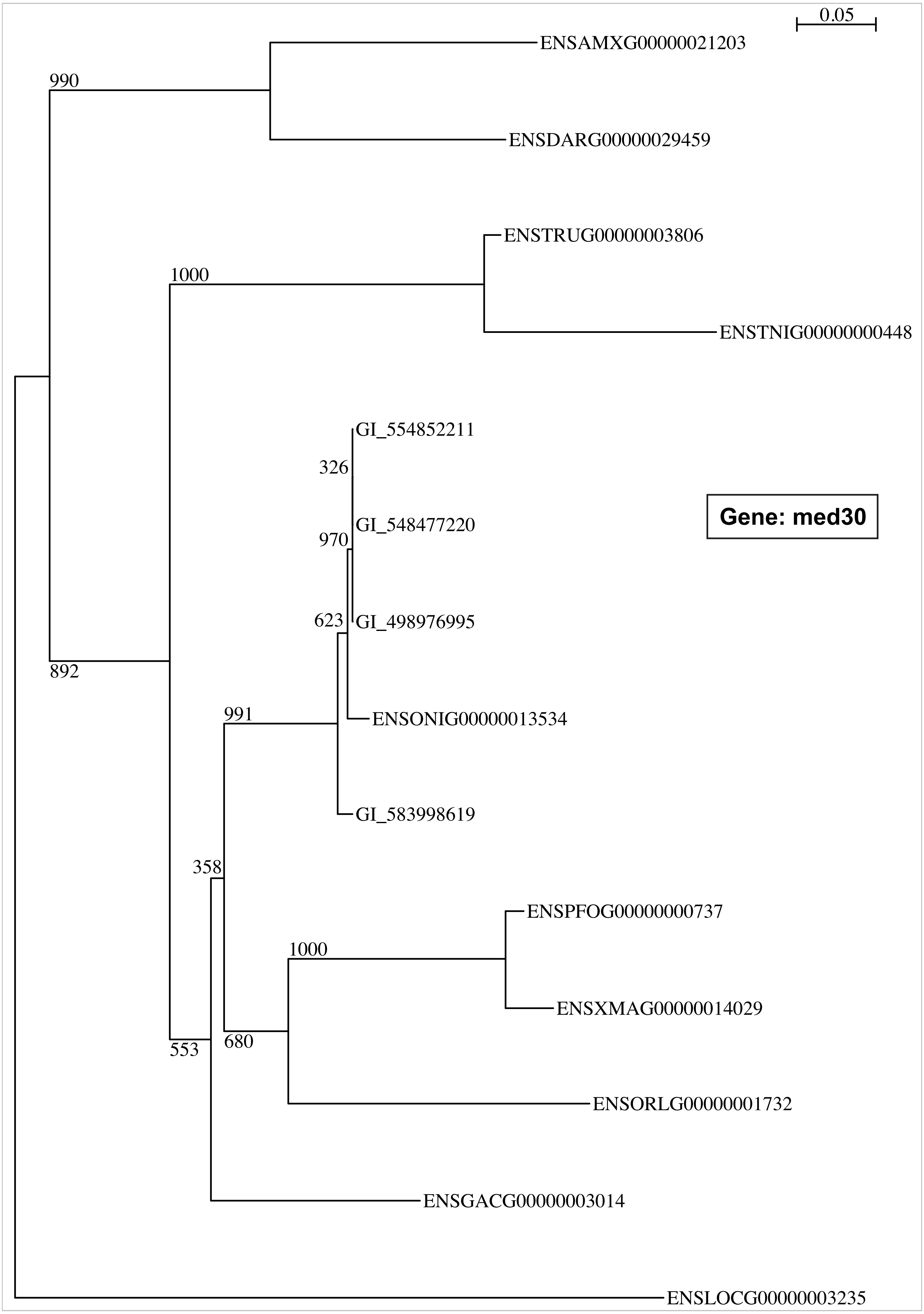
0.05
ENSAMXG00000021203
990
ENSDARG00000029459
ENSTRUG00000003806
1000
ENSTNIG00000000448
GI_554852211
326
Gene: med30
GI_548477220
970
623
GI_498976995
892
991
ENSONIG00000013534
GI_583998619
358
ENSPFOG00000000737
1000
ENSXMAG00000014029
553
680
ENSORLG00000001732
ENSGACG00000003014
ENSLOCG00000003235

Figure S1

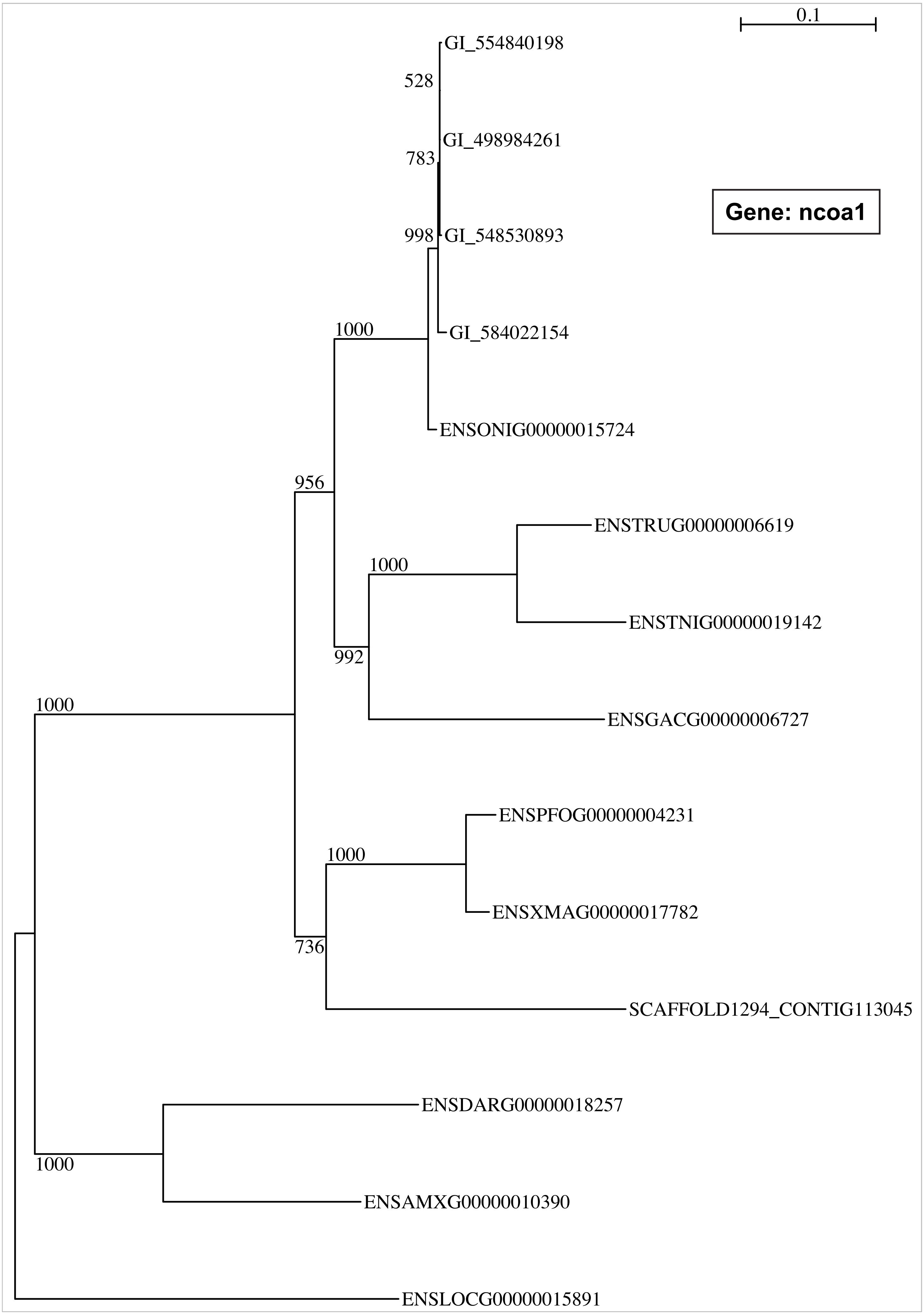
0.1
Gene: ncoa1
GI_554840198
528
GI_498984261
783
998
GI_548530893
1000
GI_584022154
ENSONIG00000015724
956
ENSTRUG00000006619
1000
ENSTNIG00000019142
992
1000
ENSGACG00000006727
ENSPFOG00000004231
1000
ENSXMAG00000017782
736
SCAFFOLD1294_CONTIG113045
ENSDARG00000018257
1000
ENSAMXG00000010390
ENSLOCG00000015891

Figure S1

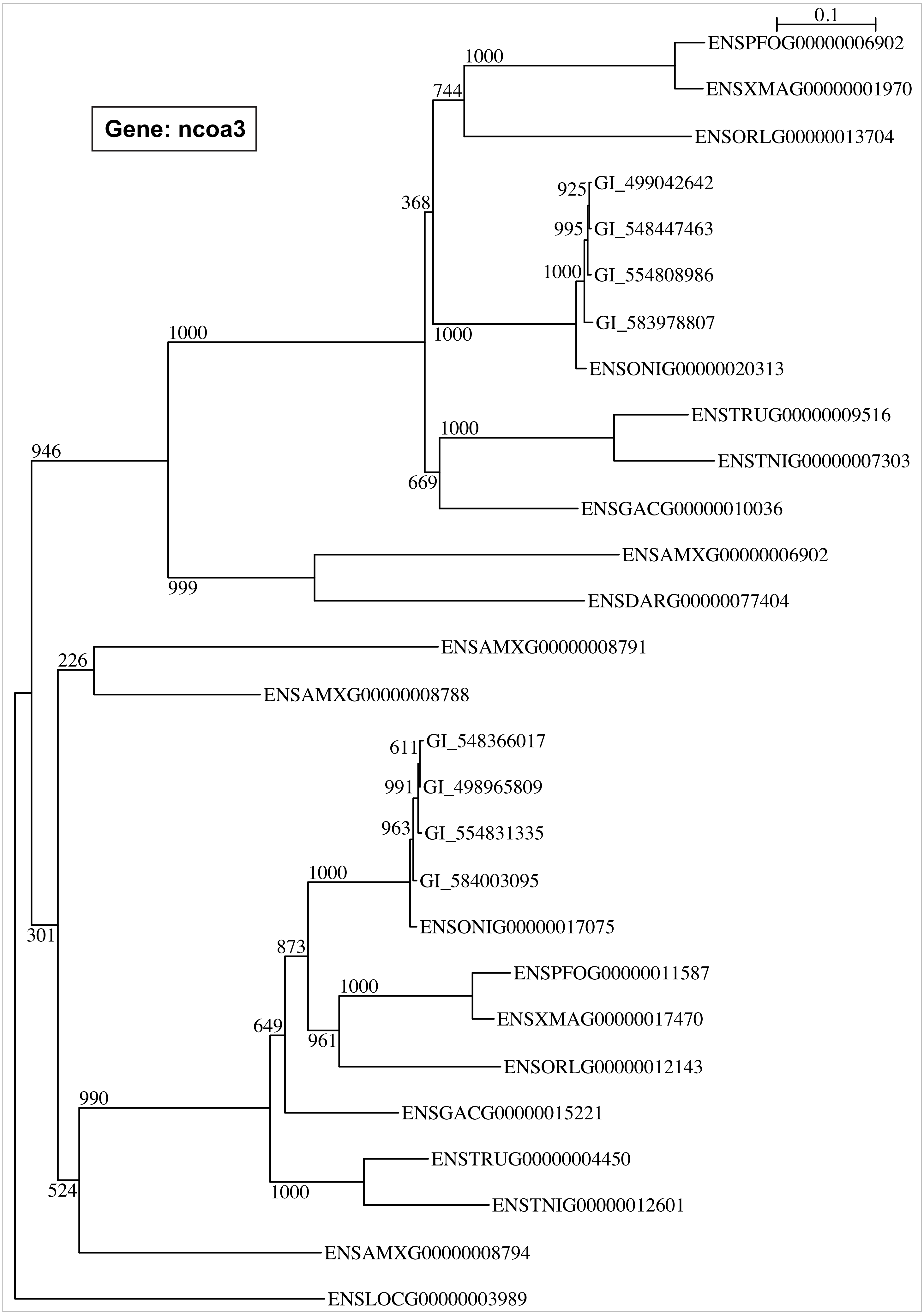
Gene: ncoa3
0.1
1000
744
368
1000
946
925
995
1000
1000
1000
669
999
226
611
991
963
1000
873
1000
649
961
990
524
1000
301
ENSPFOG00000006902
ENSXMAG00000001970
ENSORLG00000013704
GI_499042642
GI_548447463
GI_554808986
GI_583978807
ENSONIG00000020313
ENSTRUG00000009516
ENSTNIG00000007303
ENSGACG00000010036
ENSAMXG00000006902
ENSDARG00000077404
ENSAMXG00000008791
ENSAMXG00000008788
GI_548366017
GI_498965809
GI_554831335
GI_584003095
ENSONIG00000017075
ENSPFOG00000011587
ENSXMAG00000017470
ENSORLG00000012143
ENSGACG00000015221
ENSTRUG00000004450
ENSTNIG00000012601
ENSAMXG00000008794
ENSLOCG00000003989

Figure S1

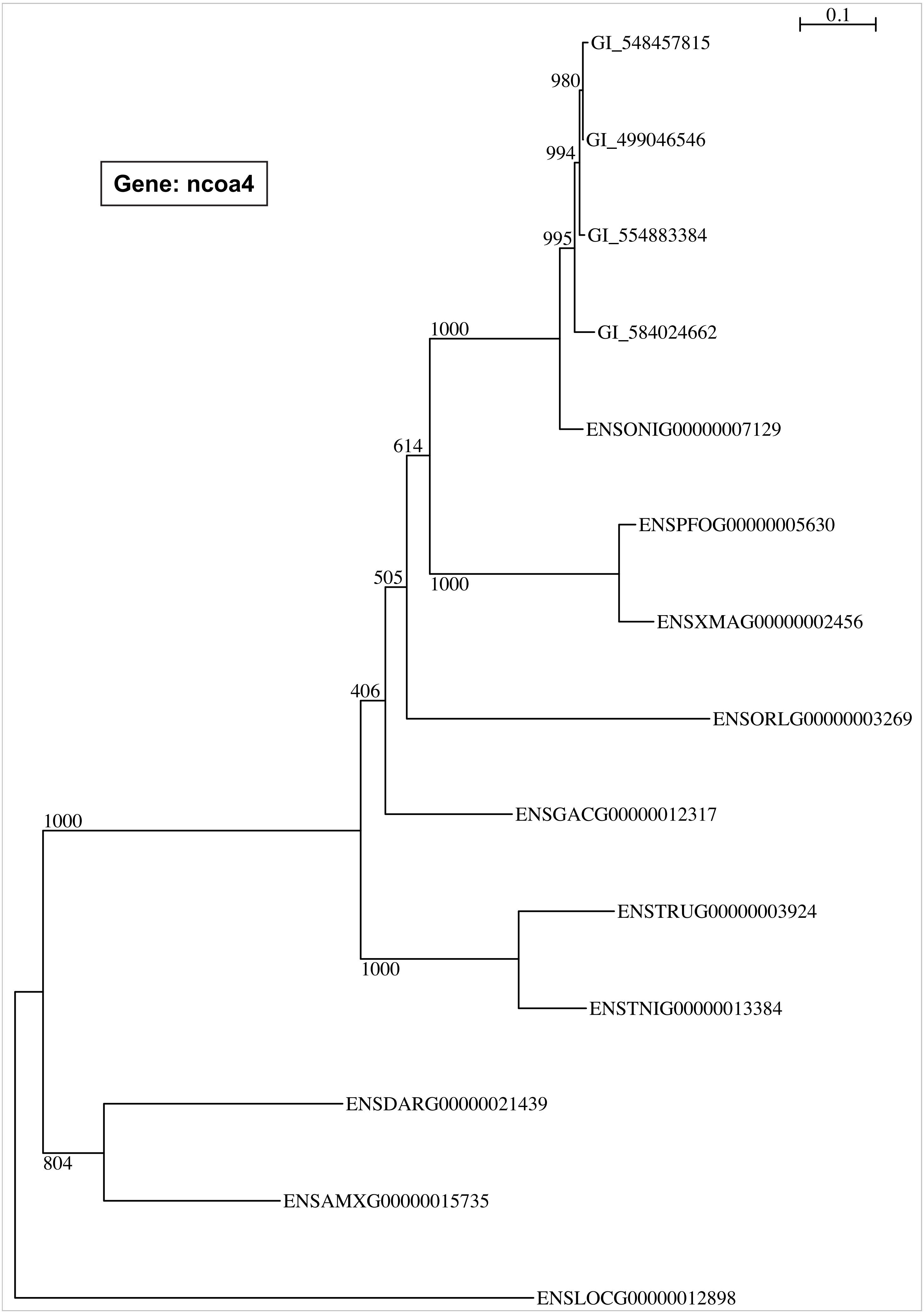
0.1
Gene: ncoa4
GI_548457815
980
GI_499046546
994
GI_554883384
995
1000
GI_584024662
ENSONIG00000007129
614
ENSPFOG00000005630
505
1000
ENSXMAG00000002456
406
ENSORLG00000003269
1000
ENSGACG00000012317
ENSTRUG00000003924
1000
ENSTNIG00000013384
ENSDARG00000021439
804
ENSAMXG00000015735
ENSLOCG00000012898

Figure S1

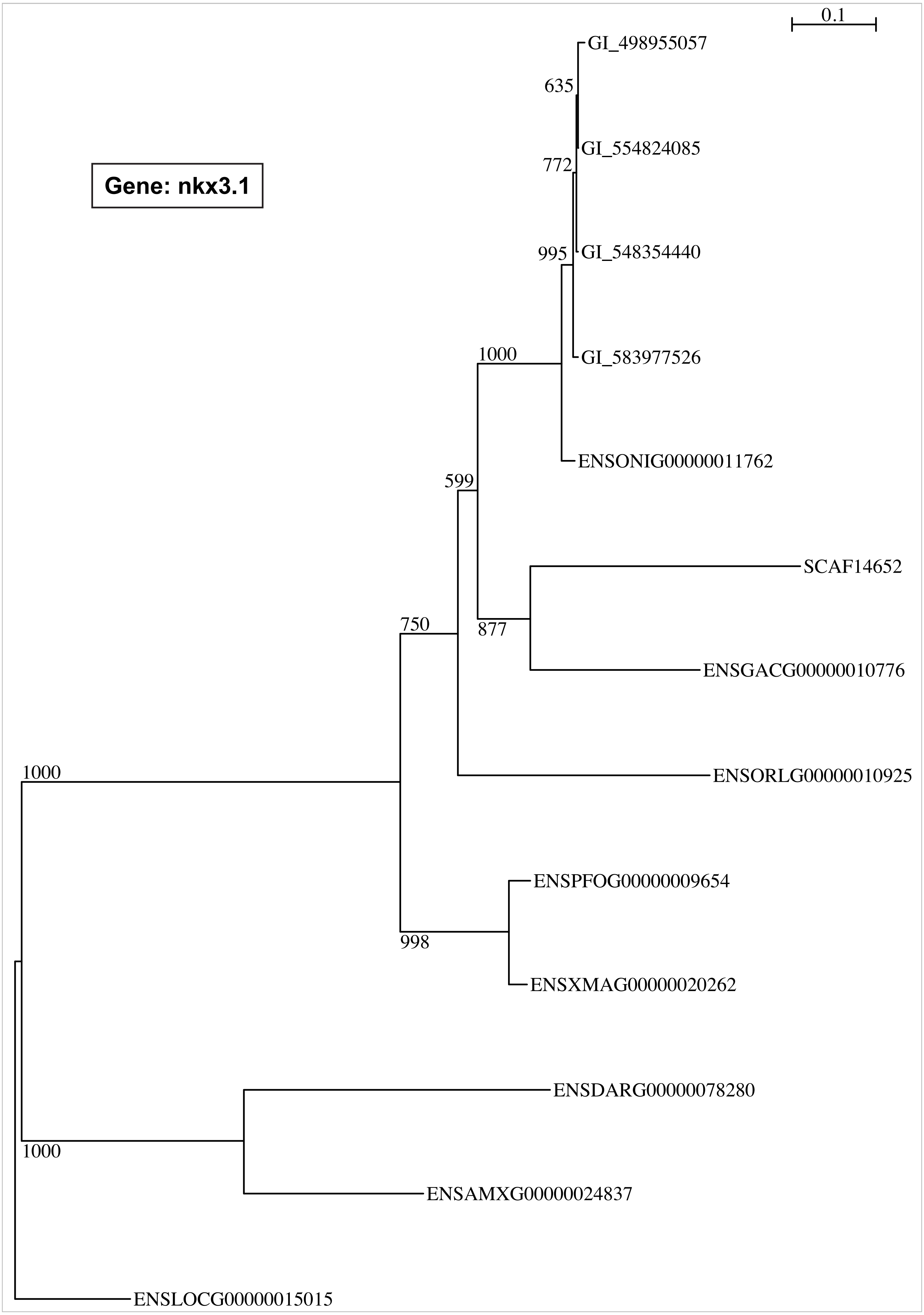

0.1
Gene: nkx3.1
GI_498955057
635
GI_554824085
772
GI_548354440
995
GI_583977526
1000
ENSONIG00000011762
599
SCAF14652
877
750
ENSGACG00000010776
ENSORLG00000010925
1000
ENSPFOG00000009654
998
ENSXMAG00000020262
ENSDARG00000078280
1000
ENSAMXG00000024837
ENSLOCG00000015015

Figure S1

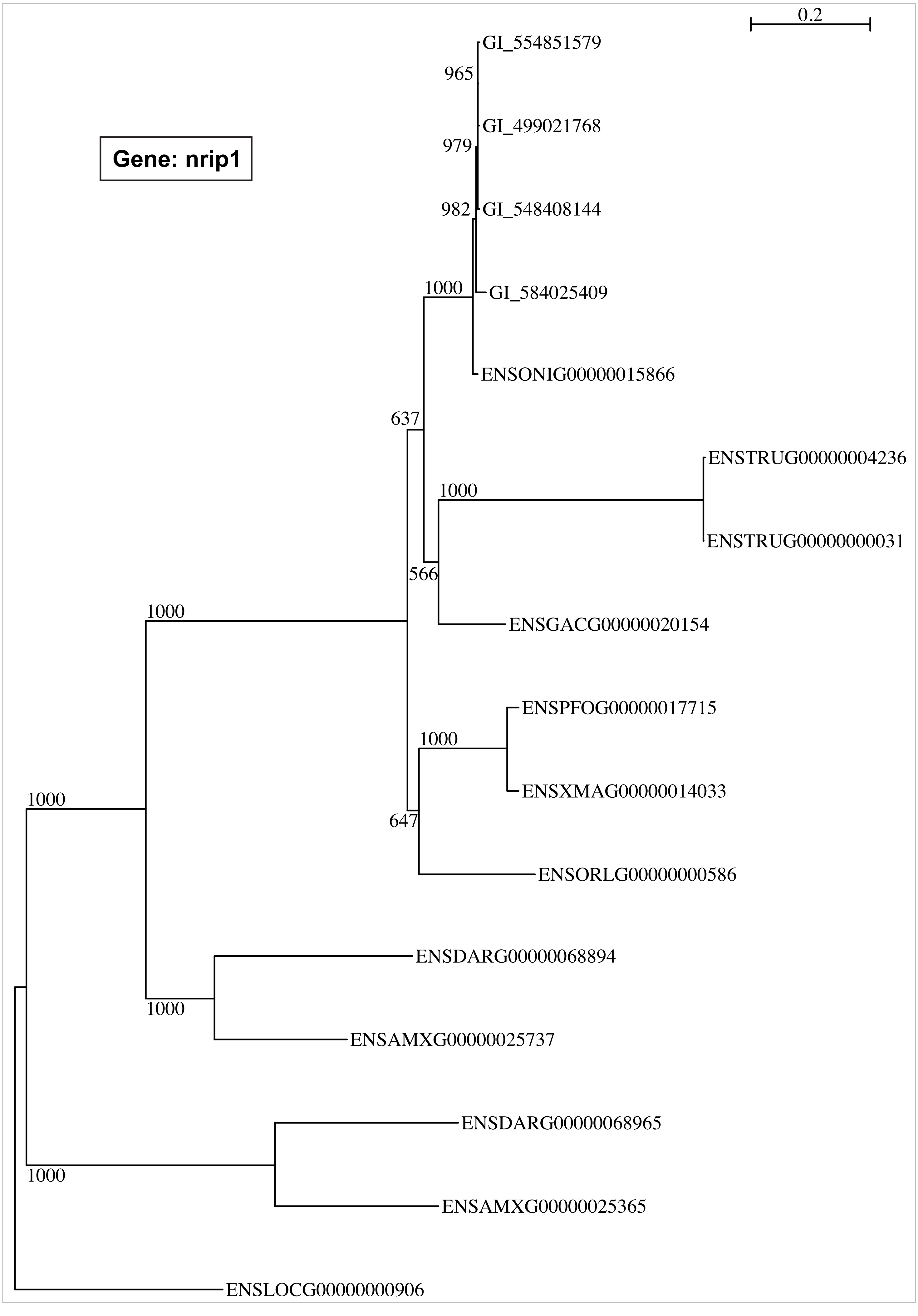

0.2
Gene: nrip1
GI_554851579
965
GI_499021768
979
982
GI_548408144
1000
GI_584025409
ENSONIG00000015866
637
ENSTRUG00000004236
1000
ENSTRUG00000000031
566
1000
ENSGACG00000020154
ENSPFOG00000017715
1000
ENSXMAG00000014033
1000
647
ENSORLG00000000586
ENSDARG00000068894
1000
ENSAMXG00000025737
ENSDARG00000068965
1000
ENSAMXG00000025365
ENSLOCG00000000906

Figure S1

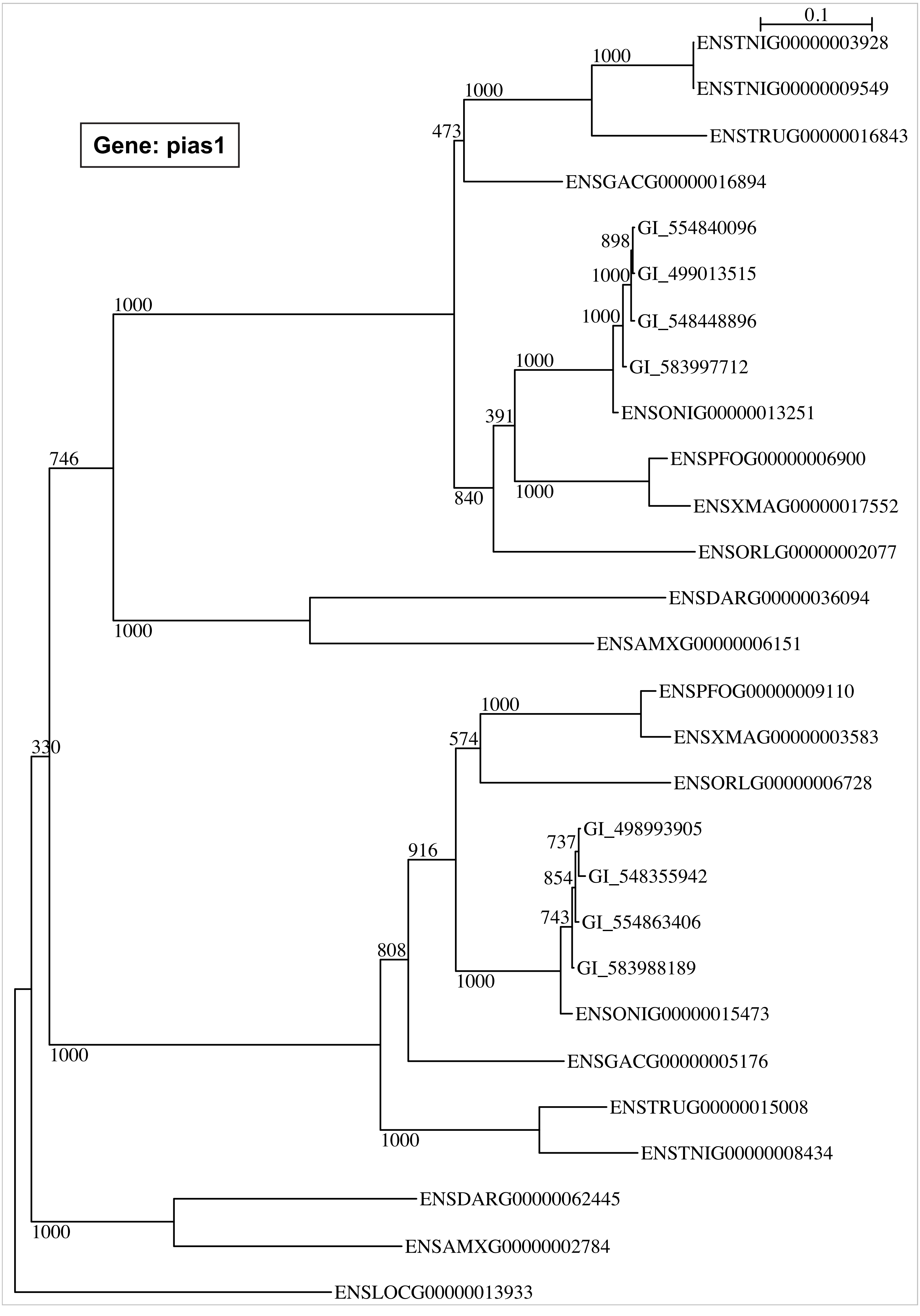

 T. Lorin, W. Salzburger & A. Böhne

Figure S1

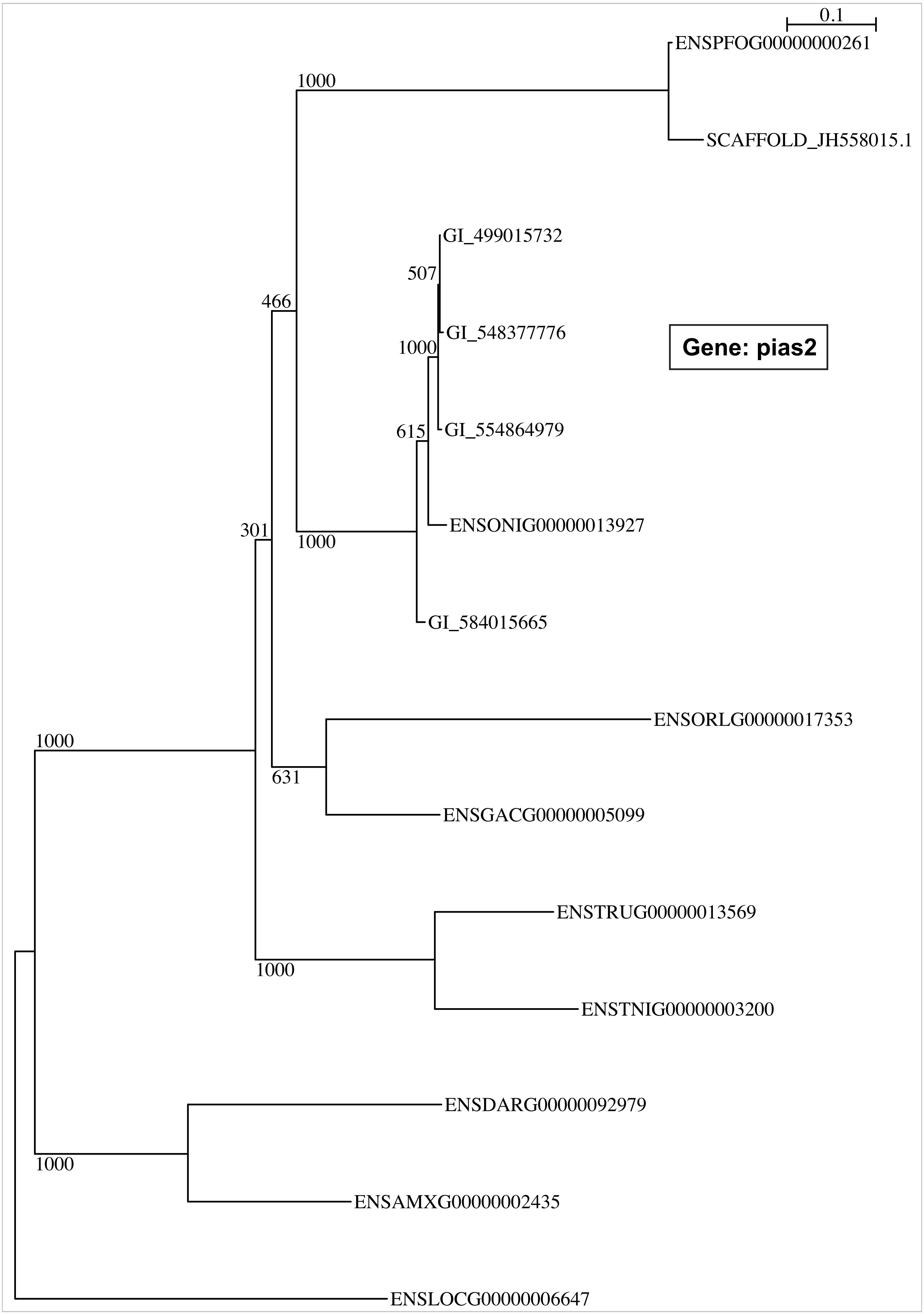
0.1
ENSPFOG00000000261
1000
SCAFFOLD_JH558015.1
GI_499015732
507
466
GI_548377776
1000
Gene: pias2
615
GI_554864979
301
ENSONIG00000013927
1000
GI_584015665
1000
ENSORLG00000017353
631
ENSGACG00000005099
ENSTRUG00000013569
1000
ENSTNIG00000003200
ENSDARG00000092979
1000
ENSAMXG00000002435
ENSLOCG00000006647

Figure S1

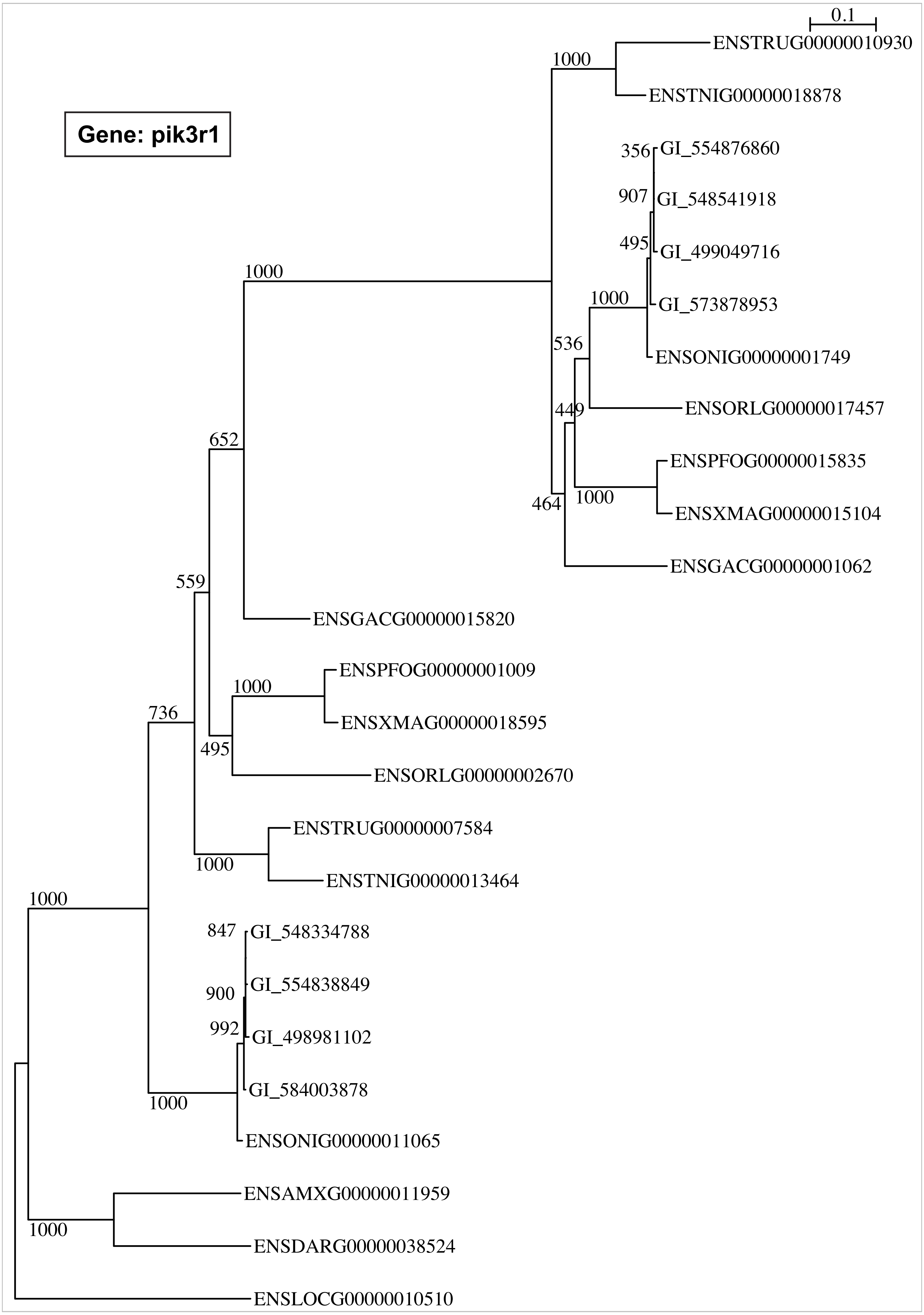
0.1
Gene: pik3r1
ENSTRUG00000010930
ENSTNIG00000018878
GI_554876860
GI_548541918
GI_499049716
GI_573878953
ENSONIG00000001749
ENSORLG00000017457
ENSPFOG00000015835
ENSXMAG00000015104
ENSGACG00000001062
ENSGACG00000015820
ENSPFOG00000001009
ENSXMAG00000018595
ENSORLG00000002670
ENSTRUG00000007584
ENSTNIG00000013464
GI_548334788
GI_554838849
GI_498981102
GI_584003878
ENSONIG00000011065
ENSAMXG00000011959
ENSDARG00000038524
ENSLOCG00000010510
1000
356
907
495
1000
536
449
464
1000
1000
652
559
1000
495
736
1000
1000
847
900
992
1000
1000

Figure S1

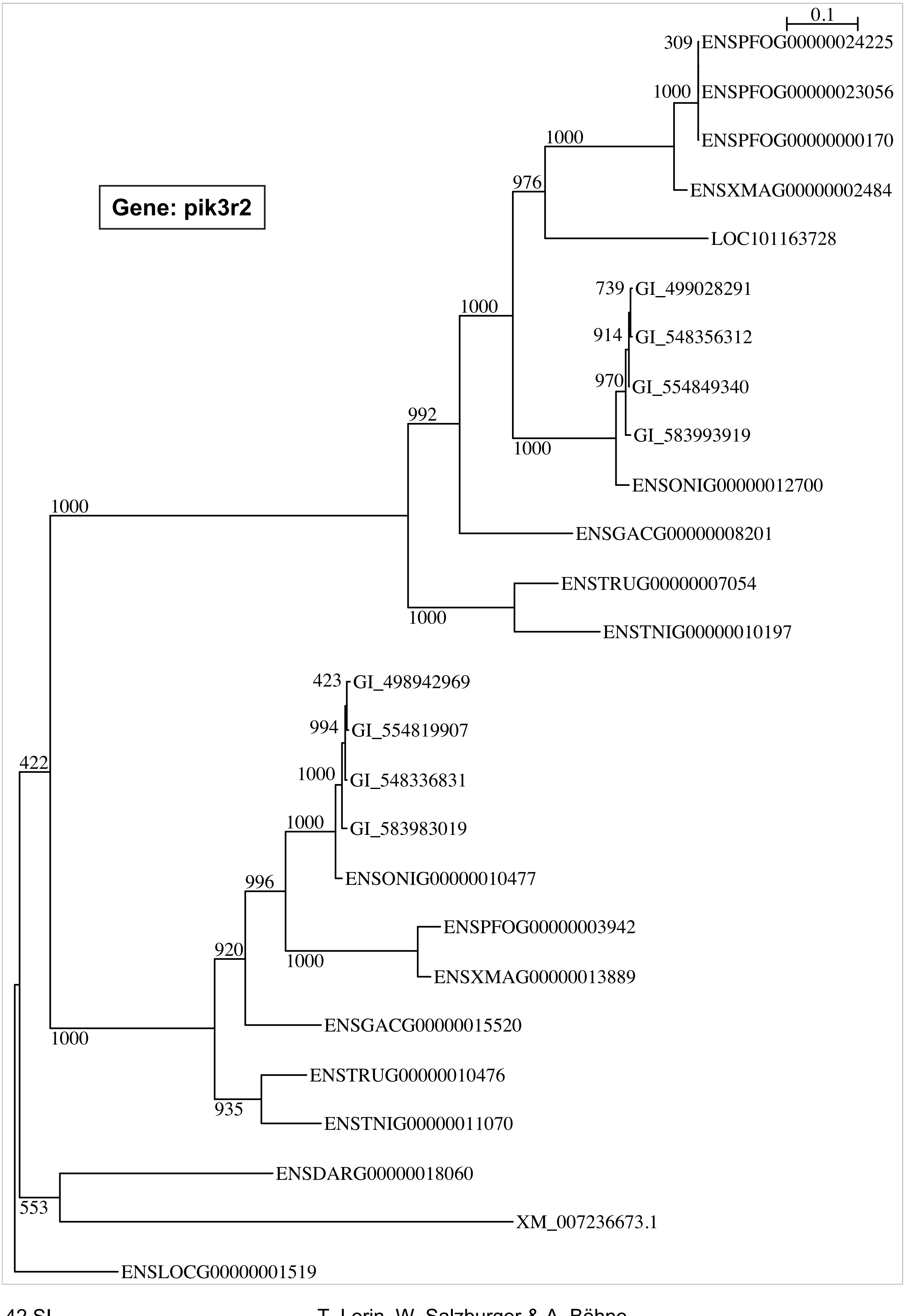
0.1
Gene: pik3r2
309
ENSPFOG00000024225
1000
ENSPFOG00000023056
1000
ENSPFOG00000000170
976
ENSXMAG00000002484
LOC101163728
739
GI_499028291
1000
914
GI_548356312
970
GI_554849340
992
GI_583993919
1000
ENSONIG00000012700
1000
ENSGACG00000008201
ENSTRUG00000007054
1000
ENSTNIG00000010197
423
GI_498942969
994
GI_554819907
422
1000
GI_548336831
1000
GI_583983019
996
ENSONIG00000010477
ENSPFOG00000003942
920
1000
ENSXMAG00000013889
ENSGACG00000015520
1000
ENSTRUG00000010476
935
ENSTNIG00000011070
ENSDARG00000018060
553
XM_007236673.1
ENSLOCG00000001519

Figure S1

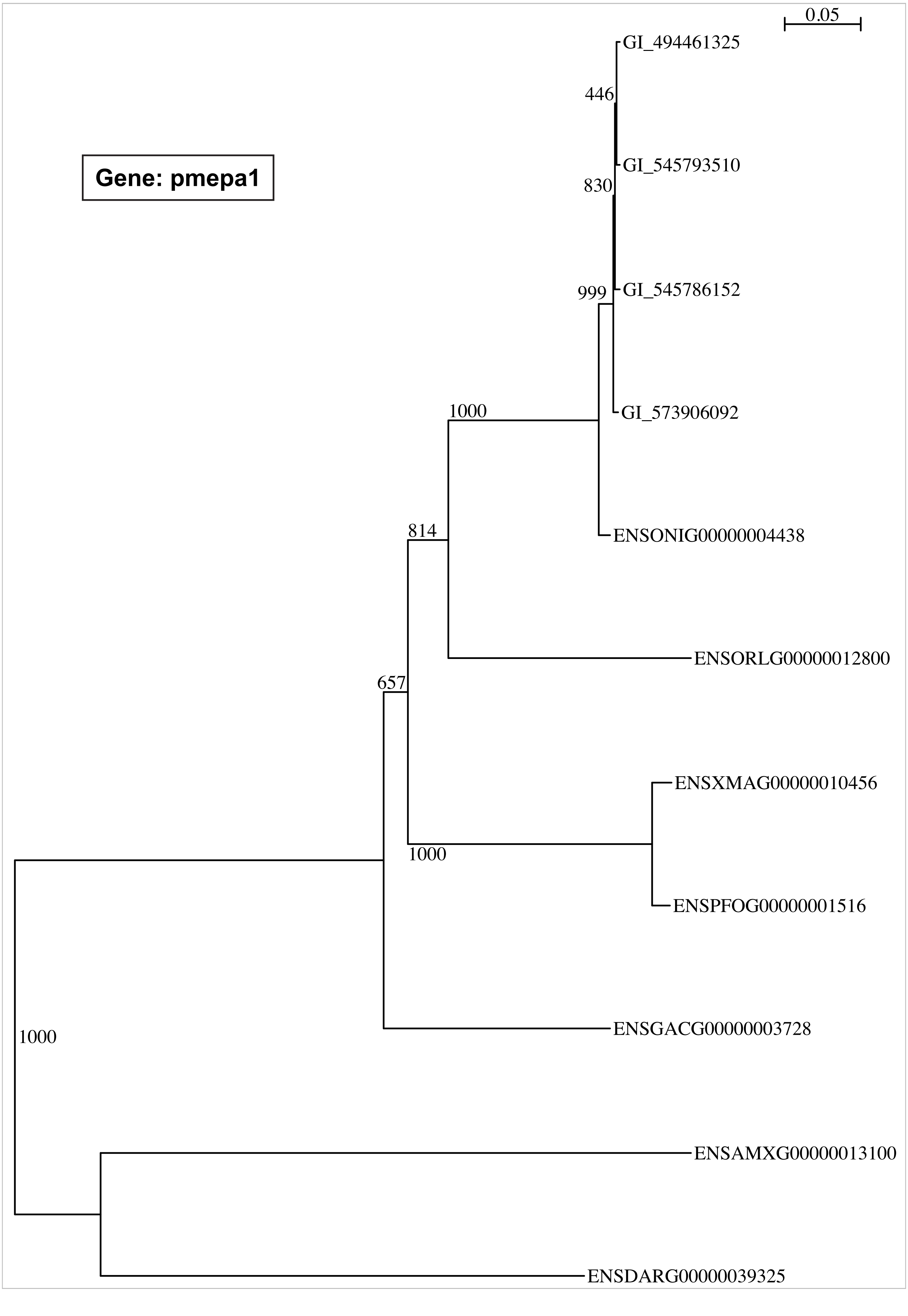

0.05
Gene: pmepa1
GI_494461325
446
GI_545793510
830
GI_545786152
999
GI_573906092
1000
ENSONIG00000004438
814
ENSORLG00000012800
657
ENSXMAG00000010456
1000
ENSPFOG00000001516
ENSGACG00000003728
1000
ENSAMXG00000013100
ENSDARG00000039325

Figure S1

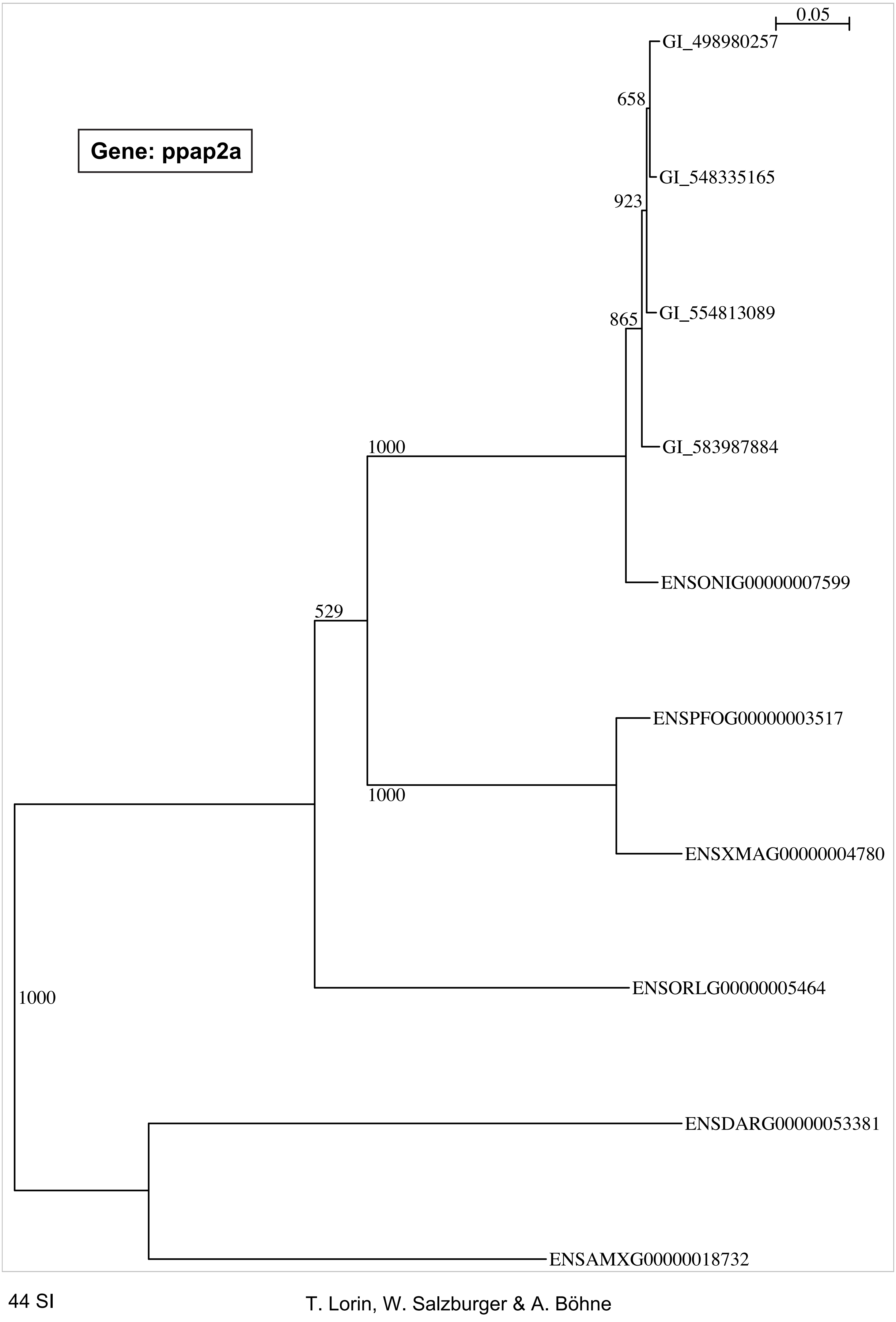

0.05
Gene: ppap2a
GI_498980257
658
GI_548335165
923
GI_554813089
865
GI_583987884
1000
ENSONIG00000007599
529
ENSPFOG00000003517
1000
ENSXMAG00000004780
ENSORLG00000005464
1000
ENSDARG00000053381
ENSAMXG00000018732

Figure S1

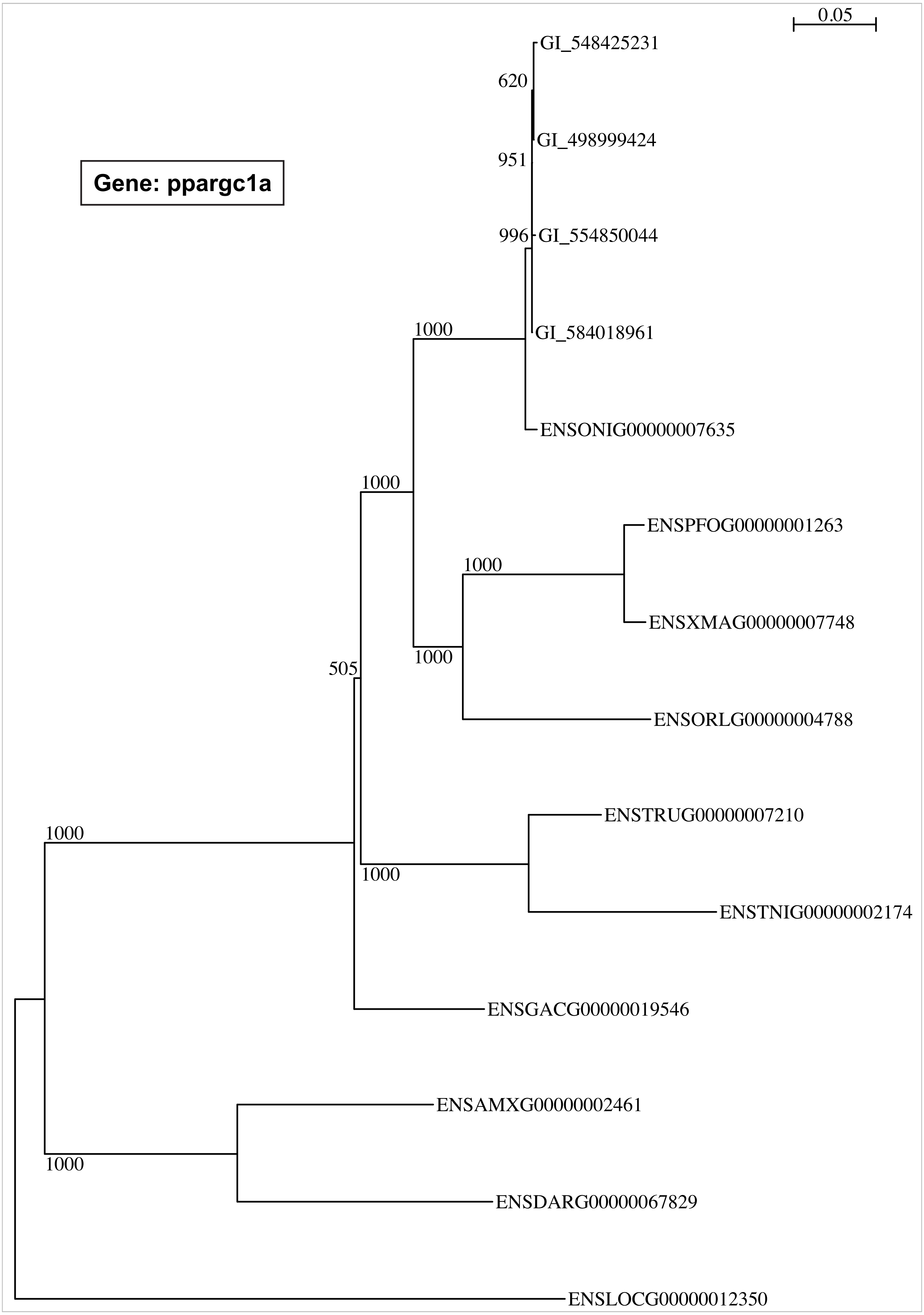
0.05
GI_548425231
620
GI_498999424
951
Gene: ppargc1a
996
GI_554850044
1000
GI_584018961
ENSONIG00000007635
1000
ENSPFOG00000001263
1000
ENSXMAG00000007748
1000
505
ENSORLG00000004788
ENSTRUG00000007210
1000
1000
ENSTNIG00000002174
ENSGACG00000019546
ENSAMXG00000002461
1000
ENSDARG00000067829
ENSLOCG00000012350

Figure S1

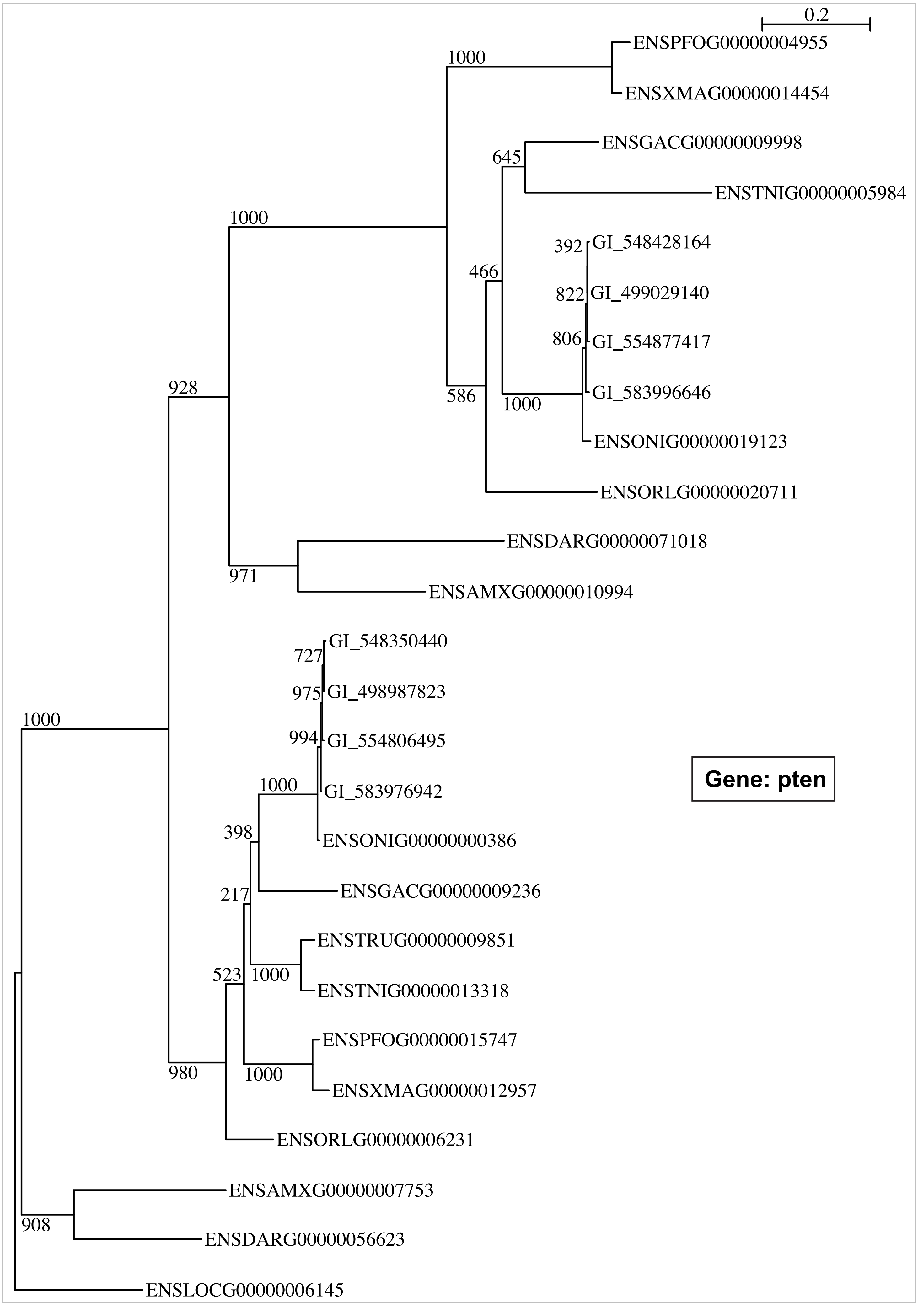
0.2
1000
ENSPFOG00000004955
ENSXMAG00000014454
645
ENSGACG00000009998
ENSTNIG00000005984
1000
392
GI_548428164
466
822
GI_499029140
806
GI_554877417
928
586
1000
GI_583996646
ENSONIG00000019123
ENSORLG00000020711
ENSDARG00000071018
971
ENSAMXG00000010994
GI_548350440
727
975
GI_498987823
1000
994
GI_554806495
1000
GI_583976942
398
ENSONIG00000000386
ENSGACG00000009236
217
ENSTRUG00000009851
523
1000
ENSTNIG00000013318
ENSPFOG00000015747
980
1000
ENSXMAG00000012957
ENSORLG00000006231
ENSAMXG00000007753
908
ENSDARG00000056623
ENSLOCG00000006145
Gene: pten

Figure S1

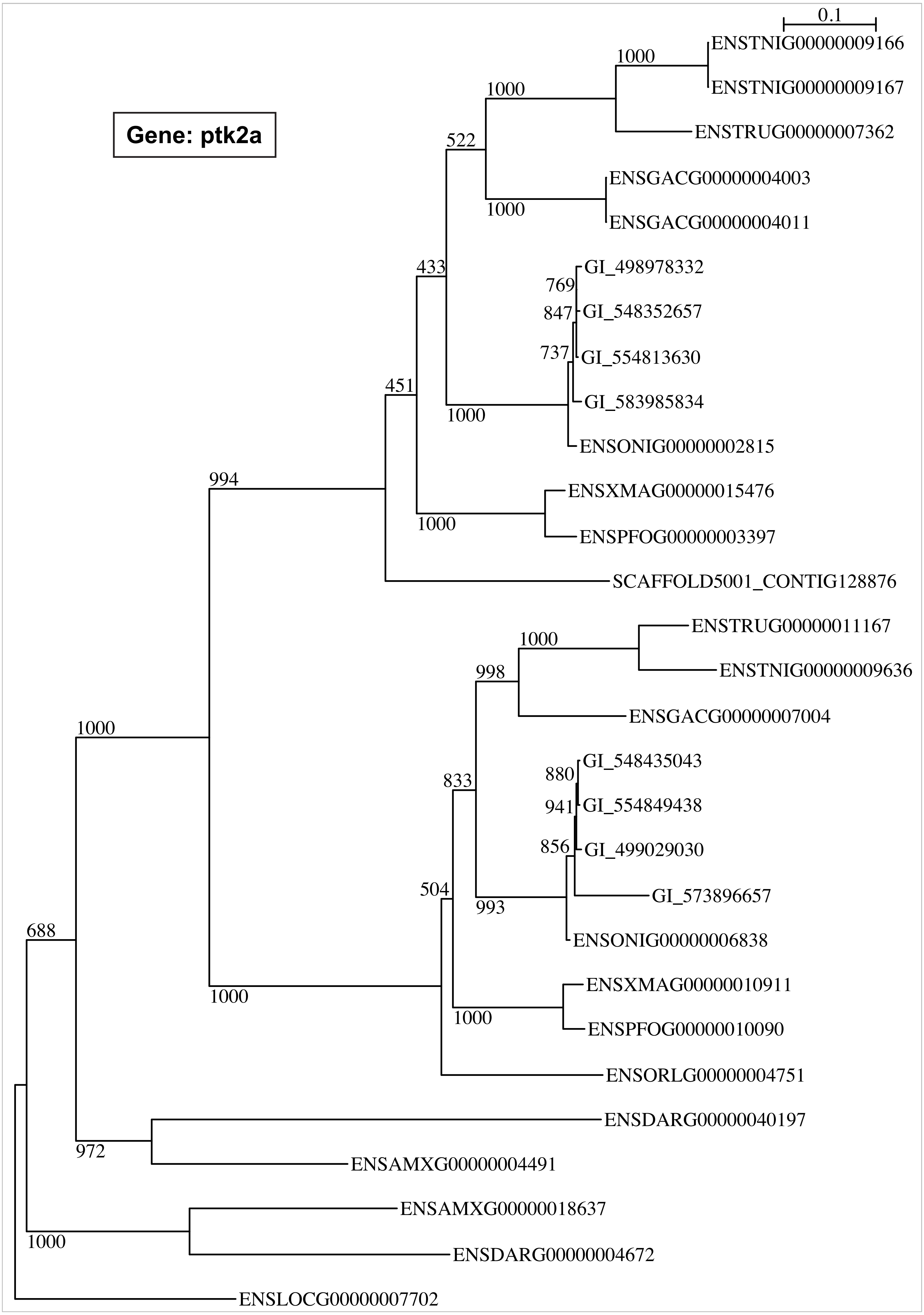

0.1
Gene: ptk2a
1000
1000
522
1000
433
769
847
737
451
1000
994
1000
1000
998
1000
833
880
941
856
504
993
1000
1000
688
972
1000
ENSTNIG00000009166
ENSTNIG00000009167
ENSTRUG00000007362
ENSGACG00000004003
ENSGACG00000004011
GI_498978332
GI_548352657
GI_554813630
GI_583985834
ENSONIG00000002815
ENSXMAG00000015476
ENSPFOG00000003397
SCAFFOLD5001_CONTIG128876
ENSTRUG00000011167
ENSTNIG00000009636
ENSGACG00000007004
GI_548435043
GI_554849438
GI_499029030
GI_573896657
ENSONIG00000006838
ENSXMAG00000010911
ENSPFOG00000010090
ENSORLG00000004751
ENSDARG00000040197
ENSAMXG00000004491
ENSAMXG00000018637
ENSDARG00000004672
ENSLOCG00000007702

Figure S1

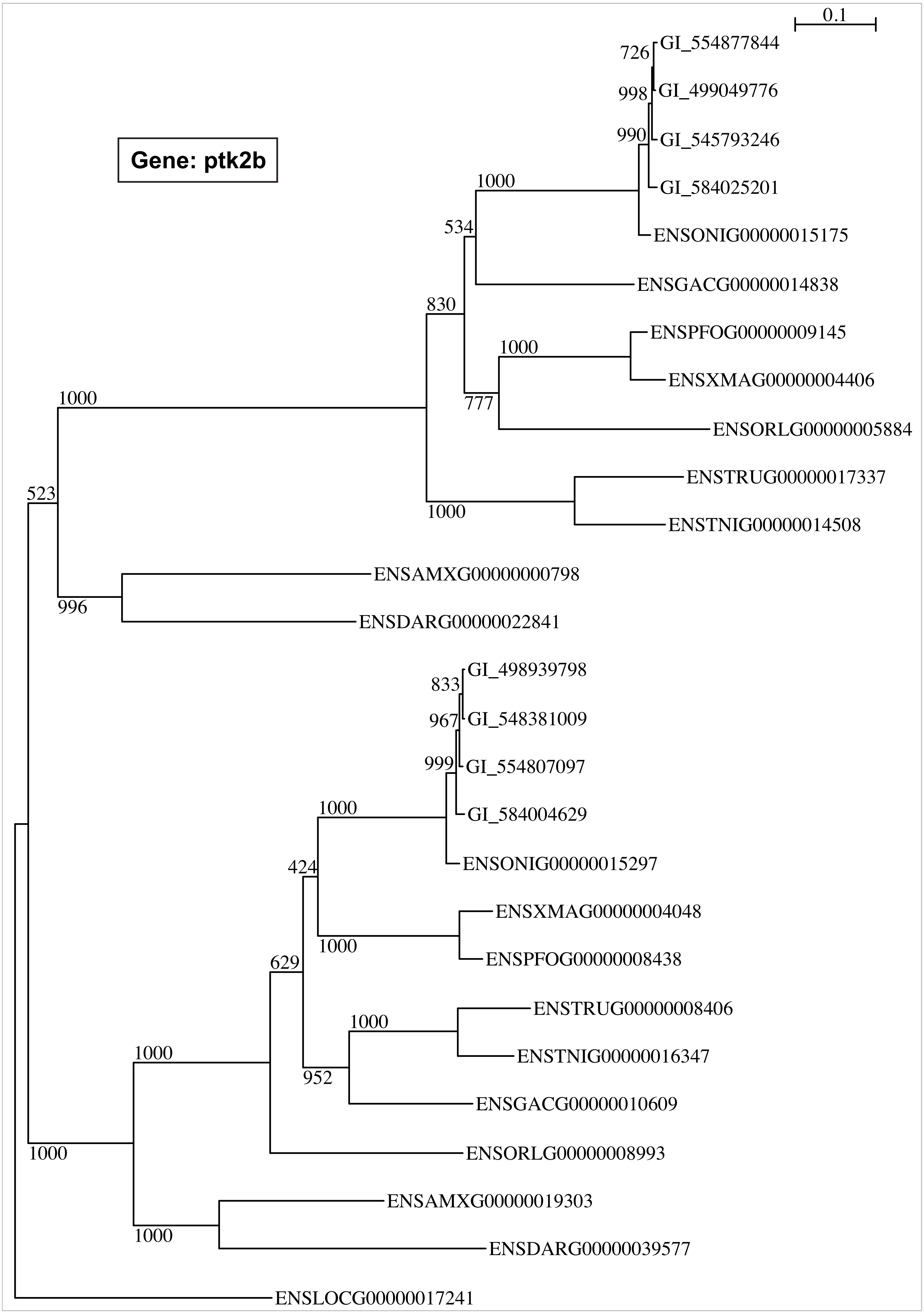
0.1
Gene: ptk2b
726
GI_554877844
998
GI_499049776
990
GI_545793246
GI_584025201
1000
ENSONIG00000015175
534
ENSGACG00000014838
830
ENSPFOG00000009145
1000
ENSXMAG00000004406
777
ENSORLG00000005884
1000
523
ENSTRUG00000017337
1000
ENSTNIG00000014508
ENSAMXG00000000798
996
ENSDARG00000022841
GI_498939798
833
GI_548381009
967
999
GI_554807097
1000
GI_584004629
424
ENSONIG00000015297
ENSXMAG00000004048
1000
629
ENSPFOG00000008438
ENSTRUG00000008406
1000
1000
ENSTNIG00000016347
952
ENSGACG00000010609
1000
ENSORLG00000008993
ENSAMXG00000019303
1000
ENSDARG00000039577
ENSLOCG00000017241

Figure S1

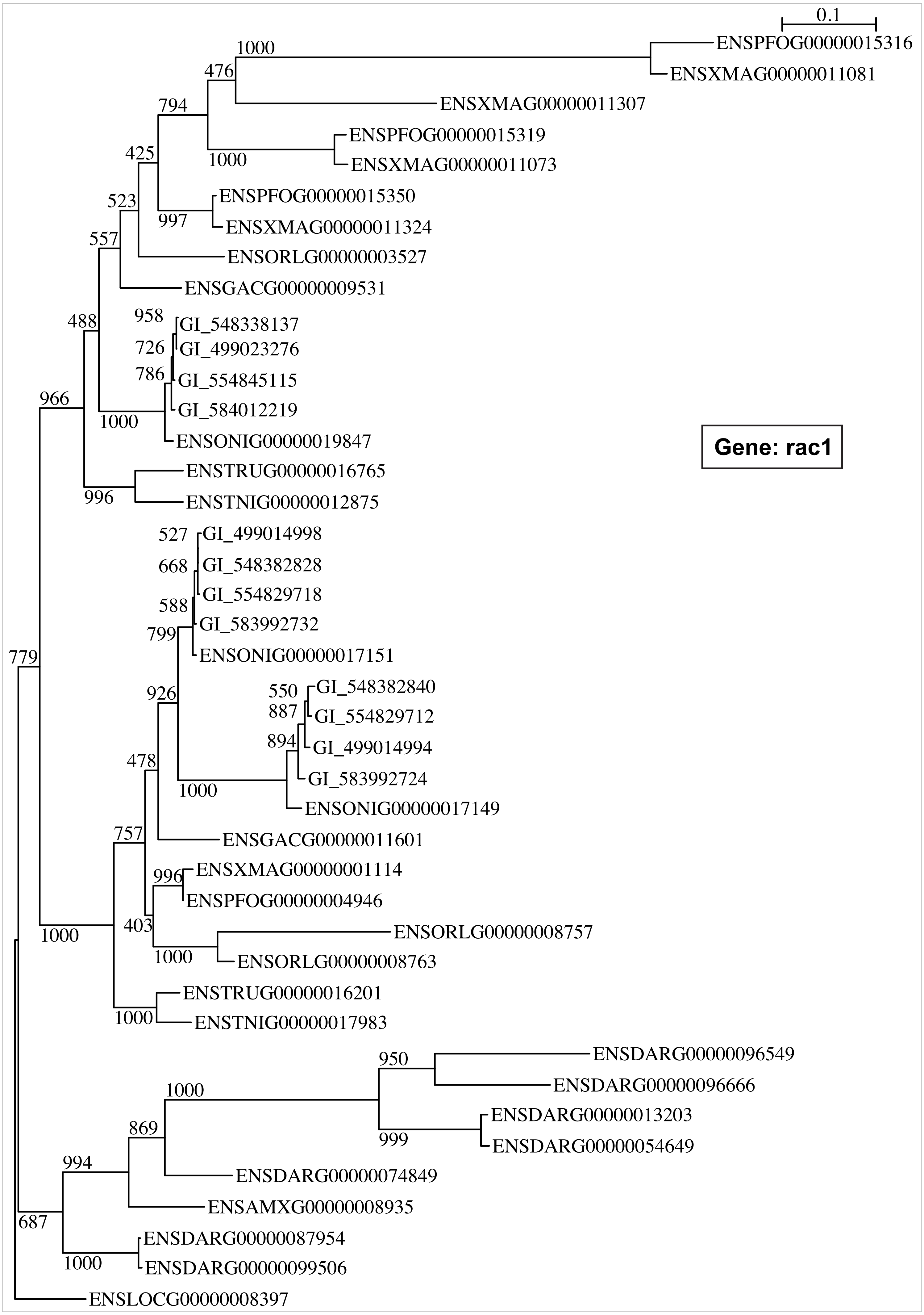
0.1
ENSPFOG00000015316
ENSXMAG00000011081
ENSXMAG00000011307
ENSPFOG00000015319
ENSXMAG00000011073
ENSPFOG00000015350
ENSXMAG00000011324
ENSORLG00000003527
ENSGACG00000009531
GI_548338137
GI_499023276
GI_554845115
GI_584012219
ENSONIG00000019847
Gene: rac1
ENSTRUG00000016765
ENSTNIG00000012875
GI_499014998
GI_548382828
GI_554829718
GI_583992732
ENSONIG00000017151
GI_548382840
GI_554829712
GI_499014994
GI_583992724
ENSONIG00000017149
ENSGACG00000011601
ENSXMAG00000001114
ENSPFOG00000004946
ENSORLG00000008757
ENSORLG00000008763
ENSTRUG00000016201
ENSTNIG00000017983
ENSDARG00000096549
ENSDARG00000096666
ENSDARG00000013203
ENSDARG00000054649
ENSDARG00000074849
ENSAMXG00000008935
ENSDARG00000087954
ENSDARG00000099506
ENSLOCG00000008397

Figure S1

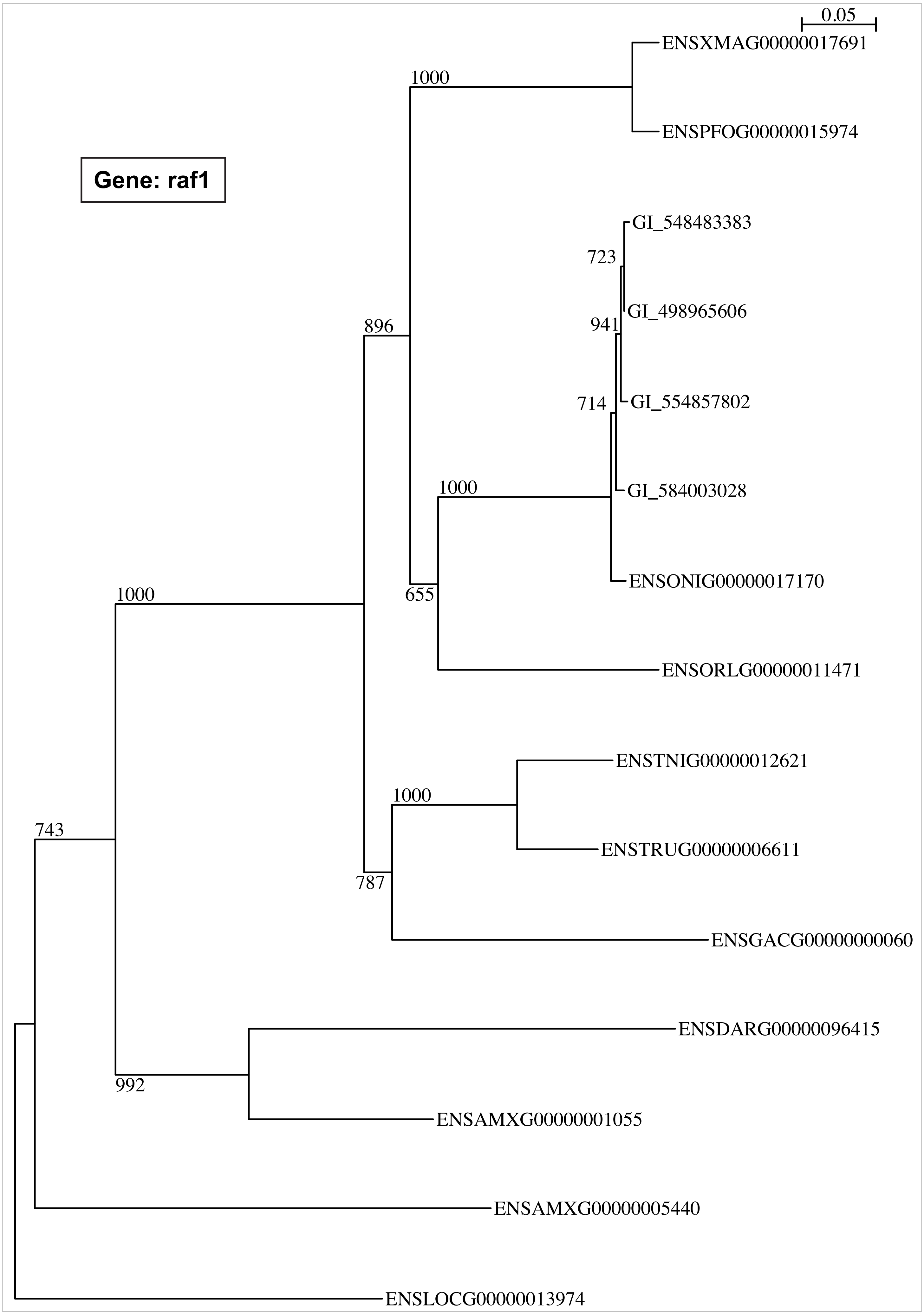
0.05
Gene: raf1
1000
ENSXMAG00000017691
ENSPFOG00000015974
GI_548483383
723
GI_498965606
941
896
GI_554857802
714
GI_584003028
1000
ENSONIG00000017170
1000
655
ENSORLG00000011471
ENSTNIG00000012621
1000
ENSTRUG00000006611
743
787
ENSGACG00000000060
ENSDARG00000096415
992
ENSAMXG00000001055
ENSAMXG00000005440
ENSLOCG00000013974

Figure S1

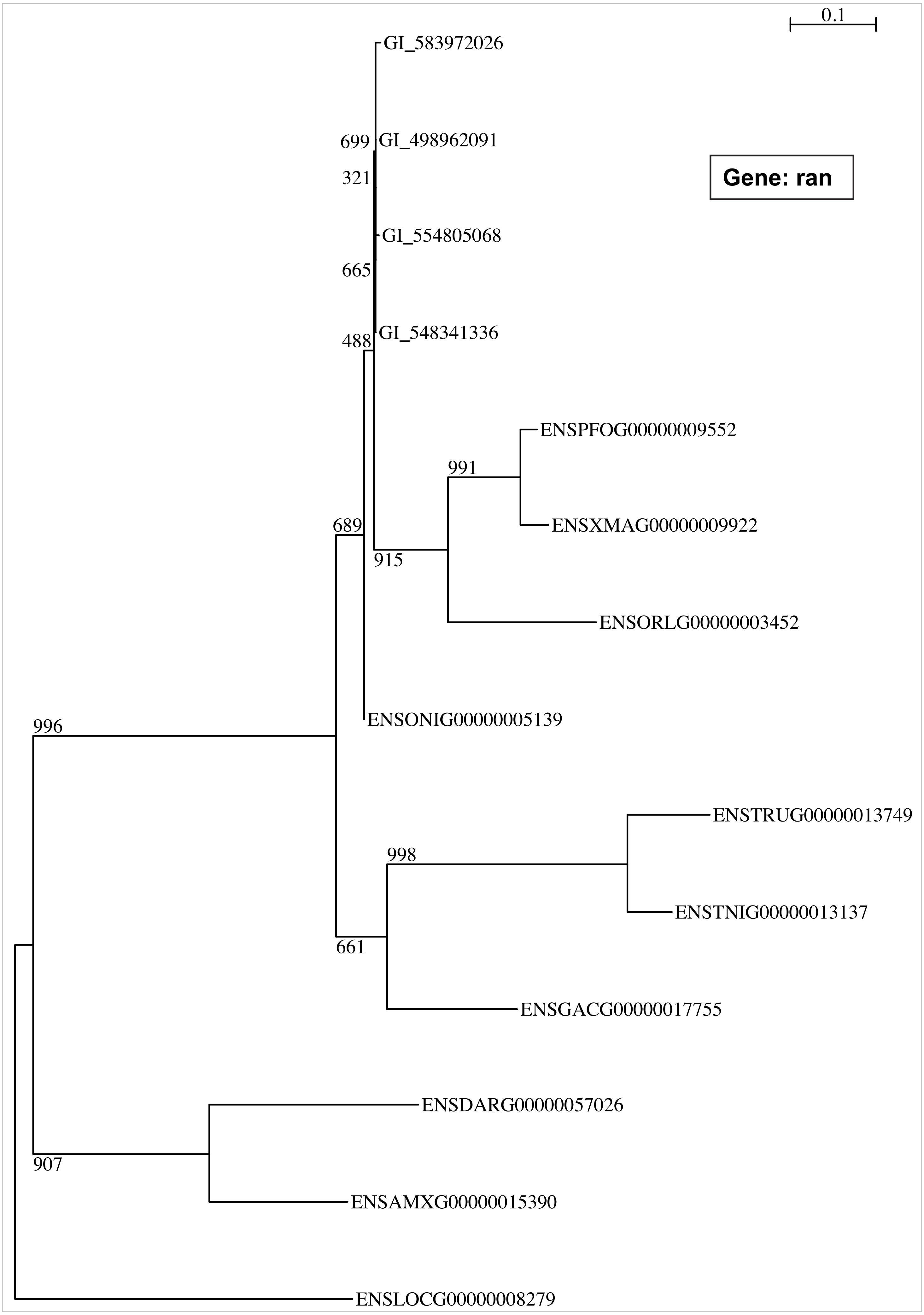

0.1
Gene: ran
GI_583972026
699
GI_498962091
321
GI_554805068
665
GI_548341336
488
ENSPFOG00000009552
991
ENSXMAG00000009922
689
915
ENSORLG00000003452
996
ENSONIG00000005139
ENSTRUG00000013749
998
ENSTNIG00000013137
661
ENSGACG00000017755
ENSDARG00000057026
907
ENSAMXG00000015390
ENSLOCG00000008279

Figure S1

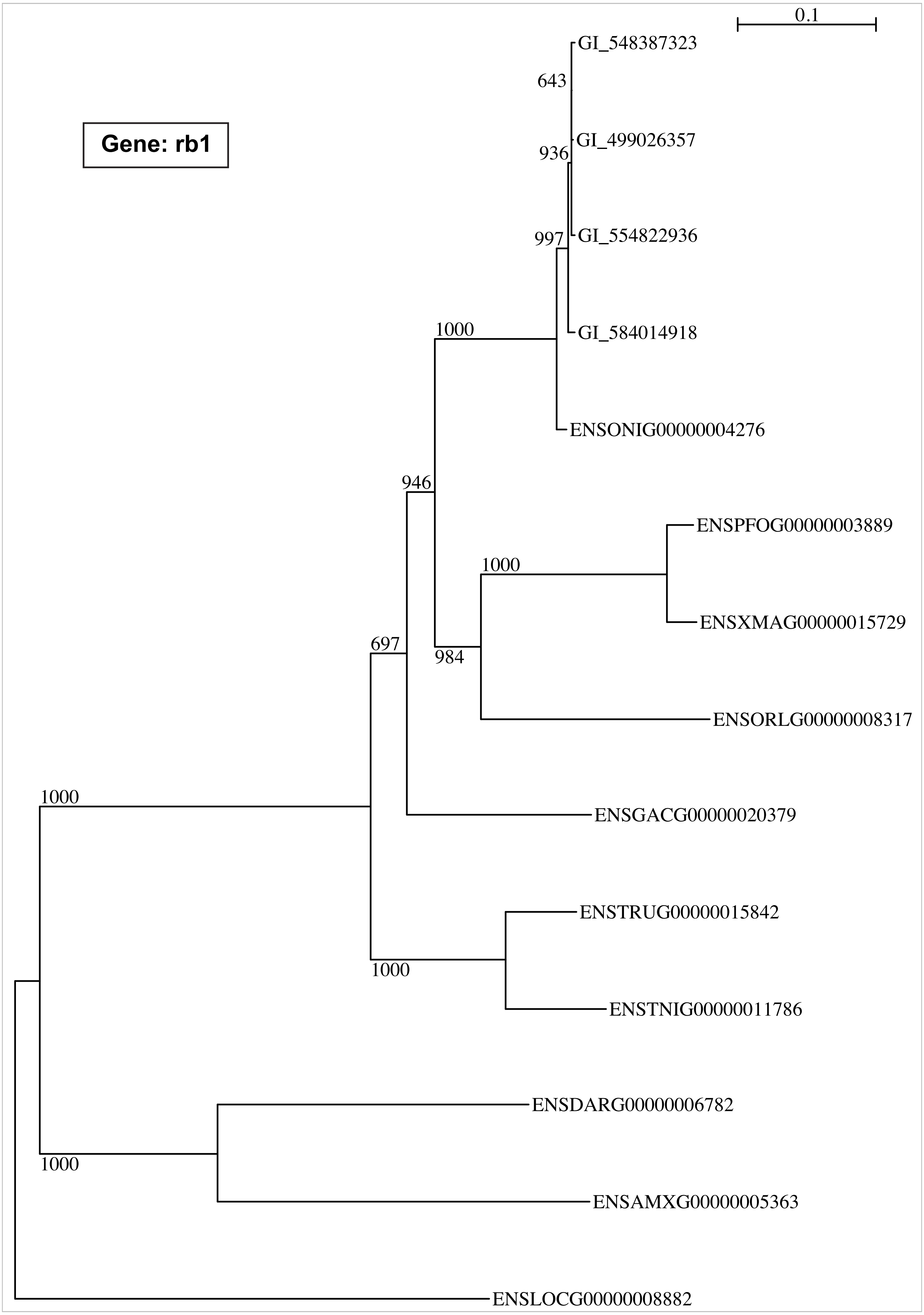
0.1
Gene: rb1
GI_548387323
643
GI_499026357
936
GI_554822936
997
GI_584014918
1000
ENSONIG00000004276
946
ENSPFOG00000003889
1000
ENSXMAG00000015729
697
984
ENSORLG00000008317
1000
ENSGACG00000020379
ENSTRUG00000015842
1000
ENSTNIG00000011786
ENSDARG00000006782
1000
ENSAMXG00000005363
ENSLOCG00000008882

Figure S1

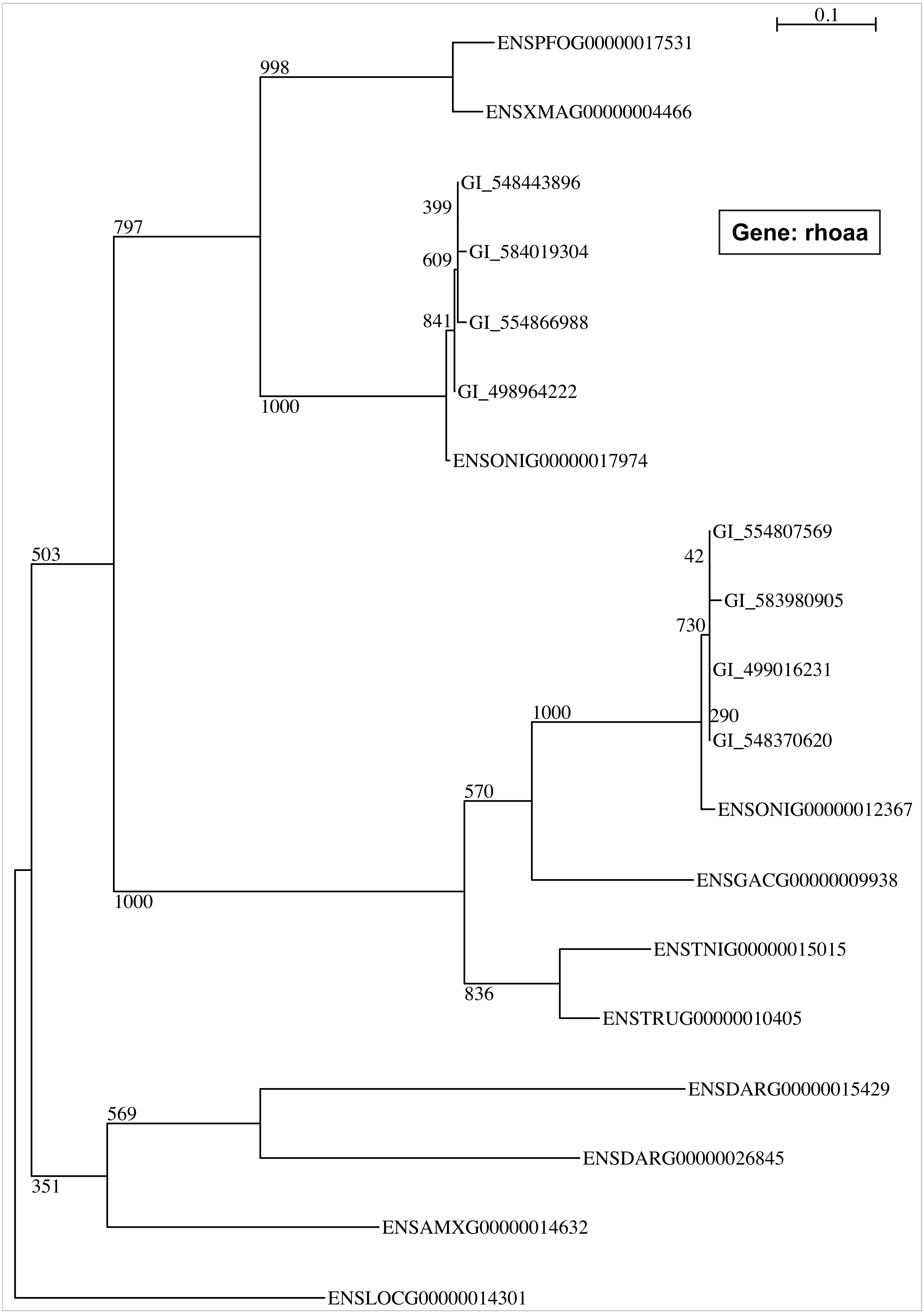
0.1
Gene: rhoaa
998
ENSPFOG00000017531
ENSXMAG00000004466
797
GI_548443896
399
GI_584019304
609
GI_554866988
841
GI_498964222
1000
ENSONIG00000017974
503
GI_554807569
42
GI_583980905
730
GI_499016231
290
GI_548370620
1000
ENSONIG00000012367
570
ENSGACG00000009938
1000
ENSTNIG00000015015
836
ENSTRUG00000010405
ENSDARG00000015429
569
ENSDARG00000026845
351
ENSAMXG00000014632
ENSLOCG00000014301

Figure S1

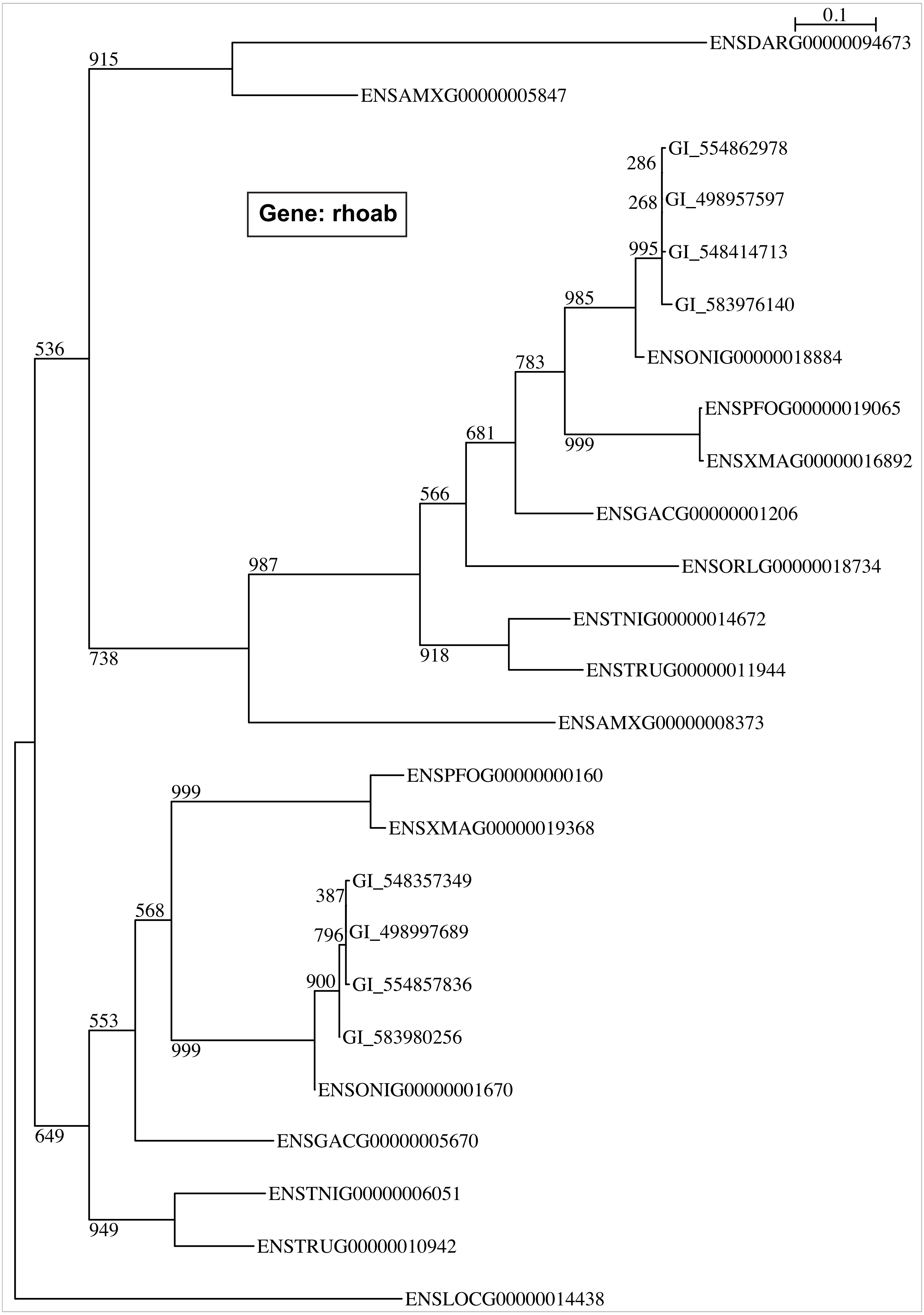
0.1
ENSDARG00000094673
915
ENSAMXG00000005847
GI_554862978
286
Gene: rhoab
268
GI_498957597
995
GI_548414713
985
GI_583976140
536
783
ENSONIG00000018884
ENSPFOG00000019065
681
999
ENSXMAG00000016892
566
ENSGACG00000001206
987
ENSORLG00000018734
ENSTNIG00000014672
738
918
ENSTRUG00000011944
ENSAMXG00000008373
ENSPFOG00000000160
999
ENSXMAG00000019368
GI_548357349
387
568
796
GI_498997689
900
GI_554857836
553
GI_583980256
999
ENSONIG00000001670
649
ENSGACG00000005670
ENSTNIG00000006051
949
ENSTRUG00000010942
ENSLOCG00000014438

Figure S1

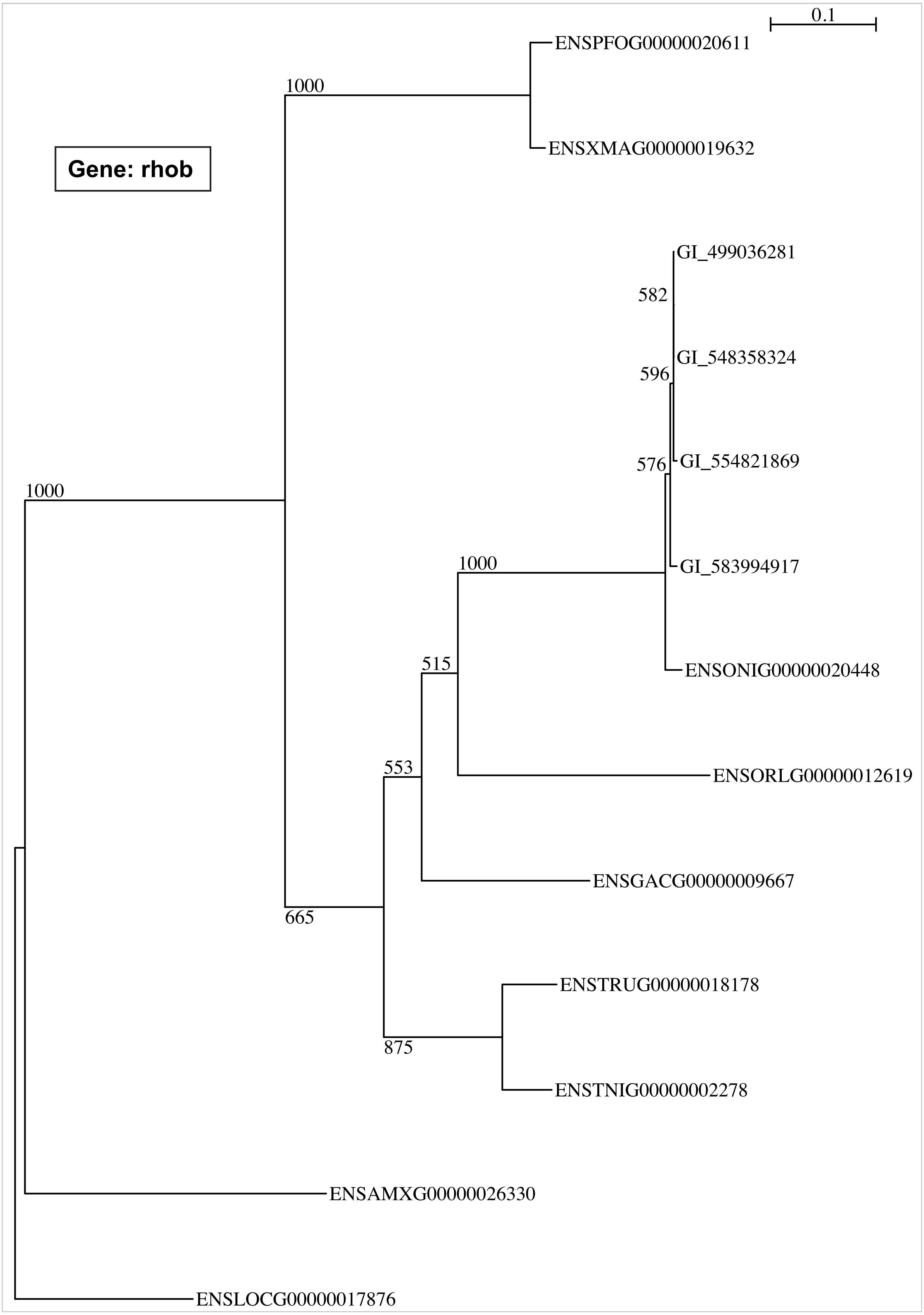
0.1
Gene: rhob
1000
ENSPFOG00000020611
ENSXMAG00000019632
GI_499036281
582
GI_548358324
596
GI_554821869
576
1000
GI_583994917
1000
ENSONIG00000020448
515
553
ENSORLG00000012619
ENSGACG00000009667
665
ENSTRUG00000018178
875
ENSTNIG00000002278
ENSAMXG00000026330
ENSLOCG00000017876

Figure S1

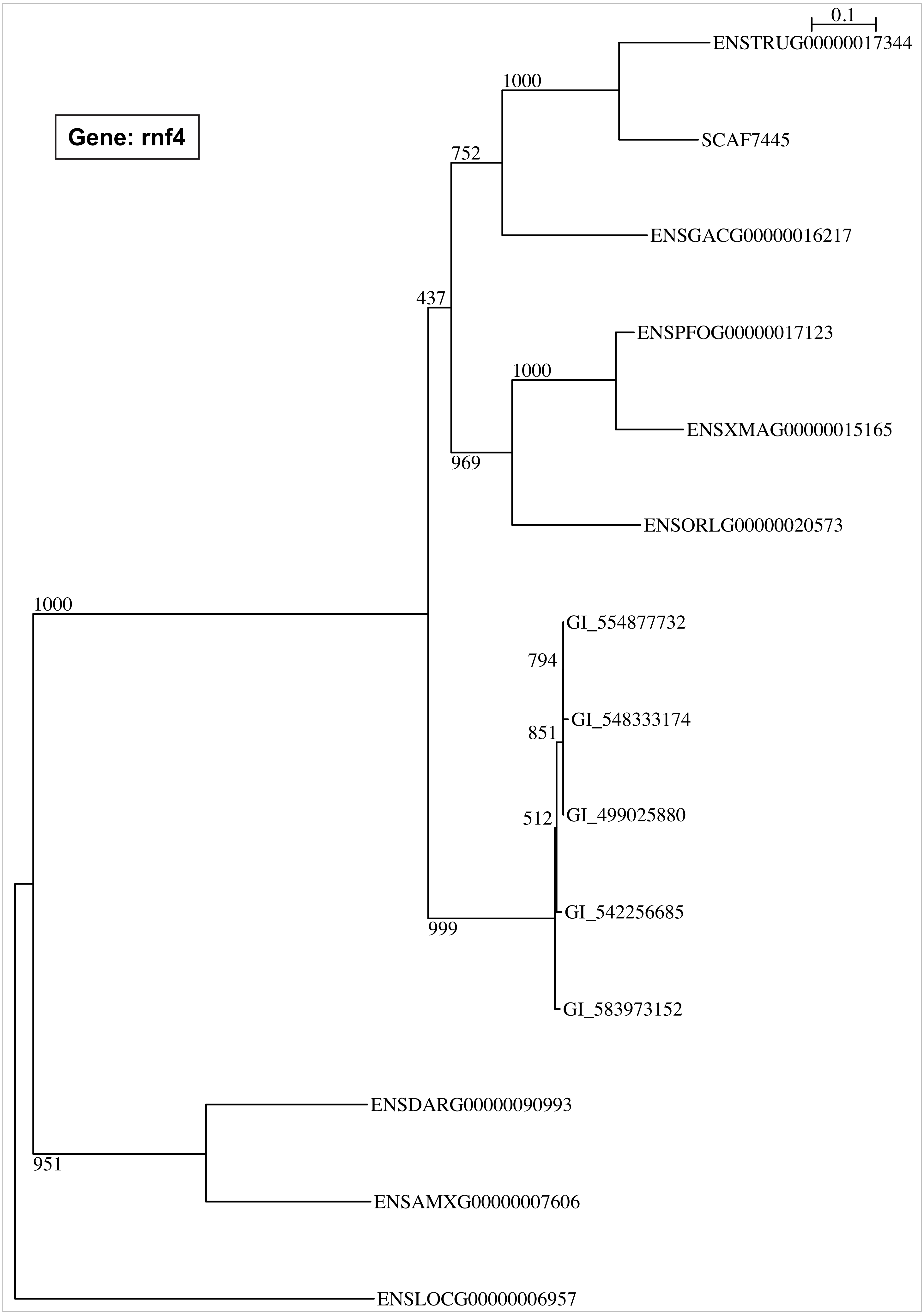
Gene: rnf4
0.1
ENSTRUG00000017344
SCAF7445
ENSGACG00000016217
ENSPFOG00000017123
ENSXMAG00000015165
ENSORLG00000020573
GI_554877732
GI_548333174
GI_499025880
GI_542256685
GI_583973152
ENSDARG00000090993
ENSAMXG00000007606
ENSLOCG00000006957
1000
752
437
1000
969
1000
794
851
512
999
951

Figure S1

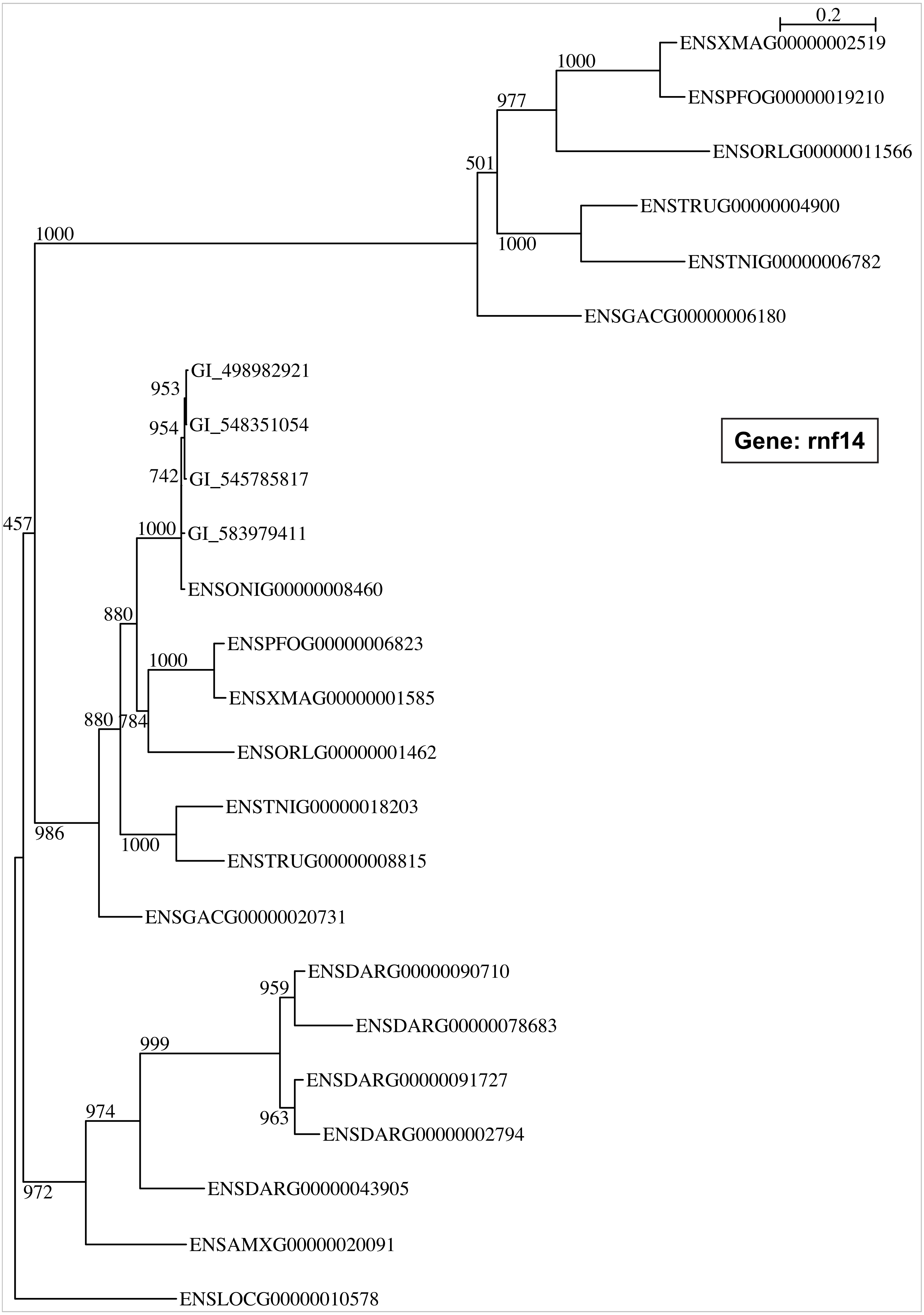
0.2
ENSXMAG00000002519
ENSPFOG00000019210
ENSORLG00000011566
ENSTRUG00000004900
ENSTNIG00000006782
ENSGACG00000006180
GI_498982921
GI_548351054
GI_545785817
GI_583979411
ENSONIG00000008460
ENSPFOG00000006823
ENSXMAG00000001585
ENSORLG00000001462
ENSTNIG00000018203
ENSTRUG00000008815
ENSGACG00000020731
ENSDARG00000090710
ENSDARG00000078683
ENSDARG00000091727
ENSDARG00000002794
ENSDARG00000043905
ENSAMXG00000020091
ENSLOCG00000010578
1000
977
501
1000
1000
953
954
742
1000
457
880
1000
880
784
986
1000
959
999
974
963
972
Gene: rnf14

Figure S1

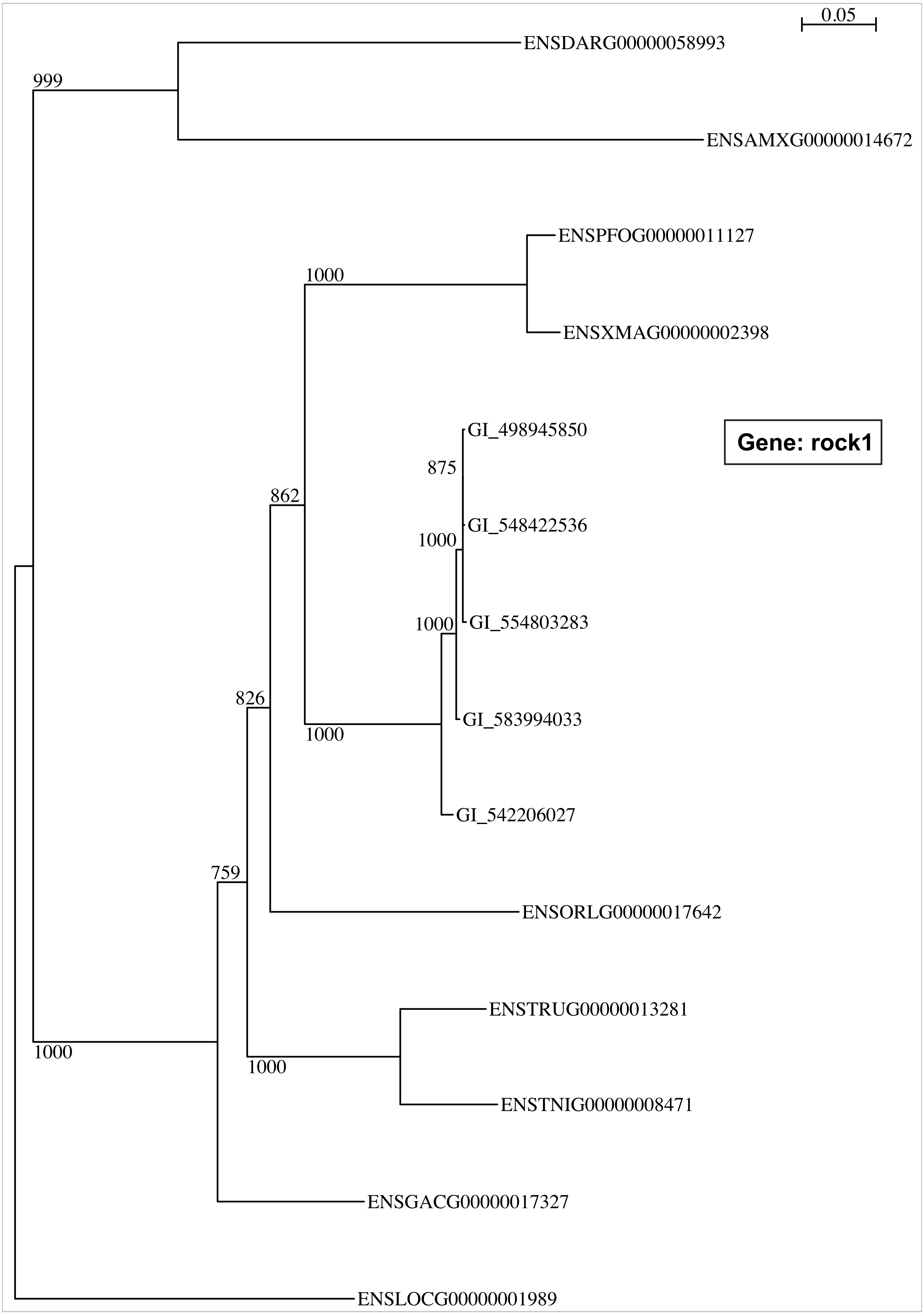
0.05
ENSDARG00000058993
999
ENSAMXG00000014672
ENSPFOG00000011127
1000
ENSXMAG00000002398
GI_498945850
Gene: rock1
875
862
GI_548422536
1000
1000
GI_554803283
826
GI_583994033
1000
GI_542206027
759
ENSORLG00000017642
ENSTRUG00000013281
1000
1000
ENSTNIG00000008471
ENSGACG00000017327
ENSLOCG00000001989

Figure S1

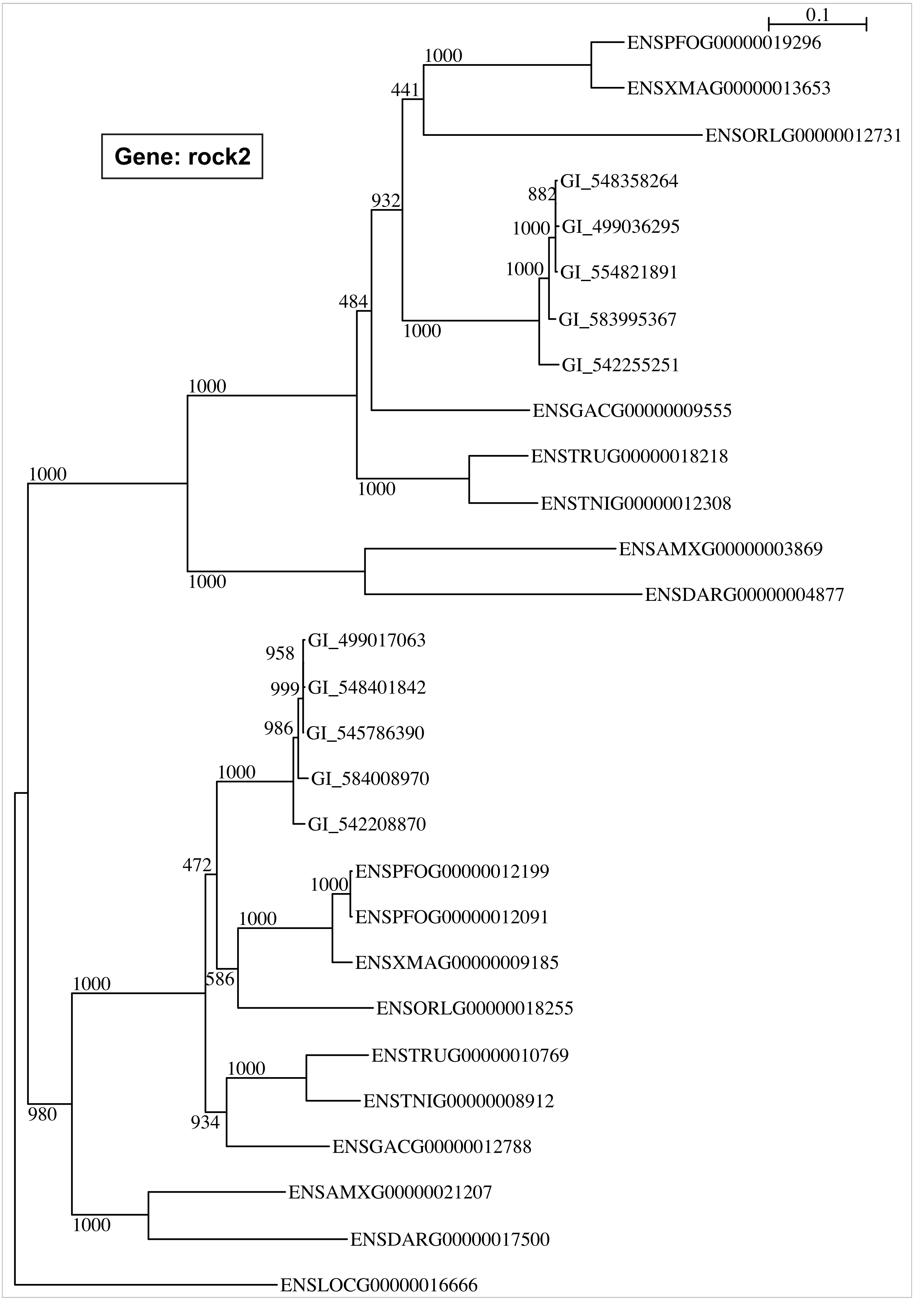

 T. Lorin, W. Salzburger & A. Böhne

Figure S1

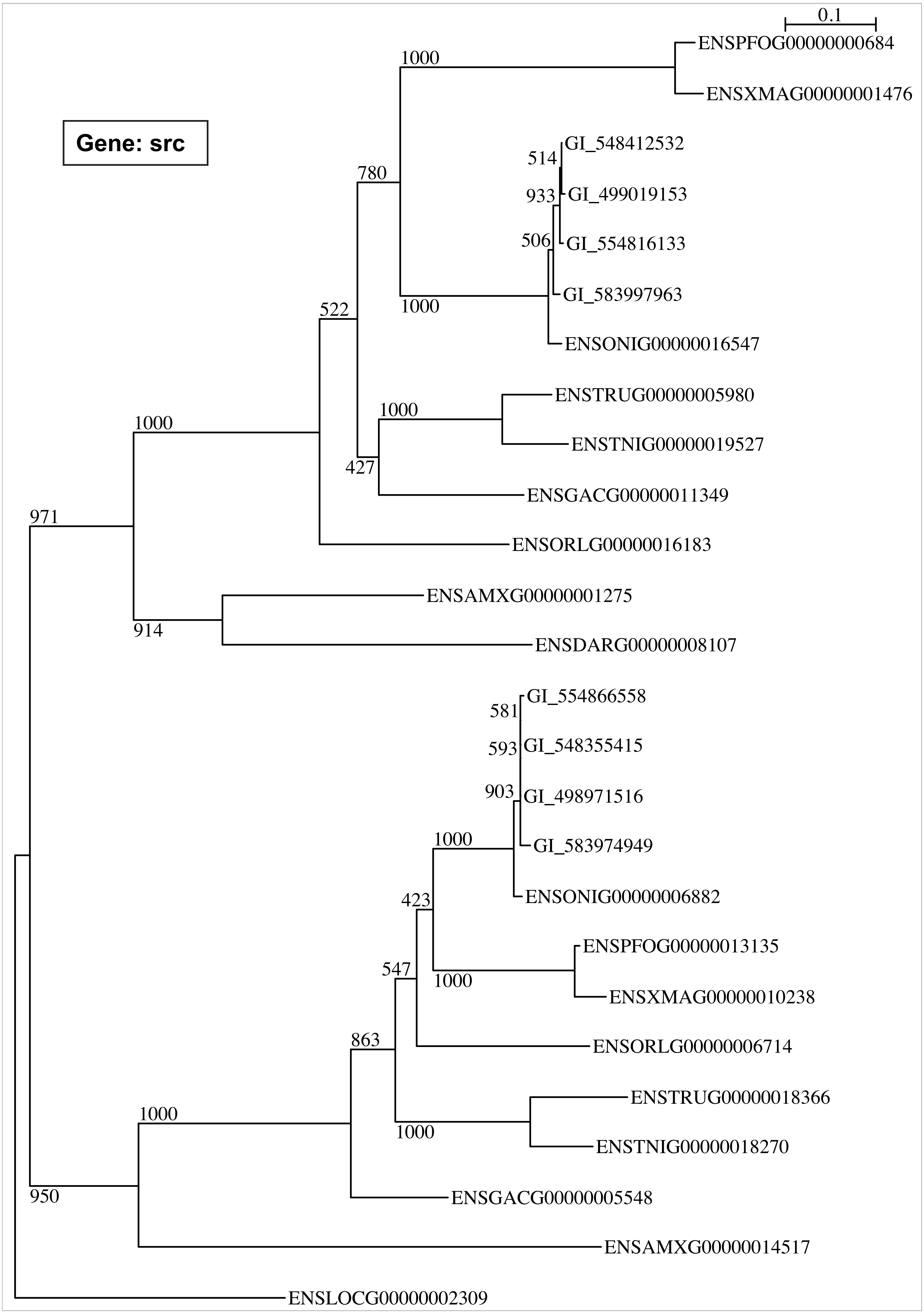

0.1
Gene: src
1000
ENSPFOG00000000684
ENSXMAG00000001476
514
GI_548412532
933
GI_499019153
506
GI_554816133
780
GI_583997963
522
1000
ENSONIG00000016547
ENSTRUG00000005980
1000
ENSTNIG00000019527
1000
427
ENSGACG00000011349
971
ENSORLG00000016183
ENSAMXG00000001275
914
ENSDARG00000008107
GI_554866558
581
593
GI_548355415
903
GI_498971516
1000
GI_583974949
423
ENSONIG00000006882
ENSPFOG00000013135
547
1000
ENSXMAG00000010238
863
ENSORLG00000006714
ENSTRUG00000018366
1000
1000
ENSTNIG00000018270
950
ENSGACG00000005548
ENSAMXG00000014517
ENSLOCG00000002309

Figure S1

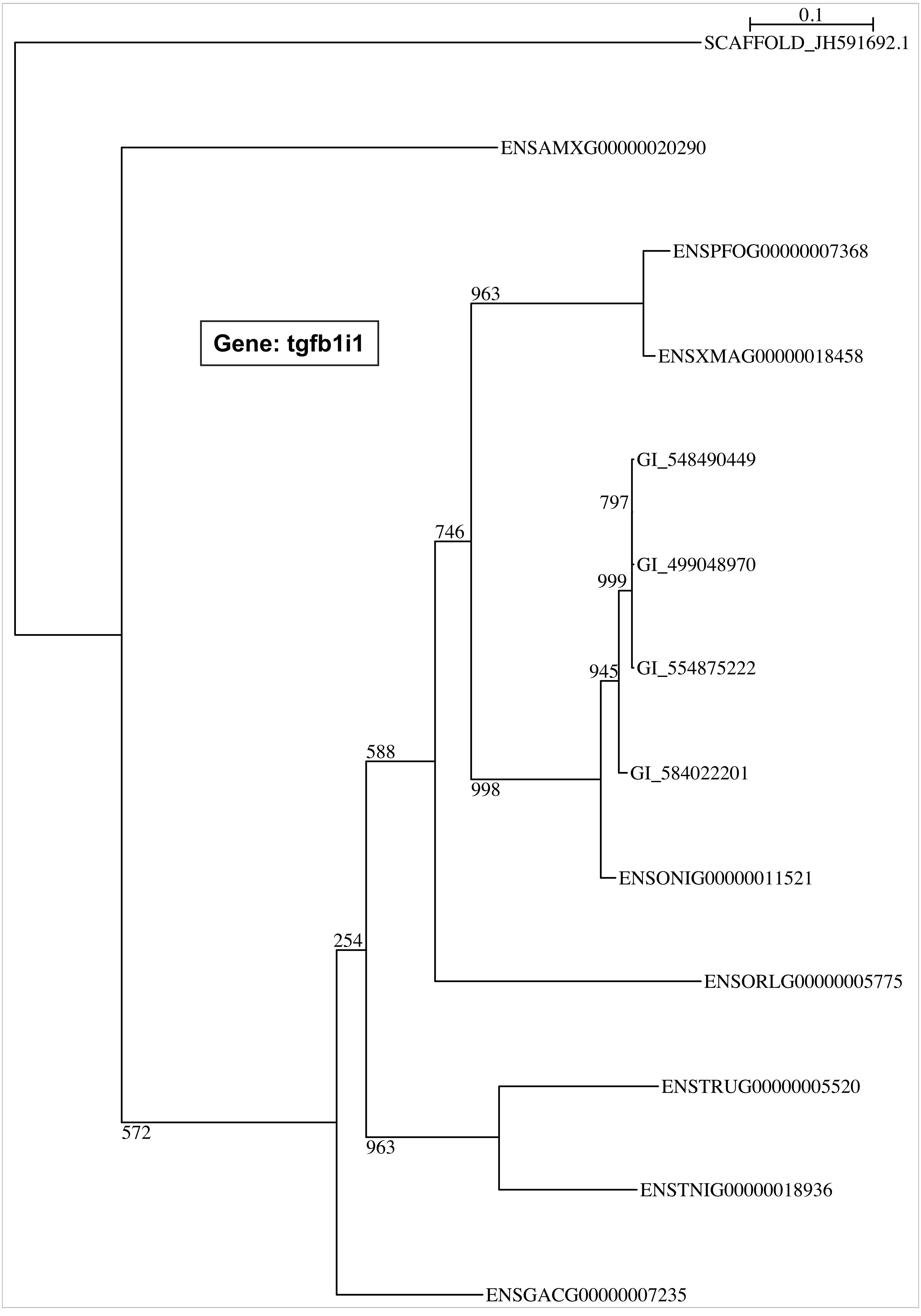
0.1
SCAFFOLD_JH591692.1
ENSAMXG00000020290
ENSPFOG00000007368
963
Gene: tgfb1i1
ENSXMAG00000018458
GI_548490449
797
746
GI_499048970
999
GI_554875222
945
588
GI_584022201
998
ENSONIG00000011521
254
ENSORLG00000005775
ENSTRUG00000005520
572
963
ENSTNIG00000018936
ENSGACG00000007235

Figure S1

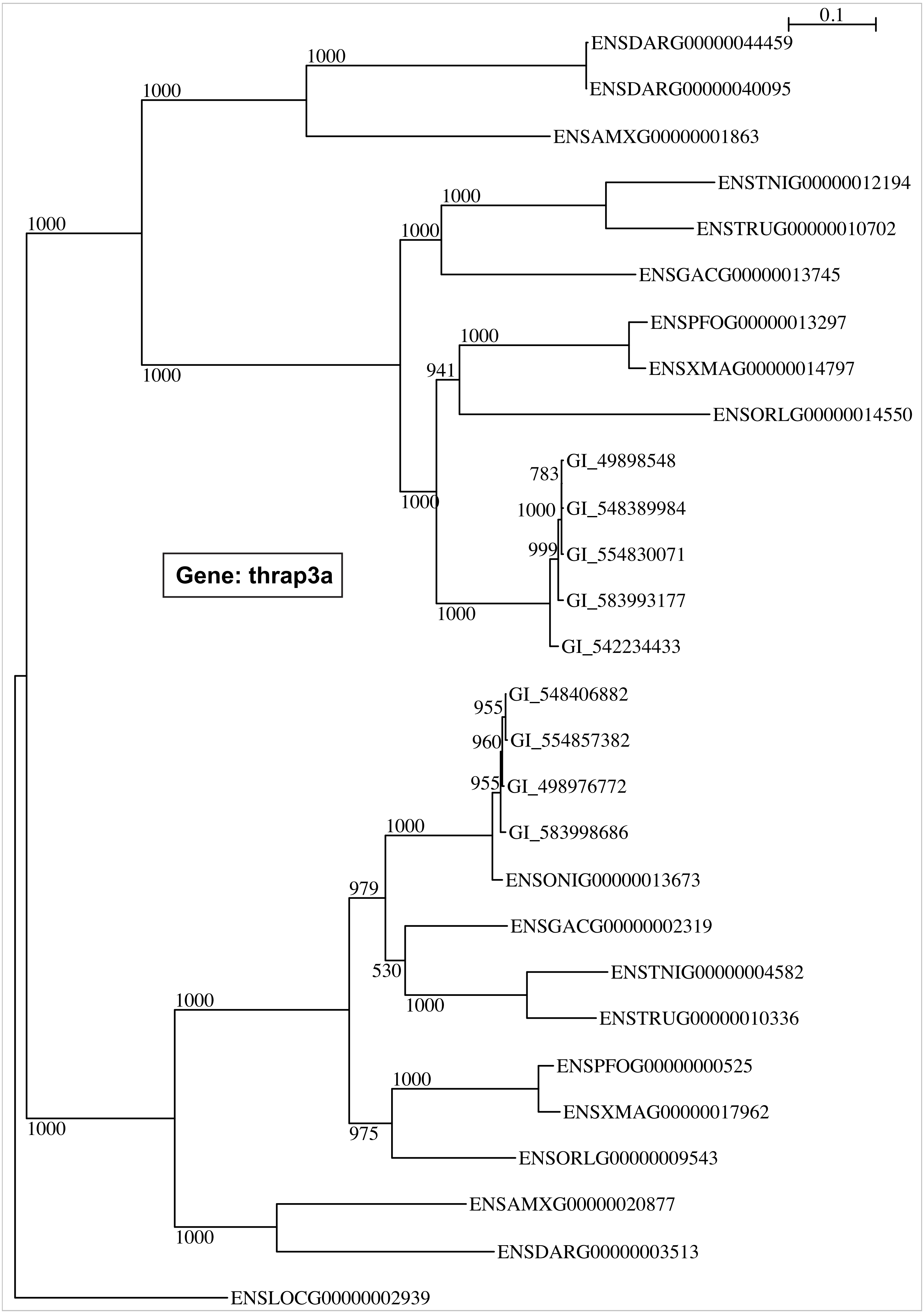
0.1
1000
1000
1000
1000
1000
1000
1000
1000
941
1000
783
1000
999
1000
955
960
955
1000
979
530
1000
1000
1000
1000
975
1000
ENSDARG00000044459
ENSDARG00000040095
ENSAMXG00000001863
ENSTNIG00000012194
ENSTRUG00000010702
ENSGACG00000013745
ENSPFOG00000013297
ENSXMAG00000014797
ENSORLG00000014550
GI_49898548
GI_548389984
GI_554830071
GI_583993177
GI_542234433
Gene: thrap3a
GI_548406882
GI_554857382
GI_498976772
GI_583998686
ENSONIG00000013673
ENSGACG00000002319
ENSTNIG00000004582
ENSTRUG00000010336
ENSPFOG00000000525
ENSXMAG00000017962
ENSORLG00000009543
ENSAMXG00000020877
ENSDARG00000003513
ENSLOCG00000002939

Figure S1

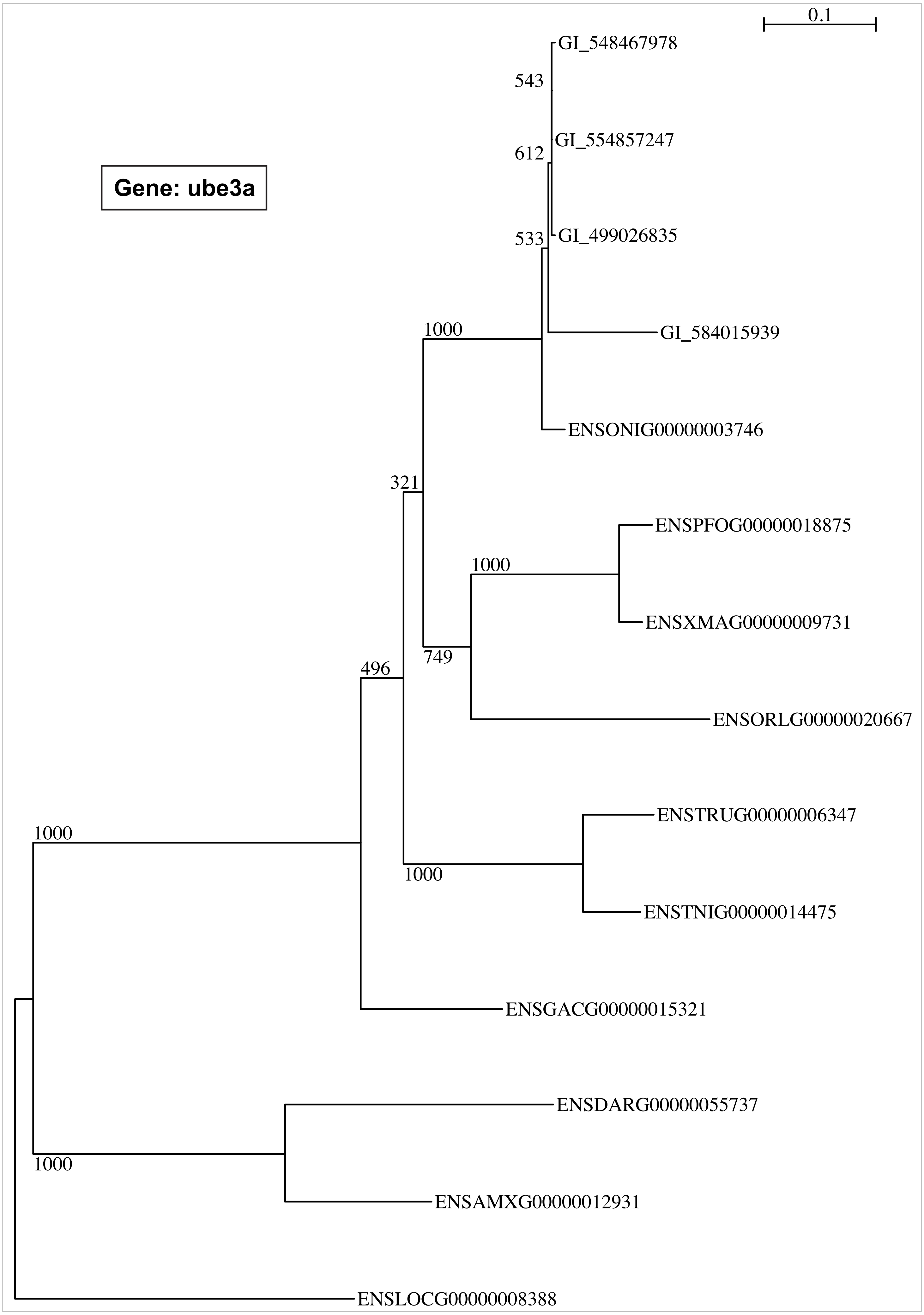
0.1
Gene: ube3a
GI_548467978
543
GI_554857247
612
GI_499026835
533
GI_584015939
1000
ENSONIG00000003746
321
ENSPFOG00000018875
1000
ENSXMAG00000009731
749
496
ENSORLG00000020667
ENSTRUG00000006347
1000
1000
ENSTNIG00000014475
ENSGACG00000015321
ENSDARG00000055737
1000
ENSAMXG00000012931
ENSLOCG00000008388
