# Supplementary material for: Evolutionary Fate of the Androgen Receptor−Signaling Pathway in Ray-Finned Fishes with a Special Focus on Cichlids
Source: G3 (Bethesda). 2015 Sep 1;5(11):2275–83. doi: 10.1534/g3.115.020685 (PMC4632047; doi:10.1534/g3.115.020685)
Supplement: Supporting Information [file supp_g3.115.020685_FigureS2.pdf]

**Figure S2**

Site-wise Ka/Ks estimates using Selecton under the M8 model allowing for positive selection. In case positive selected sites were detected, they were compared to the null model M8a. If the likelihood ratio test returned significant results for positive selection, these are indicated below the sequence.

**Figure S2**

**Gene: *akt1***

| | | | | |
|---|---|---|---|---|
| 1 | 11 | 21 | 31 | 41 |
| MNEVVIVKEG | WLHKRGEYIK | TWRPRYFLLK | SDGTFIGYKE | RPQDVDQLET |
| 51 | 61 | 71 | 81 | 91 |
| PLNNFSVAQC | QLMKTERPKP | NTFIIRCLQW | TTVIERTFHV | ETPEEREEWT |
| 101 | 111 | 121 | 131 | 141 |
| KTIQAVADSL | QKQEEEMMDC | RSGSPSDNIG | IEDMEVYLTK | PRLKVTMHDF |
| 151 | 161 | 171 | 181 | 191 |
| EYLKLLGKGT | FGKVILVKEK | ATGRYYAMKI | LKKEVIVAKD | EVAHTLTENR |
| 201 | 211 | 221 | 231 | 241 |
| VLQSSKHPFL | TGLKYSFQTH | DRLCFVMEYA | NGGELFFHLS | RERVFSEERA |
| 251 | 261 | 271 | 281 | 291 |
| RFYGAEIVSA | LDYLHSEKNV | VYRDLKLENL | MLDKDGHIKI | TDFGLCKEGI |
| 301 | 311 | 321 | 331 | 341 |
| KDGATMKTFC | GTPEYLAPEV | LEDNDYGRAV | DWWGLGVVMY | EMMCGRLPFY |
| 351 | 361 | 371 | 381 | 391 |
| NQDHEKLFEL | ILMEDIRFPR | TLAPEGQSLL | SGLLKKDPKQ | RLGGGPDDAK |
| 401 | 411 | 421 | 431 | 441 |
| EVMQHKFFAG | IVWQDVYEKK | LIPPFKPQVT | SETDTRYFDE | EFTAQTITIT |
| 451 | 461 | 471 | | |
| PPGQDDNMES | MDSERRPHFP | QFSYSASGTA | | |

**Legend:**

**The selection scale:**

1 2 3 4 5 6 7

Positive selection Purifying selection

**Figure S2**

**Gene: *ar***

```
1          11         21         31         41
MRQTNRQLPH RGLCIGTSHI SDTGVKEVQS ISFPRQTDVL ARSSHFAKRL
51         61         71         81         91
LGSRSEFSSV TKADDVEEGA YGFLPGLQGN TEQKSSIPIP PHHRKETKTH
101        111        121        131        141
GDALVESSNS DIASTTISET ARELCKAVSV SLGLNMDPNE MNDLGTSHDP
151        161        171        181        191
SLVSDRSQEN YLFQMHLADR SANGTGVTVT DYKCPIANDG PSVQSHENIR
201        211        221        231        241
MLKRVHANTT PEVVPSQHIS AAHSKQDGHE SRLNAELSNA TCEDSENHMN
251        261        271        281        291
MDGGHTVFCQ FDQSVRPNPA AYGQNDPLSV EQGNVASHGL YKCEAFPNAS
301        311        321        331        341
EENSEEVAAY PPEYRVRIKK EVSNEHADVW PVQYRYSDNG NTPYGLIRQF
351        361        371        381        391
THPQNTGQGT GSSFACNAYG EDGVSPEQWY SGGMVGRVPY TSSSCLKNEM
401        411        421        431        441
GDWMEVPYTD IRFESGRENL FPMEYFFPPQ RTCLICGDEA SGCHYGALTC
451        461        471        481        491
GSCKVFFKRA AEGKQKYLCA SRNDCTIDKL RRKNCPSCRL RKCFEAGMTL
501        511        521        531        541
GARKLKKIGQ LKGLEEAGNQ NPIETVQCMS PKPSITTHTQ PVFLNILESI
551        561        571        581        591
EPEVVNAGHD NAQPDSAACL LTSLNELGER QLVKVVKWAK GLPGFRSLHV
601        611        621        631        641
DDQMTVIQHA WMGVMVFALG WRSYKNVNAR MLYFAPDLVF NDHRMHISSM
651        661        671        681        691
YDHCIRMKRL AQEFAWLQIT QEEYLCMKAL LLFSIIPVEG LKSQTYFDEL
701        711        721        731        741
RTNYIHELDR LVSSGRKTSS NYSQRFFQLT QLMDSLQPIV RMLHQFTFEL
751        761        771        781
FVQAQSYPTK VNFPEMMSEI ISVQVPKMLA GMVKPILFHK
```

**Legend:**

**The selection scale:**

| 1 | 2 | 3 | 4 | 5 | 6 | 7 |
|---|---|---|---|---|---|---|

Positive selection — Purifying selection

**Figure S2**

**Gene: *arid1a***

```
1          11         21         31         41
MAAQVASVAT LNTSPPSELK KADRDPQEEP VPGEKQQENK EPGSESGSPG
51         61         71         81         91
QKELQDGADG GNAGGGGDPE MKNGNGNPSR VNNNNNQNDS GAPEGNNHPG
101        111        121        131        141
MAHHHPAAFP PPPYGYSPHY GRGPFHQHGG QQSPGMAAAA GPAVQPGSMM
151        161        171        181        191
DSYQPNSHDH GFPNHQFNNY SPFPNRTAYP GQGYTMNSPR NNQPPAAGGQ
201        211        221        231        241
PAKQQQQQPP PPAGGTTAMA VTYNNQRYTM GNPQPTSTPT LNQLLTSPSS
251        261        271        281        291
TRGYQNYPPS DYSNQEGANK GPVDVGSSSQ YGGHQGWQQR THHPPPMSPG
301        311        321        331        341
STGQPLSRNQ PSSPMDQMGK MRGQPYAAAN PYSQQPQQGP PPGSQQGASY
351        361        371        381        391
PGQGYGPPTP QRYPMGMQSR TPGGMGSMQY GQQMPPYGQQ GPAGYGQQGQ
401        411        421        431        441
ASYYSQQAQP PHAAQQQSPY PQQPQSQPGA QTPYSQPSAP LAQAQPPYSQ
451        461        471        481        491
PHQTPQSQAQ QQQQQQQQQP PPVPPQSQAP YSQSQAPQSA QPPYPQQQVP
501        511        521        531        541
PPPPQQQQQP PSQPPPPQPT PATQPQSQQP PGHAPPQQSQ AAPYSQAPPQ
551        561        571        581        591
QQQQQPSPYQ RFPPPPQELS QDSFSSQSSV PPSNPAMASS KSGSEDVNMQ
601        611        621        631        641
GRPSSLPDLS GSIDDLPTGT EGALSPGVST SGVSSSQGEQ SNPAQSPFSP
651        661        671        681        691
HTSPHLPGIR GPSPSPVGSP ASVTQSRSGP LSPAGVPGTQ MPPRPPSGQS
701        711        721        731        741
ESILHPSMNQ SAMGQDRVYM QRNPQMPAYG SPQPGSALSP RQSSGGQMHA
751        761        771        781        791
GMGPYQQNNS MGNYGPQGAQ YGPQGYPRQP NYTGMPNASY PGPGMGGSMN
801        811        821        831        841
PMPGQGGGPP YTGMPPGRMG PGQMGTRPYG PNMGPNMGSM PPQVGSGMCP
851        861        871        881        891
PPGGLNRKAQ EAAAAAMHAA VNSSHNRPPG YPNMPQSGMI PAGTPYGQSM
901        911        921        931        941
NSMPGMMNPQ GPPYPMGGNM PNNTAGMAPS PELMGLDVKL NQAQKMNNKA
951        961        971        981        991
DGTPKPETKS KKSSSSTTTN EKITKLYELG PEPERKMWVD RYLAFTEEKA
1001       1011       1021       1031       1041
```

**Figure S2**

```
MGMNNLPAVG RKPLDLFRLY VSVKEIGGLT QVNKNKKWRE LATNLNVGTS
1051       1061       1071       1081       1091
SSAASSLKKQ YIQCLYAFEC KIERGEDPPP DIFSAAEAKK NQPKIQPPSP
1101       1111       1121       1131       1141
AGSGSLQGPQ TPQSTSSSMA EGGDLKPPTP ASTPHSQMPP MPGVRSSVVS
1151       1161       1171       1181       1191
LQDPFADSSD PAFPRRNSMT PNSAYQQGMN TPDMMGRMPY EPNKDPFSAM
1201       1211       1221       1231       1241
RKGGEFMSPG QGPNSGMGEQ YNRAPPGSMG NMGMGQRQQY PYGPGYDRRQ
1251       1261       1271       1281       1291
EPGMGPEGSM GPGAPQPNLM PSNADTGMYS PSRYPPQQQR HDSYGNQYPG
1301       1311       1321       1331       1341
QGAPSGGPYP NQPPGMYAQQ QPNYKRPVDG GYGPPAKRHE GEMYNVPFSG
1351       1361       1371       1381       1391
QQQQQAQQQT APPAQQEMYS QYGNAYAGSE RRPPGPQNQF PFQFGRERVQ
1401       1411       1421       1431       1441
ATAGPNSQQS MPPQMMGSPM QSTPDGPQGS MWPNRNDMGY NFPNRQGPGA
1451       1461       1471       1481       1491
AAQGPGYHSM NRSEEMMPSD QRMNHEGQWP AHVNQRQPPY GPSGPVPPMT
1501       1511       1521       1531       1541
RPLQPNYQTP PAIQNHIPQV SSPAPMPRPI ESRTSPSKPF MHSGIKMQKA
1551       1561       1571       1581       1591
GPPVPASHIT PAPVQPPLIR RDITFPPGSI EATQPILKPR RRLTMKDIGT
1601       1611       1621       1631       1641
PEAWRVMMSL KSGLLAESTW ALDTINILLY DDNSIATFSL CQLPGFLELL
1651       1661       1671       1681       1691
VEYFRRCLIE IFGILKEYEV GDPGQRTLLD PEGLNSERDT GSEDEEQEPE
1701       1711       1721       1731       1741
EAEEMEEEDE DEDEEQPEAS EQQQQQQQTP QPPALEKQEG EQQNGERAGE
1751       1761       1771       1781       1791
QQEEQEGEAA VKDPSVLTLS QDTGTAQEKP KQASKFDKLP IKLVRKKDPF
1801       1811       1821       1831       1841
VVDCSDKLGR LQEFDSGLLH WRIGGGDTTE HIQTHFESKL DLLQARKRVP
1851       1861       1871       1881       1891
PASGGSAGRK KSPAGENVTE GVEKVKTSEE QPPAKSITAT IDDVLSARPG
1901       1911       1921       1931       1941
SMTVEAVRGT PESHKENSKF LFSINPAQSH RNIKILEDEP RSKDETPLST
1951       1961       1971       1981       1991
IADWQDSLAK RCICVSNIVR SLSFVPGNDL EMSKHPGLLL LLGRLILLHH
2001       2011       2021       2031       2041
EHPERKQAPL TYEKEEEEDE GVSCEKDEWW WDCLELLREN TLVTLANISG
2051       2061       2071       2081       2091
QLDLSIYPES ICLPLLDGLL HWAVCPSAEA QDPFPTLGVN GVLSPQRLVL
2101       2111       2121       2131       2141
ETLSKLSIQD NNVDLILATP PFSRLEKLYG TLVRLVGERK IPVCREMAVV
2151       2161       2171       2181       2191
```

**Figure S2**

LLANLAQGDS LAARAIAVQK GSIGNLLGFL EDSLAATQFQ QSQSSLLHMQ
2201 2211 2221 2231 2241
SAPFEPTSVD MMRRAARALH AMAKVEENHS EFTLYESRLL DISVSPLMNS
2251 2261
LVSHVICDVL FLIGQS

**Legend:**

**The selection scale:**

| 1 | 2 | 3 | 4 | 5 | 6 | 7 |
|---|---|---|---|---|---|---|

Positive selection Purifying selection

**Figure S2**

**Gene: *brca1***

```
1          11         21         31         41
MAAPTCKVED VKQVVSMIRR NLECPICLDL LRDPVSTRCD HQFCRMCMLK
51         61         71         81         91
HLSGSKRAGA QCPLCKAEVT KRSLQDSSRF KQLVEALQRT IHAFEQDTGT
101        111        121        131        141
KYFSEVLLEQ KNGLGGLTEA QAIGTKTRAE SSACERLGGR ITDSESQQEL
151        161        171        181        191
EAAISSAAAA KEGFAKLMDL DDSSYFANSG TAVAGSSEPT TQDRERSAAD
201        211        221        231        241
VTDPVIPVRL HMGNNRDLTS SREEEEHSFV SASKGENKTF DVSEKSKTNN
251        261        271        281        291
KVSSNKILEK RRKTSVQKVM DWLSRIDEKA CFPEEENRPA ETPSDADSLL
301        311        321        331        341
ATEKCDELVQ ECVESLKKTD RRSTLEDQVF GVVYKRARRT SAQLNKSFSS
351        361        371        381        391
DRKGSSKEHQ ANKKVCKRKS NELTPADFIK RPSPVAETSD ATSQPARSDA
401        411        421        431        441
GGRVRSESLH VLVSDGNQPE IEQRPSPSQR TARAETTQAG GSWKDEEEDL
451        461        471        481        491
QPDRQERKGK KSKPNTKDNR KRNALTRKKA IKAAKPLDLV SCQQNNTGQD
501        511        521        531        541
ADSAGPWKPT INETEAQIES YPSSEEPAGR AGEPHATRRS RRLQLLTEKV
551        561        571        581        591
RCTGRRLRTE DSELERDPQA PDQSENDQAG MLTCKGVEQL CESSEKENET
601        611        621        631        641
TEVLKVSEGY EDIQQQSGHI VVESSLVSII RNAESPCNSN KAFKGDQLVP
651        661        671        681        691
SALDISEGPD TGSDVAVMDE LLPGELTEGK NDSELETEQI LKTFKISKRR
701        711        721        731        741
SFNLLPSPEY PSQESEFCDN KASEKNENPE EEKGQVNVAV ERTSLQSEHV
751        761        771        781        791
ETENSGCTDI IPPTKPASPS CRNVDIKSAD KISDGSTSTF SSICTDSQRP
801        811        821        831        841
RDRNGRRKKT KMDESTLGSV RGRKKNLNLS SIHFSQVPPN TEQLVCASLL
851        861        871        881        891
NRQIPIDGLP QENESLVENC KVKSTMKESF VGNGEQSDFV QSFPLARKEL
901        911        921        931        941
STGPNISQES SMTPDGLLPL RAVASDRADN EEQCLPGEGK EQLSQWSLNS
951        961        971        981        991
NPRKRMRARM LESSDSDSNT EEDLPSLAEL LGPKQPATNP QEDSNQWNPL
1001       1011       1021       1031       1041
```

**Figure S2**

QNRDLEASPS STVESSVVLP VQQSVPASQG SVDLFSTQSD SPSHRIEQID

1051 1061 1071 1081 1091

LVETESSQSE ILTTQQKDTI QKDLRRIEEE MAVLEAALKG QPDYPKSSLQ

1101 1111 1121 1131 1141

TVVAQQSTET QPVSHEFRAL PCHVQLNKSV KRNLIKPLEK HANENTNIKP

1151 1161 1171 1181 1191

GKHIFYPGFS VFILSSCFEG LLSVAKLGIG TTKQSGSSGA CDASGPIPNP

1201 1211 1221 1231 1241

RVSADRGAAR GRMVLVGSGL DRSELIMVQK FARKMDGTLA AQVTPGTTHV

1251 1261 1271 1281 1291

IMKTDPELVC ERTLKYFLGI AGRMWVVSFL WIRECFRQGQ LLDEAAFEVK

1301 1311 1321 1331 1341

GDVINGRNHQ GPRRARTATR EELLLSNYEI CCHGSFTDMT TGQLEWMVEL

1351 1361 1371 1381 1391

CGATVAKEPF LFTYNPELIQ VVVVQPEAEE SQTDYKALQR KYSARVVTRE

1401 1411 1421

WVLDSVALYS CQALDSYLVK PLS

**Legend:**

**The selection scale:**

1 2 3 4 5 6 7

Positive selection Purifying selection

**Figure S2**

## Gene: *cav1*

| 1 | 11 | 21 | 31 | 41 |
|---|---|---|---|---|
| MTGGIKDEES | EGFLHSPFIR | KQGNIYKPNN | KSMDNDSMNE | KAMHDVHTKE |
| **51** | **61** | **71** | **81** | **91** |
| IDLVNRDPKH | LNDDVVKVDF | EDVIAEPEGT | HSFDGIWKAS | FTTFTVTKYW |
| **101** | **111** | **121** | **131** | **141** |
| CYRLLTALVG | IPLALIWGLF | FAILSFLHIW | AVVPCVKSYL | IEIQCVSRVY |
| **151** | **161** | **171** | | |
| SICIHTFCDP | LFEAMGKVFS | GVRVSMTKEV | | |

**Legend:**

**The selection scale:**

| 1 | 2 | 3 | 4 | 5 | 6 | 7 |
|---|---|---|---|---|---|---|

Positive selection — Purifying selection

**Figure S2**

**Gene: *ccne1***

| 1 | 11 | 21 | 31 | 41 |
|---|---|---|---|---|
| MPRKNLKTGL | QTEANDEVTK | ERSTTRSRKR | KADVAIHLQD | PDEEVIEMTN |
| 51 | 61 | 71 | 81 | 91 |
| KKQYDTQTSW | NPATGCTSPC | RLICTPDKEV | DEPVALNDVG | CAQYMFKNIF |
| 101 | 111 | 121 | 131 | 141 |
| ITPTRSSPLP | VLCWANKDDV | WNNLLQKDKV | YVRDNRFMER | HPHLQPKMRA |
| 151 | 161 | 171 | 181 | 191 |
| ILLDWLMEVC | EVYKLHRETF | YLAQDFFDRF | MATQMNVVKT | RLQLIGISSL |
| 201 | 211 | 221 | 231 | 241 |
| FIAAKLEEIY | PPKLHQFAYV | TDGACTEDEI | LDMELIIMKE | LKWSLSPLTA |
| 251 | 261 | 271 | 281 | 291 |
| MSWLNIYMQV | AYLKESSEML | IPQYPQTTFV | QIAELLDLCA | LDVRCLEFSY |
| 301 | 311 | 321 | 331 | 341 |
| GVLAASALFH | FSSVELVQKV | SGYQLQDLEP | CVKWMVPFAM | SIREVGSSEL |
| 351 | 361 | 371 | 381 | 391 |
| KKFKGIAADD | LHNIQTHAAL | LEWLEKAYAY | EAMGLDQNQR | SPVPLSVLTP |
| 401 | 411 | | | |
| PPSSEKTGVS | ES | | | |

**Legend:**

**The selection scale:**

| 1 | 2 | 3 | 4 | 5 | 6 | 7 |
|---|---|---|---|---|---|---|

Positive selection    Purifying selection

**Figure S2**

**Gene: *cdc42***

```
1          11         21         31         41
MQTIKCVVVG DGAVGKTCLL ISYTTNKFPS EYVPTVFDNY AVTVMIGGEP
51         61         71         81         91
YTLGLFDTAG QEDYDRLRPL SYPQTDVFLV CFSVVSPSSF ENVKEKWVPE
101        111        121        131        141
ITHHCPKTPF LLVGTQIDLR DDPSTIEKLA KNKQKPITPE TAEKLARDLK
151        161        171        181
AVKYVECSAL TQKGLKNVFD EAILAALEPP EPKKKRKCKL L
```

**Legend:**

**The selection scale:**

| 1 | 2 | 3 | 4 | 5 | 6 | 7 |
|---|---|---|---|---|---|---|

Positive selection — Purifying selection

Figure S2

**Gene: *cdk7***

```
1          11         21         31         41
MAVDVKSRAK RYEKLDFLGE GQFATVYKAR DKNTNTIVAI KKIKVGHRAE
51         61         71         81         91
AKDGINRTAL REIKLLQELS HPNIIGLLDA FGHKSNISLV FDYMETDLEV
101        111        121        131        141
IIKDTSLVLT PANIKAYILM TLQGLEYMHQ HWILHRDLKP NNLLLDENGV
151        161        171        181        191
LKLADFGLAK AFGSPNRVYT HQVVTRWYRS PELLFGARMY GVGVDMWAVG
201        211        221        231        241
CILAELLLRV PFLPGDSDLD QLTKIFEALG TPTEETWPGL TSLPDYISFK
251        261        271        281        291
MFPGTPLEHI FSAAGDDLLD LLRGLFTYNP CTRITATQAL KKRYFSNRPG
301        311        321        331        341
PTPGPQLPRP NCSVEALKEQ ENFKIGVKRK RDGTEQGSLK KKLIF
```

**Legend:**

**The selection scale:**

| 1 | 2 | 3 | 4 | 5 | 6 | 7 |
|---|---|---|---|---|---|---|

Positive selection Purifying selection

**Figure S2**

## Gene: *cfl1l*

| 1 | 11 | 21 | 31 | 41 |
|---|---|---|---|---|
| MASGVAVHDD | VVNIFNAMKV | RKAGEAEQDR | LKAILFRLSN | DEKQIIVDEG |
| 51 | 61 | 71 | 81 | 91 |
| SELHVRDLQP | GEDIFKRVVG | LLPEKDCRYA | LYDSSYETKD | SKKEDLVFIM |
| 101 | 111 | 121 | 131 | 141 |
| WAPENAPLKR | KMIYASSKNA | LRSKLTGIKF | EWQINEMGEI | KDPSTLVEKF |
| 151 | 161 | | | |
| GGKGLVVSVE | GKPI | | | |

**Legend:**

**The selection scale:**

| 1 | 2 | 3 | 4 | 5 | 6 | 7 |
|---|---|---|---|---|---|---|

Positive selection    Purifying selection

**Figure S2**

## Gene: *ctnnb1*

| | | | | |
|---|---|---|---|---|
| 1 | 11 | 21 | 31 | 41 |
| MATQADLMEL | DMAMEPDRKA | AVSHWQQQSY | LDSGIHSGAT | TTAPSLSGKG |
| 51 | 61 | 71 | 81 | 91 |
| NPEDDDVDNQ | VLYEWETGFS | QSFTQDQVAD | IDGQYAMTRA | QRVRAAMFPE |
| 101 | 111 | 121 | 131 | 141 |
| TLDEGMQIPS | TQFDGAHPTN | VQRLAEPSQM | LKHAVVNLIN | YQDDAELATR |
| 151 | 161 | 171 | 181 | 191 |
| AIPELTKLLN | DEDQVVVNKA | AVMVHQLSKK | EASRHAIMRS | PQMVSAIVRT |
| 201 | 211 | 221 | 231 | 241 |
| MQNTNDVETA | RCTAGTLHNL | SHHREGLLAI | FKSGGIPALV | KMLGSPVDSV |
| 251 | 261 | 271 | 281 | 291 |
| LFYAITTLHN | LLLHQEGAKM | AVRLAGGLQK | MVALLNKTNV | KFLAITTDCL |
| 301 | 311 | 321 | 331 | 341 |
| QILAYGNQES | KLIILASGGP | QALVNIMRTY | TYEKLLWTTS | RVLKVLSVCS |
| 351 | 361 | 371 | 381 | 391 |
| SNKPAIVEAG | GMQALGLHLT | DPSQRLVQNC | LWTLRNLSDA | ATKQEGMEGL |
| 401 | 411 | 421 | 431 | 441 |
| LGTLVQLLGS | DDINVVTCAA | GILSNLTCNN | YKNKMMVCQV | GGIEALVRTV |
| 451 | 461 | 471 | 481 | 491 |
| LRAGDREDIT | EPAICALRHL | TSRHQDAEMA | QNAVRLHYGL | PVVVKLLHPP |
| 501 | 511 | 521 | 531 | 541 |
| SHWPLIKATV | GLIRNLALCP | ANHAPLREQG | AIPRLVQLLV | RAHQDTQRRT |
| 551 | 561 | 571 | 581 | 591 |
| SMGGTQQQFV | EGVRMEEIVE | GCTGALHILA | RDVHNRIVIR | GLNTIPLFVQ |
| 601 | 611 | 621 | 631 | 641 |
| LLYSPIENIQ | RVAAGVLCEL | AQDKEAAEAI | EAEGATAPLT | ELLHSRNEGV |
| 651 | 661 | 671 | 681 | 691 |
| ATYAAAVLFR | MSEDKPQDYK | KRLSVELTSS | LFRTEPMTWN | ETGDLGLDIG |
| 701 | 711 | 721 | 731 | 741 |
| AQGEPLGYRQ | DDPSYRSFHS | GGYGQDAMGM | DPMMDHDMGG | HHPGAEYPVD |
| 751 | 761 | 771 | | |
| GLPDLGHAQD | LIEGLPPGDS | NQLAWFDTDL | | |

**Legend:**

**The selection scale:**

| 1 | 2 | 3 | 4 | 5 | 6 | 7 |
|---|---|---|---|---|---|---|

Positive selection Purifying selection

**Figure S2**

## Gene: *daxx*

1 XXXXXXXXXX 11 XXXXXXXXXX 21 XXXXXXXXXX 31 XXXXXXXXXX 41 XXXXXXXXXX

51 XXXXXXXXXX 61 XXXXXXXXXX 71 XXXXXXXXXX 81 XXXXXXXXXX 91 XXXXXXXXXX

101 XXXXXXXXXX 111 XXXXXXXXXX 121 XXXXXXXXXX 131 XXXXXXXXXX 141 XXXXXXXXXX

151 XXXXXXXXXX 161 XXXXXXXXXX 171 XXXXXXXXXX 181 XXXXXXXXXX 191 XXXXXXXXXX

201 XXXXXXXXXX 211 XXXXXXXXXX 221 XXXXXXXXMV 231 TDSSLSPLPP 241 AQIDKFINSQ

251 ESRLCPPDYS 261 DILRVVRRAD 271 ERHGLGLSRR 281 QLTQIAQDAF 291 RDTGNSLQER

301 RHLDMVYNFG 311 SHLTDGYQPA 321 TDPALADPTL 331 DRRLRTNRTV 341 ALISLDDVIX

351 XXXXXXXXXX 361 XXXXXXXXXX 371 XXXXXXXXXX 381 XXXXXXXXXX 391 XXXXXXXXXX

401 XXXXXXXXXX 411 XXXXXXXXXX 421 XXXXXXXXXX 431 XXXXXXXXXX 441 XXXXXXXXXX

451 XXXXXXXXXX 461 XXXXXXXXXX 471 XXXXXXXXXX 481 XXXXXXXXXX 491 XXXXXXXXXX

501 XXXXXXXXXX 511 XXXXXXXXXX 521 XXXXXXXXXX 531 XXXXXXXXXX 541 XXXXXXXXXX

551 XXXXXXXXXX 561 XXXXXXXXXX 571 XXXXXXXXXX 581 XXXXXXXXXX 591 XXXXXXXXXX

601 XXXXXXXXXX 611 XXXXXXXXXX 621 XXXXXXXXXX 631 XXXXXXXXXX 641 XXXXXXXXXX

651 XXXXXXXXXX 661 XXXXXXXXXX 671 XXXXXXXXXX 681 XXXXXXXX

**Legend:**

**The selection scale:**

| 1 | 2 | 3 | 4 | 5 | 6 | 7 |
|---|---|---|---|---|---|---|

Positive selection — Purifying selection

**Figure S2**

**Gene: *dnaja1***

| 1 | 11 | 21 | 31 | 41 |
|---|---|---|---|---|
| MVKETGYYDV | LGVKPNASAD | ELKKAYRKLA | LKYHPDKNPN | EGEKFKQISQ |
| 51 | 61 | 71 | 81 | 91 |
| AYEVLSDSKK | RELYDRGGEQ | AIKEGGMGGG | GFGSPMDIFD | MFFGGGGRMH |
| 101 | 111 | 121 | 131 | 141 |
| RERRGKNVVH | QLTVSLEDLY | NGATRKLAVQ | KNVICDRCEG | RGGRRGVVEC |
| 151 | 161 | 171 | 181 | 191 |
| CPMCRGSGMQ | VRLHQIGPGM | VQQISTICVG | CQGQGQRISQ | KDRCKACNAR |
| 201 | 211 | 221 | 231 | 241 |
| KIVRQKKILE | VHIDKGMKDG | QKITFHGEGD | QEPGLEPGDI | IIVLDQKVHP |
| 251 | 261 | 271 | 281 | 291 |
| VYTRQGEDLI | MNLEIKLVEA | LCGFQKPIKT | LDDRILLLTS | HPGEVIRPGD |
| 301 | 311 | 321 | 331 | 341 |
| RKCIMNEGMP | FHRRPFEKGR | LIVLFTVNFP | EENFLPLDKL | TQLERYLPER |
| 351 | 361 | 371 | 381 | 391 |
| EETDDTENMD | DLYIYADLED | CDLSQERPHF | DDDEYHSSGG | VQCQTS |

**Legend:**

**The selection scale:**

| 1 | 2 | 3 | 4 | 5 | 6 | 7 |
|---|---|---|---|---|---|---|

Positive selection    Purifying selection

**Figure S2**

## Gene: *egfr*

```
1          11         21         31         41
MQFEIAIVLS SLLTLGCCSI QDKRVCQGLS NKLTLLGTSQ DHYNNIVRMY
51         61         71         81         91
SNCTVVMENL EITYVEKDYD LSFLKSIREV GGYVLIALNK VSRIPIDNLR
101        111        121        131        141
IIRGHILYQN SYALAVLSNF DKQSSVNKNQ SSNVGLQELS LKSLTEILKG
151        161        171        181        191
GVKFTGNPFL CNAETIQWYD IVDKSKNPEM FLIFANNTLN CKKCDPNCFN
201        211        221        231        241
GSCWAPGPSN CQTLTKLNCA EQCSHRCKGP KPSDCCNEHC AAGCTGPRST
251        261        271        281        291
DCLACRDFQD EGTCKQVCPQ LMLYNPNTHQ LEMNPDGKYS FGATCVKSCP
301        311        321        331        341
HNYVVTDHGS CVRTCTANTH EVDENGVRKC KKCEGPCPKA CNGLGMGNLV
351        361        371        381        391
NVLSINASNI DSFRNCTKIN GDVSILPVAF RGDSYTKTPV LDPSKLDVFK
401        411        421        431        441
TVKEITGFLL IQAWPENMTS LSPLENLEII RGRTKQHGTV SVAAVNIDIT
451        461        471        481        491
SLGLRSLKEI SDGDVVIRGN PHLCYTNADQ WKRLFKLEKQ NARVSENADP
501        511        521        531        541
TECSALTQTC DELCTSEGCW GPGPSMCFSC QHFMRQQQCV NACNVLEGLP
551        561        571        581        591
REFIMDKKCI ECDPECMPQN GTQTCTGSGP DKCAECAHFK DGPHCVHKCP
601        611        621        631        641
SGIPGENDTF IWKYADEKKV CQLCHPNCTQ GCTGPGLAGC DHQTSQLSSI
651        661        671        681        691
AAGVVGGLLV TVIIALAIFI LMRRRYIKRK RTLRRLLQER ELVEPLTPSG
701        711        721        731        741
EAPNQALLRI LKETEFKKIQ VLGSGAFGTV YKGLWIPEGE DVKIPVAIKV
751        761        771        781        791
LREATSPKAN KEILDEAYVM ASVNNPHVCR LLGICLTSTV QLITQLMPYG
801        811        821        831        841
CLLDYVKEHK DNIGSQYLLN WCVQIAKGMN YLEERHLVHR DLAARNVLVK
851        861        871        881        891
TPQHVKITDF GLAKLLSADE KEYHADGGKV PIKWMALESI LHRIYTHQSD
901        911        921        931        941
VWSYGVTVWE LMTFGSKPYD GIPASEIAGI LEKGERLPQP PICTIDVYMI
951        961        971        981        991
MVKCWMIDAD SRPRFRELIA EFSKMARDPP RYLVIQGDER MHLPSPTDSK
1001       1011       1021       1031       1041
```

**Figure S2**

FYRTLIDGDE MDDVVDADEY LVPYNGFFNS PSTSRMQLLH SMSLNSSMGS

1051 1061 1071 1081 1091

CTIRNGQGYP MREDSFIQRY ITDPTDGAFE KDFQPVPEYV NQNGNPVSDV

1101 1111 1121 1131 1141

MNPIYQDSVV PISAVTRAQN ADSQYLNSFS NTADFHEYLN TVQPALPRPL

1151 1161 1171 1181 1191

FDNPHYCDQK VTHQTSLDNP DYQQDFFPPK SKVNGHLPAA ENTEYLALSA

1201

QAEVH

**Legend:**

**The selection scale:**

| 1 | 2 | 3 | 4 | 5 | 6 | 7 |
|---|---|---|---|---|---|---|

Positive selection Purifying selection

**Figure S2**

**Gene: *fhl2***

| | | | | |
|---|---|---|---|---|
| 1 | 11 | 21 | 31 | 41 |
| MTERFDCHYC | KESLFGKKYI | LREENPYCVK | CYESLYSNAC | EECKKPIGCN |
| 51 | 61 | 71 | 81 | 91 |
| SKDLSYKDRH | WHEDCFHCFQ | CKRSLVDKPF | SAKDELLLCT | ECYSNEYSSK |
| 101 | 111 | 121 | 131 | 141 |
| CHECKKTIMP | GTRKMEHKGN | SWHETCFTCH | RCQQPIGTKS | FIPKDAHNYC |
| 151 | 161 | 171 | 181 | 191 |
| VPCYEKQFAM | QCVQCKKPIT | SGGVTYHEQP | WHKDCFLCTG | CKKQLSGQRF |
| 201 | 211 | 221 | 231 | 241 |
| TSRDDFAYCL | NCFCNLYAKK | CAGCTNPISG | LGGSKYISFE | ERQWHNDCFN |
| 251 | 261 | 271 | | |
| CKKCSVSLVG | RGFLTERDDI | LCPECGKDI | | |

**Legend:**

**The selection scale:**

| 1 | 2 | 3 | 4 | 5 | 6 | 7 |
|---|---|---|---|---|---|---|

Positive selection Purifying selection

 

**Figure S2**

**Gene: *fkbp4***

| | | | | |
|---|---|---|---|---|
| 1 | 11 | 21 | 31 | 41 |
| MTAEEVNAEG | QQTTQMEGED | ITPKKDGGVL | KSIKKEGTGD | ENPMIGDKVF |
| 51 | 61 | 71 | 81 | 91 |
| VHYVGTLLDG | TKFDSSRDRS | EKFSFDLGKG | QVIKAWDIGV | ATMKIGEVCQ |
| 101 | 111 | 121 | 131 | 141 |
| LICKPEYAYG | SAGSPPKIPP | NSTLVFEIEL | FGFQGEDLTD | DEDGGIIRRI |
| 151 | 161 | 171 | 181 | 191 |
| ATKGEGYSKA | NEGATVEVSL | EGTFEGRVFD | QRELKFEIGD | GESLGIPPGV |
| 201 | 211 | 221 | 231 | 241 |
| EKALQSMEKG | EESLITLSPK | YGFGTAGNSQ | FNIPPDAMLN | YKIKLISFEK |
| 251 | 261 | 271 | 281 | 291 |
| AKESWEMNTD | EKLEQSVIVK | EKGTQYFKEG | KYKQASVQYK | KIVSWLEHES |
| 301 | 311 | 321 | 331 | 341 |
| GLSEEYENKA | KALRLAAHLN | LAMCYLKVQE | HSQAVENCNK | ALELDSSNEK |
| 351 | 361 | 371 | 381 | 391 |
| ALFRRGEALF | AMKEFEQARD | DFQKVVQLYP | SNKAAKTQVV | VCQRQIKEQH |
| 401 | 411 | 421 | 431 | 441 |
| EKDKRLYANM | FQKFAERDAK | KEAEKQKDTK | KQENTEGQNG | DEMDIDQNET |
| 451 | | | | |
| QEQTAA | | | | |

**Legend:**

**The selection scale:**

1 2 3 4 5 6 7

Positive selection — Purifying selection

Likelihood ratio test between the null model (no positive selection) and the alternative model (enabling positive selection) shows a significance level of: 0.05

 

**Figure S2**

**Gene: *flna***

```
1          11         21         31         41
MSSPHPRLNQ SSAPSSAAVS NAALADKDAD MPATEKDLAE DAPWKKIQQN
51         61         71         81         91
TFTRWCNEHL KCVNKRIANL QTDLSDGLRL IGLLEVLSQK KMFRKYNQRP
101        111        121        131        141
TFRQMQLENV SVALEFLDKE NIKLVSIDSK AIVDGNLKLI LGLIWTLILH
151        161        171        181        191
YSISMPMWDE EEDDDVAKQK TPKQRLLGWI QNKLPQLPIT NFSRDWQSGK
201        211        221        231        241
ALGALVDSCA PGLCPDWDSW DQTKPVDNAR EAMQQADDWL GIPQVITPEE
251        261        271        281        291
IVDPNVDEHS VMTYLSQFPK AKLKPGAPLR PKLNPKKARA YGPGIEPTGN
301        311        321        331        341
VVMKKAEFTV ETISAGMGEV LVYVEDPAGH REEAKVTANN DKNRTYSVVY
351        361        371        381        391
IPKVTGTHKV TVLFAGLHIS KSPFDVDVGM SQGDASKVTA QGPGLEPSGN
401        411        421        431        441
IANKTTYFDV YTAGAGIGEV GVSIVDPTGK KDTVECNIED KGNSSYRCTY
451        461        471        481        491
KPTKEGVHTI YITFAGSQIS KSPFTVTVGE ACNPSLCRAK GRGLQPKGLR
501        511        521        531        541
VKETADFKVY TKGAGTGDLK VTIKGPKGLE EPCKKKDLGD GVYSFEYYPT
551        561        571        581        591
TTGNYTITIT WGGQQIPRSP FEVKIGTEAG PQKVRAWGPG LEGGVVGSSA
601        611        621        631        641
DFVVEAVGDD VGTLGFSVEG PSQAKIECDD KGDGSCDVRY WPTEAGEYAV
651        661        671        681        691
HVLCNNEDIQ YSPFMAEIKP SPGKDFHPEK VKAYGPGLQS TGLAMSKPAE
701        711        721        731        741
FTVDAKQGGK APLKIQAQDG DGNPVDVQVK DNGNGTYSCF YTPRKPLKHT
751        761        771        781        791
VMVSWGGVNI PDSPFRMSIG AGSHPNKVKV SGPGVAKTGL KAFEPTYFTV
801        811        821        831        841
DCSEAGQGDI SIGIKCAPGV VGPAEADIDF DIIRNDNDTF TVKYTPPGAG
851        861        871        881        891
SYTIMVLFAD QAIPMTPFRI KVDPSHDASK VKAEGPGLSR SGIEWNKPTH
901        911        921        931        941
FTVNTKGAGK AKLDVQFTGP TKADAVKDFD IVNNHDNTYT VKYTPVQQGN
951        961        971        981        991
MGVNVTYGGD SIPKSPFSVG VAPSLDLSKI KVSGPGEKMT VGKDQEFTVK
1001       1011       1021       1031       1041
```

**Figure S2**

SKGAGGQGKV AAKVTGPSGK PVPCKVEPSL SPETSQVRFI PREQGPYEVE
1051 1061 1071 1081 1091

LTYDGAPIPG SPFPVEAVAP ADPSKVRCSG PGLERAKVGE TGKFNVDCTN
1101 1111 1121 1131 1141

AGPAELTIEI ISDNGTEAEV HIQDNGDGTY TITYIPLYPG VYTITIRYGG
1151 1161 1171 1181 1191

QDVPNFPARL TVEPAVDSTG VKVFGPGVGN KGVFREATTD FTVDARALTK
1201 1211 1221 1231 1241

SGGNHIKTRI NNPSGNRTDA LIRDLGDGTY QVEYTPYEEG VHNVEVCYDD
1251 1261 1271 1281 1291

APVPNSPFRV PVTEGCDPAR VRVHGPGLQS GITNKPNKFT VETRGAGTGG
1301 1311 1321 1331 1341

LGLAVEGPSE AKMSCTDNKD GSCSVEYVPY EPGTYNLNIT YGGQPVKGSP
1351 1361 1371 1381 1391

FSVPVHDVVD ASKVKCLGQG LGNNVRANIP QSFTVDTSKA GVAPLQVRVQ
1401 1411 1421 1431 1441

GPKGIVEPVE VVDNGDKTHT VSYVPTREGP YSVSVLYADE EIPRSPFKVK
1451 1461 1471 1481 1491

VLPTHDASKV KASGPGLNTT GVPASLPVEF TIDAKDAGEG LLAVQITDPE
1501 1511 1521 1531 1541

GKPKKANIRD NQDGTYLVSY VPDMTGRYTI LIKYGGDDIP YSPYRIRALP
1551 1561 1571 1581 1591

TGDASKCTVT VSIGGHGLGA GVGPTIQIGE ETVITVDAKA AGKGKVTCTV
1601 1611 1621 1631 1641

CTPDGTEVDV DVVENEDGTF DIFYTAPQPG KYVICVRFGG EHIPNSPFQV
1651 1661 1671 1681 1691

TALDGAPTEQ MLQQTQVPQY AYAPGVGQPW ATDRPVGMNG LDVAGLRPFD
1701 1711 1721 1731 1741

LVIPFTIKKG EITGDVRMPS GKIAKPDITD NKDGTVTVKY APTEAGLHEM
1751 1761 1771 1781 1791

DIKYDGIHIP GSPLQFYVDY VNSGHVTAYG PGLIHGMVNK PAIFTVNTKD
1801 1811 1821 1831 1841

AGEGGLSLAI EGPSKADISC TDNQDGTCTV SYLPVLPGDY NILVRYNDKH
1851 1861 1871 1881 1891

IPGSPFVAKI TGDDSMRMSH LKVGSAADIP LDIGELDLSQ LTASLTTPSG
1901 1911 1921 1931 1941

REEPCLLKML RNGHVGISFV PKEIGEHLVN IKKNGRHIPS SPITVMINQS
1951 1961 1971 1981 1991

EIGDASRVRV SGPGLSEART FEPAEFIIDT REAGYGGLSL SIEGPSKVDI
2001 2011 2021 2031 2041

NTEDQEDGTC KVTYCPTEPG NYIINIKFAD QHVPGSAFTV KVTGEGRMKE
2051 2061 2071 2081 2091

SITRRRRAAS VANVGSQCDL SLKIPEINIG DMTAQVTSPS GKVHKAEIME
2101 2111 2121 2131 2141

GENNTYCIRF VPTEMGVHTV SVKYQGQHVP GSPFQFTVGP LGEGGAHKVR
2151 2161 2171 2181 2191

**Figure S2**

AGGPGLERAE AGIPAEFSIW TREAGAGGLS IAVEGPSKAE IAFEDRKDGS
2201 2211 2221 2231 2241
SGVSYIVQEP GDYEVSIKFN DEHIPDSPFI VPVASPSDDA RRLTVASLQE
2251 2261 2271 2281 2291
SGLKVNQPAS FAVSLNGAKG VIDAKVHSPS GALEECCVTE IDEDKYAVRF
2301 2311 2321 2331 2341
IPRENGLYLI DVKFNGSHIP GSPFKIRVGE TGQAGDPGMV SAYGAGLEGG
2351 2361 2371 2381 2391
NTGSPCEFIV NTSSAGPGAL AVTIDGPSKV KMDCQECPEG YKVIYTPMAP
2401 2411 2421 2431 2441
GNYLISIKYG GPYHIVGSPY KAKITGPRLV SSHSLHETSS VLVDPISRSS
2451 2461 2471 2481 2491
ATMQQGAPAW ASSDASHVVA KGLGLTKGFV GQKNSFSVDC SKAGRNMLLV
2501 2511 2521 2531 2541
GVDGPKVPCE EILVKHLGNR LYNVSYQLKE KGDYILVVKW GDEHIPGSPF
2551
HISVP

**Legend:**

**The selection scale:**

| 1 | 2 | 3 | 4 | 5 | 6 | 7 |
|---|---|---|---|---|---|---|

Positive selection — Purifying selection

**Figure S2**

**Gene: *gnb2l1***

| | | | | |
|---|---|---|---|---|
| 1 | 11 | 21 | 31 | 41 |
| M T E Q M T V R G T | L K G H N G W V T Q | I A T T P Q F P D M | I L S A S R D K S I | I M W K L T R D E T |
| 51 | 61 | 71 | 81 | 91 |
| N Y G I P Q R A L R | G H S H F V S D V V | I S S D G Q F A L S | G S W D G T L R L W | D L T T G T T T R R |
| 101 | 111 | 121 | 131 | 141 |
| F V G H T K D V L S | V A F S A D N R Q I | V S G S R D K T I K | L W N T L G V C K Y | T I Q D E S H T E W |
| 151 | 161 | 171 | 181 | 191 |
| V S C V R F S P N S | S N P I I V S C G W | D K M V K V W N L A | N C K L K T N H I G | H T G Y L N T V T V |
| 201 | 211 | 221 | 231 | 241 |
| S P D G S L C A S G | G K D G Q A M L W D | L N E G K H L Y T L | D G G D T I N A L C | F S P N R Y W L C A |
| 251 | 261 | 271 | 281 | 291 |
| A T G P S I K I W D | L E G K I I V D E L | R Q E V I S T S S K | A E P P Q C T S L A | W S A D G Q T L F A |
| 301 | 311 | | | |
| G Y T D N L I R V W | Q V T I G T R | | | |

**Legend:**

**The selection scale:**

| | | | | | | |
|---|---|---|---|---|---|---|
| 1 | 2 | 3 | 4 | 5 | 6 | 7 |

Positive selection Purifying selection

Figure S2

**Gene: *grip1***

| | | | | |
|---|---|---|---|---|
| 1 | 11 | 21 | 31 | 41 |
| MERFLGFVKQ | IRRSRRRKGK | KYRPEEDYHE | GYEDVYYYAS | EHLTFVRDEG |
| 51 | 61 | 71 | 81 | 91 |
| PYTKPSNPSK | PPDGALAVRR | QSIPDEFKGS | TIVELMKKEG | TTLGLTVSGG |
| 101 | 111 | 121 | 131 | 141 |
| IDKDGKPRVS | NLRQGGIAAR | SDQLNVGDYI | KSVNGINLTK | FRHDEIISLL |
| 151 | 161 | 171 | 181 | 191 |
| KNVGERVVLE | VEYELPPISV | QGSGVIFKNV | EVTLHKEGNT | FGFVIRGGAH |
| 201 | 211 | 221 | 231 | 241 |
| EDRNKSRPVV | ITTVRPGGPA | DREGTVKSGD | RLLSIDGIRL | HGTSHAEAMS |
| 251 | 261 | 271 | 281 | 291 |
| ILKQCGQEAT | LLIEYDVSVM | DSIATASGPL | LVEVAKSPGS | SLGIALTTSM |
| 301 | 311 | 321 | 331 | 341 |
| YCNKQVIVID | KVKPASIADR | CGALHAGDHI | LSVDGTSMEY | CTLAEATQLL |
| 351 | 361 | 371 | 381 | 391 |
| ASACEHVKLE | ILPHHQTRLP | LKGSDHVKVQ | RSNRQLPWDS | CANNNRNFLP |
| 401 | 411 | 421 | 431 | 441 |
| YQHYNTYHPD | HSRTQASKYQ | KPSPNNLPLV | SSSFSPTSMS | AYSLSSLNMG |
| 451 | 461 | 471 | 481 | 491 |
| TLPRNMYPTS | PRGTLMRRKL | KKKDHKSSLS | LASSTLGLAG | QVVHTETTEV |
| 501 | 511 | 521 | 531 | 541 |
| TLVGDPILGF | GIQLQGGVFA | TETLSSPPLI | AYMDPDSPAE | RSGILQIGDR |
| 551 | 561 | 571 | 581 | 591 |
| ILAINGIPTE | DSTLEETNQL | LRDSSITSKV | TLEIEFDVAE | SVIPSSGTFH |
| 601 | 611 | 621 | 631 | 641 |
| VKLPKKPGVE | LGITISSPSS | RKPGDPLIIS | DIKKGSVAHR | TGTLELGDKL |
| 651 | 661 | 671 | 681 | 691 |
| LAIDNIRLDN | CSMEDAVQIL | QQCEDLVKLK | IRKDEDNSDE | QESSGAIIYT |
| 701 | 711 | 721 | 731 | 741 |
| VELKRYGGPL | GITISGTEEP | FDPIIISSLT | KGGLAERTGA | IHIGDRILAI |
| 751 | 761 | 771 | 781 | 791 |
| NSNSLKGKPL | SEAIHLLQMA | GESVTLKIKK | QGEATSPKKP | SASGRLSELS |
| 801 | 811 | 821 | 831 | 841 |
| DVEDESQAAQ | KTGKLSDMYS | TTIPSVDSAV | ESWDGSGIDT | VFGTQVPGYQ |
| 851 | 861 | 871 | 881 | 891 |
| ASGYSFHSHE | WRNAKSRGSL | SPVSRQRNNI | FQDIGLSDDE | WDRPTTSGFT |
| 901 | 911 | 921 | 931 | 941 |
| VGNDGTEPDQ | EENFWSQALE | DLETCGQSGI | LRELEATIMS | GSTMSLNHEP |
| 951 | 961 | 971 | 981 | 991 |
| QPQRSLLGRQ | ASFQERSLSR | PQYTPTNRSN | TLPTEAGRKA | FAMRKIKQEM |
| 1001 | 1011 | 1021 | 1031 | 1041 |

**Figure S2**

KDIMSPTPVE LHKVTLYKDT DAEDFGFSVS DGLLEKGVYV NNIRPSGPAD
1051 1061 1071 1081 1091
LGGLKPYDRL LQINHVRTRD FDCCLVVPLI AESGNKLDLV ISRNPLASQP
1101 1111 1121 1131 1141
VTELDQQPSE APSEWSEQND ALQQPQQQQS SRLAGVDARE PTKTL

**Legend:**

**The selection scale:**

| 1 | 2 | 3 | 4 | 5 | 6 | 7 |
|---|---|---|---|---|---|---|

Positive selection Purifying selection

 

**Figure S2**

**Gene: *kat5***

| 1 | 11 | 21 | 31 | 41 |
|---|---|---|---|---|
| MADTSMEIVE | GCRLPVLRKN | QENEDEWPLA | EILSVKDTLG | RKLFYVHYID |
| 51 | 61 | 71 | 81 | 91 |
| FNKRLDEWVT | PDRLDMKKLQ | FPKKEAKTPT | KNGLPGSRPS | SPEREVRKSL |
| 101 | 111 | 121 | 131 | 141 |
| DLNVQAASAP | SRGKTLPTPK | RKAETVSLAS | QVTPATSVPS | LPSSAEATQA |
| 151 | 161 | 171 | 181 | 191 |
| SVYPAARETF | TTKTREDEPL | TSITTVTNGT | TRRVLPPSQP | GRKRKTCGTD |
| 201 | 211 | 221 | 231 | 241 |
| EMIKVFQYNS | PRTTSGCLPP | GEDSQDSSDG | IPTAPRMTGS | LVSDRSHDDI |
| 251 | 261 | 271 | 281 | 291 |
| ITRMKNIDCI | ELGRHRLKPW | YFSPYPQELT | TLPILYLCEF | CLKYLKSLKC |
| 301 | 311 | 321 | 331 | 341 |
| LQRHLTKCNL | RHPPGNEIYR | KGTISFFEID | GRKNKTYSQN | LCLLAKCFLD |
| 351 | 361 | 371 | 381 | 391 |
| HKTLYYDTDP | FLFYVMTEYD | CKGFHIVGYF | SKEKESTEDY | NVACILTLPP |
| 401 | 411 | 421 | 431 | 441 |
| YQRRGYGKLL | IEFSYELSKV | EGKTGTPEKP | LSDLGLLSYR | SYWSQTILEI |
| 451 | 461 | 471 | 481 | 491 |
| LMDLKSDNGE | RPQITINEIS | EITSVKKEDV | ISTLQYLNLI | NYYKGQYILT |
| 501 | 511 | 521 | 531 | |
| LSEDIVDGHE | RAMQKRHLRI | DPKCLHFTPK | DWSKRGKW | |

**Legend:**

**The selection scale:**

| 1 | 2 | 3 | 4 | 5 | 6 | 7 |
|---|---|---|---|---|---|---|

Positive selection    Purifying selection

**Figure S2**

**Gene: *limk2***

```
1          11         21         31         41
MEDRQGDESY RCVGCGSRIL DTFYIKVLQD VWHNSCFQCS ECCDHLTNWY
51         61         71         81         91
YEKDGKLYCR KHYWENFGES CHGCSLLMTG PVMAAGEYKY HPECFVCLSC
101        111        121        131        141
KVVIEERDTY ALVERSKLYC GTCHQQIVLT PVLEKRSSVL ACDSLPHTVT
151        161        171        181        191
LISVPAATNG KRGLSVSVIG DCSGGTTSVQ VKEVLGMHIS PEVRNAIHAG
201        211        221        231        241
DRILEINGVP VGTLMEEEVE DLIQRTNQTL QLVIEYDPVR QRLDRLRLGS
251        261        271        281        291
PRSRLRVPGA SRLRLPSPAD PVLESRETLE DSTLKRRSLR RSNSICKSPG
301        311        321        331        341
PASPKEYPGL FRDISRSESL RSSTSCSHRI FRPCDLIHGE VLGKGFFGQA
351        361        371        381        391
IKVTHKATGE VMVMKELIRC DEETQKTFLK EVKVMRSLEH PNVLKFIGVL
401        411        421        431        441
YKDKRLNLIT EFIEGGTLKD FIRDMDPFPW EQRVSFAKGI ASGMAYLHSM
451        461        471        481        491
SIIHRDLNSH NCLVKLDNTV VVADFGLSRL IVEEKSRAPP EKQSAKKRTF
501        511        521        531        541
RRIDRKKRYT VVGNPYWMAP EMLNGKRYDE RVDIFSFGIV LCEIIGQVYA
551        561        571        581        591
DPECLPRTLD FGLNVRKFLE KFLPEDCPSA FFPLAVACCD LVPENRPTFE
601        611        621        631        641
KLVDCFDALA LNLELGIPLP AELEVLHHHL CGAHSPVGAD LPGSGPPVRH
651
PASASLGENG S
```

**Legend:**

**The selection scale:**

| | | | | | | |
|---|---|---|---|---|---|---|
| 1 | 2 | 3 | 4 | 5 | 6 | 7 |

Positive selection Purifying selection

**Figure S2**

## Gene: *mapk1*

```
1          11         21         31         41
MATAAVSGAA GGAPNTGSGA ELVRGQAFDV GPRYSNLSYI GEGAYGMVCS
51         61         71         81         91
AYDKENKVRV AIKKISPFEH QTYCQRTLRE IKILLRFKHE NIIGINDIIR
101        111        121        131        141
APSIDQMKDV YIVQDLMETD LYKLLKTQHL SNDHICYFLY QILRGLKYIH
151        161        171        181        191
SANVLHRDLK PSNLLLNTTC DLKICDFGLA RVADPDHDHT GFLTEYVATR
201        211        221        231        241
WYRAPEIMLN SKGYTKSIDI WSVGCILAEM LSNRPIFPGK HYLDQLNHIL
251        261        271        281        291
GILGSPSQED LNCIINIKAR NYLLSLPLRN KVPWNRLFPN ADPKALDLLD
301        311        321        331        341
KMLTFNPHKR IEVEEALAHP YLEQYYDPTD EPVAEAPFKF DMELDDLPKE
351        361
TLKELIFEET ARFQPGYRS
```

**Legend:**

**The selection scale:**

| 1 | 2 | 3 | 4 | 5 | 6 | 7 |
|---|---|---|---|---|---|---|

Positive selection    Purifying selection

**Figure S2**

**Gene: *mapk3***

| | | | | |
|---|---|---|---|---|
| 1 | 11 | 21 | 31 | 41 |
| MAESGSSAAA | AGAAGSNSSA | AGPGGAVAPG | GPSGAAGSKP | GLESVKGQNF |
| 51 | 61 | 71 | 81 | 91 |
| DVGPRYTDLQ | YIGEGAYGMV | CSAFDNVNKI | RVAIKKISPF | EHQTYCQRTL |
| 101 | 111 | 121 | 131 | 141 |
| REIKILLRFH | HENIIGINDI | LRARHIDYMR | DVYIVQDLME | TDLYKLLKTQ |
| 151 | 161 | 171 | 181 | 191 |
| QLSNDHICYF | LYQILRGLKY | IHSANVLHRD | LKPSNLLINT | TCDLKICDFG |
| 201 | 211 | 221 | 231 | 241 |
| LARIADPEHD | HTGFLTEYVA | TRWYRAPEIM | LNSKGYTKSI | DIWSVGCILA |
| 251 | 261 | 271 | 281 | 291 |
| EMLSNRPIFP | GKHYLDQLNH | ILGVLGSPSQ | DDLNCIINMK | ARNYLQSLPQ |
| 301 | 311 | 321 | 331 | 341 |
| KPKIPWNKLF | PKADNKALDL | LDRMLTFNPL | KRINVEQALA | HPYLEQYYDP |
| 351 | 361 | 371 | 381 | 391 |
| SDEPVAEEPF | TFNMELDDLP | KEKLKELIFE | ETARFQANYQ | GS |

**Legend:**

**The selection scale:**

| | | | | | | |
|---|---|---|---|---|---|---|
| 1 | 2 | 3 | 4 | 5 | 6 | 7 |

Positive selection    Purifying selection

**Figure S2**

## Gene: *med1*

| | | | | |
|---|---|---|---|---|
| 1 | 11 | 21 | 31 | 41 |
| MAAVSGVVIS | GCSPARELGP | GAPTVGPPTI | GDRPRPEEGT | EAEKQSRVGA |
| 51 | 61 | 71 | 81 | 91 |
| LLERLHAKHC | ASRPWQETSK | VVRQAMEKRN | VLNAGGHQLL | LTCLETLQRA |
| 101 | 111 | 121 | 131 | 141 |
| LKVSSLPAMT | DRLESIARQN | GLGSHLSPSG | TECYITSDMF | YVEVQLDSGG |
| 151 | 161 | 171 | 181 | 191 |
| QLVDVKVAHH | GENPASCPEL | VQHLRERNFE | EFSKHLKGLV | NLYKLPGDNK |
| 201 | 211 | 221 | 231 | 241 |
| LKTKMYLALQ | SLEMDLTKMM | HMFRLATNAN | TVETILHGSV | GLVTARSGGH |
| 251 | 261 | 271 | 281 | 291 |
| LVTLQCYVSP | YDVFEEETGA | LLNLTDSNVP | RNLGVGVSVT | IEGTSSVYKL |
| 301 | 311 | 321 | 331 | 341 |
| PIAPLITGTH | PVDNKGTPSF | SSVTNSNCVD | LPACFFLKLR | RPLPFSLSFI |
| 351 | 361 | 371 | 381 | 391 |
| HRMGNATGIP | LFETAPPLAP | LYELITQSQL | QEEGGGALPP | LAHNMRFYAS |
| 401 | 411 | 421 | 431 | 441 |
| LPGQQHCYFL | NRDAPVQDGR | CLQGALVTKV | PFRHPAQVPA | LLDIIRHQAA |
| 451 | 461 | 471 | 481 | 491 |
| YNTLIGSCVK | RTVLKEDTPG | LLQFEVCPLT | DSSFSVSFQH | PVNESLVCVV |
| 501 | 511 | 521 | 531 | 541 |
| MEVLDSRQVS | CKLYKGLSDA | LICTDDFITK | VVQRCMSIPV | TMRAIRRKAE |
| 551 | 561 | 571 | 581 | 591 |
| TIQADTPALS | LIAETVEDMV | KKNLPPAGSP | GYGMGTGGGG | NLMGIPGVGG |
| 601 | 611 | 621 | 631 | 641 |
| GNTPTGGGGS | SAGAAGGGGG | AGASFPGPIS | TLFGMGLALK | ERHEGRGPGG |
| 651 | 661 | 671 | 681 | 691 |
| EPMSQAGGAQ | QQQAPLQQPA | QQGHGDDFSK | VTQNPILTSL | LQITGNVGSS |
| 701 | 711 | 721 | 731 | 741 |
| PTQAPPAAGH | QPHHTPPPAS | SPASNTKNHP | MLMNLLKDNP | SQDFSSLYSS |
| 751 | 761 | 771 | 781 | 791 |
| SPLERQNSSG | SPRTDIMGGG | SCGGGGGVSG | GKTKKKRQRN | PDKAGGMGGA |
| 801 | 811 | 821 | 831 | 841 |
| MGLKPQGSLP | LALQHHQHHQ | LEDDFHRELF | SMDVDASQNP | IFDVNLPGDG |
| 851 | 861 | 871 | 881 | 891 |
| LDTPHSITPA | PSQCGTPPTG | PGVPYLSQGP | PQSQSQQQQQ | QQQVAPPQPP |
| 901 | 911 | 921 | 931 | 941 |
| PSGPSRMVRL | SSSDSIGADI | NEILSDIPEQ | AGKMAAGGGG | GHGPHHHGLG |
| 951 | 961 | 971 | 981 | 991 |
| GGDDGGALGT | PIRDSSSSGQ | GSAVFEADLF | SAGSNENPFT | DPVDLIADAA |
| 1001 | 1011 | 1021 | 1031 | 1041 |

**Figure S2**

TAATPNSDSS SNNFFPDTVD FNPELLAGPG FPQAYFEDSS PSADGDLDLV

1051 1061 1071 1081 1091

KGFGGGSQQH TPAGTPQNPT PHGRSTPDPS MKDPFEMSMV FGGSGGGGKP

1101 1111 1121 1131 1141

LLGGPLPAPD LADSHSGGGQ SPLMMMGMGG GGGSDFKSDA KAKQQQLMRG

1151 1161 1171 1181 1191

GGKEGNGGGG GGGSGLGGAS GGSSEGKQGK RSRTPSSDGK SKDKPPKRKK

1201 1211 1221 1231 1241

LDPEGKSPSH SSGGRPYTPP SGIGGSGGGS MGAGGGSKSP GSSGRSQTPP

1251 1261 1271 1281 1291

GGATPPIPKI TIQIPKGTLT GGKTSSHGGY TSSSSAGGGG GGAAGGGGSK

1301 1311 1321 1331 1341

SHHSHSSSSS SSSGKMKGSK SDGSLGQGGG SKPGGGGGGC SGGGGGGGGG

1351 1361 1371 1381 1391

SQSKGSSQGM GGGKPGSSPI TKHGLAGAGV VAGGGVGGGA KMKPQGGKPP

1401 1411 1421 1431 1441

ASLINPNIKP NISPSHSRGV GAPDKLSSPM KLQQQQVPGT PPSSKAKSPM

1451 1461 1471 1481 1491

GSGGGGLGSG SKTSSGSGMG PQKQQMGGTS SSGSSSSSSS SSSSSSSSSS

1501 1511 1521 1531 1541

SSSSSSFSAG SQSQYGGGGG GGGGGGGNNN PNAKGKSPSR NKKPSLTAVI

1551 1561 1571 1581 1591

DKLKHGVGTG GHGGEYMSKR DKGDKESKSK VSMSGVGSGD KKALDSKSGV

1601 1611 1621 1631 1641

VSSTGVAKII ISKPDGGSPS IKAKVTLQKP GEGSGEAMRS QLSGPKAYGS

1651 1661 1671 1681 1691

PLFSASTPKH DRCSPSHGRS PGYTPLNPDS ESESGSSSVA EKSHQNSPSS

1701 1711 1721 1731 1741

DDDQALRPLQ PPHEYMSSMP ISSGEKHKKH KKEKKKQKER ERERERDRDK

1751 1761 1771 1781 1791

EKKKSSSSCS TSSSHPLKAD SWSRSPIAAP DPSLSLLGSD RPARPSPAYL

1801 1811

RTEDDDLMDS ALTGNLDSFK

**Legend:**

**The selection scale:**

| 1 | 2 | 3 | 4 | 5 | 6 | 7 |
|---|---|---|---|---|---|---|

Positive selection — Purifying selection

**Figure S2**

## Gene: *med4*

```
1          11         21         31         41
XXXXXXXXXX XXXXXXXXXX XXXXXXXXXX XXXXXXXXXX XXXXXXXXXX
51         61         71         81         91
XXXXXXXXXX XXXXXXXXXX XXXXXXXXXX XXXXXXXXXX XXXXXXXXXX
101        111        121        131        141
XXXXXXMVHT DVECALMVNV FVSGSISSEE IIKYAHRISA SNAVCAPLNW
151        161        171        181        191
VPGDPRRPYP TDLEMRSGML GHMSNLPTNG VNGHLPGDAL AAGRLPGSLV
201        211        221        231        241
SPAAPTCLXX XXXXXXXXXX XXXXXXXXXX XXXXXXXXXX XXXXXXXXXX
251
XX
```

**Legend:**

**The selection scale:**

| 1 | 2 | 3 | 4 | 5 | 6 | 7 |
|---|---|---|---|---|---|---|

Positive selection    Purifying selection



T. Lorin, W. Salzburger & A. Böhne

Figure S2

**Gene: *med12***

| | | | | |
|---|---|---|---|---|
| 1 | 11 | 21 | 31 | 41 |
| MAAFGILSYE | HRPLKRPRLG | PPDVYPQDPK | QKEDELTALN | VKQGFNNQPA |
| 51 | 61 | 71 | 81 | 91 |
| VSGDEHGSAK | NVNFNPSKIS | SNFSSIIAEK | LRCNTFPDTG | KRKPQVNQKD |
| 101 | 111 | 121 | 131 | 141 |
| NFWLVTARSQ | SSINNWFTDL | AGTKPLTQLA | KKVPIFSKKE | EVFGYLAKYT |
| 151 | 161 | 171 | 181 | 191 |
| VPVMRAAWMI | KMTCAYYAAI | TETKVKKRHV | IDPCIEWTQI | ITKYLWEQLQ |
| 201 | 211 | 221 | 231 | 241 |
| KVAEFYRQSS | SQGCVSPLPS | TPVEVETAMK | QWEYNEKLAM | FMFQDGMLDR |
| 251 | 261 | 271 | 281 | 291 |
| HEFLTWVLEC | FEKIRPGEDE | LLKLLLPLML | QYSGEFVQSA | YLSRRLAYFC |
| 301 | 311 | 321 | 331 | 341 |
| TRRLNLLLSD | GTVGPGAGGH | QTHSITAQPG | NALPPTPTPQ | PAGGNQPQTP |
| 351 | 361 | 371 | 381 | 391 |
| FTDFYICPQH | RPLVFGLSCM | LQSIVLCCPS | ALVWHYSLTD | SRNKTGSPLD |
| 401 | 411 | 421 | 431 | 441 |
| LLPIAPSNLP | MPGGNTTFTQ | QVRAKVREIE | EQVKERGQAV | EFRWSFDKCQ |
| 451 | 461 | 471 | 481 | 491 |
| ETTAGFTIGR | VLHTLEVLDS | HSFEKSDFSN | SLDSLYNRIF | GSGQSKDGHE |
| 501 | 511 | 521 | 531 | 541 |
| MNPDDDAVVT | LLCEWAVSCK | RSGRHRAMVV | AKLLEKRQAE | IEAERCGESE |
| 551 | 561 | 571 | 581 | 591 |
| VVDEKGSVSS | GSLSAATLPV | FQDVLLQFLD | TQAPMLTEPG | NESERVEFSN |
| 601 | 611 | 621 | 631 | 641 |
| LVLLFCELIR | HDVFSHNIYM | CTLISRGDLA | SDSHLPRPRS | PSDEPSDESE |
| 651 | 661 | 671 | 681 | 691 |
| RKEQEAASSV | KMEDAGLSES | MEIDHNSSAI | FDDVMFSPPM | HCESKGSPSP |
| 701 | 711 | 721 | 731 | 741 |
| EKPAPEQDGK | STTKDKTLDP | AFPLVYELPR | HIQYATHFPI | PQEESASHEC |
| 751 | 761 | 771 | 781 | 791 |
| NQRLVVLYGV | GKQRDEARHT | IKKITKDILK | VLNRKSTAET | GGEEGQKRKR |
| 801 | 811 | 821 | 831 | 841 |
| TKPEAFPTAE | DIFSKFQHLS | HFDQHQVTSQ | VSRNVLEQIT | SFALGMSYHL |
| 851 | 861 | 871 | 881 | 891 |
| PLVQHIQFIF | DLMEYSLNIS | GLIDFAIQLL | NELSLVEAEL | LLKSSSLVGS |
| 901 | 911 | 921 | 931 | 941 |
| YTTGLCLCIV | AVLRRYHSCL | ILNPDQTAQV | FDGLRIVVKH | GVNPADCSSA |
| 951 | 961 | 971 | 981 | 991 |
| ERCILAYLYD | LYTSCSHLKS | KFGEIFSDFC | SKVKNSIYCN | IDPSDSNMLW |
| 1001 | 1011 | 1021 | 1031 | 1041 |

**Figure S2**

DPVFMIETIA NPSAHNFNHS MVGKILNDSP ANRYSFVCNV LMDVCVDHRD
1051 1061 1071 1081 1091
PERVNDIGIL CAELTAYCRS LSAEWLGVLK ALCCSSNNGN CGFNDLLCNV
1101 1111 1121 1131 1141
DVSDLSFHDS LATFVAILIA RQCLLLEDLV RCVAIPSLLN AACSEQDSEP
1151 1161 1171 1181 1191
GARLTCRILL HLFKTPQRNP SPQDSSKSDK PAVGIRSSCD RHLLAASQNS
1201 1211 1221 1231 1241
IVVGAVFAVL KAVFMLGDAE LKGSGFPHPA GLDDTPEDDL GSKKSGGRAV
1251 1261 1271 1281 1291
SIETASLDVY AKYVLKSICQ QEWVGERCLK SLSEDSSALQ DPVLVNIQAQ
1301 1311 1321 1331 1341
RLLQLICYPH RQLDSEEGEN PQRQRIKRIL QNMDQWTMRQ SSLELQLMIK
1351 1361 1371 1381 1391
QSTNNELNSL LENIAKATIE VFQKSAEMIS SNPLGNGSAV SGPIQGPVTN
1401 1411 1421 1431 1441
SNNASKMKPV LSSSERSGVW LVAPLIAKLP TTVQGHVLKA AGEELEKGQH
1451 1461 1471 1481 1491
LGSSSRKERD RQKQKSMSLL SQQPFLSLVL TCLKGQDEQR EGLLTSLYSQ
1501 1511 1521 1531 1541
VQQIVTNWRE DQYQDDCKAK QMMHEALKLR LNLVGGMFDT VQRSTQQTTE
1551 1561 1571 1581 1591
WAVLLLDIIS SGTVDMQSNN ELFTTVLDML SVLINGTLAA DMSSISQGSM
1601 1611 1621 1631 1641
EENKRAYMNL VKKLRKELGD RQSESLEKVR QLLPLPKQTR DVITCEPQGS
1651 1661 1671 1681 1691
LIDTKGNKIA GFEKEGLQVS TKQKISPWDV FEGLKHSAPL SWGWFGTVRV
1701 1711 1721 1731 1741
DRKVTKFEEQ QRLLLYHTHL KPKPRSYYLE PLPLPPEEEE PPTPVAPEPE
1751 1761 1771 1781 1791
KKLAEAVKPE KSSAAVATDS SKKKSSKKKR NHSSSKTEDF ASTQRGVPYT
1801 1811 1821 1831 1841
AGMPPEMLHG QQGHPFNRMV YGPQSMGMYP QNQPLPPGGP RLDTTYRPTR
1851 1861 1871 1881 1891
TLPMRPNRPA AYPNMMTGMP GGVGNLITAL DQQAYRAYKP QPPIQGQILR
1901 1911 1921 1931 1941
QQLQAKLSQG MLGQQVRQMP PNPSYGTLQP TQVPPPQGYT SYGPHMGMQQ
1951 1961 1971 1981 1991
HPSQTGGMVP PAYANQPFQG SHPAPNPAMV DSLRQMQQRP SGYIHQQAPG
2001 2011 2021 2031 2041
YVHAMQNTPR FAHQQASIIQ GLSHMPGQGV HPGMRSNQML DFLRQQQALR
2051 2061 2071 2081 2091
VGIHSLFGLS GHQACLLLHW RAGAHVTVML LCLYQTPFDQ VSAAQPPAQA
2101 2111 2121 2131 2141
QPQALGMQPL PPQQPMFPRQ GMQQTQQQQQ TAALVRQLQQ QLSNTQPQQN
2151

**Figure S2**

T N S F Y

**Legend:**

**The selection scale:**

| 1 | 2 | 3 | 4 | 5 | 6 | 7 |
|---|---|---|---|---|---|---|

Positive selection    Purifying selection

Figure S2

**Gene: *med13***

1 11 21 31 41
MSSCFVPNGA SLEDCHSNLF CLADLTGIKW KKYVWQGPTS APILFPVTEE
51 61 71 81 91
DPILCSFSRC LKADVLSVWR RHQTAGRREL WLFWWGEDPN FAELIHHELT
101 111 121 131 141
GEEEGAWESG LSYECRTLLF KAIHNLLERC LMNRSFVRIG KWFVKPYEKD
151 161 171 181 191
EKPINKSEHL SCAFTFFLHG DSNVCTSVEI NQHQPIYHLS EGHLTLAQQS
201 211 221 231 241
NSPFQVILSP FGLNGTLTGQ SFKLSDPPTQ KLIEEWKQFY PISPSSKESS
251 261 271 281 291
EDKPDDMDWE DDSLAAVEVL VAGVRMVYPA CLVLVPQSDI PAVTPVGSSH
301 311 321 331 341
CTAVYSGVHQ VPASTRDPAI SSVTLTPPTS PEEAQTMDSQ SAQKWVKFSS
351 361 371 381 391
VSDGFSTDST SHHGGKIPRK LASQVVDRVW QECNINRAQN KRKFSATTNG
401 411 421 431 441
TCEEESTDKA GSWDFVDPTQ RSNCNCSRHK NLKQRAGSTP GQPQAAGQPP
451 461 471 481 491
QPPPKHKAVE KPEKGEKQQK RPLTPFHHRT SICEDVSMEP DATGQRLGMR
501 511 521 531 541
AQDGGEPLKN PATPHSQHFY QPAPEPCLVP QKPAEDPRLD PLAQPFPPAY
551 561 571 581 591
SEAMEPTVYV GAAVSPDEDN AHAPWKFFIL PRKKDSDFAP PQLPGDKLRD
601 611 621 631 641
DLPGTGSQES VVSVTELMAT SKNPLKVSEG LVQMYIQRRT QYLSAAISDS
651 661 671 681 691
DHEPELDPYA FVDGDVEFSF RDKKDKQGGE REAGKKHKAD DGSNGGPVPA
701 711 721 731 741
EGEDAMSLFS PSAKTEDAQR SAAHNRTAST SLMHENDLAV SINDLDNLFN
751 761 771 781 791
SDEDELAPGA RRAVNGTDEK FGNKEAKPAS LDQLSCISSA DLHQMFPTPP
801 811 821 831 841
SLEQHIMGYS PMNMNSKEYG SLDNASGMTV LDGSSVLGGQ FKIEVEEGFC
851 861 871 881 891
SPKPSEIKDF SFVYKPEGCQ AFVGCSMFAP LKTLPSQCLP PIKLPEECLY
901 911 921 931 941
RPSWTVGKLE LLNPVPALTF LNKDGNIPSV GSAMDQDYIQ TYTPQTHTPF
951 961 971 981 991
MSNSAPPSNG GTGILPSPAT PRFSAPTPRT PRTPRTPRGP ASVQGSLKYE
1001 1011 1021 1031 1041

**Figure S2**

NSDLYSPAST PSTCRPLNSV EPATVPSIPE AHSLYVNLIL SESVMNLFKD

1051 1061 1071 1081 1091

CNFDSCCVCV CNMNIKGADV GVYIPDPNCE VQYSCTCGFS AVMNRRYGNG

1101 1111 1121 1131 1141

SGLFLEDELD IIGRGSDASR EVEKRFEAVH GSGASAERAG SLRDQVPDDL

1151 1161 1171 1181 1191

ILLLQDQCTN PFSPITAPEP DSAPARGPGA PPPCVRVEER DCYSDCYLAL

1201 1211 1221 1231 1241

EHGRQFMDNM SGGKVDEALV KSTCLHHWAK RNAVDVSMLC SQDVLRVLLS

1251 1261 1271 1281 1291

LQPVLQDAIQ KKRTVRSWGV QGPLTWQQFH KMAGRGSYGT DESPEPLPIP

1301 1311 1321 1331 1341

TFLVGYEYDF VVLSPFGLPY WEKLLLDPFG SQRDVGYLVL CPENEALLSG

1351 1361 1371 1381 1391

AKGFFRDLTA VYESCRLGQH RPISKTHPDG IVRVGTAAAK KLADQPVSDW

1401 1411 1421 1431 1441

FLKAASSNSD SFAKLKLYAQ VCRHDLAPYL AAQSLDSSLL VQPSPPPASS

1451 1461 1471 1481 1491

QSSSTPAPVV SAPGSQGTLT SSGTMTATAG TAIPPSASGT PSSTLPSSGP

1501 1511 1521 1531 1541

VGMGSSLQSS KPSSFPPFGN MGAQSGSSQS GTLGQQAGTQ APGITGENVP

1551 1561 1571 1581 1591

GAAQTQGPAE PPESTMEREK VGVPTDGDSH AITYPPAIVV YIVDPFTYEE

1601 1611 1621 1631 1641

MEGGAQSSSV WTLGLLRCYL EMLQSLPPHI RNAVSVQIIP CQYLLQPVKN

1651 1661 1671 1681 1691

DERHIYAQHL KSLAFSVFSQ CRRPLPISTN VKTLTGFGPG LALDTALKSP

1701 1711 1721 1731 1741

ERPECLRLYT PPFILAPVKD KQTELGETFG EASQKYNVLF VGYCLSHDQK

1751 1761 1771 1781 1791

WLLATCTDLY GELLETCIIN IDVPNRARRK KGSARRLGLQ KLWEWCLGLV

1801 1811 1821 1831 1841

QMTSLPWRVV IGRLGRIGHG ELRDWSILLS RRNLQSLSRR LKEMCRMCGI

1851 1861 1871 1881 1891

SAADTPSILS ACLVAMEPQG SFVIMPDSVS TGSVFGRSTT LNMQTSQLNT

1901 1911 1921 1931 1941

PQDTSCTHIL VFPTSAFVQV ANSNYTTENI DIAFNPINDG SDGMGIFDLL

1951 1961 1971 1981 1991

DTGEDLVDPD IINILPASPT TSPVHSPGSH YPHGGDGSKG QSTDRLESHE

2001 2011 2021 2031 2041

EAPNILQQPL ALGYFVSTAK AGPLPDWFWA ACPQAQNQCP LFLKASLHLH

2051 2061 2071 2081 2091

VSSVQSDELL HSKHSHPLDS NQTSDVLRFV LEQYNALSWL TCDPATQDRR

2101 2111

SCLPIHFVVL NQMYNFIMNM L

## Figure S2

**Legend:**

**The selection scale:**

| | | | | | | |
|---|---|---|---|---|---|---|
| 1 | 2 | 3 | 4 | 5 | 6 | 7 |

Positive selection Purifying selection

**Figure S2**

## Gene: *med14*

```
1          11         21         31         41
MAPVQIGSDG QLVPAGGPTS ALQPPPPGAP SGTPATHGVR LSVLIEFLLQ
51         61         71         81         91
RTYHEITLLA ELLPRKTDME RKIEIVQFAS RTRQLFVRLL ALVKWASNAG
101        111        121        131        141
KVEKCAMISS FLDQQAFLFV DTADRLASLA RDALVHARLP SFAIPFAIDV
151        161        171        181        191
LTTGSYPRLP TCIRDKIIPP DPITKAEKQA TLSQLNQILR HRLVTTDLPP
201        211        221        231        241
QLANLTVANG RVKFRVEGEF EATLTVMGDD PDIPWRLLKL EILVEDKETG
251        261        271        281        291
DGRALVHSMQ VSFIHELVQS RLFADEKPLQ DMYNCLHSFC LSLQLEVLHS
301        311        321        331        341
QTLMLIRERW GDLVQVERYI PAKCLTLAVW NQQVLGRKTG TASVHKVHIK
351        361        371        381        391
IDETDGSKPL QISHEPLLPA CDSKLMERAM KIDHLSVEKL LIDSVHARSH
401        411        421        431        441
QKLQELKAIL KNYNPSDNSF IETALPTLVI PILEPCGRSE CLHIFVDLHS
451        461        471        481        491
GTFQPMLYGT DQSTLDEMEK SINDDMKRII PWLQQLKFWL GEQRCRQSVK
501        511        521        531        541
HLPTVCSKTL HLSNAASHPA GSLSKHRLFI RLTRLPQYYI VAEMFDVPGC
551        561        571        581        591
PTELEYKYYF LSVTMLEGDE GPPSAQLLQQ FKPNLEELVL DATPGRGAKS
601        611        621        631        641
GTKRKLSGDQ GAIEPKKPKR AGEMCAFNKV LAHLVAMCDT NMPFIGLRCE
651        661        671        681        691
LSTMEIPHQG VQVEGDGCSH AIRILKIPPS KGVSEETRRV LERSLLDCTF
701        711        721        731        741
RLQGRNNRTW VAELVFANCP LTSTSSKEQA STRHVYLTYE NPLSEPVGGR
751        761        771        781        791
KVVEMFLNDW CSIAQLYECV LDFARSLGDM PSYLSLFSEI RLYNYRKLVL
801        811        821        831        841
GYGSTKGSSI TIQWNSVTQK FHISLGTVGP NSGCSNCHNI ILHQLQEMFN
851        861        871        881        891
KTPSVVQLLQ VLFDTQGPLN AINKLPTVPM LGLTQRTNTA YQCFSILPQS
901        911        921        931        941
PTHIRLAFRN MYCIDIYCRS RGVVAIRDGA YSLFDNTKIV EGFYPAPGLK
951        961        971        981        991
TFLNMFVDSN QDARRRSVNE DDNPPSPVGV DVMDALMSQL QPPPGQPQPF
1001       1011       1021       1031       1041
```

**Figure S2**

GKQAGGTGVY PITSPPTSYH TGVTPSPSMM PTQSPGTIHA ASSPSGALRA

1051 1061 1071 1081 1091

PSPASFGPTP SPSSLGISMG QTSNFASPHG TLDPSSPYAL MSPGQRSSNW

1101 1111 1121 1131 1141

PGSPQVSGPS PAARMPGMSP ANPSLHSPIP DASHSPRAGT SSQAMPTNMP

1151 1161 1171 1181 1191

PPRKLPQRSW AASIPTILTH SALHVLLLPS PTPCLVPGLA GSYLCSPLER

1201 1211 1221 1231 1241

FLGSVIMRRH LQRIIQQEPN LQIVNSNEPG VIMFKTDVLK CRVALNPKTY

1251 1261 1271 1281 1291

QTLQLKVTPD NTGPWSQEEL QVLEKFFETR VAGPPFKYNT LNAFTKLLGA

1301 1311 1321 1331 1341

PTHILRDCVR IMKLELFPDQ ATQLKWNVQF CLTIPPSAPP IAPPGTIAVV

1351 1361 1371 1381 1391

LKSKMLFFLQ LTQRLPVPQE PISIIVPIVY DMATGLTQQA DIPRQPSSSG

1401 1411 1421 1431 1441

AAALMVSSIL KRFNEMHPPR QSECTIFASV HELMANLTLP PGGRP

**Legend:**

**The selection scale:**

| 1 | 2 | 3 | 4 | 5 | 6 | 7 |
|---|---|---|---|---|---|---|

Positive selection Purifying selection

**Figure S2**

**Gene: *med16***

| | | | | |
|---|---|---|---|---|
| 1 | 11 | 21 | 31 | 41 |
| MDIAYVCEWE | KRPKSNHCPS | IPLVCAWSCR | NLLAFTTDLK | NEEEEKDLTH |
| 51 | 61 | 71 | 81 | 91 |
| MIHIIDSEHP | WDVYSINSGH | TEVISCLEWD | QSGSRLLSAD | GDGQIKCWAM |
| 101 | 111 | 121 | 131 | 141 |
| TEHLVNSWES | TLGSAVEGDP | IVALSWLHNG | VKLALHVEKS | GSTNFGEKFS |
| 151 | 161 | 171 | 181 | 191 |
| RVKFSPSLTL | FGGKPMEGWM | AVTVSGLVTV | SLLKPNGALL | TASESLCRLR |
| 201 | 211 | 221 | 231 | 241 |
| GRVALADIAF | TGGGNIVVAA | TDGSSSAPVQ | FYKVCVSVVN | EKCRIDTELL |
| 251 | 261 | 271 | 281 | 291 |
| PSLFMRCTTD | PARKDKYPAV | THLKFLTREN | SEQVLLCASS | QLGSIVECWS |
| 301 | 311 | 321 | 331 | 341 |
| LRKEGLPVNN | IFQHRSPVVG | EKQPMILKWR | ILSATNDLDR | VSAVALPKLP |
| 351 | 361 | 371 | 381 | 391 |
| ISISNTDLKV | ASDTKFCPGL | GLALAFHDGS | IHILHRLSLH | TMGVFYGSSS |
| 401 | 411 | 421 | 431 | 441 |
| SNPQRVGEEP | ALKRQRGGGP | TTHFKALQFS | CTSLALVGVD | NHGKLHMLRV |
| 451 | 461 | 471 | 481 | 491 |
| SPSMGQMLDI | NTLLRHLLFL | LEYCMVTGYD | WWDVLLHVQP | NMVHNLVEKL |
| 501 | 511 | 521 | 531 | 541 |
| HEEYMRQNQA | LQQVLSTRVV | AMKASLCKLS | SATAPRACDF | HAKLLLIAIS |
| 551 | 561 | 571 | 581 | 591 |
| STLKSLLRPH | LLNTPDKSPG | DRLAEICAKN | TDTDIDKVMI | NLKTEEFVLD |
| 601 | 611 | 621 | 631 | 641 |
| ASTLQSLQQL | IQWVGDFVLY | LLANLPNQGS | IVRPGFGFLR | DGASLGLLRE |
| 651 | 661 | 671 | 681 | 691 |
| MMVVIRIWGL | LKPGCLPIYT | ATSDNQDSMF | LLFRLLTKLW | LCSRDESHPQ |
| 701 | 711 | 721 | 731 | 741 |
| EPDEPLIDEC | CLLPSQLLVP | AMDWLPVSDG | IICKLQGKHP | LRLQFGKPYS |
| 751 | 761 | 771 | 781 | 791 |
| LPGLNSGTQV | EIFTRTPGSQ | RMDNLRCLHM | GVCPTEDSKA | CTRCGCVTML |
| 801 | 811 | 821 | 831 | |
| RSPNKTTAVK | QWEQRWIKNC | LCGGLWRRIP | TTIS | |

**Legend:**

**The selection scale:**

1 2 3 4 5 6 7

Positive selection — Purifying selection

**Figure S2**

## Gene: *med17*

| 1 | 11 | 21 | 31 | 41 |
|---|---|---|---|---|
| MSGGPAVRVS | IESACEKQVQ | EVSLDGIETY | VQPLSMSQNL | AKLAQRIDFG |
| 51 | 61 | 71 | 81 | 91 |
| QGSDSEDEGG | EGESRDQEWG | KQEAEEDEAT | VKFQPSLWPW | DSVRNNLRSA |
| 101 | 111 | 121 | 131 | 141 |
| LTEMCVLYDV | LSVVKEKKYM | TLDPVSHDVT | AGKTPQVLQL | IGKKKSLATA |
| 151 | 161 | 171 | 181 | 191 |
| AQLLLKGAEK | LSKSVAENQE | NRRQRDFNSE | LLRLRSQWKL | RKVGDKILGD |
| 201 | 211 | 221 | 231 | 241 |
| LSYKSAGSLF | PHHGTFEVIK | NTDIDLDKKI | PDDYCPLDVQ | IPSDLEGSAY |
| 251 | 261 | 271 | 281 | 291 |
| IKVSIQKQAP | DIGDLGTVSL | FRRQPKAKSG | SQPWHAKLEA | AQNVLLCKEI |
| 301 | 311 | 321 | 331 | 341 |
| FAQLSREAVQ | IKSQIPHIVV | KNQIISQPFP | GLQLSISLCH | STGEKKNQKA |
| 351 | 361 | 371 | 381 | 391 |
| SPEKSKPDDH | LYVLEHNLHQ | LMREFHKQTL | SSAVMPHPAS | APFGHKRLRL |
| 401 | 411 | 421 | 431 | 441 |
| AGPMAFDKAE | IASLQQNEGL | LEKIIKQAKH | IFLRRRTART | IDSLASRIED |
| 451 | 461 | 471 | 481 | 491 |
| PQIQAHWSNI | NDVYESSVKV | LITSQGYEQI | CKSIQLQLNI | GVEQIRVVHR |
| 501 | 511 | 521 | 531 | 541 |
| DGRVITLSHQ | EQELQDFLLS | QMSQHQVHAV | QQLAKVMGWH | VLSFSNHVGL |
| 551 | 561 | 571 | 581 | 591 |
| GSVESIGNAS | AITVASPNGE | FAISVRNGPE | SGCKVIVQFP | RSQCKDLPKS |
| 601 | 611 | 621 | 631 | 641 |
| DVIQDNKWNH | LRGPSKEVHW | NKMEGRNFVY | KMELLMAALT | PCP |

**Legend:**

**The selection scale:**

1 2 3 4 5 6 7

Positive selection Purifying selection

**Figure S2**

**Gene: *med24***

| 1 | 11 | 21 | 31 | 41 |
|---|---|---|---|---|
| MKVVNLKQAI | LQAWKERWSD | FQWAINIKKN | FPKGATWDYL | NLAEALLEQA |
| 51 | 61 | 71 | 81 | 91 |
| MIGLSPNPLI | LSYLKYAISS | QMVSYSSILT | AVSKFDDFSR | ELCVKSLLEI |
| 101 | 111 | 121 | 131 | 141 |
| MDMFSHQLSC | HGKAEECIGL | CRSLLAALVW | LLQGCAWYSA | RLREQGEQAG |
| 151 | 161 | 171 | 181 | 191 |
| EASLRACVDR | LEGLLRGTKN | RALIHIARLE | EQASWTNVEQ | AVIRVTENLN |
| 201 | 211 | 221 | 231 | 241 |
| SLTNQVLRSK | LEECLSLVKS | IPLMLVVQAE | PPQRTSFPSV | HTLVMLEGTM |
| 251 | 261 | 271 | 281 | 291 |
| NLTGETQPLV | EQLMMIKRMQ | RIPSPLFVLE | IWKACFTGLI | ESPEGTEELK |
| 301 | 311 | 321 | 331 | 341 |
| WTAFTFLKIP | QVLLRLKKYP | QGEKDFMEDV | NIAFEYLLKL | TPLLDKADQR |
| 351 | 361 | 371 | 381 | 391 |
| CNCDCIELLL | QECRKLGLLS | EANTSNLSTK | RAVDREHAPR | LKTAENANIQ |
| 401 | 411 | 421 | 431 | 441 |
| PNPGLILRAE | PTVTNILKTV | DADHSKSPEG | LLGVLGHMLS | GKSLDLLLAA |
| 451 | 461 | 471 | 481 | 491 |
| AAATGKLKSF | ARKFIKLNEF | PKHISGEGSK | PASVRALLFD | ISFLMLCHVV |
| 501 | 511 | 521 | 531 | 541 |
| QTYGSEVILS | DPSPSGETPF | FETWLQTCMP | EDGKILNPDH | PCFRPEPGKV |
| 551 | 561 | 571 | 581 | 591 |
| ENLVALLNNS | SEMKLVQMKW | HEICLSTPAA | ILEVLNAWEN | GVLTVESVQQ |
| 601 | 611 | 621 | 631 | 641 |
| KITDNIKGKV | CSMAICAVAW | LVAHVRMLGL | DEREKPQTMI | RQLMTPLYGH |
| 651 | 661 | 671 | 681 | 691 |
| SAENTLQFYN | ERVVIMSSIL | EHMCADVFQQ | TGVVLRPPME | GLEPVPYRNL |
| 701 | 711 | 721 | 731 | 741 |
| LAPREPIRAA | LSHQFSEVLQ | RGWVDSQALH | LFESLLHMGG | VFWFTNNLVK |
| 751 | 761 | 771 | 781 | 791 |
| ELLRGTRQEW | VMCAVELLYS | IFCLDMQQIT | LTLLGQILPS | LLTDSARWHS |
| 801 | 811 | 821 | 831 | 841 |
| LADPPGKALA | KLAVWCALSS | YSTHHKGQAS | ARQRKRQRED | IEDYNSLFPL |
| 851 | 861 | 871 | 881 | 891 |
| DDTQPSKLMR | LLSSNEDDPV | ILSSPGDRSM | SSSLSASQLH | TVNMRDPLNR |
| 901 | 911 | 921 | 931 | 941 |
| VLANLFLLVS | SILSSKTAGP | HTQFVQSFME | ECVECLEQGS | RGSILQFMPF |
| 951 | 961 | 971 | 981 | |
| TMVSELVKLT | ALAKPKVVLA | ITDLSLPLGR | RVAAKAIAAL | |

**Figure S2**

**Legend:**

**The selection scale:**

| | | | | | | |
|---|---|---|---|---|---|---|
| 1 | 2 | 3 | 4 | 5 | 6 | 7 |

Positive selection    Purifying selection

**Figure S2**

**Gene: *med30***

```
1          11         21         31         41
MATPPVAGAG MPAGAFGGQQ AQAAREVNTA SLCRIGQETV QDIVFRTMEI
51         61         71         81         91
FQLLRNMQLP NGVTYHASTH QDRLGKLQEH LRQLSVLFRK LRLVYDKCNE
101        111        121        131        141
NCAGLDPVPA EQLIPYVEDD GSKHDDRSTN LARYATEERR EVLEVNQKLK
151        161        171
QKNQQLKQIM DQLRNLIWEI NSMLAVRN
```

**Legend:**

**The selection scale:**

| 1 | 2 | 3 | 4 | 5 | 6 | 7 |
|---|---|---|---|---|---|---|

Positive selection    Purifying selection

**Figure S2**

**Gene: *ncoa1***

1 MSGLGESALD 11 PTNLDSLKRK 21 GSPCDTSGQS 31 VEKRRRELEC 41 RYIEELAELL

51 SANMGDIDSL 61 SVKPDKCHIL 71 KSTVDQIQQI 81 KRREQEKAAA 91 ALMSPDDEVQ

101 KSDISSSSQG 111 MIEKEALCPL 121 LLEALDGFFF 131 VVNREGRIVF 141 VSENVTNYLG

151 YPQEELMSSS 161 VYSILHVGDH 171 NEFVRNLLPK 181 SLVNGVPWPQ 191 ESTGRNSHTF

201 NCRMLKRPPD 211 EVDSENQEAR 221 QQYEIMQCFT 231 VSQPRAMHEE 241 GEDLQSCLIC

251 IACRLPRPQL 261 PVSTESFITR 271 QDPTGKIISI 281 ETSALRATGR 291 PGWEDLVRKC

301 IYAFFQPQGK 311 EPSYAKQLLQ 321 EVISHGTAIS 331 PVYRFTLNDG 341 TTLSAQTRCK

351 LCYPPNQDMQ 361 PFIMGIHTID 371 REHNTASSQE 381 NTNTGPLSTH 391 GSQSPAQPTR

401 SPAVQPSMEL 411 GQTAGMGVHL 421 NNGNSSGTGP 431 ATPTSHPPGY 441 LTPSRMGTQQ

451 LNSPSPLGSP 461 LGTTPTSFMS 471 PRPPRGSPGL 481 GGSPRVSGNP 491 FSPSTPSLHS

501 PAGAPSGSGN 511 LSRQQSGCHG 521 ECAGTPLSFS 531 TPSPVPQRQT 541 STPASSPVRP

551 PLAKPAEGLP 561 ESREDHTKTP 571 QQLSNAKLGQ 581 LLDGCGGRPS 591 ETDTPPSLPA

601 PCPASHSSLT 611 ERHKILHRLL 621 QDNSPTEPTE 631 GGRKDMEIKK 641 EPSASSPSSR

651 EPQDHQLLRF 661 LLDTDDKDLG 671 GLPPSSALSL 681 QTVRVKAGKT 691 PEAESSASPK

701 PNDRPRNQVA 711 LFPSMPELKH 721 HSGFLRLEKK 731 IQSVISFKYP 741 LFVILQVSDS

751 TYHLFQPSLQ 761 LNPATLLYYF 771 FTYLRIREVL 781 AAADLDTVSQ 791 LLHTLAGGPG

801 VKLPEEQGDS 811 PQPGESSLPR 821 GVSVKQEPSS 831 TPGRGFSDGP 841 RLQSQSPFEF

851 CSPSTPSQGQ 861 GDHFLSPKGS 871 SPFRESGHVN 881 TFRTDTGLPK 891 MELTGSQQFH

901 PPPMAEPMPF 911 EGNMGSVNDS 921 ALTVPPEQCI 931 PCPLDEMLCP 941 PTTVEGRNDE

951 KALLDQLVSF 961 LSGTDESELA 971 ALDRALGIDK 981 LVQGCCLDSL 991 SQQFPAQAPM

1001 1011 1021 1031 1041

**Figure S2**

LMDPKPPGYT SQFPPGSPQF SSAMGRAQGL GFGGPRGAFP GGIGMGPRPP
1051 1061 1071 1081 1091
LARPPGLPNQ LRLQLQQRLQ GQQQLMQQNR QAQMGQFPGA VPVAMGMRPS
1101 1111 1121 1131 1141
MQQPQLPSQQ PPLNAQMLAQ RQRELYSIQH RQRQLLQQKA MLMRQQGFAG
1151 1161 1171 1181 1191
GPMGVARIPK GPQQQQQFTF PPGYSPAAGN PPNSPSPFTP MGGPPDPTLP
1201 1211 1221 1231 1241
ARGPMANQGL MGNVQGQFGG AMNSPVQPSI FQQFGGPGMV QQGEPPFPPA
1251 1261 1271 1281 1291
LSPTSPLLSP QISTSQSPML QQTQPPPGYQ SPDLKNWQQG SMGNNSMYNQ
1301 1311 1321 1331 1341
SGQTPPQAFG QQGVYNNMSI TVSMAGGTGG VGSIPSMGPP VGMGNSNMGS
1351 1361 1371 1381 1391
VSSMCNDQVQ QVQVFADVQC TVNLVGSDPY LNQSGPLGSQ KGGAGAQTTP
1401 1411
SQQKSLLQQL LTE

**Legend:**

**The selection scale:**

1 2 3 4 5 6 7

Positive selection Purifying selection

 

**Figure S2**

**Gene: *ncoa3***

| | | | | |
|---|---|---|---|---|
| 1 | 11 | 21 | 31 | 41 |
| MSGLGDNSLD | PLCSDRKRKL | STCDTPGLGC | DKRRREQESK | YIEELAELIS |
| 51 | 61 | 71 | 81 | 91 |
| ANLSDIDNFN | VKPDKCAILK | ETVRQIRQIK | EQGKASSNDD | DVQKADVSST |
| 101 | 111 | 121 | 131 | 141 |
| GQGVIDKDHL | GPLLLQALDG | FLFVVNRDGS | IVFVSDNVTQ | YLQFKQEELI |
| 151 | 161 | 171 | 181 | 191 |
| NTSVYNILHE | EDREEFHKNL | PKSNMNGVSW | ATEAPRQKSH | TFNCRMLMKY |
| 201 | 211 | 221 | 231 | 241 |
| GHSPPEEGAG | SPRYETMQCF | ALTQPKAMME | EGDDLQSCMI | CVARRITAVE |
| 251 | 261 | 271 | 281 | 291 |
| RTESFITKHE | LSGKLIQIDH | SSLRSTMRPG | WEDLLRRCIQ | MFMHHSEGQP |
| 301 | 311 | 321 | 331 | 341 |
| WSHKRHYQEA | FMQGHAETPL | YRFSLSDGTP | VTAQTKSKLY | RNPMTNEPQG |
| 351 | 361 | 371 | 381 | 391 |
| FISTHLLQRE | QNGYRANQGP | MIQGMRPMGN | STPNASMNMP | PGPGMGMGGN |
| 401 | 411 | 421 | 431 | 441 |
| RGFSMNESGH | MGQMGGSMYG | ATNRMMQMNP | MSQMSQMGQM | SQMNHPGPGM |
| 451 | 461 | 471 | 481 | 491 |
| QQQPPYQGSG | YGLGMNSPSQ | GSPGMNVPQQ | NLMVSPRTRG | SPKMVPSQFS |
| 501 | 511 | 521 | 531 | 541 |
| PGGMHSPMGP | GSAGGAGGGG | NSNFSSSSLN | ALQAISEGVG | NPMPSSLSSP |
| 551 | 561 | 571 | 581 | 591 |
| AHKPDSSPSI | NSSQQQQQQN | QQCKAGRVDS | PSPAGMYVPG | GDHHHHLHHH |
| 601 | 611 | 621 | 631 | 641 |
| HHTPTESATD | RPDSQASLRV | TKEGSEVGTG | GAEPQRRLSD | SKGNKKLLQL |
| 651 | 661 | 671 | 681 | 691 |
| LTSPTDDLGM | VAGGVPPTGA | STPSTLEPKE | PAGCVTSPSS | TGVSSSSSSS |
| 701 | 711 | 721 | 731 | 741 |
| SSAAQPPGGV | SSSSSSAHHA | ASLQEKHKIL | HKLLQNGNSP | DEVAKITAEA |
| 751 | 761 | 771 | 781 | 791 |
| TGKETSSHEA | GVADLGTIGT | AGTGGGGVGG | SGVPDIKQEQ | PSPKKTHALL |
| 801 | 811 | 821 | 831 | 841 |
| HYLLNNDPKE | PADIKPKLEE | LEGKTQQNAC | SSSGLMPSTP | ENGENKIKTE |
| 851 | 861 | 871 | 881 | 891 |
| QPDELHDTLE | TILGGFRNSS | SSFYQESGVG | AGSDVANKQP | ACPDDAMHGL |
| 901 | 911 | 921 | 931 | 941 |
| RSPAGLRSPE | LGPRGPFQRA | VSVDGKPPVG | ANSLGRRSAP | CPMLVKQESM |
| 951 | 961 | 971 | 981 | 991 |
| DNQRMMGGPE | NFPGNMGMVN | RGLGVPQRSP | MGGSGEWGIQ | RSNASPVGTS |
| 1001 | 1011 | 1021 | 1031 | 1041 |

**Figure S2**

SHPMVRPGMD YSSKPMMGGP MVGRSNSVPG NRSMLQQQLM DMGPNDINMG
1051 1061 1071 1081 1091
MTPFNQQGPP NQSPSWPENV MPIEHGPQNS QSRRQFANPL DDLLVPPATS
1101 1111 1121 1131 1141
EGQSDERALL DQLDSLLNNT DVMALEEIDR ALGIPDLVSQ GQGVDPQSEP
1151 1161 1171 1181 1191
FPGQDPALVM DQKPPMYSQG YPGPPGMAMQ PAYSSPMQGQ PPGFNPMMNQ
1201 1211 1221 1231 1241
MSQQGNFPMA GMHPRPNMIR PRMMNALKPL RQQLVQRLQG QQFMNQSRQG
1251 1261 1271 1281 1291
LGMKMDNTTA GNPGLRPALQ APMGGQPGFL NAQMMAQRNR EMLNHQLRRQ
1301 1311 1321 1331 1341
RMMMLMQQQQ QAAAGGFSPP PNVTAPAGMD TPMGGPPMTQ PGPQQFSYAA
1351 1361 1371 1381 1391
NYGINQQGDP SFVRAAGSPP NSIMSARLGP PQNPMMQQHP QGGPMYPTAD
1401 1411 1421 1431 1441
MKSWPQGGMP RSSSYPQQQF PQQGNPGSYG NMMMNGSLPV NGNGGHMGQM
1451 1461 1471
PGQMNMNPMA MGRMPMGPEQ KYC

**Legend:**

**The selection scale:**

| 1 | 2 | 3 | 4 | 5 | 6 | 7 |
|---|---|---|---|---|---|---|

Positive selection Purifying selection

**Figure S2**

## Gene: *ncoa4*

```
1          11         21         31         41
MSSAVEDRDR DSLRRCLQAR CQLEASISGV TRAELQLRDN SREVKAQIHS
51         61         71         81         91
CISRHLEVLR SREVWLLEQI EIVQHLKEEA LQQQHHQLHC LLGQLNFLIY
101        111        121        131        141
QLENNNSNNL ANQITSCLEK LSSLNLKPEE TPEMSFQADI RSLRQAITSF
151        161        171        181        191
GTIETKQLSY YLISPPSKSS LDDVWLFQNC PLAYTQPKVE SKLWTELDNW
201        211        221        231        241
ILKSSSGSSP QSGCQLSFNL DDWLYKPKEK SQVSCPFAQF EFDKMCTKTD
251        261        271        281        291
VEAWVLKDRV PPRERSVSET SSSTMMSIEK IEDADLNLSE GEEGQETEDL
301        311        321        331        341
KDWLVTSPTE EKEELAENKS HADDKPKTMF WPFSEPFRAS DWLVKYDDSC
351        361        371        381        391
GACRGQSKAV EIENLGALKC LKHSPSPSPA ATSSTETVRV EQVCKANEPC
401        411        421        431        441
GSFLDCVCEE NCGREALCGL LFKKEGRDKN GMPLDKNKVL EESYVNDIDR
451        461        471        481        491
WLHPRKKEVP ASSFPSYGLT LQPQEQEKTT QEEPELKCKD PFKVSFNPED
501        511        521        531        541
WLMPRRKPSL PAPCTNTEED KWLLRKRAPV AEQNGPTSFS DLFACMTLKG
551        561
DMEKWLYKAP IQHILPDH
```

**Legend:**

**The selection scale:**

| 1 | 2 | 3 | 4 | 5 | 6 | 7 |
|---|---|---|---|---|---|---|

Positive selection — Purifying selection

**Figure S2**

**Gene: *nkx3.1***

1 MSASVKPLTS 11 FLIQDILAFK 21 EESRLSTPRY 31 ACAKEPRERD 41 KSPDSISAGR

51 DTRHARETGS 61 HDKNTSTDLK 71 AVGNGEETPQ 81 SSSSGGKQKR 91 SRAAFTHLQV

101 LELEKKFSHQ 111 KYLSAPERAH 121 LAHSLRLTET 131 QVKIWFQNRR 141 YKTKRKQLAT

151 EYGKDPFKRA 161 TGLPCVGTEE 171 DLIRASLFAT 181 MYKTYHDQQP 191 CLYGLNVWNS

201 AV

**Legend:**

**The selection scale:**

| 1 | 2 | 3 | 4 | 5 | 6 | 7 |
|---|---|---|---|---|---|---|

Positive selection — Purifying selection

Figure S2

**Gene: *nrip1***

1 11 21 31 41

MTHGEEPGSE MHQDSAVLTY LEGLLMHQVS GRQSAAATRR SDAGHSERNQ

51 61 71 81 91

NNKTVGSHQL PNHSSNQEDR SVPLGRATQH LKKARLLRSS EAWNGPETQQ

101 111 121 131 141

LPASVVNLNG QNRDLLTGAL DSSPKCKAES TLLASLLQSF SSRLQSVALS

151 161 171 181 191

QQIMQNLKQQ DNLQSDKSTQ VEDEALRCYG SASSRLKGLL KKNKMQNHNS

201 211 221 231 241

VPYQRRNSQE RFSDSPEASQ SSVQPAARDS ISCAARLKAV ASIVKNRSSP

251 261 271 281 291

TSSPKPSVAC SQLALLLSSE AHLQQYSREQ ALKAQLSGRS ASERLAAMAT

301 311 321 331 341

QQTQDIKQSS MGQHQVSADI VNPLNGQNGT LPQTVDSSKP SPSPIQGQSR

351 361 371 381 391

VNSSQRSLHS FRDKHSFDRH SSRPSPNCSS LLLHLLNNHN TQKYTNGNSL

401 411 421 431 441

MEEDYSVFPN HSSPLRSESE YSNLENSLTK DNSDAESSHS SCSPIDLSVK

451 461 471 481 491

GRASGSGLGS SSSLDKLTET LISNWNPETS SHKVTEARES ENSSVIKPHH

501 511 521 531 541

KVTLLQLLLG HKNNEKVNKN SDNPDLQSCT TSKPNCLPTG RVTPSSRFEE

551 561 571 581 591

TRTRNSPDAL CFRKLQSLPV FSQEQDTNGS ASPYSLSLSP QVQAIPLDLC

601 611 621 631 641

KAKSHSNENV EESSFSASKL LQNLAQCGLQ KSIPSPPVET SVSPGIRQTY

651 661 671 681 691

EPRTDKPVAL LERLNAPLTK NKTTVLEEPL VNSMKLPYVM DPSPSVSEIE

701 711 721 731 741

NLLERRTVLQ LLLGATTSKE KASGKRKRPV DKGDSLDKHS DPSPGSGNSY

751 761 771 781 791

EPTLDIKIKT EPRDEVHLSN TNGEEKRSQV EERLNGGNNP HSSNQKDIKS

801 811 821 831 841

EVLSAEAIPK DGLLSQLLKH PPSTYQVKNQ DVCTISGKEG LSLNQGPAIP

851 861 871 881 891

KKRKLCMGMD ETLNTEPCIR AVSAVQRDGS SEPSGSRTSD GRNVHNEVNR

901 911 921 931 941

LEADSLLQVG CPVSESLPKD GKGFNVLKQL LLSDNCLKDI SLPRSATSPS

951 961 971 981 991

IIQANCKING NIPSKSGYNH DFAVLQHNSS PQGPVSLDFK PLSAASERSK

1001 1011 1021 1031 1041

**Figure S2**

PPGSPWGYHA ARQDSPKLNL IPVKRELEGP IQWVISGEEK QDPSIDSPRL

1051 1061 1071 1081 1091

TKSNPILYYM LQKGNSHIGK LRKAEAPECE MQVKVKEEPM SELDAYSHRL

1101 1111 1121

RLKHHHQLSN EKQGNETESF NRTVEKC

**Legend:**

**The selection scale:**

1 2 3 4 5 6 7

Positive selection Purifying selection


T. Lorin, W. Salzburger & A. Böhne

**Figure S2**

## Gene: *pias1*

| 1 | 11 | 21 | 31 | 41 |
|---|---|---|---|---|
| MVMSLRVSEL | QVLLGYAGRN | KHGRKHELLT | KALHLLKAGC | SPAVQMKIKE |
| 51 | 61 | 71 | 81 | 91 |
| LYRRRFPTKM | VTPAELSLPS | VHSTTAPLPA | GLPQLSFDGH | AASSPLLPVS |
| 101 | 111 | 121 | 131 | 141 |
| LLGPKHELDL | PHLPSALHPV | HPDVKLQKLP | FYDMLDELIK | PTSLASDNSQ |
| 151 | 161 | 171 | 181 | 191 |
| RFQETCFAFA | LTPQQVQQIS | SSIFLSQQIR | HITFQDTLKF | CLSETSCPQE |
| 201 | 211 | 221 | 231 | 241 |
| DHFPPNLCVK | VNGKPCNLPG | YLPPTKNGVE | PKRPSRPINI | TSLVRLSTTV |
| 251 | 261 | 271 | 281 | 291 |
| PNTIVVSWTA | EIGRSYSMAV | YLVKQQSSSV | LLQRLRSKGI | RNPDHSRALI |
| 301 | 311 | 321 | 331 | 341 |
| KEKLTADPDS | EIATTSLRVS | LLCPLGKMRL | MIPCRALTCS | HLQCFDATLY |
| 351 | 361 | 371 | 381 | 391 |
| IQMNEKKPTW | VCPVCDKKAP | YEHLIIDGLF | MEILNSCVDC | DEIQFKEDGS |
| 401 | 411 | 421 | 431 | 441 |
| WAPMRSKKEV | QQVNSYNGVE | GACRTAIPEH | RHSSSSNSKK | VEVIDLTLDS |
| 451 | 461 | 471 | 481 | 491 |
| SSDEEEDEEP | PPKRTCPSLS | PASPPVNKGV | LNLHHQASPV | ARAPSMPPVD |
| 501 | 511 | 521 | 531 | 541 |
| TNYIPPPPPL | IQDYRHSYHT | LPYELQGLDF | FPFLQGDNQH | YSTNLLVAAA |
| 551 | 561 | 571 | 581 | 591 |
| AAASASDEHD | MLHPSLNRFL | PYNSSQMYLE | QPGTPVSSAL | VPANGGGSSS |
| 601 | 611 | 621 | 631 | 641 |
| SSSSSLVSSS | SLRESHGHPG | PTRATAEAAA | AIYGSMPDVI | SLD |

**Legend:**

**The selection scale:**

| 1 | 2 | 3 | 4 | 5 | 6 | 7 |
|---|---|---|---|---|---|---|

Positive selection    Purifying selection

**Figure S2**

**Gene: *pias2***

| 1 | 11 | 21 | 31 | 41 |
|---|---|---|---|---|
| MTLTQPAPPI | PPVHPDVQLK | SLPFYDVLDV | LIKPSSLGAS | SIQRFQEKYF |
| 51 | 61 | 71 | 81 | 91 |
| IFALTPQQVR | EVCISRDFLP | GGRRDYMVQI | QLRFCLAETS | CPQEDNYPNS |
| 101 | 111 | 121 | 131 | 141 |
| LCIKVNGKPF | PLPGYPPPPK | NGVEQKRQGR | PLNITSSVRL | SSAVPNQISV |
| 151 | 161 | 171 | 181 | 191 |
| TWAPEIGKTY | SMSVYLVRQL | TSSLLLQRLK | MKGIRNPDHS | RALIKEKLTA |
| 201 | 211 | 221 | 231 | 241 |
| DPDSEIATTS | LRVSLMCPLG | KMRLTVPCRA | VTCSHLQCFD | AALYLQMNEK |
| 251 | 261 | 271 | 281 | 291 |
| KPTWICPVCD | KKAAYESLII | DGLFMEILND | CTDVDEIKFQ | EDGSWCPMRP |
| 301 | 311 | 321 | 331 | 341 |
| KKETLKVSAQ | CSTKIETSDP | SSLKKADIID | LTIESSSEGE | EEEEEIPPLK |
| 351 | 361 | 371 | 381 | 391 |
| KRCLYMSKPE | EVHGKGVLTY | QPSTVRVPNV | QTVETSYITS | PLADYAVPFH |
| 401 | 411 | 421 | 431 | 441 |
| HSTLSTISAD | MQISETDAQW | DSGRYRTPMF | LDNLPSTLQQ | QATSGGLIST |
| 451 | 461 | 471 | 481 | |
| SSHYESSSHS | SGSARNETGV | ITGGGGTIIP | DIISLD | |

**Legend:**

**The selection scale:**

1 2 3 4 5 6 7

Positive selection    Purifying selection

**Figure S2**

## Gene: *pik3r1*

| | | | | |
|---|---|---|---|---|
| 1 | 11 | 21 | 31 | 41 |
| MSAEGYQYRA | LYDYKKEREE | DIDLHLGDIL | IVSKGALLAL | GYGDGFEEKP |
| 51 | 61 | 71 | 81 | 91 |
| DEIGWLHGFN | ETTQEKGDFP | GTYVEFIGKK | RISPPTPKPR | PQRPLPVAPG |
| 101 | 111 | 121 | 131 | 141 |
| PTKADSDSEQ | ALLDLTEQFS | PPELAPPILV | KLVEAIERKG | LDCPTLYRNF |
| 151 | 161 | 171 | 181 | 191 |
| GSCSGLEIRQ | CFESDPCAVD | LDQFDLQTLC | DGLRRYLQDL | PHPVVPVSVY |
| 201 | 211 | 221 | 231 | 241 |
| SEMVHVAQEV | HNPEECALLL | RRIVQSPNVP | HQYWLTLQYL | IKHFCRVCQN |
| 251 | 261 | 271 | 281 | 291 |
| TGKNLLSARV | LGDVFSPLVF | RHQAASCDAS | PEYHIRIIEV | FITSEWNESQ |
| 301 | 311 | 321 | 331 | 341 |
| AAPALPPKPP | KPAPVTNNGM | NNNMSLQDAE | WYWGDISREE | VNEKLRDTAD |
| 351 | 361 | 371 | 381 | 391 |
| GTFLVRDAST | KMHGDYTLTL | RKGGNNKLIK | IFHRDGKYGF | SDPLTFNSVV |
| 401 | 411 | 421 | 431 | 441 |
| ELINHYRHES | LAQYNPKLDV | KLLYPVSKHQ | QDQVVKEDNV | EAVGKKLHEY |
| 451 | 461 | 471 | 481 | 491 |
| HQQYQEKNKE | YDRLYEEYTR | TSQEIQMKRT | AIEAFNETIK | IFEEQCQTQE |
| 501 | 511 | 521 | 531 | 541 |
| RYSKEYIEKF | RREGNEKEIQ | RIMVNYEKLK | SRISEIVDSK | RRLEEDLKKQ |
| 551 | 561 | 571 | 581 | 591 |
| AADYREIDKR | MNSIKPDLIQ | LRKTRDQYLM | WLTQKGVRQK | KLNEWLGIKN |
| 601 | 611 | 621 | 631 | 641 |
| ETTEDQYSMV | EDDEDLPHHD | ERSWKLGNIN | RVQAEALLRG | KRDGTFLVRD |
| 651 | 661 | 671 | 681 | 691 |
| SSKQGCFACS | VVVDGEVKHC | VINKTPTGYG | FAEPYNLYNS | LKELVLHYQH |
| 701 | 711 | 721 | | |
| TSLVQHNDSL | NVTLAYPVYA | HQRR | | |

**Legend:**

**The selection scale:**

1 2 3 4 5 6 7

Positive selection Purifying selection

**Figure S2**

**Gene: *pik3r2***

1 MSAEGFQYRA 11 LYVYNKDWDE 21 DVDLQPGDLL 31 TVNKASLLAL 41 GFKEGDEERP
51 GELGWMVGVN 61 ERTKQRGDFP 71 GTYVQYVGPV 81 KMSLPLNQPR 91 FQRPLPAAPR
101 AEPPPVRQAL 111 PPKPPKTKAL 121 TNLVANGNSS 131 LLADAEWYWG 141 DISREEVNEK
151 MRDTPDGTFL 161 VRDASSKVQG 171 EYTLTLRKGG 181 NNKLIKIFHR 191 GGMYGFSEPL
201 TFLSVVELIS 211 HYRHESLAQY 221 NAKLDTRLLY 231 PVSKYQQEQV 241 VKEDSIEAVG
251 EQLRVYHEQY 261 QEKSREYDVL 271 YEEYTRTSQE 281 LQMKRTAIEA 291 FNETIKIFEE
301 QCETQERYSK 311 EYIEKFRREG 321 NDKEIQRIQS 331 NSEKLKSRVT 341 EIHDSKRKLE
351 QDLKKQATDN 361 REIDKRMNSL 371 KPDLMQLRKI 381 RDQYLVWLTQ 391 KGTRQRRINE
401 WLGITNEAED 411 LYSLMDEEDD 421 LPHHDERMWY 431 VGDVKRVQAE 441 DLLRGKRDGT
451 FLIRDSQSQK 461 GSYACSVVVD 471 GDIKHCVIYK 481 TVTGYGFAEP 491 YNLYGSLKEL
501 VLHYKHTSLV 511 QHNDSLNVTL 521 AWPVLSPQPR

**Legend:**

**The selection scale:**

| 1 | 2 | 3 | 4 | 5 | 6 | 7 |
|---|---|---|---|---|---|---|

Positive selection — Purifying selection

**Figure S2**

**Gene: *ppap2a***

| 1 | 11 | 21 | 31 | 41 |
|---|---|---|---|---|
| MFDTKGIPFV | LLDLACLVLA | GLPLAAFNLA | KIKPYRRGFF | CNDESIGYPF |
| **51** | **61** | **71** | **81** | **91** |
| HSSTVTSTTL | YTVGFTLPIC | SMVVGECLSV | YLNRLKSKSS | FSNSYVACVY |
| **101** | **111** | **121** | **131** | **141** |
| KAVGTFVFGA | AMSQSLTDIA | KYSIGRLRPH | FLDVCKPDWR | LINCSAGAYI |
| **151** | **161** | **171** | **181** | **191** |
| ENFTCTGDKT | MVNEGSSSFK | QGHNIIKLIR | LLKEILALYL | QARMQAEWAR |
| **201** | **211** | **221** | **231** | **241** |
| LLRPTIQFFL | IAASVYTGLS | RVSDYKHHWS | DVLTGLIQGA | VMAVLVVFFV |
| **251** | **261** | **271** | **281** | |
| SDFFKKPVQP | QKPVEIPHTT | LQETPTNGNH | YDSPN | |

**Legend:**

**The selection scale:**

| 1 | 2 | 3 | 4 | 5 | 6 | 7 |
|---|---|---|---|---|---|---|

Positive selection — Purifying selection

**Figure S2**

**Gene: *pmepa1***

| 1 | 11 | 21 | 31 | 41 |
|---|---|---|---|---|
| MGLTNGTTDT | LANVSCTCNC | QRSTSFQSMA | ISQLEFVQIL | VIVVVMMMMV |
| 51 | 61 | 71 | 81 | 91 |
| LVITCLLNHY | RLSARSLMSR | HTHERRRHLP | LPSEGSLWSS | DGPGSSSAMS |
| 101 | 111 | 121 | 131 | 141 |
| EVYTPRAPDR | VPSFLQRERV | SRFQPTFPFL | PPVIELPPTI | ALSDGEEPPP |
| 151 | 161 | 171 | 181 | 191 |
| YQGPCTLQLR | DREQQLELNR | ESVRPPPNRT | VYDSALTHTG | ISGGRLQGPP |
| 201 | 211 | 221 | 231 | 241 |
| PAYSEVIGHY | YHPTTLPSPC | RFRAPSAGTS | PTAVVLASAP | PLSLPNIHSS |
| 251 | 261 | 271 | 281 | 291 |
| TRMFSPKPGH | RKRPEELLPN | QLTWKILGSL | APGRCSMLSQ | WERY |

**Legend:**

**The selection scale:**

| 1 | 2 | 3 | 4 | 5 | 6 | 7 |
|---|---|---|---|---|---|---|

Positive selection    Purifying selection

**Figure S2**

**Gene: *ppargc1a***

| | | | | |
|---|---|---|---|---|
| 1 | 11 | 21 | 31 | 41 |
| MAWDRCNQDS | VWRELECAAL | VGEDQPLCSD | LPELDLSELD | VSDLDADSFL |
| 51 | 61 | 71 | 81 | 91 |
| GGLKWYSDQS | EIISSQYGSE | PSNLFEKIDE | ENEANLLAVL | TETLDSLPVD |
| 101 | 111 | 121 | 131 | 141 |
| EDGLPSFDAL | AEGDVTNTSD | QSCPSSPDGS | PPTPEAEEPS | LLKKLLLAPA |
| 151 | 161 | 171 | 181 | 191 |
| NSQLSYNQYT | GGKAQNEAPS | NQRIRPTPAV | VKTENPSWNN | KQRVPCQQQQ |
| 201 | 211 | 221 | 231 | 241 |
| KPTRRPCAEL | LKYLTASDET | IQTKACEQKS | GGGSSSKVKS | GGASSSSSSV |
| 251 | 261 | 271 | 281 | 291 |
| ASSSSSSSKK | KPVHPSQQQL | HHQRAKPTTL | PLPLTPESPN | DQKGSPFENK |
| 301 | 311 | 321 | 331 | 341 |
| SIERTLSVEL | SGTAGLTPPT | TPPHKATQEN | PFKPSVKPKL | SSCSSSAQAS |
| 351 | 361 | 371 | 381 | 391 |
| KRPHYSDSAV | CPSIKKGPEQ | TELYAQLSKV | SALPAGQEDR | KSKRSTLRLF |
| 401 | 411 | 421 | 431 | 441 |
| GDHDYCQFVS | TKRDISLAYS | VESQDGRQLE | CWDLVLSPSS | LAVQQQNYSS |
| 451 | 461 | 471 | 481 | 491 |
| PDQDLWCNRE | GKQLQTYQNR | QQANSRKPLR | DQEIRAELNK | HFGNPKQAIY |
| 501 | 511 | 521 | 531 | 541 |
| NEDEKAGPQQ | RDESDSEYYY | KLTDYVHPGL | PMEGVFDDSE | DENEKFLYPW |
| 551 | 561 | 571 | 581 | 591 |
| EGTHLDLLFG | GSPSCSPSSC | SPSRSSVSPP | KSLFSSQRCH | WSRSASHSRS |
| 601 | 611 | 621 | 631 | 641 |
| RSRSSSCHRR | RSYSRSPYSR | SRSRSPYSRS | SSRSHHCVDS | SNTRPRSRRS |
| 651 | 661 | 671 | 681 | 691 |
| PPIHSRSQSR | SPFSRRPRYD | SYEEYQHERL | KREEYRRDYE | KREFERAEQR |
| 701 | 711 | 721 | 731 | 741 |
| ERQRQKAIEE | RRVVYVGRLR | PDITRTELKR | RFEVFGEIEE | CAVNLRDDGD |
| 751 | 761 | 771 | 781 | 791 |
| NFGFITYRYT | CDAFAALENG | HTLRRSNEPQ | FELCFGGRKQ | FCKSNYTDLD |
| 801 | 811 | 821 | 831 | |
| SHSDDFDPSS | TKSKYDSMDF | DSLLKEAQRS | LRR | |

**Legend:**

**The selection scale:**

1 2 3 4 5 6 7

Positive selection — Purifying selection

**Figure S2**

## Gene: *pten*

| | | | | |
|---|---|---|---|---|
| 1 | 11 | 21 | 31 | 41 |
| M F Q W R R S C C L | A H L V I S Y I Y P | N I I A M G F P A E | R L E G V Y R N N I | D D V V R F L D S K |
| 51 | 61 | 71 | 81 | 91 |
| H K N H Y K I Y N L | C A E R H Y D A A K | F N C R V A Q Y P F | E D H N P P Q L E L | I K P F C E D L D Q |
| 101 | 111 | 121 | 131 | 141 |
| W L S E D D N H V A | A I H C K A G K G R | T G V M I C A Y L L | H R G K F L K A Q E | A L D F Y G E V R T |
| 151 | 161 | 171 | 181 | 191 |
| R D K K G V T I P S | Q R R Y V Y Y Y S Y | L L K N Q L E Y K P | V A L L F H K M V F | E T V P M F S G G T |
| 201 | 211 | 221 | 231 | 241 |
| C R D T A V K N K T | E Q I P H G F R K G | N W H W D P Q F V V | Y Q L K V K I H T S | N P A H T R R E D K |
| 251 | 261 | 271 | 281 | 291 |
| Y M F F E F P Q P L | P V C G D I K V E F | F H K Q N K M M K K | D K M F H F W I N T | F F I P G P E E N L |
| 301 | 311 | 321 | 331 | 341 |
| E K V E N G S L V G | V K E L D G I Q S T | E R G D N D K D Y L | I L T L T K N D L D | K A N K D K A N K N |
| 351 | 361 | 371 | 381 | 391 |
| F S P N F K V K L Y | F T K T V E E P S N | S E A S T S T S V T | P D V S D N E P D H | Y R Y S D T T D S D |
| 401 | 411 | | | |
| P E N E P F D E E Q | H T Q I T K V | | | |

**Legend:**

**The selection scale:**

| 1 | 2 | 3 | 4 | 5 | 6 | 7 |
|---|---|---|---|---|---|---|

Positive selection — Purifying selection

 T. Lorin, W. Salzburger & A. Böhne

**Figure S2**

**Gene: *ptk2a***

1 11 21 31 41
MAAAYLDPNL NHALGSGAKS RLSMGMERSP GALDRVLKVF HYFESNSEPS
51 61 71 81 91
TWASNIRHGD ATDVRGIIQK IVDIHKVKCV SCYGLRLSHL QSEEIHWLHP
101 111 121 131 141
DMGVSHVREK YELNHPQEEW RYELRIRYLP KGFLNQFTDD KPTLNYFYQQ
151 161 171 181 191
VRNDYMLEIA DQVDQEIALK LGCLEIRRFF REMRGNALDK KSNYELLEKD
201 211 221 231 241
VGLRRFFPKS LLDSVKAKTL RKLIQQTFKQ FANLNDEQSI LKFFEILSPV
251 261 271 281 291
YRFDKECFKC ALGSSWVISV ELAIGPEEGI SYLTDKGSTP THLANFNQVQ
301 311 321 331 341
TIQYSSSDDK DRKGMLQLNV AGAPEPLTVT TPSLTIAENM ADLIDGYCRL
351 361 371 381 391
VNGASQSFII RPQKAEGERA LPSIPKLANN EKQGVRTRTV SVSETDDYAE
401 411 421 431 441
IIDEEDTYTM PSTRDYEIQR ERIELGRCIG EGQFGDVHQG VYMSPENPSL
451 461 471 481 491
SVAIKTCKNC TSDSVREKFL QEALTMRQFD HPHIVKLIGV ITENPVWIIM
501 511 521 531 541
ELCTLGEVRT QFMGIVGKES MSLSVTLMYV VHNLLKPLNT DSSYCQSKLI
551 561 571 581 591
VVVAVAENNS RTSTSLSLLL QHLWENKYIH NNTHLNTEEL IEQLVLPVLW
601 611 621 631 641
PADRSSERDT VFAGVCMWEI LMYGVKPFQG VKNNDVIGRI ENGERLAMPP
651 661 671 681 691
NCPPTLYSLM TKCWAYDPSK RPRFTELKVQ LSTILEEEKA QQEERIRMEM
701 711 721 731 741
RRQVTVSWDS GGSDEAPPKP SRPGYPSPRS SEGFYPSPQH AVQHNHYQVS
751 761 771 781 791
GYPGSHGMSS MPSAVYPPQA SVLDPHDSWN HHRPQDIPMW SPNMEEGGAL
801 811 821 831 841
DLRGMGQGLP THLMEERLMM QQQQMEEDQR WLEQEERFLK PDPRNSRGSI
851 861 871 881 891
DREDCSLQGP MGNQHIYQPV GKPEHVAPPK KPPRPGAPSH LGSLASLNPV
901 911 921 931 941
DSYNEGVKIQ PQEISPPPTA NLDRSNDKVY ENVTGLVKAV IEMSSKIQPA
951 961 971 981 991
PPEEYVPMVK EVGLALRTLL ATVDETIPVL PASTHREIEM AQKLLNSDLA
1001 1011 1021 1031 1041

**Figure S2**

ELINKMKLAQ QYVMTSLQQE YKKQMLTAAH ALAVDAKNLL DVIDQARLKM

1051

IGQPRPH

**Legend:**

**The selection scale:**

| 1 | 2 | 3 | 4 | 5 | 6 | 7 |
|---|---|---|---|---|---|---|

Positive selection    Purifying selection

Figure S2

**Gene: *ptk2b***

| | | | | |
|---|---|---|---|---|
| 1 | 11 | 21 | 31 | 41 |
| MSGDSRTLSW | TLGSTGKGEP | SGLSERTNDT | LSVGDKIIKV | CFTSNSFNLG |
| 51 | 61 | 71 | 81 | 91 |
| KNFKLVKCDS | SWEVKEIINS | ILSSGRLGPN | ITFSGCYGLL | LKHLKSDEIY |
| 101 | 111 | 121 | 131 | 141 |
| WLHPDLTVGD | VVQKYEQRHC | EAEWRYDLRI | RYIPNNFIEK | FKEDRTTLLY |
| 151 | 161 | 171 | 181 | 191 |
| FYQQVRCDYM | QRCANKVSDG | MALQLGCLEI | SNRRFYKDMN | AKGLEKKSNF |
| 201 | 211 | 221 | 231 | 241 |
| ELLEKDVGLD | LFFPKELMDS | MKPKHLRKMI | QQTFQQYATL | KEDECIAKFF |
| 251 | 261 | 271 | 281 | 291 |
| ETLSSVSSFE | EEVYPCELVQ | GWSIAVDLVI | GPKGIRQRSN | KEAVAICLAE |
| 301 | 311 | 321 | 331 | 341 |
| FKQIRSIKCS | LQDNDRGQLH | LEITGAKQLL | SINTSNLAMA | ENMADLIDGY |
| 351 | 361 | 371 | 381 | 391 |
| CRLENGANTS | VIVRPRKDKD | FRNSLPAVPT | ADHKNMGSNT | TVRESVGSDI |
| 401 | 411 | 421 | 431 | 441 |
| YAEIPDERPK | SVIKFGISRD | DIVLGRILGE | GFFGEVHEGV | YKDKKGERIS |
| 451 | 461 | 471 | 481 | 491 |
| VAVKTCKDCS | PDVKEKFMSE | AVIMKKLDHP | HIVRLIGIIE | EDPVWIVMEL |
| 501 | 511 | 521 | 531 | 541 |
| YQYGELGNYL | TDNKHNLTTA | TLILYSLQIC | KALAYLEGVN | MVHRDIAVRN |
| 551 | 561 | 571 | 581 | 591 |
| VLVAAPECVK | LGDFGLSRYI | EDEEYYKASV | TRLPIKWMAP | ESINFRRFTC |
| 601 | 611 | 621 | 631 | 641 |
| SSDVWMFAVC | MWEIMSMGQQ | PFFWLENKDV | INQLEQGVRL | PKPDICPPTL |
| 651 | 661 | 671 | 681 | 691 |
| YTLMTRCWTY | DPQERPKFTE | LVCKLSDMFK | MEKEQETLQQ | SKNRSRATKF |
| 701 | 711 | 721 | 731 | 741 |
| FDPIITVSEP | PPKPSRMKSS | RFGSTLNVGL | QIQLPESLCA | SSPAIASPPD |
| 751 | 761 | 771 | 781 | 791 |
| YQTPVDSNNR | LLLPRQVPRR | RSMGEGDFIV | EPVSKEDAQR | LWEIEKARMQ |
| 801 | 811 | 821 | 831 | 841 |
| ETLKRQKQEM | IEDNKWLEKE | EKLLDPMIQE | GSKTSEVPEK | ESGYAQFTGP |
| 851 | 861 | 871 | 881 | 891 |
| PEKPPRLTAQ | QPAPTAEMDR | SEDKVYHFVM | ELVKVVVQLK | NDVNVLPASE |
| 901 | 911 | 921 | 931 | 941 |
| YVNVVKSVGL | TLRDLIRSVD | EVLPTLHASK | RTEIEGTQKL | LNKDMAELIS |
| 951 | 961 | 971 | 981 | 991 |
| KMKLAQQNAI | TSLSEECKRQ | MLAAAHTLAM | DSKNLLDAVD | QARVRANTAK |
| 1001 | | | | |

**Figure S2**

PSAS

**<u>Legend:</u>**

**The selection scale:**

| 1 | 2 | 3 | 4 | 5 | 6 | 7 |
|---|---|---|---|---|---|---|

Positive selection Purifying selection

**Figure S2**

**Gene: *rac1***

| | | | | |
|---|---|---|---|---|
| 1 | 11 | 21 | 31 | 41 |
| MQAIKCVVVG | DGAVGKTCLL | ISYTTNAFPG | EYIPTVFDNY | SANVMVDGKP |
| 51 | 61 | 71 | 81 | 91 |
| VNLGLWDTAG | QEDYDRLRPL | SYPQTDVFLI | CFSLVSPASF | ENVRAKWYPE |
| 101 | 111 | 121 | 131 | 141 |
| VRHHCPNTPI | ILVGTKLDLR | DDKDTIEKLK | EKKLTPITYP | QGLAMAKEIG |
| 151 | 161 | 171 | 181 | 191 |
| AVKYLECSAL | TQRGLKTVFD | EAIRAVLCPP | PVKKRRRKCR | LF |

**Legend:**

**The selection scale:**

| 1 | 2 | 3 | 4 | 5 | 6 | 7 |
|---|---|---|---|---|---|---|

Positive selection    Purifying selection

Figure S2

**Gene: *raf1***

| | | | | |
|---|---|---|---|---|
| 1 | 11 | 21 | 31 | 41 |
| MEHIQGAWKT | ISNGFGLKES | VFDGQCMSPT | IVQGFPYQRR | SSDDGKIPDP |
| 51 | 61 | 71 | 81 | 91 |
| SKASSTIRVY | LPNKQRTVVN | VRPGVTLHNC | LIKALKVRGL | QPECCAVFRL |
| 101 | 111 | 121 | 131 | 141 |
| HPGQRSRKSR | MDWNTDSTSL | IGEELLVEVL | DHVPLTTHNF | VRKTFLKLAF |
| 151 | 161 | 171 | 181 | 191 |
| CDICQKFLLN | GFRCQTCGYK | FHEHCSTKVP | TMCVDWSNIR | QLLLFPTPGE |
| 201 | 211 | 221 | 231 | 241 |
| SGAPSLPPLT | SRRMRDSLSR | FPPSSQHRHS | TPHAFNYITP | YPPTGGALSQ |
| 251 | 261 | 271 | 281 | 291 |
| RQRSTSTPNV | HMVSTTMPVD | SSIIEDAIRS | HESGSPTSQS | PTGWSQSKTP |
| 301 | 311 | 321 | 331 | 341 |
| VPAHRERAPA | SNTQEKNKIR | PRDKRDSSYY | WEIEANEVML | LSRIGSGSFG |
| 351 | 361 | 371 | 381 | 391 |
| TVYKGKWHGD | VAVKILKVTD | PTPEQFQAFR | NEVAVLRKTR | HVNILLFMGY |
| 401 | 411 | 421 | 431 | 441 |
| MTKDNLAIVT | QWCEGSSLYK | HLHVQETNFQ | MFRLIDIARQ | TAQGMDYLHA |
| 451 | 461 | 471 | 481 | 491 |
| KHIIHRDMKS | NNIFLHEGLT | VKIGDFGLAT | VKARWSGSHQ | VEQPSGSILW |
| 501 | 511 | 521 | 531 | 541 |
| MAPEVIRMQD | NNPYSFQSDV | YSYGIVLFEL | MTGELPYSHI | ASRDQIIYMV |
| 551 | 561 | 571 | 581 | 591 |
| GRGYLSPDLS | KLYKSCPKAM | KRLVADCIKK | SKDERPLFPQ | ILSSIELLQH |
| 601 | 611 | 621 | 631 | |
| SLPKINRSAS | EPSLHRASHT | EDINSCTLTS | MKLPVF | |

**Legend:**

**The selection scale:**

| 1 | 2 | 3 | 4 | 5 | 6 | 7 |
|---|---|---|---|---|---|---|

Positive selection Purifying selection

 T. Lorin, W. Salzburger & A. Böhne

**Figure S2**

**Gene: *ran***

```
1          11         21         31         41
XXXXXXXXXX XXXXXXXXXX XXXXXXXXXX XXXXXXXXXA TLGVEVHPLV
51         61         71         81         91
FHTNRGAIKF NVWDTAGQEK FGGLRDGYYI QAQCAIIMFD VTSRVTYKNV
101        111        121        131        141
PNWHRDLVRV CENIPIVLCG NKVDIKDRKV KAKSIVFHRK KNLQYYDISA
151        161        171        181        191
KSNYNFEKPF LWLARKLIGD PNLEFVAMPA LAPPEVSMDP SLAAQYEHDL
201        211
KVASETALPD EEDDL
```

**Legend:**

**The selection scale:**

| 1 | 2 | 3 | 4 | 5 | 6 | 7 |
|---|---|---|---|---|---|---|

Positive selection    Purifying selection

**Figure S2**

**Gene: *rb1***

1 MPPKRRSAAP 11 SAQLSKAAED 21 KHTNRVGVPG 31 TPGRIPQLSL 41 ERHKDKEPPF
51 VSLCQELRVT 61 DEVCNLAWKI 71 WETMVASVDI 81 VLLLGIKDTS 91 RKHWGACIFV
101 AATDLDAMTF 111 TITEVQKTVG 121 LNVVEFFNLL 131 KKMDINMDTI 141 SVKVNTAVSR
151 LEKKYEVLLA 161 LFQRFERTCG 171 KIYIAVSDNA 181 GNAGPDAKQI 191 MRSCWTLFLL
201 AKGNVLQMED 211 DLVISFQLLL 221 CVLEYFIKRS 231 PPTVLQEPFK 241 IAANGVHSTT
251 PTRTSRRGLG 261 RGARAAPKPA 271 DTDLRVMEAL 281 CKENECTVDE 291 VKNVYFTSFV
301 AFLDSVGLAG 311 SQEIPESEDL 321 SKQYEELYFK 331 SKDFDARLFL 341 DHDETLQPVK
351 TDVSQLERTP 361 RKNPLEEEVL 371 PIPPQTPVRA 381 AMNCIQQLKV 391 ILNSASDWPS
401 ENLVKYFSNC 411 TVNPSEEITE 421 RVVNMRELFI 431 DRFARAVGQQ 441 CIEIGSQRFV
451 LGVRLYYRVM 461 ESMLRSEEER 471 LSIQNFSKLL 481 SNADFHTSLL 491 ACALEVVMAT
501 YDWSTFKSSS 511 SSSETDLSFP 521 WILDVFKLKA 531 FDFYKVIESF 541 IKAEPSLTRE
551 MIKHLERCEH 561 LIMESIAWRS 571 DSPLFELLRQ 581 SREQEGAVEQ 591 AEPPGMLNQL
601 LQHDHTAADL 611 YLSPVRSPWK 621 KSQSVASSAS 631 QGADAQTGPS 641 SQSQTSSHKH
651 LKSTSLSLFY 661 KKVYRLAYLR 671 LNMLFSHLLK 681 DHPELEHIIW 691 TLFQHTLQHE
701 HELLKDRHLD 711 QIMMSAMYAI 721 CKVKNIDLRF 731 KTIVTAYKEL 741 PNTHQETFKH
751 VLIREGQYDS 761 IIAFYNQVFM 771 QKLKTNILQY 781 ASPRPPTLSP 791 IPHIPCSPYK
801 FSNSPLRVPG 811 SNNVYVSPLK 821 SARMSPSVMT 831 PRSRILVSIG 841 ESFGTSEKFQ
851 RINQMVSSSD 861 WSLKRGLESN 871 SAPKPLKRLR 881 FDTDGQDEAD 891 GSKSAGESKF
901 QQKLAEMTST 911 RTRMQEQKLK 921 DAAVDSLKEE 931 KH

**Legend:**

**The selection scale:**

1 2 3 4 5 6 7

Positive selection — Purifying selection

**Figure S2**

**Gene: *rhoaa***

| 1 | 11 | 21 | 31 | 41 |
|---|---|---|---|---|
| MAAIRKKLVI | VGDGACGKTC | LLIVFSKDQF | PEVYVPTVFE | NYVADIEVDG |
| 51 | 61 | 71 | 81 | 91 |
| KQVELALWDT | AGQEDYDRLR | PLSYPDTDVI | LMCFSIDSPD | SLENIPEKWT |
| 101 | 111 | 121 | 131 | 141 |
| PEVKHFCPNV | PIILVGNKKD | LRNDEHTRRE | LAKMKQEPVK | PEEGRDMANR |
| 151 | 161 | 171 | 181 | 191 |
| INAFGYLECS | AKTKDGVREV | FEMATRAALQ | AKKRGKKNAC | LLL |

**Legend:**

**The selection scale:**

| 1 | 2 | 3 | 4 | 5 | 6 | 7 |
|---|---|---|---|---|---|---|

Positive selection    Purifying selection

**Figure S2**

**Gene: *rhoab***

```
1          11         21         31         41
MAAIRKKLVI VGDGACGKTC LLIVFSKDQF PEVYVPTVFE NYVADIEVDG
51         61         71         81         91
KQVPLNALKM EIYDRLRPLS YPDTDVILMC FSIDSPDSLE NIPEKWTPEV
101        111        121        131        141
KHFCPNVPII LVGNKKDLRN DEHTRRELAK MKQEPVKPEE GRDMANRIGA
151        161        171        181
FGYMECSAKT KDGVREVFEM ATRAALQARR GKKTSKCLLL
```

**Legend:**

**The selection scale:**

| 1 | 2 | 3 | 4 | 5 | 6 | 7 |
|---|---|---|---|---|---|---|

Positive selection Purifying selection

 T. Lorin, W. Salzburger & A. Böhne

**Figure S2**

**Gene: *rhob***

| 1 | 11 | 21 | 31 | 41 |
|---|---|---|---|---|
| MAAIRKKLVV | VGDGACGKTC | LLIVFSKDEF | PEVYVPTVFE | NYVADIEVDS |
| 51 | 61 | 71 | 81 | 91 |
| KQVELALWDT | AGQEDYDRLR | PLSYPDTDVI | LMCFSVDSPD | SLENIPEKWV |
| 101 | 111 | 121 | 131 | 141 |
| PEVKHFCPNV | PIILVANKKD | LRNDENVRNE | LSRMKQEPVK | TEDGRAMAVR |
| 151 | 161 | 171 | 181 | 191 |
| IGAYDYLECS | AKTKEGVREV | FETATRAALQ | KRSRPSGGCL | NCCKLL |

**Legend:**

**The selection scale:**

| 1 | 2 | 3 | 4 | 5 | 6 | 7 |
|---|---|---|---|---|---|---|

Positive selection — Purifying selection

Likelihood ratio test between the null model (no positive selection) and the alternative model (enabling positive selection) shows a significance level of: 0.05

**Figure S2**

**Gene: *rnf4***

1 XQAQIKRRSG 11 GSSRRPSKRR 21 RVTNAPPAME 31 PETIEVLENN 41 LHSSEDVVDL
51 TCEGSEPAVV 61 DLTSNDSVVI 71 VDEGLQGHRE 81 ASGESYVLSS 91 DEEEVEDSGM
101 MPTPAFLSSL 111 ESSNKARATP 121 GTISCPICLD 131 TYGEIVGSGR 141 LVVSTKCGHL
151 FCSQCIRDCL 161 RKASKCPTCR 171 KKLNYKQYHP 181 IYI

**Legend:**

**The selection scale:**

| 1 | 2 | 3 | 4 | 5 | 6 | 7 |
|---|---|---|---|---|---|---|

Positive selection Purifying selection

**Figure S2**

**Gene: *rnf14***

1 MSSEDLEAQE 11 DELLALASIY 21 NEEEFHRAES 31 AQGGEIRINL 41 ELPQDFRIFA
51 KGEEHIKYPV 61 SFLPTLILNF 71 ELPSDYPSNS 81 PPVFTLSSRW 91 LSRVQLGTLC
101 KRLDELWQEN 111 VGSVVLFSWI 121 QFLKEDLFSF 131 LSIESPLEVT 141 NSNKGKQERK
151 KEGAGKDNAE 161 DTEKDSKEQP 171 DPRAIQEVDP 181 RSNLLLQLLD 191 FDEMQQQKAF
201 DSKMFCCNIC 211 FSEKMGSECT 221 CFKDCKHVYC 231 KECMRDYFQI 241 QIRDGNVQCL
251 NCPEPECSSV 261 ATPAQVKHLV 271 GEELFARYDR 281 LLLQSSLDLM 291 ADVMYCPRKM
301 CETAVLVEPD 311 TTMGICPSCQ 321 YAFCTLCKMG 331 YHGLSPCRAT 341 AAELRGLRDE
351 YLKASEEEKQ 361 FLEQRFGKRV 371 IQKAVEESFS 381 SEWLEKNSKA 391 CPRCSSNIQK
401 VDGCNKMTCT 411 SCKQYFCWLC 421 LGPLSRVNPY 431 GHFNDTSSPC 441 FNQLFQGVDI
451 DDDEEFWSDE 461 ED

**Legend:**

**The selection scale:**

| 1 | 2 | 3 | 4 | 5 | 6 | 7 |
|---|---|---|---|---|---|---|

Positive selection    Purifying selection



T. Lorin, W. Salzburger & A. Böhne

**Figure S2**

**Gene: *rock1***

| | | | | |
|---|---|---|---|---|
| 1 | 11 | 21 | 31 | 41 |
| MSAGESLEAR | FEKIDAMLKD | PKSEVNTDCL | LDGLDALVYD | LDFPALRKNK |
| 51 | 61 | 71 | 81 | 91 |
| SIDNFLNRYK | DTISKIRDLR | MKAEDYEVVK | VIGRGAFGEV | QLVRHKATRK |
| 101 | 111 | 121 | 131 | 141 |
| VYAMKLLSKF | EMIKRSDSAF | FWEERDIMAF | ANSPWVVQLF | YAFQDDRYLY |
| 151 | 161 | 171 | 181 | 191 |
| MVMEYMPGGD | LVNLMSNYDV | PEKWARFYTA | EVVLALDGIH | SMGFIHRDVK |
| 201 | 211 | 221 | 231 | 241 |
| PDNMLLDKAG | HLKLADFGTC | MKMNQDGMVR | CDTAVGTPDY | ISPEVLKSQG |
| 251 | 261 | 271 | 281 | 291 |
| GDGYYGRECD | WWSVGVFLYE | MLVGDTPFYA | DSLVGTYSKI | MNHKNALTFP |
| 301 | 311 | 321 | 331 | 341 |
| DDSEISKDAK | NLICAFLTDR | EVRLGRNGVD | EIKRHPFFKN | DQWTWENIRE |
| 351 | 361 | 371 | 381 | 391 |
| TAAPVVPELS | SDIDTSNFDD | IEEDKGDEET | FPIPKAFVGN | QLPFVGFTYY |
| 401 | 411 | 421 | 431 | 441 |
| SSNQLARGLS | TKTSEKRSSS | VKEDKSQLEN | LQKRIYLLEE | QLHSEMQLKD |
| 451 | 461 | 471 | 481 | 491 |
| EMEQKCRASN | IKLDKIMKEL | DEETNLRKNM | DSTVSHLEKE | KMMAQHRATE |
| 501 | 511 | 521 | 531 | 541 |
| YQRKAEQEAE | KRRNVENEVS | TLKEQLEDMR | KISQNSQISN | DKITQLQKQL |
| 551 | 561 | 571 | 581 | 591 |
| EEANDLLRVE | SDTAARLRKS | HTELTKSMSH | LETVNRELQE | KSRAADGARH |
| 601 | 611 | 621 | 631 | 641 |
| QLEKEVLQLQ | AALESERRNW | SQGSEEIQEL | QGRITGLQED | LKNLKLTLSK |
| 651 | 661 | 671 | 681 | 691 |
| VETERKQAQE | RSNNLEKEKN | NLEIDLNYKL | KTLQQRLEHE | LAEHAATKAR |
| 701 | 711 | 721 | 731 | 741 |
| LTDKYESIEE | AKSATMQAVE | QKVSEESMAR | LKAENRVVEV | EKQCSMLEFD |
| 751 | 761 | 771 | 781 | 791 |
| LKQSVQKIEQ | LMKQKERLEE | EVRSVRVQLE | QESGRRVQAQ | AELKTRAVEA |
| 801 | 811 | 821 | 831 | 841 |
| DRMKGSEKQL | KQEMNTALEA | KRSLEFQLAQ | LTNYSPDMSG | IFFPKTTHSS |
| 851 | 861 | 871 | 881 | 891 |
| LVTAVSAQYF | EKACLDDIKL | GFNGFDSRAC | FSVAICFCTD | EGGSCAGNIF |
| 901 | 911 | 921 | 931 | 941 |
| SSCGSRRILC | EWSRRMFRCR | QLEETNKTLT | KDVENLSKEK | TELSEKMRVQ |
| 951 | 961 | 971 | 981 | 991 |
| EEVSATEKEE | LTNSVKAYYE | KILNTERTLK | TQAVNKLAEI | MNRKDMKLDQ |
| 1001 | 1011 | 1021 | 1031 | 1041 |

## Figure S2

KKKGSTADLR KKEKENRKLQ LELNQEKEKF NHMAIKYQKE LSEMQAQLAE
1051 1061 1071 1081 1091
ESTYRNELQM QLDSKESDIE QLREKLNDLQ LRMDNSSVAS LQTDEADGNI
1101 1111 1121 1131 1141
AESRLEGWLS IPNRANIKRY GWKKQYVVVS SKKILFYNDE QDKEQSNPSM
1151 1161 1171 1181 1191
VLDIDKLFHV RPVTQGDVYR AETDEIPRIF QILYANEGEC RKDADTESVH
1201 1211 1221 1231 1241
QGEKTNCLPH KGHEFIPTLY HFPTNCEACA KPLWHVFKPP PALECRRCHV
1251 1261 1271 1281 1291
KCHKDHLDRK EDVIAPCKVN YDVTTARDML LLALSQEEQK KWIGHLGKKI
1301 1311 1321 1331
PKTPPSSFTR ASPRTMSTRS VANQSFRRNP KSVTGKPR

**Legend:**

**The selection scale:**

| 1 | 2 | 3 | 4 | 5 | 6 | 7 |
|---|---|---|---|---|---|---|

Positive selection Purifying selection

**Figure S2**

**Gene: *rock2***

| 1 | 11 | 21 | 31 | 41 |
|---|---|---|---|---|
| MSAGAERRME | NRLKKLEAMI | KDPRSLINLE | SLLDSMNALV | LDLDFPALRK |
| 51 | 61 | 71 | 81 | 91 |
| NKNIETFLNR | YEKVTGKIQD | LQMKSEDYEV | VKVIGRGAFG | EVQLVRHKAS |
| 101 | 111 | 121 | 131 | 141 |
| QKVYAMKLLS | KFEMIKRSDS | AFFWEERDIM | AFANSPWVVQ | LCCAFQDDRY |
| 151 | 161 | 171 | 181 | 191 |
| LYMVMEYMPG | GDLVNLTSTY | DVPEKWAKFY | TAEVVLALDA | IHSMGFIHRD |
| 201 | 211 | 221 | 231 | 241 |
| VKPDNMLLDR | HGHLKLADFG | TCMKMDKTGM | VRCDTAVGTP | DYISPEVLKS |
| 251 | 261 | 271 | 281 | 291 |
| QGGDGYYGRE | CDWWSVGVFI | FEMLVGDTPF | YADSLVGTYS | KIMDHKNSLN |
| 301 | 311 | 321 | 331 | 341 |
| FPDDVEISED | AKNLICAFLT | DREVRLGRSG | VEEIKRHPFF | KNDQWTFDTI |
| 351 | 361 | 371 | 381 | 391 |
| RETVAPVVPE | LSSDIDTSNF | DEIEDDKGDV | ETFPVPKAFV | GNQLPFIGFT |
| 401 | 411 | 421 | 431 | 441 |
| YFREDQLLSD | SNPSAVENEH | RTSIKGEDSV | ASVQLQKKLH | QLEEQLNNEM |
| 451 | 461 | 471 | 481 | 491 |
| QSKDELDHKC | RAACSRLEKV | SKELDEEITS | RKHLESSLRQ | LEREKALLQH |
| 501 | 511 | 521 | 531 | 541 |
| KNVEYQRKAE | NEADKKRCLE | NDVNSLRDQL | EDLKKRNQNS | QISNEKNIQL |
| 551 | 561 | 571 | 581 | 591 |
| QRQLDEVNSL | LRTESDTAAR | LRKTQTEMTK | QTQQLESNNR | ELQDKCCMLE |
| 601 | 611 | 621 | 631 | 641 |
| NAKLKLEKDF | ISLQSALESE | KRDRNHGTEI | ISDLQGRISG | LEDELKHVKC |
| 651 | 661 | 671 | 681 | 691 |
| SLSKAEMEKR | QLHERLTDFE | KEKSNIEIDM | TYKLKVLQQS | LEQEEAEHKA |
| 701 | 711 | 721 | 731 | 741 |
| TKARLADKNK | IYESIEEAKS | EAMKEMEKKL | QEERSSKMKV | ENILLEVEKQ |
| 751 | 761 | 771 | 781 | 791 |
| CSMLDCDLKQ | SHQKLEELRR | QKEKLTEEVK | NLTLKIEQET | QKRSLTQNDL |
| 801 | 811 | 821 | 831 | 841 |
| KMQSQQVNAL | KMSEKQLKQE | INHLLEIKLS | LEKQNSELRK | ERQDADGQMK |
| 851 | 861 | 871 | 881 | 891 |
| ELQDQLEAEQ | YFSTLYKTQV | RELKEECEEK | NKLCKEMQQK | LQELQDERDS |
| 901 | 911 | 921 | 931 | 941 |
| LAAQLEITLT | KADSEQLARS | IAEEQYSDLE | KEKIMKELEI | KEMMARHKQE |
| 951 | 961 | 971 | 981 | 991 |
| LAEKDATIGS | LEEANRTLTS | DVANLANEKE | ELNNRLKETQ | EQLQKAKEDE |
| 1001 | 1011 | 1021 | 1031 | 1041 |

**Figure S2**

```
HSMALTKVQF EKQLQSERTL KTQAVNKLAE IMNRKEVRGG ASRRGNDTDV
1051       1061       1071       1081       1091
RRKEKENRKL QLELRSEREK LNGTIIKYQK EINEMQAQIA DESQVRIELQ
1101       1111       1121       1131       1141
MALDSKDSDI EQLRSLLNSM NVHGLDSASI GSGPDFDSDE SYPDTTRLEG
1151       1161       1171       1181       1191
WLSLPVRNNT KRFGWEKKYV VVSSKKILFY NNEQDKEQSN PYMVLDIDKL
1201       1211       1221       1231       1241
FHVRPVTQTD VYRADAKEIP RIFQILYANE GESKKDQEFP VEPLPIGEKS
1251       1261       1271       1281       1291
SYICHKGHEF IPTLYHFPTN CEACTKPLWN MFKPPPALEC RRCHIKCHKD
1301       1311       1321       1331       1341
HMDKKEEVIA PCRVNYDVST AKNLLLLANS QEEQQKWVSR LVKKIPKKPP
1351       1361       1371       1381       1391
APDHFSRSSP RASMKVQPNQ SIRRPSRQLP PNKPSTGPML RIILH
```

**Legend:**

**The selection scale:**

| 1 | 2 | 3 | 4 | 5 | 6 | 7 |
|---|---|---|---|---|---|---|

Positive selection — Purifying selection

**Figure S2**

## Gene: *src*

| | | | | |
|---|---|---|---|---|
| 1 | 11 | 21 | 31 | 41 |
| MGATKSKPKD | PGQRSRSLDG | TIGIVGSYHP | SHSPVQQTLT | PNRSPAAEGS |
| 51 | 61 | 71 | 81 | 91 |
| RRGNQSLTSN | AELVLFGGVD | SSIGITSPQR | VLIFLFSVTG | GVTTFVALYD |
| 101 | 111 | 121 | 131 | 141 |
| YESRTASDLS | FKKGERLQIV | NNTEGDWWLA | RSLTTGESGY | IPSNYVAPSD |
| 151 | 161 | 171 | 181 | 191 |
| SIQAEEWYFG | KITRRDSERL | LLNLENRRGT | FLVRESETTK | GAYCLSVLDY |
| 201 | 211 | 221 | 231 | 241 |
| DNTKGLNVKH | YKIRKLDSGG | FYITSRTQFS | NLQQLVSHYR | KHADGLCHPL |
| 251 | 261 | 271 | 281 | 291 |
| TEVCPVIKPQ | TQGLARDAWE | IPRESLRLDV | KLGQGCFGEV | WMGTWNGTTR |
| 301 | 311 | 321 | 331 | 341 |
| VAIKTLKPGT | MSTEAFLQEA | QVMKKLRHEK | LVQLYAVVSE | EPIYIVTEFM |
| 351 | 361 | 371 | 381 | 391 |
| GQGSLLDFLK | GDMGKILRLP | QLVDMASQIA | AGMAYVERMN | YVHRDLRAAN |
| 401 | 411 | 421 | 431 | 441 |
| ILVGDNLVCK | VADFGLARLI | EDNEYTARQG | AKFPIKWTAP | EAALYGRFTI |
| 451 | 461 | 471 | 481 | 491 |
| KSDVWSFGIL | LTELATKGRV | PYPGMVNREV | LDQVERGYRM | PCPAECPESL |
| 501 | 511 | 521 | 531 | 541 |
| HELMLTCWRK | EPEERPTFEY | LQSFLEDYFT | STEPQYQPGE | NL |

**Legend:**

**The selection scale:**

| | | | | | | |
|---|---|---|---|---|---|---|
| 1 | 2 | 3 | 4 | 5 | 6 | 7 |

Positive selection — Purifying selection

**Figure S2**

**Gene: *tgb1i1***

```
1          11         21         31         41
XXXXXXXXXX XXXXXXXXXX XXXXXXXXXX XXXXXXXXXX XXXXXXXXXX
51         61         71         81         91
XXXXXXXXXX XXXXXXXXXX XXXXXXXXXX XXXXXXXXXX XXXXXXXXXX
101        111        121        131        141
XXXXXXXXXX XXXXXXXXXX XXXXXXXXXX XXXXXXXXXX XXXXXXXXXX
151        161        171        181        191
XXXXXXXXXX XXXXXXXXXX XXXXXXXXXX PVLTSSRQAA PPPQNPSGSL
201        211        221        231        241
DSMLGLLQSD LSRQGITTTA KGTCSACQKP IVGQVXXXXX XXXXXXXXXX
251        261        271        281        291
XXXXXXXXXX XXXXXXXXXX XXXXXXXXXX XXXXXXXXXX XXXXXXXXXX
301        311        321        331        341
XXXXXXXXXX XXXXXXXXXX XXXXXXXXXX XXXXXXXXXX XXXXXXXXXX
351        361        371        381        391
XXXXXXXXXX XXXXXXXXSE CYTPFVNGSF FEHEGQPLCE AHYHRSRGSV
401        411        421        431        441
CQACQEPIRG RCVTAMGAKF HPQHLVCAFC LKALSKGSFK EQDNKPYCQG
451
CFIKLFG
```

**Legend:**

**The selection scale:**

| 1 | 2 | 3 | 4 | 5 | 6 | 7 |
|---|---|---|---|---|---|---|

Positive selection Purifying selection

Figure S2

**Gene: *thrap3***

| | | | | |
|---|---|---|---|---|
| 1 | 11 | 21 | 31 | 41 |
| MSKVPVSKSP | SRSRSGSGSR | SPSRSFSRSR | TRTRSRSRSR | KHRYSSRSRS |
| 51 | 61 | 71 | 81 | 91 |
| RSRSHSPPHN | RERNYPREYQ | NNREFRGYNR | GFRRPYYYRG | RGRGYYPRGR |
| 101 | 111 | 121 | 131 | 141 |
| YQRGGGYGNY | RPNWQNYRQQ | PQQHHQHSPR | RGRSRSRSPK | RRSGTPRSRS |
| 151 | 161 | 171 | 181 | 191 |
| RSRRSGSSKR | NAAKELKESG | RPDSAAKEAQ | RAGSRDEEAA | GAAGGEGAPD |
| 201 | 211 | 221 | 231 | 241 |
| RASGSWQGLI | DYDTSPKRTS | PAVRSAIIVS | QGTAHPSPSL | QSVAVKRPSP |
| 251 | 261 | 271 | 281 | 291 |
| AVKGGSPSPS | RSSAAAQSKS | LGNAPWQSSG | PAPTSKSPPQ | QSPTAVFSGF |
| 301 | 311 | 321 | 331 | 341 |
| GFFSKDDVRA | GEKPSSVSTA | FKKFLEEHKN | KIQVAEWENG | REKEQKMAEL |
| 351 | 361 | 371 | 381 | 391 |
| ERDKGNGKAG | SFDKGAAYSG | LKPDYYSKNE | EEKYGYDDEF | ELGSAAEFLK |
| 401 | 411 | 421 | 431 | 441 |
| GPQFGSAEAG | EDQEKRHKVR | NQKEREMEDE | PKHKSKITIT | ANRDMFDERF |
| 451 | 461 | 471 | 481 | 491 |
| NKWDELAYFP | SAKEKLRKEE | DAGEDDIDDV | EEELYRSRKQ | ERAAAAAAAA |
| 501 | 511 | 521 | 531 | 541 |
| KKAEASGYRG | FSPDKAPKAS | RKKEKQGQSP | SPPARKSSEN | REREMFNVRR |
| 551 | 561 | 571 | 581 | 591 |
| DDSPPRSTPA | YSGKRSAEVS | VRMDPFHEDY | ASSSGVLANE | RRFSRDLVHP |
| 601 | 611 | 621 | 631 | 641 |
| SKKDQEFRSI | FQHIQSTQLR | RSPSELFAQH | IVTIVHHIKA | QHFESSGMTL |
| 651 | 661 | 671 | 681 | 691 |
| NERFAMYQRR | AAEMMKPRKS | PEIHRRIDVS | PSAFRKHSHL | FEEMKSSRES |
| 701 | 711 | 721 | 731 | 741 |
| SSKDEGKKMK | TDSMDLRLDI | ERRKKYSSKE | REHKRDGMRD | SGESRGSSLS |
| 751 | 761 | 771 | 781 | 791 |
| RERSTEKSSK | HHKKTKKRKK | TRERSRSSSE | SSSSSHSFKG | GDYPEGPEQK |
| 801 | 811 | 821 | 831 | 841 |
| EEGFNKARLG | VRDYGGPMER | GRGRGGFQFR | IRGRGWNRGN | YPGNNSNGNP |
| 851 | 861 | 871 | 881 | 891 |
| SNPGNPVRSK | EEEWDPEYTP | KSKKYYLHDD | REGEGEKKWV | DNRGRGRGTF |
| 901 | 911 | 921 | 931 | 941 |
| QRGRGRFMFR | KTSSSPKWTH | DKFQGSGEEG | ELPDDSDAEN | KEEDKSGGTA |
| 951 | | | | |
| ATEQ | | | | |

**Figure S2**

**Legend:**

**The selection scale:**

| | | | | | | |
|---|---|---|---|---|---|---|
| 1 | 2 | 3 | 4 | 5 | 6 | 7 |

Positive selection Purifying selection

**Figure S2**

## Gene: *ube3a*

| 1 | 11 | 21 | 31 | 41 |
|---|---|---|---|---|
| MKRAAAKHLI | ERYYHQLTEG | CGNEACTNEF | CASCPSFHRM | DNNAAAVKAL |
| 51 | 61 | 71 | 81 | 91 |
| ELYKINAKLC | DPHPSKKGTS | SAYLENSAKG | AHNNSCSDRK | MNRKEAHAAR |
| 101 | 111 | 121 | 131 | 141 |
| DDFKDLNYLT | EEKVYEILEI | CGEKEDYSPL | IRVIGRVFSS | AEGLVQSFRK |
| 151 | 161 | 171 | 181 | 191 |
| AKQHTKEELK | SLQGKDEDKD | EDEKEKAACS | AAAAAMEEDS | GASSSRIGDG |
| 201 | 211 | 221 | 231 | 241 |
| ATQGDNNVHK | LGPDEVSVDV | EAVRRVYNKL | LANEKIETAF | LNALVYLSPN |
| 251 | 261 | 271 | 281 | 291 |
| VECDLTYHNV | YSRDPNYLNL | FIIVMENSNL | HSPEYLEIAL | PLFCKAMSKL |
| 301 | 311 | 321 | 331 | 341 |
| PLPAQAKLVR | LWSQYSAEQI | RRMMETFQQL | ITYKVISNEF | NSRNLVNDDD |
| 351 | 361 | 371 | 381 | 391 |
| SVVAATKCLK | IVYYANVLGG | DVDTDHNEEE | DDEPIPESSE | LTLQELLGEE |
| 401 | 411 | 421 | 431 | 441 |
| RRNKKGPRVD | PLETELGVKT | IDCRKPLIPF | EEFVNEPLND | VLEMDKDYTF |
| 451 | 461 | 471 | 481 | 491 |
| FKVETENKFS | FMTCPFILNA | VTKNLGLYYD | NRIRMYSERR | ITVLYSLVQG |
| 501 | 511 | 521 | 531 | 541 |
| QQLNPYLRLK | VRRDHIIDDA | LVRLEMIAME | NPADLKKQLY | VEFEGEQGVD |
| 551 | 561 | 571 | 581 | 591 |
| EGGVSKEFFQ | LVVEEIFNPD | IGMFTYDETT | KLFWFNPSSF | ETEGQFTLIG |
| 601 | 611 | 621 | 631 | 641 |
| IVLGLAIYNN | CILDVHFPMV | VYRKLMGKKG | TFRDLADSHP | VLFQSLKELL |
| 651 | 661 | 671 | 681 | 691 |
| EYEGSVEEDM | MITFQISQTD | LFGNPLMYDL | RENGDKIPVT | NENRKDFVTQ |
| 701 | 711 | 721 | 731 | 741 |
| YAEYILNKAV | EKQFKAFRRG | FHMVTNESPL | KYLFRPEEIE | LLICGSRNLD |
| 751 | 761 | 771 | 781 | 791 |
| FQALEETTEY | DGGYNRDSRI | IKDFWDIVHA | FGEEQKRLFL | QFTTGTDRAP |
| 801 | 811 | 821 | 831 | 841 |
| VGGLGKLKMI | IAKNGPDTDR | LPTSHTCFNV | LLLPEYTTKE | KLKERLLKAI |
| 851 | | | | |
| TYAKGFGML | | | | |

**Legend:**

**The selection scale:**

1 2 3 4 5 6 7
