# Supplementary material for: Evolutionary Fate of the Androgen Receptor−Signaling Pathway in Ray-Finned Fishes with a Special Focus on Cichlids
Source: G3 (Bethesda). 2015 Sep 1;5(11):2275–83. doi: 10.1534/g3.115.020685 (PMC4632047; doi:10.1534/g3.115.020685)
Supplement: Supporting Information [file supp_g3.115.020685_FigureS3.pdf]

Figure S3

Resulting trees of branch-site model aBS-REL implemented in HyPhy allowing for different Ka/Ks ratios among sites and among branches for all retrieved genes of the AR signaling pathway. When available, the sequence of the spotted gar was used to root the tree, otherwise trees were rooted at the split of Otophysa from the other teleosts.

Figure S3

Resulting trees of branch-site model aBS-REL implemented in HyPhy (Version 2.2.4) allowing for different Ka/Ks ratios among sites and among branches.
Visulaization over http://veg.github.io/hyphy-vision/absrel/ web interface

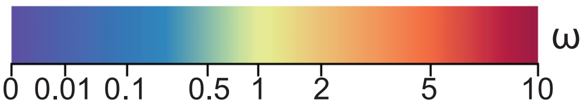

Ka/Ks selection scale for all pages of Figure S1

akt1

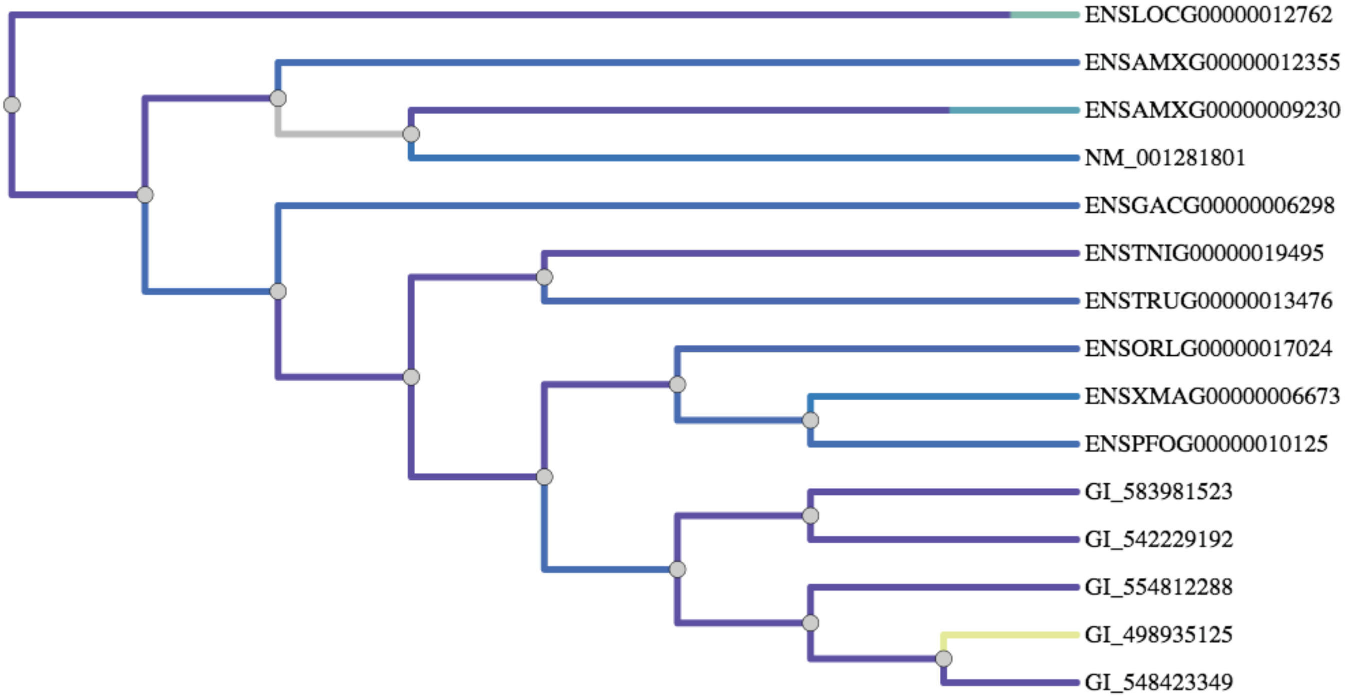

T. Lorin, W. Salzburger & A. Böhne

Figure S3

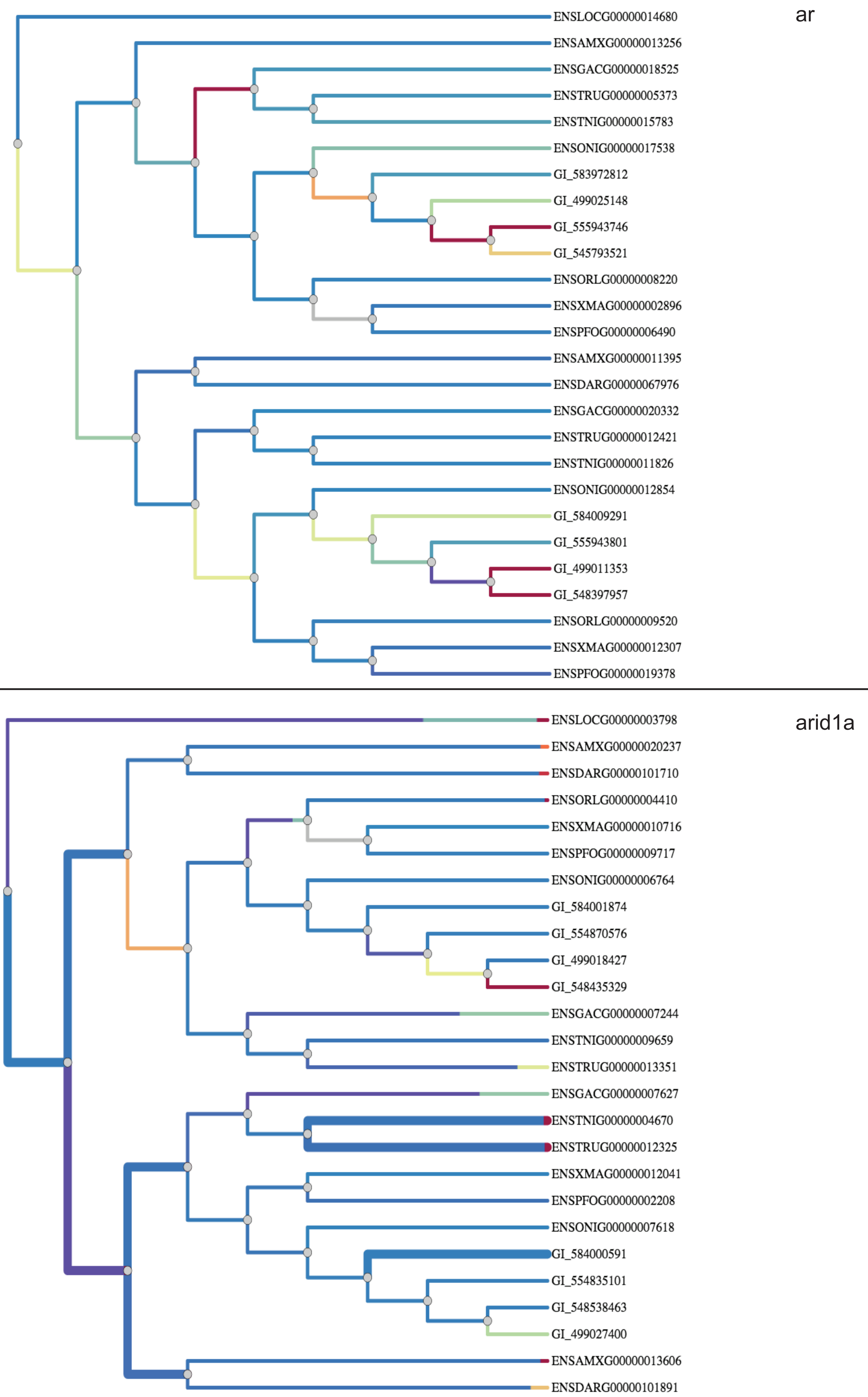
ar
ENSLOCG00000014680
ENSAMXG00000013256
ENSGACG00000018525
ENSTRUG00000005373
ENSTNIG00000015783
ENSONIG00000017538
GI_583972812
GI_499025148
GI_555943746
GI_545793521
ENSORLG00000008220
ENSXMAG00000002896
ENSPFOG00000006490
ENSAMXG00000011395
ENSDARG00000067976
ENSGACG00000020332
ENSTRUG00000012421
ENSTNIG00000011826
ENSONIG00000012854
GI_584009291
GI_555943801
GI_499011353
GI_548397957
ENSORLG00000009520
ENSXMAG00000012307
ENSPFOG00000019378
arid1a
ENSLOCG00000003798
ENSAMXG00000020237
ENSDARG00000101710
ENSORLG00000004410
ENSXMAG00000010716
ENSPFOG00000009717
ENSONIG00000006764
GI_584001874
GI_554870576
GI_499018427
GI_548435329
ENSGACG00000007244
ENSTNIG00000009659
ENSTRUG00000013351
ENSGACG00000007627
ENSTNIG00000004670
ENSTRUG00000012325
ENSXMAG00000012041
ENSPFOG00000002208
ENSONIG00000007618
GI_584000591
GI_554835101
GI_548538463
GI_499027400
ENSAMXG00000013606
ENSDARG00000101891

Figure S3

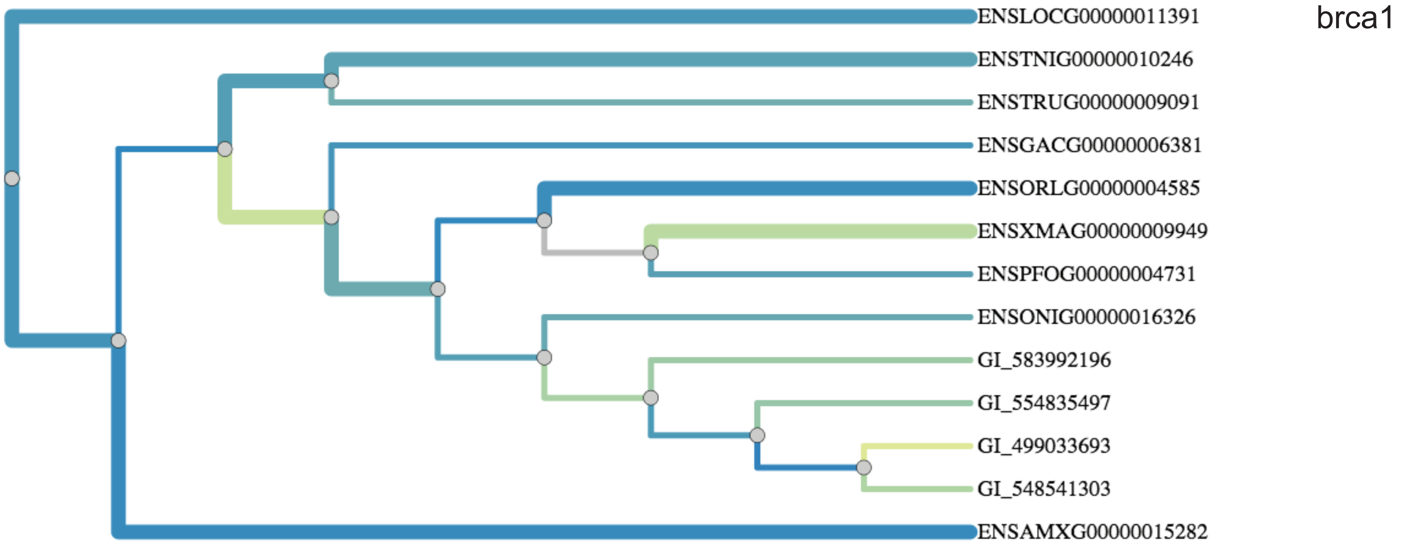
ENSLOCG00000011391
ENSTNIG00000010246
ENSTRUG00000009091
ENSGACG00000006381
ENSORLG00000004585
ENSXMAG00000009949
ENSPFOG00000004731
ENSONIG00000016326
GI_583992196
GI_554835497
GI_499033693
GI_548541303
ENSAMXG00000015282
brca1

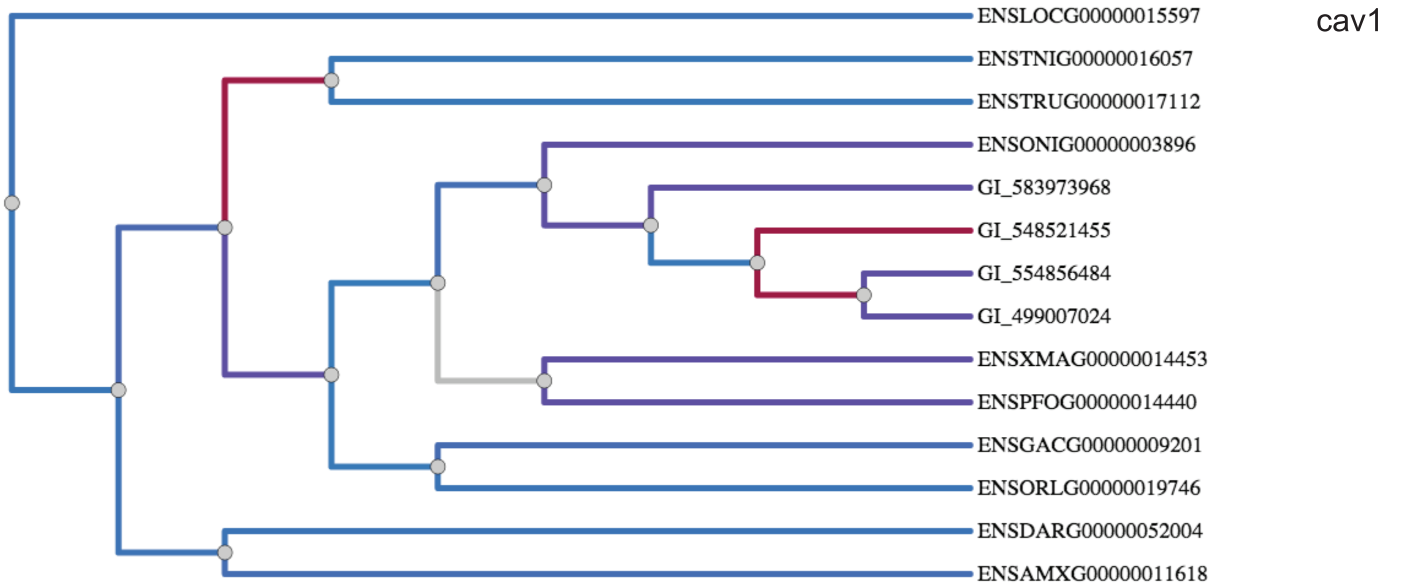
ENSLOCG00000015597
ENSTNIG00000016057
ENSTRUG00000017112
ENSONIG00000003896
GI_583973968
GI_548521455
GI_554856484
GI_499007024
ENSXMAG00000014453
ENSPFOG00000014440
ENSGACG00000009201
ENSORLG00000019746
ENSDARG00000052004
ENSAMXG00000011618
cav1

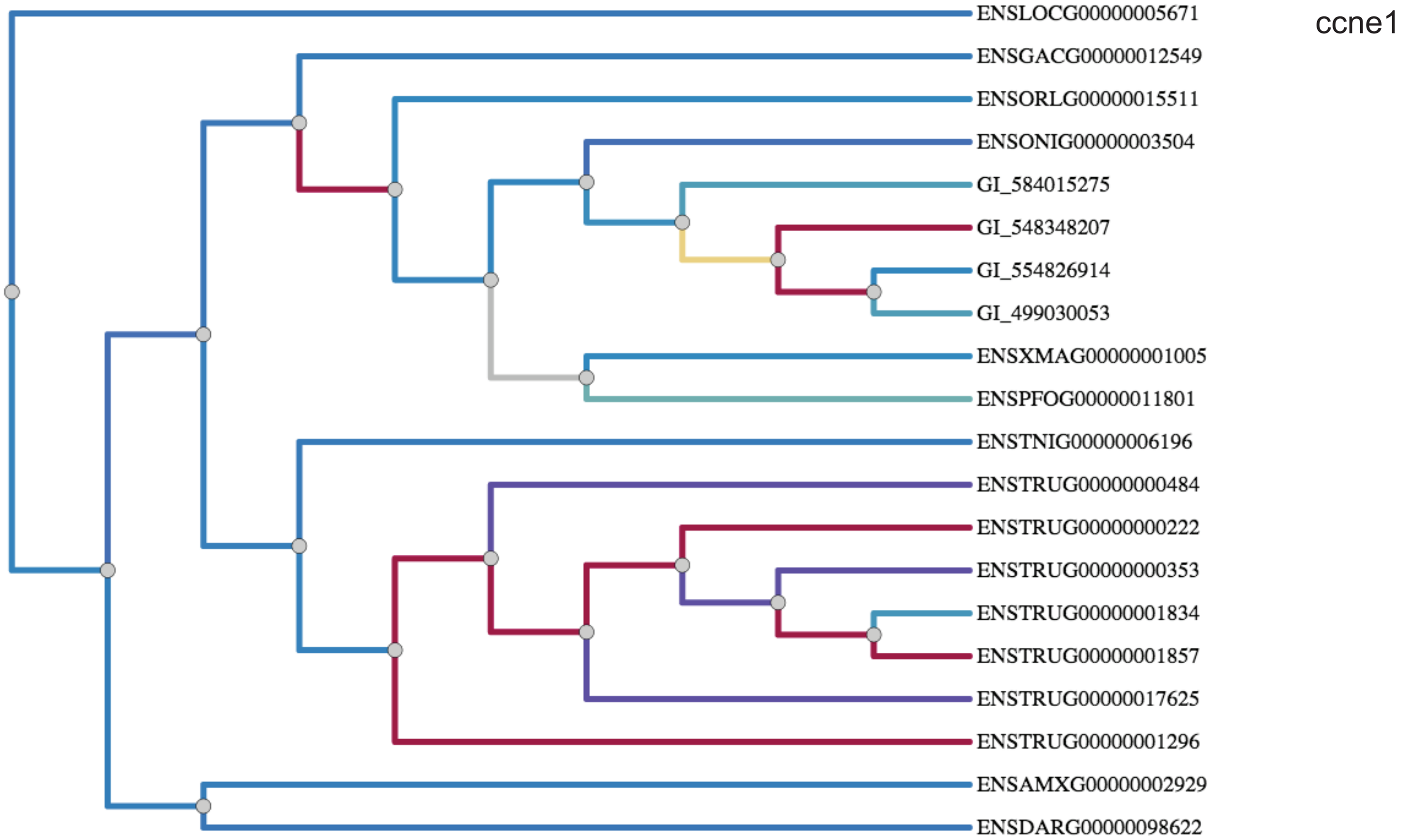
ENSLOCG00000005671
ENSGACG00000012549
ENSORLG00000015511
ENSONIG00000003504
GI_584015275
GI_548348207
GI_554826914
GI_499030053
ENSXMAG00000001005
ENSPFOG00000011801
ENSTNIG00000006196
ENSTRUG00000000484
ENSTRUG00000000222
ENSTRUG00000000353
ENSTRUG00000001834
ENSTRUG00000001857
ENSTRUG00000017625
ENSTRUG00000001296
ENSAMXG00000002929
ENSDARG00000098622
ccne1

Figure S3

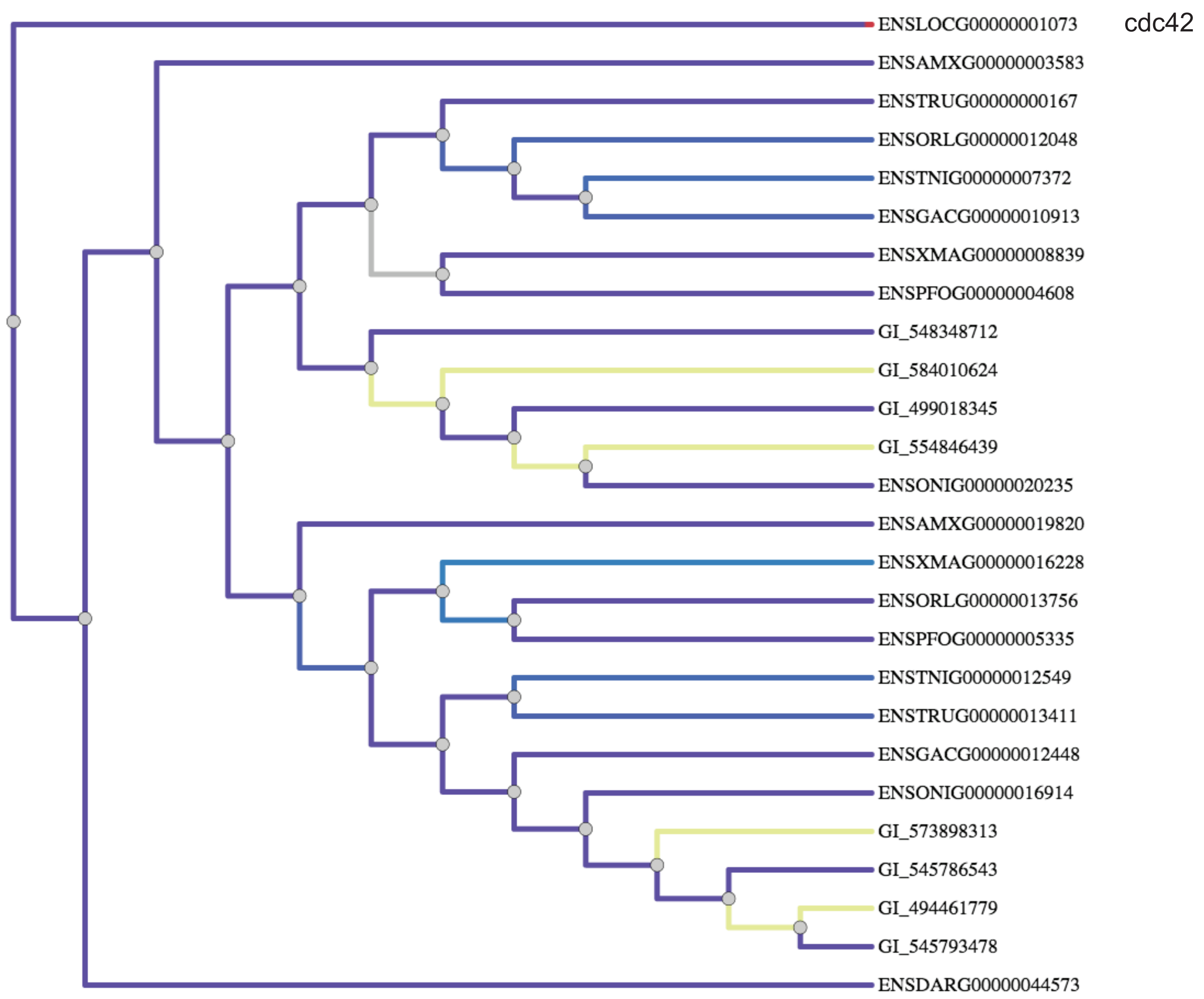
cdc42
ENSLOCG00000001073
ENSAMXG00000003583
ENSTRUG00000000167
ENSORLG00000012048
ENSTNIG00000007372
ENSGACG00000010913
ENSXMAG00000008839
ENSPFOG00000004608
GI_548348712
GI_584010624
GI_499018345
GI_554846439
ENSONIG00000020235
ENSAMXG00000019820
ENSXMAG00000016228
ENSORLG00000013756
ENSPFOG00000005335
ENSTNIG00000012549
ENSTRUG00000013411
ENSGACG00000012448
ENSONIG00000016914
GI_573898313
GI_545786543
GI_494461779
GI_545793478
ENSDARG00000044573

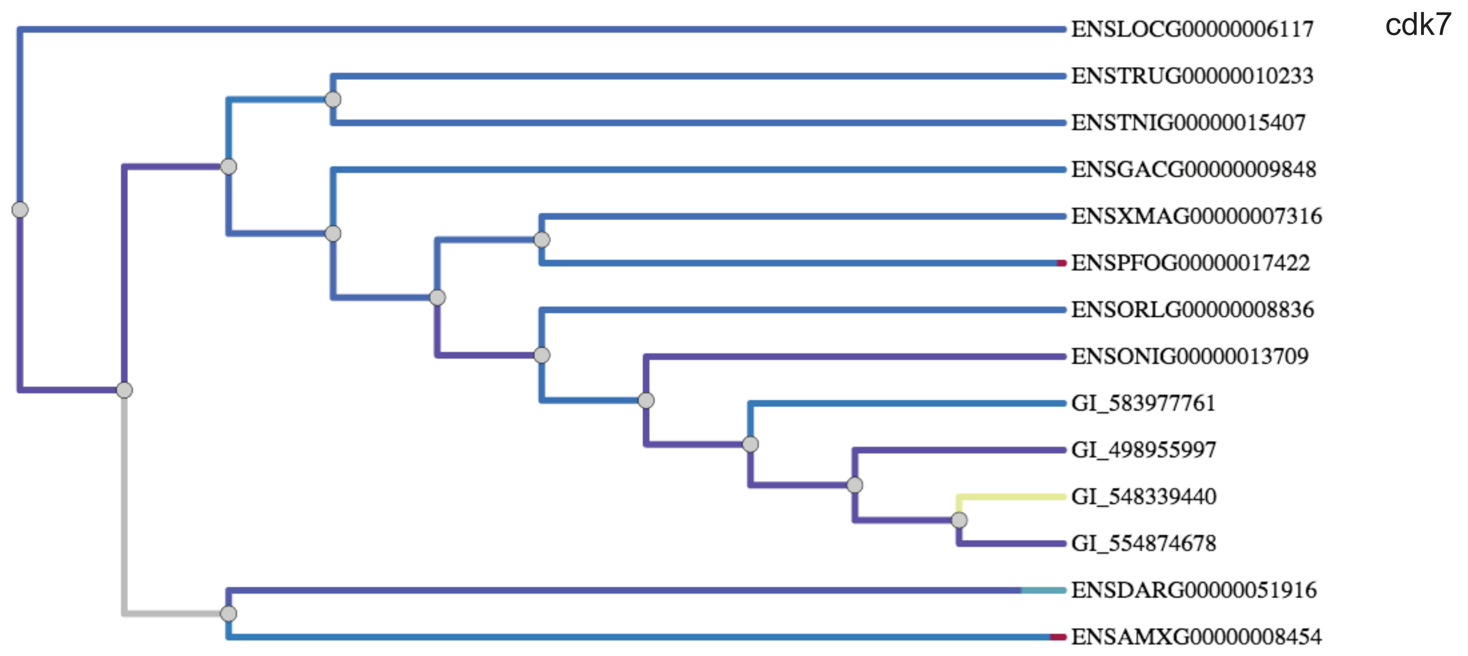
cdk7
ENSLOCG00000006117
ENSTRUG00000010233
ENSTNIG00000015407
ENSGACG00000009848
ENSXMAG00000007316
ENSPFOG00000017422
ENSORLG00000008836
ENSONIG00000013709
GI_583977761
GI_498955997
GI_548339440
GI_554874678
ENSDARG00000051916
ENSAMXG00000008454

Figure S3

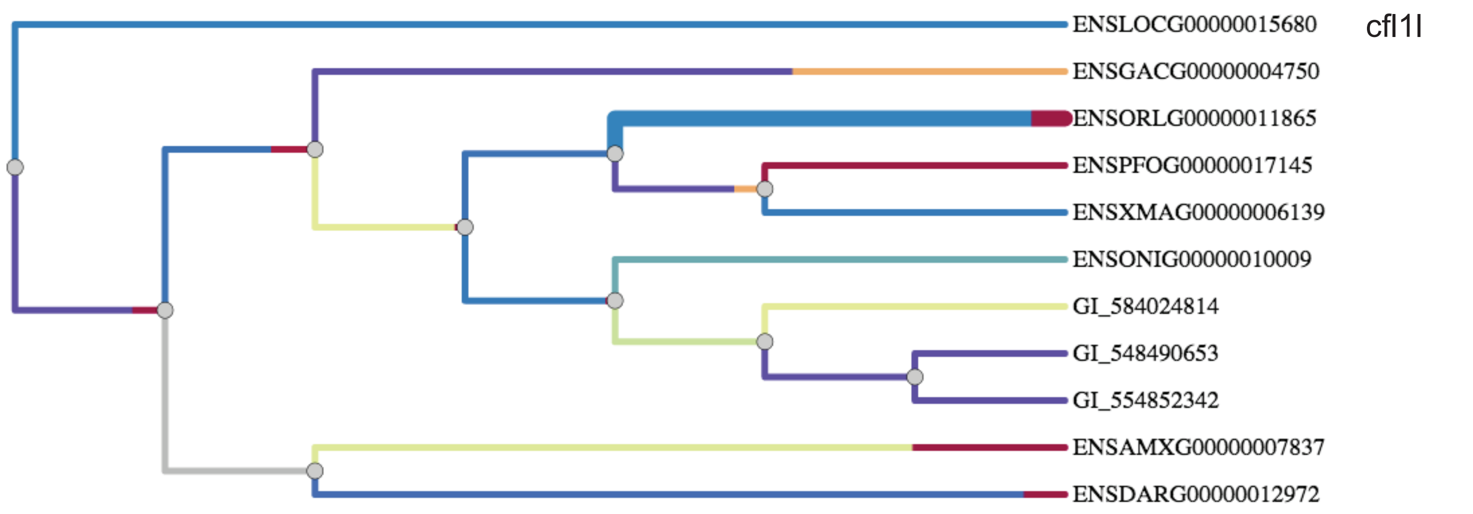
ENSLOCG00000015680
cfl1l
ENSGACG00000004750
ENSORLG00000011865
ENSPFOG00000017145
ENSXMAG00000006139
ENSONIG00000010009
GI_584024814
GI_548490653
GI_554852342
ENSAMXG00000007837
ENSDARG00000012972

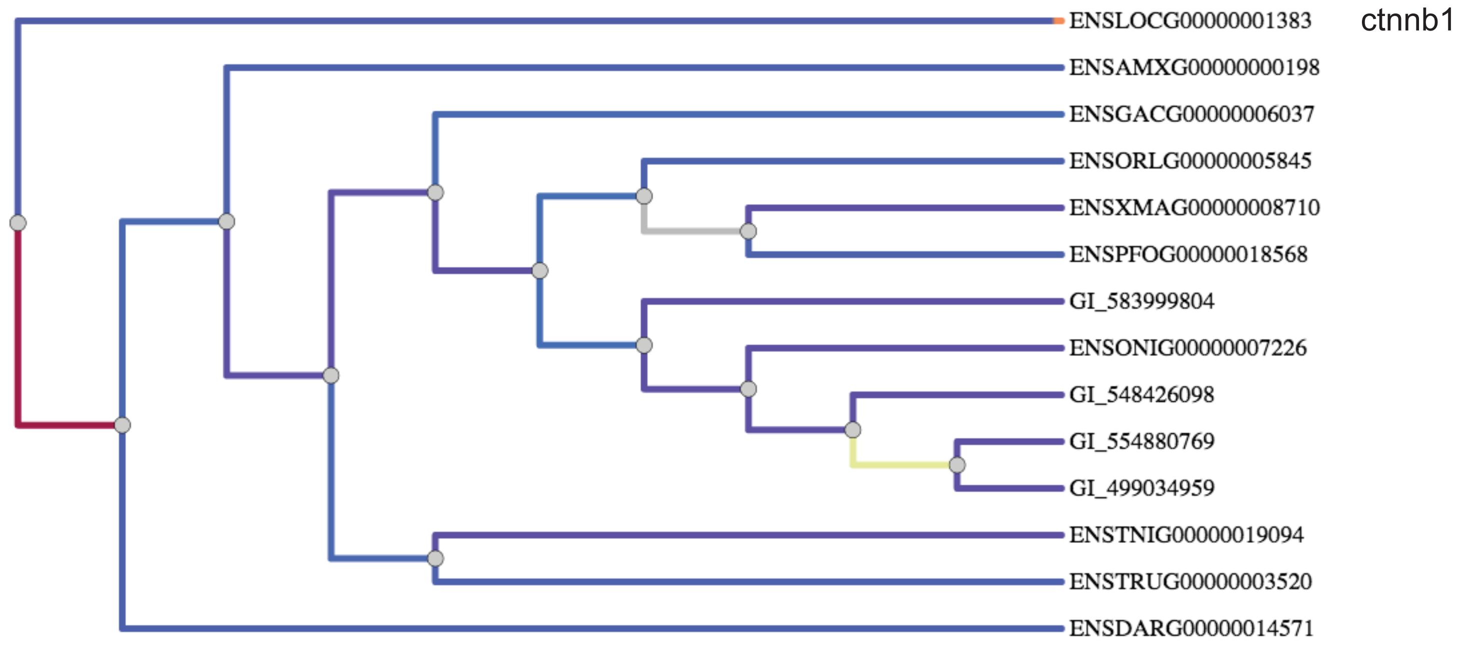
ENSLOCG00000001383
ctnnb1
ENSAMXG00000000198
ENSGACG00000006037
ENSORLG00000005845
ENSXMAG00000008710
ENSPFOG00000018568
GI_583999804
ENSONIG00000007226
GI_548426098
GI_554880769
GI_499034959
ENSTNIG00000019094
ENSTRUG00000003520
ENSDARG00000014571

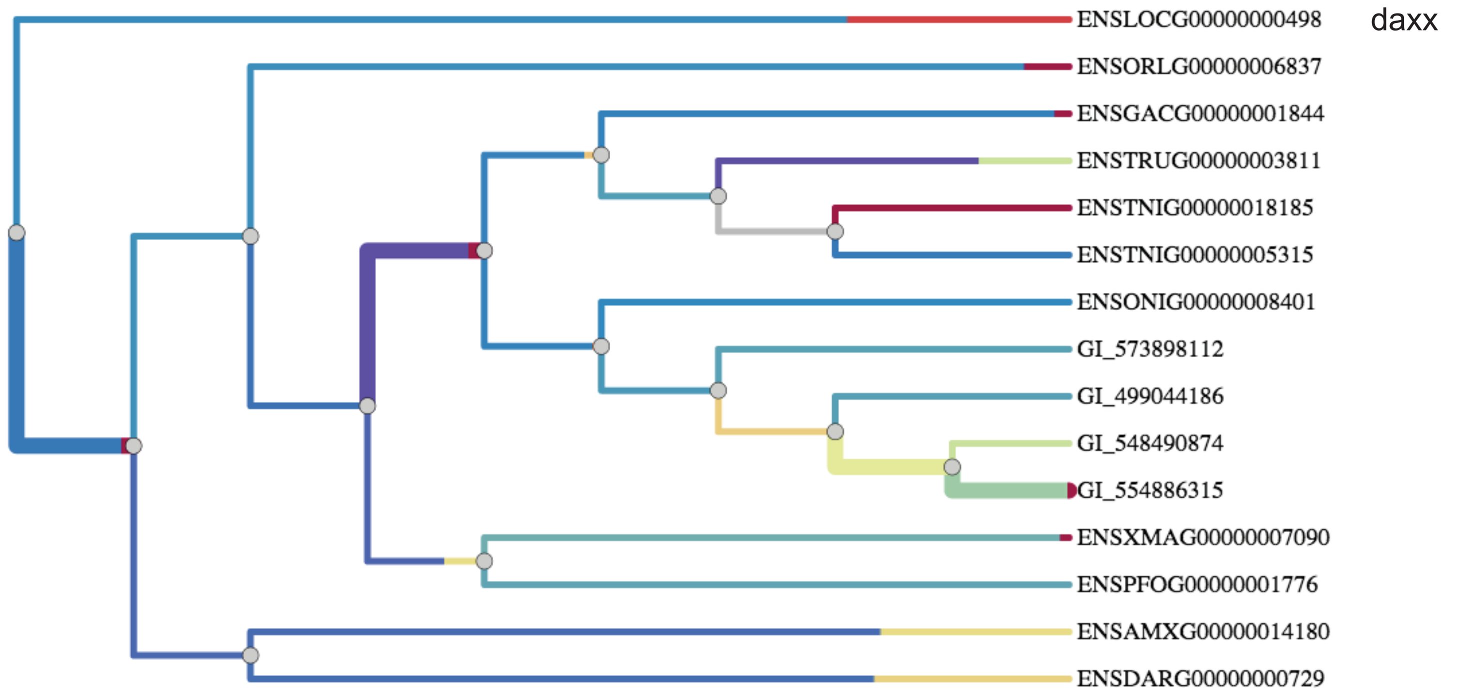
ENSLOCG00000000498
daxx
ENSORLG00000006837
ENSGACG00000001844
ENSTRUG00000003811
ENSTNIG00000018185
ENSTNIG00000005315
ENSONIG00000008401
GI_573898112
GI_499044186
GI_548490874
GI_554886315
ENSXMAG00000007090
ENSPFOG00000001776
ENSAMXG00000014180
ENSDARG00000000729

Figure S3

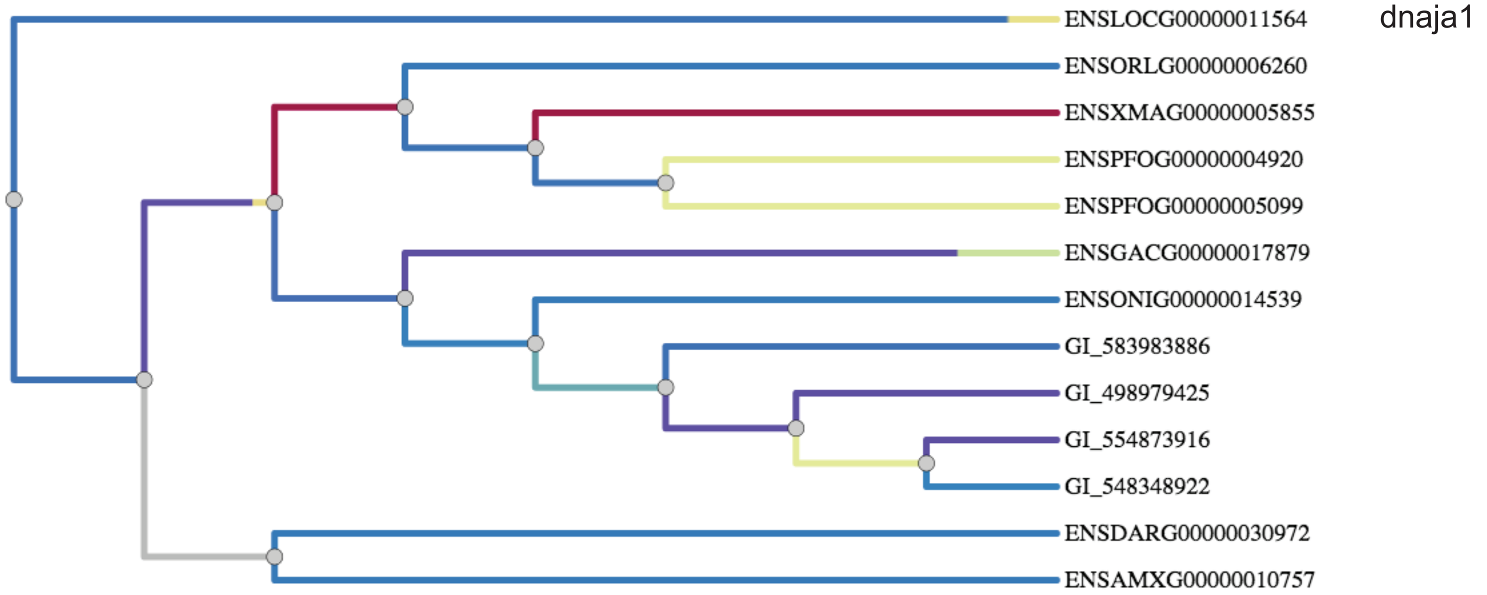
ENSLOCG00000011564
dnaja1
ENSORLG00000006260
ENSXMAG00000005855
ENSPFOG00000004920
ENSPFOG00000005099
ENSGACG00000017879
ENSONIG00000014539
GI_583983886
GI_498979425
GI_554873916
GI_548348922
ENSDARG00000030972
ENSAMXG00000010757

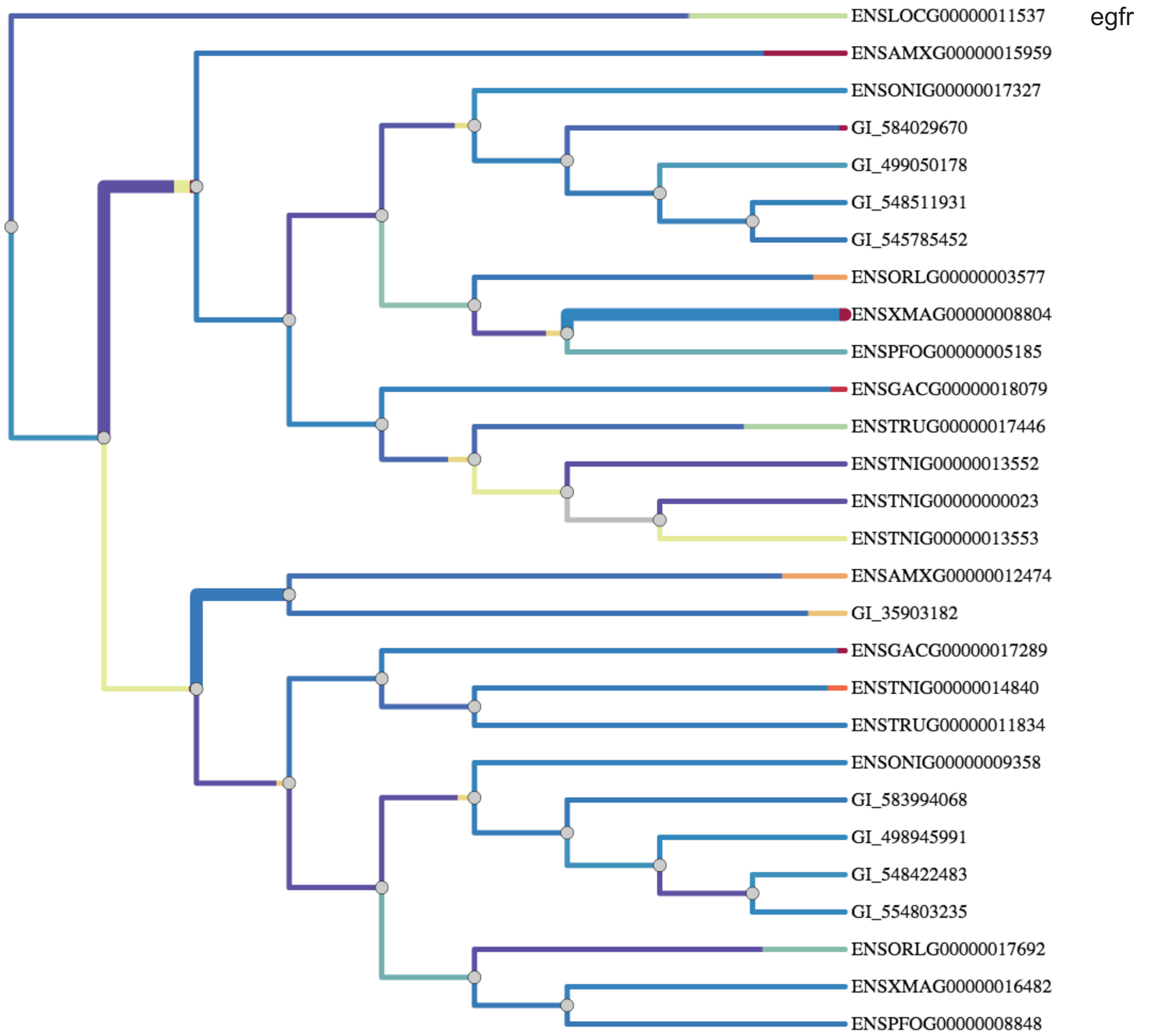
ENSLOCG00000011537
egfr
ENSAMXG00000015959
ENSONIG00000017327
GI_584029670
GI_499050178
GI_548511931
GI_545785452
ENSORLG00000003577
ENSXMAG00000008804
ENSPFOG00000005185
ENSGACG00000018079
ENSTRUG00000017446
ENSTNIG00000013552
ENSTNIG00000000023
ENSTNIG00000013553
ENSAMXG00000012474
GI_35903182
ENSGACG00000017289
ENSTNIG00000014840
ENSTRUG00000011834
ENSONIG00000009358
GI_583994068
GI_498945991
GI_548422483
GI_554803235
ENSORLG00000017692
ENSXMAG00000016482
ENSPFOG00000008848

Figure S3

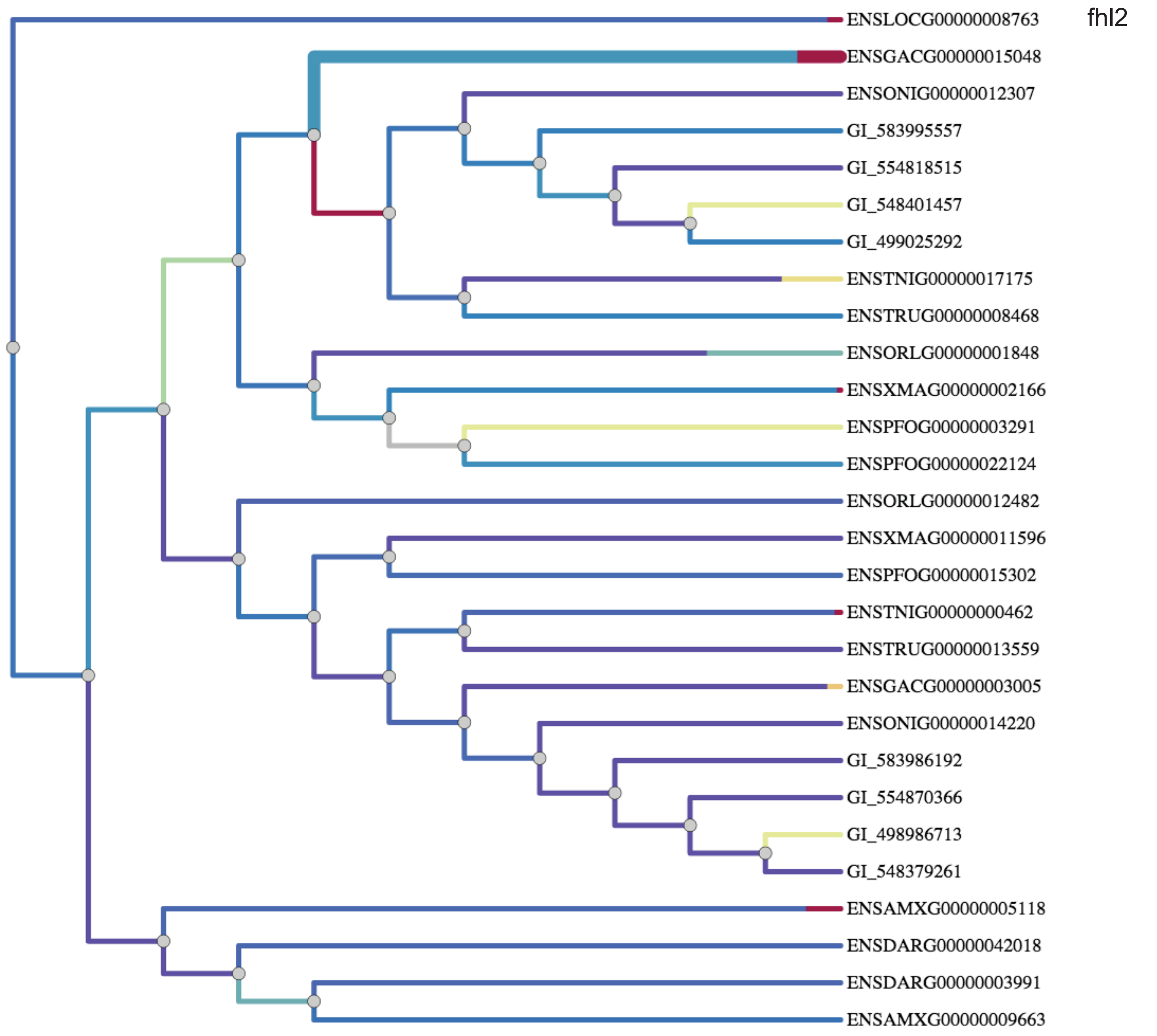
ENSLOCG00000008763
ENSGACG00000015048
ENSONIG00000012307
GI_583995557
GI_554818515
GI_548401457
GI_499025292
ENSTNIG00000017175
ENSTRUG00000008468
ENSORLG00000001848
ENSXMAG00000002166
ENSPFOG00000003291
ENSPFOG00000022124
ENSORLG00000012482
ENSXMAG00000011596
ENSPFOG00000015302
ENSTNIG00000000462
ENSTRUG00000013559
ENSGACG00000003005
ENSONIG00000014220
GI_583986192
GI_554870366
GI_498986713
GI_548379261
ENSAMXG00000005118
ENSDARG00000042018
ENSDARG00000003991
ENSAMXG00000009663
fhl2

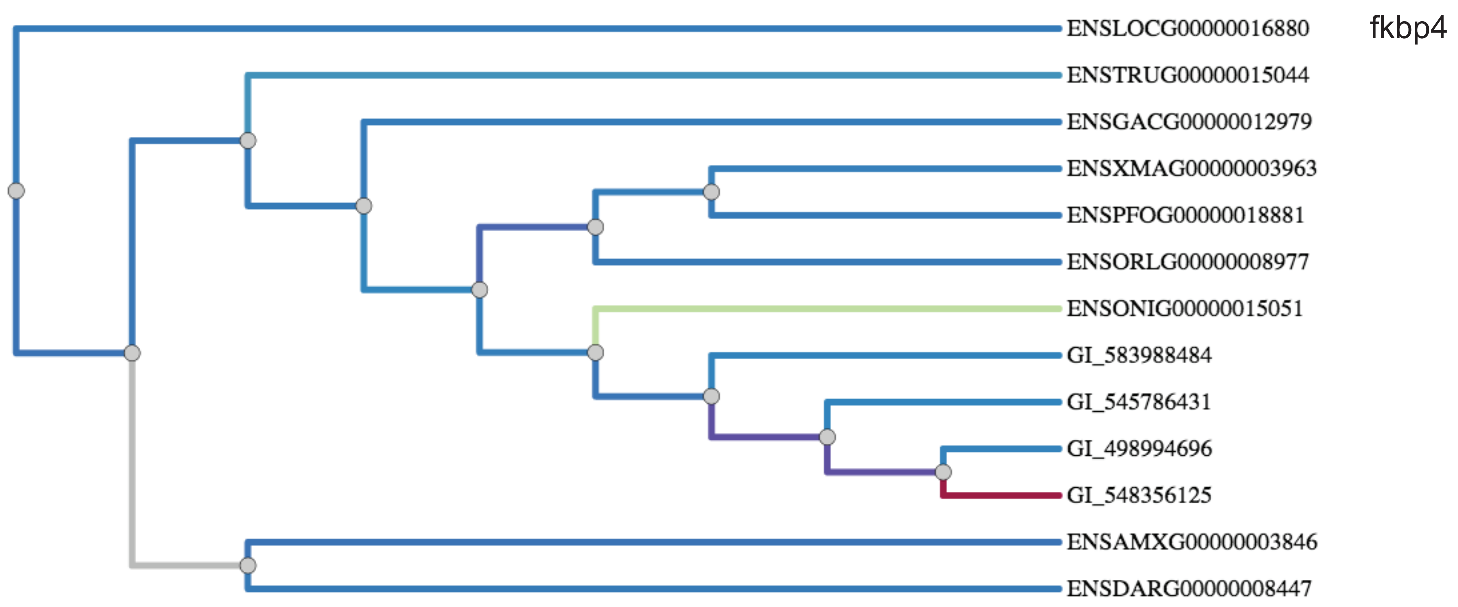
ENSLOCG00000016880
ENSTRUG00000015044
ENSGACG00000012979
ENSXMAG00000003963
ENSPFOG00000018881
ENSORLG00000008977
ENSONIG00000015051
GI_583988484
GI_545786431
GI_498994696
GI_548356125
ENSAMXG00000003846
ENSDARG00000008447
fkbp4

Figure S3

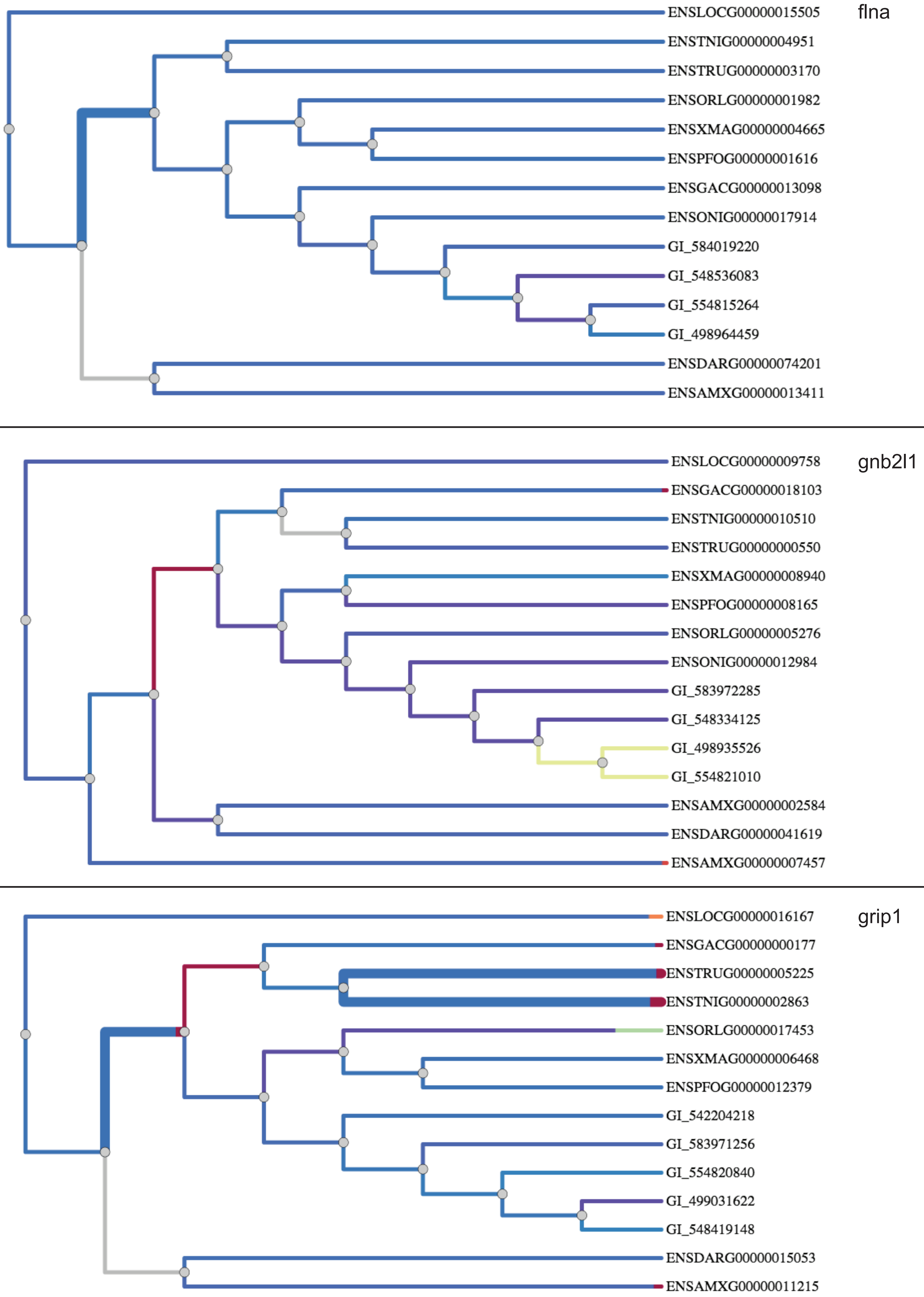
flna
ENSLOCG00000015505
ENSTNIG00000004951
ENSTRUG00000003170
ENSORLG00000001982
ENSXMAG00000004665
ENSPFOG00000001616
ENSGACG00000013098
ENSONIG00000017914
GI_584019220
GI_548536083
GI_554815264
GI_498964459
ENSDARG00000074201
ENSAMXG00000013411
gnb2l1
ENSLOCG00000009758
ENSGACG00000018103
ENSTNIG00000010510
ENSTRUG00000000550
ENSXMAG00000008940
ENSPFOG00000008165
ENSORLG00000005276
ENSONIG00000012984
GI_583972285
GI_548334125
GI_498935526
GI_554821010
ENSAMXG00000002584
ENSDARG00000041619
ENSAMXG00000007457
grip1
ENSLOCG00000016167
ENSGACG00000000177
ENSTRUG00000005225
ENSTNIG00000002863
ENSORLG00000017453
ENSXMAG00000006468
ENSPFOG00000012379
GI_542204218
GI_583971256
GI_554820840
GI_499031622
GI_548419148
ENSDARG00000015053
ENSAMXG00000011215

Figure S3

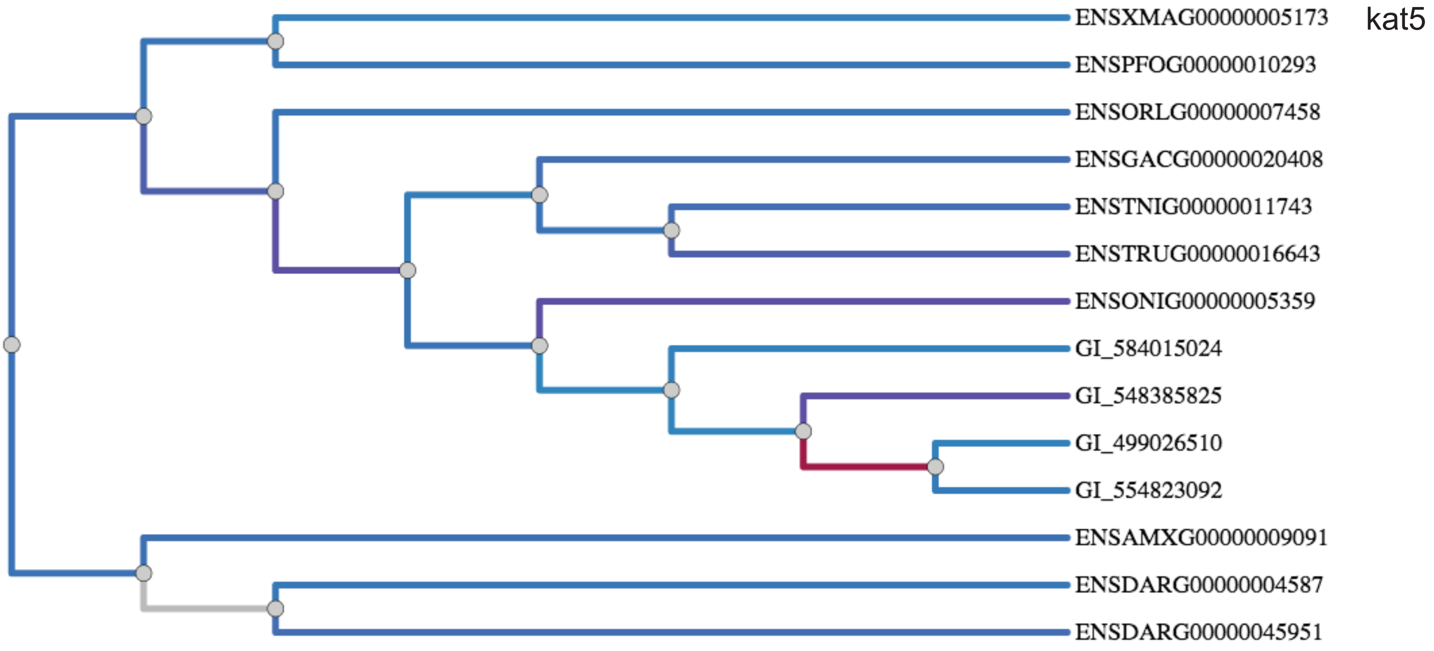
ENSXMAG00000005173
ENSPFOG00000010293
ENSORLG00000007458
ENSGACG00000020408
ENSTNIG00000011743
ENSTRUG00000016643
ENSONIG00000005359
GI_584015024
GI_548385825
GI_499026510
GI_554823092
ENSAMXG00000009091
ENSDARG00000004587
ENSDARG00000045951
kat5

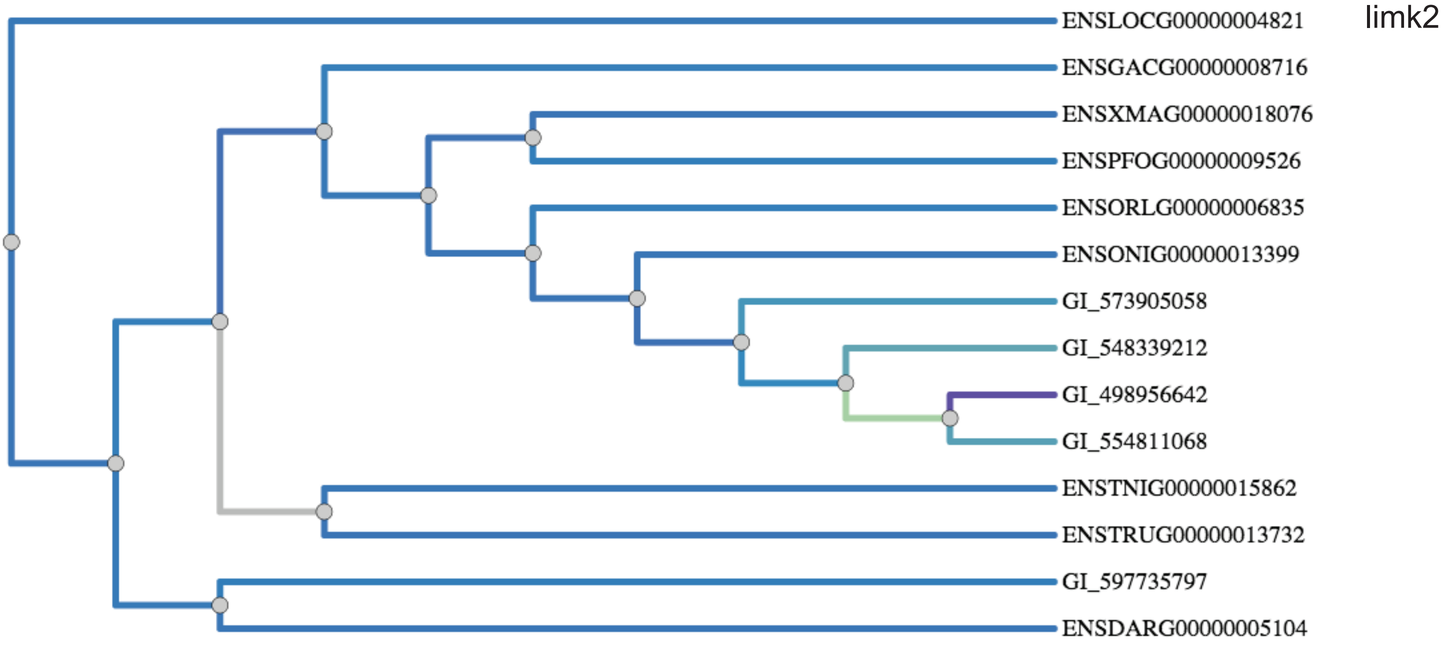
ENSLOCG00000004821
ENSGACG00000008716
ENSXMAG00000018076
ENSPFOG00000009526
ENSORLG00000006835
ENSONIG00000013399
GI_573905058
GI_548339212
GI_498956642
GI_554811068
ENSTNIG00000015862
ENSTRUG00000013732
GI_597735797
ENSDARG00000005104
limk2

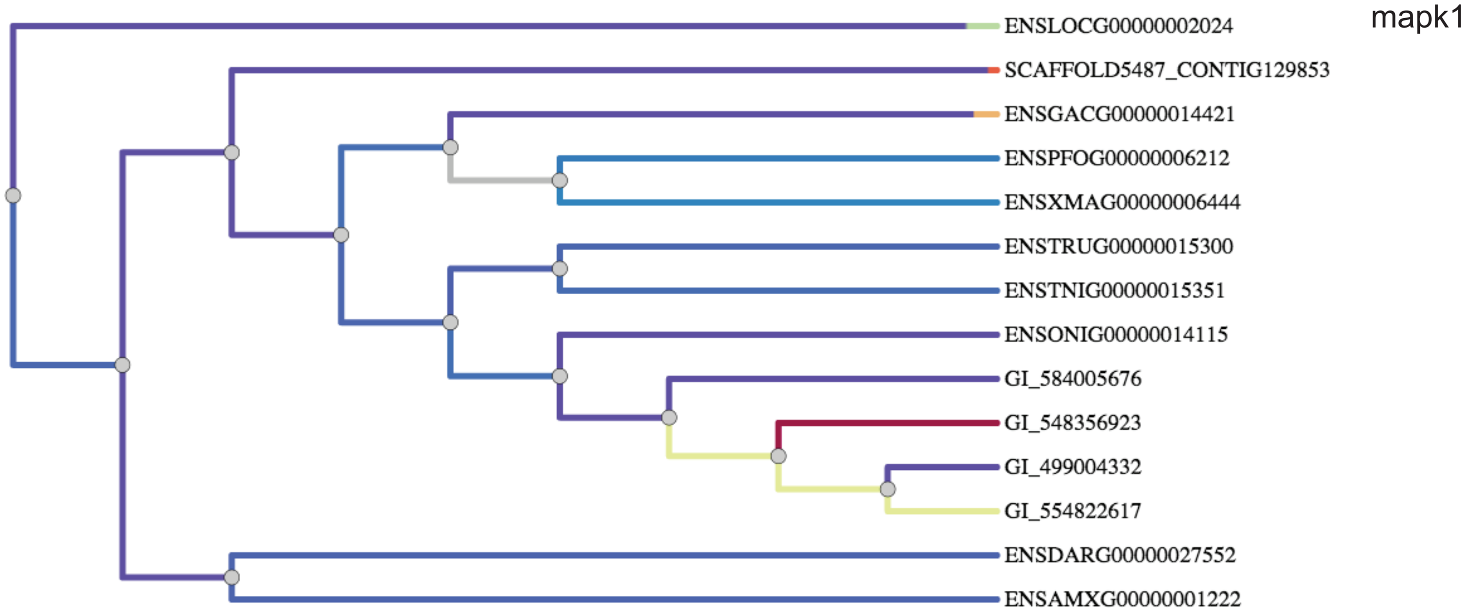
ENSLOCG00000002024
SCAFFOLD5487_CONTIG129853
ENSGACG00000014421
ENSPFOG00000006212
ENSXMAG00000006444
ENSTRUG00000015300
ENSTNIG00000015351
ENSONIG00000014115
GI_584005676
GI_548356923
GI_499004332
GI_554822617
ENSDARG00000027552
ENSAMXG00000001222
mapk1

Figure S3

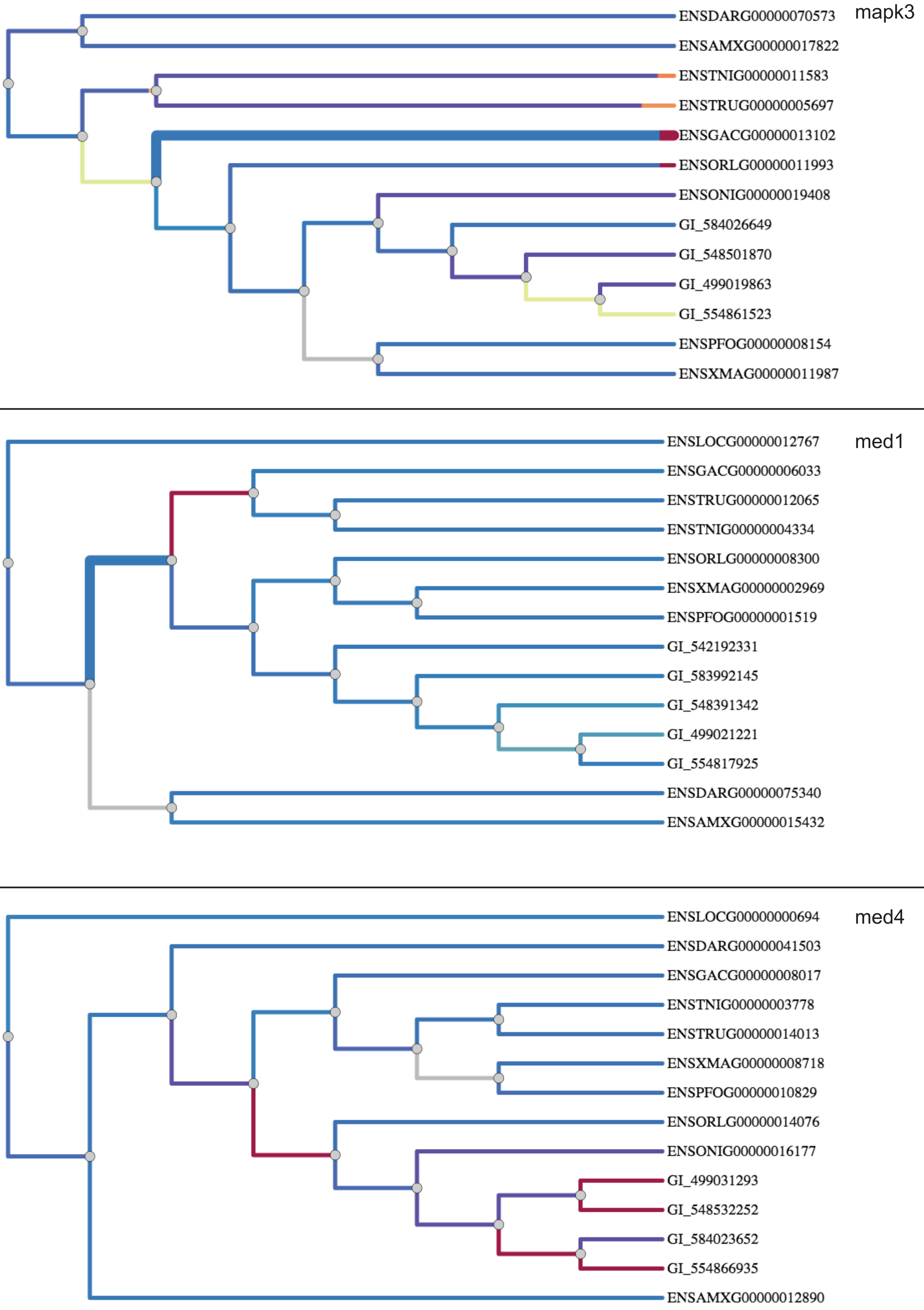

mapk3
ENSDARG00000070573
ENSAMXG00000017822
ENSTNIG00000011583
ENSTRUG00000005697
ENSGACG00000013102
ENSORLG00000011993
ENSONIG00000019408
GI_584026649
GI_548501870
GI_499019863
GI_554861523
ENSPFOG00000008154
ENSXMAG00000011987
med1
ENSLOCG00000012767
ENSGACG00000006033
ENSTRUG00000012065
ENSTNIG00000004334
ENSORLG00000008300
ENSXMAG00000002969
ENSPFOG00000001519
GI_542192331
GI_583992145
GI_548391342
GI_499021221
GI_554817925
ENSDARG00000075340
ENSAMXG00000015432
med4
ENSLOCG00000000694
ENSDARG00000041503
ENSGACG00000008017
ENSTNIG00000003778
ENSTRUG00000014013
ENSXMAG00000008718
ENSPFOG00000010829
ENSORLG00000014076
ENSONIG00000016177
GI_499031293
GI_548532252
GI_584023652
GI_554866935
ENSAMXG00000012890

Figure S3

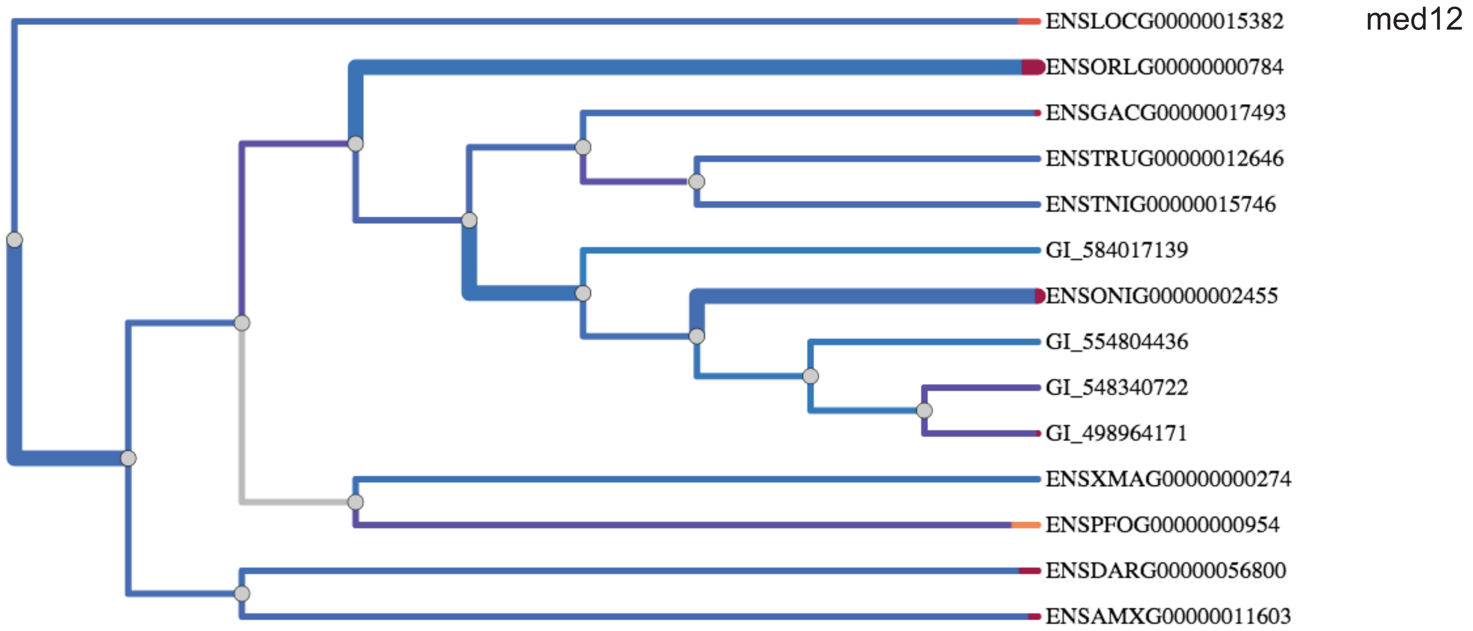

ENSLOCG00000015382
ENSORLG00000000784
ENSGACG00000017493
ENSTRUG00000012646
ENSTNIG00000015746
GI_584017139
ENSONIG00000002455
GI_554804436
GI_548340722
GI_498964171
ENSXMAG00000000274
ENSPFOG00000000954
ENSDARG00000056800
ENSAMXG00000011603
med12

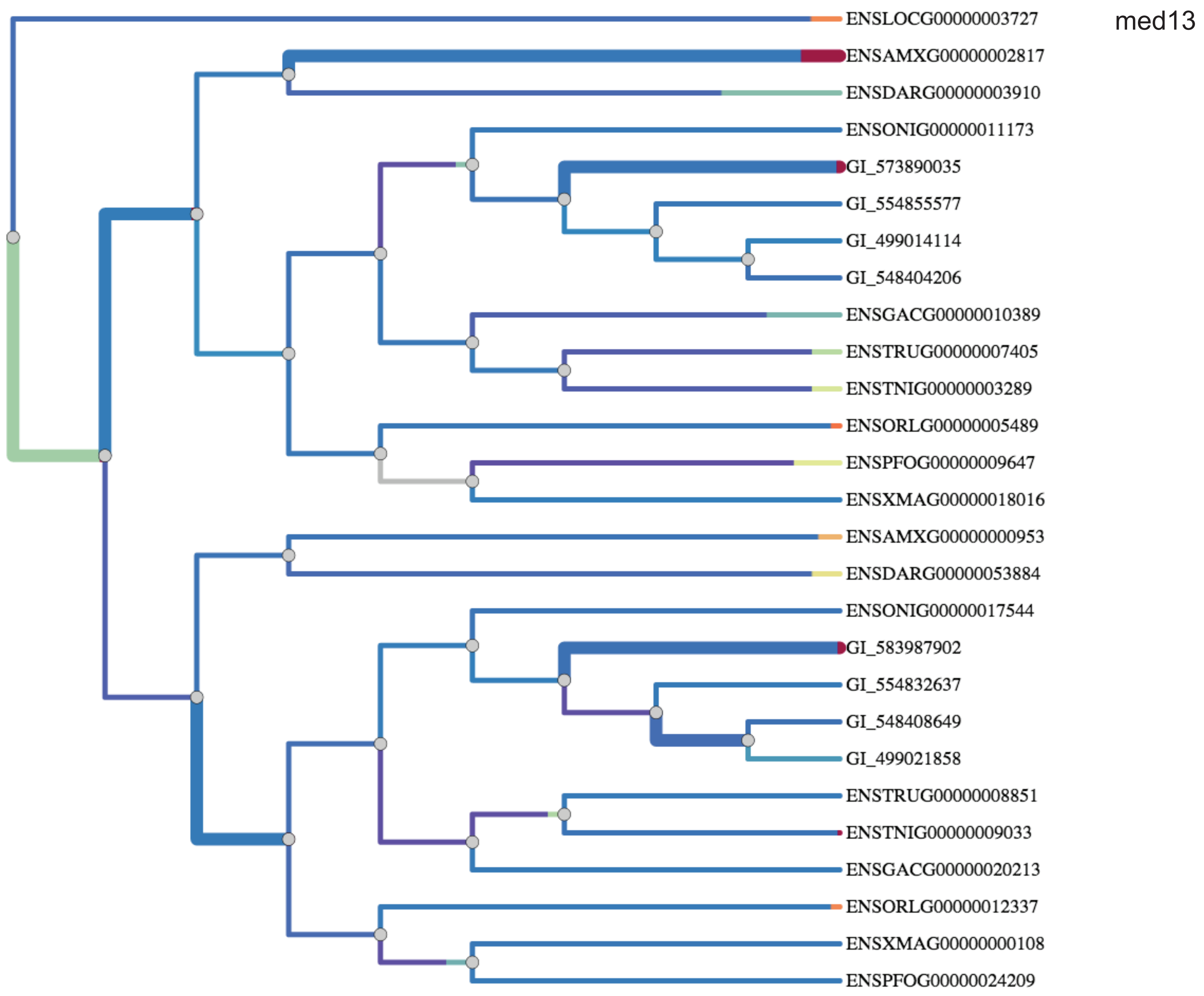

ENSLOCG00000003727
ENSAMXG00000002817
ENSDARG00000003910
ENSONIG00000011173
GI_573890035
GI_554855577
GI_499014114
GI_548404206
ENSGACG00000010389
ENSTRUG00000007405
ENSTNIG00000003289
ENSORLG00000005489
ENSPFOG00000009647
ENSXMAG00000018016
ENSAMXG00000000953
ENSDARG00000053884
ENSONIG00000017544
GI_583987902
GI_554832637
GI_548408649
GI_499021858
ENSTRUG00000008851
ENSTNIG00000009033
ENSGACG00000020213
ENSORLG00000012337
ENSXMAG00000000108
ENSPFOG00000024209
med13

Figure S3

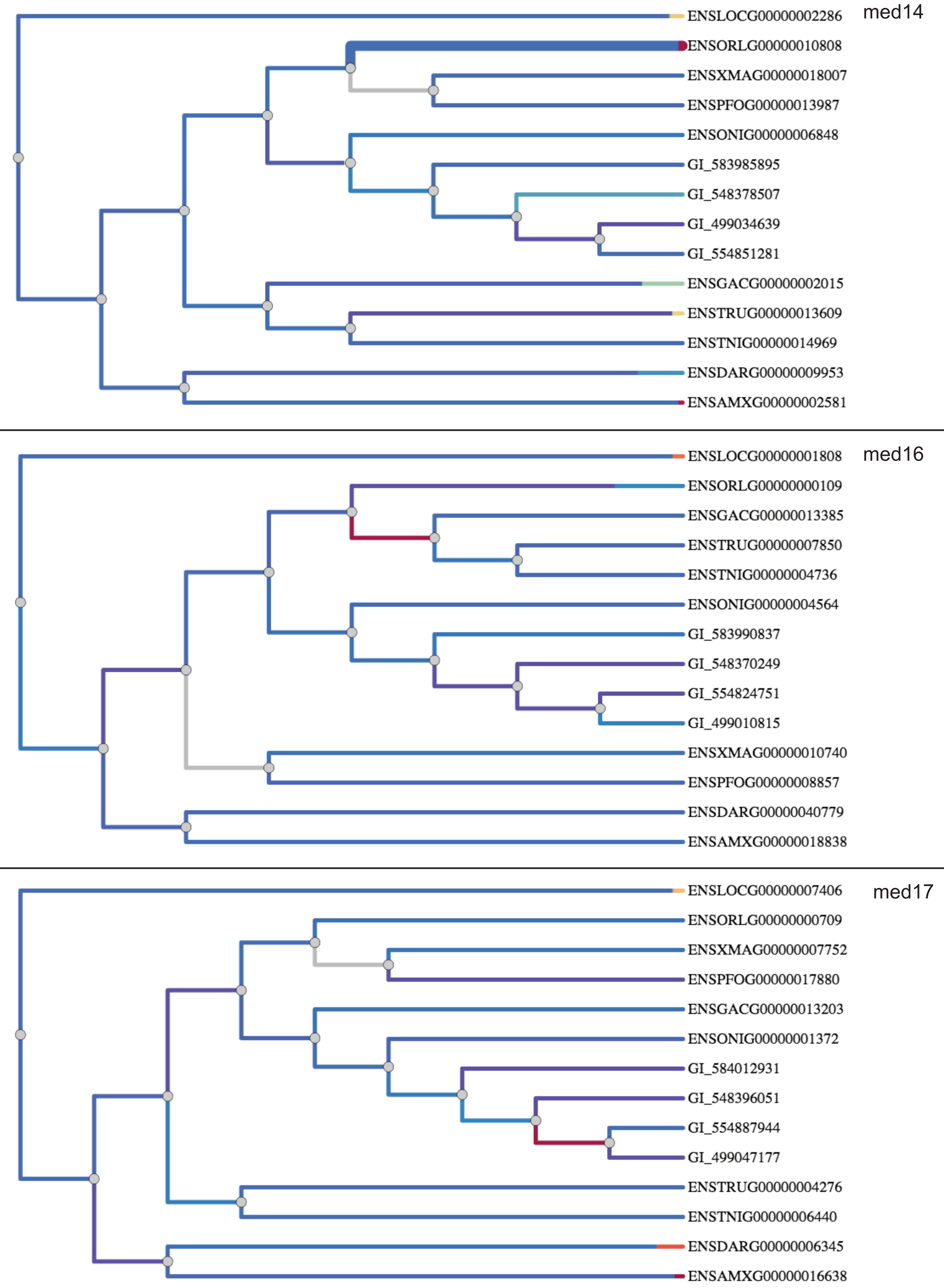
med14
ENSLOCG00000002286
ENSORLG00000010808
ENSXMAG00000018007
ENSPFOG00000013987
ENSONIG00000006848
GI_583985895
GI_548378507
GI_499034639
GI_554851281
ENSGACG00000002015
ENSTRUG00000013609
ENSTNIG00000014969
ENSDARG00000009953
ENSAMXG00000002581
med16
ENSLOCG00000001808
ENSORLG00000000109
ENSGACG00000013385
ENSTRUG00000007850
ENSTNIG00000004736
ENSONIG00000004564
GI_583990837
GI_548370249
GI_554824751
GI_499010815
ENSXMAG00000010740
ENSPFOG00000008857
ENSDARG00000040779
ENSAMXG00000018838
med17
ENSLOCG00000007406
ENSORLG00000000709
ENSXMAG00000007752
ENSPFOG00000017880
ENSGACG00000013203
ENSONIG00000001372
GI_584012931
GI_548396051
GI_554887944
GI_499047177
ENSTRUG00000004276
ENSTNIG00000006440
ENSDARG00000006345
ENSAMXG00000016638

Figure S3

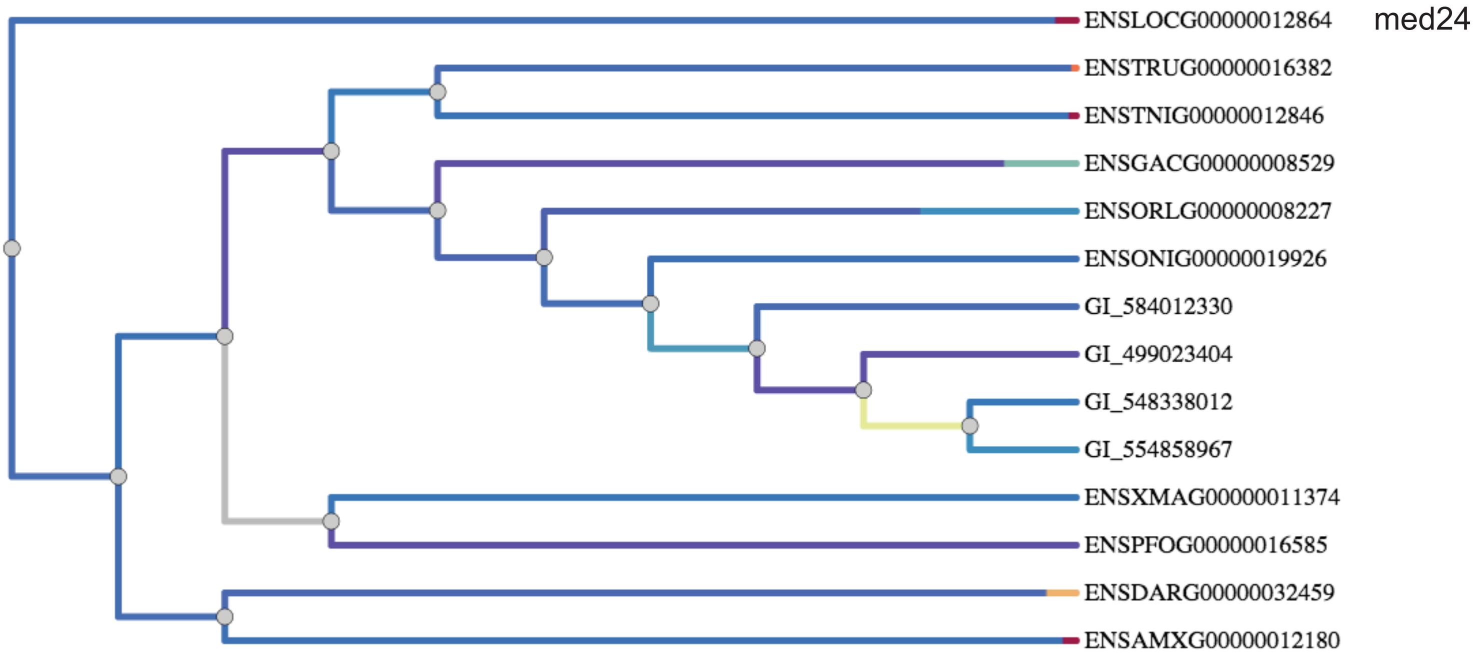

ENSLOCG00000012864
med24
ENSTRUG00000016382
ENSTNIG00000012846
ENSGACG00000008529
ENSORLG00000008227
ENSONIG00000019926
GI_584012330
GI_499023404
GI_548338012
GI_554858967
ENSXMAG00000011374
ENSPFOG00000016585
ENSDARG00000032459
ENSAMXG00000012180

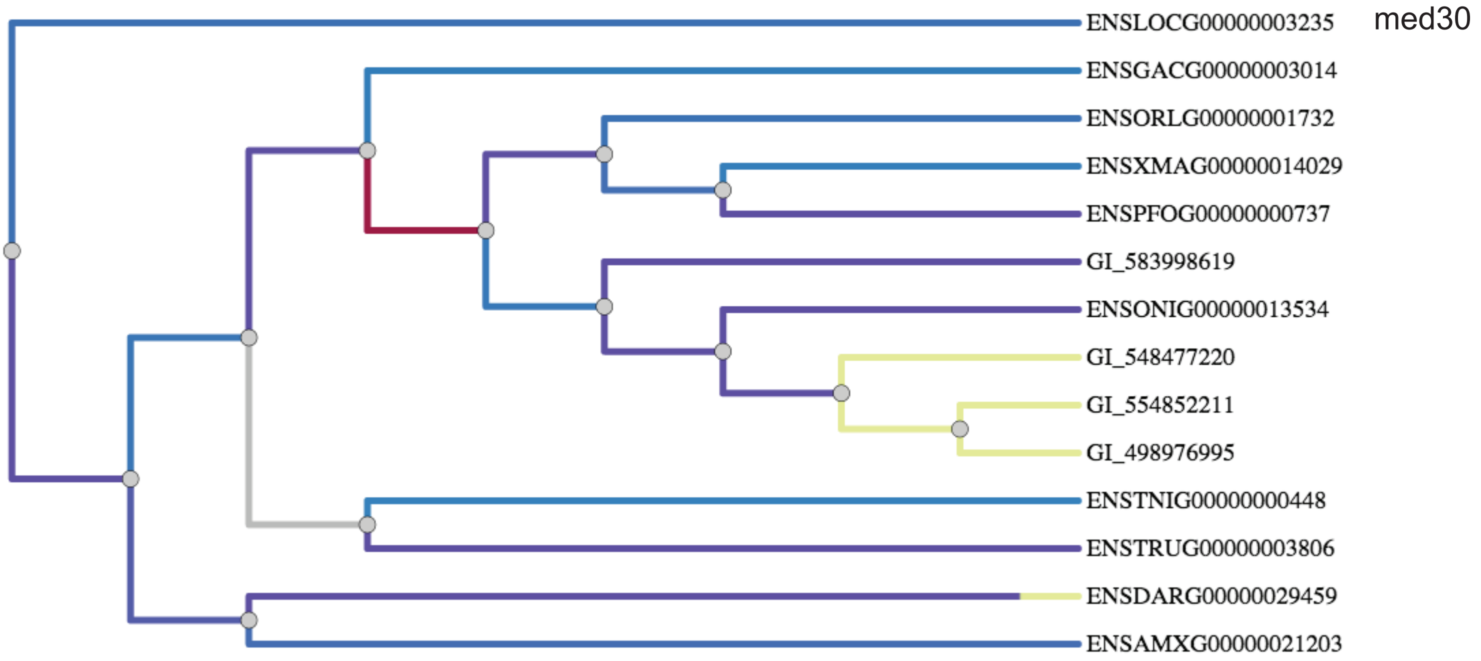

ENSLOCG00000003235
med30
ENSGACG00000003014
ENSORLG00000001732
ENSXMAG00000014029
ENSPFOG00000000737
GI_583998619
ENSONIG00000013534
GI_548477220
GI_554852211
GI_498976995
ENSTNIG00000000448
ENSTRUG00000003806
ENSDARG00000029459
ENSAMXG00000021203

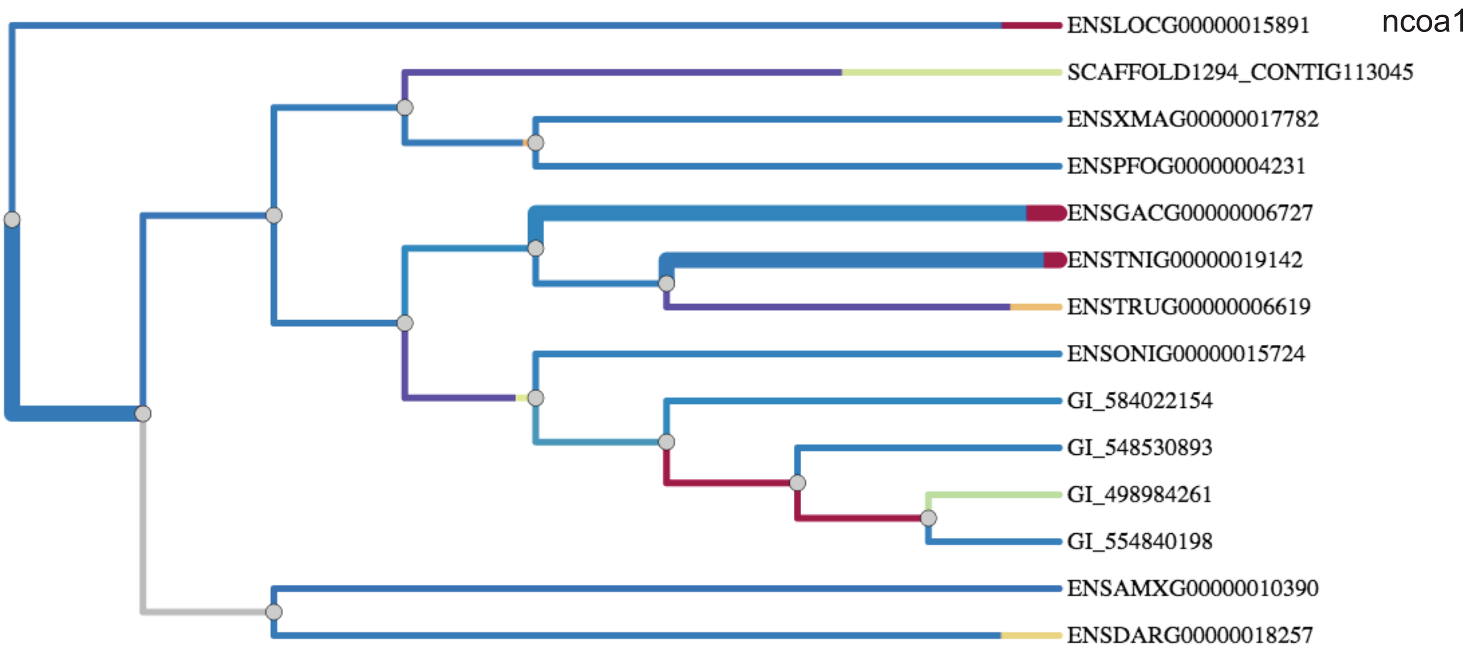

ENSLOCG00000015891
ncoa1
SCAFFOLD1294_CONTIG113045
ENSXMAG00000017782
ENSPFOG00000004231
ENSGACG00000006727
ENSTNIG00000019142
ENSTRUG00000006619
ENSONIG00000015724
GI_584022154
GI_548530893
GI_498984261
GI_554840198
ENSAMXG00000010390
ENSDARG00000018257

Figure S3

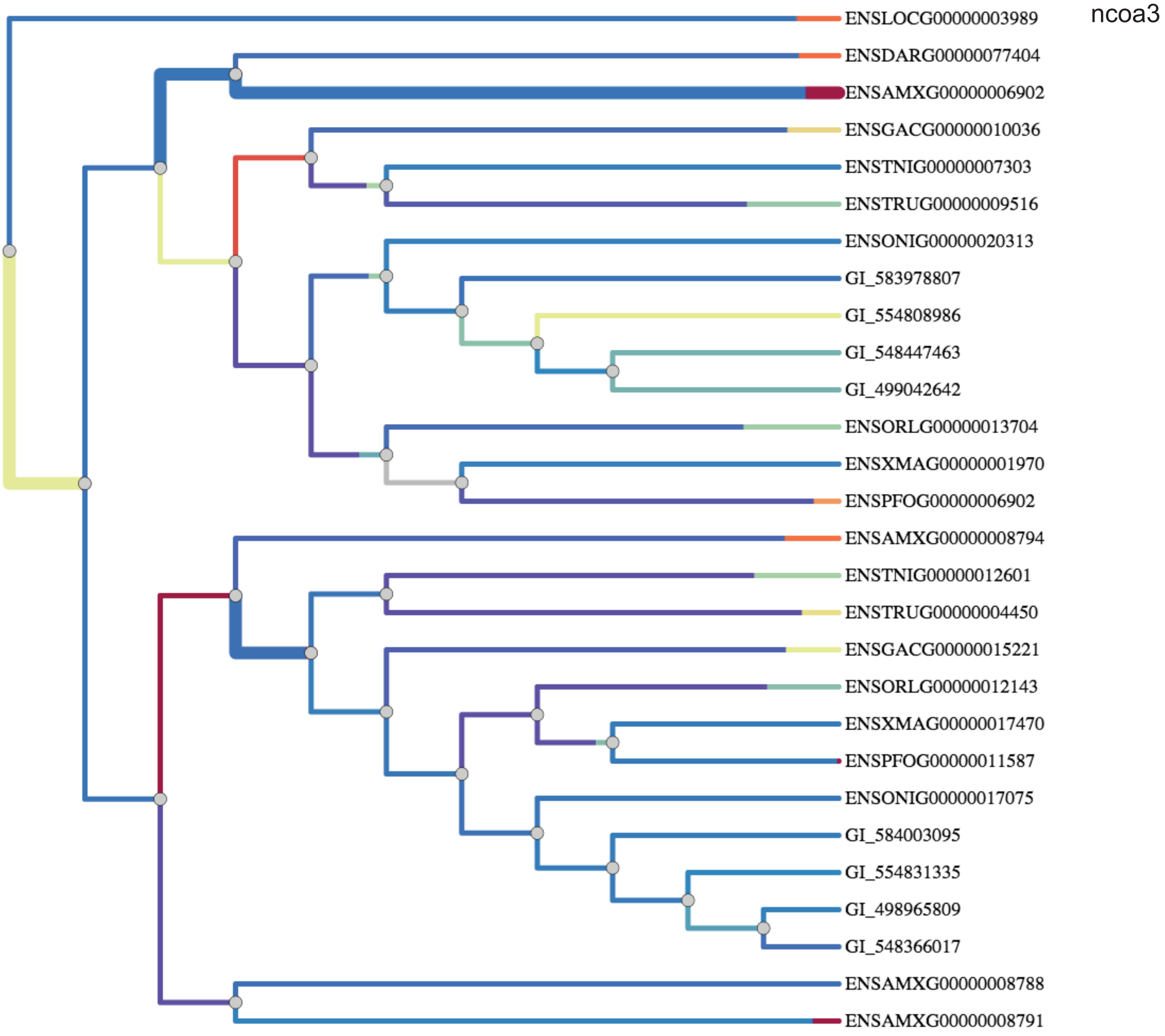

ncoa3
ENSLOCG00000003989
ENSDARG00000077404
ENSAMXG00000006902
ENSGACG00000010036
ENSTNIG00000007303
ENSTRUG00000009516
ENSONIG00000020313
GI_583978807
GI_554808986
GI_548447463
GI_499042642
ENSORLG00000013704
ENSXMAG00000001970
ENSPFOG00000006902
ENSAMXG00000008794
ENSTNIG00000012601
ENSTRUG00000004450
ENSGACG00000015221
ENSORLG00000012143
ENSXMAG00000017470
ENSPFOG00000011587
ENSONIG00000017075
GI_584003095
GI_554831335
GI_498965809
GI_548366017
ENSAMXG00000008788
ENSAMXG00000008791

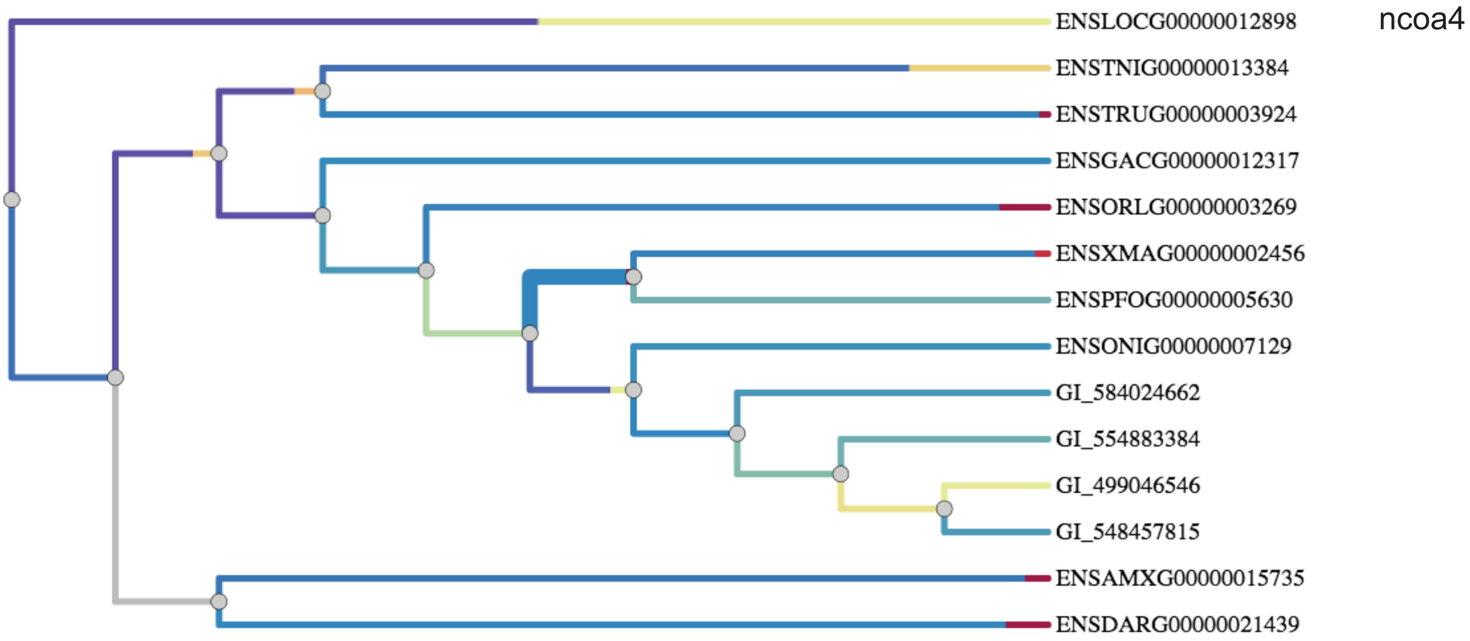

ncoa4
ENSLOCG00000012898
ENSTNIG00000013384
ENSTRUG00000003924
ENSGACG00000012317
ENSORLG00000003269
ENSXMAG00000002456
ENSPFOG00000005630
ENSONIG00000007129
GI_584024662
GI_554883384
GI_499046546
GI_548457815
ENSAMXG00000015735
ENSDARG00000021439

Figure S3

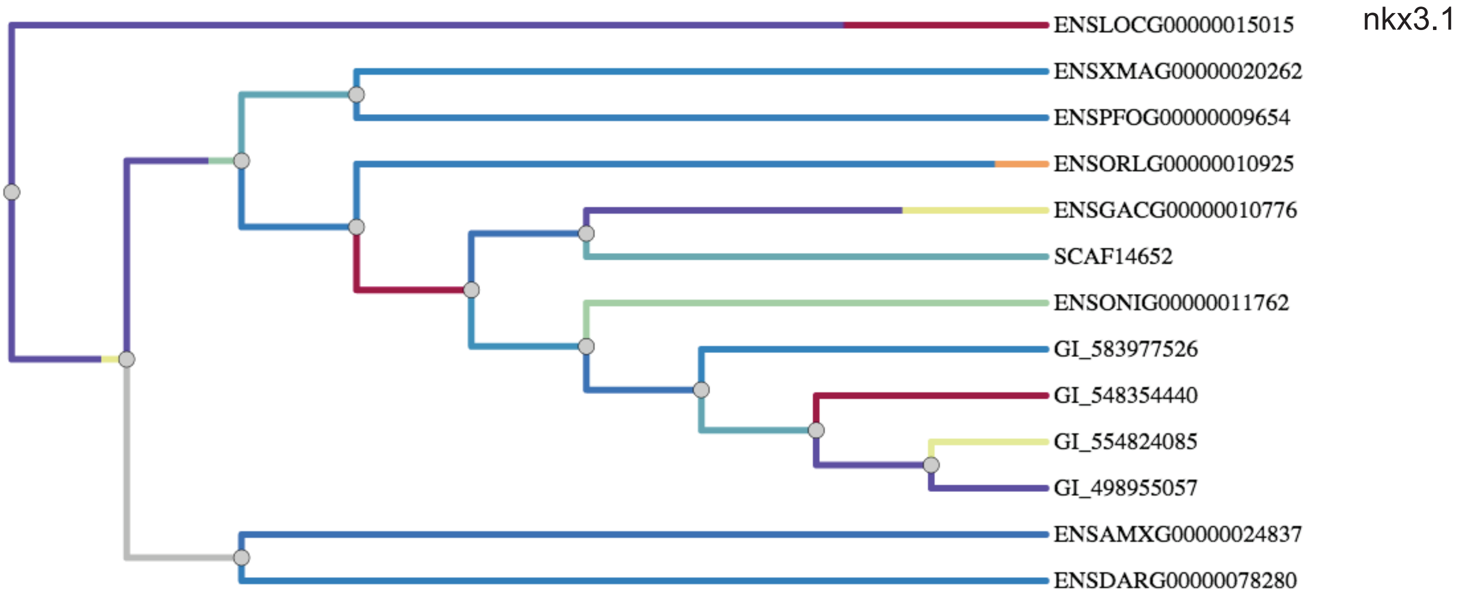
ENSLOCG00000015015
ENSXMAG00000020262
ENSPFOG00000009654
ENSORLG00000010925
ENSGACG00000010776
SCAF14652
ENSONIG00000011762
GI_583977526
GI_548354440
GI_554824085
GI_498955057
ENSAMXG00000024837
ENSDARG00000078280
nkx3.1

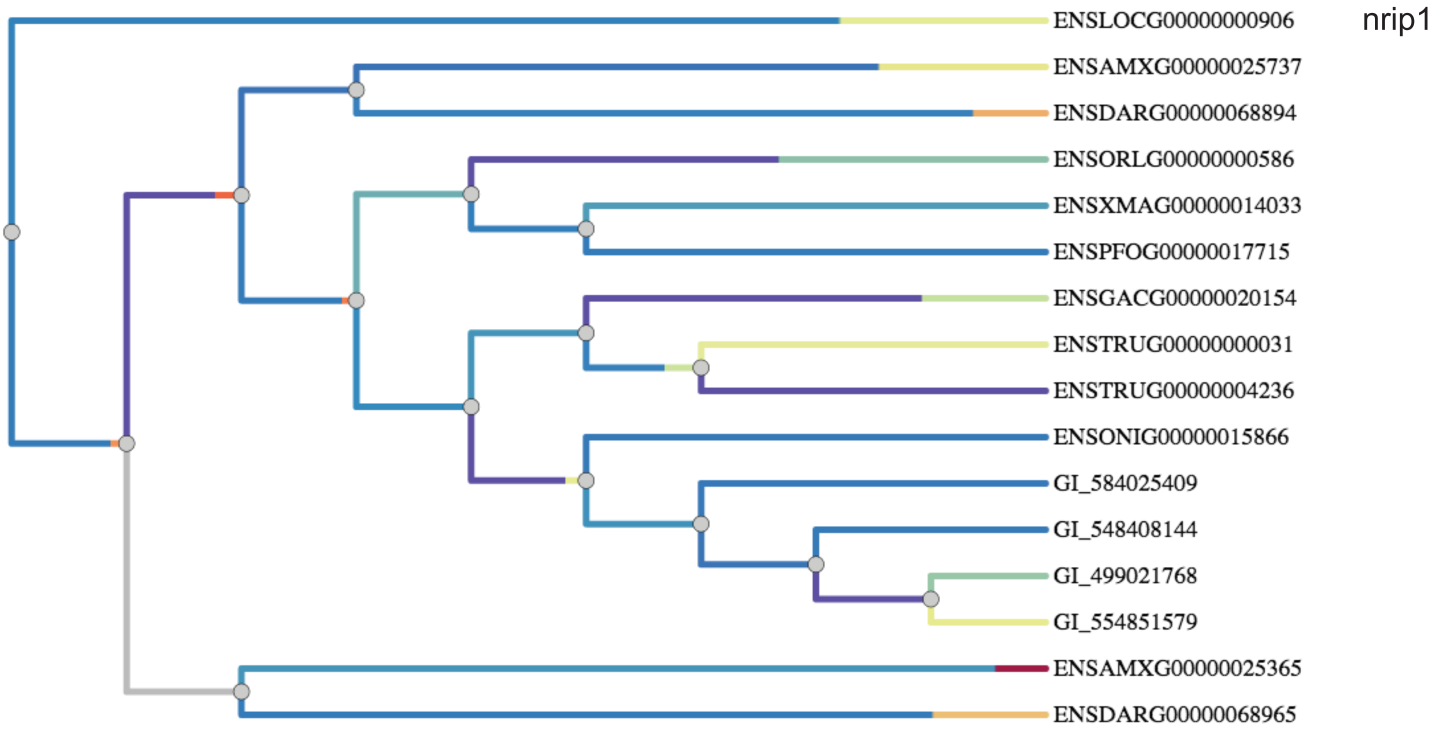
ENSLOCG00000000906
ENSAMXG00000025737
ENSDARG00000068894
ENSORLG00000000586
ENSXMAG00000014033
ENSPFOG00000017715
ENSGACG00000020154
ENSTRUG00000000031
ENSTRUG00000004236
ENSONIG00000015866
GI_584025409
GI_548408144
GI_499021768
GI_554851579
ENSAMXG00000025365
ENSDARG00000068965
nrip1

Figure S3

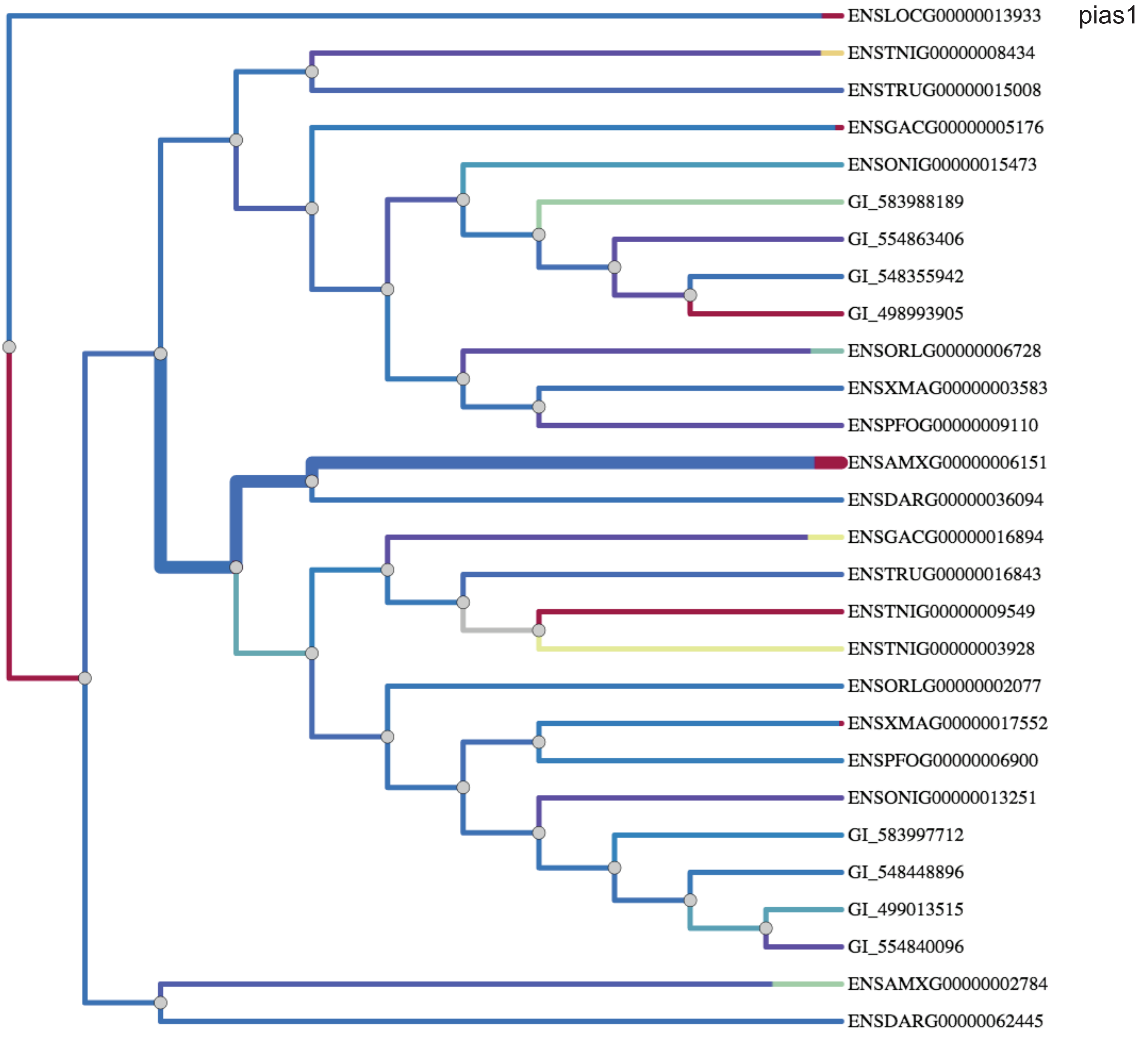

pias1
ENSLOCG00000013933
ENSTNIG00000008434
ENSTRUG00000015008
ENSGACG00000005176
ENSONIG00000015473
GI_583988189
GI_554863406
GI_548355942
GI_498993905
ENSORLG00000006728
ENSXMAG00000003583
ENSPFOG00000009110
ENSAMXG00000006151
ENSDARG00000036094
ENSGACG00000016894
ENSTRUG00000016843
ENSTNIG00000009549
ENSTNIG00000003928
ENSORLG00000002077
ENSXMAG00000017552
ENSPFOG00000006900
ENSONIG00000013251
GI_583997712
GI_548448896
GI_499013515
GI_554840096
ENSAMXG00000002784
ENSDARG00000062445

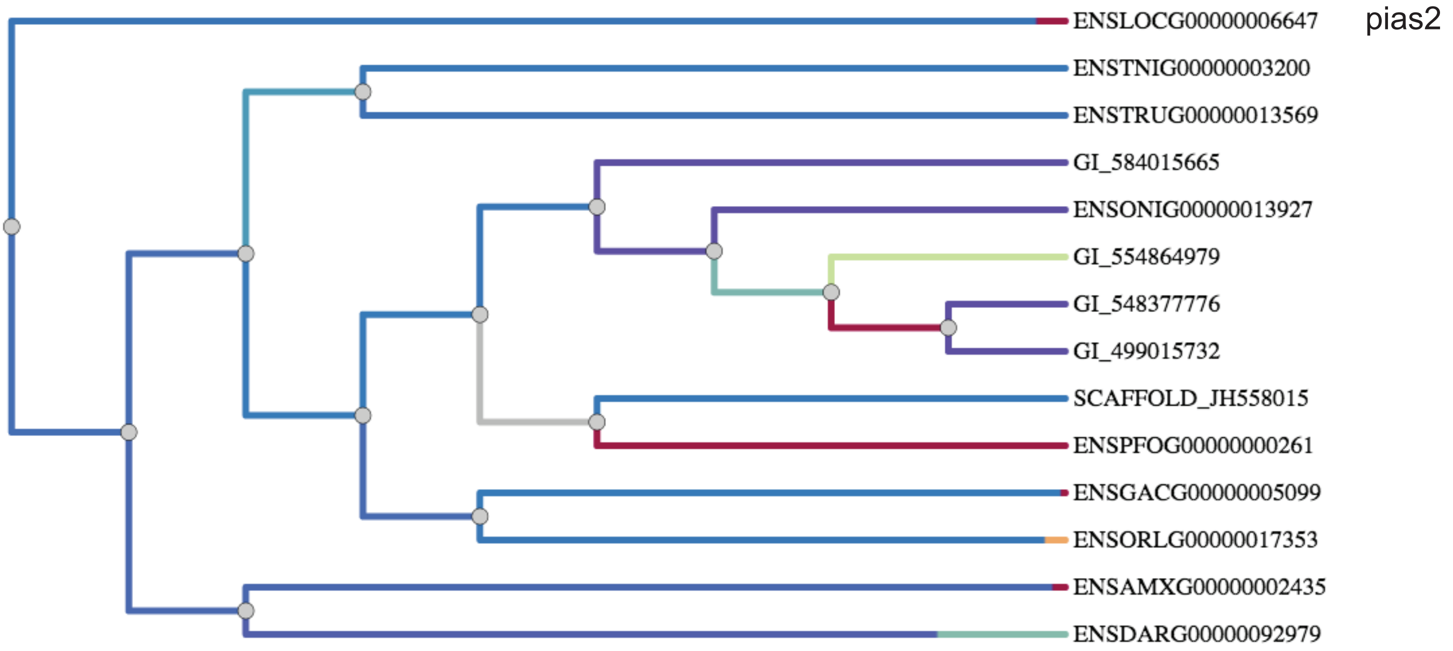

pias2
ENSLOCG00000006647
ENSTNIG00000003200
ENSTRUG00000013569
GI_584015665
ENSONIG00000013927
GI_554864979
GI_548377776
GI_499015732
SCAFFOLD_JH558015
ENSPFOG00000000261
ENSGACG00000005099
ENSORLG00000017353
ENSAMXG00000002435
ENSDARG00000092979

Figure S3

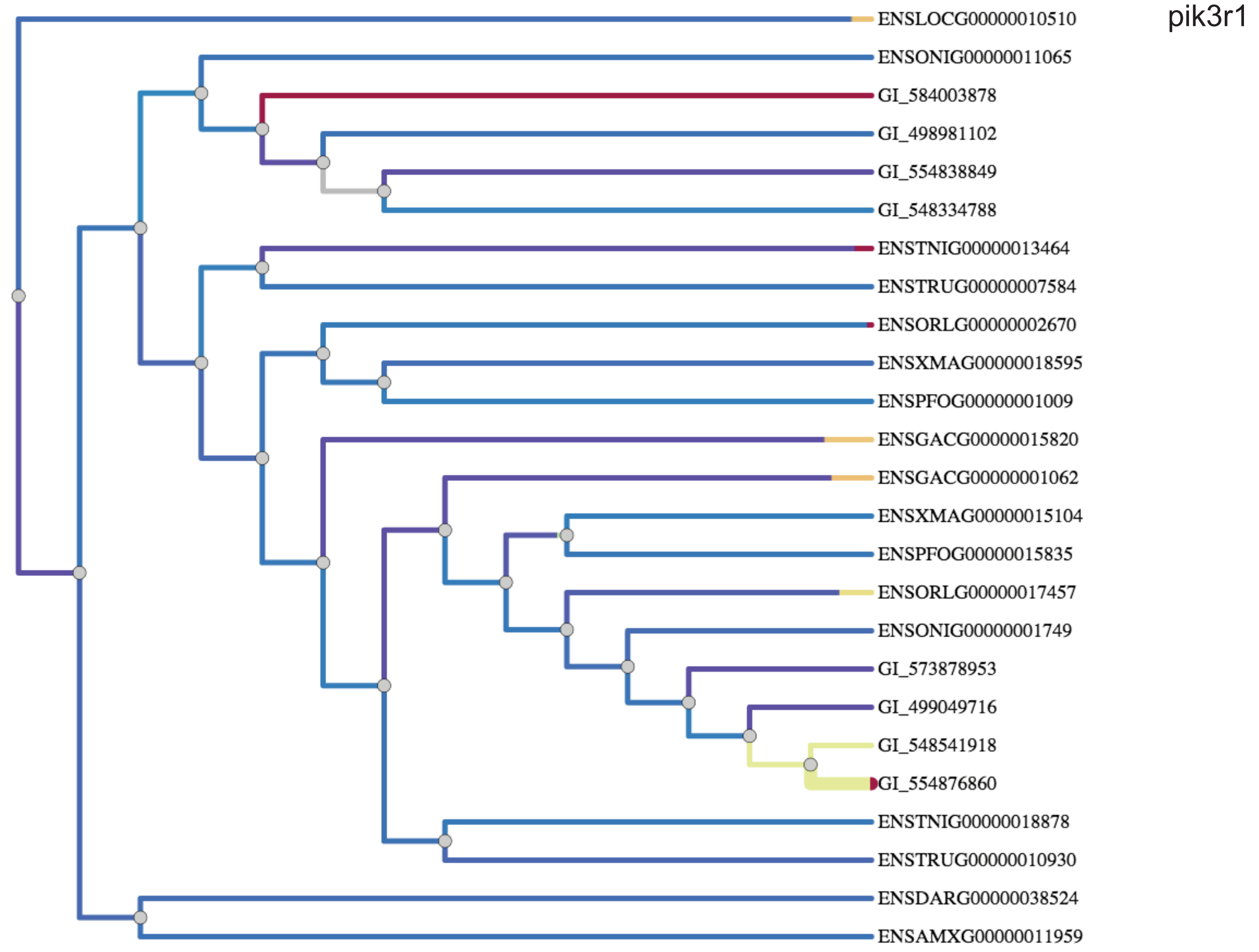

pik3r1
ENSLOCG00000010510
ENSONIG00000011065
GI_584003878
GI_498981102
GI_554838849
GI_548334788
ENSTNIG00000013464
ENSTRUG00000007584
ENSORLG00000002670
ENSXMAG00000018595
ENSPFOG00000001009
ENSGACG00000015820
ENSGACG00000001062
ENSXMAG00000015104
ENSPFOG00000015835
ENSORLG00000017457
ENSONIG00000001749
GI_573878953
GI_499049716
GI_548541918
GI_554876860
ENSTNIG00000018878
ENSTRUG00000010930
ENSDARG00000038524
ENSAMXG00000011959

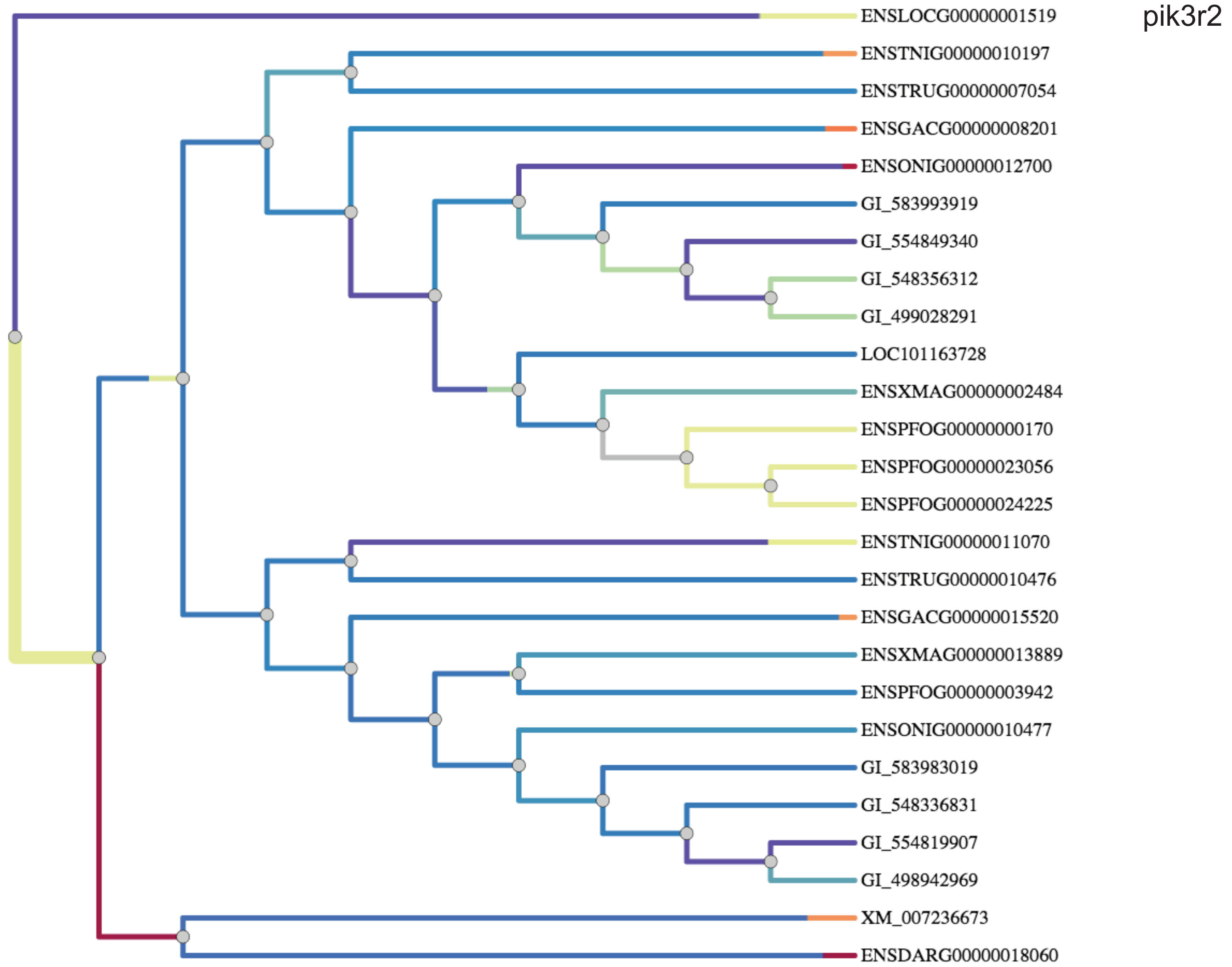

pik3r2
ENSLOCG00000001519
ENSTNIG00000010197
ENSTRUG00000007054
ENSGACG00000008201
ENSONIG00000012700
GI_583993919
GI_554849340
GI_548356312
GI_499028291
LOC101163728
ENSXMAG00000002484
ENSPFOG00000000170
ENSPFOG00000023056
ENSPFOG00000024225
ENSTNIG00000011070
ENSTRUG00000010476
ENSGACG00000015520
ENSXMAG00000013889
ENSPFOG00000003942
ENSONIG00000010477
GI_583983019
GI_548336831
GI_554819907
GI_498942969
XM_007236673
ENSDARG00000018060

Figure S3

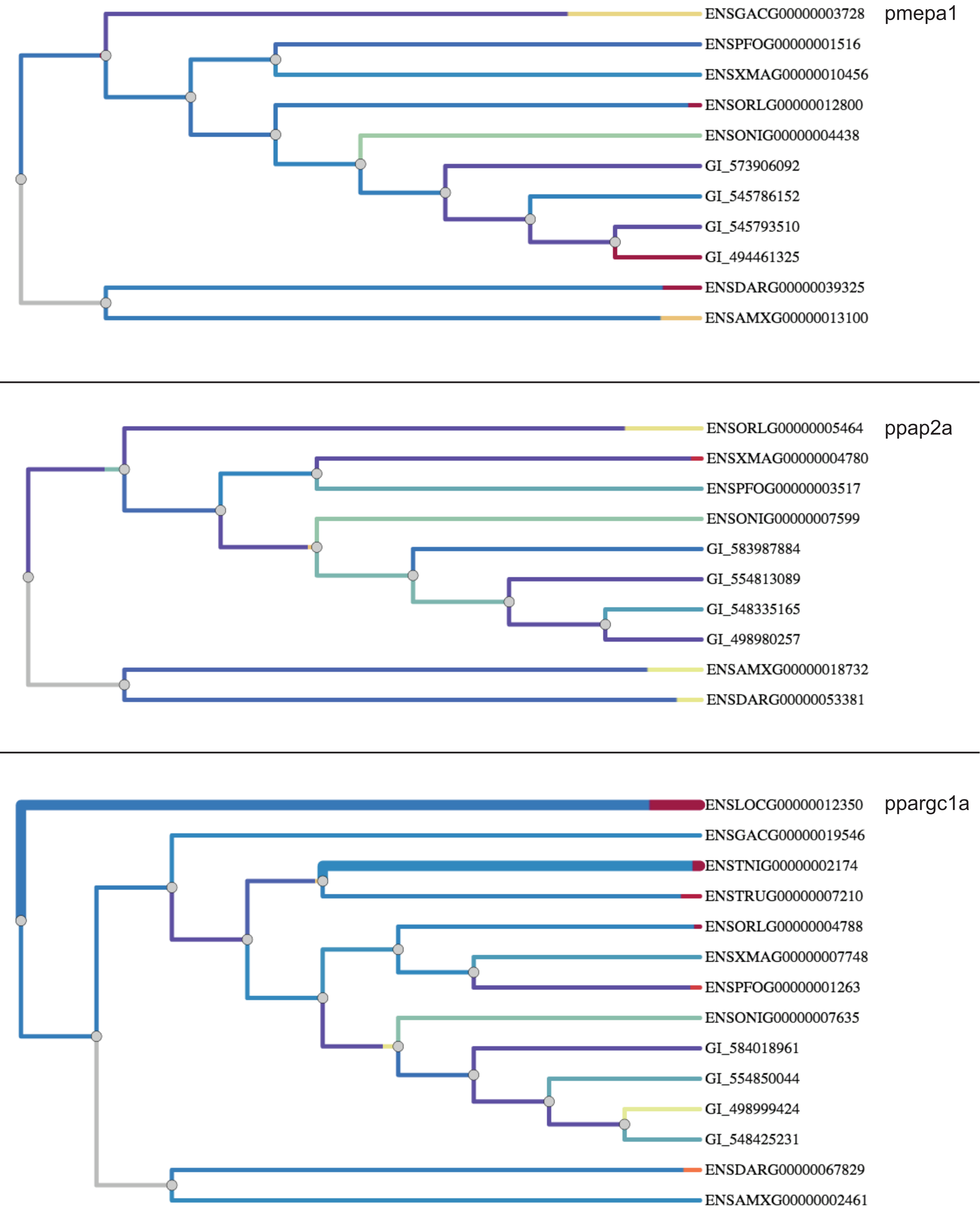

pmepa1
ENSGACG00000003728
ENSPFOG00000001516
ENSXMAG00000010456
ENSORLG00000012800
ENSONIG00000004438
GI_573906092
GI_545786152
GI_545793510
GI_494461325
ENSDARG00000039325
ENSAMXG00000013100
ppap2a
ENSORLG00000005464
ENSXMAG00000004780
ENSPFOG00000003517
ENSONIG00000007599
GI_583987884
GI_554813089
GI_548335165
GI_498980257
ENSAMXG00000018732
ENSDARG00000053381
ppargc1a
ENSLOCG00000012350
ENSGACG00000019546
ENSTNIG00000002174
ENSTRUG00000007210
ENSORLG00000004788
ENSXMAG00000007748
ENSPFOG00000001263
ENSONIG00000007635
GI_584018961
GI_554850044
GI_498999424
GI_548425231
ENSDARG00000067829
ENSAMXG00000002461

Figure S3

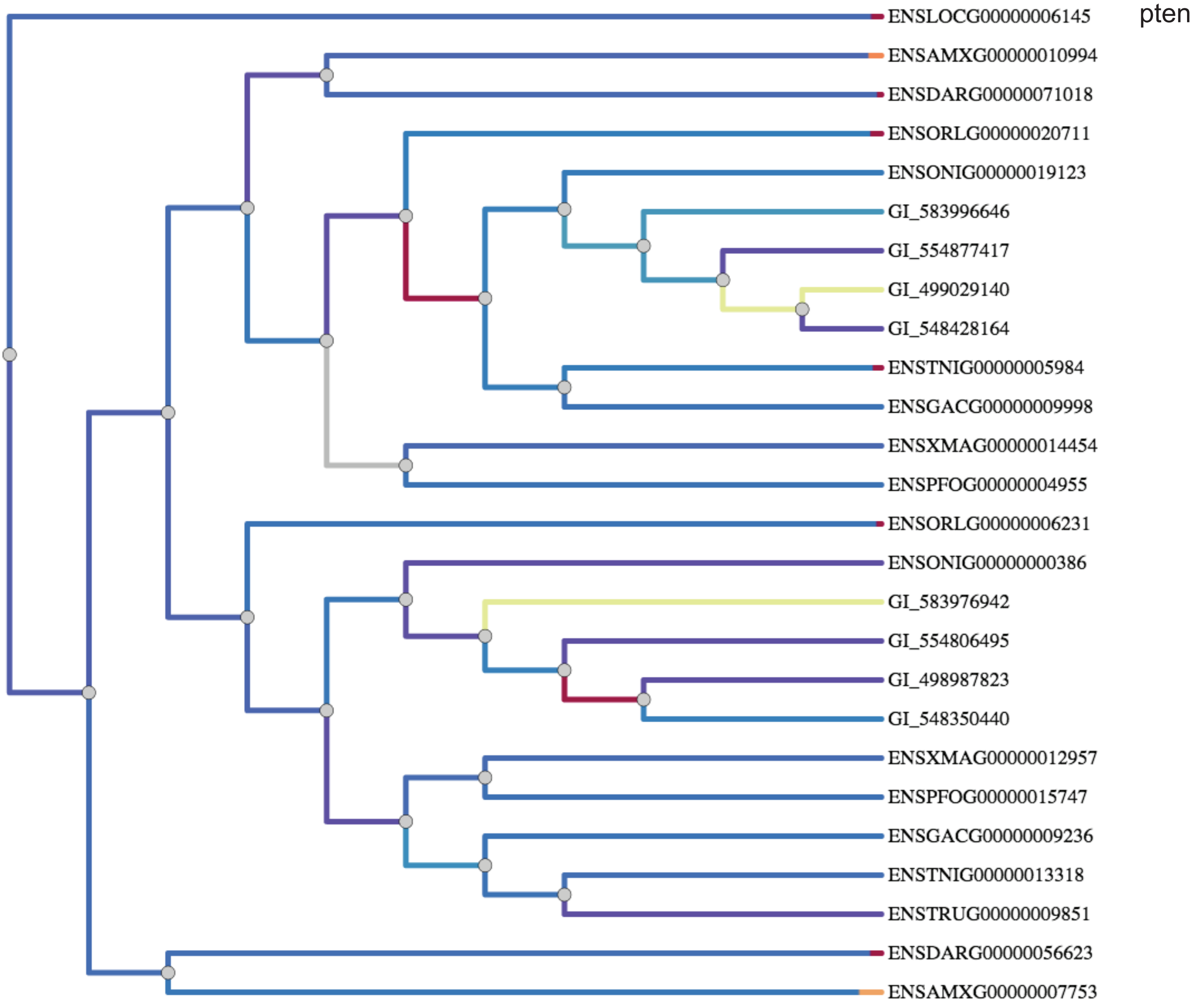

pten
ENSLOCG00000006145
ENSAMXG00000010994
ENSDARG00000071018
ENSORLG00000020711
ENSONIG00000019123
GI_583996646
GI_554877417
GI_499029140
GI_548428164
ENSTNIG00000005984
ENSGACG00000009998
ENSXMAG00000014454
ENSPFOG00000004955
ENSORLG00000006231
ENSONIG00000000386
GI_583976942
GI_554806495
GI_498987823
GI_548350440
ENSXMAG00000012957
ENSPFOG00000015747
ENSGACG00000009236
ENSTNIG00000013318
ENSTRUG00000009851
ENSDARG00000056623
ENSAMXG00000007753

Figure S3

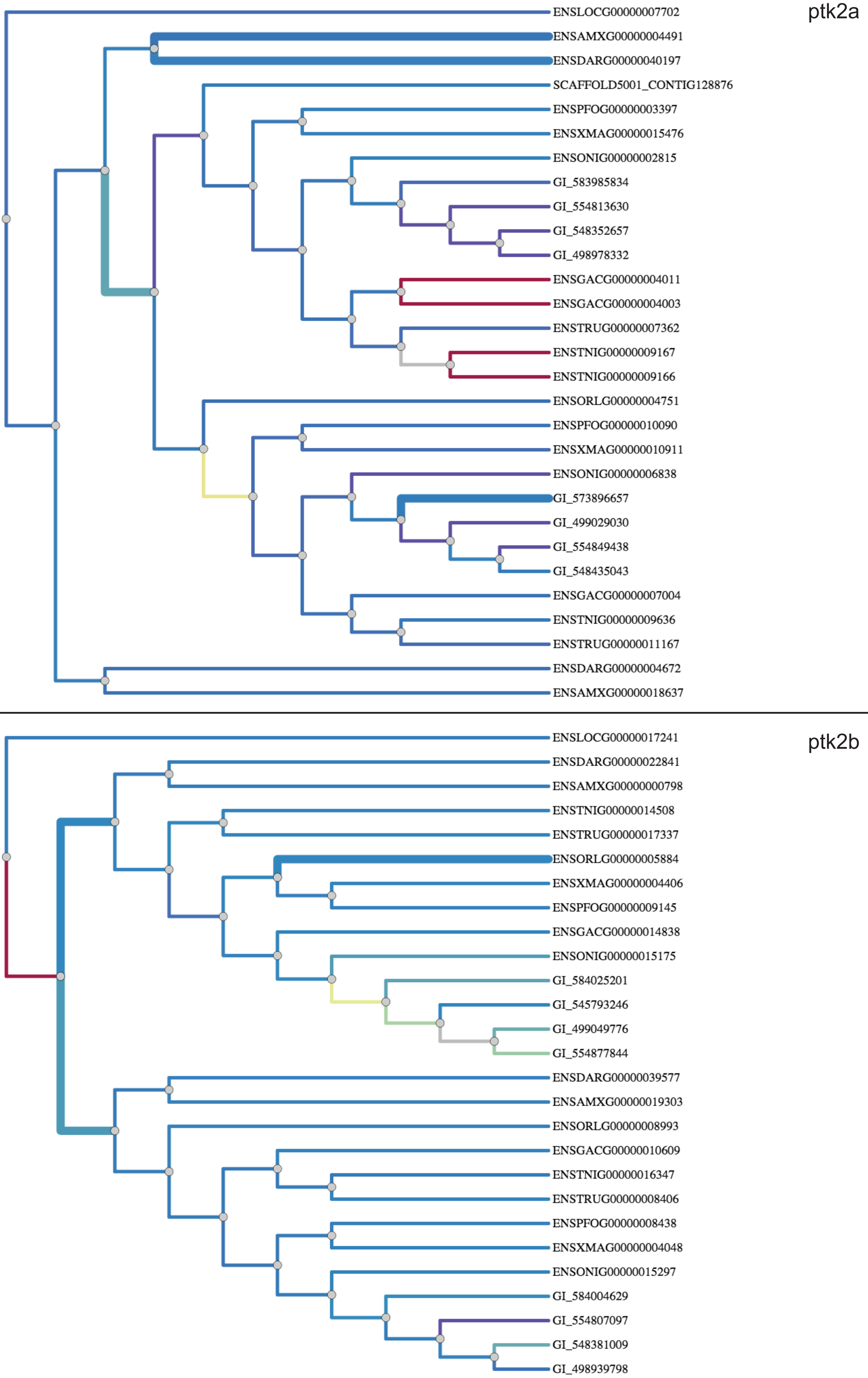

ptk2a
ENSLOCG00000007702
ENSAMXG00000004491
ENSDARG00000040197
SCAFFOLD5001_CONTIG128876
ENSPFOG00000003397
ENSXMAG00000015476
ENSONIG00000002815
GI_583985834
GI_554813630
GI_548352657
GI_498978332
ENSGACG00000004011
ENSGACG00000004003
ENSTRUG00000007362
ENSTNIG00000009167
ENSTNIG00000009166
ENSORLG00000004751
ENSPFOG00000010090
ENSXMAG00000010911
ENSONIG00000006838
GI_573896657
GI_499029030
GI_554849438
GI_548435043
ENSGACG00000007004
ENSTNIG00000009636
ENSTRUG00000011167
ENSDARG00000004672
ENSAMXG00000018637
ptk2b
ENSLOCG00000017241
ENSDARG00000022841
ENSAMXG00000000798
ENSTNIG00000014508
ENSTRUG00000017337
ENSORLG00000005884
ENSXMAG00000004406
ENSPFOG00000009145
ENSGACG00000014838
ENSONIG00000015175
GI_584025201
GI_545793246
GI_499049776
GI_554877844
ENSDARG00000039577
ENSAMXG00000019303
ENSORLG00000008993
ENSGACG00000010609
ENSTNIG00000016347
ENSTRUG00000008406
ENSPFOG00000008438
ENSXMAG00000004048
ENSONIG00000015297
GI_584004629
GI_554807097
GI_548381009
GI_498939798

Figure S3

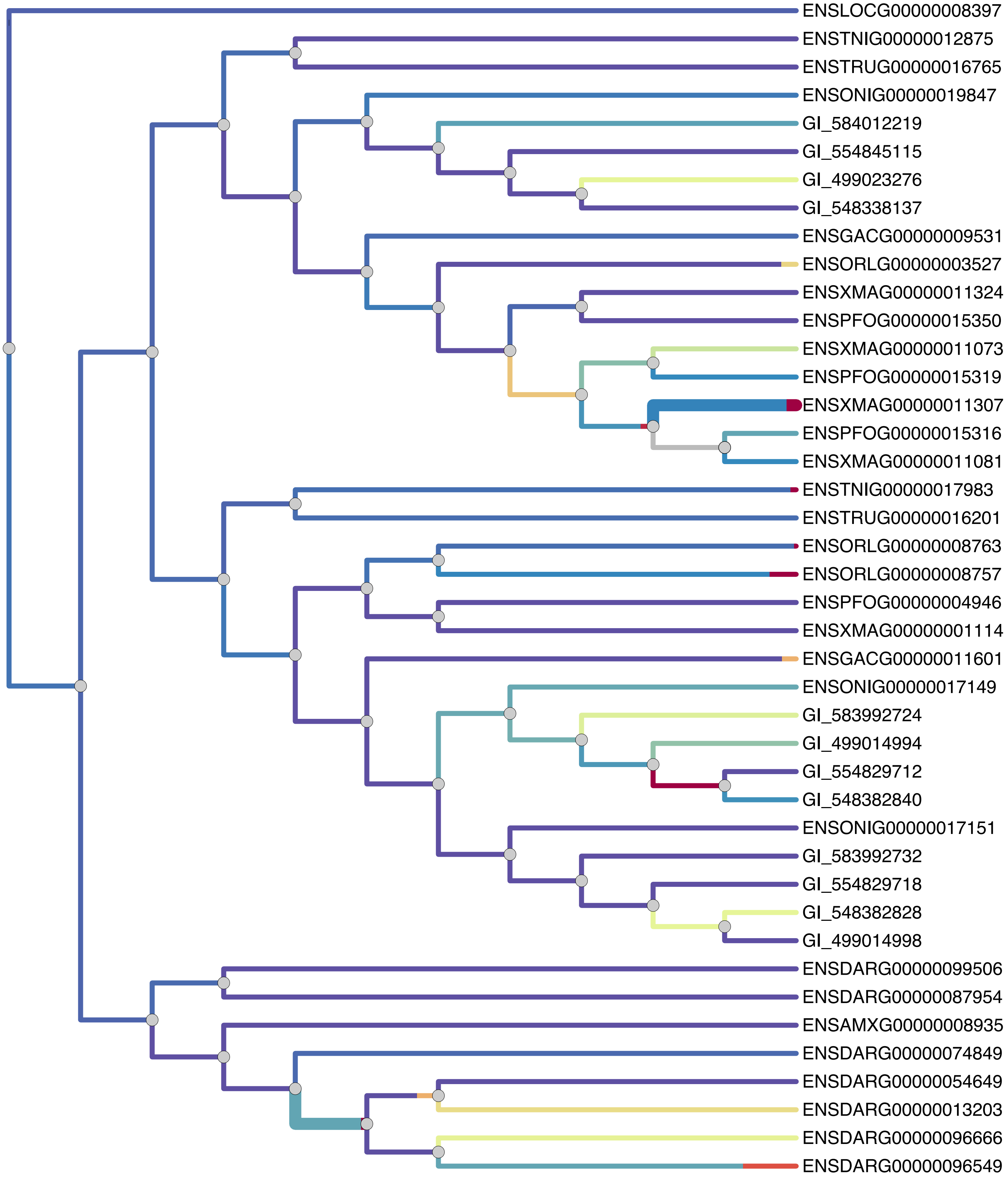

rac1

Figure S3

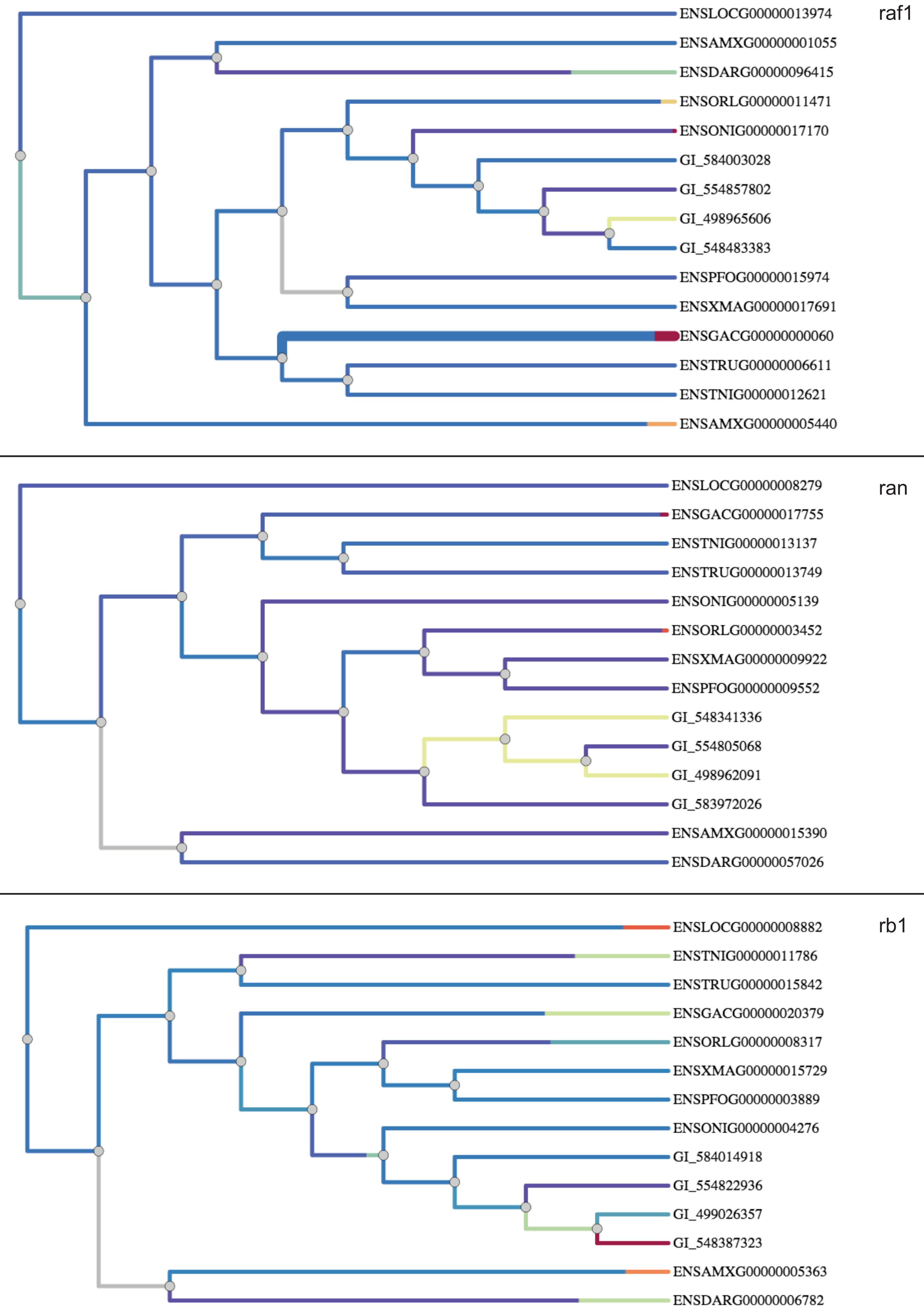
raf1
ENSLOCG00000013974
ENSAMXG00000001055
ENSDARG00000096415
ENSORLG00000011471
ENSONIG00000017170
GI_584003028
GI_554857802
GI_498965606
GI_548483383
ENSPFOG00000015974
ENSXMAG00000017691
ENSGACG00000000060
ENSTRUG00000006611
ENSTNIG00000012621
ENSAMXG00000005440
ran
ENSLOCG00000008279
ENSGACG00000017755
ENSTNIG00000013137
ENSTRUG00000013749
ENSONIG00000005139
ENSORLG00000003452
ENSXMAG00000009922
ENSPFOG00000009552
GI_548341336
GI_554805068
GI_498962091
GI_583972026
ENSAMXG00000015390
ENSDARG00000057026
rb1
ENSLOCG00000008882
ENSTNIG00000011786
ENSTRUG00000015842
ENSGACG00000020379
ENSORLG00000008317
ENSXMAG00000015729
ENSPFOG00000003889
ENSONIG00000004276
GI_584014918
GI_554822936
GI_499026357
GI_548387323
ENSAMXG00000005363
ENSDARG00000006782

Figure S3

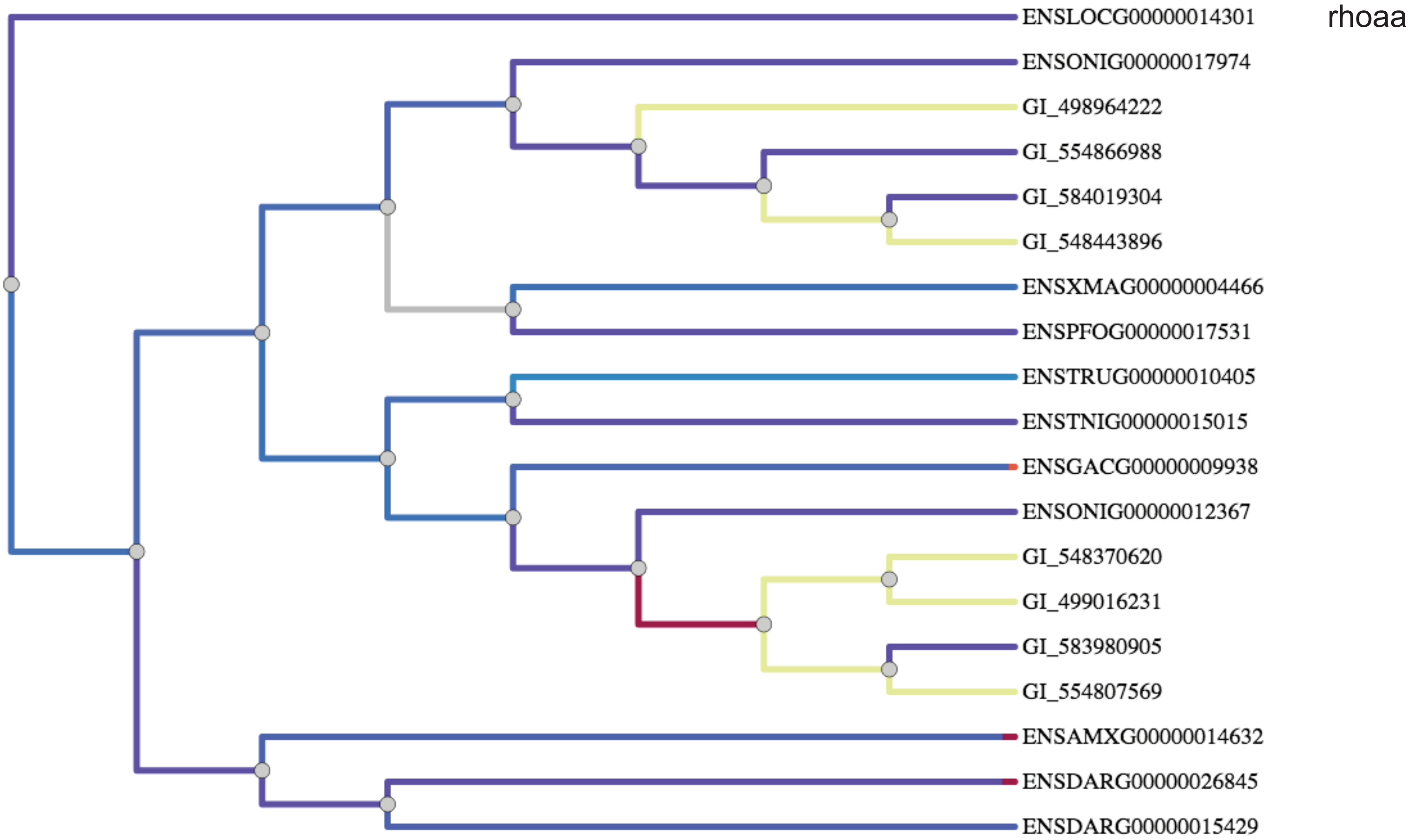
ENSLOCG00000014301
ENSONIG00000017974
GI_498964222
GI_554866988
GI_584019304
GI_548443896
ENSXMAG00000004466
ENSPFOG00000017531
ENSTRUG00000010405
ENSTNIG00000015015
ENSGACG00000009938
ENSONIG00000012367
GI_548370620
GI_499016231
GI_583980905
GI_554807569
ENSAMXG00000014632
ENSDARG00000026845
ENSDARG00000015429
rhoaa

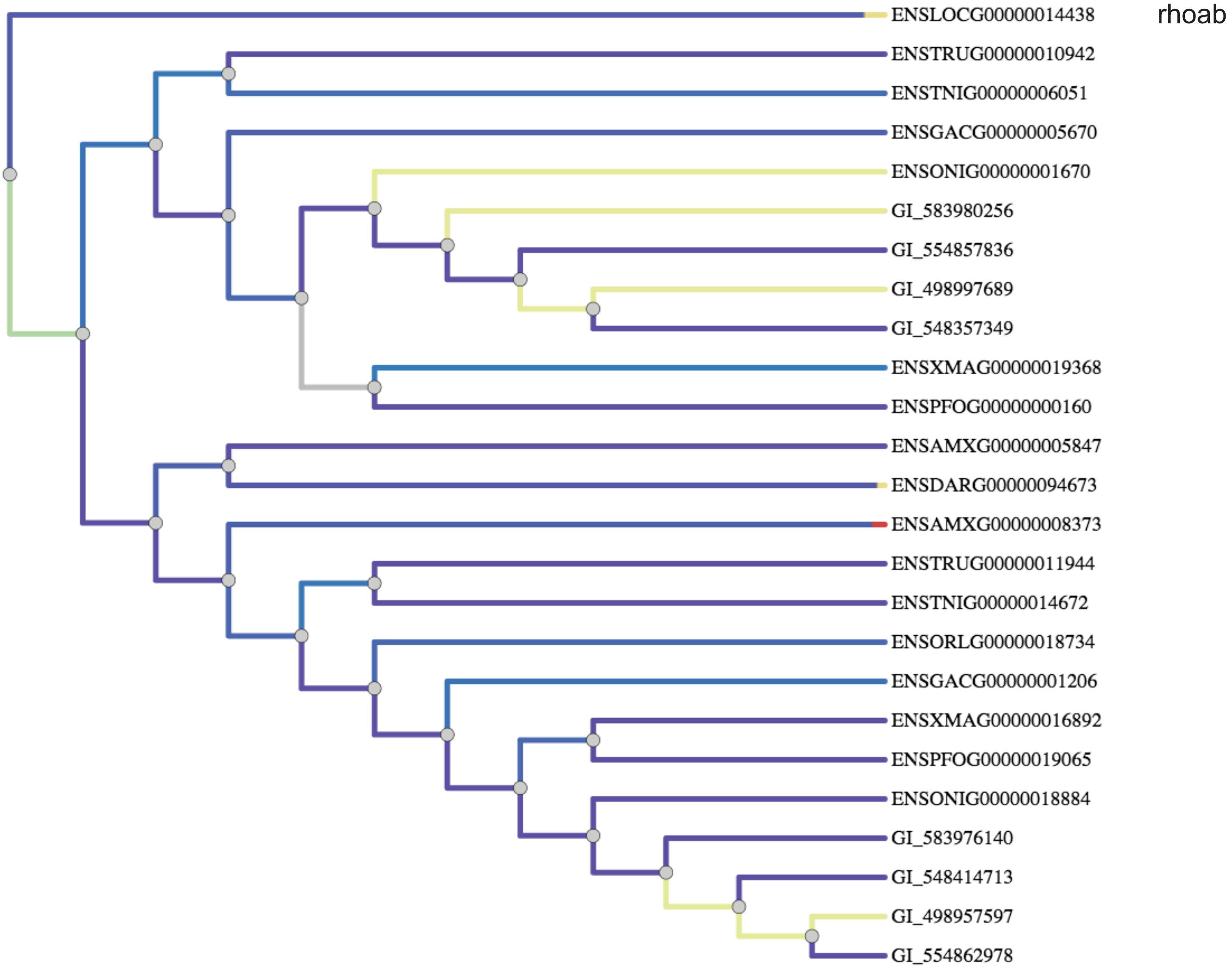
ENSLOCG00000014438
ENSTRUG00000010942
ENSTNIG00000006051
ENSGACG00000005670
ENSONIG00000001670
GI_583980256
GI_554857836
GI_498997689
GI_548357349
ENSXMAG00000019368
ENSPFOG00000000160
ENSAMXG00000005847
ENSDARG00000094673
ENSAMXG00000008373
ENSTRUG00000011944
ENSTNIG00000014672
ENSORLG00000018734
ENSGACG00000001206
ENSXMAG00000016892
ENSPFOG00000019065
ENSONIG00000018884
GI_583976140
GI_548414713
GI_498957597
GI_554862978
rhoab

Figure S3

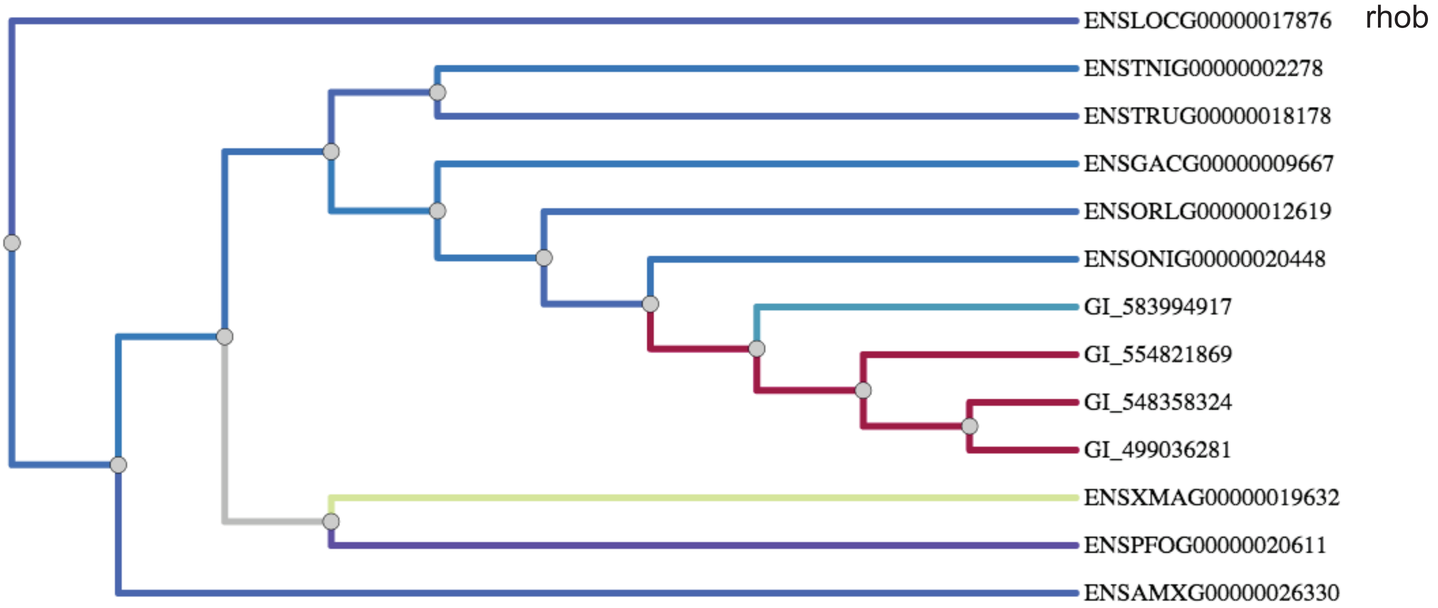
ENSLOCG00000017876
rhob
ENSTNIG00000002278
ENSTRUG00000018178
ENSGACG00000009667
ENSORLG00000012619
ENSONIG00000020448
GI_583994917
GI_554821869
GI_548358324
GI_499036281
ENSXMAG00000019632
ENSPFOG00000020611
ENSAMXG00000026330

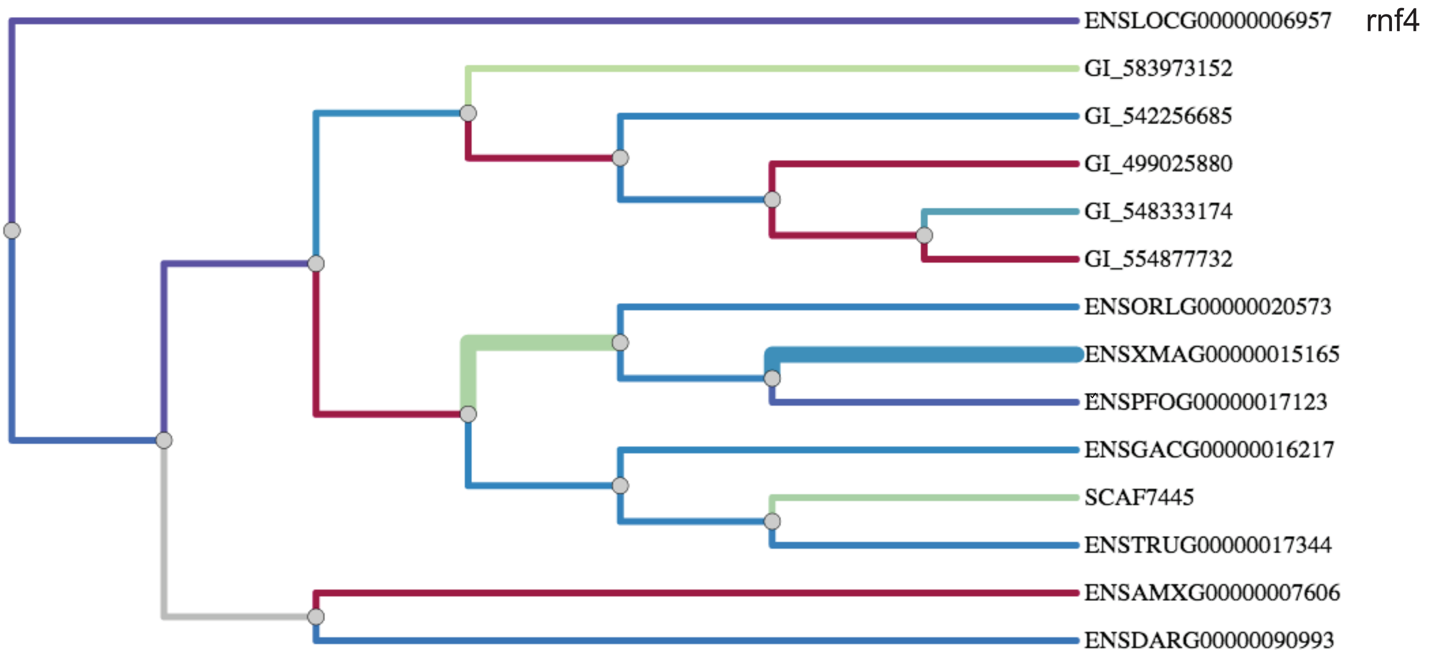
ENSLOCG00000006957
rnf4
GI_583973152
GI_542256685
GI_499025880
GI_548333174
GI_554877732
ENSORLG00000020573
ENSXMAG00000015165
ENSPFOG00000017123
ENSGACG00000016217
SCAF7445
ENSTRUG00000017344
ENSAMXG00000007606
ENSDARG00000090993

Figure S3

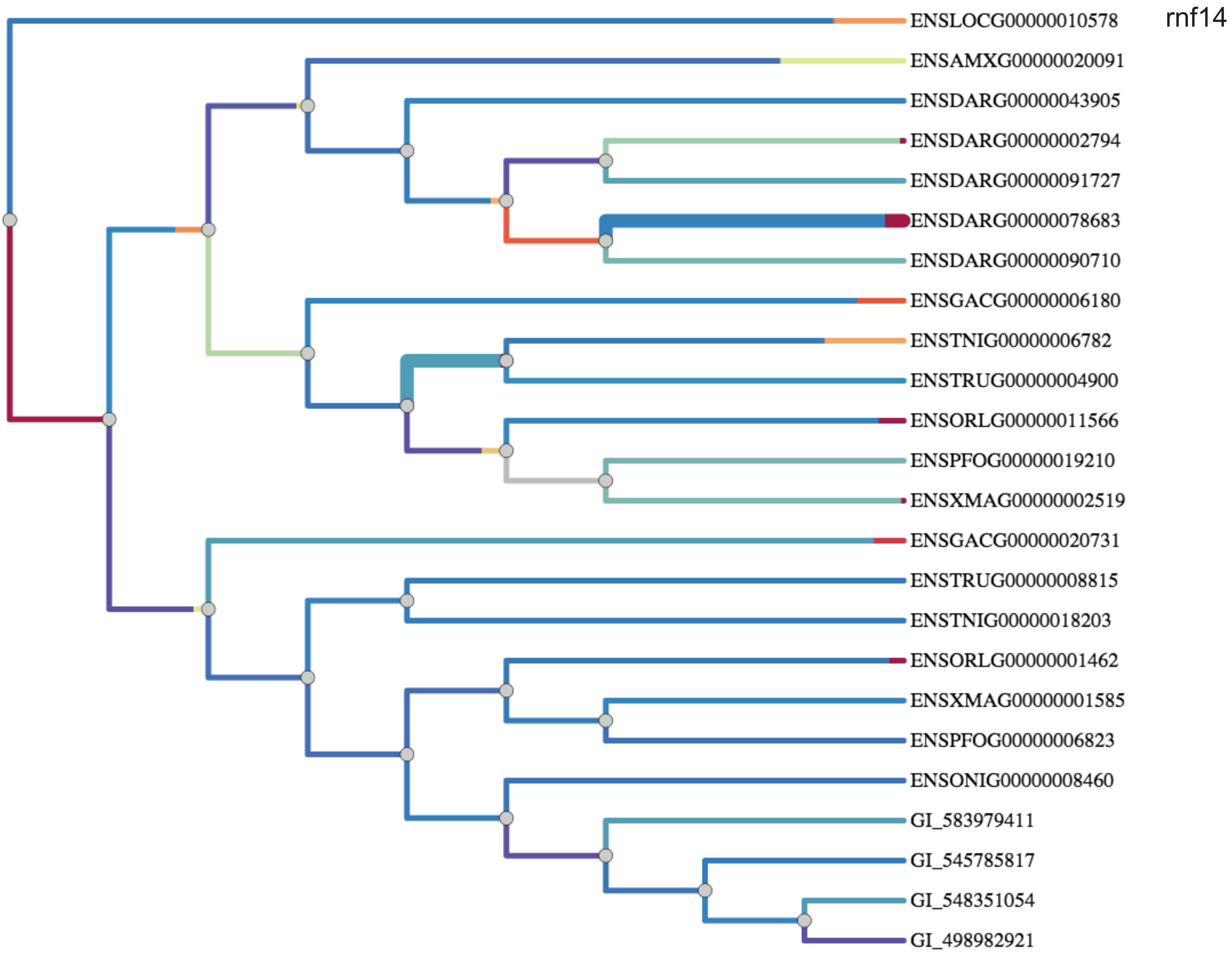

ENSLOCG00000010578
rnf14
ENSAMXG00000020091
ENSDARG00000043905
ENSDARG00000002794
ENSDARG00000091727
ENSDARG00000078683
ENSDARG00000090710
ENSGACG00000006180
ENSTNIG00000006782
ENSTRUG00000004900
ENSORLG00000011566
ENSPFOG00000019210
ENSXMAG00000002519
ENSGACG00000020731
ENSTRUG00000008815
ENSTNIG00000018203
ENSORLG00000001462
ENSXMAG00000001585
ENSPFOG00000006823
ENSONIG00000008460
GI_583979411
GI_545785817
GI_548351054
GI_498982921

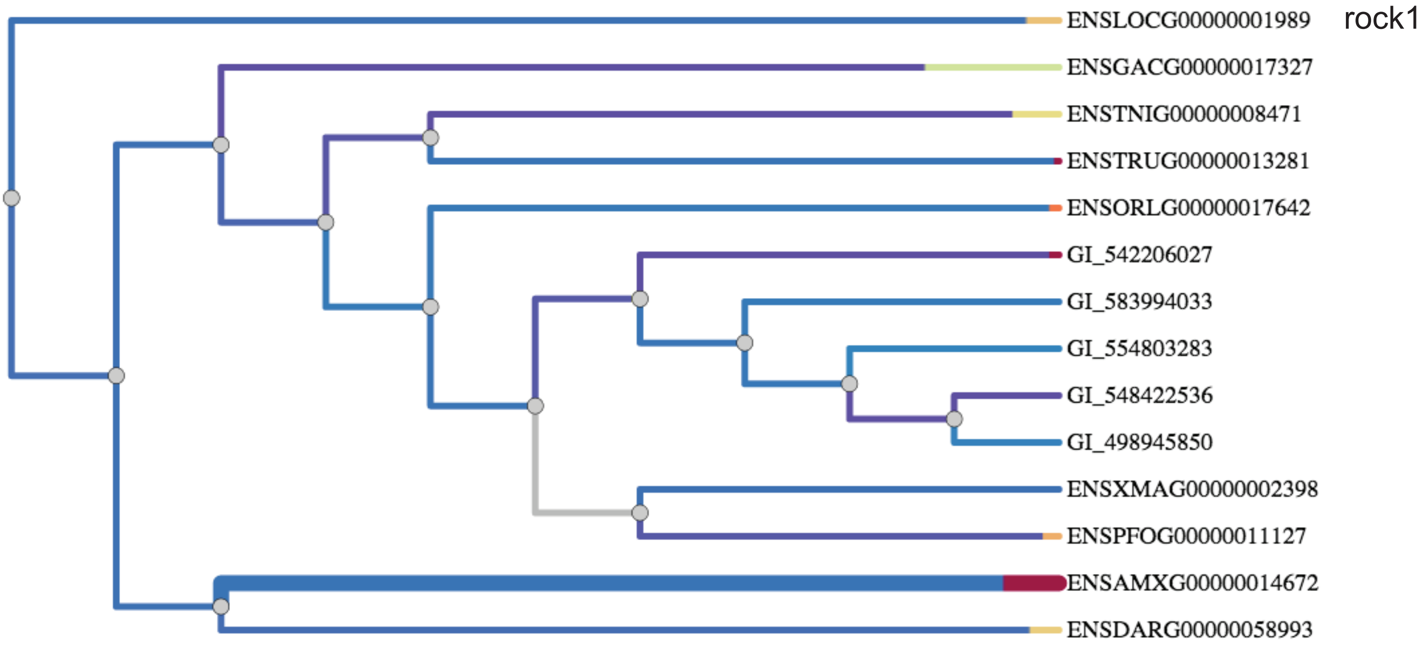

ENSLOCG00000001989
rock1
ENSGACG00000017327
ENSTNIG00000008471
ENSTRUG00000013281
ENSORLG00000017642
GI_542206027
GI_583994033
GI_554803283
GI_548422536
GI_498945850
ENSXMAG00000002398
ENSPFOG00000011127
ENSAMXG00000014672
ENSDARG00000058993

Figure S3

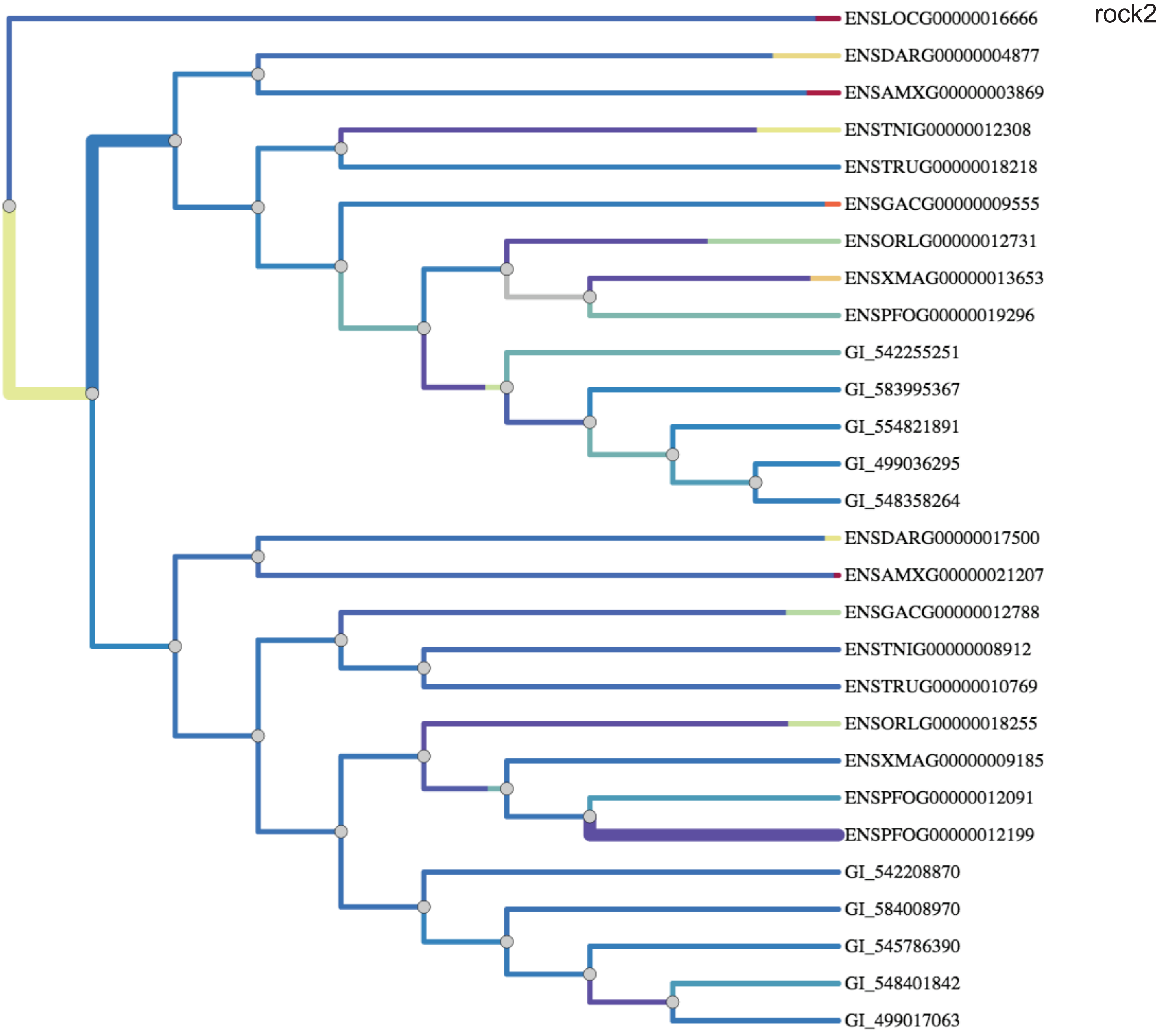
rock2
ENSLOCG00000016666
ENSDARG00000004877
ENSAMXG00000003869
ENSTNIG00000012308
ENSTRUG00000018218
ENSGACG00000009555
ENSORLG00000012731
ENSXMAG00000013653
ENSPFOG00000019296
GI_542255251
GI_583995367
GI_554821891
GI_499036295
GI_548358264
ENSDARG00000017500
ENSAMXG00000021207
ENSGACG00000012788
ENSTNIG00000008912
ENSTRUG00000010769
ENSORLG00000018255
ENSXMAG00000009185
ENSPFOG00000012091
ENSPFOG00000012199
GI_542208870
GI_584008970
GI_545786390
GI_548401842
GI_499017063

Figure S3

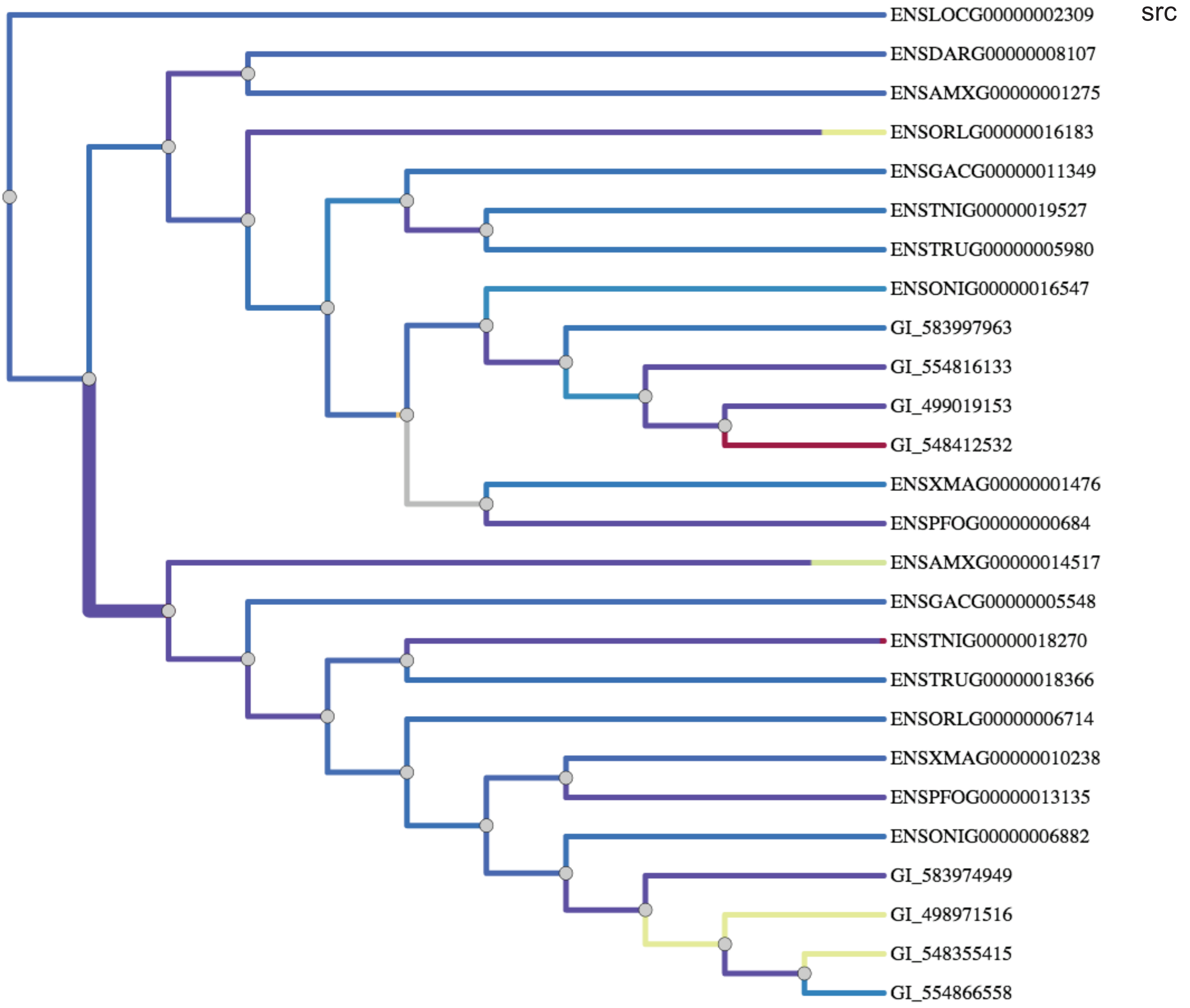
ENSLOCG00000002309
src
ENSDARG00000008107
ENSAMXG00000001275
ENSORLG00000016183
ENSGACG00000011349
ENSTNIG00000019527
ENSTRUG00000005980
ENSONIG00000016547
GI_583997963
GI_554816133
GI_499019153
GI_548412532
ENSXMAG00000001476
ENSPFOG00000000684
ENSAMXG00000014517
ENSGACG00000005548
ENSTNIG00000018270
ENSTRUG00000018366
ENSORLG00000006714
ENSXMAG00000010238
ENSPFOG00000013135
ENSONIG00000006882
GI_583974949
GI_498971516
GI_548355415
GI_554866558

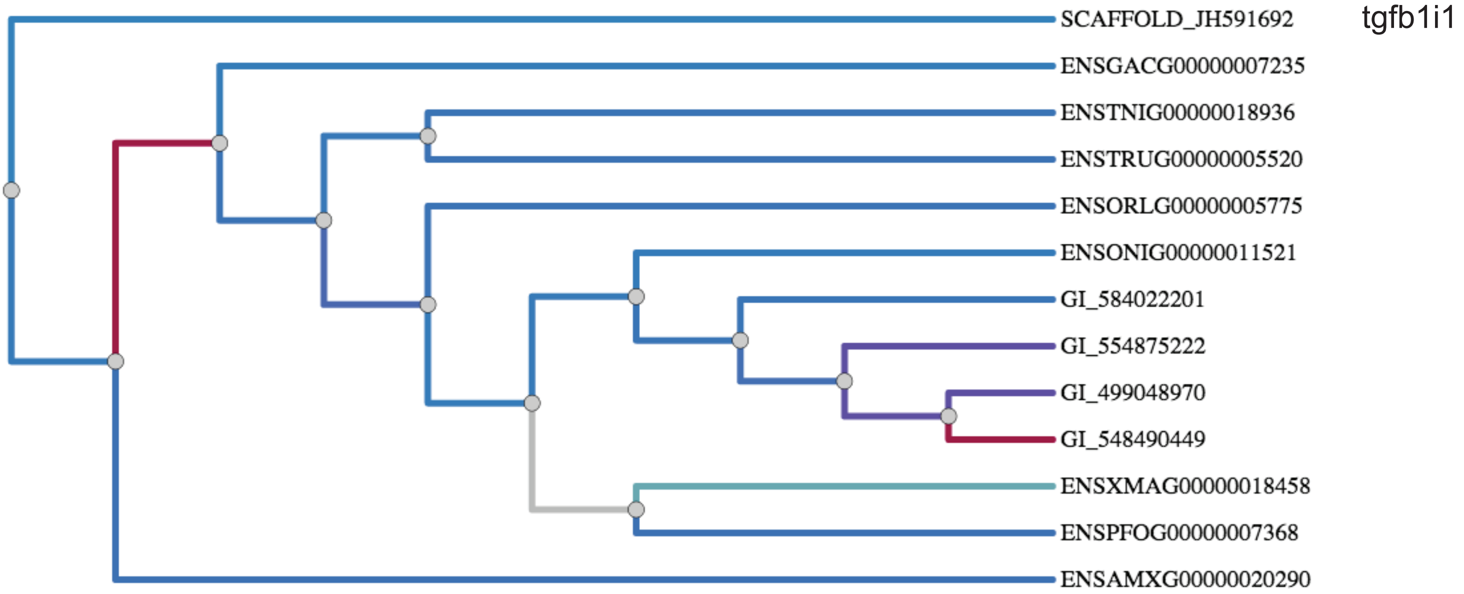
SCAFFOLD_JH591692
tgfb1i1
ENSGACG00000007235
ENSTNIG00000018936
ENSTRUG00000005520
ENSORLG00000005775
ENSONIG00000011521
GI_584022201
GI_554875222
GI_499048970
GI_548490449
ENSXMAG00000018458
ENSPFOG00000007368
ENSAMXG00000020290

Figure S3

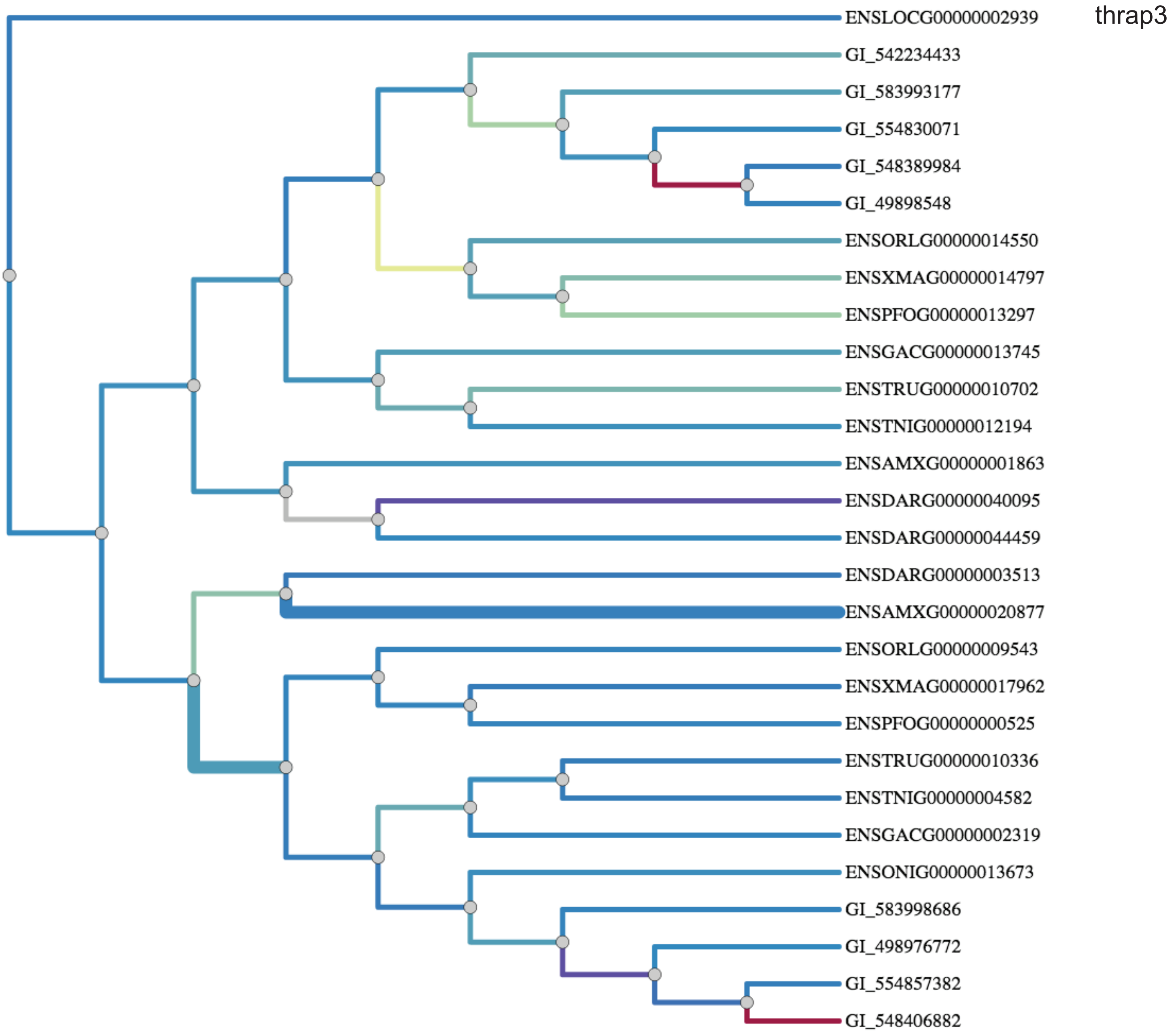
thrap3
ENSLOCG00000002939
GI_542234433
GI_583993177
GI_554830071
GI_548389984
GI_49898548
ENSORLG00000014550
ENSXMAG00000014797
ENSPFOG00000013297
ENSGACG00000013745
ENSTRUG00000010702
ENSTNIG00000012194
ENSAMXG00000001863
ENSDARG00000040095
ENSDARG00000044459
ENSDARG00000003513
ENSAMXG00000020877
ENSORLG00000009543
ENSXMAG00000017962
ENSPFOG00000000525
ENSTRUG00000010336
ENSTNIG00000004582
ENSGACG00000002319
ENSONIG00000013673
GI_583998686
GI_498976772
GI_554857382
GI_548406882

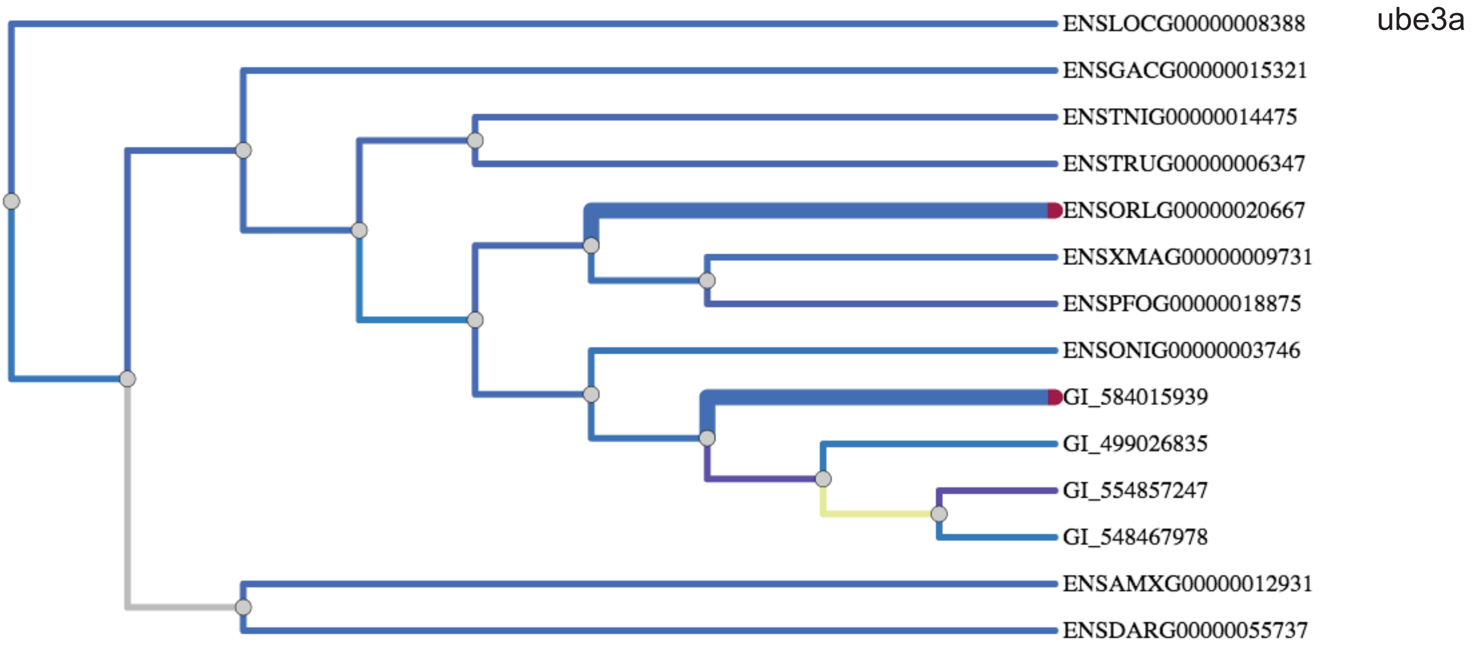
ube3a
ENSLOCG00000008388
ENSGACG00000015321
ENSTNIG00000014475
ENSTRUG00000006347
ENSORLG00000020667
ENSXMAG00000009731
ENSPFOG00000018875
ENSONIG00000003746
GI_584015939
GI_499026835
GI_554857247
GI_548467978
ENSAMXG00000012931
ENSDARG00000055737
