# Supplementary material for: Evolutionary Fate of the Androgen Receptor−Signaling Pathway in Ray-Finned Fishes with a Special Focus on Cichlids
Source: G3 (Bethesda). 2015 Sep 1;5(11):2275–83. doi: 10.1534/g3.115.020685 (PMC4632047; doi:10.1534/g3.115.020685)
Supplement: Supporting Information [file supp_g3.115.020685_TableS1.pdf]

Table S1

Ray-finned fish genes of the non-genomic and genomic Androgen Receptor signaling pathway according to NetPath, GO0030521, Bennett *et al.* 2010, Foradori *et al.* 2008

Gene	Species *L.oculatus*	Gene	Species *D.rerio*	*A.mexicanus*	*G.aculeatus*	*T.nigroviridis*	*T.rubripes*	*P.formosa*	*X.maculatus*	*O.latipes*	*O.niloticus*	*N.brichardi*	*M.zebra*	*P.nyererei*	*A.burtoni*	Source of gene	Pathway association
AKT1	ENSLOCG00000012762		NM_001281801.1	ENSAMXG00000009230<br>ENSAMXG00000012355	ENSGACG00000006298	ENSTNIG00000019495	ENSTRUG00000013476	ENSPFOG00000010125	ENSXMAG00000006673	ENSORLG00000017024	gi_542229192	gi_583981523	gi_498935125	gi_548423349	gi_554812288	NetPath, Bennett et al 2010, Foradori et al 2008	non-genomic, genomic
AR	ENSLOCG00000014680	ARA		ENSAMXG00000013256	ENSGACG00000018525	ENSTNIG00000015783	ENSTRUG00000005373	ENSPFOG00000006490	ENSXMAG00000002896	ENSORLG00000008220	ENSONIG00000017538	gi_583972812	gi_499025148	gi_545793521	gi_555943746	NetPath	receptor, genomic, non-genomic
		ARB	ENSDARG00000067976	ENSAMXG00000011395	ENSGACG00000020332	ENSTNIG00000011826	ENSTRUG00000012421	ENSPFOG00000019378	ENSXMAG00000012307	ENSORLG00000009520	ENSONIG00000012854	gi_584009291	gi_499011353	gi_548397957	gi_555943801	NetPath	receptor, genomic, non-genomic
ARID1	ENSLOCG00000003798	ARID1AA	ENSDARG00000101710	ENSAMXG00000020237	ENSGACG00000007244	ENSTNIG00000009659	ENSTRUG00000013351	ENSPFOG00000009717	ENSXMAG00000010716	ENSORLG00000004410	ENSONIG00000006764	gi_584001874	gi_499018427	gi_548435329	gi_554870576	GO:0030521	genomic
		ARID1AB	ENSDARG00000101891	ENSAMXG00000013606	ENSGACG00000007627	ENSTNIG00000004670	ENSTRUG00000012325	ENSPFOG00000002208	ENSXMAG00000012041		ENSONIG00000007618	gi_584000591	gi_499027400	gi_548538463	gi_554835101	GO:0030521	genomic
BRCA1	ENSLOCG00000011391	BRCA1		ENSAMXG00000015282	ENSGACG00000006381	ENSTNIG00000010246	ENSTRUG00000009091	ENSPFOG00000004731	ENSXMAG00000009949	ENSORLG00000004585	ENSONIG00000016326	gi_583992196	gi_499033693	gi_548541303	gi_554835497	NetPath, GO:0030521	genomic, positive regulator
CAV1	ENSLOCG00000015597	CAV1	ENSDARG00000052004	ENSAMXG00000011618	ENSGACG00000009201	ENSTNIG00000016057	ENSTRUG00000017112	ENSPFOG00000014440	ENSXMAG00000014453	ENSORLG00000019746	ENSONIG00000003896	gi_583973968	gi_499007024	gi_548521455	gi_554856484	NetPath	non-genomic
CCNE1	ENSLOCG00000005671	CCNE1	ENSDARG00000098622	ENSAMXG00000002929	ENSGACG00000012549	ENSTNIG00000006196	ENSTRUG00000000222<br>ENSTRUG00000000353<br>ENSTRUG00000001296<br>ENSTRUG00000001834<br>ENSTRUG00000001857<br>ENSTRUG00000017625<br>ENSTRUG00000000484	ENSPFOG00000011801	ENSXMAG00000001005	ENSORLG00000015511	ENSONIG00000003504	gi_584015275	gi_499030053	gi_548348207	gi_554826914	NetPath, GO:0030521	genomic, positive regulator
CDC4	ENSLOCG00000001073	CDC42A		ENSAMXG00000019820	ENSGACG00000012448	ENSTNIG00000012549	ENSTRUG00000013411	ENSPFOG00000005335	ENSXMAG00000016228	ENSORLG00000013756	ENSONIG00000016914	gi_573898313	gi_494461779	gi_545793478	gi_545786543	NetPath	non-genomic
		CDC42B	ENSDARG00000044573	ENSAMXG00000003583	ENSGACG00000010913	ENSTNIG00000007372	ENSTRUG00000000167	ENSPFOG00000004608	ENSXMAG00000008839	ENSORLG00000012048	ENSONIG00000020235	gi_584010624	gi_499018345	gi_548348712	gi_554846439	NetPath	non-genomic
CDK7	ENSLOCG00000006117	CDK7	ENSDARG00000051916	ENSAMXG00000008454	ENSGACG00000009848	ENSTNIG00000015407	ENSTRUG00000010233	ENSPFOG00000017422	ENSXMAG00000007316	ENSORLG00000008836	ENSONIG00000013709	gi_583977761	gi_498955997	gi_548339440	gi_554874678	GO:0030521	genomic
CFL1L	ENSLOCG00000015680	CFL1L	ENSDARG00000012972	ENSAMXG00000007837	ENSGACG00000004750			ENSPFOG00000017145	ENSXMAG00000006139	ENSORLG00000011865	ENSONIG00000010009	gi_584024814	no blast hit on n	gi_548490653	gi_554852342	NetPath	non-genomic
CTNNB1	ENSLOCG00000001383	CTNNB1	ENSDARG00000014571	ENSAMXG00000000198	ENSGACG00000006037	ENSTNIG00000019094	ENSTRUG00000003520	ENSPFOG00000018568	ENSXMAG00000008710	ENSORLG00000005845	ENSONIG00000007226	gi_583999804	gi_499034959	gi_548426098	gi_554880769	NetPath, GO:0030521	genomic, positive regulator
DAXX	ENSLOCG00000000498	DAXX	ENSDARG00000000729	ENSAMXG00000014180	ENSGACG00000001844	ENSTNIG00000005315<br>ENSTNIG00000018185	ENSTRUG00000003811	ENSPFOG00000001776	ENSXMAG00000007090	ENSORLG00000006837	ENSONIG00000008401	gi_573898112	gi_499044186	gi_548490874	gi_554886315	NetPath, GO:0030521	genomic, negative regulator
DNAJA1	ENSLOCG00000011564	DNAJA1	ENSDARG00000030972	ENSAMXG00000010757	ENSGACG00000017879			ENSPFOG00000004920<br>ENSPFOG00000005099	ENSXMAG00000005855	ENSORLG00000006260	ENSONIG00000014539	gi_583983886	gi_498979425	gi_548348922	gi_554873916	GO:0030521	genomic
EGFR	ENSLOCG00000011537	EGFRA	gi_35903182	ENSAMXG00000012474	ENSGACG00000017289	ENSTNIG00000014840	ENSTRUG00000011834	ENSPFOG00000008848	ENSXMAG00000016482	ENSORLG00000017692	ENSONIG00000009358	gi_583994068	gi_498945991	gi_548422483	gi_554803235	NetPath	non-genomic
		EGFRB		ENSAMXG00000015959	ENSGACG00000018079	ENSTNIG00000000023<br>ENSTNIG00000013552<br>ENSTNIG00000013553	ENSTRUG00000017446	ENSPFOG00000005185	ENSXMAG00000008804	ENSORLG00000003577	ENSONIG00000017327	gi_584029670	gi_499050178	gi_548511931	gi_545785452	NetPath	non-genomic
FHL2	ENSLOCG00000008763	FHL2A	ENSDARG00000003991	ENSAMXG00000005118	ENSGACG00000015048	ENSTNIG00000017175	ENSTRUG00000008468	ENSPFOG00000003291<br>ENSPFOG00000022124	ENSXMAG00000002166	ENSORLG00000001848	ENSONIG00000012307	gi_583995557	gi_499025292	gi_548401457	gi_554818515	NetPath, GO:0030521	genomic, positive regulator
		FHL2B	ENSDARG00000042018	ENSAMXG00000009663	ENSGACG00000003005	ENSTNIG00000000462	ENSTRUG00000013559	ENSPFOG00000015302	ENSXMAG00000011596	ENSORLG00000012482	ENSONIG00000014220	gi_583986192	gi_498986713	gi_548379261	gi_554870366	NetPath, GO:0030521	genomic, positive regulator
FKBP4	ENSLOCG00000016880	FKBP4	ENSDARG00000008447	ENSAMXG00000003846	ENSGACG00000012979	NA	ENSTRUG00000015044	ENSPFOG00000018881	ENSXMAG00000003963	ENSORLG00000008977	ENSONIG00000015051	gi_583988484	gi_498994696	gi_548356125	gi_545786431	GO:0030521	genomic, negative regulator
FLNA	ENSLOCG00000015505	FLNA	ENSDARG00000074201	ENSAMXG00000013411	ENSGACG00000013098	ENSTNIG00000004951	ENSTRUG00000003170	ENSPFOG00000001616	ENSXMAG00000004665	ENSORLG00000001982	ENSONIG00000017914	gi_584019220	gi_498964459	gi_548536083	gi_554815264	NetPath	non-genomic
GNB2I1	ENSLOCG00000009758	GNB2I1	ENSDARG00000041619	ENSAMXG00000007457<br>ENSAMXG00000002584	ENSGACG00000018103	ENSTNIG00000010510	ENSTRUG00000000550	ENSPFOG00000008165	ENSXMAG00000008940	ENSORLG00000005276	ENSONIG00000012984	gi_583972285	gi_498935526	gi_548334125	gi_554821010	NetPath	genomic
GRIP1	ENSLOCG00000016167	GRIP1	ENSDARG00000015053	ENSAMXG00000011215	ENSGACG00000000177	ENSTNIG00000002863	ENSTRUG00000005225	ENSPFOG00000012379	ENSXMAG00000006468	ENSORLG00000017453	gi_542204218	gi_583971256	gi_499031622	gi_548419148	gi_554820840	GO:0030521	genomic
KAT5	NA	KAT5	ENSDARG00000004587<br>ENSDARG00000045951	ENSAMXG00000009091	ENSGACG00000020408	ENSTNIG00000011743	ENSTRUG00000016643	ENSPFOG00000010293	ENSXMAG00000005173	ENSORLG00000007458	ENSONIG00000005359	gi_584015024	gi_499026510	gi_548385825	gi_554823092	NetPath, GO:0030521	genomic, positive regulator
KDM3A	ENSLOCG00000010817	KDM3A	NA	NA	NA	NA	NA	NA	NA	NA	NA	NA	NA	NA	NA	GO:0030521	genomic
LIMK2	ENSLOCG00000004821	LIMK2	ENSDARG00000005104	gi_597735797	ENSGACG00000008716	ENSTNIG00000015862	ENSTRUG00000013732	ENSPFOG00000009526	ENSXMAG00000018076	ENSORLG00000006835	ENSONIG00000013399	gi_573905058	gi_498956642	gi_548339212	gi_554811068	NetPath	non-genomic
MAPK1	ENSLOCG00000002024	MAPK1	ENSDARG00000027552	ENSAMXG00000001222	ENSGACG00000014421	ENSTNIG00000015351	ENSTRUG00000015300	ENSPFOG00000006212	ENSXMAG00000006444	scaffold5487_contig1298[illegible]	ENSONIG00000014115	gi_584005676	gi_499004332	gi_548356923	gi_554822617	Bennett et al 2010	non-genomic, genomic
MAPK3	NA	MAPK3	ENSDARG00000070573	ENSAMXG00000017822	ENSGACG00000013102	ENSTNIG00000011583	ENSTRUG00000005697	ENSPFOG00000008154	ENSXMAG00000011987	ENSORLG00000011993	ENSONIG00000019408	gi_584026649	gi_499019863	gi_548501870	gi_554861523	Bennett et al 2010	non-genomic, genomic
MED1	ENSLOCG00000012767	MED1	ENSDARG00000075340	ENSAMXG00000015432	ENSGACG00000006033	ENSTNIG00000004334	ENSTRUG00000012065	ENSPFOG00000001519	ENSXMAG00000002969	ENSORLG00000008300	gi_542192331	gi_583992145	gi_499021221	gi_548391342	gi_554817925	GO:0030521	genomic
MED4	ENSLOCG00000000694	MED4	ENSDARG00000041503	ENSAMXG00000012890	ENSGACG00000008017	ENSTNIG00000003778	ENSTRUG00000014013	ENSPFOG00000010829	ENSXMAG00000008718	ENSORLG00000014076	ENSONIG00000016177	gi_584023652	gi_499031293	gi_548532252	gi_554866935	GO:0030521	genomic
MED12	ENSLOCG00000015382	MED12	ENSDARG00000056800	ENSAMXG00000011603	ENSGACG00000017493	ENSTNIG00000015746	ENSTRUG00000012646	ENSPFOG00000000954	ENSXMAG00000000274	ENSORLG00000000784	ENSONIG00000002455	gi_584017139	gi_498964171	gi_548340722	gi_554804436	GO:0030521	genomic
MED13	ENSLOCG00000003727	MED13A	ENSDARG00000053884	ENSAMXG00000000953	ENSGACG00000020213	ENSTNIG00000009033	ENSTRUG00000008851	ENSPFOG00000024209	ENSXMAG00000000108	ENSORLG00000012337	ENSONIG00000017544	gi_573902086	gi_499021858	gi_548408649	gi_554832637	GO:0030521	genomic
		MED13B	ENSDARG00000003910	ENSAMXG00000002817	ENSGACG00000010389	ENSTNIG00000003289	ENSTRUG00000007405	ENSPFOG00000009647	ENSXMAG00000018016	ENSORLG00000005489	ENSONIG00000011173	gi_573890035	gi_499014114	gi_548404206	gi_554855577	GO:0030521	genomic
MED14	ENSLOCG00000002286	MED14	ENSDARG00000009953	ENSAMXG00000002581	ENSGACG00000002015	ENSTNIG00000014969	ENSTRUG00000013609	ENSPFOG00000013987	ENSXMAG00000018007	ENSORLG00000010808	ENSONIG00000006848	gi_583985895	gi_499034639	gi_548378507	gi_554851281	GO:0030521	genomic
MED16	ENSLOCG00000001808	MED16	ENSDARG00000040779	ENSAMXG00000018838	ENSGACG00000013385	ENSTNIG00000004736	ENSTRUG00000007850	ENSPFOG00000008857	ENSXMAG00000010740	ENSORLG00000000109	ENSONIG00000004564	gi_583990837	gi_499010815	gi_548370249	gi_554824751	GO:0030521	genomic
MED17	ENSLOCG00000007406	MED17	ENSDARG00000006345	ENSAMXG00000016638	ENSGACG00000013203	ENSTNIG00000006440	ENSTRUG00000004276	ENSPFOG00000017880	ENSXMAG00000007752	ENSORLG00000000709	ENSONIG00000001372	gi_584012931	gi_499047177	gi_548396051	gi_554887944	GO:0030521	genomic
MED24	ENSLOCG00000012864	MED24	ENSDARG00000032459	ENSAMXG00000012180	ENSGACG00000008529	ENSTNIG00000012846	ENSTRUG00000016382	ENSPFOG00000016585	ENSXMAG00000011374	ENSORLG00000008227	ENSONIG00000019926	gi_584012330	gi_499023404	gi_548338012	gi_554858967	GO:0030521	genomic
MED30	ENSLOCG00000003235	MED30	ENSDARG00000029459	ENSAMXG00000021203	ENSGACG00000003014	ENSTNIG00000000448	ENSTRUG00000003806	ENSPFOG00000000737	ENSXMAG00000014029	ENSORLG00000001732	ENSONIG00000013534	gi_583998619	gi_498976995	gi_548477220	gi_554852211	GO:0030521	genomic
NCOA1	ENSLOCG00000015891	NCOA1	ENSDARG00000018257	ENSAMXG00000010390	ENSGACG00000006727	ENSTNIG00000019142	ENSTRUG00000006619	ENSPFOG00000004231	ENSXMAG00000017782	scaffold1294_contig11304	ENSONIG00000015724	gi_584022154	gi_498984261	gi_548530893	gi_554840198	NetPath, GO:0030521	genomic, positive regulator
NCOA3	ENSLOCG00000003989	NCOA3A		ENSAMXG00000008788<br>ENSAMXG00000008791<br>ENSAMXG00000008794	ENSGACG00000015221	ENSTNIG00000012601	ENSTRUG00000004450	ENSPFOG00000011587	ENSXMAG00000017470	ENSORLG00000012143	ENSONIG00000017075	gi_584003095	gi_498965809	gi_548366017	gi_554831335	NetPath, GO:0030521	genomic, positive regulator
		NCOA3B	ENSDARG00000077404	ENSAMXG00000006902	ENSGACG00000010036	ENSTNIG00000007303	ENSTRUG00000009516	ENSPFOG00000006902	ENSXMAG00000001970	ENSORLG00000013704	ENSONIG00000020313	gi_583978807	gi_499042642	gi_548447463	gi_554808986	NetPath, GO:0030521	genomic, positive regulator
NCOA4	ENSLOCG00000012898	NCOA4	ENSDARG00000021439	ENSAMXG00000015735	ENSGACG00000012317	ENSTNIG00000013384	ENSTRUG00000003924	ENSPFOG00000005630	ENSXMAG00000002456	ENSORLG00000003269	ENSONIG00000007129	gi_584024662	gi_499046546	gi_548457815	gi_554883384	NetPath, GO:0030521	genomic, positive regulator
NKX3.1	ENSLOCG00000015015	NKX3.1	ENSDARG00000078280	ENSAMXG00000024837	ENSGACG00000010776	SCAF14652	NA	ENSPFOG00000009654	ENSXMAG00000020262	ENSORLG00000010925	ENSONIG00000011762	gi_583977526	gi_498955057	gi_548354440	gi_554824085	GO:0030521	genomic
NR1I3	NA	NR1I3	NA	NA	NA	NA	NA	NA	NA	NA	NA					GO:0030521	genomic
NRIP1	ENSLOCG00000000906	NRIP1A	ENSDARG00000068965	ENSAMXG00000025365												GO:0030521	genomic
		NRIP1B	ENSDARG00000068894	ENSAMXG00000025737	ENSGACG00000020154		ENSTRUG00000004236<br>ENSTRUG00000000031	ENSPFOG00000017715	ENSXMAG00000014033	ENSORLG00000000586	ENSONIG00000015866	gi_584025409	gi_499021768	gi_548408144	gi_554851579	GO:0030521	genomic
PIAS1	ENSLOCG00000013933	PIAS1A	ENSDARG00000036094	ENSAMXG00000006151	ENSGACG00000016894	ENSTNIG00000003928<br>ENSTNIG00000009549	ENSTRUG00000016843	ENSPFOG00000006900	ENSXMAG00000017552	ENSORLG00000002077	ENSONIG00000013251	gi_583997712	gi_499013515	gi_548448896	gi_554840096	NetPath, GO:0030521	genomic, negative regulator
		PIAS1B	ENSDARG00000062445	ENSAMXG00000002784	ENSGACG00000005176	ENSTNIG00000008434	ENSTRUG00000015008	ENSPFOG00000009110	ENSXMAG00000003583	ENSORLG00000006728	ENSONIG00000015473	gi_583988189	gi_498993905	gi_548355942	gi_554863406	NetPath, GO:0030521	genomic, negative regulator
PIAS2	ENSLOCG00000006647	PIAS2	ENSDARG00000092979	ENSAMXG00000002435	ENSGACG00000005099	ENSTNIG00000003200	ENSTRUG00000013569	ENSPFOG00000000261	scaffold_JH558015.1	ENSORLG00000017353	ENSONIG00000013927	gi_584015665	gi_499015732	gi_548377776	gi_554864979	NetPath, GO:0030521	genomic, negative regulator
PIK3R1	ENSLOCG00000010510	PIK3R1A	ENSDARG00000038524	ENSAMXG00000011959	ENSGACG00000015820	ENSTNIG00000013464	ENSTRUG00000007584	ENSPFOG00000001009	ENSXMAG00000018595	ENSORLG00000002670	ENSONIG00000011065	gi_584003878	gi_498981102	gi_548334788	gi_554838849	NetPath, Bennett et al 2010, Foradori et al 2008	non-genomic
		PIK3R1B			ENSGACG00000001062	ENSTNIG00000018878	ENSTRUG00000010930	ENSPFOG00000015835	ENSXMAG00000015104	ENSORLG00000017457	ENSONIG00000001749	gi_573878953	gi_499049716	gi_548541918	gi_554876860	NetPath, Bennett et al 2010, Foradori et al 2008	non-genomic
PIK3R2	ENSLOCG00000001519	PIK3R2A	ENSDARG00000018060	XM_007236673.1	ENSGACG00000008201	ENSTNIG00000010197	ENSTRUG00000007054	ENSPFOG00000000170<br>ENSPFOG00000023056<br>ENSPFOG00000024225	ENSXMAG00000002484	LOC101163728	ENSONIG00000012700	gi_583993919	gi_499028291	gi_548356312	gi_554849340	NetPath, Bennett et al 2010, Foradori et al 2008	non-genomic
		PIK3R2B	NA	NA	ENSGACG00000015520	ENSTNIG00000011070	ENSTRUG00000010476	ENSPFOG00000003942	ENSXMAG00000013889	NA	ENSONIG00000010477	gi_583983019	gi_498942969	gi_548336831	gi_554819907	NetPath, Bennett et al 2010, Foradori et al 2008	non-genomic
PMEPA1	NA	PMEPA1	ENSDARG00000039325	ENSAMXG00000013100	ENSGACG00000003728	NA	NA	ENSPFOG00000001516	ENSXMAG00000010456	ENSORLG00000012800	ENSONIG00000004438	gi_573906092	gi_494461325	gi_545793510	gi_545786152	GO:0030521	genomic
PPAP2A	NA	PPAP2A	ENSDARG00000053381	ENSAMXG00000018732	NA	NA	NA	ENSPFOG00000003517	ENSXMAG00000004780	ENSORLG00000005464	ENSONIG00000007599	gi_583987884	gi_498980257	gi_548335165	gi_554813089	GO:0030521	genomic
PPARGC1A	ENSLOCG00000012350	PPARGC1A	ENSDARG00000067829	ENSAMXG00000002461	ENSGACG00000019546	ENSTNIG00000002174	ENSTRUG00000007210	ENSPFOG00000001263	ENSXMAG00000007748	ENSORLG00000004788	ENSONIG00000007635	gi_584018961	gi_498999424	gi_548425231	gi_554850044	GO:0030521	genomic
PTEN	ENSLOCG00000006145	PTENA	ENSDARG00000071018	ENSAMXG00000010994	ENSGACG00000009998	ENSTNIG00000005984	NA	ENSPFOG00000004955	ENSXMAG00000014454	ENSORLG00000020711	ENSONIG00000019123	gi_583996646	gi_499029140	gi_548428164	gi_554877417	NetPath	non-genomic
		PTENB	ENSDARG00000056623	ENSAMXG00000007753	ENSGACG00000009236	ENSTNIG00000013318	ENSTRUG00000009851	ENSPFOG00000015747	ENSXMAG00000012957	ENSORLG00000006231	ENSONIG00000000386	gi_583976942	gi_498987823	gi_548350440	gi_554806495	NetPath	non-genomic
PTK2A	ENSLOCG00000007702	PTK2AA	ENSDARG00000040197	ENSAMXG00000018637	ENSGACG00000004011<br>ENSGACG00000004003	ENSTNIG00000009167<br>ENSTNIG00000009166	ENSTRUG00000007362	ENSPFOG00000003397	ENSXMAG00000015476	scaffold5001_contig12887	ENSONIG00000002815	gi_583985834	gi_498978332	gi_548352657	gi_554813630	NetPath	non-genomic
		PTK2AB	ENSDARG00000004672	ENSAMXG00000004491	ENSGACG00000007004	ENSTNIG00000009636	ENSTRUG00000011167	ENSPFOG00000010090	ENSXMAG00000010911	ENSORLG00000004751	ENSONIG00000006838	gi_573896657	gi_499029030	gi_548435043	gi_554849438	NetPath	non-genomic
PTK2B	ENSLOCG00000017241	PTK2BB	ENSDARG00000039577	ENSAMXG00000019303	ENSGACG00000010609	ENSTNIG00000016347	ENSTRUG00000008406	ENSPFOG00000008438	ENSXMAG00000004048	ENSORLG00000008993	ENSONIG00000015297	gi_584004629	gi_498939798	gi_548381009	gi_554807097	NetPath	non-genomic
		PTK2BA	ENSDARG00000022841	ENSAMXG00000000798	ENSGACG00000014838	ENSTNIG00000014508	ENSTRUG00000017337	ENSPFOG00000009145	ENSXMAG00000004406	ENSORLG00000005884	ENSONIG00000015175	gi_584025201	gi_499049776	gi_545793246	gi_554877844	NetPath	non-genomic
RAC1	ENSLOCG00000008397	RAC1AA	ENSDARG00000074849<br>ENSDARG00000099506<br>ENSDARG00000087954<br>ENSDARG00000013203<br>ENSDARG00000096666<br>ENSDARG00000096549<br>ENSDARG00000054649	ENSAMXG00000008935	ENSGACG00000011601	ENSTNIG00000017983	ENSTRUG00000016201	ENSPFOG00000004946	ENSXMAG00000001114	ENSORLG00000008757<br>ENSORLG00000008763	ENSONIG00000017149	gi_583992724	gi_499014994	gi_548382840	gi_554829712	NetPath	non-genomic
		RAC1AB								ENSORLG00000008757<br>ENSORLG00000008763	ENSONIG00000017151	gi_583992732	gi_499014998	gi_548382828	gi_554829718	NetPath	non-genomic
		RAC1B			ENSGACG00000009531	ENSTNIG00000012875	ENSTRUG00000016765	ENSPFOG00000015316<br>ENSPFOG00000015319<br>ENSPFOG00000015350	ENSXMAG00000011073<br>ENSXMAG00000011081<br>ENSXMAG00000011307<br>ENSXMAG00000011324	ENSORLG00000003527	ENSONIG00000019847	gi_584012219	gi_499023276	gi_548338137	gi_554845115	NetPath	non-genomic

RAF1	ENSLOCG00000013974	RAF1	ENSDARG00000059406<br>ENSDARG00000096415	ENSAMXG00000005440<br>ENSAMXG00000001055	ENSGACG00000000060	ENSTNIG00000012621	ENSTRUG00000006611	ENSPFOG00000015974	ENSXMAG00000017691	ENSORLG00000011471	ENSONIG00000017170	gi_584003028	gi_498965606	gi_548483383	gi_554857802	Foradori et al 2008	non-genomic
RAN	ENSLOCG00000008279	RAN	ENSDARG00000057026	ENSAMXG00000015390	ENSGACG00000017755	ENSTNIG00000013137	ENSTRUG00000013749	ENSPFOG00000009552	ENSXMAG00000009922	ENSORLG00000003452	ENSONIG00000005139	gi_583972026	gi_498962091	gi_548341336	gi_554805068	NetPath, GO:0030521	genomic, positive regulator
RB1	ENSLOCG00000008882	RB1	ENSDARG00000006782	ENSAMXG00000005363	ENSGACG00000020379	ENSTNIG00000011786	ENSTRUG00000015842	ENSPFOG00000003889	ENSXMAG00000015729	ENSORLG00000008317	ENSONIG00000004276	gi_584014918	gi_499026357	gi_548387323	gi_554822936	NetPath, GO:0030521	genomic, positive regulator
RHOAA	ENSLOCG00000014301	RHOAAA	ENSDARG00000026845	ENSAMXG00000014632				ENSPFOG00000017531	ENSXMAG00000004466		ENSONIG00000017974	gi_584019304	gi_498964222	gi_548443896	gi_554866988	NetPath	non-genomic
		RHOAAB	ENSDARG00000015429		ENSGACG00000009938	ENSTNIG00000015015	ENSTRUG00000010405				ENSONIG00000012367	gi_583980905	gi_499016231	gi_548370620	gi_554807569	NetPath	non-genomic
RHOAB	ENSLOCG00000014438	RHOABA	ENSDARG00000094673	ENSAMXG00000005847	ENSGACG00000005670	ENSTNIG00000006051	ENSTRUG00000010942	ENSPFOG00000000160	ENSXMAG00000019368	ENSORLG00000002742	ENSONIG00000001670	gi_583980256	gi_498997689	gi_548357349	gi_554857836	NetPath	non-genomic
		RHOABB		ENSAMXG00000008373	ENSGACG00000001206	ENSTNIG00000014672	ENSTRUG00000011944	ENSPFOG00000019065	ENSXMAG00000016892	ENSORLG00000018734	ENSONIG00000018884	gi_583976140	gi_498957597	gi_548414713	gi_554862978	NetPath	non-genomic
RHOB	ENSLOCG00000017876	RHOB		ENSAMXG00000026330	ENSGACG00000009667	ENSTNIG00000002278	ENSTRUG00000018178	ENSPFOG00000020611	ENSXMAG00000019632	ENSORLG00000012619	ENSONIG00000020448	gi_583994917	gi_499036281	gi_548358324	gi_554821869	NetPath	non-genomic
RNF4	ENSLOCG00000006957	RNF4	ENSDARG00000090993	ENSAMXG00000007606	ENSGACG00000016217	SCAF7445	ENSTRUG00000017344	ENSPFOG00000017123	ENSXMAG00000015165	ENSORLG00000020573	gi_542256685	gi_583973152	gi_499025880	gi_548333174	gi_554877732	NetPath, GO:0030521	genomic, positive regulator
RNF14	ENSLOCG00000010578	RNF14A	ENSDARG00000002794	ENSAMXG00000020091	ENSGACG00000020731	ENSTNIG00000018203	ENSTRUG00000008815	ENSPFOG00000006823	ENSXMAG00000001585	ENSORLG00000001462	ENSONIG00000008460	gi_583979411	gi_498982921	gi_548351054	gi_545785817	GO:0030521	genomic
		RNF14B	ENSDARG00000043905<br>ENSDARG00000078683<br>ENSDARG00000090710<br>ENSDARG00000091727		ENSGACG00000006180	ENSTNIG00000006782	ENSTRUG00000004900	ENSPFOG00000019210	ENSXMAG00000002519	ENSORLG00000011566						GO:0030521	genomic
ROCK1	ENSLOCG00000001989	ROCK1	ENSDARG00000058993	ENSAMXG00000014672	ENSGACG00000017327	ENSTNIG00000008471	ENSTRUG00000013281	ENSPFOG00000011127	ENSXMAG00000002398	ENSORLG00000017642	gi_542206027	gi_583994033	gi_498945850	gi_548422536	gi_554803283	NetPath	non-genomic
ROCK2	ENSLOCG00000016666	ROCK2A	ENSDARG00000017500	ENSAMXG00000021207	ENSGACG00000012788	ENSTNIG00000008912	ENSTRUG00000010769	ENSPFOG00000012091<br>ENSPFOG00000012199	ENSXMAG00000009185	ENSORLG00000018255	gi_542208870	gi_584008970	gi_499017063	gi_548401842	gi_545786390	NetPath	non-genomic
		ROCK2B	ENSDARG00000004877	ENSAMXG00000003869	ENSGACG00000009555	ENSTNIG00000012308	ENSTRUG00000018218	ENSPFOG00000019296	ENSXMAG00000013653	ENSORLG00000012731	gi_542255251	gi_583995367	gi_499036295	gi_548358264	gi_554821891	NetPath	non-genomic
SCGB2A1	NA	SCGB2A1	NA	NA	NA	NA	NA	NA	NA	NA	NA	NA	NA	NA	NA	GO:0030521	genomic
SRC	ENSLOCG00000002309	SRCA		ENSAMXG00000014517	ENSGACG00000005548	ENSTNIG00000018270	ENSTRUG00000018366	ENSPFOG00000013135	ENSXMAG00000010238	ENSORLG00000006714	ENSONIG00000006882	gi_583974949	gi_498971516	gi_548355415	gi_554866558	NetPath, Bennett et al 2010, Foradori et al 2008	non-genomic
		SRCB	ENSDARG00000008107	ENSAMXG00000001275	ENSGACG00000011349	ENSTNIG00000019527	ENSTRUG00000005980	ENSPFOG00000000684	ENSXMAG00000001476	ENSORLG00000016183	ENSONIG00000016547	gi_583997963	gi_499019153	gi_548412532	gi_554816133	NetPath, Bennett et al 2010, Foradori et al 2008	non-genomic
TGFB1I1	Scaffold_JH591692.1	TGFB1I1		ENSAMXG00000020290	ENSGACG00000007235	ENSTNIG00000018936	ENSTRUG00000005520	ENSPFOG00000007368	ENSXMAG00000018458	ENSORLG00000005775	ENSONIG00000011521	gi_584022201	gi_499048970	gi_548490449	gi_554875222	NetPath, GO:0030521	genomic, positive regulator
THRAP3	ENSLOCG00000002939	THRAP3A	ENSDARG00000040095<br>ENSDARG00000044459	ENSAMXG00000001863	ENSGACG00000013745	ENSTNIG00000012194	ENSTRUG00000010702	ENSPFOG00000013297	ENSXMAG00000014797	ENSORLG00000014550	gi_542234433	gi_583993177	gi_498985480	gi_548389984	gi_554830071	GO:0030521	genomic
		THRAP3B	ENSDARG00000003513	ENSAMXG00000020877	ENSGACG00000002319	ENSTNIG00000004582	ENSTRUG00000010336	ENSPFOG00000000525	ENSXMAG00000017962	ENSORLG00000009543	ENSONIG00000013673	gi_583998686	gi_498976772	gi_548406882	gi_554857382	GO:0030521	genomic
UBE3A	ENSLOCG00000008388	UBE3A	ENSDARG00000055737	ENSAMXG00000012931	ENSGACG00000015321	ENSTNIG00000014475	ENSTRUG00000006347	ENSPFOG00000018875	ENSXMAG00000009731	ENSORLG00000020667	ENSONIG00000003746	gi_584015939	gi_499026835	gi_548467978	gi_554857247	NetPath, GO:0030521	genomic, positive regulator
