# Supplementary material for: Evolutionary Fate of the Androgen Receptor−Signaling Pathway in Ray-Finned Fishes with a Special Focus on Cichlids
Source: G3 (Bethesda). 2015 Sep 1;5(11):2275–83. doi: 10.1534/g3.115.020685 (PMC4632047; doi:10.1534/g3.115.020685)
Supplement: Supporting Information [file supp_g3.115.020685_TableS2.pdf]

Table S2
Results of jmodeltest on each coding sequence alignment indicating the best fitting nucelotide substitution model per gene

Tree	Best model according to AICc
akt1	GTR+G
ar	GTR+G+I
arid1a	GTR+G+I
brca1	GTR+G+I
cav1	HKY+I
ccne1	GTR+G+I
cdc42	SYM+G+I
cdk7	GTR+G+I
cfl1l	GTR+G
ctnnb1	GTR+G+I
daxx	GTR+G+I
dnaja1	GTR+G+I
egfr	GTR+G+I
fhl2	GTR+G
fkbp4	GTR+G+I
flna	GTR+G+I
gnb2l1	GTR+G+I
grip1	GTR+G
kat5	GTR+G+I
limk2	GTR+G+I
mapk1	GTR+G+I
mapk3	GTR+G
med1	GTR+G+I
med4	GTR+G
med12	GTR+G+I
med13	GTR+G+I
med14	GTR+G+I
med16	GTR+G+I
med17	GTR+G+I
med24	GTR+G+I
med30	HKY+G
ncoa1	HKY+G
ncoa3	GTR+G+I
ncoa4	GTR+G+I
nkx3-1	HKY+G+I
nrip1	GTR+G+I
pias1	HKY+G+I
pias2	GTR+G+I
pik3r1	GTR+G+I
pik3r2	GTR+G+I
pmepa1	HKY+G+I
ppap2a	GTR+G
ppargc1a	GTR+G
pten	GTR+G+I
ptk2a	GTR+G
ptk2b	GTR+G+I
rac1	SYM+G
raf1	GTR+G+I
ran	SYM+G+I
rb1	HKY+G
rhoaa	GTR+G+I
rhoab	GTR+G+I
rhob	HKY+G
rnf14	GTR+G+I
rnf4	GTR+G+I
rock1	GTR+G+I
rock2	GTR+G+I
src	GTR+G+I
tgfb1i1	GTR+G
thrap3	GTR+G+I
ube3a	GTR+G+I
