# Supplementary material for: Evolutionary Fate of the Androgen Receptor−Signaling Pathway in Ray-Finned Fishes with a Special Focus on Cichlids
Source: G3 (Bethesda). 2015 Sep 1;5(11):2275–83. doi: 10.1534/g3.115.020685 (PMC4632047; doi:10.1534/g3.115.020685)
Supplement: Supporting Information [file supp_g3.115.020685_TableS3.pdf]

Table S3

p-values of two-sided Welch's t-test of Ka/Ks comparisons Lake cichlids *vs.* Nile tilapia for TSGD gene copy A *vs.* gene copy B as shown in Figure 3

Gene	p-value
ar	1.26E-06
arid1a	0.3198596
cdc42	NA
egfr	0.1058882
fhl2	0.0004413
med13	0.0017968
ncoa3	0.0335987
pias1	0.0003377
pik3r1	0.0240627
pik3r2	0.0063452
pten	0.0122406
ptk2a	0.3775205
ptk2b	7.81E-05
rac1a	7.16E-06
rhoaa	0.0052176
rhoab	NA
rock2	0.0003164
src	0.0040781
thrap3	0.0004625
