# Supplementary material for: Evolutionary Fate of the Androgen Receptor−Signaling Pathway in Ray-Finned Fishes with a Special Focus on Cichlids
Source: G3 (Bethesda). 2015 Sep 1;5(11):2275–83. doi: 10.1534/g3.115.020685 (PMC4632047; doi:10.1534/g3.115.020685)
Supplement: Supporting Information [file supp_g3.115.020685_TableS4.pdf]

Table S4
p-values of two-sided Welch's t-test for comparisons of FPKM values of TSGD duplicate genes in four cichlid species in brain, ovary and testis as shown in Figure 4

		**Gene**																		
**Tissue**	**Species**	*ar*	*cdc42*	*egfr*	*med13*	*pik3r1*	*pik3r2*	*pten*	*ptk2a*	*ptk2b*	*rac1a*	*rhoaa*	*rhoab*	*rock2*	*src*	*thrap3*	*arid1a*	*fhl2a*	*ncoa3*	*pias1*
Brain	*A. burtoni*	**0.00346**	0.05275	**0.00049**	**0.00000**	**0.00002**	**0.00256**	**0.01560**	0.13330	**0.01368**	**0.00001**	**0.00023**	**0.00023**	0.34635	**0.00033**	**0.00005**	**0.00484**	**0.00829**	**0.00065**	**0.01194**
Brain	*E. cyanostictus*	**0.00045**	**0.00008**	**0.00006**	**0.00001**	**0.00000**	**0.00035**	**0.00000**	0.10212	**0.01937**	**0.00000**	**0.00000**	**0.00300**	**0.00566**	**0.00000**	**0.00000**	**0.00019**	0.08535	**0.00006**	**0.00000**
Brain	*J. ornatus*	**0.01436**	**0.00000**	**0.00075**	**0.00000**	0.26959	**0.00000**	**0.00000**	0.66626	**0.00638**	**0.00000**	**0.00000**	**0.00073**	**0.00013**	**0.00002**	**0.00000**	0.21432	0.90236	**0.00000**	**0.00002**
Brain	*O. ventralis*	**0.00024**	**0.03156**	**0.00004**	**0.00003**	0.05098	0.16351	**0.00000**	**0.01268**	**0.02682**	**0.00000**	**0.00001**	**0.00246**	**0.04742**	**0.00113**	**0.00000**	**0.00024**	**0.00001**	**0.00000**	**0.00003**
Ovary	*A. burtoni*	0.40845	0.30001	0.29335	0.08093	**0.01684**	0.11070	**0.02884**	**0.02092**	0.32720	**0.01615**	0.99574	**0.03633**	0.06140	0.13777	0.06973	**0.03892**	0.16489	0.08868	0.17044
Ovary	*E. cyanostictus*	**0.03572**	**0.00000**	**0.01759**	0.14124	**0.00007**	0.08404	0.06825	0.24108	**0.01788**	**0.00001**	**0.00002**	**0.00000**	0.23874	**0.00018**	**0.00101**	**0.00005**	0.39100	**0.01284**	**0.00483**
Ovary	*J. ornatus*	0.20389	**0.00011**	**0.03002**	0.11506	**0.01300**	**0.03726**	**0.00926**	**0.02540**	0.66233	**0.00079**	**0.00016**	**0.00006**	0.47422	**0.00033**	**0.01942**	**0.00116**	**0.00561**	**0.00020**	0.10924
Ovary	*O. ventralis*	**0.01364**	**0.01686**	0.92110	0.44459	**0.00426**	0.28511	0.19111	**0.00001**	0.39317	**0.00114**	0.62893	**0.00186**	**0.00199**	**0.00903**	**0.00379**	**0.01364**	**0.01016**	**0.00651**	0.44459
Testis	*A. burtoni*	**0.01080**	**0.00154**	0.08909	**0.00005**	**0.00001**	**0.00759**	**0.00328**	**0.00055**	0.19324	**0.00073**	0.44422	**0.00701**	**0.00049**	**0.00013**	**0.00000**	**0.00023**	**0.00072**	**0.00029**	**0.01682**
Testis	*E. cyanostictus*	0.75190	**0.00007**	**0.00501**	0.29676	**0.02859**	**0.02680**	**0.02637**	**0.00001**	0.14388	**0.00009**	0.08819	0.05501	**0.00200**	**0.02596**	**0.00155**	0.52935	0.58034	**0.01598**	**0.02277**
Testis	*J. ornatus*	0.78731	**0.00002**	**0.00000**	**0.00006**	0.06754	**0.00616**	0.76850	**0.00885**	0.88815	**0.00181**	**0.00550**	**0.00681**	**0.00034**	0.50971	**0.00002**	**0.00341**	**0.01825**	**0.00189**	**0.00898**
Testis	*O. ventralis*	**0.02149**	**0.00885**	0.06161	0.06250	0.25395	**0.03823**	**0.03967**	**0.00582**	0.11568	**0.00258**	**0.00217**	0.07943	**0.00377**	0.68541	**0.00106**	**0.02149**	**0.00202**	**0.00002**	0.06250
